# Druggable transcriptomic pathways revealed in Parkinson’s patient-derived midbrain neurons

**DOI:** 10.1038/s41531-022-00400-0

**Published:** 2022-10-18

**Authors:** Mark van den Hurk, Shong Lau, Maria C. Marchetto, Jerome Mertens, Shani Stern, Olga Corti, Alexis Brice, Beate Winner, Jürgen Winkler, Fred H. Gage, Cedric Bardy

**Affiliations:** 1 South Australian Health and Medical Research Institute (SAHMRI), Laboratory for Human Neurophysiology and Genetics, Adelaide, SA Australia; 2 Laboratory of Genetics, Salk Institute for Biological Studies, La Jolla, CA USA; 3 Department of Anthropology, University of California San Diego, La Jolla, CA USA; 4 Neural Aging Laboratory, Institute of Molecular Biology, CMBI, Leopold-Franzens-University Innsbruck, Innsbruck, Tyrol Austria; 5 Sagol Department of Neurobiology, Faculty of Natural Sciences, University of Haifa, Haifa, Israel; 6 Sorbonne Université, Institut du Cerveau - Paris Brain Institute - ICM, Inserm, CNRS, AP-HP, Hôpital de la Pitié Salpêtrière, DMU BioGeM, Paris, France; 7 Department of Stem Cell Biology, University Hospital Erlangen, Friedrich-Alexander University Erlangen-Nürnberg (FAU), Erlangen, Germany; 8 Center of Rare Diseases Erlangen (ZSEER), University Hospital Erlangen, FAU Erlangen-Nürnberg, Erlangen, Germany; 9 Department of Molecular Neurology, University Hospital Erlangen, FAU Erlangen-Nürnberg, Erlangen, Germany; 10 Flinders Health and Medical Research Institute, Flinders University, Adelaide, SA Australia

**Keywords:** Parkinson's disease, Cellular neuroscience, Cellular signalling networks, Genomics, Induced pluripotent stem cells

## Abstract

Complex genetic predispositions accelerate the chronic degeneration of midbrain substantia nigra neurons in Parkinson’s disease (PD). Deciphering the human molecular makeup of PD pathophysiology can guide the discovery of therapeutics to slow the disease progression. However, insights from human postmortem brain studies only portray the latter stages of PD, and there is a lack of data surrounding molecular events preceding the neuronal loss in patients. We address this gap by identifying the gene dysregulation of live midbrain neurons reprogrammed in vitro from the skin cells of 42 individuals, including sporadic and familial PD patients and matched healthy controls. To minimize bias resulting from neuronal reprogramming and RNA-seq methods, we developed an analysis pipeline integrating PD transcriptomes from different RNA-seq datasets (unsorted and sorted bulk vs. single-cell and Patch-seq) and reprogramming strategies (induced pluripotency vs. direct conversion). This PD cohort’s transcriptome is enriched for human genes associated with known clinical phenotypes of PD, regulation of locomotion, bradykinesia and rigidity. Dysregulated gene expression emerges strongest in pathways underlying synaptic transmission, metabolism, intracellular trafficking, neural morphogenesis and cellular stress/immune responses. We confirmed a synaptic impairment with patch-clamping and identified pesticides and endoplasmic reticulum stressors as the most significant gene-chemical interactions in PD. Subsequently, we associated the PD transcriptomic profile with candidate pharmaceuticals in a large database and a registry of current clinical trials. This study highlights human transcriptomic pathways that can be targeted therapeutically before the irreversible neuronal loss. Furthermore, it demonstrates the preclinical relevance of unbiased large transcriptomic assays of reprogrammed patient neurons.

## Introduction

Parkinson’s disease (PD) is a chronic neurodegenerative disorder characterized by the selective loss of dopaminergic neurons in the midbrain substantia nigra pars compacta, among others^1^. Unfortunately, despite numerous research efforts over the last decades, no clinically approved treatment can slow the progression of PD pathophysiology. Furthermore, only a small proportion of active clinical trials currently focus on disease-modifying therapies (only 3/28 in Phase 3 in 2020)^2,3^. Therefore, there is an urgent unmet need for innovative therapeutic pipelines targeting the cellular dysfunction that precedes dopaminergic neuron loss. However, the early neuronal molecular dysfunctions in sporadic PD patients remain poorly determined^4,5^.

Mendelian genetic investigations and large-scale genome-wide association studies (GWAS) demonstrated the complex polygenic nature of PD, highlighting a broad range of genes and variants associated with PD clinical phenotypes^6–16^. However, individually most of these variants have minor effects and cannot be consistently associated with PD. As a result, it remains challenging to use GWAS findings to guide clinical practice or therapeutic development^17–19^. Nevertheless, the substantial role of genetic predispositions in PD is well recognized^18,20^ and, collectively, a large set of low-penetrance risk factors can become pathogenic. Thus, genome-wide expression profiling in patient neurons is required to portray a molecular picture that better represents the heterogeneity of PD gene variants. While post-mortem samples of bulk midbrain substantia nigra from PD and healthy subjects have been sequenced^21–23^, these case-control comparisons most likely reflect terminal cytoarchitectural differences rather than primary pathogenic mechanisms^24^. In contrast, human-induced pluripotent stem cell (hiPSC) models can address this technical gap by revealing the relatively early molecular stages of the disease in live neurons^25–27^.

Previous work has demonstrated the capability of hiPSC-derived neuronal models to reveal downstream cellular impairments caused by complex PD genetic predispositions (see, for review^20^). Since the first hiPSC PD report in 2011, >60 original peer-reviewed articles have used hiPSC-derived neurons from familial PD (e.g.^28–30^,) and >3 from sporadic PD^31–33^. Altogether, these studies show that, despite the diverse PD patient stratifications, common dysfunctional cellular pathways emerge^20^. However, all current reports from iPSC models of PD consist of hypothesis-driven projects with a relatively small number of subject lines tested (~3 controls and ~3 PD, on average)^20^. Breakthroughs and paradigm shifts are infrequent and often result from serendipity because the specific hypotheses that drive experimental designs evolve slowly from past results^34^. Instead, holistic omics analysis of patient-derived tissue can accelerate the discoveries of innovative therapeutic directions^5^. Recently, a few iPSC studies used RNA-seq to reveal cellular mechanisms underlying PD^31,32,35–38^. However, the number of individuals compared in each study remains relatively low (≤3 controls, ≤3 PD), and it is not clear whether these findings can be generalized to the broader PD community.

Here, we compared the transcriptome profiles of PD to healthy controls with midbrain neurons reprogrammed from donors’ fibroblasts from 42 individuals (25 healthy, 17 PD patients). We found that variance in neuronal reprogramming and RNA-seq methods could substantially bias transcriptome profiles. Therefore, we developed an analysis pipeline integrating a range of RNA-seq datasets (unsorted and sorted bulk vs. single-cell and Patch-seq) and neuronal reprogramming strategies (induced pluripotency vs. direct conversion). Gene expression signatures consistent across independent methods and datasets will arguably be more disease-relevant. Furthermore, notwithstanding the disproportionate clinical preponderance of sporadic PD over familial PD (8:1), the large majority of pre-clinical research focuses on the familial forms of the disease. Thus, we used the integrated analysis pipeline to identify a PD signature common to both genetic forms of PD (LRRK2, PARK2 and SNCA) and the sporadic form (broad range of low penetrance risk variants). Defining the molecular perturbations in PD patients’ midbrain neurons can help guide the design and evaluation of much-needed PD therapeutics. Identifying a molecular profile resistant to bias from RNA-seq, cellular reprogramming methods, and patient stratifications will maximize clinical relevance.

## Results

### Transcriptomic data of midbrain neurons reprogrammed from PD patients and healthy controls

**Fig. 1:**
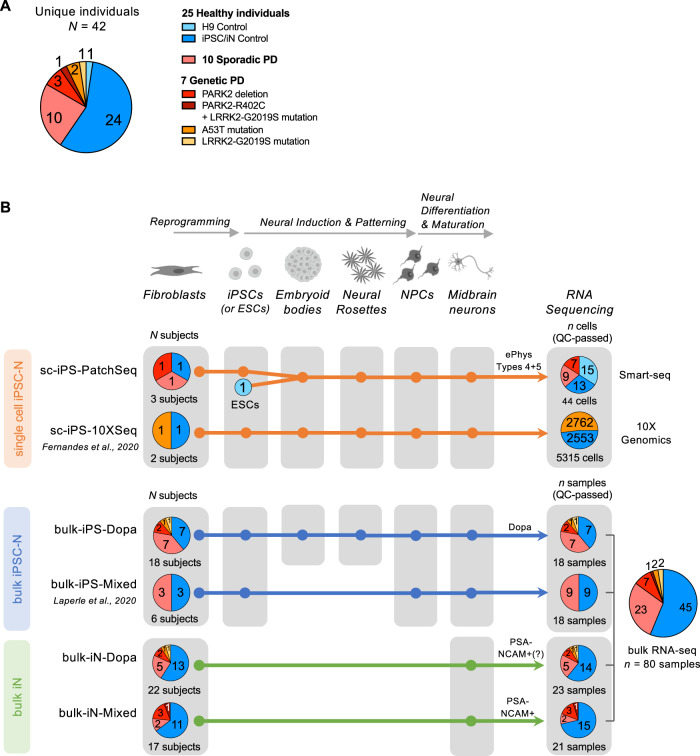
Patient and sample distributions of six transcriptomic datasets of Parkinson’s disease reprogrammed neurons. **A** The transcriptome data analyzed in the present study were collected across six different datasets and originate from the reprogrammed neurons of *n* = 25 healthy individuals, *n* = 7 patients with known mutations in PD genes, and *n* = 10 patients with a sporadic form of the disease (*n* = 42 unique subjects total). **B** Overview of tissue culture trajectories used in six reprogramming studies to generate in vitro midbrain-like neurons from PD patient- and healthy subject-derived fibroblasts (see Methods for details). Pie charts on the left summarize the number of subjects from which neurons were derived, color-coded by disease phenotype and mutation type as shown in (***A***). Neuron cultures were generated via induced pluripotent stem cell (iPSC) technology (*n* = 4 studies) or by direct conversion of fibroblasts into induced neurons (iNs, *n* = 2 studies), and neuronal transcriptome data was obtained by single-cell or bulk RNA-seq (*n* = 2 and 4 studies, respectively). The trajectory color reflects the combinatory choice of the reprogramming and sequenced method used (*orange*: single-cell iPSC neuron datasets; *blue*: bulk iPSC neuron datasets; *green*: bulk iN datasets). Single-cell RNA-seq was performed on (i) functionally mature (AP Types 4 + 5) single neurons collected after patch-clamp recording (PatchSeq, *n* = 44) or on (ii) wild-type and isogenic *SNCA-A53T* neurons harvested using 10X Chromium technology^38^. The two iN datasets enriched for successfully reprogrammed (i.e., PSA-NCAM-positive) neurons using fluorescence-activated cell sorting (FACS). The bulk-iPS-Dopa and bulk-iN-Dopa studies used an optimized differentiation protocol to generate a high proportion of midbrain dopaminergic neurons. Pie charts on the right indicate the number of cells or samples sequenced per disease state and/or mutation type. A total of 5,359 single neurons and 80 neuronal bulk samples were analyzed.

We investigated the molecular mechanisms dysregulated in PD through transcriptomic analysis of human midbrain neurons reprogrammed from the fibroblasts of a large and diverse cohort of 42 individuals (Fig. 1; Supplementary Table 1). Our cohort comprises 10 sporadic PD patients (including four young onset, <40 years old), 7 familial PD patients with mendelian variants in PD genes (*PARK2*, *LRRK2*, *SNCA*), and 25 matched healthy subjects. We analysed a total of 5,359 single cells and 80 bulk RNA-seq samples. These raw sequencing data were combined from three unpublished transcriptomic datasets (sc-iPS-PatchSeq, bulk-iPS-Dopa, bulk-iN-Dopa) and three recently published datasets (sc-iPS-10XSeq^38^, bulk-iPS-Mixed^31^, bulk-iN-Mixed^39^). The midbrain neuronal cells were derived from fibroblasts using a range of reprogramming strategies (Sendai viral, retroviral or episomal) and neural differentiation protocols (induced pluripotency or direct conversion) (Fig. 1B). Half of the bulk transcriptomic samples *(n* = 44/80) originated from reprogrammed induced neurons (iNs) generated using two different conversion protocols (bulk-iN-Dopa and bulk-iN-Mixed; see Methods for details). The other half (*n* = 36/80) were derived from iPSCs using either an embryoid body-based (bulk-iPS-Dopa) or monolayer-based (bulk-iPS-Mixed) neural induction. The bulk-iPS-Dopa and bulk-iN-Dopa datasets were optimized to generate higher proportions of dopaminergic neurons (see Methods for details). The single-cell RNA-seq data were collected either with Patch-seq, from electrophysiologically mature neurons (AP Types 4 + 5 as previously described in^40,41^) (sc-iPS-PatchSeq), or with the high-throughput 10X Genomics Chromium system^38^ (sc-iPS-10XSeq). A subset of individuals (*N* = 17) was selected to generate both iPSC and iN models. Another subset of neuronal lines (*n* = 3) was profiled using both single-cell and bulk RNA-sequencing (Supplementary Table 1).

### Sample inclusion criteria for RNA-seq datasets

Quality assessment at the level of the individual sample (bulk RNA-seq) or cell (single-cell RNA-seq) is an essential step in identifying outliers, filtering out spurious samples, and optimizing the reproducibility of biologically relevant results. We excluded cells or samples with low read mapping, a low number of detected genes, and a low number of expressed housekeeping genes (Supplementary Fig. 1; see Methods for details). The average read mapping rate across all 80 bulk transcriptomes was 85%, with 78 of 80 libraries showing >75% alignment, indicating the high quality of these samples. We also quantified the number of total genes and housekeeping genes detected and excluded any sample with <1000 genes detected or with less than 65 housekeeping genes expressed (out of a curated list of 98 housekeeping genes^42^; Supplementary Table 2). For single-cell transcriptome data, we only retained cells with an overall read alignment (mapping) rate of at least 50%^43^ (Supplementary Fig. 1). In addition, to exclude potential cell doublets or multiplets from single-cell analyses, we removed cells with exceptionally high gene or UMI detection (i.e., >8000 genes or >37,500 unique molecular identifiers detected). Quality control metrics for each sample are detailed in Supplementary Table 3. Published PD iPSC datasets that failed to pass these quality control criteria were not included in this study.

### Neuronal reprogramming methods sway the overall transcriptomic profiles

**Fig. 2:**
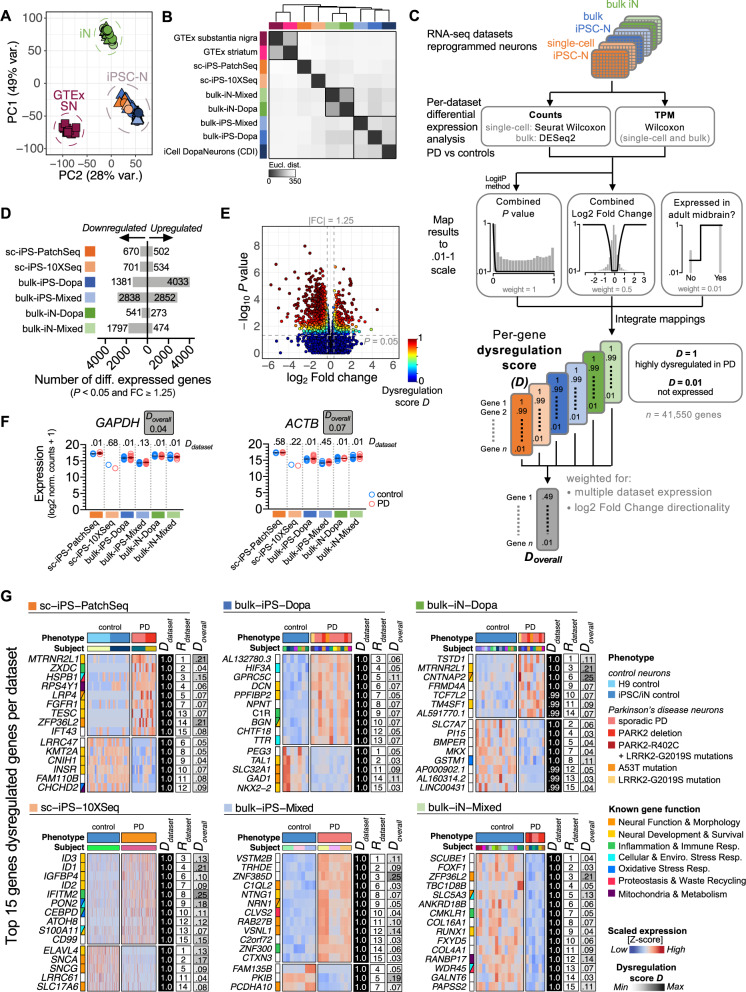
Identification, scoring and ranking of genes dysregulated in Parkinson’s disease reprogrammed neurons. **A** Principal component analysis (PCA) of the transcriptome data from PD patient- and control- reprogrammed neurons collected across six datasets, post-mortem adult human substantia nigra samples (GTEx), and highly pure populations of human floor plate-derived midbrain dopaminergic neurons (iCell® DopaNeurons). In vitro-engineered neuronal tissue clusters separately from post-mortem substantia nigra tissue (GTEx) and groups by method of derivation (iPSC reprogramming or direct iN conversion) irrespective of the laboratory of origin. Each data point represents a bulk or artificial bulk (for single-cell RNA-seq datasets) tissue transcriptome. Artificial bulk samples were generated by summing up the gene counts from all cells of the same subject. Color indicates dataset of origin (annotation shown in (***B***)). **B** Heatmap clustering of the average transcriptomes of the six reprogrammed neuron datasets, GTEx substantia nigra and striatum tissues, and iCell® DopaNeurons using all expressed genes (≥1 transcript per million [TPM] across all samples). Hierarchical clustering is based on average linkage and Euclidean distance-based similarity. The darker shade denotes higher similarity. **C** Pipeline for computation of a per-gene dysregulation score (*D*) based on individual-dataset differential expression (DE) analysis results. DE analysis was performed on each dataset independently on both read counts and TPM values, and results were combined using logitp method (for a combination of *P*-values) and arithmetic mean averaging (for a combination of log2 fold changes). Combined *P*-value and log2 fold change measures were mapped to a continuous (0.01-1) scale using desirability functions^48^, and integrated by weighted geometric averaging to obtain an overall dysregulation score (*D*) for each gene. Information about whether the gene was expressed in the adult human midbrain was used as a soft filter at 1% of the total weight to prioritize the ranking of relevant genes. Dysregulation scores are integrated across multiple datasets, weighting for cross-dataset similarity in log2 fold change directionality, to obtain an overall dysregulation score per gene (D_overall_; refer to Methods for details). **D** Number of genes (*P* < 0.05 and |fold change | ≥ 1.25) up- and downregulated in each dataset of PD versus healthy control reprogrammed neurons. **E** Volcano plot of differentially expressed genes between PD and control reprogrammed neurons (shown for dataset bulk-iN-Mixed). Genes with greater statistical significance and/or greater fold change in expression have a larger dysregulation score (*D*; color-coded in plot). **F** Dataset expression levels of housekeeping genes *GAPDH* and *ACTB* are very similar between patients and controls and are associated with a low overall (multiple-dataset) dysregulation score. **G** Relative expression levels of the top 15 differentially expressed genes in PD versus control neurons in each of the six analyzed RNA-seq datasets. Each heatmap cell represents a single cell (single-cell datasets) or bulk tissue sample (bulk datasets). Disease phenotype and subject ID are annotated horizontally, and gene function is annotated vertically according to the legend. A subset of individuals (*N* = 17) was selected to generate both IPSC and iN models, and three neuronal lines were profiled using both single-cell and bulk RNA-sequencing (see Supplementary Table 1 for subject details). For each gene, the dataset-specific dysregulation score and rank, as well as the overall dysregulation score calculated across all six datasets, are shown on the right with dark intensity indicating the strength of the score.

To identify the main sources of variation between transcriptomic samples, we compared the datasets with hierarchical clustering and principal components analysis (PCA). To compare single-cell and bulk RNA-seq samples, we generated artificial bulk transcriptomes for the Patch-seq and the 10X Chromium datasets (sc-iPS-PatchSeq, sc-iPS-10XSeq) by summing up the read counts across all cells sequenced from each subject. We then performed PCA clustering with the transcriptomes of 86 (*n* = 80 bulk and six artificial single-cell bulk) reprogrammed neuronal samples that passed all quality control (QC), 19 postmortem adult human substantia nigra samples from the Gene-Tissue Expression (GTEx) project, and four samples of commercially available iPSC-derived human midbrain floorplate dopaminergic neurons (iCell DopaNeurons, Fujifilm Cellular Dynamics). This analysis revealed a distinct clustering of samples based on the derivation method independently of disease status (i.e., neurons generated from iPSCs clustered separately from neurons generated by direct conversion (iNs)) (Fig. 2A). Hierarchical clustering analysis based on Euclidean distance-based similarity revealed that GTEx adult substantia nigra and striatum tissue grouped into a distinct cluster separate from in vitro-engineered datasets (Fig. 2B), suggesting a combination of technical and endogenous differences between post-mortem adult brain tissue and live in vitro-engineered neuronal tissue. Nevertheless, the Euclidean distance with adult brain tissue was relatively small overall, highlighting the quality of in vitro*-*engineered tissues despite their limitations^20^. Altogether, our study shows that the technical variance resulting from the methods used to obtain the neuronal tissue (post-mortem vs. iPSC-derived vs. direct conversion) biases the transcriptomic profiles and outweighs patient differences (PD vs controls) or RNA-seq method biases (single-cell vs bulk).

### Scoring differential mRNA expression in PD within unique transcriptomics datasets

Identifying the molecular differences between bioengineered midbrain neurons from PD and healthy controls may provide valuable mechanistic insights into the early stages of PD pathogenesis (prior to neurodegeneration). First, we performed differential gene expression testing between PD and matched control samples in each independent dataset, as outlined in Fig. 1. However, the choice of bioinformatic methods for transcriptomics analysis has not reached an unequivocal consensus yet, and the poor concordance between differential expression results across methods remains a challenge^44–47^. For example, using either raw read counts or normalized expression values (e.g., TPM/CPM), or simply choosing different statistical analysis packages can lead to substantially different results from the same dataset. Furthermore, differential gene expression calls that are exclusively based on arbitrary binary cut-offs of *P*-value (significant/non-significant) and/or fold-change lead to loss of information, bias, and lower statistical power^48–50^. Therefore, rather than only relying on single *P*-values for inferring each gene’s significance in PD, we implemented an analytical pipeline to determine multivariate gene dysregulation scores. In particular, we used both raw counts and normalized TPM expression values as input and combined the log2 fold changes and *P*-values from multiple statistical tests (logit method). The combined *P*-values and log_2_ fold-changes were mapped to a continuous scale from 0.01-1 using non-binary desirability functions^48^ and combined by weighted averaging to obtain a *dysregulation score* (*D*) for each gene. We also adjusted the scores with weighted functions for statistical significance (*P-*value), the magnitude of fold change, and the relevance of the gene in the adult midbrain in vivo (Fig. 2C, see also Methods for details). The genes with the highest *D score* (*max* = 1) were most strongly differentially expressed in PD relative to controls, and genes with the lowest *D score* (*min* = 0.01) were not expressed or not dysregulated. Information on the genes’ expression detection in adult human midbrain was used as a soft adjustment variable at 1% of the total weight to slightly prioritize genes that are expressed in adult midbrain tissue. All in all, the dataset-specific dysregulation *D score* identified midbrain genes with the best combination of significance (low *P*-value) and expression change (high fold-change) (Fig. 2D, E).

Among the top dysregulated genes in each dataset (highest *D*_*dataset*_ *scores*), we identified a substantial number of genes with functions related to neuronal function, morphology and survival (Fig. 2G). A subset of these genes has been previously implicated in PD [e.g., *SNCA*^9,51^, *CHCHD2*^6,52^, *GAD1*^53^, *SLC17A6/VGLUT2*^54^]. However, despite two common genes within the top 15 genes list across datasets (*MTRNRL1* and *ZFP36L2*), and some overlap in gene families and associated cellular functions, most of the top 15 genes appeared unique to each dataset. Therefore, we next aimed to identify the most common molecular profile across all these datasets.

### Integrated scoring of differential mRNA expression across independent transcriptomics datasets

The genes consistently dysregulated across multiple independent transcriptomic datasets may be particularly implicated in initiating PD pathology and overt symptoms. Therefore, to identify such a molecular phenotype, we developed a statistical analysis framework that determines a single overall dysregulation score (*D*_*overall*_) per gene across multiple transcriptomic datasets generated by different researchers, methods, and conditions (Fig. 2C). *D*_*overall*_ is calculated from the gene dysregulation scores from all six datasets (*D*_*datasets*_) and adjusted for the degree of concordance in the fold-change direction to prioritize the genes changing expression in the same direction (up- or down-regulated) across datasets (see Methods for details). As a result, genes with similar expression patterns between PD and healthy neurons across datasets display a low *D*_*overall*_ score (e.g. housekeeping genes, *GAPDH*: 0.04; *ACTB*: 0.07) (Fig. 2F). In contrast, a higher *D*_*overall*_ score indicates a greater gene transcript dysregulation in PD relative to controls, consistently across all or most datasets.

### Genes consistently dysregulated in PD patients across independent datasets

**Fig. 3:**
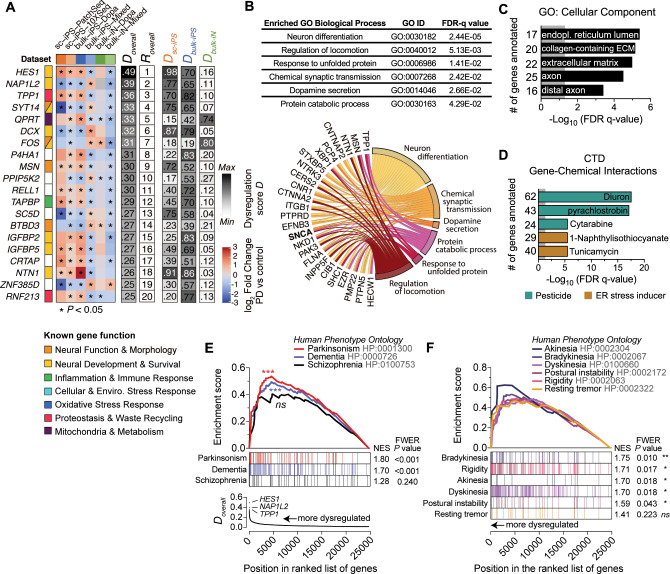
Integrative analysis of multiple transcriptomics datasets reveal Parkinson’s disease-related gene signatures and biological processes in patient-reprogrammed neurons. **A** Heatmap of mean log2 fold changes in expression of the top 20 genes with the highest dysregulation across all six datasets of PD versus control reprogrammed neurons. Red and blue indicate, respectively, up- and downregulation in PD cells relative to control. Gene function and overall dysregulation score (*D*_*overall*_) are annotated vertically according to the legend. *D*_*sc-iPS*_, *D*_*bulk-iPS*_ and *D*_*bulk-iN*_ scores indicate the strength of dysregulation for, respectively, the two single-cell iPSC neuron datasets (*orange*), the two bulk iPSC neuron datasets (*blue*) and the two bulk iN datasets (*green*) (see Methods for details). *, nominal *P*_*combined*_ < 0.05. **B**, **C**, **D** ToppFun functional enrichment analysis of the 200 most highly dysregulated genes across all datasets highlights fundamental biological processes (**B**), cellular components (**C**) and chemicals (**D**) implicated or suspected to be involved in PD. Chord plot in (**B**) indicates genes annotated to dysregulated GO biological processes, ordered by decreasing overall dysregulation score. Length of bars in (**C, D**) represent Benjamini-Hochberg-corrected significance values and numbers indicate number of genes annotated to GO term; grey color indicates significance threshold. Extended data in Supplementary Tables 4 and 5. **E** Gene Set Enrichment Analysis (GSEA) of multiple-dataset PD dysregulated genes for genes associated with the terms “Parkinsonism”, “Dementia” and “Schizophrenia” from the Human Phenotype Ontology (HPO) database^131^. “Parkinsonism”- and “Dementia”-related genes were significantly overrepresented at the top of the list of expressed genes (*n* = 24,693) rank-ordered by decreasing *D*_*overall*_ score. **F** GSEA shows a significant enrichment of PD-symptom-related HPO terms “Bradykinesia”, “Rigidity”, “Akinesia”, “Dyskinesia” and “Postural instability” among the multiple-dataset PD dysregulated genes. Vertical bars in (**E, F**) represent the “gene hits”, i.e., the location of genes from each indicated HPO term within the *D*_*overall*_ rank-ordered list. *P < 0.05; **P < 0.01; ***P < 0.001; ****P < 0.0001; ns, not significant (*P* > 0.05); all *P*-values are FWER-corrected to exclude any possibility of false-positive enrichment. NES Normalized Enrichment Score. Detailed enrichment results are provided in Supplementary Table 6.

We rank-ordered the entire list of genes (*n* = 24,693 unique genes expressed in ≥1 dataset) by decreasing D_overall_ score. Among the 20 genes with the highest D_overall_ score, 13 were significantly dysregulated in five out of six independent datasets and *HES1* and *NAP1L2* ranked the highest (Fig. 3A). *HES1* plays an important role in regulating the location and density of mesencephalic dopaminergic neurons^55^. *NAP1L2* appears important for regulating histone acetylation during neuronal differentiation^56^. *NAP1L2* expression was also found to be down-regulated in adult post-mortem brain tissue from a large multi-cohort of patients (*n* = 1,270) with a range of neurodegenerative diseases^57^, suggesting a broad role for NAP1L2 in neurodegeneration.

Gene Ontology (GO) analysis of the top 200 highest-ranked genes revealed significant enrichment for biological processes related to neuronal differentiation and synaptic transmission, and several PD-related biological processes, including dopamine secretion, locomotion, cellular stress response and proteostasis (Fig. 3B and Supplementary Table 4). The endoplasmic reticulum (ER), extracellular matrix and (distal) axon were identified as the major enriched cellular components (Fig. 3C), all of which have well-established roles in the pathogenesis of PD as well as several other neurodegenerative disorders^58–66^.

Overrepresentation analysis for gene-chemical interactions from the Comparative Toxicogenomics Database (CTD)^67^ uncovered significant associations between our cross-dataset integrated molecular signature and a variety of environmental toxins (i.e., pesticides) and ER stress inducers (Fig. 3D and Supplementary Table 5). Exposure to pesticides, most notably rotenone, has been shown to trigger selective degeneration of dopaminergic neurons in the substantia nigra and is a well-recognized risk factor for the onset and development of PD^68–72^.

### Dysregulated genes in patient-derived midbrain cells associate with parkinsonism symptoms ontology

Parkinsonism is a human phenotype, which is defined typically by shaking, rigidity, slowness of movement and difficulty with walking and gait. Global genomic consortiums (e.g., “Human Phenotype Ontology”, “Gene Ontology”) have associated parkinsonism and other human phenotypes with compilations of genes based on the meta-analysis of large genetic studies. Using Gene Set Enrichment Analysis (GSEA), we tested whether genes known to be related to parkinsonism and other human disease phenotypes were significantly overrepresented among our top-ranked dysregulated genes (high D_overall_ score) (Fig. 3E, F). Notably, GSEA revealed statistical enrichment for parkinsonism- and dementia-related genes (both FWER *P*-values *<* 0.001) but not for schizophrenia (FWER *P*-value = 0.240, Kolmogorov-Smirnov test) (Fig. 3E). Schizophrenia is a neuropsychiatric disorder that, like PD, is also characterized by dopaminergic dysfunction within nigrostriatal pathways^73^, but with very different symptoms overall. The significant enrichment for both parkinsonism and dementia phenotypes is perhaps not surprising given the considerable gene overlap between these disease conditions (Supplementary Fig. 2) and the fact that an estimated 20-80% of people with PD will develop dementia during the course of the disease^74–77^. Alpha-synuclein (*SNCA*) was the top gene candidate in our integrated analysis of reprogrammed neurons with the highest contribution to parkinsonism-related enrichment. Synaptojanin 1 (*SYNJ1*) and phosphodiesterase 10 A (*PDE10A*) were the second and third top enriched genes, both of which play an essential role in the regulation of neuronal synaptic function in the context of PD^78–81^. Further GSEA analyses revealed a significant association of our multiple-dataset dysregulated genes with genes related to key PD clinical symptoms, including bradykinesia, rigidity, akinesia, dyskinesia and postural instability. (Fig. 3F and Supplementary Table 6). Genes associated with resting tremors were not significantly enriched; however, it should be noted that this early and cardinal symptom of PD is absent in ~30% of patients^82^. Importantly, the associations with parkinsonism, dementia and PD-like phenotypes were completely abolished when the gene-dysregulation score relationship was eliminated by random permutation of the gene labels (Supplementary Fig. 2). Taken together, our integrative analysis of multiple datasets of PD patient-reprogrammed neurons reveals a gene expression signature that captures known phenotypic features of the disease.

### Perturbed PD transcriptomic pathways integrated across datasets

**Fig. 4:**
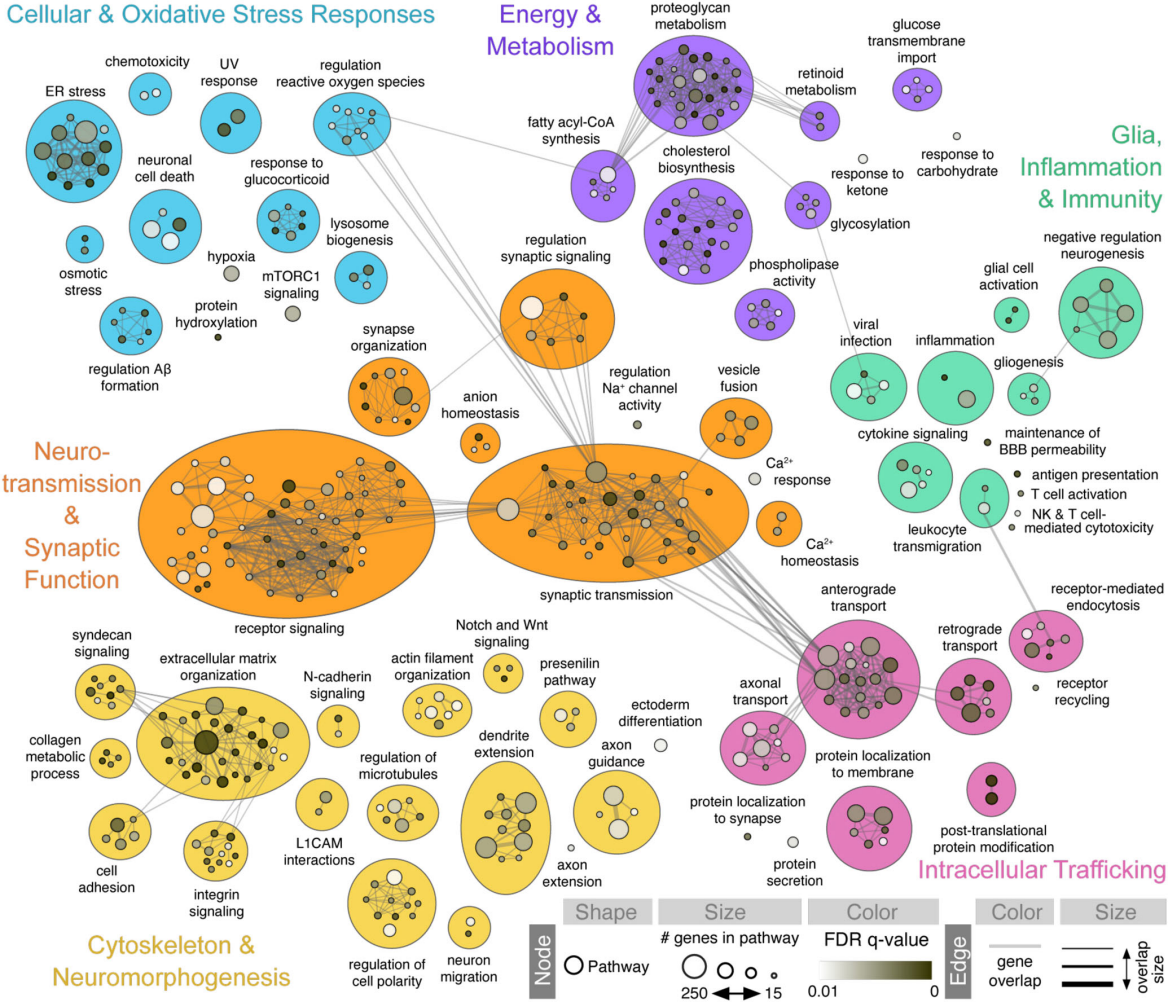
Gene Set Enrichment Analysis identifies transcriptomic pathways perturbed in Parkinson’s patient-derived neurons. Enrichment map representation of the main biological processes dysregulated in PD versus control reprogrammed neurons. Pathway gene sets (*n* = 5654 passing size filters) corresponding to gene ontology (GO) biological process terms were tested for enrichment by Gene Set Enrichment Analysis (GSEA)^133^ following a recent protocol^83^. GSEA revealed significant (FDR-*q* < 0.01) overrepresentation of 579 pathway gene sets at the top of the list of expressed genes (*n* = 24,693) rank-ordered by decreasing overall dysregulation score (*D*_*overall*_, genes with highest dysregulation across all studies at top). Functionally related pathway sets were assigned a label and grouped together based on similarity, resulting in 408 dysregulated pathways related to six main biological themes, including (i) neurotransmission and synaptic function, (ii) cytoskeleton and neuromorphogenesis, (iii) cellular and oxidative stress responses, (iv) energy and metabolism, (v) glia, inflammation and immunity, and (vi) intracellular trafficking. Node size is proportional to the number of genes in the pathway gene set, and node color intensity indicates the statistical significance of GO term enrichment. Edge thickness represents the degree of gene overlap between connected biological pathways. Detailed pathway enrichment results are provided in Supplementary Table 7.

A broad range of cellular functions may be impaired in PD. Therapeutically, it remains challenging to predict if one drug targeting a specific pathway in a rare genetic type of PD (e.g., LRRK2-G2019S) will also stop the pathology in other pathways and across the broad spectrum of PD patients. Pre-clinical transcriptomics analysis of bioengineered neurons from patients’ cells is attractive to advance this issue because it can assess the dysregulation of all pathways at once in live human cells with minimal bias. Therefore, we aimed to identify the biological pathways that are overrepresented among the dysregulated transcripts in PD midbrain cells integrated across datasets. First, we performed a pathway enrichment analysis with GSEA^83^. This analysis revealed significant (Benjamini-Hochberg FDR-q < 0.01) enrichment of 579 pathway gene sets, 408 (70%) of which could be annotated to one of six main biological/functional themes with previously implicated roles in PD pathogenesis (Fig. 4 and Supplementary Table 7). “Neurotransmission and synaptic function” was the major enriched biological theme, comprised of 118 pathways with functions in synaptic transmission, ion channel regulation, neuronal excitability, and intracellular signalling. Other enriched pathways were related to cytoskeleton and neuromorphogenesis, energy and metabolism, cellular and oxidative stress responses, inflammation and immunity, and intracellular trafficking (Fig. 4), i.e., all processes which are known or have been proposed to be implicated in PD^20,84–89^. Notably, abolishment of the original gene label–*D*_*overall*_ score relationship by random permutation of gene labels resulted in zero pathways that reached significance, indicating the methodological robustness of our integration approach and the biological specificity of our findings. Taken together, these results reveal disease-relevant biology captured in human reprogrammed neurons. These results also suggest the convergence of various genetic predispositions on the dysregulation of cellular processes related to synaptic communication, metabolic function, inflammation and cellular waste recycling.

### Synaptic dysfunction of PD patient reprogrammed neurons

**Fig. 5:**
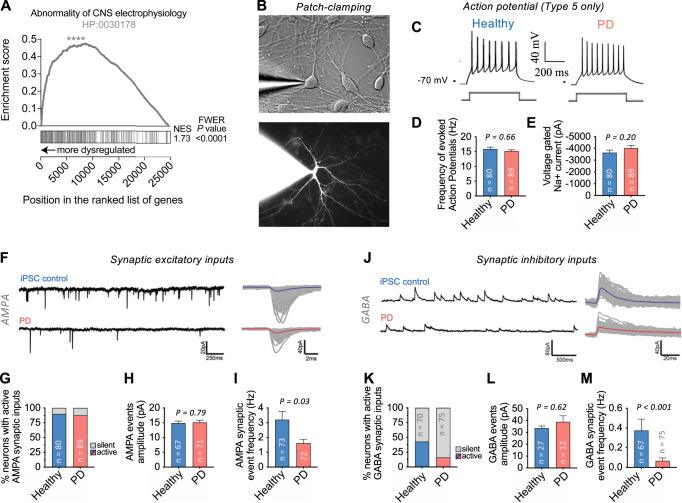
Synaptic impairments in PD patient reprogrammed neurons. **A** GSEA shows significant enrichment (FDR-*q* < 0.0001) of the Human Phenotype Ontology gene set for “abnormality of central nervous system electrophysiology” (HP:0030178) among the genes dysregulated in PD patient-derived neurons. **B** Example image of a typical neuronal culture used for patch-clamping electrophysiology in DIC (top image) or filled with Rhodamine (bottom image). Cells with characteristic neuronal morphology and brightest Synapsin:GFP expression were selected for patch-clamp recordings after a minimum of three weeks (average 43 days) of maturation in BrainPhys™ neuronal medium. Cells were patched from a total of 76 coverslips. **C, D** All patch-clamped neurons included in the analysis (*n* = 80 healthy subject-derived, *n* = 89 PD patient-derived) were classified as “Type 5” cells^40^, indicating equivalent functional maturity (see Methods for details). **C** Typical evoked action potential (AP) traces from PD patient and control-derived neurons following a 500-ms depolarizing current step. **D** The maximum firing frequency of evoked APs with amplitudes above -10 mV was similar between PD and control neurons. **E** Voltage-dependent sodium current characteristics were similar between PD and control neurons. **F**–**M** Synaptic properties of patch-clamped midbrain neurons from PD and healthy controls. **F** Typical recordings of excitatory postsynaptic synaptic currents mediated by AMPA receptors (left) and superimposed detected events and average trace (right). Data are presented as mean ± SEM. *P* values determined via nonparametric Mann-Whitney test (two-tailed, unpaired).

Pathways involved in neurotransmission and synaptic function comprised the largest group of pathways transcriptomically dysregulated across all the datasets (Fig. 4). We also observed a significant overrepresentation of genes related to ontology linked to “abnormal central nervous system electrophysiology” among the genes with the highest dysregulation in PD (top ranked D_overall_ score) (Fig. 5A). We confirmed the synaptic dysfunction with patch-clamping by comparing the electrophysiological and synaptic properties of 169 “mature” (Type 5 definition based on^40^) neurons from a subset of patients (*n* = 2) and controls (*n* = 2) (Fig. 5B–M). We selected for patch-clamping the cells with typical neuronal morphology and evident synapsin:GFP expression following lentiviral vector transduction after maturation in BrainPhys neuronal medium (Fig. 5B). We measured evoked action potentials (in current-clamp) and voltage-dependent sodium/potassium currents (in voltage-clamp) at an imposed resting membrane potential of −70 mV by applying a series of incremental depolarizing steps of current/voltage (Fig. 5C). Our results showed no difference between PD patient- and healthy subject-derived neurons in their ability to fire action potentials (Fig. 5D). Similarly, no significant difference was found in voltage-gated sodium (Nav) currents (Fig. 5E). These results demonstrate the homogeneity of our samples and suggest no major impairment in PD iPSC-derived neurons’ ability to generate healthy action potential trains. We then investigated the dendritic summation of excitatory and inhibitory synaptic events, which is essential for shaping the input/output activity of neural networks. In the human brain, synaptic excitation is mainly mediated by the neurotransmitter glutamate, whereas gamma-aminobutyric acid (GABA) is the primary neurotransmitter for inhibitory synaptic transmission. Hence, we examined whether PD iPSC-derived neurons were able to sustain excitatory (glutamatergic) and inhibitory (GABAergic) basic synaptic functions. Spontaneous glutamatergic and GABAergic synaptic events were distinguished by voltage clamping at the reversal potential of anions (−70 mV) and cations (0 mV) and were further confirmed with reversible blockade with the receptor antagonist 2,3-dioxo-6-nitro-7-sulfamoyl-benzo[f]quinoxaline (NBQX; to block AMPA receptors) or SR-95531 (gabazine; to block GABA_A_ receptors). Over 90% of the neurons analysed displayed spontaneously active AMPA-mediated excitatory synaptic inputs in both PD and healthy groups (Fig. 5G). The average amplitudes of the synaptic events recorded were also similar in both groups (~15 pA for AMPA and ~30pA for GABA) (Fig. 5H, L). However, the frequency of the excitatory synaptic events was significantly reduced by half in the PD group (Fig. 5I). The proportion of cells receiving active synaptic inhibitory inputs was lower by ~3 fold in PD (Fig. 5K) and the frequency of the inhibitory synaptic events was significantly reduced by ~5 fold in the PD group (Fig. 5M). Altogether, these results reinforce our molecular findings suggesting an essential, relatively early role of synaptic dysfunction in PD pathology. However, we also observed significant dysregulation of other pathways, such as bioenergetics, intracellular trafficking and cellular stress responses, which are critical for effective synaptic function. Therefore, we do not know if the synaptic dysfunction occurs upstream or downstream of the pathology. Nevertheless, these results demonstrate the value of transcriptomics assays on reprogrammed neurons to assess the health of intrinsically connected cellular pathways and further implicate synaptic dysfunction in PD pathology driven by genetic predispositions.

### Prediction of disease-modifying therapeutics for PD based on perturbed transcriptomic pathways

**Fig. 6:**
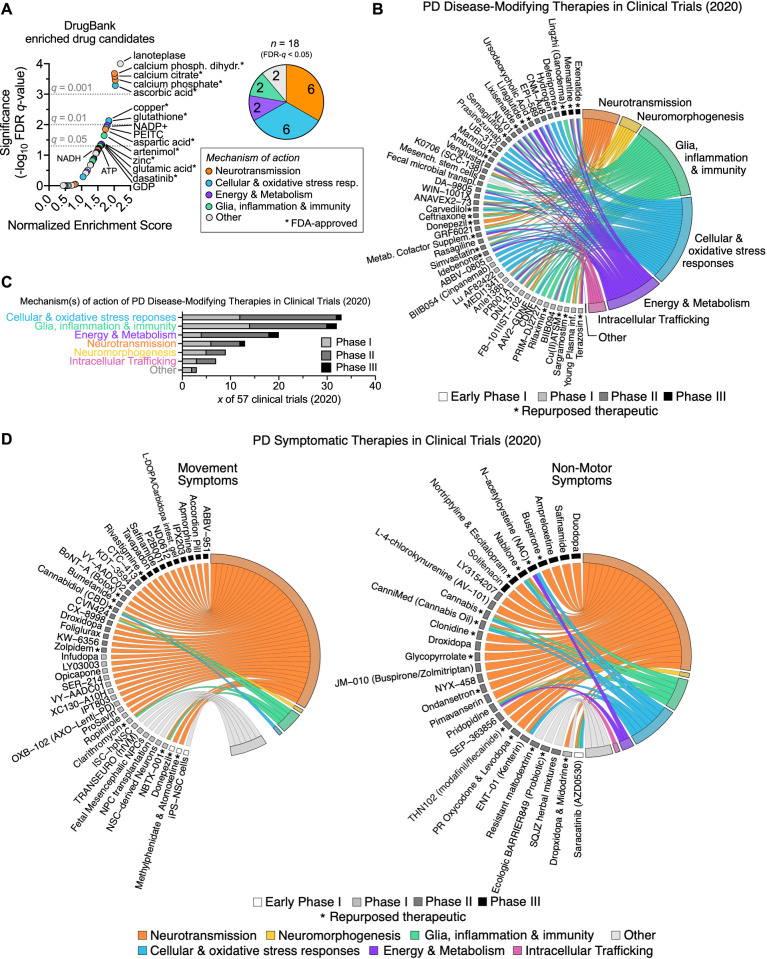
Prediction of therapeutics for Parkinson’s disease based on dysregulated transcriptomic pathways. **A** GSEA against DrugBank’s database of drug-target gene associations^90^ (*n* = 5,724 gene sets) identifies 18 therapeutic drugs, each associated with a minimum of 15 target genes, that are significantly (FDR-*q* < 0.05) overrepresented among PD-dysregulated genes. The top 15 enriched drug candidates with the highest significance and enrichment score are annotated on the right, with FDA-approved drugs indicated by asterisks. The color code indicates each drug’s main mechanism of action in relation to the major biological themes dysregulated in PD reprogrammed neurons (Fig. 5). **B, D**. Chord plots of disease-modifying (**B**) and symptomatic (**D**) PD clinical trial therapies in 2020 (identified by^2^, left semicircle) and their involvement in dysregulated transcriptomic pathways in PD (right semicircle). Therapies were filtered for redundancy and are grouped by clinical trial phase (see legend). Asterisks indicate repurposed therapeutics. Symptomatic therapies in (**D**) are categorized into those targeting movement symptoms (*left*) and those targeting non-motor symptoms (*right*). **C, E**. The number of disease-modifying (**C**) and symptomatic (**E**) clinical trials targeting the various dysregulated molecular pathways implicated in PD, subcategorized by clinical trial phase.

All current PD treatments pivot on restoring dopamine levels in the brain to reduce the severity of symptoms. PD is a chronic neurodegenerative disorder. However, no available treatments can halt or slow the accelerated cellular loss. To identify potential drug candidates based on the PD transcriptional signature from the reprogrammed neurons, we performed a GSEA of the ranked list of dysregulated genes against DrugBank’s database of 5,724 drug-target gene associations^90^. Our analysis revealed significant enrichment for 18 therapeutic drug candidates, most of which have established or predicted roles in the regulation of neurotransmission and cellular/oxidative stress responses (Fig. 6A). Two enriched candidates (i.e., glutamate and aspartate) directly function as neurotransmitters. Several other candidates (i.e., calcium phosphate dihydrate, calcium citrate, calcium phosphate, copper and zinc) affect the levels of calcium and metal ions, which can modulate neurotransmitter action, release and metabolism through their interaction with ion channels and synaptic vesicles^91–96^. Calcium ions are essential for the regulation of both synaptic vesicle exocytosis and endocytosis, as well as some forms of synaptic plasticity^97–100^. Notably, the chemicals affecting calcium levels accounted for three of the five candidates with the highest enrichment scores. We also identified robust and significant enrichment for several antioxidant molecules, including ascorbic acid, glutathione and phenyl-ethyl isothiocyanate (PEITC), all of which can mitigate the oxidative stress resulting from mitochondrial dysfunction in the context of PD (ascorbic acid:^101–103^; glutathione:^104,105^; isothiocyanate:^106,107^). Surprisingly, Lanoteplase, a third-generation thrombolytic agent used to treat acute myocardial infarction^108^, was the candidate with the strongest and most significant enrichment. Future studies should investigate whether and how this new drug could be beneficial in treating PD. Although some of these drugs may not be suitable in clinics, they could be substituted with more clinically relevant candidates with similar mechanisms of action, some of which are already being evaluated in clinical trials.

### Therapeutics in PD clinical trials align with dysregulated pathways in PD reprogrammed midbrain neurons

A recent meta-analysis review identified 145 registered and active clinical trials for therapeutics for PD^2^. The majority of these trials (*n* = 88, 61%) focus on symptomatic relief; the remaining (*n* = 57, 39%) employ a disease-modifying strategy. We performed a literature search for each therapeutic and linked its molecular function(s) to the transcriptomic pathway(s) identified as dysregulated in iPSC-derived PD neurons (Fig. 6B–D). This analysis revealed that most clinical trials for disease-modifying therapies attempt to combat cellular and oxidative stress by targeting alpha-synuclein and clearing reactive oxygen species, reducing inflammation or increasing mitochondrial function (Fig. 6B, C). Overall, our data further support the rationale of these current clinical trials that target dysregulated pathways across our PD transcriptomics datasets. In particular, our observation that synaptic transmission is dysregulated at both the transcriptional and functional levels in PD reprogrammed neurons further reinforces the need for clinical trials aiming to improve neuronal synaptic function. Although neurosynaptic interventions represent the vast majority of symptomatic therapies (Fig. 6D), currently, they might be under-appreciated as potential disease-modifying treatments (Fig. 6B).

## Discussion

We investigated the transcriptomic signature of Parkinson’s disease with reprogrammed midbrain neurons from 42 individuals (80 bulk, 5,315 single-cell RNA-seq and 44 Patch-seq samples). The success of patient-derived stem cell models relies on the presence of genetic predispositions conserved throughout cellular reprogramming. Parkinsonism is increasingly described as a spectrum disorder originating from complex interactions between genetic predispositions, environmental stressors and aging. However, reliable and clinically relevant stratifications of PD patients remain limited. Currently, the best stratification relies on single nucleotide polymorphisms (SNPs) of a handful of genes loosely associated with PD. This approach creates a strong research bias toward rare single-gene mutations with relatively high mendelian inheritance (e.g. LRRK2, GBA), which are rare in global PD populations. Sporadic PD accounts for about 85% of cases globally, whereas the remaining 15% of cases are familial^20^. As a result, rare mendelian cases do not represent the sporadic PD cohorts enrolled in most clinical trials, creating a disconnect between pre-clinical and clinical research. Therefore, we combined the transcriptomes of sporadic genotypes (*n* = 10 individuals) and familial genotypes (*n* = 7 individuals; PARK2, LRK2-G2019S, SNCA-A53T). Despite the relatively large number of individuals analysed in our study compared to the current standard in the iPSC field (*n* total = 42), the cohort was still too small to gain substantial insights into differences between genetic and clinical groups of PD. Instead, we focused on identifying PD transcriptomic signatures that can be generalized to a broad PD population, including hereditary and sporadic cases. Significant variation between neuronal transcriptomics of genetic and sporadic cases and lack of clustering was also reported previously^39^. To the best of our knowledge^109^, this is the most extensive database exploring the transcriptomic profiles of PD using patient-derived reprogrammed neurons to date. As such, this study will serve as a solid starting point, which can be refined in the future with new data.

The neural stem cell reprogramming field has rapidly proliferated since the seminal discovery of iPSCs^110,111^. All agree that more rigorous technical harmonization between laboratories would increase reproducibility. However, researchers continue to improve the protocols for neural reprogramming, differentiation and maturation, and a gold-standard consensus has not been reached yet. For the moment, different methods have advantages and limitations, which may be more or less suited for specific laboratories or projects^112^. Therefore, in this study, we compared and combined the transcriptomics signature from PD patients using the two major neural reprogramming strategies from fibroblasts: iPSC reprogramming and direct conversion. Midbrain and dopaminergic neuronal profiles were enriched in all the datasets included in our study. Accordingly, the transcriptomes were remarkably similar to adult brain biopsies for the midbrain substantia nigra and striatum. In our integrative transcriptome analysis, we prioritized genes expressed in adult human substantia nigra to further increase clinical relevance and reduce cellular reprogramming biases.

Despite the significant progress in human neuronal reprogramming technologies, it is evident that human brain tissue in vitro cannot fully recapitulate the complexity of the adult brain in vivo. As such, we cannot exclude the possibility that the transcriptomic signatures reported in this study are missing clinically relevant features. However, bio-engineered human neuronal models address major limitations of 1) postmortem brain transcriptomic studies, which essentially highlight differences between “healthy” diseased brains and those damaged by decades of PD pathology, and 2) animal models, which have different genetics than humans. Instead, bio-engineered neuronal tissues represent a unique experimental opportunity to reveal the molecular dysregulations that precede neurodegeneration in humans. The PD transcriptomic signature of midbrain cells of this cohort was significantly associated with the human phenotype ontology relevant to Parkinson’s. Our analysis highlighted known and new genetic signatures that may guide or reinforce future therapeutic pipelines. In particular, we identified six major pathways dysregulated in PD patients across our entire cohort: neurotransmission, energy and metabolism, neuromorphogenesis, intracellular trafficking, glia inflammation and immunity, and cellular and oxidative stress responses. These cellular functions are highly interdependent, and we do not know which occurs upstream of the others and should therefore be targeted therapeutically as a priority to slow PD pathology. However, this study highlights candidate therapeutics that can target each of these pathways. Future studies will be required to assess their pharmacological potential to rescue multiple transcriptomic pathways dysregulated in PD neurons.

## Methods

### Ethics statement

All human cell lines and experimental protocols in the present study were used with approval and in accordance with policies of the following ethics committees: Women’s and Children’s Health Network Human Research Ethics Committee, South Australia, Australia [HREC/17/WCHN/70]; Salk Institute Institutional Review Board, La Jolla, USA. All subjects gave written informed consent to the derivation of iPSC and/or iN lines from skin biopsies, mutation screening and use of tissue for research. H9 (WA09) human embryonic stem cells were obtained from WiCell (Agreement No. 20-W0500).

### Human pluripotent stem cell culture and midbrain neuronal differentiation

Primary fibroblast cultures were derived from participants’ dermal skin punch biopsies and reprogrammed either by retroviral delivery or non-integrating (Sendai virus or episomal vectors) methods in different laboratories. Subject details and source information are provided in Supplementary Table 1.

The differential analysis in this paper was performed on six independent datasets from human midbrain neuronal cultures generated either through iPSC reprogramming (sc-iPS-PatchSeq, sc-iPS-10XSeq, bulk-iPS-Dopa, bulk-iPS-Mixed) or direct fibroblast conversion (bulk-iN-Mixed, bulk-iN-Dopa) as follows:

#### Midbrain cultures for sc-iPS-PatchSeq dataset

Human induced pluripotent stem cells and WA09 (H9) embryonic stem cells (WiCell, Wisconsin, U.S.A.) were maintained in mTeSR™1 medium (STEMCELL Technologies #85850) on cell culture ware coated with human Embryonic Stem Cell (hESC)-qualified Matrigel (Corning #354277) as per manufacturer’s instructions. Differentiation of pluripotent cells into neural progenitor cells (NPCs) was performed using an embryoid body (EB)-based protocol as described previously^113,114^. NPCs were expanded for three to five passages (split ratio of 1:2–1:3 per passage) on Matrigel-coated six-well plates and cryobanked in vapour nitrogen. For experiments, cells were thawed and expanded for at least one extra passage in neural progenitor medium (NPM). NPM was composed of DMEM/F12 + GlutaMax™ basal medium (Thermo Fisher Scientific #10565018) supplemented with 1× N2 (Thermo Fisher Scientific #17502048), 1× B27 (Thermo Fisher Scientific #17504044), FGF-8b (100 ng/mL, PeproTech #100-25), Sonic Hedgehog (200 ng/mL, R&D Systems #1314SH) and laminin (1 μg/mL, Thermo Fisher Scientific #23017015). For neuronal maturation, NPCs were dissociated and re-plated (density of 1.5 × 10^5^ cells/cm^2^) onto glass coverslips (Fisher Scientific #12-545-80) coated with 10 μg/mL poly-L-ornithine (Sigma-Aldrich #P3655) and 5 μg/mL laminin (Thermo Fisher Scientific #23017015) in 24- or 48-well plates. Twenty-four hours later, the cells were switched gradually (half medium change) to neuronal maturation medium (NMM): BrainPhys™ basal medium^114^ supplemented with 1× N2 (Thermo Fisher Scientific #17502-048), 1× B27 (Thermo Fisher Scientific #17504-044), BDNF (20 ng/mL, Thermo Fisher Scientific #450-02), GDNF (20 ng/mL, PeproTech #450-10), dibutyryl cyclic AMP (1 mM, Sigma-Aldrich #D0627), ascorbic acid (200 nM, Sigma-Aldrich #A0278), and laminin (1 μg/mL, Thermo Fisher Scientific #23017015). Half of the neuronal medium was gently replaced three times a week. Plates were kept in a humidified incubator at 37 °C with 5% CO_2_ and 21% O_2_. The osmolarity (~300–305 mOsmol/L) and pH (~7.3–7.4) of the medium were maintained constant over time.

#### Midbrain cultures for sc-iPS-10XSeq dataset^38^

Details regarding the dopaminergic neuronal differentiation of wild-type (WT) and isogenic *SNCA-A53T* iPSCs are available in the original publication^38^.

#### Midbrain cultures for bulk-iPS-Dopa dataset

The neuronal cultures for the bulk-iPSC-Dopa dataset were generated following a published protocol^115^ . Briefly, small molecules/factors (SB431542, LDN193-189, purmorphamine, FGF-8, SAG, CHIR99021, BDNF, GDNF, TGFb, ascorbic acid, cAMP) were used to differentiate iPSCs into midbrain DA neurons. These cells were not sorted but instead simply peeled off using a three to five minutes Accutase treatment at room temperature.

#### Neuronal cultures for bulk-iPS-Mixed dataset^31^

Details regarding iPSC maintenance and midbrain dopaminergic neuron differentiation are available in the original publication^31^. Briefly, iPSCs were maintained in E8 medium on Matrigel and passaged every five days (split ratio of 1:6–1:12 per passage) using Versene. For neuronal differentiation, iPSCs were grown to ~80% confluency, singularized using Accutase (Millipore/Sigma #SCR005) for 5 min at 37 °C, and plated as a fully confluent monolayer (density of 2 × 10^5^ cells/cm^2^) onto Matrigel-coated 6-well plates in E8 medium containing Y-27632 (5 μM, StemGent). Twenty-four hours after plating, the medium was changed to Stage 1 medium (50% DMEM/F12 and 50% Neurobasal supplemented with 1× N2, 1× B27-vitamin A, LDN-193189 [LDN] and SB431542 [SB]) for 3 days, then to Stage 2 medium (50% DMEM/F12 and 50% Neurobasal supplemented with 1× N2, 1× B27-vitamin A, LDN, SB, purmorphamine [PMN], CHIR99021 [CHIR], SHH and FGF8) for 4 days, then to Stage 3 medium (50% DMEM/F12 and 50% Neurobasal supplemented with 1× N2, 1× B27-vitamin A, LDN, CHIR and all-*trans* retinoic acid [ATRA]) for 4 days, and finally to Stage 4 medium (50% DMEM/F12 and 50% Neurobasal supplemented with 1× N2, 1× B27-vitamin A, BDNF, GDNF, dbCAMP, L-ascorbic acid, γ-secretase inhibitor (DAPT), CHIR and TGF-β3) for 3 days. Stage 1, Stage 2, Stage 3 and Stage 4 media changes were performed daily. On day 15, cultures were dissociated to single cells using Accutase (20 min at 37 °C), re-suspended in maturation medium (50% DMEM/F12 and 50% Neurobasal supplemented with 1× N2, 1× B27-vitamin A, BDNF, GDNF, dbCAMP, L-ascorbic acid, DAPT and TGF-β3) plus Y-27632 (5 μM), and re-plated onto Matrigel-coated culture ware (density of 2 × 10^5^ cells per cm^2^) or coverslips (density of 2 × 10^5^ cells per 50-μl drop). Cells were allowed to attach for 45 min at 37 °C, and maturation medium was then added to a final volume of 3 or 1.5 mL/well for, respectively, six-well plates and 24-well plates with coverslips. The medium was changed 48 hours after seeding and gently replaced every three days until day 30.

#### Neuronal cultures for bulk-iN-Mixed dataset^39^

Primary human dermal fibroblasts were directly reprogrammed into induced neurons (iNs) as previously described^39,116^. Briefly, cells were cultured in Dulbecco’s Modified Eagle’s Medium (DMEM) supplemented with 15% tetracycline-free fetal bovine serum and 0.1% NEAA (Thermo Fisher Scientific), transduced with lentiviral particles for EtO (^117,118^) and XTP-Ngn2:2 A:Ascl1 (N2A), and expanded in the presence of G418 (200 µg/ml; Life Technologies) and puromycin (1 µg/ml; Sigma-Aldrich). For iN conversion, fibroblasts were pooled into high densities and the medium was changed to neuron conversion (NC) medium after 24 hours, for three weeks. NC medium was composed of DMEM:F12/Neurobasal (1:1) supplemented with 1× N2 and 1× B27 (Thermo Fisher Scientific), doxycycline (2 μg/ml; Sigma-Aldrich), laminin 1 μg/ml; Thermo Fisher Scientific), dibutyryl cyclic AMP (500 μg/ml; Sigma-Aldrich), human recombinant Noggin (150 ng/ml; PeproTech), LDN-193189 (0.5 μM; Cayman Chemicals), A83-1 (0.5 μM; Stemgent), CHIR99021 (3 μM; LC Laboratories), forskolin (5 μM; LC Laboratories), and SB-431542 (10 μM; Cayman Chemicals). The medium was changed every third day. For further maturation, iNs were switched to DMEM:F12/Neurobasal-based neural maturation medium (NMM) containing N2, B27, GDNF and BDNF (both 20 ng/ml; R&D Systems), dibutyryl cyclic AMP (500 μg/ml), doxycycline (2 μg/ml), and laminin (1 μg/ml). For maturation on astrocyes, iNs were dislodged during week 4 using TrypLE, replated on a feeder layer of mouse astrocytes, and cultured in NMM containing 1% knockout serum replacement (KOSR) (Thermo Fisher Scientific).

Successfully induced neurons were purified from non-converted fibroblasts by fluorescence-activated cell sorting (FACS) of cells with high expression the polysialylated form of Neuronal Cell Adhesion Molecule (PSA-NCAM). In particular, after three weeks of iN conversion, cells were detached using TrypLE and stained for PSA-NCAM (1:100, Milteny) for 45 min at 4 °C in sorting buffer (PBS containing 1% KOSR, 250 mM myo-inositol and 5 μg/ml polyvinyl alcohol [PVA]). Cells were washed, stained with Alexa-647-conjugated anti-mouse IgM secondary Ab for 30 min at 4°, resuspended in sorting buffer supplemented with EDTA and DNase, and filtered through a 40-μm cell strainer. Alexa-647-positive cells were sorted directly into TRIzol LS Reagent, and RNA was isolated and digested using TURBO DNase (Thermo Fisher Scientific) following manufacturer’s instructions.

#### Neuronal cultures for bulk-iN-Dopa dataset

Method for iN-Dopa conversion is based on the same as iN-mixed protocol, adding an extra lentiviral vector XTP-Lmx1a to the transcriptional factor cocktail and purmorphomine (2 μM; Adooq Bioscience), SAG(0.25 μM; Adooq Bioscience), and FGF-8 (100 ng/mL, Peprotech) in the conversion medium. Neurons were harvested at week 4 for RNA extraction.

### Sample preparation for RNA-seq

Three sample preparation methods (bulk RNAseq, single-cell RNAseq and Patch-seq) were used as follows:

#### RNAseq sample preparation for sc-iPS-PatchSeq dataset

Individual coverslips containing neurons were transferred into a heated (25 °C) recording chamber and continuously perfused (1 ml•min^−1^) with either BrainPhys™ basal medium^114^ or artificial cerebrospinal fluid (ACSF) bubbled with a mixture of CO_2_ (5%) and O_2_ (95%). ACSF composition was adjusted to match the inorganic salt concentration and osmolarity of BrainPhys™ basal and contained: 121 mM NaCl, 4.2 mM KCl, 1.1 mM CaCl_2_, 1 mM MgSO_4_ (or 0.4 mM MgSO_4_ and 0.3 mM MgCl), 29 mM NaHCO_3_, 0.45 mM NaH_2_PO_4_-H_2_O, 0.5 mM Na_2_HPO_4_ and 20 mM D-glucose (all chemicals from Sigma-Aldrich). Whole patch-clamp electrophysiological recordings were performed on single neurons infected with and expressing Synapsin:GFP lentiviral vector according to the protocol detailed below under “Patch-clamp recordings”. Single neurons were collected after electrophysiological recording as previously described^40,41^. Briefly, negative pressure (−0.15 PSI) was applied to the patch pipette to establish a strong seal between the patch electrode and the recorded neuron following patch-clamp recording. The neuron, including its processes (axon and dendrites), was then slowly withdrawn from the rest of the culture and transferred (in a volume of ~2 μl of internal patch solution) into 8.0 μl of sample lysis buffer (SMARTer™ Ultra Low Input RNA kit, Clontech #634828) by breaking the tip of the electrode along the inside wall of the PCR tube. Successful cell collection was always confirmed by DIC optics, and cDNA was synthesized from poly(A) + RNA with minimal delay after sample collection following manufacturer’s instructions (Clontech #634828). cDNA samples were assessed for quality (2100 Bioanalyzer, Agilent) and quantity (Qubit fluorometer, Thermo Fisher Scientific), and construction of mRNA-seq libraries was performed with 0.25 ng of input cDNA using Nextera XT DNA sample prep reagents (Illumina #FC-131-1096). Library fragments were size-selected, purified, quantified and examined for the correct size (2200 TapeStation High Sensitivity D1K ScreenTape Assay, Agilent #5067-5363), and equimolar amounts of uniquely barcoded libraries were multiplexed and sequenced on a HiSeq 2000 or 2500 platform (Illumina) using 100-bp paired-end sequencing. Only functionally mature neurons were included in the analysis (AP types 4 and 5 based on^40^).

#### RNAseq sample preparation for sc-iPS-10XSeq dataset^38^

Details regarding the neuronal sample preparation for single-cell transcriptomics with the 10x Genomics Chromium™ system are available in the original publication^38^. Briefly, after three weeks of differentiation into dopaminergic neurons, cells were dissociated using Accutase (Thermo Fisher Scientific), counted, and assessed for viability. Cell suspensions (8000 viable single cells) were loaded onto a 10x Chromium instrument (10x Genomics) according to the manufacturer’s protocol.

#### RNAseq sample preparation for bulk-iPS-Mixed dataset^31^

mRNA was isolated in triplicate for each differentiated cell line and prepared for sequencing as per previously described methods in the original publication^31,119^. RNA samples were assessed for quality (2100 Bioanalyzer, Agilent) and quantity (Qubit fluorometer, Thermo Fisher Scientific), and up to 1 μg of total RNA per sample was processed for cDNA synthesis, amplification and library construction using the TruSeq Stranded mRNA library preparation kit (Illumina). Constructed libraries were purified using Agencourt AMPure XP beads (Beckman Coulter #A63881), quantified for yield (Qubit fluorometer), and quality-assessed by fragment analysis (2100 Bioanalyzer). Sample libraries were then multiplexed and sequenced on a NextSeq 500 sequencer (Illumina) using 75-bp single-end SBS chemistry.

#### RNAseq sample preparation for bulk-iPS-Dopa, bulk-iN-Mixed and bulk-iN-Dopa datasets

mRNA-seq libraries were constructed using the TruSeq Stranded mRNA Sample Prep kit following the manufacturer’s protocol (Illumina) and sequenced on a HiSeq 2500 platform (Illumina) using 50-bp single-end SBS chemistry.

### Bioinformatics RNA-seq data processing, expression quantification, and quality control

All transcriptomics data was processed from raw FASTQ files. *Bulk RNA-seq data.* FASTQ files from all bulk RNA-seq datasets were read-trimmed using TrimGalore (v0.6.6) on default settings. Trimmed, high-quality reads were pseudo-aligned, and transcript-level abundances were quantified using Salmon^120^ (v1.3.0) with a decoy-aware transcriptome index for the *Homo sapiens* GRCh38 genome built from human GENCODE v35 annotation. Read quality, trimming performance and alignment statistics were assessed for each sample using FastQC (v0.11.9) and MultiQC (v1.9)^121^. The average number of mapped reads across all bulk RNA-seq samples was 28.3 million (minimum 14.6 million), with 78 of 80 samples having >75% mapped reads (average 85%, minimum 57%). Quality control metrics for each sample are detailed in Supplementary Table 3. *Single-cell 10x Chromium RNA-seq data.* Transcript quantification from sample-demultiplexed 10x Chromium FASTQ files (dataset sc-iPS-10XSeq^38^) was performed with Salmon’s single-cell processing module Alevin^122^ using the same GRCh38 (GENCODE v35)-based transcriptome index as used for the bulk RNA-seq datasets. 60% of all reads were properly mapped in the two 10x samples processed. The gene-by-cell matrix of counts was imported into R (v4.0.2) using tximport^123^ (v1.16.1) for further filtering with Seurat^124,125^ (v4.0.0). For downstream analysis, we conservatively only retained higher-quality cells with an overall mapping rate of at least 50% of reads^43^. For each of these cells, we quantified the number of total genes and housekeeping genes^42^ detected (i.e., genes with at least one read count). We then excluded all cells with fewer than 1,000 detected genes or with less than 66% housekeeping genes expressed (based on Tirosh et al.’s curated list of 98 HK genes^42^). To exclude potential cell doublets or multiplets from downstream analysis, we additionally removed cells with an exceptionally high gene or UMI content (i.e., more than 8000 genes or 37,500 unique molecular identifiers detected). This left us with a total of 5,315 QC-passed cells for further analysis. *Single-cell PatchSeq data.* For the PatchSeq dataset (dataset sc-iPS-PatchSeq), FASTQ reads were adapter and quality trimmed with Trimmomatic^126^ (v0.39) using a custom list of adapter/primer sequences and the following parameters: *ILLUMINACLIP*:path/to/adapters.fa:2:30:10 *LEADING*:3 *TRAILING*:3 *SLIDINGWINDOW*:4:15 *MINLEN*:36. Transcript quantification was carried out using Salmon as performed for bulk RNA-seq data, but with the invocation of the *--gcBias* argument to correct for fragment-level GC biases in the paired-end input data, and using a decoy-aware index that included sequences for added RNA spike-ins (ERCCs and ArrayControl spikes) and used fluorescent reporters in addition to the human GRCh38 (GENCODE v35) reference. Cells (*n* = 6) with a read alignment rate below 50% were considered poor quality and removed from further analysis. Transcript-level counts from Salmon were summarized to the gene level using tximport^123^ (v1.16.1) with *countsFromAbundance* set to “lengthScaledTPM”, and imported into with Seurat^124,125^ (v4.0.0) for calculation of per-cell QC metrics and cell filtering. Consistent with the QC performed on the 10x Chromium data, cells with fewer than 1000 or more than 8000 total genes detected, or with fewer than 65 detected housekeeping genes, were removed. Across all remaining cells (*n* = 44), the average number of mapped reads was 6.4 million, corresponding to an alignment rate of 77%.

### GTEx RNA-seq data processing

Genotype-Tissue Expression (GTEx)^127,128^ v8 transcriptome data (dbGaP Accession phs000424.v8.p2, accessed December 2020) and sample annotation information for 55 adult tissue types were retrieved from the GTEx portal ((http://www.gtexportal.org/). The expression data (read counts and transcripts per million [TPM] values) were read into R using the read.gct function from the CePa^129^ (Centrality-Based Pathway Enrichment) package (v0.7.0). Transcriptome samples were filtered to only include those with an RNA integrity (RIN) score of 7.5 or higher (SMRIN column of annotation file) and that have been identified by the GTEX Consortium to be best suited for RNA-seq analysis (SMAFRZE column of annotation file). We subsetted the data for substantia nigra (*n* = 19) and striatum (caudate, *n* = 126; putamen, *n* = 70) tissue samples for clustering with data from reprogrammed neurons (Fig. 2A, B, analysis details provided below).

### Clustering analysis

Principal components analysis (PCA) and hierarchical clustering of reprogrammed neuron and GTEx (post-mortem adult substantia nigra and striatum tissue) samples passing quality control was performed using R (v4.0.2) and RStudio (v1.3.1073) software based on normalized expression values. *PCA* (Fig. 2A): For proper comparison with bulk RNA-seq data, we generated artificial bulk samples for the two single-cell RNA-seq datasets by summing the read counts across all cells sequenced from each subject. PCA analysis of reprogrammed neuron (*n* = 80 bulk and 6 artificial bulk) and GTEx substantia nigra (*n* = 19 bulk) samples was performed on variance-stabilized transformed counts using the plotPCA function in DESeq2^130^ (v.1.28.1) using all genes expressed at a minimum of 10 counts across all samples. *Hierarchical clustering* (Fig. 2B): Salmon TPM (Transcripts Per Million) values for all samples were imported into R using tximport^123^ (v1.16.1). For the heatmap clustering of datasets based on an Euclidean distance metric, TPM values were averaged across all cells or samples in each respective dataset, log-transformed and Z-scaled, and computed distances were clustered by average-linkage hierarchical clustering. Only genes expressed at greater than or equal to 1 TPM across the total number of samples were used to compute Euclidean distances. Heatmap visualization was performed using the pheatmap^131^ package (v1.0.12) in R.

### Differential gene expression analysis

Differential gene expression analyses were performed on Salmon-generated gene counts and TPM values using slightly different methods to identify genes dysregulated in each dataset of PD versus healthy control reprogrammed neurons. Single-cell RNA-seq datasets were tested for differentially expressed genes using both (i) Seurat^124,125^ (v4.0.0)’s standard Wilcoxon rank sum test applied to count data, and a (ii) Wilcoxon rank sum test applied to raw TPM data within R (wilcox.test function). For bulk RNA-seq datasets, differential expression analysis on raw counts was performed using the DESeq2^130^’s (v1.28.1) Wald test, and raw TPM data were analysed by Wilcoxon rank sum test as performed for single-cell RNA-seq data. Non-expressed genes had their *p* values set to 1. The raw *p* values generated by the two DE analysis tests were combined into a single combined *p-*value by logit method (*logitp* function from the *metaseqR* package, v1.28.0). Log2-fold changes of mean gene expression were calculated for each of the two DE analysis tests separately and then averaged to obtain a single-fold change measure for PD versus control reprogrammed neurons.

### Calculation of per-dataset gene dysregulation scores (*D*_*dataset*_)

For all genes expressed (≥1 TPM in at least 10% of cells or samples) in each dataset *d,* the combined gene fold changes and combined *p-values* were each mapped to a continuous 0.01-1 scale using desirability functions provided by the *desiR* package^48^. Specifically, the gene with the lowest (most significant) *p* value received maximum desirability of 1, whereas genes with values >0.05 all received low desirability of 0.01. Genes with *p-*values in between these cut-offs received intermediate value desirability values. Desirability values for fold changes were computed in a similar way. We considered genes with a large fold change (>2) maximally desirable (value of 1), and genes with a small fold change (<1.25) minimally desirable (value of 0.01); intermediate desirability values were assigned to genes with a fold change in between these cut-offs. Additionally, we calculated a midbrain expression desirability score for each gene, where genes with transcript detection in the adult midbrain above cut-off (NX ≥ 1; as per The Human Brain/Protein Atlas^132^) were considered maximally desirable (score of 1), and genes not detected in midbrain received a low score of 0.25. A value of 0.01 instead of zero was chosen as the minimum desirability score for each dataset’s gene fold change and p-value as we did not want to exclude any genes, only make them least important.

For each gene in each dataset, the desirability scores computed for *p*-value, fold change and midbrain expression were subsequently combined into a dysregulation score (*D*_*dataset*_*)*, using a weighted geometric mean with weights of 1, 0.5 and 0.01, respectively. The midbrain expression desirability score was integrated at a low weight of 1/100th that of the *p* value desirability score and 1/50^th^ that of the fold change score to ensure the *D*_*dataset*_ scores were predominantly driven by the differential testing results, with midbrain gene expression level only serving as a soft adjustment variable to the final ranking of the genes. Genes not expressed in the dataset were given a minimal *D*_*dataset*_ *score* of 0.01. All source data are in Supplementary Data 1.

### Calculation of multiple-dataset gene dysregulation scores (*D*_*overall*_*)*

To identify a common signature of genes perturbed in PD reprogrammed neurons across multiple datasets, we combined the individual dataset dysregulation scores into a higher-level overall dysregulation score (*D*_*overall*_). Score *D*_*overall*_ is calculated by geometric averaging of all individual (*n* = 6) dataset dysregulation scores (at equal weights) with a factor that adjusts for concordance of directionality of fold change values between datasets (at one-tenth of the total weight); i.e., *D*_*overall*_ score is proportionally reduced with the degree of discordance in fold change directionality between the datasets in which the gene is expressed. Refer to Supplementary Data 1 for source data.

### Functional enrichment analysis

Functional enrichment analysis of the top 200 genes with the highest *D*_*overall*_ score was performed using ToppFun (https://toppgene.cchmc.org/, database March 2021) to test for overrepresentation in Gene Ontology (GO) biological process (Fig. 3B) and cellular component (Fig. 3C) terms, as well as gene-chemical interactions curated by the Comparative Toxicogenomics Database^67^ (CTD; Fig. 3D). Benjamini-Hochberg method was used to adjust *P*-values for multiple testing, with enriched terms being considered significant for adjusted *P*-values < 0.05. Detailed enrichment results are provided as Supplementary Table 4.

### Gene Set Enrichment Analysis

Gene Set Enrichment Analysis (GSEA)^133^ was performed to explore whether the multiple dataset dysregulated genes showed a significant correlation with genes involved in Parkinsonism, dementia and schizophrenia (Fig. 3E), PD symptoms (Fig. 3F) and abnormal central nervous system electrophysiology (Fig. 5A). Input data were (1) our list of genes (*n* = 24,693 unique expressed in ≥1 dataset) rank-ordered by decreasing D_overall_ score, and (2) one of several phenotype gene sets retrieved from the Human Phenotype Ontology (HPO) database^131^: “Parkinsonism”/HP:0001300 (*n* = 74 genes), “Dementia”/HP:0000726 (*n* = 132 genes), “Schizophrenia”/HP:0100753 (*n* = 51 genes); “Akinesia”/HP:0002304 (*n* = 19 genes), “Bradykinesia”/HP:0002067 (*n* = 68 genes), “Dyskinesia”/HP:0100660 (*n* = 164 genes), “Postural instability”/HP:0002172 (*n* = 42 genes), “Rigidity”/HP:0002063 (*n* = 131 genes), “Resting tremor”/HP:0002322 (*n* = 28 genes); “Abnormality of CNS electrophysiology”/HP:0030178 (*n* = 358 genes). The D_overall_-ranked gene list was analyzed for overrepresentation of each HPO gene set at the top using GSEAPreranked (v4.1.0) with default parameters (enrichment statistic: weighted; min size: 15; max size: 500; normalization mode: meandiv; the number of gene-set permutations: 1000). All p-values were FWER-corrected to exclude any possibility of false-positive enrichment. As an additional validation step, gene labels were manually shuffled at random to destroy the original gene label–*D*_*overall*_ score relationship, and the resulting randomized gene list was tested for enrichment for the same gene sets (Supplementary Fig. 2). Detailed enrichment results are provided in Supplementary Table 4.

### Pathway enrichment and network analysis

Pathway enrichment analysis was performed (Fig. 4) to explore whether the genes commonly dysregulated between datasets showed significant correlation with genes annotated to known biological pathways^83^. We rank-ordered the entire list of genes (*n* = 24,693 unique genes expressed in ≥1 dataset) by decreasing *D*_*overall*_ score and statistically tested for overrepresentation of a broad collection of pathway gene sets at the top of the list using Gene-Set Enrichment Analysis (GSEA;^83,133^). The collection of 18,684 pathway gene sets was downloaded as a single GMT file from download.baderlab.org/EM_Genesets (Human_GOBP_AllPathways_no_GO_iea_February_05_2021_symbol.gmt) and filtered for gene set size (min = 15 and max = 250 features after restricting to dataset). The remaining 5,654 pathways were analyzed for statistical overrepresentation using GSEA’s “pre-ranked” tool with default parameters (enrichment statistic: weighted; normalization mode: meandiv; 1000 gene-set permutations). The Enrichment Score (ES) calculated for each pathway reflects the degree of overrepresentation of pathway genes at the top of the ranked list. Pathway genes were randomly permuted 1,000 times, and the significance of the actual ES versus the 1,000 times permuted ES evaluated to calculate significance (*p*-value). Pathway ES scores were normalized relative to pathway size to obtain a normalized enrichment score (NES) that was used to rank the biological pathways by degree of enrichment in the list of dysregulated genes. Enrichment results were interpreted and statistically significant pathways (FDR-q < 0.01) visualized as a network representation using Cytoscape v3.8.0 with the EnrichmentMap v3.3.1 and AutoAnnotate v1.3.3 plug-ins as per a previously described protocol^83^ (also available at https://cytoscape.org/ cytoscape-tutorials/protocols/enrichment-pipeline) using Jaccard Overlap combined coefficient >0.375 with combined constant = 0.5. As an additional validation step to confirm the specificity of the identified biological pathways, gene labels were manually shuffled at random to destroy the original gene label–*D*_*overall*_ score relationship. The resulting randomized gene list was tested for enrichment for the same collection of pathway gene sets. Random permutation of gene labels resulted in no significant enrichment of any pathway gene set (FDR-q < 0.01). Detailed enrichment results of this analysis are provided in Supplementary Table 8.

### Drug prediction based on dysregulated transcriptomic pathways

DrugBank’s database of *n* = 5,724 drug-target gene associations^90^ was obtained as a single GMT file from download.baderlab.org/EM_Genesets (Human_DrugBank_all_symbol.gmt). To test for overrepresentation of DrugBank therapeutic candidates amongst the rank-ordered list of PD-dysregulated genes, a GSEA was performed using GSEAPreranked (v4.1.0) with default parameters (enrichment statistic: weighted; min size: 15; max size: 500; normalization mode: meandiv; the number of gene-set permutations: 1000). We downloaded McFarthing et al.’s^2^ comprehensive list of 145 registered and active clinical trial therapies for PD (the year 2020) and performed a systematic literature search to link each therapy’s mechanism(s) of action to the major theme(s) of transcriptomic dysregulation. Clinical trial therapies were categorized into disease-modifying and symptomatic therapies similar to McFarthing et al.’s classification^2^ and grouped by clinical trial phase. Trials were filtered for redundancy, keeping only the trial in the most advanced trial phase.

### Patch-clamp recordings

A total of >169 mature (Type 5; see definition below) neurons were patched and analyzed (Fig. 5). For whole-cell patch clamp recordings, individual coverslips were transferred into a heated recording chamber and continuously perfused (1 ml•min-1) with either artificial cerebrospinal fluid (ACSF) or BrainPhys™ basal media^114,134^ bubbled with a mixture of CO_2_ (5%) and O_2_ (95%), and maintained at 25 °C. For targeted whole-cell recordings, we used a 40x water-immersion objective, differential interference contrast filters (all Olympus), an infrared digital camera (Rolera XR – Qimaging), a digidata 1440 A/Multiclamp 700B and Clampex 10.3 (Molecular Devices). Patch electrodes were filled with internal solutions containing 130 mM K-gluconate, 6 mM KCl, 4 mM NaCl, 10 mM Na-HEPES, 0.2 mM K-EGTA; 0.3 mM GTP, 2 mM Mg-ATP, 0.2 mM cAMP, 10 mM D-glucose, 0.15% biocytin and 0.06% rhodamine. The pH and osmolarity of the internal solution were close to physiological conditions (pH 7.3, 290–300 mOsmol). Data were all corrected for liquid junction potentials (10 mV). Electrode capacitances were compensated on-line in cell-attached mode (~7 pF). Recordings were low-pass filtered at 2 kHz, digitized, and sampled at intervals of 50 ms (20 kHz). To control the quality and the stability of the recordings throughout the experiments, access resistance, capacitance and membrane resistance were continuously monitored on-line and recorded. The resistance of the patch pipettes was between 3 and 5 MOhm. The access resistance of the cells in our sample was ~40 MOhm on average. Synaptic antagonists were only used on a subset of neurons to confirm the nature of the spontaneous synaptic events detected. All events showing typical synaptic AMPA receptor-mediated kinetics were blocked by NBQX (10 µM; Sigma-Aldrich Cat. No. N183) and were observed exclusively in voltage clamp at −70 mV (close to Cl^-^ reversal potential). All events showing typical synaptic GABA_A_ receptor-mediated kinetics were blocked by gabazine (SR95531, 10 µM; Sigma-Aldrich Cat. No. S106) and were observed exclusively in voltage clamp at 0 mV (close to Na^+^ reversal potential).

### Action potential (AP) type classification

Action potential type classification was based on a previously published definition^40^ as follows: “Type 0 cells” are most likely non-neuronal cells and do not express voltage-dependent sodium currents; “Type 1 neurons” express small Nav currents but are not able to fire action potentials (APs) above −10 mV; “Type 2 neurons” fire only one AP above −10 mV, which is typically followed by a plateau; “Type 3 neurons” also fire an AP above −10 mV and one or a few aborted spikes below −10 mV; “Type 4 neurons” fire more than one AP above −10 mV but at a frequency below 10 Hz; “Type 5 neurons” fire APs above −10 mV at 10 Hz or more. The threshold of −10 mV was chosen as it is close to the reversal potential of cations (0 mV), and a sign of healthy mature APs.

### Statistical analysis of electrophysiological data

Statistical analysis of electrophysiology data was assisted with Clampfit 10.3, MATLAB 2011b, Igor Pro v6, GraphPad Prism v8, MiniAnalyis and Microsoft Excel. Data are presented as mean ± SEM. Statistical significance was assessed with two-tailed non-parametric paired (Wilcoxon) or unpaired (Mann Whitney) tests. The criterion for significance was set as *P* < 0.05.

## Supplementary information

Supplementary Figures 1-2

Supplementary Table 1

Supplementary Table 2

Supplementary Table 3

Supplementary Table 4

Supplementary Table 5

Supplementary Table 6

Supplementary Table 7

Supplementary Table 8

Supplementary Data

## Data Availability

The following sequencing datasets available under NCBI GEO or ArrayExpression accession codes were used in this study: “ArrayExpress E-MTAB-9154” (10x Chromium single-cell RNA-seq data of iPSC-derived dopamine neurons; dataset sc-iPS-10XSeq^38^, “GEO GSE120746” (transcriptome data from midbrain dopaminergic mixed neural cultures; dataset bulk-iPS-Mixed^31^, and “ArrayExpress E-MTAB-3037” (transcriptome data from PSA-NCAM-positive iNs sorted from mixed cultures; dataset bulk-iN-Mixed^39^. Datasets sc-iPS-PatchSeq, bulk-iPS-Dopa and bulk-iN-Dopa are not publicly available due to specific consents of individuals who donated biological material to conduct this study but are available on reasonable request from the corresponding author to qualified researchers to the extent permitted by the Research Ethics Committee. All other relevant data supporting the key findings of this study are available within the article and its Supplementary Information files, or from the corresponding author upon request. Patient-related information not included in the manuscript may be subject to patient confidentiality.

## Code availability

The code for data cleaning and analysis is available at https://github.com/bardylab/RNAseq_iPSC_PD.git or from the corresponding author.
