# Supplementary material for: Druggable transcriptomic pathways revealed in Parkinson’s patient-derived midbrain neurons
Source: NPJ Parkinsons Dis. 2022 Oct 18;8:134. doi: 10.1038/s41531-022-00400-0 (PMC9579158; doi:10.1038/s41531-022-00400-0)
Supplement: Supplementary file 1 — Supplementary Figures 1-2 [file 41531_2022_400_MOESM1_ESM.pdf]

# *Supplementary Figures 1 - 2*

# Druggable transcriptomic pathways revealed in Parkinson's patient-derived midbrain neurons

Mark van den Hurk[1], Shong Lau[2], Maria C. Marchetto[2], Jerome Mertens[2,3], Shani Stern[2,4], Olga Corti[5], Alexis Brice[5], Beate Winner[6,7,8], Jürgen Winkler[6,7,8], Fred H. Gage[2] and Cedric Bardy[1,9,*]

[1]South Australian Health and Medical Research Institute (SAHMRI), Laboratory for Human Neurophysiology and Genetics, Adelaide, SA, Australia

[2]Laboratory of Genetics, Salk Institute for Biological Studies, La Jolla, CA, USA

[3]Neural Aging Laboratory, Institute of Molecular Biology, CMBI, Leopold-Franzens-University Innsbruck, Tyrol, Austria

[4]Sagol Department of Neurobiology, Faculty of Natural Sciences, University of Haifa, Haifa, Israel

[5]Sorbonne Université, Institut du Cerveau - Paris Brain Institute - ICM, Inserm, CNRS, AP-HP, Hôpital de la Pitié Salpêtrière, DMU BioGeM, Paris, France

[6]Department of Stem Cell Biology, University Hospital Erlangen, Friedrich-Alexander University Erlangen-Nürnberg (FAU), Erlangen, Germany

[7]Center of Rare Diseases Erlangen (ZSEER), University Hospital Erlangen, FAU Erlangen-Nürnberg, Erlangen, Germany

[8]Department of Molecular Neurology, University Hospital Erlangen, FAU Erlangen-Nürnberg, Erlangen, Germany

[9]Flinders Health and Medical Research Institute, Flinders University, Adelaide, SA, Australia

[*]**Correspondence:**

Associate Professor Cedric Bardy. SAHMRI, Adelaide SA 5000, Australia.

Email: cedric.bardy@sahmri.com

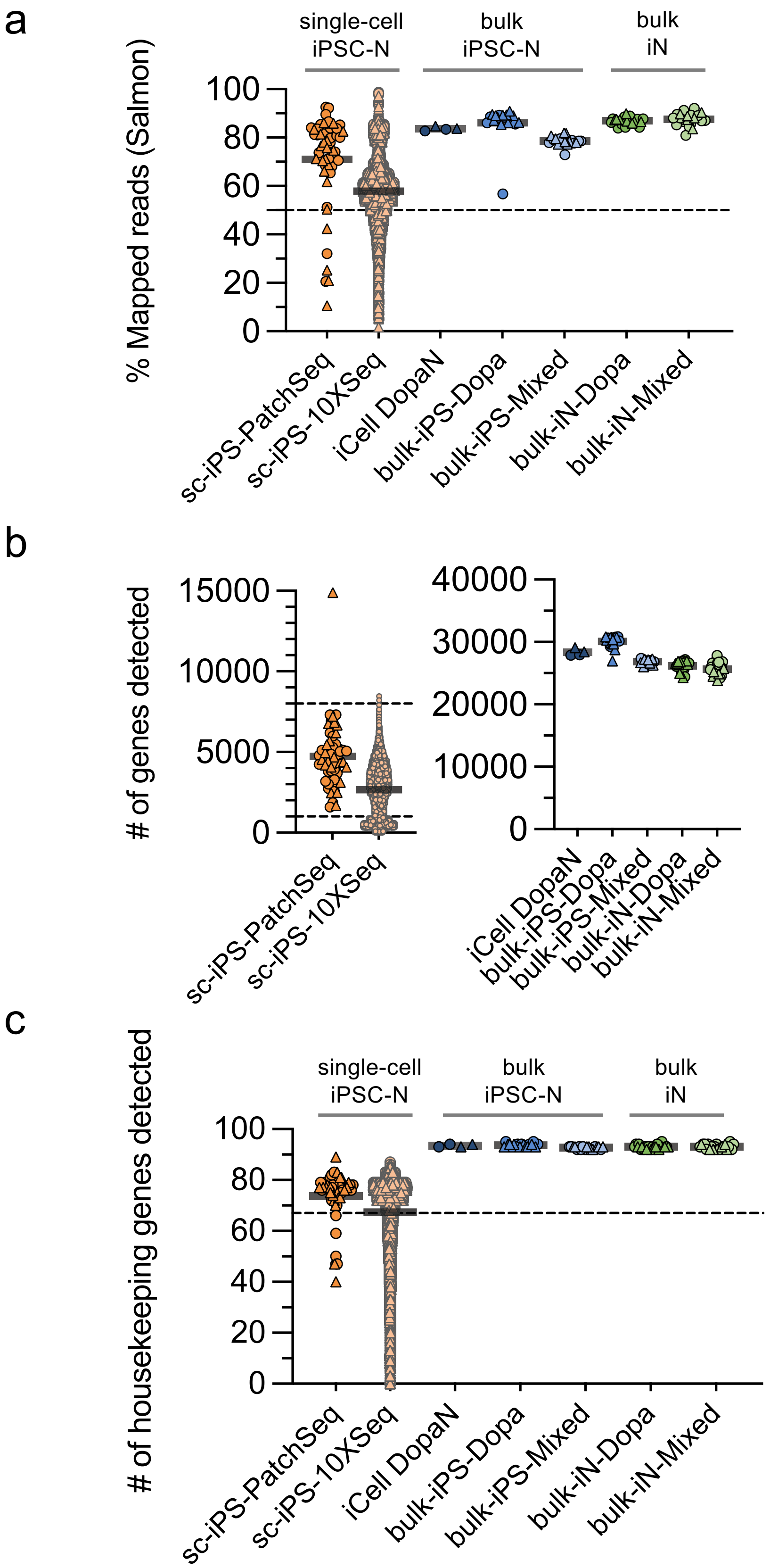


**Supplementary Figure 1.** Quality control metrics (percentage of mapped reads (**a**), number of genes detected (**b**) and number of housekeeping genes expressed (**c**)) for each single-cell or bulk RNA-seq library. Horizontal dotted lines indicate critical thresholds for sample inclusion/exclusion (refer to Methods for details).

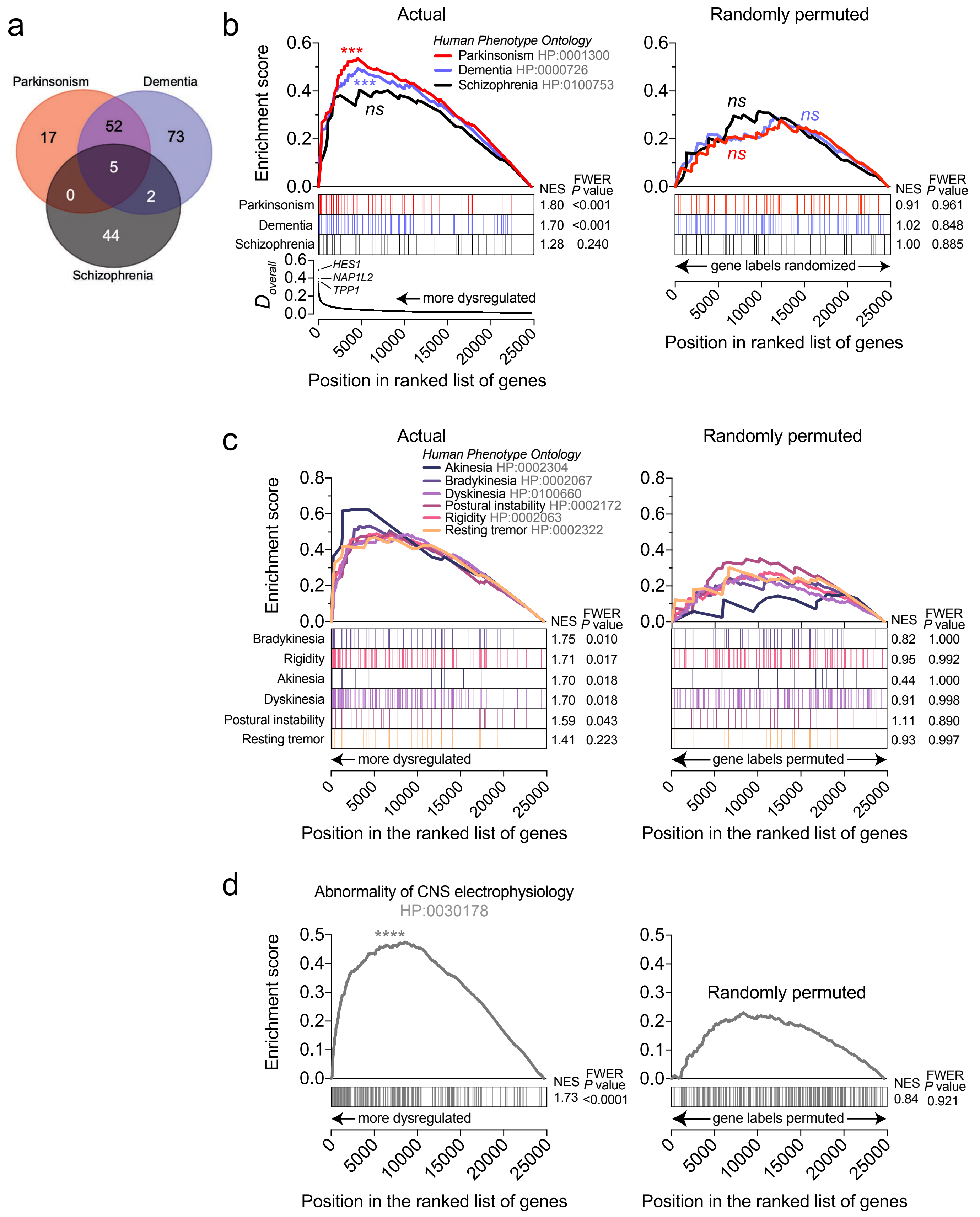


**Supplementary Figure 2. a** Venn analysis of genes annotated to the Human Phenotype Ontologies for Parkinsonism, Dementia and Schizophrenia reveals substantial overlap between parkinsonism and dementia genes. **b-d** Significant associations of the dysregulated PD transcriptome with Parkinsonism (**b**), PD clinical symptoms ontology (**c**) and abnormality of central nervous system electrophysiology (**d**) is completely abolished upon random permutation of gene labels, effectively destroying the gene-dysregulation score relationship.
