# Supplementary material for: Druggable transcriptomic pathways revealed in Parkinson’s patient-derived midbrain neurons
Source: NPJ Parkinsons Dis. 2022 Oct 18;8:134. doi: 10.1038/s41531-022-00400-0 (PMC9579158; doi:10.1038/s41531-022-00400-0)
Supplement: Supplementary file 9 — Supplementary Table 8 [file 41531_2022_400_MOESM9_ESM.pdf]

Supplementary Table 8. No significantly enriched pathways (FDR-q < 0.01) exist after destroying the gene-dysregulation score relationship by random permutation of gene labels.

name	ES	NES	gene set size	FDR-q	rank at max
NOTCH4 INTRACELLULAR DOMAIN REGULATES TRANSCRIPTION%REACTOME%R-HSA-9013695.1	0.6041971	1.7313178	19	0.85556304	1090
PID_FAS_PATHWAY%MSIGDB_C2%PID_FAS_PATHWAY	0.42682242	1.3035955	28	0.8572201	4268
HALLMARK_HEDGEHOG_SIGNALING%MSIGDB_C2%HALLMARK_HEDGEHOG_SIGNALING	0.6307089	1.9107088	26	0.85764205	3447
THYROTROPIN-RELEASING HORMONE RECEPTOR SIGNALING PATHWAY%PANTHER PATHWAY%P04394	0.3975021	1.3041861	56	0.8576839	10042
NEGATIVE REGULATION OF CATION TRANSMEMBRANE TRANSPORT%GOBP%GO:1904063	0.39285558	1.3036168	55	0.85834944	6440
PHAGOCYTOSIS, ENGULFMENT%GOBP%GO:0006911	0.4244446	1.30384	31	0.8584057	8987
SOMATIC DIVERSIFICATION OF IMMUNOGLOBULINS%GOBP%GO:0016445	0.44425026	1.3042376	22	0.8586879	7203
TRANSCRIPTIONAL REGULATION OF WHITE ADIPOCYTE DIFFERENTIATION%REACTOME%R-HSA-381340.2	0.3833245	1.3030955	80	0.8588177	4972
VESICLE BUDDING FROM MEMBRANE%GOBP%GO:0006900	0.3773757	1.3025392	99	0.85952765	9298
MEMBRANE PROTEIN PROTEOLYSIS%GOBP%GO:0033619	0.40811825	1.291898	38	0.85983014	9285
MATURATION OF SSU-RRNA FROM TRICISTRONIC RRNA TRANSCRIPT (SSU-RRNA, 5.8S RRNA, LSU-RRNA)%GOBP%GO:0000462	0.4243484	1.3042523	29	0.8598757	930
NEGATIVE REGULATION OF MYELOID LEUKOCYTE DIFFERENTIATION%GOBP%GO:0002762	0.4440322	1.30444	22	0.8600561	6752
BIOCARTA_TPO_PATHWAY%MSIGDB_C2%BIOCARTA_TPO_PATHWAY	0.4555831	1.3026547	18	0.8600881	4523
REGULATION OF CARDIAC MUSCLE CONTRACTION%GOBP%GO:0055117	0.3891159	1.3048176	58	0.8603709	6246
MYELOID CELL DEVELOPMENT%GOBP%GO:0061515	0.46763483	1.2926501	15	0.86037177	311
COPII-COATED VESICLE BUDDING%GOBP%GO:0090114	0.3856934	1.3019285	70	0.8604198	9298
RAC PROTEIN SIGNAL TRANSDUCTION%GOBP%GO:0016601	0.45438462	1.301705	19	0.86047965	7389
PROTEIN PEPTIDYL-PROLYL ISOMERIZATION%GOBP%GO:0000413	0.44554886	1.3050143	22	0.8604826	2273
REGULATION OF MITOCHONDRIAL MEMBRANE PERMEABILITY%GOBP%GO:0046902	0.39390767	1.305195	61	0.8606798	8236
NEUROTRANSMITTER UPTAKE%GOBP%GO:0001504	0.450246	1.2900784	19	0.86071455	592
LIPOSACCHARIDE METABOLIC PROCESS%GOBP%GO:1903509	0.3817657	1.290273	96	0.860762	5803
POSITIVE REGULATION OF POTASSIUM ION TRANSPORT%GOBP%GO:0043268	0.41037035	1.290906	33	0.860765	4165
REGULATION OF PEPTIDYL-THREONINE PHOSPHORYLATION%GOBP%GO:0010799	0.41375643	1.3045266	37	0.8607835	1365
PROTEASOME DEGRADATION%WIKIPATHWAYS_20210110%WP183%HOMO SAPIENS	0.40768307	1.292977	40	0.8608845	8274
BY HDAC CLASS III	0.43229035	1.3235731	25	0.86090267	2646
EMBRYONIC AXIS SPECIFICATION%GOBP%GO:0000578	0.4590691	1.2927597	17	0.8609352	7031
PHOSPHORYLATION OF THE APC C%REACTOME%R-HSA-176412.3	0.44141915	1.291898	20	0.8609984	9837
REGULATION OF NITRIC-OXIDE SYNTHASE ACTIVITY%GOBP%GO:0050999	0.42054486	1.3053555	31	0.8610595	9404
ACTIN CYTOSKELETON REORGANIZATION%GOBP%GO:0031532	0.38868102	1.2912565	58	0.86118054	11195
THE PHOTOTRANSDUCTION CASCADE%REACTOME%R-HSA-2514856.1	0.45289558	1.2922859	19	0.86120003	4570
HEART VALVE MORPHOGENESIS%GOBP%GO:0003179	0.40426394	1.3055558	46	0.8612042	6191
MEMBRANE LIPID CATABOLIC PROCESS%GOBP%GO:0046466	0.41934434	1.2903537	28	0.86147684	6316
REGULATION OF CALCIUM ION TRANSMEMBRANE TRANSPORT%GOBP%GO:1903169	0.38309097	1.3019477	89	0.8615585	8086
REGULATION OF MITOTIC SISTER CHROMATID SEPARATION%GOBP%GO:0010965	0.39453265	1.2920136	56	0.86156064	9847
NEGATIVE REGULATION OF PROTEIN DEPHOSPHORYLATION%GOBP%GO:0035308	0.42627686	1.289308	27	0.8616136	3289
STIMULUS%GOBP%GO:1901522	0.4418926	1.2890995	21	0.86169666	2706
REGULATION OF MEMBRANE PERMEABILITY%GOBP%GO:0090559	0.3971954	1.3258685	70	0.8617452	8236
ACTIVATION OF HOX GENES DURING DIFFERENTIATION%REACTOME%R-HSA-5619507.3	0.38883156	1.3236691	80	0.86177844	5917
T CELL ACTIVATION%GOBP%GO:0042110	0.37953502	1.2909292	94	0.86179715	6569
NADH REGENERATION%GOBP%GO:0006735	0.42892766	1.2930192	25	0.86181456	4362
NEUROTROPHIN SIGNALING PATHWAY%GOBP%GO:0038179	0.44011232	1.2894535	21	0.8619562	4944
REGULATION OF AMYLOID PRECURSOR PROTEIN CATABOLIC PROCESS%GOBP%GO:1902991	0.40715706	1.2904451	38	0.8620635	6101
INTERSTRAND CROSS-LINK REPAIR%GOBP%GO:0036297	0.39153916	1.2912896	54	0.8621252	8740
POSITIVE REGULATION OF EPITHELIAL CELL MIGRATION%GOBP%GO:0010634	0.37757844	1.3265605	120	0.8621372	8196
SIGNALING BY NOTCH4%REACTOME%R-HSA-9013694.2	0.38476786	1.3055936	80	0.86228365	8913
REGULATION OF CARDIAC MUSCLE HYPERTROPHY%GOBP%GO:0010611	0.42112184	1.293742	29	0.8623928	9770
KERATINIZATION%GOBP%GO:0031424	0.41410977	1.3238163	45	0.8624143	5532
REGULATION OF CHOLESTEROL EFFLUX%GOBP%GO:0010874	0.42180413	1.293944	26	0.8624245	6121
POSITIVE REGULATION OF POTASSIUM ION TRANSMEMBRANE TRANSPORTER ACTIVITY%GOBP%GO:1901018	0.47059882	1.3254894	18	0.8625688	4165
SUPERPATHWAY OF INOSITOL PHOSPHATE COMPOUNDS%HUMANCYC%PWY-6371	0.3885553	1.2887285	65	0.8626495	6089
CORTICAL ACTIN CYTOSKELETON ORGANIZATION%GOBP%GO:0030866	0.4355967	1.2895247	23	0.86270887	11064
GAMMA CARBOXYLATION, HYPUSINE FORMATION AND ARYLSULFATASE ACTIVATION%REACTOME%R-HSA-163841.4	0.41167173	1.2880983	38	0.8627212	9825
CLEAVAGE INVOLVED IN RRNA PROCESSING%GOBP%GO:0000469	0.44657394	1.3266966	23	0.862825	875
PID_E2F_PATHWAY%MSIGDB_C2%PID_E2F_PATHWAY	0.40422076	1.3259281	56	0.86284447	4837
POSITIVE REGULATION OF ERBB SIGNALING PATHWAY%GOBP%GO:1901186	0.42372373	1.2930449	28	0.8628584	9727
CYTOSOLIC SENSORS OF PATHOGEN-ASSOCIATED DNA%REACTOME%R-HSA-1834949.1	0.3921378	1.2932397	60	0.8629339	8398
PID_PLK1_PATHWAY%MSIGDB_C2%PID_PLK1_PATHWAY	0.42899874	1.2940382	27	0.8630071	2901
RHOBTB GTPASE CYCLE%REACTOME%R-HSA-9706574.1	0.46230572	1.288458	16	0.8630134	8055
DISEASES ASSOCIATED WITH THE TLR SIGNALING CASCADE%REACTOME DATABASE ID RELEASE 75%5602358	0.44705975	1.3056958	21	0.86303365	6158
PID_INTEGRIN3_PATHWAY%MSIGDB_C2%PID_INTEGRIN3_PATHWAY	0.4137378	1.2934059	31	0.86316043	8376
OXIDATIVE PHOSPHORYLATION%GOBP%GO:0006119	0.37393028	1.2941915	105	0.8633364	6679
NEGATIVE REGULATION OF MET ACTIVITY%REACTOME%R-HSA-6807004.1	0.44947124	1.2881902	20	0.86334056	3385
NRAGE SIGNALS DEATH THROUGH JNK%REACTOME DATABASE ID RELEASE 75%193648	0.39545995	1.3009721	55	0.8634563	2999
REGULATION OF CALCINEURIN-MEDIATED SIGNALING%GOBP%GO:0106056	0.44633073	1.3268281	24	0.86356056	3097
PLATELET DEGRANULATION%GOBP%GO:0002576	0.38134253	1.3204316	105	0.86364746	3486
PURINE RIBONUCLEOTIDE CATABOLIC PROCESS%GOBP%GO:0009154	0.4482318	1.323841	22	0.8637108	662
HEART PROCESS%GOBP%GO:0003015	0.3967329	1.3201241	68	0.86405575	7057
RNA POLYMERASE II PROMOTER ESCAPE%REACTOME%R-HSA-73776.3	0.41652453	1.3227767	44	0.86408937	6737
REGULATION OF HYDROGEN PEROXIDE-INDUCED CELL DEATH%GOBP%GO:1903205	0.4724964	1.2942549	16	0.86412627	1532
POSITIVE REGULATION OF LYMPHOCYTE PROLIFERATION%GOBP%GO:0050671	0.39786732	1.3259581	71	0.8641462	5724
ELECTRON TRANSPORT CHAIN (OXPHOS SYSTEM IN MITOCHONDRIA)%WIKIPATHWAYS_20210110%WP111%HOMO SAPIENS	0.38202497	1.2872481	74	0.8641713	6679
GAMMA-AMINOBUTYRIC ACID SIGNALING PATHWAY%GOBP%GO:0007214	0.45306093	1.3057169	19	0.86421144	1839
CD4+ TCR PATHWAY	0.45711714	1.2876287	17	0.86424917	10501
AMB2 INTEGRIN SIGNALING%PATHWAY INTERACTION DATABASE NCI-NATURE CURATED DATA%AMB2 INTEGRIN SIGNALING	0.43459073	1.2870075	24	0.8643661	3503
MIRNA REGULATION OF PROSTATE CANCER SIGNALING PATHWAYS%WIKIPATHWAYS_20210110%WP3981%HOMO SAPIENS	0.43564498	1.324925	25	0.8644975	4202
FORMATION OF THE CORNIFIED ENVELOPE%REACTOME%R-HSA-6809371.3	0.42648956	1.3268714	32	0.8647604	7524
PRESYNAPTIC FUNCTION OF KAINATE RECEPTORS%REACTOME DATABASE ID RELEASE 75%500657	0.45256087	1.3204535	20	0.8649181	9314
DNA REPLICATION INITIATION%GOBP%GO:0006270	0.43097776	1.311277	27	0.864951	6258
ONCOGENE INDUCED SENESCENCE%REACTOME%R-HSA-2559585.6	0.428672	1.3216364	32	0.8650135	9790
SARCOMERE ORGANIZATION%GOBP%GO:0045214	0.45887452	1.294498	18	0.86508745	8020
MONOSACCHARIDE BIOSYNTHETIC PROCESS%GOBP%GO:0046364	0.4082595	1.3238556	41	0.8651027	9236
BIOCARTA_STRESS_PATHWAY%MSIGDB_C2%BIOCARTA_STRESS_PATHWAY	0.44662115	1.2942809	21	0.8651544	4417
PYRIMIDINE DEOXYRIBONUCLEOTIDE METABOLIC PROCESS%GOBP%GO:0009219	0.47883987	1.3273028	16	0.8651872	5891
EFFECTOR OF FAS AND TNF-ALPHA	0.41861477	1.2872517	29	0.8652948	4417
POSITIVE REGULATION OF RESPONSE TO ENDOPLASMIC RETICULUM STRESS%GOBP%GO:1905898	0.4234137	1.3062077	32	0.8653417	6568
5HT2 TYPE RECEPTOR MEDIATED SIGNALING PATHWAY%PANTHER PATHWAY%P04374	0.40228805	1.3064296	45	0.86538106	6094
POSITIVE REGULATION OF TYROSINE PHOSPHORYLATION OF STAT PROTEIN%GOBP%GO:0042531	0.41569754	1.305735	35	0.8654214	4466
REGULATION OF SPINDLE CHECKPOINT%GOBP%GO:0090231	0.4632568	1.2946253	15	0.8654911	10929
CORNIFICATION%GOBP%GO:0070268	0.405987	1.295007	42	0.865546	5532
REGULATION OF NATURAL KILLER CELL MEDIATED CYTOTOXICITY%GOBP%GO:0042269	0.45043477	1.298006	19	0.86561334	4842
NUCLEOTIDE-EXCISION REPAIR, DNA DUPLEX UNWINDING%GOBP%GO:0000717	0.4398822	1.2951832	22	0.8657122	4807
REGULATION OF MONOOXYGENASE ACTIVITY%GOBP%GO:0032768	0.4185651	1.321753	38	0.8657563	9404
GLYCOLYTIC PROCESS THROUGH GLUCOSE-6-PHOSPHATE%GOBP%GO:0061620	0.42892766	1.2981954	25	0.8657774	4362
THROMBIN SIGNALLING THROUGH PROTEINASE ACTIVATED RECEPTORS (PARS)%REACTOME%R-HSA-456926.1	0.43623504	1.3205574	27	0.86577904	9509
REGULATION OF KERATINOCYTE PROLIFERATION%GOBP%GO:0010837	0.4457682	1.3207786	22	0.865833	7338
PID_RXR_VDR_PATHWAY%MSIGDB_C2%PID_RXR_VDR_PATHWAY	0.45322987	1.2947602	18	0.8658566	6646
OXIDATIVE PHOSPHORYLATION%WIKIPATHWAYS_20210110%WP623%HOMO SAPIENS	0.41118768	1.3113445	40	0.86589956	6679
MULTIVESICULAR BODY ORGANIZATION%GOBP%GO:0036257	0.42896527	1.3269298	31	0.8659073	7828
MIRNAS INVOLVEMENT IN THE IMMUNE RESPONSE IN SEPSIS%WIKIPATHWAYS_20210110%WP4329%HOMO SAPIENS	0.44012937	1.3219723	24	0.8659122	5609
SPERM MOTILITY%GOBP%GO:0097722	0.42057973	1.327419	39	0.86600554	6473
SIGNALING EVENTS MEDIATED BY THE HEDGEHOG FAMILY%PATHWAY INTERACTION DATABASE NCI-NATURE CURATED DATA%SIGNALING EVENTS MEDIATED BY THE HEDGEHOG FAMILY	0.44639906	1.2864705	20	0.86623544	4911
REGULATION OF MITOTIC METAPHASE/ANAPHASE TRANSITION%GOBP%GO:0030071	0.39548135	1.309009	53	0.86631835	9847
POSTTRANSCRIPTIONAL GENE SILENCING%GOBP%GO:0016441	0.39700234	1.2991415	56	0.8663998	7405
GLYCOLYSIS%REACTOME%R-HSA-70171.6	0.39551872	1.3064768	62	0.86643595	5123
MEIOTIC CHROMOSOME SEPARATION%GOBP%GO:0051307	0.4761784	1.3057834	15	0.8664565	11025
REGULATION OF SYNAPTIC VESICLE CYCLE%GOBP%GO:0098693	0.40917683	1.2982751	36	0.86651766	4793
REGULATION OF ESTABLISHMENT OF ENDOTHELIAL BARRIER%GOBP%GO:1903140	0.47664672	1.3238633	16	0.86653155	2901
EMBRYONIC PATTERN SPECIFICATION%GOBP%GO:0009880	0.41780543	1.2969487	30	0.8665652	7372
HEART VALVE DEVELOPMENT%GOBP%GO:0003170	0.39945123	1.3208863	51	0.8666385	6191
ACTIVATION OF PHOSPHOLIPASE C ACTIVITY%GOBP%GO:0007202	0.45416245	1.2952278	20	0.86666423	5574
HSF1-DEPENDENT TRANSACTIVATION%REACTOME%R-HSA-3371571.1	0.43611062	1.2971457	24	0.86671066	2491
NUCLEOTIDE-SUGAR METABOLIC PROCESS%GOBP%GO:0009225	0.43766773	1.3220797	27	0.8667328	5673
NEURAL CREST CELL MIGRATION%GOBP%GO:0001755	0.41692162	1.2988721	33	0.86674434	7434
AMINO ACID IMPORT%GOBP%GO:0043090	0.4329338	1.2984422	24	0.86675763	2649
NEGATIVE REGULATION OF PROTEIN LOCALIZATION TO MEMBRANE%GOBP%GO:1905476	0.43011212	1.2997377	28	0.8668229	4209
RESPONSE TO MISFOLDED PROTEIN%GOBP%GO:0051788	0.4373371	1.299292	23	0.8668298	4425
REGULATION OF TRANSLATION IN RESPONSE TO STRESS%GOBP%GO:0043555	0.4711396	1.2973264	15	0.8669479	5362
GRANULOCYTE MIGRATION%GOBP%GO:0097530	0.405234	1.3113909	47	0.8669572	6153
GLYCOLYSIS%PANTHER PATHWAY%P00024	0.53830224	1.5306638	18	0.86696666	4362
CYTOSKELETON-DEPENDENT CYTOKINESIS%GOBP%GO:0061640	0.38510907	1.3072776	83	0.8670573	4988
POSTTRANSLATIONAL REGULATION OF ADHERENS JUNCTION STABILITY AND DISSASSEMBLY%PATHWAY INTERACTION DATABASE NCI-NATURE CURATED DATA%POSTTRANSLATIONAL REGULATION OF ADHERENS JUNCTION STABILITY AND DISSASSEMBLY	0.40325317	1.2966512	47	0.8670975	1727

TAK1 ACTIVATES NFKB BY PHOSPHORYLATION AND ACTIVATION OF IKKS COMPLEX%REACTOME%R-HSA-445989.3	0.4294807	1.2985855	26	0.86714697	4266
CELL-SUBSTRATE JUNCTION ASSEMBLY%GOBP%GO:0007044	0.4163913	1.2998979	33	0.86716825	9254
MITOCHONDRIAL CALCIUM ION HOMEOSTASIS%GOBP%GO:0051560	0.44431385	1.3074722	22	0.8672742	5549
GABA-B_RECEPTOR_II_SIGNALING%PANTHER PATHWAY%P05731	0.40704197	1.2953281	33	0.86731684	3211
REGULATION OF PROTEIN STABILITY%GOBP%GO:0031647	0.3663815	1.3115594	250	0.8673229	6445
CELL-CELL COMMUNICATION%REACTOME%R-HSA-1500931.4	0.37542918	1.2994249	103	0.867326	7574
ACTIN-MEDIATED CELL CONTRACTION%GOBP%GO:0070252	0.3870919	1.3065214	69	0.86748475	7804
PID_FRA_PATHWAY%MSIGDB_C2%PID_FRA_PATHWAY	0.43367016	1.327419	29	0.86751163	10576
CERAMIDE METABOLIC PROCESS%GOBP%GO:0006672	0.38547224	1.3090193	79	0.8675935	5213
MITOCHONDRIAL FUSION%GOBP%GO:0008053	0.4641196	1.2961308	18	0.86760354	5945
SIGNALING EVENTS MEDIATED BY FOCAL ADHESION KINASE%PATHWAY INTERACTION DATABASE NCI-NATURE CURATED DATA%SIGNALING EVENTS MEDIATED BY FOCAL ADHESION KINASE	0.38868052	1.3000246	57	0.86766	5243
CARGO CONCENTRATION IN THE ER%REACTOME%R-HSA-5694530.1	0.4232837	1.3278835	32	0.86772203	8320
TP53 NETWORK%WIKIPATHWAYS_20210110%WP1742%HOMO SAPIENS	0.48454946	1.3209412	15	0.86772424	2651
CELL-SUBSTRATE ADHESION%GOBP%GO:0031589	0.37963113	1.3239027	119	0.86775833	6547
NUCLEAR PORE COMPLEX (NPC) DISASSEMBLY%REACTOME%R-HSA-3301854.2	0.41408536	1.2973984	33	0.86781114	4994
REGULATION OF NOTCH SIGNALING PATHWAY%GOBP%GO:0008593	0.38507873	1.3119258	79	0.86792785	9519
NEGATIVE REGULATION OF EXOCYTOSIS%GOBP%GO:0045920	0.46834025	1.3275961	20	0.86796916	6814
CYTOSOLIC TRNA AMINOACYLATION%REACTOME%R-HSA-379716.1	0.4347876	1.3080195	24	0.8680351	8292
PID_ECADHERIN_STABILIZATION_PATHWAY%MSIGDB_C2%PID_ECADHERIN_STABILIZATION_PATHWAY	0.42016298	1.2954141	30	0.8680383	7649
PATHOGENIC ESCHERICHIA COLI INFECTION%WIKIPATHWAYS_20210110%WP2272%HOMO SAPIENS	0.4149491	1.3075511	39	0.8681639	5887
REGULATION OF RHO PROTEIN SIGNAL TRANSDUCTION%GOBP%GO:0035023	0.3969494	1.3091457	59	0.86818594	7265
REGULATION OF COLLAGEN METABOLIC PROCESS%GOBP%GO:0010712	0.4368863	1.2958218	21	0.8682198	8102
G BETA:GAMMA SIGNALLING THROUGH PLC BETA%REACTOME%R-HSA-418217.3	0.45387387	1.3240577	19	0.86828583	6094
MIDBODY ABSCISSION%GOBP%GO:0061952	0.4654919	1.3100432	17	0.86829555	2411
RUNX1 REGULATES TRANSCRIPTION OF GENES INVOLVED IN DIFFERENTIATION OF HSCS%REACTOME DATABASE ID RELEASE 75%8939236	0.34940058	1.1775388	85	0.86829674	9483
POSITIVE REGULATION OF ANGIOGENESIS%GOBP%GO:0045766	0.3778255	1.3066007	116	0.86830413	7365
LYMPHOCYTE MEDIATED IMMUNITY%GOBP%GO:0002449	0.40412414	1.3116151	54	0.86838096	3741
POSITIVE REGULATION OF B CELL ACTIVATION%GOBP%GO:0050871	0.40652436	1.3104825	44	0.86843514	8693
ORGANOPHOSPHATE CATABOLIC PROCESS%GOBP%GO:0046434	0.37663686	1.3093231	127	0.8684854	7601
HEXOSE BIOSYNTHETIC PROCESS%GOBP%GO:0019319	0.41967928	1.3077137	37	0.8685228	9236
ERBB4 SIGNALING EVENTS%PATHWAY INTERACTION DATABASE NCI-NATURE CURATED DATA%ERBB4 SIGNALING EVENTS	0.37571114	1.1776012	34	0.86859924	8059
ARRHYTHMOGENIC RIGHT VENTRICULAR CARDIOMYOPATHY%WIKIPATHWAYS_20210110%WP2118%HOMO SAPIENS	0.35787705	1.1780956	56	0.86862063	5247
CYCLIC NUCLEOTIDE METABOLIC PROCESS%GOBP%GO:0009187	0.4436867	1.2961618	22	0.868644	3159
REGUCALCIN IN PROXIMAL TUBULE EPITHELIAL KIDNEY CELLS%WIKIPATHWAYS_20210110%WP4838%HOMO SAPIENS	0.44001728	1.30951	23	0.86865145	7004
INTERLEUKIN-1 SIGNALING%REACTOME DATABASE ID RELEASE 75%9020702	0.3450682	1.178197	97	0.86875343	8274
AGGREPHAGY%REACTOME%R-HSA-9646399.3	0.44683513	1.3081195	23	0.86878324	8489
SIGNAL TRANSDUCTION BY P53 CLASS MEDIATOR%GOBP%GO:0072331	0.38358864	1.3188399	104	0.86878586	6397
CLEC7A (DECTIN-1) SIGNALING%REACTOME DATABASE ID RELEASE 75%5607764	0.34861103	1.1771694	91	0.8688391	8274
TNFSF8%IOB%TNFSF8	0.47024372	1.2954931	15	0.8688518	10344
PID_AP1_PATHWAY%MSIGDB_C2%PID_AP1_PATHWAY	0.3606482	1.1776505	48	0.86896193	10831
NEGATIVE REGULATION OF OSSIFICATION%GOBP%GO:0030279	0.36526534	1.1782682	45	0.8690021	2628
APC-CDC20 MEDIATED DEGRADATION OF NEK2A%REACTOME%R-HSA-179409.3	0.4318604	1.3066921	26	0.86910665	9837
DOWNREGULATION OF ERBB2 SIGNALING%REACTOME%R-HSA-8863795.2	0.38876343	1.1762178	26	0.86911404	10165
LEUKOCYTE PROLIFERATION%GOBP%GO:0070661	0.39486024	1.1750762	23	0.8691299	8250
MESODERM MORPHOGENESIS%GOBP%GO:0048332	0.43834975	1.3096621	24	0.86913717	5634
ION HOMEOSTASIS%REACTOME%R-HSA-5578775.2	0.403696	1.311952	48	0.86914724	5310
INTERACTION BETWEEN L1 AND ANKYRINS%REACTOME DATABASE ID RELEASE 75%445095	0.39016235	1.1772318	26	0.869152	4223
SYNTHESIS OF GLYCOSYLPHOSPHATIDYLINOSITOL (GPI)%REACTOME DATABASE ID RELEASE 75%162710	0.48252815	1.3278931	17	0.86916906	6403
VIRAL BUDDING VIA HOST ESCRT COMPLEX%GOBP%GO:0039702	0.52785933	1.5313438	21	0.8692026	8531
REGULATION OF ACTIN FILAMENT BUNDLE ASSEMBLY%GOBP%GO:0032231	0.346467	1.176446	77	0.8692028	6955
NEURAL CREST CELL MIGRATION DURING DEVELOPMENT%WIKIPATHWAYS_20210110%WP4564%HOMO SAPIENS	0.38948303	1.176058	27	0.869253	10579
MUSCLE CELL DEVELOPMENT%GOBP%GO:0055001	0.35507086	1.1778283	71	0.8692698	8020
WNT5A-DEPENDENT INTERNALIZATION OF FZD4%REACTOME DATABASE ID RELEASE 75%5099900	0.4303283	1.1797417	15	0.8693376	1332
INTEGRIN-MEDIATED SIGNALING PATHWAY%GOBP%GO:0007229	0.3971846	1.3189707	63	0.8693752	6802
EXTRACELLULAR VESICLE-MEDIATED SIGNALING IN RECIPIENT CELLS%WIKIPATHWAYS_20210110%WP2870%HOMO SAPIENS	0.3984007	1.1783085	21	0.8693937	5331
GLYCOSPHINGOLIPID METABOLISM%REACTOME%R-HSA-1660662.5	0.41306797	1.3184652	41	0.8694836	2425
REGULATION OF AXONOGENESIS%GOBP%GO:0050770	0.33964655	1.1789062	114	0.8695169	5503
DEGRADATION OF BETA-CATENIN BY THE DESTRUCTION COMPLEX%REACTOME DATABASE ID RELEASE 75%195253	0.34656993	1.1776545	80	0.8695374	3511
ACTIVATION OF GABAB RECEPTORS%REACTOME%R-HSA-991365.3	0.40815222	1.3100616	42	0.86954814	6719
BICELLULAR TIGHT JUNCTION ASSEMBLY%GOBP%GO:0070830	0.36868888	1.1751012	39	0.86961555	8039
RENAL WATER HOMEOSTASIS%GOBP%GO:0003091	0.3844467	1.1783861	27	0.86963195	1178
POSITIVE REGULATION OF NUCLEAR DIVISION%GOBP%GO:0051785	0.35350505	1.1758432	53	0.8696738	7911
MITOPHAGY%REACTOME DATABASE ID RELEASE 75%5205647	0.3876597	1.1762222	26	0.8696888	2992
GERM CELL DEVELOPMENT%GOBP%GO:0007281	0.3477	1.1764616	87	0.8697325	9659
TIGHT JUNCTION ORGANIZATION%GOBP%GO:0120193	0.36388597	1.1748097	42	0.8697491	8039
FC-GAMMA RECEPTOR SIGNALING PATHWAY INVOLVED IN PHAGOCYTOSIS%GOBP%GO:0038096	0.35279727	1.1790963	63	0.86986786	6350
LEISHMANIA INFECTION%REACTOME%R-HSA-9658195.2	0.33351097	1.17683	187	0.8698683	6299
NOVEL INTRACELLULAR COMPONENTS OF RIG-I-LIKE RECEPTOR (RLR) PATHWAY%WIKIPATHWAYS_20210110%WP3865%HOMO SAPIENS	0.40945953	1.3148575	46	0.86992574	5648
NEGATIVE REGULATION OF PROTEIN COMPLEX DISASSEMBLY%GOBP%GO:0043242	0.39213738	1.2802465	52	0.8699333	8104
REGULATION OF MUSCLE ADAPTATION%GOBP%GO:0043502	0.37716272	1.1797435	35	0.8699334	9770
POSTSYNAPSE ORGANIZATION%GOBP%GO:0099173	0.3630605	1.175149	50	0.87000626	2107
REGULATION OF AMINE TRANSPORT%GOBP%GO:0051952	0.3609685	1.1784323	52	0.87000793	9573
COMPLEX I BIOGENESIS%REACTOME%R-HSA-6799198.1	0.395243	1.3081323	55	0.870043	6736
AQUAPORIN-MEDIATED TRANSPORT%REACTOME%R-HSA-445717.2	0.36542138	1.1789125	42	0.8700888	6560
PEPTIDYL-THREONINE PHOSPHORYLATION%GOBP%GO:0018107	0.35218814	1.1752425	61	0.870156	7719
RECEPTOR INTERNALIZATION%GOBP%GO:0031623	0.37108037	1.1803308	45	0.87018037	8598
REGULATION OF UBIQUITIN-DEPENDENT PROTEIN CATABOLIC PROCESS%GOBP%GO:2000058	0.3371163	1.1799409	134	0.87018114	7254
CELLULAR POLYSACCHARIDE BIOSYNTHETIC PROCESS%GOBP%GO:0033692	0.42561582	1.3279576	35	0.87030435	4449
HAIR FOLLICLE DEVELOPMENT: CYTODIFFERENTIATION (PART 3 OF 3)%WIKIPATHWAYS_20210110%WP2840%HOMO SAPIENS	0.35589635	1.1764681	58	0.8703083	5634
POSITIVE REGULATION OF VIRAL PROCESS%GOBP%GO:0048524	0.38249186	1.3131574	103	0.8703278	6975
RESPONSE TO RETINOIC ACID%GOBP%GO:0032526	0.3558272	1.179121	53	0.8703434	6955
IL4%NETPATH%IL4	0.3574181	1.17454	50	0.8704111	4268
CELL JUNCTION ASSEMBLY%GOBP%GO:0034329	0.37112176	1.3119665	136	0.8704286	5922
STRIATED MUSCLE CELL DEVELOPMENT%GOBP%GO:0055002	0.36092165	1.1801422	58	0.8704536	8020
PHASE II - CONJUGATION OF COMPOUNDS%REACTOME DATABASE ID RELEASE 75%156580	0.35049596	1.17976	70	0.8704615	5007
HEMATOPOIETIC STEM CELL GENE REGULATION BY GABP ALPHA/BETA COMPLEX%WIKIPATHWAYS_20210110%WP3657%HOMO SAPIENS	0.45747012	1.2674373	16	0.87046164	3938
PREASSEMBLY OF GPI ANCHOR IN ER MEMBRANE%GOBP%GO:0016254	0.41672558	1.1804003	17	0.8704701	6403
SIGNALING BY CYTOSOLIC FGFR1 FUSION MUTANTS%REACTOME DATABASE ID RELEASE 75%1839117	0.46259427	1.280339	16	0.8705433	7425
SEMI-LUNAR VALVE DEVELOPMENT%GOBP%GO:1905314	0.37524763	1.1752856	36	0.8705558	8580
TNFSF1%IOB%TNFSF1	0.4293776	1.1784387	15	0.87057465	5208
EPHA FORWARD SIGNALING%PATHWAY INTERACTION DATABASE NCI-NATURE CURATED DATA%EPHA FORWARD SIGNALING	0.4192122	1.2785336	27	0.8706874	5361
SIGNALING BY ERBB4%REACTOME DATABASE ID RELEASE 75%1236394	0.38784254	1.2670346	51	0.8706883	8401
VASCULAR ENDOTHELIAL GROWTH FACTOR RECEPTOR SIGNALING PATHWAY%GOBP%GO:0048010	0.35360125	1.1734862	61	0.87069774	6350
ACTIVATION OF ANTERIOR HOX GENES IN HINDBRAIN DEVELOPMENT DURING EARLY EMBRYOGENESIS%REACTOME%R-HSA-5617472.2	0.38883156	1.3128601	80	0.8707281	5917
POSITIVE REGULATION OF RHO PROTEIN SIGNAL TRANSDUCTION%GOBP%GO:0035025	0.40768978	1.1799495	20	0.87074983	6319
MET ACTIVATES PTK2 SIGNALING%REACTOME DATABASE ID RELEASE 75%8874081	0.47375453	1.3286182	17	0.87078846	7302
NON-CODING RNA METABOLISM%REACTOME%R-HSA-194441.1	0.3571843	1.1764934	50	0.8707906	7797
PID_RAC1_REG_PATHWAY%MSIGDB_C2%PID_RAC1_REG_PATHWAY	0.43831375	1.3135347	23	0.87079656	10236
BIOCARTA_BCR_PATHWAY%MSIGDB_C2%BIOCARTA_BCR_PATHWAY	0.3965857	1.1791549	23	0.87080264	10110
REGULATION OF CENTRIOLE REPLICATION%GOBP%GO:0046599	0.40450135	1.1732019	22	0.87085193	8711
NUCLEOSIDE METABOLIC PROCESS%GOBP%GO:0009116	0.35064194	1.1784984	73	0.8708817	5423
TP53 REGULATES TRANSCRIPTION OF CELL DEATH GENES%REACTOME DATABASE ID RELEASE 75%5633008	0.3703965	1.1730536	41	0.8709316	6040
NEGATIVE REGULATION OF RESPONSE TO BIOTIC STIMULUS%GOBP%GO:0002832	0.3836471	1.179247	30	0.8709712	4545
REGULATION OF INTERFERON-GAMMA-MEDIATED SIGNALING PATHWAY%GOBP%GO:0060334	0.41644862	1.1804222	18	0.87098336	1297
REGULATION OF MUSCLE HYPERTROPHY%GOBP%GO:0014743	0.42112184	1.2814585	29	0.87100065	9770
NEGATIVE REGULATION OF MRNA SPLICING, VIA SPLICEOSOME%GOBP%GO:0048025	0.42945	1.1752987	15	0.8710886	4952
DOWNSTREAM TCR SIGNALING%REACTOME%R-HSA-202424.4	0.37544432	1.2786527	87	0.87110305	8770
MEMBRANE FUSION%GOBP%GO:0061025	0.36568907	1.2665862	114	0.87110853	2509
POSITIVE REGULATION OF MONONUCLEAR CELL PROLIFERATION%GOBP%GO:0032946	0.391765	1.3148905	72	0.87111014	5724
PHOTODYNAMIC THERAPY-INDUCED NFE2L2 (NRF2) SURVIVAL SIGNALING%WIKIPATHWAYS_20210110%WP3612%HOMO SAPIENS	0.41395906	1.1735195	17	0.8711307	4681
BIOCARTA_HCMV_PATHWAY%MSIGDB_C2%BIOCARTA_HCMV_PATHWAY	0.42585123	1.1741401	15	0.871173	10095
GOLGI VESICLE BUDDING%GOBP%GO:0048194	0.37599874	1.2831954	77	0.871189	9298
RIBONUCLEOPROTEIN COMPLEX DISASSEMBLY%GOBP%GO:0032988	0.46877575	1.2663903	15	0.8711906	2975
INFLAMMATION MEDIATED BY CHEMOKINE AND CYTOKINE SIGNALING PATHWAY%PANTHER PATHWAY%P00031	0.33363602	1.1732476	161	0.8712149	6248
CITRIC ACID CYCLE (TCA CYCLE)%REACTOME DATABASE ID RELEASE 75%71403	0.43420607	1.2816018	22	0.8713046	856
PYRUVATE METABOLISM AND CITRIC ACID (TCA) CYCLE%REACTOME DATABASE ID RELEASE 75%71406	0.36039922	1.1742375	50	0.8713209	5317
CARDIAC CONDUCTION%GOBP%GO:0061337	0.3889814	1.315086	83	0.8713438	7588
RESPONSE TO OSMOTIC STRESS%GOBP%GO:0006970	0.37305543	1.1804742	34	0.8713553	5724
SYNTHESIS OF IP3 AND IP4 IN THE CYTOSOL%REACTOME DATABASE ID RELEASE 75%1855204	0.42858544	1.2796179	26	0.8713608	7110
P53 TRANSCRIPTIONAL GENE NETWORK%WIKIPATHWAYS_20210110%WP4963%HOMO SAPIENS	0.35945258	1.180601	56	0.87136513	3242
VASCULAR ENDOTHELIAL GROWTH FACTOR SIGNALING PATHWAY%GOBP%GO:0038084	0.46356407	1.3280321	19	0.87138236	7352
DISEASES OF IMMUNE SYSTEM%REACTOME%R-HSA-5260271.2	0.44705975	1.3125042	21	0.87141144	6158
PHOTORECEPTOR CELL DIFFERENTIATION%GOBP%GO:0046530	0.41599333	1.279199	27	0.8714426	2387
RESPONSE TO ALKALOID%GOBP%GO:0043279	0.42800906	1.2674426	21	0.8714574	3564
ACETYL-COA METABOLIC PROCESS%GOBP%GO:0006084	0.44977248	1.2839272	20	0.87146467	2554
SYNTHESIS OF PA%REACTOME DATABASE ID RELEASE 75%1483166	0.41650295	1.2803725	31	0.87146986	5174

CELL-CELL RECOGNITION%GOBP%GO:0009988	0.42757827	1.313202	28	0.8714742	1958
NADH METABOLIC PROCESS%GOBP%GO:0006734	0.41909203	1.3120221	34	0.87147695	4362
NEGATIVE REGULATION OF INTERLEUKIN-1 PRODUCTION%GOBP%GO:0032692	0.42455506	1.1736944	15	0.8715141	6935
CELL MIGRATION AND INVASION THROUGH P75NTR%WIKIPATHWAYS_20210110%WP4561%HOMO SAPIENS	0.39200142	1.1735628	25	0.8715213	6191
INWARDLY RECTIFYING K+ CHANNELS%REACTOME DATABASE ID RELEASE 75%1296065	0.42406923	1.313648	32	0.8715294	7129
MITOTIC G1 DNA DAMAGE CHECKPOINT%GOBP%GO:0031571	0.38459986	1.2841048	61	0.87154627	6040
CONSTITUTIVE SIGNALING BY NOTCH1 PEST DOMAIN MUTANTS%REACTOME DATABASE ID RELEASE 75%2644606	0.3863104	1.2666926	56	0.87155735	8274
EUKARYOTIC TRANSCRIPTION INITIATION%WIKIPATHWAYS_20210110%WP405%HOMO SAPIENS	0.41130948	1.2805525	32	0.87157613	5103
REGULATION OF NUCLEAR DIVISION%GOBP%GO:0051783	0.36221337	1.2670532	148	0.8715915	9847
MODIFICATION BY HOST OF SYMBIONT MORPHOLOGY OR PHYSIOLOGY%GOBP%GO:0051851	0.39381292	1.2809508	54	0.87160593	5991
NEGATIVE REGULATION OF MITOTIC METAPHASE/ANAPHASE TRANSITION%GOBP%GO:0045841	0.40899327	1.1753169	22	0.8716078	8983
PROTEIN PROCESSING%GOBP%GO:0016485	0.367106	1.279767	124	0.87164754	8887
DEFECTIVE BINDING OF RB1 MUTANTS TO E2F1,(E2F2, E2F3)%REACTOME%R-HSA-9661069.1	0.47310838	1.3122268	16	0.87165946	4567
MEMBRANE LIPID METABOLIC PROCESS%GOBP%GO:0006643	0.3607745	1.2724543	176	0.8717199	5803
NEGATIVE REGULATION OF DEFENSE RESPONSE%GOBP%GO:0031348	0.37892628	1.3140887	122	0.87172294	6229
REGULATION OF VENTRICULAR CARDIAC MUSCLE CELL MEMBRANE REPOLARIZATION%GOBP%GO:0060307	0.44825408	1.2660922	16	0.8717659	5620
NADH DEHYDROGENASE COMPLEX ASSEMBLY%GOBP%GO:0010257	0.39926827	1.3287083	65	0.8717743	6741
INTRINSIC APOPTOTIC SIGNALING PATHWAY IN RESPONSE TO ENDOPLASMIC RETICULUM STRESS%GOBP%GO:0070059	0.3946938	1.1726053	27	0.87184644	5362
APC C:CDC20 MEDIATED DEGRADATION OF MITOTIC PROTEINS%REACTOME%R-HSA-176409.3	0.34699005	1.1737447	74	0.87184936	9837
ALZHEIMER DISEASE-PRESENILIN PATHWAY%PANTHER PATHWAY%P00004	0.3397929	1.1738687	104	0.87185407	6299
APICAL JUNCTION ASSEMBLY%GOBP%GO:0043297	0.39799866	1.2832708	46	0.8718763	8039
INTRACELLULAR SIGNAL TRANSDUCTION INVOLVED IN G1 DNA DAMAGE CHECKPOINT%GOBP%GO:1902400	0.40771866	1.3297055	56	0.8719143	6040
PURINE NUCLEOSIDE METABOLIC PROCESS%GOBP%GO:0042278	0.37084368	1.1806177	41	0.8719176	5423
MEMBRANE LIPID BIOSYNTHETIC PROCESS%GOBP%GO:0046467	0.37782067	1.3138129	122	0.8719205	5803
PID_EPHA_FWDPATHWAY%MSIGDB_C2%PID_EPHA_FWDPATHWAY	0.43199384	1.2816799	22	0.8719848	5361
INTERACTION WITH SYMBIONT%GOBP%GO:0051702	0.38931286	1.2762052	57	0.87199795	5991
REGULATION OF ACTIN POLYMERIZATION OR DEPOLYMERIZATION%GOBP%GO:0008064	0.3638899	1.2725928	137	0.8720309	6307
ANATOMICAL STRUCTURE MATURATION%GOBP%GO:0071695	0.38475955	1.2786722	51	0.872088	2885
AGE/RAGE PATHWAY%WIKIPATHWAYS_20210110%WP2324%HOMO SAPIENS	0.3933475	1.2777227	48	0.87211484	10153
NEGATIVE REGULATION OF METAPHASE/ANAPHASE TRANSITION OF CELL CYCLE%GOBP%GO:1902100	0.43522772	1.2818462	23	0.8721215	2966
CELLULAR RESPONSE TO ARSENIC-CONTAINING SUBSTANCE%GOBP%GO:0071243	0.45134488	1.2779155	17	0.87212574	6249
REGULATION OF PHOSPHOPROTEIN PHOSPHATASE ACTIVITY%GOBP%GO:0043666	0.39732394	1.2765387	40	0.8723008	8400
POSITIVE REGULATION OF PROTEIN LOCALIZATION TO PLASMA MEMBRANE%GOBP%GO:1903078	0.39432183	1.2806154	46	0.8723327	5087
NEURON MIGRATION%GOBP%GO:0001764	0.38409576	1.2674689	54	0.87234277	5930
SIGNALING BY NOTCH1%REACTOME DATABASE ID RELEASE 75%1980143	0.3819266	1.2820003	71	0.8723465	8274
REGULATION OF RAS PROTEIN SIGNAL TRANSDUCTION%GOBP%GO:0046578	0.33776194	1.1726276	123	0.8723474	7338
TERMINATION OF RNA POLYMERASE I TRANSCRIPTION%GOBP%GO:0006363	0.43245596	1.3298819	27	0.8724429	6737
UNCERTAIN%REACTOME%R-HSA-6804115.1	0.43693826	1.2788106	21	0.8724451	3556
PLASMA LIPOPROTEIN CLEARANCE%REACTOME DATABASE ID RELEASE 75%8964043	0.42510998	1.2851567	26	0.87244934	8486
MECP2 AND ASSOCIATED RETT SYNDROME%WIKIPATHWAYS_20210110%WP3584%HOMO SAPIENS	0.36130387	1.1806321	53	0.8724715	6298
REGULATION OF CHROMOSOME SEPARATION%GOBP%GO:1905818	0.3805867	1.276708	61	0.8724773	9847
EPITHELIAL TO MESENCHYMAL TRANSITION IN COLORECTAL CANCER%WIKIPATHWAYS_20210110%WP4239%HOMO SAPIENS	0.37233856	1.2792084	113	0.87248373	8582
RNA POL II CTD PHOSPHORYLATION AND INTERACTION WITH CE DURING HIV INFECTION%REACTOME%R-HSA-167160.2	0.42121497	1.2841237	27	0.87256044	6737
HISTONE DEUBIQUITINATION%GOBP%GO:0016578	0.43151903	1.2749491	22	0.87257534	6210
VESICLE TARGETING, ROUGH ER TO CIS-GOLGI%GOBP%GO:0048207	0.3766579	1.2736273	63	0.8725828	9586
CHEMOKINE SIGNALING PATHWAY%WIKIPATHWAYS_20210110%WP3929%HOMO SAPIENS	0.3735537	1.280973	103	0.8725924	6134
CYTOPLASMIC TRANSLATION%GOBP%GO:0002181	0.35093436	1.1684434	62	0.87259513	6626
REGULATION OF INTERFERON-ALPHA PRODUCTION%GOBP%GO:0032647	0.4146966	1.1680505	19	0.8726863	4751
VESICLE TARGETING, TO, FROM OR WITHIN GOLGI%GOBP%GO:0048199	0.3788774	1.2732213	70	0.87269115	9298
ABERRANT REGULATION OF MITOTIC G1 S TRANSITION IN CANCER DUE TO RB1 DEFECTS%REACTOME DATABASE ID RELEASE 75%9659787	0.47310838	1.3141613	16	0.8726953	4567
GLYCOLIPID METABOLIC PROCESS%GOBP%GO:0006664	0.38749626	1.3280673	95	0.8726976	5803
TNF RECEPTOR SIGNALING PATHWAY%PATHWAY INTERACTION DATABASE NCI-NATURE CURATED DATA%TNF RECEPTOR SIGNALING PATHWAY	0.39392796	1.2780055	43	0.87272114	6538
REGULATION OF METAPHASE/ANAPHASE TRANSITION OF CELL CYCLE%GOBP%GO:1902099	0.40524977	1.3150893	54	0.8727286	9847
REGULATION OF G PROTEIN-COUPLED RECEPTOR SIGNALING PATHWAY%GOBP%GO:0008277	0.34274665	1.16852	96	0.8727936	7219
CANONICAL GLYCOLYSIS%GOBP%GO:0061621	0.42892766	1.2833105	25	0.8728163	4362
PEPTIDE LIGAND-BINDING RECEPTORS%REACTOME%R-HSA-375276.5	0.3725761	1.2730079	115	0.8728383	6610
ERK1 AND ERK2 CASCADE%GOBP%GO:0070371	0.4505037	1.2848799	20	0.87285906	7719
BIOCARTA_ALK_PATHWAY%MSIGDB_C2%BIOCARTA_ALK_PATHWAY	0.43424693	1.3303174	29	0.8728992	6318
BIOCARTA_HIVNEF_PATHWAY%MSIGDB_C2%BIOCARTA_HIVNEF_PATHWAY	0.40653113	1.2846638	37	0.8728999	5356
ERBB SIGNALING PATHWAY%WIKIPATHWAYS_20210110%WP673%HOMO SAPIENS	0.34414673	1.1694838	72	0.8729019	11091
CYTOSKELETAL REGULATION BY RHO GTPASE%PANTHER PATHWAY%P00016	0.38774195	1.2774001	63	0.8729142	5884
MITOTIC CELL CYCLE ARREST%GOBP%GO:0071850	0.472434	1.2768077	15	0.8729259	4662
AORTIC VALVE DEVELOPMENT%GOBP%GO:0003176	0.41159067	1.2762284	31	0.8729545	6191
RNA POL II CTD PHOSPHORYLATION AND INTERACTION WITH CE%REACTOME DATABASE ID RELEASE 75%77075	0.42121497	1.2820928	27	0.8729661	6737
OSTEOBLAST DIFFERENTIATION%WIKIPATHWAYS_20210110%WP4787%HOMO SAPIENS	0.37689918	1.27261	84	0.87297887	5640
G1 TO S CELL CYCLE CONTROL%WIKIPATHWAYS_20210110%WP45%HOMO SAPIENS	0.3642751	1.169952	46	0.87298393	6377
CELL CYCLE%PANTHER PATHWAY%P00013	0.42464906	1.1713506	15	0.87299216	3377
DIOL METABOLIC PROCESS%GOBP%GO:0034311	0.43010908	1.2727964	24	0.8730017	5616
NEGATIVE REGULATION OF INFLAMMATORY RESPONSE%GOBP%GO:0050728	0.39535436	1.329271	70	0.8730221	5213
ADHERENS JUNCTIONS INTERACTIONS%REACTOME DATABASE ID RELEASE 75%418990	0.38675743	1.1806407	28	0.8730466	6938
POTASSIUM CHANNELS%REACTOME%R-HSA-1296071.2	0.35817307	1.2177051	87	0.8730517	6719
MALE GAMETE GENERATION%GOBP%GO:0048232	0.32975864	1.1680758	176	0.87312734	5296
VESICLE COATING%GOBP%GO:0006901	0.38058305	1.2677073	67	0.87312925	9298
REGULATION OF MITOCHONDRION ORGANIZATION%GOBP%GO:0010821	0.3343716	1.1681947	160	0.87315226	9849
NEGATIVE REGULATION OF MITOTIC SISTER CHROMATID SEPARATION%GOBP%GO:2000816	0.44333798	1.3152465	23	0.8731728	2966
SIGNALING BY NOTCH1 HD+PEST DOMAIN MUTANTS IN CANCER%REACTOME DATABASE ID RELEASE 75%2894858	0.3863104	1.2742766	56	0.873177	8274
MATURATION OF SSU-RRNA%GOBP%GO:0030490	0.3934839	1.2750345	42	0.8731788	8277
LEUKOCYTE MEDIATED CYTOTOXICITY%GOBP%GO:0001909	0.44166547	1.2746507	20	0.8731823	3465
REGULATION OF INTRINSIC APOPTOTIC SIGNALING PATHWAY%GOBP%GO:2001242	0.36748195	1.2769521	118	0.8731927	6568
PID_ECADHERIN_KERATINOCYTE_PATHWAY%MSIGDB_C2%PID_ECADHERIN_KERATINOCYTE_PATHWAY	0.4346241	1.1695307	15	0.873252	10378
MITOTIC SISTER CHROMATID SEGREGATION%GOBP%GO:0000070	0.3372393	1.1685454	104	0.87325215	12942
REGULATION OF MITOTIC CELL CYCLE SPINDLE ASSEMBLY CHECKPOINT%GOBP%GO:0090266	0.4632568	1.2771393	15	0.8732715	10929
REGULATION OF TRANSCRIPTION FROM RNA POLYMERASE II PROMOTER IN RESPONSE TO HYPOXIA%GOBP%GO:0061418	0.37827346	1.2674857	72	0.8732763	8274
RESPONSE TO ATP%GOBP%GO:0033198	0.48298937	1.3287153	16	0.87328464	564
NON-SMALL CELL LUNG CANCER%WIKIPATHWAYS_20210110%WP4255%HOMO SAPIENS	0.3681781	1.2178049	59	0.8732868	4567
CELLULAR RESPONSE TO GAMMA RADIATION%GOBP%GO:0071480	0.4548617	1.2826304	17	0.8732949	6145
CYTOPLASMIC PATTERN RECOGNITION RECEPTOR SIGNALING PATHWAY%GOBP%GO:0002753	0.372828	1.1703811	36	0.87332284	9917
CELLULAR RESPONSE TO XENOBIOTIC STIMULUS%GOBP%GO:0071466	0.33734077	1.1687686	92	0.8733288	5320
HOMOLOGOUS CHROMOSOME SEGREGATION%GOBP%GO:0045143	0.4353412	1.2824211	23	0.87333506	1675
CELL CHEMOTAXIS%GOBP%GO:0060326	0.35372397	1.2180879	111	0.8733465	6935
REGULATION OF T CELL ACTIVATION%GOBP%GO:0050863	0.3599098	1.2822188	179	0.8733567	5108
SELECTIVE AUTOPHAGY%GOBP%GO:0061912	0.4109776	1.2736605	31	0.8734625	6862
SIGNALING BY FGFR2 IN DISEASE%REACTOME DATABASE ID RELEASE 75%5655253	0.39493167	1.2179167	32	0.8734695	5602
INNATE IMMUNE RESPONSE ACTIVATING CELL SURFACE RECEPTOR SIGNALING PATHWAY%GOBP%GO:0002220	0.34340572	1.1705974	93	0.87348026	5498
EXCRETION%GOBP%GO:0007588	0.4426916	1.2753679	18	0.8734827	5169
CELLULAR RESPONSE TO RADIATION%GOBP%GO:0071478	0.33595425	1.171366	126	0.8735069	4807
MRNA CAPPING%REACTOME DATABASE ID RELEASE 75%72086	0.38709322	1.1699631	29	0.8735122	6737
DNA UNWINDING INVOLVED IN DNA REPLICATION%GOBP%GO:0006268	0.42727405	1.1688511	15	0.873513	8062
NEGATIVE REGULATION OF TRANSPORTER ACTIVITY%GOBP%GO:0032410	0.38875633	1.2683899	55	0.87354016	4230
PID_NFAT_TFPATHWAY%MSIGDB_C2%PID_NFAT_TFPATHWAY	0.42782196	1.2833825	24	0.8735503	10501
AURKA ACTIVATION BY TPX2%REACTOME%R-HSA-8854518.3	0.351632	1.1711016	71	0.87356216	9743
SMALL GTPASE MEDIATED SIGNAL TRANSDUCTION%GOBP%GO:0007264	0.35895696	1.2743965	176	0.87356627	7389
NEUROTRANSMITTER BIOSYNTHETIC PROCESS%GOBP%GO:0042136	0.37657803	1.1695789	31	0.8735914	6194
GLUCOSE CATABOLIC PROCESS TO PYRUVATE%GOBP%GO:0061718	0.42892766	1.2732472	25	0.87360924	4362
CHROMOSOMAL AND MICROSATELLITE INSTABILITY IN COLORECTAL CANCER %WIKIPATHWAYS_20210110%WP4216%HOMO SAPIENS	0.3894785	1.2841405	60	0.87361896	7719
NEGATIVE REGULATION OF G1/S TRANSITION OF MITOTIC CELL CYCLE%GOBP%GO:2000134	0.34271064	1.1806513	92	0.87362367	6057
PLASMA MEMBRANE INVAGINATION%GOBP%GO:0099024	0.36676458	1.1708266	39	0.8736333	8987
LEUKOCYTE CELL-CELL ADHESION%GOBP%GO:0007159	0.37491733	1.1685815	33	0.87363905	4862
SIGNALING BY HEDGEHOG%REACTOME DATABASE ID RELEASE 75%5358351	0.3332835	1.1718329	129	0.8736453	8770
PEPTIDYL-S-DIACYLGLYCEROL-L-CYSTEINE BIOSYNTHETIC PROCESS FROM PEPTIDYL-CYSTEINE%GOBP%GO:0018231	0.39728522	1.1704352	23	0.8736491	5507
CYTOPLASMIC TRANSLATIONAL INITIATION%GOBP%GO:0002183	0.5271335	1.5318483	20	0.87367845	6626
PROTEIN-DNA COMPLEX ASSEMBLY%GOBP%GO:0065004	0.33423164	1.1691881	136	0.87367874	8824
POSITIVE REGULATION OF RELEASE OF CYTOCHROME C FROM MITOCHONDRIA%GOBP%GO:0090200	0.4522703	1.3289046	23	0.8737069	7910
POSITIVE REGULATION OF UBIQUITIN-DEPENDENT PROTEIN CATABOLIC PROCESS%GOBP%GO:2000060	0.34341574	1.1714399	86	0.8737448	7254
REGULATION OF PLATELET-DERIVED GROWTH FACTOR RECEPTOR SIGNALING PATHWAY%GOBP%GO:0010640	0.41052404	1.1701651	17	0.8737475	4220
CLATHRIN COAT ASSEMBLY%GOBP%GO:0048268	0.45933238	1.2756946	15	0.87378335	9281
REGULATION OF ACTIN CYTOSKELETON%WIKIPATHWAYS_20210110%WP51%HOMO SAPIENS	0.36925963	1.273784	100	0.8737883	6264
GAP-FILLING DNA REPAIR SYNTHESIS AND LIGATION IN TC-NER%REACTOME%R-HSA-6782210.1	0.39107668	1.3153737	64	0.8738072	8457
PID_CD40_PATHWAY%MSIGDB_C2%PID_CD40_PATHWAY	0.40023765	1.1706513	22	0.87382066	5640
SOMATIC DIVERSIFICATION OF IMMUNE RECEPTORS%GOBP%GO:0002200	0.4390623	1.3299129	28	0.8738267	2943
ACTIVATION OF BH3-ONLY PROTEINS%REACTOME DATABASE ID RELEASE 75%114452	0.43270412	1.3304093	29	0.8738435	4030
REGULATION OF CALCIUM ION TRANSPORT%GOBP%GO:0051924	0.3347594	1.1812427	138	0.8738652	5106
GLYCOLIPID BIOSYNTHETIC PROCESS%GOBP%GO:0009247	0.35235813	1.1716628	61	0.87386876	5699
PURINE-CONTAINING COMPOUND CATABOLIC PROCESS%GOBP%GO:0072523	0.40153208	1.2690747	39	0.8738927	6312
POSITIVE REGULATION OF CREB TRANSCRIPTION FACTOR ACTIVITY%GOBP%GO:0032793	0.44869518	1.2754815	17	0.8739271	1669
PHYSIOLOGICAL AND PATHOLOGICAL HYPERTROPHY OF THE HEART%WIKIPATHWAYS_20210110%WP1528%HOMO SAPIENS	0.44962662	1.2718657	18	0.8739499	10110
XENOBIOTIC METABOLIC PROCESS%GOBP%GO:0006805	0.347764	1.1688765	78	0.8739687	5682

DEOXYRIBONUCLEOTIDE METABOLIC PROCESS%GOBP%GO:0009262	0.38626695	1.1718873	28	0.87396944	5891
FORMATION OF RNA POL II ELONGATION COMPLEX%REACTOME%R-HSA-112382.2	0.35413688	1.167647	57	0.87397337	7447
POSITIVE REGULATION OF CARDIAC MUSCLE TISSUE GROWTH%GOBP%GO:0055023	0.42170125	1.169984	16	0.87398654	5621
NEGATIVE REGULATION OF RESPONSE TO ENDOPLASMIC RETICULUM STRESS%GOBP%GO:1903573	0.39237237	1.2842673	47	0.87401354	5841
CELLULAR RESPONSE TO MISFOLDED PROTEIN%GOBP%GO:0071218	0.4232166	1.2181046	20	0.87401825	4425
RECEPTOR ACTIVITY	0.36293277	1.1714982	49	0.87403965	6646
INTERACTIONS	0.4111773	1.1708609	16	0.87407273	7759
NEGATIVE REGULATION OF MRNA CATABOLIC PROCESS%GOBP%GO:1902373	0.36547077	1.1813335	45	0.8740838	1885
ABERRANT REGULATION OF MITOTIC CELL CYCLE DUE TO RB1 DEFECTS%REACTOME%R-HSA-9687139.2	0.37350905	1.169597	35	0.8740847	7603
CYTOKINESIS%GOBP%GO:0000910	0.37623426	1.2750658	85	0.87409145	4988
MEMBRANE PROTEIN INTRACELLULAR DOMAIN PROTEOLYSIS%GOBP%GO:0031293	0.43091202	1.2677128	22	0.8741316	7398
NUCLEOBASE-CONTAINING SMALL MOLECULE BIOSYNTHETIC PROCESS%GOBP%GO:0034404	0.36657712	1.2680943	114	0.8741441	5021
WNT SIGNALING PATHWAY, CALCIUM MODULATING PATHWAY%GOBP%GO:0007223	0.3743978	1.1806589	32	0.87420976	7526
PID_P53_REGULATION_PATHWAY%MSIGDB_C2%PID_P53_REGULATION_PATHWAY	0.36064863	1.1814123	47	0.87430257	5632
RECOGNITION AND ASSOCIATION OF DNA GLYCOSYLASE WITH SITE CONTAINING AN AFFECTED PURINE%REACTOME%R-HSA-110330.3	0.38191703	1.1833508	27	0.8743087	5210
NEGATIVE REGULATION OF ION TRANSMEMBRANE TRANSPORT%GOBP%GO:0034766	0.3479899	1.1689285	63	0.87433285	4230
IMMUNE RESPONSE-REGULATING CELL SURFACE RECEPTOR SIGNALING PATHWAY INVOLVED IN PHAGOCYTOSIS%GOBP%GO:0002433	0.35279727	1.1719366	63	0.8743572	6350
REGULATION OF WOUND HEALING%GOBP%GO:0061041	0.34512678	1.1846288	99	0.8743746	3153
TRICARBOXYLIC ACID CYCLE%GOBP%GO:0006099	0.39519614	1.2172897	28	0.87437826	3899
SIGNALING BY NOTCH1 IN CANCER%REACTOME%R-HSA-2644603.2	0.3863104	1.2684237	56	0.87439907	8274
REGULATION OF STRESS FIBER ASSEMBLY%GOBP%GO:0051492	0.37927014	1.2691735	70	0.874403	6955
TOLL RECEPTOR SIGNALING PATHWAY%PANTHER PATHWAY%P00054	0.4094944	1.3162243	42	0.87442625	10539
BILE ACID AND BILE SALT TRANSPORT%GOBP%GO:0015721	0.42699128	1.1839768	16	0.8744269	6668
REGULATION OF ESTABLISHMENT OF PROTEIN LOCALIZATION TO MITOCHONDRION%GOBP%GO:1903747	0.35561568	1.1815025	64	0.87447166	7957
FCGAMMA RECEPTOR (FCGR) DEPENDENT PHAGOCYTOSIS%REACTOME%R-HSA-2029480.1	0.34663463	1.183433	75	0.874542	6350
PYRIMIDINE-CONTAINING COMPOUND METABOLIC PROCESS%GOBP%GO:0072527	0.3798734	1.2678225	61	0.87455076	4743
ROLE OF PHOSPHOLIPIDS IN PHAGOCYTOSIS%REACTOME DATABASE ID RELEASE 75%2029485	0.41651982	1.1848439	20	0.874579	5168
GLYCOLYTIC PROCESS THROUGH FRUCTOSE-6-PHOSPHATE%GOBP%GO:0061615	0.42892766	1.2685628	25	0.8746025	4362
LPA RECEPTOR MEDIATED EVENTS%PATHWAY INTERACTION DATABASE NCI-NATURE CURATED DATA%LPA RECEPTOR MEDIATED EVENTS	0.3647091	1.2181364	59	0.8746126	3310
CITRATE METABOLIC PROCESS%GOBP%GO:0006101	0.3841446	1.1806965	29	0.87464565	3899
174178.3	0.34845197	1.1648244	72	0.87464815	8898
POSITIVE REGULATION OF T CELL MIGRATION%GOBP%GO:2000406	0.44928733	1.273811	18	0.8746752	6299
REGULATION OF POTASSIUM ION TRANSMEMBRANE TRANSPORTER ACTIVITY%GOBP%GO:1901016	0.36957487	1.1840446	41	0.874717	4230
MITOTIC METAPHASE PLATE CONGRESSION%GOBP%GO:0007080	0.36308008	1.1830044	51	0.87474304	12942
TRANSCRIPTION ELONGATION FROM RNA POLYMERASE I PROMOTER%GOBP%GO:0006362	0.43960696	1.3166436	25	0.8747572	6737
TRIF-DEPENDENT TOLL-LIKE RECEPTOR SIGNALING PATHWAY%GOBP%GO:0035666	0.4291359	1.3154434	28	0.8747668	4048
POSITIVE REGULATION OF ACTIN FILAMENT POLYMERIZATION%GOBP%GO:0030838	0.35427058	1.1831266	64	0.8747684	6264
PID_ERBB2_ERBB3_PATHWAY%MSIGDB_C2%PID_ERBB2_ERBB3_PATHWAY	0.37870485	1.1815683	38	0.87476933	10539
HALLMARK_HYPOXIA%MSIGDB_C2%HALLMARK_HYPOXIA	0.33224615	1.1658772	139	0.8747905	5362
TRANSCRIPTION INITIATION FROM RNA POLYMERASE II PROMOTER%GOBP%GO:0006367	0.35998926	1.2715101	148	0.87481314	4828
NUCLEOSIDE MONOPHOSPHATE METABOLIC PROCESS%GOBP%GO:0009123	0.33358553	1.1850481	196	0.8748286	6762
ACTIVATION OF KAINATE RECEPTORS UPON GLUTAMATE BINDING%REACTOME%R-HSA-451326.2	0.38268942	1.1667314	29	0.87483007	9314
GOLGI TO VACUOLE TRANSPORT%GOBP%GO:0006896	0.4005924	1.1643136	19	0.8748313	9131
REGULATION OF T CELL MEDIATED IMMUNITY%GOBP%GO:0002709	0.36907953	1.1842767	43	0.8748483	4842
REGULATION OF EPIDERMAL GROWTH FACTOR RECEPTOR SIGNALING PATHWAY%GOBP%GO:0042058	0.3536903	1.1841394	74	0.87487257	9444
REGULATION OF CD4-POSITIVE, ALPHA-BETA T CELL ACTIVATION%GOBP%GO:2000514	0.4170137	1.3159086	36	0.8748752	1995
EVENTS	0.41463718	1.1843958	19	0.8748913	5207
RNA POLYMERASE II TRANSCRIPTION ELONGATION%REACTOME%R-HSA-75955.2	0.35413688	1.1807739	57	0.87489825	7447
SELECTIVE AUTOPHAGY%REACTOME DATABASE ID RELEASE 75%9663891	0.3525844	1.1672068	57	0.8749095	3142
NUCLEAR RECEPTORS%WIKIPATHWAYS_20210110%WP170%HOMO SAPIENS	0.3940488	1.16417	23	0.8749181	9672
ACTOMYOSIN STRUCTURE ORGANIZATION%GOBP%GO:0031032	0.35791346	1.2182171	79	0.87493324	8428
POSITIVE EPIGENETIC REGULATION OF RRNA EXPRESSION%REACTOME DATABASE ID RELEASE 75%5250913	0.385702	1.2713073	61	0.8749355	5533
WHITE FAT CELL DIFFERENTIATION%WIKIPATHWAYS_20210110%WP4149%HOMO SAPIENS	0.39503017	1.184643	26	0.8749392	9661
TRANSLATION FACTORS%WIKIPATHWAYS_20210110%WP107%HOMO SAPIENS	0.36639675	1.1673124	40	0.8749798	5010
CELLULAR PROCESS INVOLVED IN REPRODUCTION IN MULTICELLULAR ORGANISM%GOBP%GO:0022412	0.33531645	1.1648675	123	0.8750169	9321
PID_MET_PATHWAY%MSIGDB_C2%PID_MET_PATHWAY	0.36169994	1.1834635	59	0.8750333	4865
REGULATION OF ERBB SIGNALING PATHWAY%GOBP%GO:1901184	0.3769976	1.2707169	79	0.8750865	10507
ABERRANT REGULATION OF MITOTIC EXIT IN CANCER DUE TO RB1 DEFECTS%REACTOME DATABASE ID RELEASE 75%9687136	0.40381238	1.1659323	20	0.8751012	9837
EPIGENETIC REGULATION OF GENE EXPRESSION%REACTOME DATABASE ID RELEASE 75%212165	0.3437516	1.1820041	102	0.8751049	5533
REGULATION OF ACTIN FILAMENT LENGTH%GOBP%GO:0030832	0.36163768	1.2709057	138	0.8751168	6307
PID_MYC_PATHWAY%MSIGDB_C2%PID_MYC_PATHWAY	0.41085047	1.1646098	19	0.8751494	1365
PEPTIDYL-L-CYSTEINE S-PALMITOYLATION%GOBP%GO:0018230	0.39728522	1.1848553	23	0.8751508	5507
REGULATION OF MUSCLE CONTRACTION%GOBP%GO:0006937	0.33891714	1.1654295	98	0.8751652	10141
ENTRAINMENT OF CIRCADIAN CLOCK%GOBP%GO:0009649	0.37518862	1.1643603	30	0.87516946	2423
PROTEIN TARGETING TO MITOCHONDRION%GOBP%GO:0006626	0.36444792	1.1851015	53	0.87518436	290
NEGATIVE REGULATION OF PROTEIN DEPOLYMERIZATION%GOBP%GO:1901880	0.3768552	1.2183102	41	0.8751871	8104
NEGATIVE REGULATION OF ION TRANSMEMBRANE TRANSPORTER ACTIVITY%GOBP%GO:0032413	0.35938144	1.166885	48	0.87523526	4230
HALLMARK_OXIDATIVE_PHOSPHORYLATION%MSIGDB_C2%HALLMARK_OXIDATIVE_PHOSPHORYLATION	0.3601702	1.2686356	156	0.87525344	8900
FCERI MEDIATED MAPK ACTIVATION%REACTOME DATABASE ID RELEASE 75%2871796	0.399557	1.166257	22	0.87526256	11010
BIOCARTA_AGR_PATHWAY%MSIGDB_C2%BIOCARTA_AGR_PATHWAY	0.43070304	1.316789	23	0.87526435	10579
EMBRYONIC APPENDAGE MORPHOGENESIS%GOBP%GO:0035113	0.36674008	1.1656514	37	0.87528795	6311
POSITIVE REGULATION OF TRANSMEMBRANE TRANSPORT%GOBP%GO:0034764	0.33463344	1.1644558	130	0.8752925	4165
SIGNAL TRANSDUCTION BY L1%REACTOME%R-HSA-445144.1	0.41102085	1.1667477	19	0.87533236	10579
NANOPARTICLE TRIGGERED AUTOPHAGIC CELL DEATH%WIKIPATHWAYS_20210110%WP2509%HOMO SAPIENS	0.4054043	1.1652678	18	0.87534577	3467
CYCLIC-NUCLEOTIDE-MEDIATED SIGNALING%GOBP%GO:0019935	0.34231928	1.1836599	120	0.8753478	6707
PLATELET-DERIVED GROWTH FACTOR RECEPTOR SIGNALING PATHWAY%GOBP%GO:0048008	0.41637048	1.18157	17	0.8753817	6137
RNA POLYMERASE II TRANSCRIPTION INITIATION%REACTOME%R-HSA-75953.2	0.41652453	1.3304139	44	0.8753844	6737
TOLL-LIKE RECEPTOR SIGNALING PATHWAY%GOBP%GO:0002224	0.34301564	1.1669711	79	0.87540203	4048
CONDENSATION OF PROPHASE CHROMOSOMES%REACTOME%R-HSA-2299718.2	0.3802911	1.1807941	31	0.8754186	3166
SISTER CHROMATID SEGREGATION%GOBP%GO:0000819	0.33077228	1.1659857	126	0.87542546	12942
REGULATION OF LYMPHOCYTE ACTIVATION%GOBP%GO:0051249	0.3499082	1.262711	246	0.87542915	6584
ENDOGENOUS_CANNABINOID_SIGNALING%PANTHER PATHWAY%P05730	0.42736578	1.2691739	22	0.8754416	6094
BASE-EXCISION REPAIR, AP SITE FORMATION%REACTOME%R-HSA-73929.2	0.37088537	1.1648918	33	0.8754642	3166
RNA CAPPING%GOBP%GO:0036260	0.3804613	1.1834998	34	0.87548447	6737
DNA REPAIR PATHWAYS FULL NETWORK%WIKIPATHWAYS_20210110%WP4946%HOMO SAPIENS	0.34716132	1.1827171	94	0.8754853	4837
REGULATION OF MUSCLE SYSTEM PROCESS%GOBP%GO:0090257	0.33831316	1.181677	129	0.87550205	10141
RIBONUCLEOTIDE CATABOLIC PROCESS%GOBP%GO:0009261	0.4227004	1.2632244	23	0.8755045	662
POSITIVE REGULATION OF TYPE I INTERFERON PRODUCTION%GOBP%GO:0032481	0.3484243	1.1633232	63	0.87552494	7658
REGULATION OF CARDIAC MUSCLE CELL MEMBRANE REPOLARIZATION%GOBP%GO:0099623	0.40180817	1.1663194	20	0.87553096	5620
PROTEIN UBIQUITINATION%REACTOME DATABASE ID RELEASE 75%8852135	0.35537416	1.1654679	70	0.8755477	3218
PROTEIN LOCALIZATION TO MICROTUBULE ORGANIZING CENTER%GOBP%GO:1905508	0.4155682	1.1820385	19	0.8755532	8985
ANTIGEN PROCESSING AND PRESENTATION OF ENDOGENOUS PEPTIDE ANTIGEN%GOBP%GO:0002483	0.4176651	1.1851536	17	0.87555605	4842
UNBLOCKING OF NMDA RECEPTORS, GLUTAMATE BINDING AND ACTIVATION%REACTOME DATABASE ID RELEASE 75%438066	0.4527929	1.3162707	20	0.8755668	5700
REGULATION OF G2/M TRANSITION OF MITOTIC CELL CYCLE%GOBP%GO:0010389	0.32820737	1.1649791	179	0.87563795	9743
INNATE IMMUNE RESPONSE-ACTIVATING SIGNAL TRANSDUCTION%GOBP%GO:0002758	0.32717472	1.1634132	183	0.8756772	5609
TISSUE REMODELING%GOBP%GO:0048771	0.37221223	1.1635333	36	0.87568533	6322
REGULATION OF AUTOPHAGOSOME ASSEMBLY%GOBP%GO:2000785	0.38159952	1.1817628	34	0.8756864	8876
GABA B RECEPTOR ACTIVATION%REACTOME%R-HSA-977444.2	0.40815222	1.3169487	42	0.87570465	6719
NEGATIVE REGULATION OF ACTIN FILAMENT DEPOLYMERIZATION%GOBP%GO:0030835	0.4413686	1.2709838	20	0.8757348	7948
REGULATION OF DNA BINDING%GOBP%GO:0051101	0.37267947	1.2692969	82	0.8757549	7812
NOTCH SIGNALING PATHWAY NETPATH%WIKIPATHWAYS_20210110%WP61%HOMO SAPIENS	0.36255398	1.1636316	43	0.8757873	6496
REGULATION OF ACTIN FILAMENT POLYMERIZATION%GOBP%GO:0030833	0.35353205	1.2168515	123	0.8757973	6307
SPERMATOGENESIS%GOBP%GO:0007283	0.33364442	1.1821183	169	0.87580323	5296
HIV INFECTION%REACTOME%R-HSA-162906.1	0.33283114	1.1928576	218	0.8758206	8326
REGULATION OF FIBROBLAST GROWTH FACTOR RECEPTOR SIGNALING PATHWAY%GOBP%GO:0040036	0.39826983	1.1660136	20	0.87586164	10558
VALIDATED TRANSCRIPTIONAL TARGETS OF AP1 FAMILY MEMBERS FRA1 AND FRA2%PATHWAY INTERACTION DATABASE NCI-NATURE CURATED DATA%VALIDATED TRANSCRIPTIONAL TARGETS OF AP1 FAMILY MEMBERS FRA1 AND FRA2	0.42428523	1.330593	33	0.875865	5922
INTRINSIC APOPTOTIC SIGNALING PATHWAY BY P53 CLASS MEDIATOR%GOBP%GO:0072332	0.3867314	1.2183208	36	0.87589616	6397
REGULATION OF SUPEROXIDE METABOLIC PROCESS%GOBP%GO:0090322	0.41693953	1.1663624	16	0.8759068	6318
PID_HIV_NEF_PATHWAY%MSIGDB_C2%PID_HIV_NEF_PATHWAY	0.39104337	1.165034	24	0.8759446	4417
REGULATION OF CALCIUM-MEDIATED SIGNALING%GOBP%GO:0050848	0.34850138	1.1637009	63	0.87599677	10012
RESPIRATORY ELECTRON TRANSPORT CHAIN%GOBP%GO:0022904	0.3563509	1.216662	92	0.87604535	4191
CELLULAR RESPONSE TO GLUCOSE STARVATION%GOBP%GO:0042149	0.37280777	1.1926765	43	0.87606955	8876
REGULATION OF INFLAMMATORY RESPONSE%GOBP%GO:0050727	0.33247647	1.1851687	221	0.87610096	5213
PROTEIN ACTIVATION CASCADE%GOBP%GO:0072376	0.41922978	1.3154517	36	0.87613684	6471
BIOCARTA_TCR_PATHWAY%MSIGDB_C2%BIOCARTA_TCR_PATHWAY	0.40827447	1.2694198	30	0.87613887	5787
CELL KILLING%GOBP%GO:0001906	0.40540135	1.263459	32	0.87616897	4001
GABA RECEPTOR SIGNALING%WIKIPATHWAYS_20210110%WP4159%HOMO SAPIENS	0.41758916	1.2629266	26	0.8762285	3869
CELLULAR RESPONSE TO UV%GOBP%GO:0034644	0.3758608	1.2632698	79	0.876242	4807
NEGATIVE REGULATION OF ANOIKIS%GOBP%GO:2000811	0.4417338	1.2164775	15	0.8762639	4020
NEGATIVE REGULATORS OF RIG-I MDA5 SIGNALING%REACTOME%R-HSA-936440.4	0.371801	1.1637639	34	0.8762713	2983
IMPORT INTO NUCLEUS%GOBP%GO:0051170	0.35554343	1.218395	87	0.8762767	5367
SIGNALING BY NOTCH1 PEST DOMAIN MUTANTS IN CANCER%REACTOME%R-HSA-2644602.1	0.3863104	1.2627392	56	0.87629455	8274
MUSCLE CONTRACTION%GOBP%GO:0006936	0.3469346	1.2158698	159	0.8762979	5758
PLASMA LIPOPROTEIN PARTICLE CLEARANCE%GOBP%GO:0034381	0.38735518	1.192886	30	0.8763426	8637
DEADENYLATION-DEPENDENT MRNA DECAY%REACTOME%R-HSA-429914.2	0.37216878	1.2228565	56	0.8763494	3452
NEUTROPHIL MIGRATION%GOBP%GO:1990266	0.41141108	1.3170782	43	0.8763584	5609

CELL-CELL SIGNALING BY WNT%GOBP%GO:0198738	0.33253074	1.1821268	183	0.87638426	8874
APC C:CDC20 MEDIATED DEGRADATION OF CYCLIN B%REACTOME DATABASE ID RELEASE 75%174048	0.4269934	1.2697335	24	0.8764211	9837
REGULATION OF APOPTOSIS%REACTOME DATABASE ID RELEASE 75%169911	0.37006673	1.2185043	51	0.8764456	8770
REGULATION OF RESPONSE TO EXTRACELLULAR STIMULUS%GOBP%GO:0032104	0.4349836	1.1852181	15	0.87648934	6910
NEGATIVE REGULATION OF SUPRAMOLECULAR FIBER ORGANIZATION%GOBP%GO:1902904	0.34096304	1.1822286	116	0.87654227	6307
ENDOMETRIAL CANCER%WIKIPATHWAYS_20210110%WP4155%HOMO SAPIENS	0.37118953	1.2161232	53	0.8765491	4363
SIGNALING OF HEPATOCYTE GROWTH FACTOR RECEPTOR%WIKIPATHWAYS_20210110%WP313%HOMO SAPIENS	0.38346604	1.1853254	28	0.8765759	4202
REGULATION OF ENDOTHELIAL CELL DEVELOPMENT%GOBP%GO:1901550	0.47664672	1.3314885	16	0.8765996	2901
POSITIVE REGULATION OF ION TRANSMEMBRANE TRANSPORT%GOBP%GO:0034767	0.3530016	1.2159654	98	0.87661004	4165
RESPONSE TO PURINE-CONTAINING COMPOUND%GOBP%GO:0014074	0.36852714	1.1937802	52	0.8766236	2371
REGULATION OF CELL CYCLE G2/M PHASE TRANSITION%GOBP%GO:1902749	0.3406138	1.2153589	195	0.87662625	9743
SIGNALING BY ERYTHROPOIETIN%REACTOME%R-HSA-9006335.2	0.42285633	1.2226416	20	0.8766385	5320
MMR%REACTOME%R-HSA-5358508.1	0.43536925	1.1859665	15	0.87666273	4015
DEPYRIMIDINATION%REACTOME DATABASE ID RELEASE 75%73928	0.38022873	1.1934962	32	0.87667984	3166
N-TERMINAL PROTEIN AMINO ACID MODIFICATION%GOBP%GO:0031365	0.41569224	1.2190456	24	0.87674606	1352
CELL ADHESION MEDIATED BY INTEGRIN%GOBP%GO:0033627	0.45962477	1.3322386	21	0.8767596	7674
HALLMARK_XENOBIOTIC_METABOLISM%MSIGDB_C2%HALLMARK_XENOBIOTIC_METABOLISM	0.3640809	1.2694839	124	0.8767745	4387
IL-18 SIGNALING PATHWAY%WIKIPATHWAYS_20210110%WP4754%HOMO SAPIENS	0.3333049	1.192916	177	0.87683934	8580
POSITIVE REGULATION OF CELL KILLING%GOBP%GO:0031343	0.4084609	1.1861852	21	0.8768591	3983
BCR SIGNALING PATHWAY%PATHWAY INTERACTION DATABASE NCI-NATURE CURATED DATA%BCR SIGNALING PATHWAY	0.36469457	1.1865903	55	0.876873	4266
REGULATION OF VACUOLE ORGANIZATION%GOBP%GO:0044088	0.38999844	1.2701987	43	0.87687784	4124
PURINE RIBONUCLEOSIDE TRIPHOSPHATE METABOLIC PROCESS%GOBP%GO:0009205	0.34782326	1.2229085	180	0.8768948	6762
FERTILIZATION%GOBP%GO:0009566	0.37093905	1.2154527	56	0.8769068	5543
REGULATION OF CELL SIZE%GOBP%GO:0008361	0.33503577	1.1627688	105	0.87690693	2671
HALLMARK_IL2_STAT5_SIGNALING%MSIGDB_C2%HALLMARK_IL2_STAT5_SIGNALING	0.34996802	1.2235156	141	0.8769138	7033
COPII-MEDIATED VESICLE TRANSPORT%REACTOME%R-HSA-204005.4	0.36243543	1.1942586	67	0.876932	8320
PROTEIN LOCALIZATION TO CYTOSKELETON%GOBP%GO:0044380	0.38730577	1.2155901	34	0.8769497	8985
RNA POLYMERASE I PROMOTER ESCAPE%REACTOME%R-HSA-73772.5	0.39298308	1.2698269	47	0.8769501	3876
REGULATION OF NEURON APOPTOTIC PROCESS%GOBP%GO:0043523	0.34638786	1.1939784	99	0.87695575	7591
POSITIVE REGULATION OF LAMELLIPODIUM ORGANIZATION%GOBP%GO:1902745	0.3949549	1.2161841	30	0.87699175	6946
PHOSPHATIDYLINOSITOL 3-KINASE SIGNALING%GOBP%GO:0014065	0.3980613	1.2224215	28	0.8770135	5287
MRNA 3'-END PROCESSING%REACTOME%R-HSA-72187.4	0.36159065	1.1822594	56	0.877018	3825
RESPONSE TO ELEVATED PLATELET CYTOSOLIC CA2+%REACTOME%R-HSA-76005.2	0.35126898	1.2186856	112	0.8770482	7087
CELLULAR RESPONSE TO OSMOTIC STRESS%GOBP%GO:0071470	0.40514326	1.193279	21	0.8770642	6544
ORGANELLE DISASSEMBLY%GOBP%GO:1903008	0.36690378	1.2191337	69	0.877076	6862
PEPTIDYL-THREONINE MODIFICATION%GOBP%GO:0018210	0.35518736	1.1938204	70	0.8770768	7719
HALLMARK_MITOTIC_SPINDLE%MSIGDB_C2%HALLMARK_MITOTIC_SPINDLE	0.339171	1.1862705	144	0.8770989	8284
MALE SEX DIFFERENTIATION%GOBP%GO:0046661	0.36849076	1.1860093	40	0.8771123	8945
DNA DAMAGE CHECKPOINT%GOBP%GO:0000077	0.34057996	1.1853356	121	0.87715644	6145
POLYSACCHARIDE BIOSYNTHETIC PROCESS%GOBP%GO:0000271	0.42561582	1.331139	35	0.8771644	4449
PID_TCR_CALCIUM_PATHWAY%MSIGDB_C2%PID_TCR_CALCIUM_PATHWAY	0.480333	1.3306407	15	0.8771706	10501
FORMATION OF TC-NER PRE-INCISION COMPLEX%REACTOME%R-HSA-6781823.1	0.40448645	1.3337497	53	0.87717175	8457
BASE-EXCISION REPAIR%GOBP%GO:0006284	0.406602	1.2634599	33	0.87717265	1723
7Q11.23 COPY NUMBER VARIATION SYNDROME%WIKIPATHWAYS_20210110%WP4932%HOMO SAPIENS	0.37324995	1.2622172	66	0.87719846	8792
REGULATION OF DNA-TEMPLATED TRANSCRIPTION IN RESPONSE TO STRESS%GOBP%GO:0043620	0.35255876	1.2185063	105	0.8771988	6903
PYRUVATE METABOLIC PROCESS%GOBP%GO:0006090	0.3656038	1.1922983	52	0.8772114	4362
MAINTENANCE OF LOCATION IN CELL%GOBP%GO:0051651	0.3752674	1.2636284	71	0.87722325	5399
MORPHOGENESIS OF AN EPITHELIUM%GOBP%GO:0002009	0.33617252	1.1940571	195	0.8772546	7352
WNT SIGNALING PATHWAY%GOBP%GO:0016055	0.3368486	1.1935121	181	0.87727505	8874
POSTTRANSCRIPTIONAL GENE SILENCING BY RNA%GOBP%GO:0035194	0.3827196	1.2637924	54	0.8773353	7405
ALPHA-BETA T CELL ACTIVATION%GOBP%GO:0046631	0.39682788	1.192951	25	0.8773398	2681
MITOTIC CELL CYCLE CHECKPOINT%GOBP%GO:0007093	0.32902354	1.162793	131	0.8773511	5039
G1 PHASE%REACTOME DATABASE ID RELEASE 75%69236	0.38176784	1.2229748	44	0.8773665	6377
MULTICELLULAR ORGANISMAL HOMEOSTASIS%GOBP%GO:0048871	0.33396482	1.18542	161	0.87738353	5415
POSITIVE REGULATION OF SIGNALING RECEPTOR ACTIVITY%GOBP%GO:2000273	0.3893073	1.1863457	28	0.8773899	7042
ANGIOGENESIS%PANTHER PATHWAY%P00005	0.3763047	1.3323905	153	0.87740254	4607
WNT LIGAND BIOGENESIS AND TRAFFICKING%REACTOME DATABASE ID RELEASE 75%3238698	0.42891085	1.2614366	22	0.8774139	7542
POSITIVE REGULATION OF LEUKOCYTE MEDIATED CYTOTOXICITY%GOBP%GO:0001912	0.4084609	1.2192166	21	0.8774149	3983
POSITIVE REGULATION OF ENDOTHELIAL CELL MIGRATION%GOBP%GO:0010595	0.3596238	1.2235774	89	0.877419	8377
POSITIVE REGULATION OF ALPHA-BETA T CELL ACTIVATION%GOBP%GO:0046635	0.3859095	1.1866052	33	0.87743723	3567
REGULATION OF MITOTIC SPINDLE ASSEMBLY%GOBP%GO:1901673	0.4266254	1.2232454	20	0.8775505	8514
NO/CGMP/PKG MEDIATED NEUROPROTECTION%WIKIPATHWAYS_20210110%WP4008%HOMO SAPIENS	0.38763648	1.1942675	28	0.8775619	3663
INOSITOL PHOSPHATE METABOLISM%REACTOME%R-HSA-1483249.3	0.37642106	1.2193289	48	0.8775969	7173
CLEAVAGE OF THE DAMAGED PURINE%REACTOME DATABASE ID RELEASE 75%110331	0.38191703	1.1623694	27	0.87760144	5210
POSITIVE REGULATION OF CELLULAR RESPONSE TO TRANSFORMING GROWTH FACTOR BETA STIMULUS%GOBP%GO:1903846	0.39014193	1.1624886	22	0.8776028	7534
CARBOHYDRATE CATABOLIC PROCESS%GOBP%GO:0016052	0.3723512	1.2698982	84	0.8776075	7952
HEDGEHOG%NETPATH%HEDGEHOG	0.43978006	1.3368185	28	0.8776537	4911
MITOTIC CYTOKINESIS%GOBP%GO:0000281	0.35702193	1.193018	61	0.8776789	7013
SPHINGOLIPID METABOLIC PROCESS%GOBP%GO:0006665	0.36015764	1.2619411	133	0.87769467	5803
REGULATION OF SPINDLE ORGANIZATION%GOBP%GO:0090224	0.37654787	1.1866776	38	0.8777102	8514
EVENTS	0.44955006	1.3307955	23	0.87774605	5442
PID_SMAD2_3NUCLEAR_PATHWAY%MSIGDB_C2%PID_SMAD2_3NUCLEAR_PATHWAY	0.377932	1.2604558	63	0.8777668	5951
POSITIVE REGULATION OF PEPTIDASE ACTIVITY%GOBP%GO:0010952	0.337336	1.191902	146	0.8777713	6298
FC EPSILON RECEPTOR (FCERI) SIGNALING%REACTOME DATABASE ID RELEASE 75%2454202	0.35361218	1.2140728	114	0.8777816	8274
POSITIVE REGULATION OF BINDING%GOBP%GO:0051099	0.34437826	1.2186917	121	0.87778574	3562
REGULATION OF RESPONSE TO INTERFERON-GAMMA%GOBP%GO:0060330	0.41644862	1.192017	18	0.87791353	1297
CILIARY LANDSCAPE%WIKIPATHWAYS_20210110%WP4352%HOMO SAPIENS	0.33498982	1.1854422	159	0.8779194	3784
POSITIVE REGULATION OF EPITHELIAL CELL APOPTOTIC PROCESS%GOBP%GO:1904037	0.44841456	1.2615201	17	0.8779358	1034
APC C:CDC20 MEDIATED DEGRADATION OF SECURIN%REACTOME%R-HSA-174154.2	0.36778396	1.2230064	66	0.87798697	8898
IGF1 PATHWAY%PATHWAY INTERACTION DATABASE NCI-NATURE CURATED DATA%IGF1 PATHWAY	0.38488486	1.1855581	30	0.87801284	4378
ER TO GOLGI VESICLE-MEDIATED TRANSPORT%GOBP%GO:0006888	0.34273618	1.2146244	180	0.8780536	8520
CELL DEATH SIGNALLING VIA NRAGE, NRIF AND NADE%REACTOME DATABASE ID RELEASE 75%204998	0.39507678	1.3315064	72	0.8780645	5462
DNA DAMAGE RESPONSE, SIGNAL TRANSDUCTION BY P53 CLASS MEDIATOR%GOBP%GO:0030330	0.39602467	1.3333243	73	0.8780738	6189
DOWNSTREAM SIGNALING OF ACTIVATED FGFR4%REACTOME DATABASE ID RELEASE 75%5654716	0.4277553	1.2138647	17	0.87810314	5602
SIGNALING BY NOTCH%REACTOME%R-HSA-157118.5	0.33550915	1.1871157	187	0.87816316	9483
NEGATIVE REGULATION OF THE PI3K AKT NETWORK%REACTOME DATABASE ID RELEASE 75%199418	0.3538651	1.1944178	83	0.8781636	8404
ENTRAINMENT OF CIRCADIAN CLOCK BY PHOTOPERIOD%GOBP%GO:0043153	0.39670745	1.2235824	28	0.87818986	2423
FORMATION OF FIBRIN CLOT (CLOTTING CASCADE)%REACTOME DATABASE ID RELEASE 75%140877	0.41072482	1.2193598	22	0.8782054	6471
MODIFICATION OF MORPHOLOGY OR PHYSIOLOGY OF OTHER ORGANISM INVOLVED IN SYMBIOTIC INTERACTION%GOBP%GO:0051817	0.35209164	1.194273	77	0.87820566	5991
NEGATIVE REGULATION OF BLOOD CIRCULATION%GOBP%GO:1903523	0.5459655	1.5322757	17	0.87826407	5102
CA2+ PATHWAY%REACTOME%R-HSA-4086398.3	0.37163946	1.2197915	54	0.8782701	6373
DNA MISMATCH REPAIR%WIKIPATHWAYS_20210110%WP531%HOMO SAPIENS	0.42464823	1.1866874	17	0.8783176	3931
TRANSCRIPTION-COUPLED NUCLEOTIDE-EXCISION REPAIR%GOBP%GO:0006283	0.36836007	1.260545	73	0.8783195	8457
POLYOL METABOLIC PROCESS%GOBP%GO:0019751	0.3555013	1.2194848	89	0.8783238	7173
POSITIVE REGULATION OF LYMPHOCYTE MIGRATION%GOBP%GO:2000403	0.43207085	1.263794	21	0.8783402	5015
REGULATION OF PROTEIN POLYMERIZATION%GOBP%GO:0032271	0.34580263	1.2237135	172	0.8783497	6350
SIGNALING EVENTS	0.37786007	1.1945132	39	0.87838715	4995
NEGATIVE REGULATION OF CELLULAR CATABOLIC PROCESS%GOBP%GO:0031330	0.33498392	1.1946288	213	0.8785024	5005
REGULATION OF ALPHA-BETA T CELL ACTIVATION%GOBP%GO:0046634	0.37284604	1.2140757	51	0.87851715	2681
REGULATION OF MITOTIC SPINDLE ORGANIZATION%GOBP%GO:0060236	0.381581	1.196811	35	0.8786024	8514
VALIDATED TARGETS OF C-MYC TRANSCRIPTIONAL ACTIVATION%PATHWAY INTERACTION DATABASE NCI-NATURE CURATED DATA%VALIDATED TARGETS OF C-MYC TRANSCRIPTIONAL ACTIVATION	0.391241	1.3337816	75	0.8786122	6556
CONSTITUTIVE SIGNALING BY ABERRANT PI3K IN CANCER%REACTOME%R-HSA-2219530.1	0.40879545	1.3348049	49	0.8786816	8809
REGULATION OF MRNA 3'-END PROCESSING%GOBP%GO:0031440	0.44032636	1.3324393	26	0.878692	7481
DNA-TEMPLATED TRANSCRIPTION, ELONGATION%GOBP%GO:0006354	0.36214024	1.2217977	76	0.8787012	7669
LYMPHOCYTE PROLIFERATION%GOBP%GO:0046651	0.47829148	1.3369168	17	0.87872666	6569
POSITIVE REGULATION OF GTPASE ACTIVITY%GOBP%GO:0043547	0.33368123	1.187129	179	0.8787408	8880
CELL CYCLE CHECKPOINT%GOBP%GO:0000075	0.34230217	1.2198473	166	0.8787601	5039
NEGATIVE REGULATION OF PROTEOLYSIS INVOLVED IN CELLULAR PROTEIN CATABOLIC PROCESS%GOBP%GO:1903051	0.36142045	1.2146325	60	0.8787668	5005
HYALURONAN METABOLIC PROCESS%GOBP%GO:0030212	0.44401485	1.2606317	19	0.87881905	543
PID_HIF2PATHWAY%MSIGDB_C2%PID_HIF2PATHWAY	0.4261388	1.2600747	23	0.8788295	9790
SPINDLE CHECKPOINT%GOBP%GO:0031577	0.44953385	1.3316327	22	0.8788758	8983
MEMORY%GOBP%GO:0007613	0.38645846	1.2615298	54	0.8788798	6250
RECIPROCAL MEIOTIC RECOMBINATION%GOBP%GO:0007131	0.37547177	1.186705	39	0.8788839	12396
NEGATIVE REGULATION OF EPIDERMAL GROWTH FACTOR RECEPTOR SIGNALING PATHWAY%GOBP%GO:0042059	0.37595463	1.2237597	41	0.8788951	7831
BIOCARTA_GLEEVEC_PATHWAY%MSIGDB_C2%BIOCARTA_GLEEVEC_PATHWAY	0.4161996	1.1946757	19	0.8789316	4020
NICOTINAMIDE SALVAGING%REACTOME DATABASE ID RELEASE 75%197264	0.4180642	1.1868248	16	0.8789431	6480
FLAGELLATED SPERM MOTILITY%GOBP%GO:0030317	0.42057973	1.3342332	39	0.878973	6473
KERATINIZATION%REACTOME%R-HSA-6805567.2	0.38964275	1.2609724	46	0.8789749	5532
POSITIVE REGULATION OF T CELL PROLIFERATION%GOBP%GO:0042102	0.36249053	1.1947919	53	0.8790082	6556
NEGATIVE REGULATION OF PROTEOLYSIS%GOBP%GO:0045861	0.3357091	1.1872073	182	0.87901217	5005
REGULATION OF BLOOD CIRCULATION%GOBP%GO:1903522	0.3727823	1.3329091	196	0.8790139	7229
REGULATION OF MITOTIC NUCLEAR DIVISION%GOBP%GO:0007088	0.35543853	1.263853	143	0.87903756	9847
REGULATION OF PEPTIDYL-SERINE PHOSPHORYLATION%GOBP%GO:0033135	0.3564036	1.2194859	91	0.8790909	8404
POSITIVE REGULATION OF MONOOXYGENASE ACTIVITY%GOBP%GO:0032770	0.4271746	1.2141018	18	0.87912226	8196
ACTIVATION OF APC C AND APC C:CDC20 MEDIATED DEGRADATION OF MITOTIC PROTEINS%REACTOME DATABASE ID RELEASE 75%176814	0.35045758	1.1951711	75	0.87915516	9837
PURINE RIBONUCLEOSIDE MONOPHOSPHATE METABOLIC PROCESS%GOBP%GO:0009167	0.33387554	1.1972604	189	0.87916964	6762

PROTEIN IMPORT INTO NUCLEUS%GOBP%GO:0006606	0.35492125	1.1968296	68	0.8791924	5367
CONSTITUTIVE SIGNALING BY NOTCH1 HD+PEST DOMAIN MUTANTS%REACTOME DATABASE ID RELEASE 75%2894862	0.3863104	1.2645547	56	0.87921244	8274
PURINE NUCLEOSIDE MONOPHOSPHATE METABOLIC PROCESS%GOBP%GO:0009126	0.33387554	1.1950215	189	0.87921935	6762
22Q11.2 COPY NUMBER VARIATION SYNDROME%WIKIPATHWAYS_20210110%WP4657%HOMO SAPIENS	0.39421323	1.339214	84	0.8793066	8729
PID_ARF6_TRAFFICKING_PATHWAY%MSIGDB_C2%PID_ARF6_TRAFFICKING_PATHWAY	0.3849067	1.1948651	30	0.87931657	2699
DEATH RECEPTOR SIGNALLING%REACTOME%R-HSA-73887.4	0.34615254	1.2198912	133	0.8793208	6568
BIOCARTA_MAL_PATHWAY%MSIGDB_C2%BIOCARTA_MAL_PATHWAY	0.4421326	1.2130268	15	0.8793733	10230
ENERGY RESERVE METABOLIC PROCESS%GOBP%GO:0006112	0.42371377	1.33259	33	0.8793942	7919
INACTIVATION OF APC C VIA DIRECT INHIBITION OF THE APC C COMPLEX%REACTOME DATABASE ID RELEASE 75%141430	0.41705662	1.2133191	21	0.87939984	9837
WNT SIGNALING PATHWAY, PLANAR CELL POLARITY PATHWAY%GOBP%GO:0060071	0.35736933	1.2141938	82	0.8794135	8248
BIOCARTA_CDMAC_PATHWAY%MSIGDB_C2%BIOCARTA_CDMAC_PATHWAY	0.44309527	1.2218091	15	0.8794168	9509
CARDIAC MUSCLE CELL DIFFERENTIATION%GOBP%GO:0055007	0.39211997	1.2146517	33	0.8794299	7365
PYRUVATE BIOSYNTHETIC PROCESS%GOBP%GO:0042866	0.38877508	1.1872556	32	0.87943035	4362
GLYCOSPHINGOLIPID BIOSYNTHETIC PROCESS%GOBP%GO:0006688	0.41450566	1.2607007	26	0.87944114	1313
TRANSCRIPTIONAL REGULATION BY RUNX3%REACTOME DATABASE ID RELEASE 75%8878159	0.34816524	1.1977401	94	0.8794795	8770
ARF6 SIGNALING EVENTS%PATHWAY INTERACTION DATABASE NCI-NATURE CURATED DATA%ARF6 SIGNALING EVENTS	0.40257165	1.2131517	30	0.87948257	4816
POSITIVE REGULATION OF PROTEIN LOCALIZATION TO NUCLEUS%GOBP%GO:1900182	0.37333977	1.2201524	63	0.879487	8659
RNA PHOSPHODIESTER BOND HYDROLYSIS, ENDONUCLEOLYTIC%GOBP%GO:0090502	0.42004308	1.2595782	26	0.87950206	875
POSITIVE REGULATION OF PROTEASOMAL UBIQUITIN-DEPENDENT PROTEIN CATABOLIC PROCESS%GOBP%GO:0032436	0.350686	1.1956446	75	0.87951833	5766
SPERMATID DEVELOPMENT%GOBP%GO:0007286	0.3649544	1.1957853	56	0.8795582	5296
SPHINGOLIPID METABOLISM%REACTOME%R-HSA-428157.4	0.37124014	1.2639456	81	0.8795593	7337
BIOCARTA_VEGF_PATHWAY%MSIGDB_C2%BIOCARTA_VEGF_PATHWAY	0.42046538	1.2211853	21	0.87959576	4773
IL12 SIGNALING MEDIATED BY STAT4%PATHWAY INTERACTION DATABASE NCI-NATURE CURATED DATA%IL12 SIGNALING MEDIATED BY STAT4	0.46820617	1.3394462	19	0.87959653	4455
FC RECEPTOR SIGNALING PATHWAY%GOBP%GO:0038093	0.34038696	1.1968812	148	0.8796389	6350
CLEAVAGE OF THE DAMAGED PYRIMIDINE%REACTOME DATABASE ID RELEASE 75%110329	0.38022873	1.1972983	32	0.8796626	3166
RESPONSE TO MONOSACCHARIDE%GOBP%GO:0034284	0.3748803	1.2237642	52	0.87967104	11443
PLATELET DEGRANULATION%REACTOME DATABASE ID RELEASE 75%114608	0.3518607	1.2134098	107	0.87967634	7087
GLUCOSE METABOLISM%REACTOME%R-HSA-70326.8	0.3925955	1.3370254	78	0.879698	5123
PROTEIN	0.41350913	1.3383532	57	0.8797061	4567
POSITIVE REGULATION OF MICROTUBULE POLYMERIZATION OR DEPOLYMERIZATION%GOBP%GO:0031112	0.39166164	1.1951979	29	0.8797077	10936
PROSTAGLANDIN METABOLIC PROCESS%GOBP%GO:0006693	0.43825758	1.3349074	32	0.87971056	5007
MEIOTIC SYNAPSIS%REACTOME%R-HSA-1221632.1	0.38208845	1.2214516	43	0.87977	5210
PROTEIN ADP-RIBOSYLATION%GOBP%GO:0006471	0.3899808	1.1978182	27	0.8797709	1893
CELLULAR CARBOHYDRATE BIOSYNTHETIC PROCESS%GOBP%GO:0034637	0.37478906	1.2202431	44	0.8798098	8804
ENDOCRINE PROCESS%GOBP%GO:0050886	0.39964268	1.197402	24	0.87983054	10116
CARDIAC MUSCLE CELL ACTION POTENTIAL%GOBP%GO:0086001	0.3793871	1.2199389	44	0.8798596	7804
ACTIVATION OF NF-KAPPAB IN B CELLS%REACTOME%R-HSA-1169091.1	0.36102816	1.2039363	64	0.87988085	8770
POSITIVE REGULATION OF ORGAN GROWTH%GOBP%GO:0046622	0.43360654	1.2209718	18	0.87991226	5621
RIBONUCLEOSIDE MONOPHOSPHATE METABOLIC PROCESS%GOBP%GO:0009161	0.3281707	1.1617417	192	0.8799175	6762
REGULATION OF VIRAL LIFE CYCLE%GOBP%GO:1903900	0.34615985	1.2206682	136	0.8799234	4524
POSITIVE REGULATION OF MITOCHONDRIAL MEMBRANE PERMEABILITY%GOBP%GO:0035794	0.40800735	1.1614988	18	0.8799309	9027
MUSCLE STRUCTURE DEVELOPMENT%GOBP%GO:0061061	0.3320546	1.1958421	219	0.8799548	7804
REGULATION OF RYANODINE-SENSITIVE CALCIUM-RELEASE CHANNEL ACTIVITY%GOBP%GO:0060314	0.40525422	1.1959728	22	0.8799887	10635
REGULATION OF CYCLASE ACTIVITY%GOBP%GO:0031279	0.42020983	1.2593082	25	0.87999827	4171
REGULATION OF NEURON DEATH%GOBP%GO:1901214	0.33635148	1.1872705	163	0.88000935	6322
REGULATION OF DEFENSE RESPONSE TO VIRUS BY VIRUS%GOBP%GO:0050690	0.43655872	1.3338206	27	0.88001394	4674
MEDIATED SIGNALING	0.38038346	1.1606228	30	0.88007236	7004
CHOLESTEROL EFFLUX%GOBP%GO:0033344	0.4380657	1.2300673	17	0.8800732	8196
MODULATION BY HOST OF VIRAL PROCESS%GOBP%GO:0044788	0.43419892	1.2640353	22	0.8800932	5489
ANTERIOR/POSTERIOR PATTERN SPECIFICATION%GOBP%GO:0009952	0.35730246	1.1974807	74	0.8801035	10075
T-CELL RECEPTOR AND CO-STIMULATORY SIGNALING%WIKIPATHWAYS_20210110%WP2583%HOMO SAPIENS	0.4421959	1.195389	15	0.88010514	8748
ACTIVATION OF IRF3 IRF7 MEDIATED BY TBK1 IKK EPSILON%REACTOME%R-HSA-936964.2	0.42955983	1.1960862	16	0.8801086	8274
NEGATIVE REGULATION OF CHEMOTAXIS%GOBP%GO:0050922	0.37297386	1.1894072	42	0.8801199	8141
INTERACTION WITH HOST%GOBP%GO:0051701	0.35137856	1.1904578	79	0.88012534	7026
SIGNALING BY ERBB2 TMD JMD MUTANTS%REACTOME DATABASE ID RELEASE 75%9665686	0.4376535	1.2212313	17	0.880134	11130
NCRNA CATABOLIC PROCESS%GOBP%GO:0034661	0.38686022	1.1969157	30	0.8801455	2943
DUAL INCISION IN TC-NER%REACTOME%R-HSA-6782135.2	0.35731608	1.1892653	64	0.8801466	8457
BIOCARTA_RACCYCD_PATHWAY%MSIGDB_C2%BIOCARTA_RACCYCD_PATHWAY	0.41090375	1.220472	23	0.88015133	4489
REGULATION OF CELLULAR AMINO ACID METABOLIC PROCESS%GOBP%GO:0006521	0.3664851	1.1978794	55	0.8801647	8211
THE ROLE OF GTSE1 IN G2 M PROGRESSION AFTER G2 CHECKPOINT%REACTOME DATABASE ID RELEASE 75%8852276	0.34967595	1.1615653	58	0.880171	9483
OTHER INTERLEUKIN SIGNALING%REACTOME%R-HSA-449836.10	0.40427503	1.1952372	21	0.88017243	5087
GRANULOCYTE CHEMOTAXIS%GOBP%GO:0071621	0.41919404	1.3350766	41	0.8802037	5434
APOPTOTIC CELL CLEARANCE%GOBP%GO:0043277	0.40190005	1.2040117	24	0.8802091	8987
SCF-DEPENDENT PROTEASOMAL UBIQUITIN-DEPENDENT PROTEIN CATABOLIC PROCESS%GOBP%GO:0031146	0.34616843	1.1873548	86	0.8802278	8952
RESPONSE TO ALCOHOL%GOBP%GO:0097305	0.35584244	1.1912518	69	0.88025033	5993
LOCALIZATION WITHIN MEMBRANE%GOBP%GO:0051668	0.3394139	1.1607049	83	0.8802564	9405
RHO GTPASES ACTIVATE FORMINS%REACTOME%R-HSA-5663220.1	0.34263623	1.1895018	115	0.8802956	6033
POSITIVE REGULATION OF RESPONSE TO WOUNDING%GOBP%GO:1903036	0.46656552	1.5329567	48	0.8802966	1899
INTERFERON GAMMA SIGNALING%REACTOME%R-HSA-877300.3	0.35561332	1.2035682	74	0.8803007	11389
HOMOPHILIC CELL ADHESION VIA PLASMA MEMBRANE ADHESION MOLECULES%GOBP%GO:0007156	0.3777774	1.2301661	52	0.88035774	2398
NEURAL CREST CELL DIFFERENTIATION%GOBP%GO:0014033	0.37451112	1.1963105	51	0.8803599	8147
DIFFERENTIATION OF WHITE AND BROWN ADIPOCYTE %WIKIPATHWAYS_20210110%WP2895%HOMO SAPIENS	0.4233339	1.1876007	18	0.8803727	6416
KERATINOCYTES	0.4308031	1.2595973	20	0.8804113	1935
LEUKOCYTE CHEMOTAXIS%GOBP%GO:0030595	0.3554512	1.1896057	73	0.8804263	6935
REGULATION OF ACTIN FILAMENT ORGANIZATION%GOBP%GO:0110053	0.34311974	1.2237682	208	0.8804485	6307
MALE MEIOTIC NUCLEAR DIVISION%GOBP%GO:0007140	0.4255688	1.2036794	17	0.88045204	1417
STRIATED MUSCLE CELL DIFFERENTIATION%GOBP%GO:0051146	0.39213005	1.3342551	84	0.8804528	8428
MEIOSIS I CELL CYCLE PROCESS%GOBP%GO:0061982	0.36763725	1.2207156	64	0.8804751	1675
HEMATOPOIETIC STEM CELL DIFFERENTIATION%WIKIPATHWAYS_20210110%WP2849%HOMO SAPIENS	0.38524255	1.1901217	31	0.8804774	3131
DNA-TEMPLATED TRANSCRIPTION, INITIATION%GOBP%GO:0006352	0.3455361	1.2202656	177	0.8804797	7338
POSITIVE REGULATION OF DNA-BINDING TRANSCRIPTION FACTOR ACTIVITY%GOBP%GO:0051091	0.33230343	1.1902477	211	0.8805092	5015
REGULATION OF STEROL TRANSPORT%GOBP%GO:0032371	0.38473842	1.2125168	40	0.8805421	6142
DEOXYRIBOSE PHOSPHATE CATABOLIC PROCESS%GOBP%GO:0046386	0.42500365	1.198212	17	0.8805505	5891
REGULATION OF HEART CONTRACTION%GOBP%GO:0008016	0.37743145	1.3398283	177	0.8805702	7588
PID_P73PATHWAY%MSIGDB_C2%PID_P73PATHWAY	0.36013642	1.18876	58	0.88059324	3377
POSITIVE REGULATION BY HOST OF VIRAL TRANSCRIPTION%GOBP%GO:0043923	0.43855655	1.1904932	15	0.8806095	5648
NEUTROPHIL CHEMOTAXIS%GOBP%GO:0030593	0.42273673	1.3387376	38	0.8806257	5434
NEGATIVE REGULATION OF ACTIN FILAMENT POLYMERIZATION%GOBP%GO:0030837	0.37897748	1.1884893	37	0.8806257	6204
ACTIN FILAMENT-BASED MOVEMENT%GOBP%GO:0030048	0.35591936	1.2102759	93	0.88063264	7804
CELL SURFACE RECEPTOR SIGNALING PATHWAY INVOLVED IN CELL-CELL SIGNALING%GOBP%GO:1905114	0.33299166	1.1874006	205	0.88066965	9483
CORTICOTROPIN-RELEASING HORMONE SIGNALING PATHWAY%WIKIPATHWAYS_20210110%WP2355%HOMO SAPIENS	0.4036446	1.3379296	63	0.880677	4928
NEGATIVE REGULATION OF IMMUNE RESPONSE%GOBP%GO:0050777	0.3522809	1.1961043	78	0.88070244	6153
INTERACTIONS OF VPR WITH HOST CELLULAR PROTEINS%REACTOME DATABASE ID RELEASE 75%176033	0.3863727	1.2040421	34	0.8807631	4994
HALLMARK_BILE_ACID_METABOLISM%MSIGDB_C2%HALLMARK_BILE_ACID_METABOLISM	0.36212423	1.2238609	73	0.8807718	6972
TIGHT JUNCTION ASSEMBLY%GOBP%GO:0120192	0.36388597	1.1607144	42	0.8807757	8039
POSITIVE REGULATION OF LEUKOCYTE PROLIFERATION%GOBP%GO:0070665	0.35474887	1.1978937	79	0.8807775	5724
GLYCOSPHINGOLIPID METABOLIC PROCESS%GOBP%GO:0006687	0.40740797	1.3376364	57	0.88080305	5307
IL-6-TYPE CYTOKINE RECEPTOR LIGAND INTERACTIONS%REACTOME%R-HSA-6788467.3	0.43597922	1.1982923	15	0.8808293	5355
REGULATION OF TYROSINE PHOSPHORYLATION OF STAT PROTEIN%GOBP%GO:0042509	0.41545185	1.3370917	43	0.88087946	4466
NOTCH SIGNALING PATHWAY%PATHWAY INTERACTION DATABASE NCI-NATURE CURATED DATA%NOTCH SIGNALING PATHWAY	0.36840683	1.212591	56	0.8809025	6496
NETWORK	0.35814816	1.1876175	71	0.88092405	4770
TRANSCRIPTION-COUPLED NUCLEOTIDE EXCISION REPAIR (TC-NER)%REACTOME%R-HSA-6781827.1	0.34976134	1.1896312	77	0.880959	8457
3-PHOSPHOINOSITIDE BIOSYNTHESIS%HUMANCYC%PWY-6352	0.45332623	1.3373386	24	0.8809779	6089
MITOTIC G1/S TRANSITION CHECKPOINT%GOBP%GO:0044819	0.37783176	1.2640558	62	0.8809911	6040
PROTEIN STABILIZATION%GOBP%GO:0050821	0.33326608	1.1905514	165	0.8810096	6445
NUCLEOSIDE DIPHOSPHATE PHOSPHORYLATION%GOBP%GO:0006165	0.3863598	1.2045585	39	0.8810239	4362
COSTIMULATION BY THE CD28 FAMILY%REACTOME%R-HSA-388841.3	0.36967677	1.2044129	54	0.8810279	4455
SAPIENS	0.3694567	1.1463904	30	0.88104945	2748
3Q29 COPY NUMBER VARIATION SYNDROME%WIKIPATHWAYS_20210110%WP4906%HOMO SAPIENS	0.37660858	1.2104846	45	0.88105625	6322
ATP METABOLIC PROCESS%GOBP%GO:0046034	0.34032196	1.1907929	162	0.8811506	6762
EPIBOLY%GOBP%GO:0090504	0.45645833	1.339464	23	0.88116306	6802
REGULATION OF CHOLESTEROL TRANSPORT%GOBP%GO:0032374	0.38473842	1.2301698	40	0.8811695	6142
RNA POLYMERASE II PRE-TRANSCRIPTION EVENTS%REACTOME DATABASE ID RELEASE 75%674695	0.35775742	1.1979487	77	0.88117504	6737
T CELL DIFFERENTIATION%GOBP%GO:0030217	0.3659898	1.2106065	52	0.88117754	6299
REGULATION OF HISTONE H3-K4 METHYLATION%GOBP%GO:0051569	0.4189284	1.1887714	18	0.88119864	5917
POSITIVE REGULATION OF SMOOTHENED SIGNALING PATHWAY%GOBP%GO:0045880	0.40105465	1.1607401	20	0.8812125	3284
RNA POLYMERASE I TRANSCRIPTION%REACTOME DATABASE ID RELEASE 75%73864	0.3686479	1.2239261	67	0.88121927	3876
SIGNALING BY FGFR IN DISEASE%REACTOME DATABASE ID RELEASE 75%1226099	0.36779684	1.1885014	51	0.8812219	7425
CHROMOSOME SEPARATION%GOBP%GO:0051304	0.40286124	1.1906416	23	0.8812369	11185
REGULATION OF CELL KILLING%GOBP%GO:0031341	0.42278135	1.3383715	39	0.8812475	4001
NEGATIVE REGULATION OF CD4-POSITIVE, ALPHA-BETA T CELL ACTIVATION%GOBP%GO:2000515	0.42377156	1.1983414	17	0.88127536	1995
NUCLEOTIDE-BINDING DOMAIN, LEUCINE RICH REPEAT CONTAINING RECEPTOR SIGNALING PATHWAY%GOBP%GO:0035872	0.3936687	1.2042192	26	0.8812856	9917
ZBP1(DAI) MEDIATED INDUCTION OF TYPE I IFNS%REACTOME%R-HSA-1606322.1	0.41811085	1.2102908	20	0.88130134	4266
PURINE NUCLEOTIDE CATABOLIC PROCESS%GOBP%GO:0006195	0.43001363	1.3356903	33	0.88130313	5021
NEGATIVE REGULATION OF PROTEIN MATURATION%GOBP%GO:1903318	0.40235412	1.1908878	25	0.8813107	1780
HISTONE MONOUBIQUITINATION%GOBP%GO:0010390	0.38950336	1.2040588	29	0.88139427	2545
REGULATION OF B CELL ACTIVATION%GOBP%GO:0050864	0.35703462	1.1881881	65	0.88140774	8693

SPHINGOLIPID CATABOLIC PROCESS%GOBP%GO:0030149	0.44777882	1.3399671	24	0.88140786	6316
PID_PI3KCI_PATHWAY%MSIGDB_C2%PID_PI3KCI_PATHWAY	0.3800063	1.1876475	32	0.8814365	5574
POSITIVE REGULATION OF AXONOGENESIS%GOBP%GO:0050772	0.36365008	1.1888566	49	0.88146204	2398
REGULATION OF CARDIAC CONDUCTION%GOBP%GO:1903779	0.34909034	1.1577648	63	0.88147867	8400
ADENYLATE CYCLASE-ACTIVATING G PROTEIN-COUPLED RECEPTOR SIGNALING PATHWAY%GOBP%GO:0007189	0.38673803	1.3359299	90	0.8814994	6707
SEMA4D IN SEMAPHORIN SIGNALING%REACTOME%R-HSA-400685.2	0.38830233	1.1464024	23	0.88153034	3804
CD4-POSITIVE, ALPHA-BETA T CELL ACTIVATION%GOBP%GO:0035710	0.4301631	1.2121631	16	0.88156027	1876
REGULATION OF MONONUCLEAR CELL PROLIFERATION%GOBP%GO:0032944	0.38544723	1.3351214	108	0.88156086	5816
POSITIVE REGULATION OF STRESS FIBER ASSEMBLY%GOBP%GO:0051496	0.37285182	1.1896375	41	0.8815919	6955
BIOCARTA_AT1R_PATHWAY%MSIGDB_C2%BIOCARTA_AT1R_PATHWAY	0.41094515	1.2045803	23	0.88162225	10579
BLOOD GROUP SYSTEMS BIOSYNTHESIS%REACTOME%R-HSA-9033658.2	0.39860192	1.1469299	19	0.8816526	5235
PID_AR_NONGENOMIC_PATHWAY%MSIGDB_C2%PID_AR_NONGENOMIC_PATHWAY	0.38361827	1.1460285	26	0.8816574	10474
HYPOXIC AND OXYGEN HOMEOSTASIS REGULATION OF HIF-1-ALPHA%PATHWAY INTERACTION DATABASE NCI-NATURE CURATED DATA%HYPOXIC AND OXYGEN HOMEOSTASIS REGULATION OF HIF-1-ALPHA	0.40022212	1.1607631	19	0.8816582	9541
REGULATION OF INTERLEUKIN-4 PRODUCTION%GOBP%GO:0032673	0.41795224	1.1897577	18	0.88165873	4770
HALLMARK_G2M_CHECKPOINT%MSIGDB_C2%HALLMARK_G2M_CHECKPOINT	0.34700617	1.2106595	134	0.8816594	6377
PID_AJDISS_2PATHWAY%MSIGDB_C2%PID_AJDISS_2PATHWAY	0.382631	1.1983988	37	0.881671	1720
REGULATION OF G0 TO G1 TRANSITION%GOBP%GO:0070316	0.35911855	1.1464841	40	0.881674	5189
DISASSEMBLY OF THE DESTRUCTION COMPLEX AND RECRUITMENT OF AXIN TO THE MEMBRANE%REACTOME%R-HSA-4641262.3	0.39397198	1.2047132	28	0.8816957	2071
FCGR3A-MEDIATED IL10 SYNTHESIS%REACTOME DATABASE ID RELEASE 75%9664323	0.3926187	1.2096392	29	0.8817188	2831
CELLULAR RESPONSE TO CAMP%GOBP%GO:0071320	0.38212702	1.1465808	25	0.88172156	3623
STIMULATORY C-TYPE LECTIN RECEPTOR SIGNALING PATHWAY%GOBP%GO:0002223	0.3496724	1.1877233	92	0.88172656	5498
PROTEIN IMPORT INTO MITOCHONDRIAL MATRIX%GOBP%GO:0030150	0.47896123	1.3412429	18	0.88174635	290
NEGATIVE REGULATION OF PROTEIN PROCESSING%GOBP%GO:0010955	0.40235412	1.2099209	25	0.88176525	1780
POSITIVE REGULATION OF CATION TRANSMEMBRANE TRANSPORT%GOBP%GO:1904064	0.3593046	1.2239672	89	0.8818068	4165
SPHINGOLIPID DE NOVO BIOSYNTHESIS%REACTOME DATABASE ID RELEASE 75%1660661	0.35796317	1.1455102	40	0.88180935	7226
HEART TRABECULA MORPHOGENESIS%GOBP%GO:0061384	0.41222772	1.1467843	17	0.881813	8254
E-CADHERIN SIGNALING IN THE NASCENT ADHERENS JUNCTION%PATHWAY INTERACTION DATABASE NCI-NATURE CURATED DATA%E-CADHERIN SIGNALING IN THE NASCENT ADHERENS JUNCTION	0.365997	1.1578106	36	0.881823	1727
REGULATION OF GASTRULATION%GOBP%GO:0010470	0.3729974	1.1466607	32	0.8818738	6298
SIGNALING BY ERBB2 KD MUTANTS%REACTOME%R-HSA-9664565.1	0.4049418	1.1579136	20	0.8819268	11130
VOLTAGE GATED POTASSIUM CHANNELS%REACTOME%R-HSA-1296072.2	0.38349557	1.2049439	34	0.8819487	6517
REGULATION OF ANDROGEN RECEPTOR SIGNALING PATHWAY%GOBP%GO:0060765	0.5089216	1.533765	26	0.8819559	1090
DNA DAMAGE RESPONSE (ONLY ATM DEPENDENT)%WIKIPATHWAYS_20210110%WP710%HOMO SAPIENS	0.36115018	1.2301742	84	0.8819769	7004
REGULATION OF TORC1 SIGNALING%GOBP%GO:1903432	0.3690399	1.1453544	36	0.8819913	8759
CELLULAR RESPONSE TO INTERFERON-GAMMA%GOBP%GO:0071346	0.33985037	1.1607959	87	0.8820896	4909
CHROMOSOME ORGANIZATION INVOLVED IN MEIOTIC CELL CYCLE%GOBP%GO:0070192	0.39518273	1.2086917	30	0.88211334	1997
DOWNSTREAM SIGNALING EVENTS OF B CELL RECEPTOR (BCR)%REACTOME DATABASE ID RELEASE 75%1168372	0.3583355	1.2089834	76	0.8821169	8770
OXIDATIVE DEMETHYLATION%GOBP%GO:0070989	0.43414313	1.1984408	15	0.88215053	8790
CELLULAR RESPONSE TO ENVIRONMENTAL STIMULUS%GOBP%GO:0104004	0.3226022	1.1460305	202	0.8821718	4807
REGULATION OF PROTEIN LOCALIZATION TO PLASMA MEMBRANE%GOBP%GO:1903076	0.3370674	1.1469302	81	0.88217586	8928
MITOCHONDRIAL FATTY ACID BETA-OXIDATION%REACTOME DATABASE ID RELEASE 75%77289	0.39315927	1.2240487	36	0.88218194	1358
ACETYLCHOLINE RECEPTOR SIGNALING PATHWAY%GOBP%GO:0095500	0.41800025	1.2295121	22	0.88218504	5169
REGULATION OF SISTER CHROMATID SEGREGATION%GOBP%GO:0033045	0.34049493	1.1574887	79	0.88218844	9847
INTRACELLULAR CHOLESTEROL TRANSPORT%GOBP%GO:0032367	0.4106867	1.1470395	17	0.8821895	7128
GLUCONEOGENESIS I%HUMANCYC%GLUCONEO-PWY	0.42877734	1.2047501	18	0.88223374	3328
7-METHYLGUANOSINE RNA CAPPING%GOBP%GO:0009452	0.3898842	1.2106873	33	0.882261	6737
HOST INTERACTIONS OF HIV FACTORS%REACTOME%R-HSA-162909.1	0.34586957	1.2027518	123	0.88226426	4674
TOLL LIKE RECEPTOR 9 (TLR9) CASCADE%REACTOME%R-HSA-168138.2	0.33681956	1.1455227	90	0.8822778	10539
REGULATION OF ENDOPLASMIC RETICULUM STRESS-INDUCED INTRINSIC APOPTOTIC SIGNALING PATHWAY%GOBP%GO:1902235	0.41332856	1.2028962	20	0.8822883	2200
PROTEIN MATURATION%GOBP%GO:0051604	0.34038833	1.2023343	180	0.8822899	8887
REGULATION OF PROTEIN MATURATION%GOBP%GO:1903317	0.36024845	1.2096719	67	0.88231367	3829
DRUG CATABOLIC PROCESS%GOBP%GO:0042737	0.35579133	1.205011	82	0.8823223	5625
DISEASES OF MITOTIC CELL CYCLE%REACTOME%R-HSA-9675126.2	0.36723325	1.1572112	37	0.8823275	9837
REGULATION OF PEPTIDYL-TYROSINE PHOSPHORYLATION%GOBP%GO:0050730	0.33580923	1.1877323	154	0.88233864	10380
HEART CONTRACTION%GOBP%GO:0060047	0.3732412	1.2302659	59	0.88235205	7804
MICROTUBULE DEPOLYMERIZATION%GOBP%GO:0007019	0.40755767	1.1456236	16	0.88235503	12892
INTERMEDIATE FILAMENT ORGANIZATION%GOBP%GO:0045109	0.41602546	1.1579378	16	0.88236415	12458
B CELL ACTIVATION%REACTOME DATABASE ID RELEASE 75%983705	0.38470995	1.3352449	97	0.8824227	8770
OOGENESIS%GOBP%GO:0048477	0.39382988	1.1986632	28	0.882442	9238
REGULATION OF VIRAL RELEASE FROM HOST CELL%GOBP%GO:1902186	0.3796431	1.1608377	27	0.88245034	11573
PLATELET AGGREGATION%GOBP%GO:0070527	0.38188738	1.1566889	26	0.8824528	6750
POSITIVE REGULATION OF TRANSMEMBRANE RECEPTOR PROTEIN SERINE/THREONINE KINASE SIGNALING PATHWAY%GOBP%GO:0090100	0.34240443	1.155965	81	0.8824592	6752
LENS DEVELOPMENT IN CAMERA-TYPE EYE%GOBP%GO:0002088	0.3815263	1.1470863	26	0.8825012	10969
DOWNSTREAM SIGNALING OF ACTIVATED FGFR3%REACTOME DATABASE ID RELEASE 75%5654708	0.42577106	1.1878294	15	0.8825122	5602
NEGATIVE REGULATION OF PROTEASOMAL UBIQUITIN-DEPENDENT PROTEIN CATABOLIC PROCESS%GOBP%GO:0032435	0.37780678	1.1587338	29	0.8825191	6445
ANGIOGENESIS%WIKIPATHWAYS_20210110%WP1539%HOMO SAPIENS	0.44082552	1.2078522	15	0.8825209	7352
REGULATION OF VIRAL PROCESS%GOBP%GO:0050792	0.33661702	1.2021447	189	0.88252187	6669
RESPONSE TO GLUCOSE%GOBP%GO:0009749	0.36602452	1.2055547	50	0.88252324	11443
HINDBRAIN MORPHOGENESIS%GOBP%GO:0021575	0.42109555	1.2051085	19	0.8825419	199
REGULATION OF CELLULAR AMINE METABOLIC PROCESS%GOBP%GO:0033238	0.34385845	1.1456945	60	0.8825432	8211
PHOSPHATIDYLGLYCEROL METABOLIC PROCESS%GOBP%GO:0046471	0.38477772	1.1580176	26	0.8825505	12555
GUANOSINE-CONTAINING COMPOUND METABOLIC PROCESS%GOBP%GO:1901068	0.38338307	1.1584854	26	0.8825737	2413
CELLULAR RESPONSE TO INORGANIC SUBSTANCE%GOBP%GO:0071241	0.33228424	1.1560512	128	0.8826025	2710
BIOCARTA_ATRBRCA_PATHWAY%MSIGDB_C2%BIOCARTA_ATRBRCA_PATHWAY	0.4037507	1.1572713	19	0.8826223	12221
NEGATIVE REGULATION OF RESPONSE TO WOUNDING%GOBP%GO:1903035	0.3683274	1.2091702	55	0.8826283	2064
ESTABLISHMENT OF MITOTIC SPINDLE ORIENTATION%GOBP%GO:0000132	0.40222538	1.1585935	21	0.88263535	5097
POSITIVE REGULATION OF CYTOSKELETON ORGANIZATION%GOBP%GO:0051495	0.34354454	1.2093166	164	0.8826497	6264
REGULATION OF CELL CYCLE G1/S PHASE TRANSITION%GOBP%GO:1902806	0.34194905	1.2029597	141	0.88267595	6445
NONHOMOLOGOUS END-JOINING (NHEJ)%REACTOME%R-HSA-5693571.1	0.37089494	1.198474	45	0.8826784	6189
RENIN ANGIOTENSIN ALDOSTERONE SYSTEM (RAAS)%WIKIPATHWAYS_20210110%WP4756%HOMO SAPIENS	0.38717997	1.1568792	24	0.8827163	8799
CELL FATE COMMITMENT%GOBP%GO:0045165	0.33461228	1.1589313	108	0.8827295	9791
FGFR1 MUTANT RECEPTOR ACTIVATION%REACTOME%R-HSA-1839124.2	0.42173687	1.2582495	23	0.8827555	7425
REGULATION OF CATION CHANNEL ACTIVITY%GOBP%GO:2001257	0.3272403	1.1471483	130	0.88275814	5740
POSITIVE REGULATION OF CELL GROWTH%GOBP%GO:0030307	0.3486866	1.209003	102	0.88275963	9435
ADP METABOLIC PROCESS%GOBP%GO:0046031	0.38693646	1.2087059	33	0.8827601	4362
REGULATION OF MEMBRANE PROTEIN ECTODOMAIN PROTEOLYSIS%GOBP%GO:0051043	0.40078872	1.1450672	19	0.8827681	2471
ER-PHAGOSOME PATHWAY%REACTOME DATABASE ID RELEASE 75%1236974	0.35642406	1.2107253	79	0.88281745	8770
REGULATION OF PROTEIN EXIT FROM ENDOPLASMIC RETICULUM%GOBP%GO:0070861	0.38986894	1.1583165	25	0.8828197	7178
T-CELL ANTIGEN RECEPTOR (TCR) SIGNALING PATHWAY%WIKIPATHWAYS_20210110%WP69%HOMO SAPIENS	0.3478989	1.1581957	63	0.8828234	5574
EPHA2 FORWARD SIGNALING%PATHWAY INTERACTION DATABASE NCI-NATURE CURATED DATA%EPHA2 FORWARD SIGNALING	0.42290264	1.2240798	19	0.8828239	4446
POSITIVE REGULATION OF AMINE TRANSPORT%GOBP%GO:0051954	0.45599875	1.2580603	16	0.88283366	4612
LYMPHOCYTE MIGRATION%GOBP%GO:0072676	0.3731339	1.1486456	31	0.8828418	4803
DEPURINATION%REACTOME DATABASE ID RELEASE 75%73927	0.38191703	1.158789	27	0.8828425	5210
DNA DAMAGE RESPONSE%WIKIPATHWAYS_20210110%WP707%HOMO SAPIENS	0.34864828	1.1446966	54	0.8828434	5356
POSITIVE REGULATION OF HEART GROWTH%GOBP%GO:0060421	0.43360654	1.2242182	18	0.8828467	5621
SIGNALING BY NOTCH3%REACTOME%R-HSA-9012852.2	0.36241966	1.1580641	45	0.8828821	3400
L-ALPHA-AMINO ACID TRANSMEMBRANE TRANSPORT%GOBP%GO:1902475	0.3870777	1.1567022	26	0.8829467	1776
POSITIVE REGULATION OF PROTEIN MATURATION%GOBP%GO:1903319	0.42827573	1.2023432	17	0.88295346	5561
PID_TNF_PATHWAY%MSIGDB_C2%PID_TNF_PATHWAY	0.39329478	1.2082232	34	0.8829571	6538
DIRECT P53 EFFECTORS%PATHWAY INTERACTION DATABASE NCI-NATURE CURATED DATA%DIRECT P53 EFFECTORS	0.35461295	1.2303079	131	0.8829767	5039
INOSITOL PHOSPHATE METABOLIC PROCESS%GOBP%GO:0043647	0.35402614	1.1589965	50	0.8829824	7173
INOSITOL PHOSPHATE-MEDIATED SIGNALING%GOBP%GO:0048016	0.4399345	1.2080593	15	0.8829908	6342
REGULATION OF MRNA POLYADENYLATION%GOBP%GO:1900363	0.43151313	1.2255893	18	0.88301283	7092
HIV TRANSCRIPTION INITIATION%REACTOME DATABASE ID RELEASE 75%167161	0.41652453	1.3413218	44	0.883022	6737
POSITIVE REGULATION OF PROTEIN IMPORT%GOBP%GO:1904591	0.37065312	1.1448872	28	0.8830313	6843
G PROTEIN-COUPLED RECEPTOR SIGNALING PATHWAY, COUPLED TO CYCLIC NUCLEOTIDE SECOND MESSENGER%GOBP%GO:0007187	0.3279673	1.1482595	148	0.88303983	7276
VASOPRESSIN REGULATES RENAL WATER HOMEOSTASIS VIA AQUAPORINS%REACTOME%R-HSA-432040.2	0.36494288	1.1560736	39	0.88305044	9789
FC-GAMMA RECEPTOR SIGNALING PATHWAY%GOBP%GO:0038094	0.34693912	1.1471956	64	0.883074	6350
7-METHYLGUANOSINE MRNA CAPPING%GOBP%GO:0006370	0.3898842	1.1989511	33	0.8830814	6737
REGULATION OF RESPONSE TO NUTRIENT LEVELS%GOBP%GO:0032107	0.4349836	1.2112579	15	0.88309234	6910
PYRIMIDINE NUCLEOSIDE METABOLIC PROCESS%GOBP%GO:0006213	0.3818908	1.1569189	31	0.8830966	4355
ASYMMETRIC LOCALIZATION OF PCP PROTEINS%REACTOME DATABASE ID RELEASE 75%4608870	0.36117056	1.198671	61	0.8831054	8770
SPHINGOSINE METABOLIC PROCESS%GOBP%GO:0006670	0.46575674	1.3399676	19	0.8831107	5616
HALLMARK_REACTIVE_OXYGEN_SPECIES_PATHWAY%MSIGDB_C2%HALLMARK_REACTIVE_OXYGEN_SPECIES_PATHWAY	0.39074916	1.2051293	31	0.8831478	6298
NUCLEOTIDE PHOSPHORYLATION%GOBP%GO:0046939	0.3802405	1.2078705	40	0.8831594	4362
REGULATION OF CELL CYCLE ARREST%GOBP%GO:0071156	0.3557138	1.2304215	96	0.88317776	5039
POSITIVE REGULATION OF EPIDERMAL GROWTH FACTOR RECEPTOR SIGNALING PATHWAY%GOBP%GO:0045742	0.38105306	1.1483452	26	0.883187	9727
NEGATIVE REGULATION OF REACTIVE OXYGEN SPECIES METABOLIC PROCESS%GOBP%GO:2000378	0.38425732	1.2055602	33	0.8832163	4837
PATHWAY	0.37210605	1.1447215	30	0.88324124	2429
NETWORK	0.36030495	1.1562611	40	0.8832867	12786
IL1%NETPATH%IL1	0.3510551	1.1472572	52	0.88333195	10153
NEGATIVE REGULATION OF CATABOLIC PROCESS%GOBP%GO:0009895	0.31923553	1.1484274	241	0.8833378	5005
MONOSACCHARIDE CATABOLIC PROCESS%GOBP%GO:0046365	0.37675515	1.2256807	51	0.8833466	11215
MYD88 DEPENDENT CASCADE INITIATED ON ENDOSOME%REACTOME DATABASE ID RELEASE 75%975155	0.3363212	1.1486474	86	0.8833664	10539
INTERMEDIATE FILAMENT-BASED PROCESS%GOBP%GO:0045103	0.39413747	1.202398	29	0.88337874	8092
RIBONUCLEOSIDE TRIPHOSPHATE METABOLIC PROCESS%GOBP%GO:0009199	0.33963284	1.2108996	184	0.8834077	6762

BIOCARTA_FMLP_PATHWAY%MSIGDB_C2%BIOCARTA_FMLP_PATHWAY	0.4054265	1.2110454	25	0.8834171	11273
REGULATION OF DEFENSE RESPONSE TO VIRUS BY HOST%GOBP%GO:0050691	0.40472177	1.2113312	27	0.8834464	3609
REGULATION OF PLK1 ACTIVITY AT G2 M TRANSITION%REACTOME%R-HSA-2565942.1	0.3599335	1.2242533	85	0.8834482	9743
POSITIVE REGULATION OF ANION TRANSPORT%GOBP%GO:1903793	0.39271745	1.2082741	28	0.8834576	9573
REGULATION OF SYSTEMIC ARTERIAL BLOOD PRESSURE BY HORMONE%GOBP%GO:0001990	0.3953136	1.1590122	19	0.883463	10116
RNA SPLICING, VIA ENDONUCLEOLYTIC CLEAVAGE AND LIGATION%GOBP%GO:0000394	0.48068792	1.3401767	16	0.883468	3959
ONE-CARBON METABOLIC PROCESS%GOBP%GO:0006730	0.4063023	1.2107364	24	0.88351417	5644
ATRIOVENTRICULAR VALVE MORPHOGENESIS%GOBP%GO:0003181	0.41133806	1.148039	18	0.8835526	4558
REGULATION OF LYMPHOCYTE MIGRATION%GOBP%GO:2000401	0.38214415	1.2056314	37	0.88355875	5015
CLASS A 1 (RHODOPSIN-LIKE RECEPTORS)%REACTOME%R-HSA-373076.8	0.3253778	1.1545286	187	0.88356674	6819
LOSS OF NLP FROM MITOTIC CENTROSOMES%REACTOME%R-HSA-380259.1	0.345471	1.1560793	68	0.88357824	9743
NEGATIVE REGULATION OF CELLULAR PROTEIN LOCALIZATION%GOBP%GO:1903828	0.36889446	1.2582749	84	0.8835956	6005
MITOCHONDRIAL CALCIUM ION TRANSMEMBRANE TRANSPORT%GOBP%GO:0006851	0.4165651	1.1989845	21	0.88361394	5945
EVENTS	0.40241975	1.2306502	27	0.88363975	11635
PROTEIN EXIT FROM ENDOPLASMIC RETICULUM%GOBP%GO:0032527	0.3873032	1.1563075	25	0.88364756	6658
REGULATION OF LYMPHOCYTE PROLIFERATION%GOBP%GO:0050670	0.38952187	1.3415103	107	0.88364977	5816
NEGATIVE REGULATION OF CELL CYCLE G1/S PHASE TRANSITION%GOBP%GO:1902807	0.33920252	1.1546178	93	0.88369185	6057
NETWORK	0.36538702	1.2051616	62	0.8837262	10989
NON-CANONICAL WNT SIGNALING PATHWAY%GOBP%GO:0035567	0.33372575	1.155095	111	0.8837303	8248
RIBOSOMAL LARGE SUBUNIT BIOGENESIS%GOBP%GO:0042273	0.34320992	1.1486868	70	0.8837316	8781
ENDOPLASMIC RETICULUM STRESS RESPONSE IN CORONAVIRUS INFECTION%WIKIPATHWAYS_20210110%WP4861%HOMO SAPIENS	0.39824355	1.258434	36	0.88373387	3713
RESPONSE TO UV%GOBP%GO:0009411	0.35437626	1.2245054	123	0.8837389	4807
MRNA STABILIZATION%GOBP%GO:0048255	0.37799028	1.1986817	38	0.88374865	1885
PROTEIN AUTOUBIQUITINATION%GOBP%GO:0051865	0.338565	1.1442641	63	0.88375026	3130
CARDIOCYTE DIFFERENTIATION%GOBP%GO:0035051	0.3745263	1.230468	49	0.88377327	7365
MODIFICATION OF MORPHOLOGY OR PHYSIOLOGY OF OTHER ORGANISM%GOBP%GO:0035821	0.33628437	1.1487932	87	0.88378596	5648
ANDROGENRECEPTOR%IOB%ANDROGENRECEPTOR	0.35274264	1.2057208	87	0.8838268	5039
POSITIVE REGULATION OF PROTEIN PROCESSING%GOBP%GO:0010954	0.41609094	1.1472584	15	0.88385266	5561
EMBRYONIC LIMB MORPHOGENESIS%GOBP%GO:0030326	0.36674008	1.155419	37	0.88385564	6311
THROMBOXANE SIGNALLING THROUGH TP RECEPTOR%REACTOME DATABASE ID RELEASE 75%428930	0.41602242	1.2257326	22	0.8838698	9314
BIOCARTA_TNFR1_PATHWAY%MSIGDB_C2%BIOCARTA_TNFR1_PATHWAY	0.39221525	1.15482	23	0.88387007	5356
POSITIVE REGULATION OF ACTIN FILAMENT BUNDLE ASSEMBLY%GOBP%GO:0032233	0.35409895	1.1443487	45	0.88387257	6955
P75(NTR)-MEDIATED SIGNALING%PATHWAY INTERACTION DATABASE NCI-NATURE CURATED DATA%P75(NTR)-MEDIATED SIGNALING	0.3446899	1.1590444	67	0.883879	7265
RNA POLYMERASE II HIV PROMOTER ESCAPE%REACTOME%R-HSA-167162.3	0.41652453	1.3417419	44	0.88388467	6737
POSITIVE REGULATION OF LEUKOCYTE ACTIVATION%GOBP%GO:0002696	0.3239008	1.1477315	187	0.88388646	6584
FANCONI ANEMIA PATHWAY%REACTOME%R-HSA-6783310.2	0.36310375	1.154939	37	0.88389546	3960
IL-1 SIGNALING PATHWAY%WIKIPATHWAYS_20210110%WP195%HOMO SAPIENS	0.38237628	1.2243176	43	0.8839279	10539
PROCESSING OF SMDT1%REACTOME%R-HSA-8949664.1	0.43582246	1.2058263	16	0.88401437	4454
PANCREATIC ADENOCARCINOMA PATHWAY%WIKIPATHWAYS_20210110%WP4263%HOMO SAPIENS	0.34679136	1.1488523	67	0.88405114	10579
REGULATION OF PROTEIN DEACETYLATION%GOBP%GO:0090311	0.37521684	1.1474513	29	0.8840639	7226
CELL DIVISION%GOBP%GO:0051301	0.34594345	1.2059538	127	0.88408417	5062
PHARYNGEAL SYSTEM DEVELOPMENT%GOBP%GO:0060037	0.4360573	1.1990271	16	0.8840903	7365
BETA-CATENIN INDEPENDENT WNT SIGNALING%REACTOME%R-HSA-3858494.3	0.3285574	1.154648	136	0.88409424	9672
NEGATIVE REGULATION OF PEPTIDYL-TYROSINE PHOSPHORYLATION%GOBP%GO:0050732	0.37783748	1.2113519	42	0.88409835	4988
REGULATION OF T CELL CYTOKINE PRODUCTION%GOBP%GO:0002724	0.41755277	1.2060916	20	0.884118	4417
POSITIVE REGULATION OF APOPTOTIC SIGNALING PATHWAY%GOBP%GO:2001235	0.35564262	1.2252244	116	0.8841225	6752
TOLL LIKE RECEPTOR 5 (TLR5) CASCADE%REACTOME DATABASE ID RELEASE 75%168176	0.34158704	1.1552427	82	0.8841274	10539
FACTOR NETWORK	0.43596667	1.3403412	32	0.88412863	7352
POSITIVE REGULATION OF CELL-CELL ADHESION%GOBP%GO:0022409	0.32585993	1.1477933	165	0.88414305	5015
LOSS OF PROTEINS REQUIRED FOR INTERPHASE MICROTUBULE ORGANIZATION FROM THE CENTROSOME%REACTOME%R-HSA-380284.1	0.345471	1.1540434	68	0.88414675	9743
REGULATION OF SPINDLE ASSEMBLY%GOBP%GO:0090169	0.37924546	1.1554732	26	0.8841549	8514
TOLL LIKE RECEPTOR 7 8 (TLR7 8) CASCADE%REACTOME DATABASE ID RELEASE 75%168181	0.3363212	1.147544	86	0.88417184	10539
POSITIVE REGULATION OF LYMPHOCYTE ACTIVATION%GOBP%GO:0051251	0.3380405	1.1994202	168	0.8842069	6584
NEGATIVE REGULATION OF IMMUNE EFFECTOR PROCESS%GOBP%GO:0002698	0.35888162	1.2245631	66	0.8842213	6496
NAD BIOSYNTHETIC PROCESS%GOBP%GO:0009435	0.3554028	1.1551024	46	0.88424045	4362
POSTSYNAPTIC MEMBRANE ORGANIZATION%GOBP%GO:0001941	0.43575674	1.1995479	17	0.88424706	1059
BIOCARTA_NFAT_PATHWAY%MSIGDB_C2%BIOCARTA_NFAT_PATHWAY	0.36860663	1.1595663	34	0.8843028	10230
TRANSPORT OF MATURE MRNA DERIVED FROM AN INTRON-CONTAINING TRANSCRIPT%REACTOME%R-HSA-159236.3	0.35843763	1.1996659	70	0.88432246	5209
RANKL/RANK (RECEPTOR ACTIVATOR OF NFKB (LIGAND)) SIGNALING PATHWAY%WIKIPATHWAYS_20210110%WP2018%HOMO SAPIENS	0.35038164	1.1491411	42	0.8843225	10539
NEGATIVE REGULATION OF STRESS FIBER ASSEMBLY%GOBP%GO:0051497	0.41216585	1.2257984	23	0.884332	5245
PKA ACTIVATION%REACTOME DATABASE ID RELEASE 75%163615	0.412015	1.1472682	17	0.8843465	2831
SEMAPHORIN INTERACTIONS%REACTOME DATABASE ID RELEASE 75%373755	0.34916413	1.1436614	61	0.8843497	4366
RIBONUCLEOSIDE DIPHOSPHATE METABOLIC PROCESS%GOBP%GO:0009185	0.36248213	1.1591828	39	0.88436866	4362
HIV LIFE CYCLE%REACTOME%R-HSA-162587.2	0.32949424	1.1590512	141	0.8844099	5206
INTRINSIC APOPTOTIC SIGNALING PATHWAY%GOBP%GO:0097193	0.3383569	1.1594161	112	0.88441926	5706
CARDIAC MUSCLE CONTRACTION%GOBP%GO:0060048	0.37700737	1.2306613	52	0.8844193	7804
HISTONE H4-K5 ACETYLATION%GOBP%GO:0043981	0.41039577	1.1488876	15	0.88443774	7498
REGULATION OF INTERLEUKIN-1 PRODUCTION%GOBP%GO:0032652	0.34933996	1.1502888	51	0.884493	6153
HALLMARK_IL6_JAK_STAT3_SIGNALING%MSIGDB_C2%HALLMARK_IL6_JAK_STAT3_SIGNALING	0.362901	1.2282778	65	0.88450164	9453
GLYCOSYLATION%GOBP%GO:0070085	0.33898246	1.2070295	195	0.88450724	6418
CELLULAR RESPIRATION%GOBP%GO:0045333	0.3416177	1.1997656	130	0.8845362	6628
REGULATION OF EPITHELIAL CELL MIGRATION%GOBP%GO:0010632	0.33932346	1.1990781	185	0.8845366	8463
CELLULAR RESPONSE TO ABIOTIC STIMULUS%GOBP%GO:0071214	0.3226022	1.1492131	202	0.88453686	4807
G ALPHA (S) SIGNALLING EVENTS%REACTOME DATABASE ID RELEASE 75%418555	0.34737712	1.2073069	120	0.88458174	6157
EYE PHOTORECEPTOR CELL DIFFERENTIATION%GOBP%GO:0001754	0.4183956	1.2259159	21	0.8845821	2387
MYD88 CASCADE INITIATED ON PLASMA MEMBRANE%REACTOME DATABASE ID RELEASE 75%975871	0.34158704	1.1540599	82	0.8846157	10539
VERY LONG-CHAIN FATTY ACID METABOLIC PROCESS%GOBP%GO:0000038	0.4118219	1.2575552	28	0.8846416	9354
REGULATION OF PROTEIN PROCESSING%GOBP%GO:0070613	0.36139503	1.2061234	65	0.88468033	3112
SNRNP ASSEMBLY%REACTOME%R-HSA-191859.3	0.3571843	1.1489484	50	0.88469225	7797
POSITIVE REGULATION OF PROTEIN POLYMERIZATION%GOBP%GO:0032273	0.33793727	1.1592367	96	0.8846928	6350
HISTONE H3-K4 METHYLATION%GOBP%GO:0051568	0.40260947	1.2246262	24	0.88471717	6287
POSITIVE REGULATION OF MULTI-ORGANISM PROCESS%GOBP%GO:0043902	0.34342945	1.2066972	143	0.88472116	6975
NUCLEOTIDE-EXCISION REPAIR, PREINCISION COMPLEX ASSEMBLY%GOBP%GO:0006294	0.39298233	1.2068373	29	0.88474077	4807
FATTY ACID BETA OXIDATION%WIKIPATHWAYS_20210110%WP143%HOMO SAPIENS	0.3897755	1.1436895	21	0.88474554	9399
PATHWAYS AFFECTED IN ADENOID CYSTIC CARCINOMA%WIKIPATHWAYS_20210110%WP3651%HOMO SAPIENS	0.37322757	1.2065395	50	0.8847486	12347
TOLL LIKE RECEPTOR 10 (TLR10) CASCADE%REACTOME DATABASE ID RELEASE 75%168142	0.34158704	1.1541355	82	0.8848225	10539
POSITIVE REGULATION OF PROTEOLYSIS INVOLVED IN CELLULAR PROTEIN CATABOLIC PROCESS%GOBP%GO:1903052	0.3355068	1.1492689	102	0.88482594	7254
ANTIGEN PROCESSING AND PRESENTATION OF ENDOGENOUS ANTIGEN%GOBP%GO:0019883	0.40465513	1.1503321	18	0.8848343	4842
GLOBAL GENOME NUCLEOTIDE-EXCISION REPAIR%GOBP%GO:0070911	0.3958568	1.2113531	26	0.8848398	8335
REGULATION OF METAL ION TRANSPORT%GOBP%GO:0010959	0.31806684	1.1438894	242	0.8848873	5256
NEGATIVE REGULATION OF RNA CATABOLIC PROCESS%GOBP%GO:1902369	0.3671579	1.2062242	55	0.8849	4713
C-TYPE LECTIN RECEPTORS (CLRS)%REACTOME DATABASE ID RELEASE 75%5621481	0.3528557	1.2307277	117	0.8849076	6342
REGULATION OF STRIATED MUSCLE CONTRACTION%GOBP%GO:0006942	0.4007389	1.3404816	68	0.8849135	6246
CELLULAR CARBOHYDRATE METABOLIC PROCESS%GOBP%GO:0044262	0.3585476	1.2316898	99	0.88492006	8902
RECOGNITION AND ASSOCIATION OF DNA GLYCOSYLASE WITH SITE CONTAINING AN AFFECTED PYRIMIDINE%REACTOME%R-HSA-110328.2	0.38022873	1.1991315	32	0.8849625	3166
REGULATION OF MITOCHONDRIAL MEMBRANE POTENTIAL%GOBP%GO:0051881	0.34705985	1.141197	45	0.88498414	9785
PID_NETRIN_PATHWAY%MSIGDB_C2%PID_NETRIN_PATHWAY	0.3820805	1.143173	25	0.8849909	7719
ACTIVIN RECEPTOR SIGNALING PATHWAY%GOBP%GO:0032924	0.4176315	1.1504184	15	0.88499653	9811
RNA STABILIZATION%GOBP%GO:0043489	0.3490952	1.1412997	46	0.88503367	2641
REGULATION OF MACROPHAGE MIGRATION%GOBP%GO:1905521	0.3816709	1.1422193	25	0.88508636	11417
REGULATION OF MITOTIC CELL CYCLE%REACTOME%R-HSA-453276.2	0.33668768	1.1437261	86	0.8850996	9584
POSITIVE REGULATION OF DNA BINDING%GOBP%GO:0043388	0.37582767	1.2070526	39	0.88509965	5141
DEADENYLATION OF MRNA%REACTOME DATABASE ID RELEASE 75%429947	0.3830416	1.1505098	25	0.885112	3452
NEGATIVE REGULATION OF ERBB SIGNALING PATHWAY%GOBP%GO:1901185	0.37029645	1.1997881	45	0.88511866	7831
NUCLEUS ORGANIZATION%GOBP%GO:0006997	0.35263768	1.2275376	93	0.8851254	4988
CYTOKINE PRODUCTION%GOBP%GO:0001816	0.3823437	1.2572781	58	0.8851397	9803
GLYCOLYSIS AND GLUCONEOGENESIS%WIKIPATHWAYS_20210110%WP534%HOMO SAPIENS	0.41621894	1.2571107	24	0.8851522	10263
REGULATION OF FIBROBLAST MIGRATION%GOBP%GO:0010762	0.42149457	1.2283078	21	0.88518167	2502
REGULATION OF PROTEASOMAL UBIQUITIN-DEPENDENT PROTEIN CATABOLIC PROCESS%GOBP%GO:0032434	0.32933363	1.1416153	111	0.8852035	7219
POSITIVE REGULATION OF LAMELLIPODIUM ASSEMBLY%GOBP%GO:0010592	0.38945004	1.1495414	23	0.8852041	6264
SAPIENS	0.34943062	1.1413734	50	0.8852364	4057
EML4 AND NUDC IN MITOTIC SPINDLE FORMATION%REACTOME%R-HSA-9648025.2	0.3371311	1.149655	94	0.88523775	6033
REGULATION OF PHOSPHATIDYLINOSITOL 3-KINASE ACTIVITY%GOBP%GO:0043551	0.37816665	1.143229	27	0.8852771	6797
REGULATION OF CHROMOSOME SEGREGATION%GOBP%GO:0051983	0.33529738	1.149754	99	0.88531023	9847
REGULATION OF UBIQUITIN-PROTEIN TRANSFERASE ACTIVITY%GOBP%GO:0051438	0.3568561	1.1505886	46	0.8853161	3536
NEGATIVE REGULATION OF CYTOSKELETON ORGANIZATION%GOBP%GO:0051494	0.3524913	1.2317736	118	0.8853209	8104
ACTIVATION OF ADENYLATE CYCLASE ACTIVITY%GOBP%GO:0007190	0.42209268	1.256899	26	0.8853226	3176
PD-1 SIGNALING%REACTOME%R-HSA-389948.2	0.4421396	1.2246673	16	0.88532686	8554
PI3K/AKT/MTOR - VITD3 SIGNALLING%WIKIPATHWAYS_20210110%WP4141%HOMO SAPIENS	0.4105807	1.141468	16	0.8853283	4020
MAPK6 MAPK4 SIGNALING%REACTOME%R-HSA-5687128.3	0.3369437	1.1492738	85	0.88534206	3511
HCMV EARLY EVENTS%REACTOME DATABASE ID RELEASE 75%9609690	0.34033403	1.1433219	72	0.88537467	8514
AMINO ACID TRANSPORT ACROSS THE PLASMA MEMBRANE%REACTOME%R-HSA-352230.3	0.38135323	1.1493822	24	0.88538164	6444
POSITIVE REGULATION OF MEMBRANE PERMEABILITY%GOBP%GO:1905710	0.38315582	1.1429721	21	0.88538617	9027
TRANSCRIPTIONAL REGULATION OF GRANULOPOIESIS%REACTOME%R-HSA-9616222.2	0.38395372	1.2259191	45	0.8853876	3179
REGULATION OF NMDA RECEPTOR ACTIVITY%GOBP%GO:2000310	0.37081453	1.1418008	28	0.88539076	327
VENTRICULAR CARDIAC MUSCLE CELL ACTION POTENTIAL%GOBP%GO:0086005	0.4432162	1.2062742	15	0.88539445	4811
PROTEIN LOCALIZATION TO NUCLEUS%GOBP%GO:0034504	0.3282329	1.141683	123	0.8854126	6387

RESPONSE TO CARBOHYDRATE%GOBP%GO:0009743	0.36215553	1.1998671	59	0.88541704	11443
NUCLEAR-TRANSCRIBED MRNA POLY(A) TAIL SHORTENING%GOBP%GO:0000289	0.4065399	1.2012924	20	0.88542944	3452
RELEASE OF CYTOCHROME C FROM MITOCHONDRIA%GOBP%GO:0001836	0.47413963	1.3417679	18	0.8854657	4030
REGULATION OF SYMBIOSIS, ENCOMPASSING MUTUALISM THROUGH PARASITISM%GOBP%GO:0043903	0.32345998	1.1499517	197	0.88549334	6669
PID_P75_NTR_PATHWAY%MSIGDB_C2%PID_P75_NTR_PATHWAY	0.3760414	1.2325181	53	0.8855311	7174
FGFR2 MUTANT RECEPTOR ACTIVATION%REACTOME%R-HSA-1839126.2	0.384551	1.1419989	24	0.8855326	6563
REGULATION OF PROTEIN TYROSINE KINASE ACTIVITY%GOBP%GO:0061097	0.34891427	1.1422302	58	0.8855617	9489
GLYCOGEN METABOLISM%REACTOME%R-HSA-8982491.1	0.40710753	1.2261959	26	0.8855829	8534
PLK1 SIGNALING EVENTS%PATHWAY INTERACTION DATABASE NCI-NATURE CURATED DATA%PLK1 SIGNALING EVENTS	0.3752993	1.1999719	45	0.885593	4410
POSITIVE REGULATION OF GROWTH%GOBP%GO:0045927	0.3527578	1.2342702	134	0.8856201	9435
PROTEIN LOCALIZATION TO MICROTUBULE CYTOSKELETON%GOBP%GO:0072698	0.38876966	1.2011127	32	0.88562536	8985
BIOCARTA_GPCR_PATHWAY%MSIGDB_C2%BIOCARTA_GPCR_PATHWAY	0.4257052	1.2266475	20	0.8856456	9509
VASCULAR PROCESS IN CIRCULATORY SYSTEM%GOBP%GO:0003018	0.36318648	1.23202	72	0.88564754	6319
BIOCARTA_PPARA_PATHWAY%MSIGDB_C2%BIOCARTA_PPARA_PATHWAY	0.3893839	1.2263392	38	0.88565314	10133
STRUCTURAL PATHWAY OF INTERLEUKIN 1 (IL-1)%WIKIPATHWAYS_20210110%WP2637%HOMO SAPIENS	0.38241255	1.2307452	40	0.88566214	10831
CORTOCOTROPIN RELEASING FACTOR RECEPTOR SIGNALING PATHWAY%PANTHER PATHWAY%P04380	0.4041122	1.2318655	29	0.885674	10042
NEURAL CREST CELL MIGRATION IN CANCER%WIKIPATHWAYS_20210110%WP4565%HOMO SAPIENS	0.39323965	1.2260139	30	0.8857056	10579
REGULATION OF VIRAL GENOME REPLICATION%GOBP%GO:0045069	0.3622402	1.2285306	85	0.88572836	4524
MACROMOLECULE GLYCOSYLATION%GOBP%GO:0043413	0.343438	1.2283611	185	0.88574934	6418
HCMV LATE EVENTS%REACTOME%R-HSA-9610379.2	0.34547076	1.1418294	68	0.88576186	5210
PROTEIN GLYCOSYLATION%GOBP%GO:0006486	0.34511513	1.2277277	184	0.8857644	6418
REGULATION OF POSTSYNAPTIC MEMBRANE NEUROTRANSMITTER RECEPTOR LEVELS%GOBP%GO:0099072	0.38045946	1.1506009	25	0.88580096	13223
POST-CHAPERONIN TUBULIN FOLDING PATHWAY%REACTOME DATABASE ID RELEASE 75%389977	0.40622577	1.1497571	17	0.8858332	3410
POSITIVE REGULATION OF EXTRINSIC APOPTOTIC SIGNALING PATHWAY%GOBP%GO:2001238	0.40388677	1.2308657	29	0.8858659	8404
PEROXISOMAL MEMBRANE TRANSPORT%GOBP%GO:0015919	0.4390167	1.2267628	16	0.88586915	3014
PROTEIN LOCALIZATION TO CENTROSOME%GOBP%GO:0071539	0.43384215	1.2275509	18	0.88588566	8985
POLYOL CATABOLIC PROCESS%GOBP%GO:0046174	0.44201985	1.2278562	16	0.8859496	8828
COMPLEMENT ACTIVATION%GOBP%GO:0006956	0.4053065	1.201311	21	0.8860293	6379
POSITIVE REGULATION OF INTERFERON-ALPHA PRODUCTION%GOBP%GO:0032727	0.40534243	1.142238	17	0.8860561	4751
COMPONENTS%REACTOME%R-HSA-141405.4	0.41705662	1.2325745	21	0.8860571	9837
REGULATION OF MICROTUBULE POLYMERIZATION%GOBP%GO:0031113	0.3783254	1.224681	45	0.88607484	5395
RET SIGNALING%REACTOME%R-HSA-8853659.4	0.38836354	1.2286187	35	0.88611686	4607
MESENCHYMAL CELL DIFFERENTIATION%GOBP%GO:0048762	0.35674626	1.2312901	106	0.8861737	8377
CARDIAC CONDUCTION%REACTOME%R-HSA-5576891.2	0.3438363	1.1999962	110	0.88618225	5620
PHOSPHATIDYLINOSITOL-MEDIATED SIGNALING%GOBP%GO:0048015	0.34317833	1.1506374	65	0.88618577	5543
PHOTORECEPTOR CELL DEVELOPMENT%GOBP%GO:0042461	0.4056451	1.1423235	19	0.8862153	2387
NUCLEAR-TRANSCRIBED MRNA CATABOLIC PROCESS, DEADENYLATION-DEPENDENT DECAY%GOBP%GO:0000288	0.37662086	1.2344714	54	0.88624614	3452
N-ACETYLGLUCOSAMINE METABOLIC PROCESS%GOBP%GO:0006044	0.44560954	1.2271528	15	0.88628334	1400
CELLULAR POLYSACCHARIDE METABOLIC PROCESS%GOBP%GO:0044264	0.37826425	1.2342988	51	0.8863176	8804
NUCLEAR-TRANSCRIBED MRNA CATABOLIC PROCESS, EXONUCLEOLYTIC%GOBP%GO:0000291	0.3662906	1.1424086	34	0.8863345	9274
PID_SYNDECAN_4_PATHWAY%MSIGDB_C2%PID_SYNDECAN_4_PATHWAY	0.3992333	1.1426303	20	0.8863827	11635
U12 DEPENDENT SPLICING%REACTOME DATABASE ID RELEASE 75%72165	0.37311438	1.2263554	52	0.886383	5533
PID_BCR_5PATHWAY%MSIGDB_C2%PID_BCR_5PATHWAY	0.37673512	1.2320436	48	0.8863864	4937
HEDGEHOG 'ON' STATE%REACTOME DATABASE ID RELEASE 75%5632684	0.35788563	1.2268165	81	0.8864153	8770
CAMP-MEDIATED SIGNALING%GOBP%GO:0019933	0.357011	1.2326658	108	0.8864415	6707
NEURON FATE COMMITMENT%GOBP%GO:0048663	0.43185398	1.2309147	18	0.886478	7707
PI5P, PP2A AND IER3 REGULATE PI3K AKT SIGNALING%REACTOME DATABASE ID RELEASE 75%6811558	0.3663768	1.2345892	76	0.8864894	8401
CARBOHYDRATE DERIVATIVE CATABOLIC PROCESS%GOBP%GO:1901136	0.3269932	1.1506829	144	0.8865148	5213
INTERMEDIATE FILAMENT CYTOSKELETON ORGANIZATION%GOBP%GO:0045104	0.40489686	1.2564921	28	0.8865331	8092
VPU MEDIATED DEGRADATION OF CD4%REACTOME DATABASE ID RELEASE 75%180534	0.35634306	1.1424798	50	0.88654226	8770
N-CADHERIN SIGNALING EVENTS%PATHWAY INTERACTION DATABASE NCI-NATURE CURATED DATA%N-CADHERIN SIGNALING EVENTS	0.37131277	1.1514993	33	0.88656807	10110
ATRIOVENTRICULAR VALVE DEVELOPMENT%GOBP%GO:0003171	0.42552593	1.2269382	20	0.88658905	4558
PHOTOPERIODISM%GOBP%GO:0009648	0.37784877	1.1515865	29	0.8867085	2423
VITAMIN D IN INFLAMMATORY DISEASES%WIKIPATHWAYS_20210110%WP4482%HOMO SAPIENS	0.4410282	1.2347106	18	0.8867345	8109
CELLULAR RESPONSE TO ORGANIC CYCLIC COMPOUND%GOBP%GO:0071407	0.32152715	1.1531318	221	0.88675857	8165
POSITIVE REGULATION OF PROTEIN COMPLEX ASSEMBLY%GOBP%GO:0031334	0.3206075	1.1519337	221	0.88678485	6568
ESTABLISHMENT OF PROTEIN LOCALIZATION TO MITOCHONDRION%GOBP%GO:0072655	0.34443116	1.1508518	67	0.88679665	12091
CELLULAR RESPONSE TO LIGHT STIMULUS%GOBP%GO:0071482	0.36709192	1.2557207	87	0.8868157	4807
POSITIVE REGULATION OF BLOOD VESSEL ENDOTHELIAL CELL MIGRATION%GOBP%GO:0043536	0.3604064	1.1518066	44	0.88684386	8377
G BETA:GAMMA SIGNALLING THROUGH PI3KGAMMA%REACTOME%R-HSA-392451.3	0.4076912	1.2000057	22	0.8868478	6094
HOMOLOGOUS RECOMBINATION%GOBP%GO:0035825	0.36648983	1.1507078	40	0.8869242	12396
BETA5 BETA6 BETA7 AND BETA8 INTEGRIN CELL SURFACE INTERACTIONS%PATHWAY INTERACTION DATABASE NCI-NATURE CURATED DATA%BETA5 BETA6 BETA7 AND BETA8 INTEGRIN CELL SURFACE INTERACTIONS	0.41733786	1.1512991	17	0.8869257	2064
DOWNSTREAM SIGNAL TRANSDUCTION%REACTOME%R-HSA-186763.2	0.41372705	1.2562296	26	0.88697237	6812
REGULATION OF T CELL PROLIFERATION%GOBP%GO:0042129	0.3639165	1.2320877	82	0.8869949	5015
MYD88-DEPENDENT TOLL-LIKE RECEPTOR SIGNALING PATHWAY%GOBP%GO:0002755	0.37846094	1.1509212	25	0.8870198	3389
ADP SIGNALLING THROUGH P2Y PURINOCEPTOR 12%REACTOME%R-HSA-392170.3	0.39685345	1.151996	20	0.887047	6094
MONOSACCHARIDE METABOLIC PROCESS%GOBP%GO:0005996	0.32726553	1.1406224	112	0.8870762	8383
EMBRYONIC SKELETAL SYSTEM MORPHOGENESIS%GOBP%GO:0048704	0.38843107	1.2338258	36	0.88708633	6311
FC-EPSILON RECEPTOR SIGNALING PATHWAY%GOBP%GO:0038095	0.33576486	1.1531743	99	0.8870928	7004
REGULATION OF CELL AGING%GOBP%GO:0090342	0.36935264	1.1516147	32	0.8871273	4842
RNA 5'-END PROCESSING%GOBP%GO:0000966	0.39101064	1.1522056	22	0.8871536	1901
RAS ACTIVATION UPON CA2+ INFLUX THROUGH NMDA RECEPTOR%REACTOME DATABASE ID RELEASE 75%442982	0.47308242	1.3417706	19	0.88719296	5550
SIGNALING BY FGFR1 IN DISEASE%REACTOME%R-HSA-5655302.2	0.40800515	1.2560147	29	0.8872284	7425
SIGNALING BY FGFR2%REACTOME DATABASE ID RELEASE 75%5654738	0.34839934	1.1523073	58	0.8872602	7470
ENERGY HOMEOSTASIS%GOBP%GO:0097009	0.41080022	1.239505	24	0.8872688	4571
ENDOSOME TRANSPORT VIA MULTIVESICULAR BODY SORTING PATHWAY%GOBP%GO:0032509	0.39795226	1.2326669	30	0.88729256	8586
POLYSACCHARIDE METABOLIC PROCESS%GOBP%GO:0005976	0.37723774	1.2558144	53	0.88729626	8804
CYCLIN D ASSOCIATED EVENTS IN G1%REACTOME DATABASE ID RELEASE 75%69231	0.38176784	1.2309153	44	0.8873239	6377
MITOTIC DNA DAMAGE CHECKPOINT%GOBP%GO:0044773	0.33572894	1.1525347	90	0.8873261	6040
POSITIVE REGULATION OF PROTEIN LOCALIZATION TO CELL PERIPHERY%GOBP%GO:1904377	0.38492262	1.2469085	52	0.887359	5218
ACTIVATION OF INNATE IMMUNE RESPONSE%GOBP%GO:0002218	0.32083878	1.1527584	204	0.8873664	5609
REGULATION OF ESTABLISHMENT OF PLANAR POLARITY%GOBP%GO:0090175	0.34942377	1.2006239	92	0.88738537	8248
NUCLEOTIDE EXCISION REPAIR%REACTOME%R-HSA-5696398.2	0.3338279	1.1520389	109	0.8874005	8457
ACTIN FILAMENT-BASED TRANSPORT%GOBP%GO:0099515	0.40935963	1.1526297	17	0.8874333	12795
RESPONSE TO HEXOSE%GOBP%GO:0009746	0.3748803	1.2347397	52	0.88744396	11443
GENE SILENCING BY RNA%GOBP%GO:0031047	0.34937927	1.1509322	59	0.88750684	7405
CYTOSOLIC DNA-SENSING PATHWAY%WIKIPATHWAYS_20210110%WP4655%HOMO SAPIENS	0.3662945	1.2000124	50	0.88752073	5533
POSITIVE REGULATION OF TRANSFORMING GROWTH FACTOR BETA RECEPTOR SIGNALING PATHWAY%GOBP%GO:0030511	0.39014193	1.1531996	22	0.887531	7534
NEGATIVE REGULATION OF INNATE IMMUNE RESPONSE%GOBP%GO:0045824	0.35872343	1.1510386	41	0.88755965	6024
SUBSTRATE ADHESION-DEPENDENT CELL SPREADING%GOBP%GO:0034446	0.39235085	1.2449737	42	0.8875621	5930
ENDOCARDIAL CUSHION DEVELOPMENT%GOBP%GO:0003197	0.36795667	1.1399183	29	0.8876396	8254
HALLMARK_DNA_REPAIR%MSIGDB_C2%HALLMARK_DNA_REPAIR	0.36030263	1.2447741	113	0.88770926	5953
POSITIVE REGULATION OF AXON EXTENSION%GOBP%GO:0045773	0.5269674	1.5340526	22	0.88772106	2093
NEGATIVE REGULATION OF CATION CHANNEL ACTIVITY%GOBP%GO:2001258	0.37725708	1.1401353	28	0.88774526	4230
POSITIVE REGULATION OF CALCIUM ION TRANSMEMBRANE TRANSPORTER ACTIVITY%GOBP%GO:1901021	0.37523335	1.1523173	27	0.8877625	9706
B CELL ACTIVATION INVOLVED IN IMMUNE RESPONSE%GOBP%GO:0002312	0.43950814	1.2395774	16	0.8877672	2943
G1 DNA DAMAGE CHECKPOINT%GOBP%GO:0044783	0.37829205	1.2470012	62	0.8878248	6040
G-PROTEIN BETA:GAMMA SIGNALLING%REACTOME DATABASE ID RELEASE 75%397795	0.3982844	1.2327241	28	0.88782495	6094
G PROTEIN-COUPLED ACETYLCHOLINE RECEPTOR SIGNALING PATHWAY%GOBP%GO:0007213	0.4635261	1.2546401	15	0.88784194	4871
MONOCARBOXYLIC ACID TRANSPORT%GOBP%GO:0015718	0.33543533	1.1399859	86	0.8878664	6327
POSITIVE REGULATION OF CARDIAC MUSCLE TISSUE DEVELOPMENT%GOBP%GO:0055025	0.40038943	1.152767	21	0.8878817	5634
RIP-MEDIATED NFKB ACTIVATION VIA ZBP1%REACTOME%R-HSA-1810476.2	0.43425557	1.2000797	17	0.8878936	4266
POSITIVE REGULATION OF HEART RATE%GOBP%GO:0010460	0.44670957	1.2450641	16	0.88797027	7588
POSITIVE REGULATION OF CELL ACTIVATION%GOBP%GO:0050867	0.3449655	1.2334927	194	0.8880277	6584
REGULATION OF EXECUTION PHASE OF APOPTOSIS%GOBP%GO:1900117	0.38783213	1.1397189	22	0.88803184	12553
REGULATION OF LEUKOCYTE PROLIFERATION%GOBP%GO:0070663	0.35607138	1.2328397	118	0.88803726	5816
POSITIVE REGULATION OF RAS PROTEIN SIGNAL TRANSDUCTION%GOBP%GO:0046579	0.35417354	1.1401788	47	0.8880631	3051
DNA STRAND ELONGATION%GOBP%GO:0022616	0.4106673	1.2349366	25	0.8880655	4939
BIOCARTA_BAD_PATHWAY%MSIGDB_C2%BIOCARTA_BAD_PATHWAY	0.41541925	1.1402822	15	0.8880959	4020
MYOGENESIS%REACTOME%R-HSA-525793.2	0.42314065	1.2551212	23	0.8881008	6651
LYMPHOCYTE COSTIMULATION%GOBP%GO:0031294	0.385852	1.2003154	31	0.8881434	4366
ADP SIGNALLING THROUGH P2Y PURINOCEPTOR 1%REACTOME DATABASE ID RELEASE 75%418592	0.42304277	1.2460501	22	0.88815546	9314
SCF-BETA-TRCP MEDIATED DEGRADATION OF EMI1%REACTOME%R-HSA-174113.3	0.34524342	1.13911	53	0.8881679	8770
INTERNAL PROTEIN AMINO ACID ACETYLATION%GOBP%GO:0006475	0.33410737	1.1395721	86	0.88818353	9297
NUCLEOTIDE-BINDING OLIGOMERIZATION DOMAIN CONTAINING SIGNALING PATHWAY%GOBP%GO:0070423	0.4168892	1.2329642	25	0.8882116	9917
GLYCOLYTIC PROCESS%GOBP%GO:0006096	0.408821	1.2470995	30	0.8882307	4362
REGULATION OF CELL SHAPE%GOBP%GO:0008360	0.362991	1.2347462	103	0.8882814	6196
REGULATION OF ORGANELLE ASSEMBLY%GOBP%GO:1902115	0.35127178	1.2458533	177	0.88828313	8876
CELL-CELL JUNCTION ASSEMBLY%GOBP%GO:0007043	0.39724827	1.3418512	85	0.8884029	8212
MITOCHONDRIAL COMPLEX I ASSEMBLY MODEL OXPHOS SYSTEM%WIKIPATHWAYS_20210110%WP4324%HOMO SAPIENS	0.35412094	1.1388144	45	0.88846093	6741
POSITIVE REGULATION OF SUPRAMOLECULAR FIBER ORGANIZATION%GOBP%GO:1902905	0.34029886	1.200107	149	0.88847405	6264
GLUCOSE CATABOLIC PROCESS%GOBP%GO:0006007	0.41905072	1.2391164	26	0.88856196	4362
TP53 REGULATES TRANSCRIPTION OF DNA REPAIR GENES%REACTOME%R-HSA-6796648.3	0.3716583	1.2350048	61	0.888573	7291
MITOTIC SPINDLE ASSEMBLY CHECKPOINT%GOBP%GO:0007094	0.42620462	1.2451245	21	0.888583	8983
AMYLOID-BETA METABOLIC PROCESS%GOBP%GO:0050435	0.4499141	1.2495865	15	0.8886108	7959
LYMPHOCYTE ACTIVATION%GOBP%GO:0046649	0.35543796	1.2471944	163	0.888629	5724

ENERGY DERIVATION BY OXIDATION OF ORGANIC COMPOUNDS%GOBP%GO:0015980	0.35177317	1.2395824	164	0.88864017	6628
TRANSCRIPTIONAL REGULATION BY VENTX%REACTOME DATABASE ID RELEASE 75%8853884	0.35853508	1.1391171	37	0.888653	9837
WNT SIGNALING IN KIDNEY DISEASE%WIKIPATHWAYS_20210110%WP4150%HOMO SAPIENS	0.40836734	1.2456037	27	0.88871783	7542
T-CELL ANTIGEN RECEPTOR (TCR) PATHWAY DURING STAPHYLOCOCCUS AUREUS INFECTION%WIKIPATHWAYS_20210110%WP3863%HOMO SAPIENS	0.406703	1.2546571	35	0.88873035	5574
ADENYLATE CYCLASE-MODULATING G PROTEIN-COUPLED RECEPTOR SIGNALING PATHWAY%GOBP%GO:0007188	0.323433	1.1388651	136	0.88874125	7276
CALCIUM-DEPENDENT CELL-CELL ADHESION VIA PLASMA MEMBRANE CELL ADHESION MOLECULES%GOBP%GO:0016339	0.36843386	1.1392062	32	0.888768	5568
BIOCARTA_CERAMIDE_PATHWAY%MSIGDB_C2%BIOCARTA_CERAMIDE_PATHWAY	0.4215504	1.2461174	20	0.8887751	4417
MEMBRANE PROTEIN ECTODOMAIN PROTEOLYSIS%GOBP%GO:0006509	0.41809085	1.2440681	23	0.8887795	7959
REGULATION OF ANTIGEN RECEPTOR-MEDIATED SIGNALING PATHWAY%GOBP%GO:0050854	0.39192432	1.2452515	35	0.88882554	1297
PURINE RIBONUCLEOSIDE METABOLIC PROCESS%GOBP%GO:0046128	0.39070508	1.2528812	38	0.8888322	5423
NEF-MEDIATES DOWN MODULATION OF CELL SURFACE RECEPTORS BY RECRUITING THEM TO CLATHRIN ADAPTERS%REACTOME%R-HSA-164938.1	0.42318603	1.2454144	20	0.88884395	4455
ACTIVATION OF SMO%REACTOME%R-HSA-5635838.1	0.4794479	1.3425841	16	0.8888524	5559
NEGATIVE REGULATION OF RESPONSE TO EXTERNAL STIMULUS%GOBP%GO:0032102	0.34587848	1.2330078	199	0.8888574	5391
POSITIVE REGULATION OF VASCULATURE DEVELOPMENT%GOBP%GO:1904018	0.3584222	1.251467	131	0.88891137	7365
REGULATION OF TOLL-LIKE RECEPTOR SIGNALING PATHWAY%WIKIPATHWAYS_20210110%WP1449%HOMO SAPIENS	0.36963698	1.2530243	88	0.88898027	10539
APOPTOTIC MITOCHONDRIAL CHANGES%GOBP%GO:0008637	0.38875347	1.2547828	46	0.8890212	4139
REGULATION OF TRANSCRIPTION FROM RNA POLYMERASE II PROMOTER IN RESPONSE TO STRESS%GOBP%GO:0043618	0.36426926	1.2551306	99	0.8890263	6903
PROTEIN DEPOLYMERIZATION%GOBP%GO:0051261	0.4085658	1.2472781	31	0.88906664	5784
NEGATIVE REGULATION OF MYELOID CELL DIFFERENTIATION%GOBP%GO:0045638	0.39053667	1.2496641	40	0.88914174	7447
REGULATION OF SMALL GTPASE MEDIATED SIGNAL TRANSDUCTION%GOBP%GO:0051056	0.31690463	1.139235	235	0.8891755	7389
MUSCLE SYSTEM PROCESS%GOBP%GO:0003012	0.34724182	1.2384778	178	0.88923097	5758
GABA RECEPTOR ACTIVATION%REACTOME%R-HSA-977443.4	0.3858837	1.2512189	56	0.88929856	6719
INTRACELLULAR STEROL TRANSPORT%GOBP%GO:0032366	0.43439236	1.2330886	18	0.88930744	7128
ENDOCARDIAL CUSHION MORPHOGENESIS%GOBP%GO:0003203	0.42235947	1.2519169	25	0.88930845	8254
CEREBELLUM MORPHOGENESIS%GOBP%GO:0021587	0.43791693	1.253643	18	0.88938093	199
COPII VESICLE COATING%GOBP%GO:0048208	0.3766579	1.2478981	63	0.8894058	9586
PRODUCTION OF SMALL RNA INVOLVED IN GENE SILENCING BY RNA%GOBP%GO:0070918	0.3942175	1.2350124	32	0.8894088	7133
POSITIVE REGULATION OF SMALL GTPASE MEDIATED SIGNAL TRANSDUCTION%GOBP%GO:0051057	0.34214616	1.1384827	49	0.8894196	3051
CARDIAC MUSCLE CELL ACTION POTENTIAL INVOLVED IN CONTRACTION%GOBP%GO:0086002	0.40078118	1.2463388	33	0.8894401	6324
POSITIVE REGULATION OF MICROTUBULE POLYMERIZATION%GOBP%GO:0031116	0.40773305	1.2497729	27	0.88948274	10708
B CELL ACTIVATION%GOBP%GO:0042113	0.36680833	1.235643	68	0.88949853	3819
REGULATION OF DOPAMINE SECRETION%GOBP%GO:0014059	0.4203734	1.239586	23	0.8895148	1609
PID_INTEGRIN_A9B1_PATHWAY%MSIGDB_C2%PID_INTEGRIN_A9B1_PATHWAY	0.45619985	1.2473633	16	0.8895286	5442
DOPAMINERGIC NEURON DIFFERENTIATION%GOBP%GO:0071542	0.4199683	1.2461481	22	0.88953936	2385
CANONICAL WNT SIGNALING PATHWAY%GOBP%GO:0060070	0.37757224	1.2440903	60	0.8895641	3505
NEGATIVE REGULATION OF MRNA METABOLIC PROCESS%GOBP%GO:1903312	0.372431	1.249926	69	0.88958514	4424
REGULATION OF MITOTIC SPINDLE CHECKPOINT%GOBP%GO:1903504	0.4632568	1.2509807	15	0.8896076	10929
MITOTIC SPINDLE CHECKPOINT%GOBP%GO:0071174	0.42620462	1.2409158	21	0.88962424	8983
FC RECEPTOR MEDIATED STIMULATORY SIGNALING PATHWAY%GOBP%GO:0002431	0.34325686	1.1382003	65	0.88966185	6350
REGULATION OF CALCIUM ION TRANSMEMBRANE TRANSPORTER ACTIVITY%GOBP%GO:1901019	0.40445128	1.341929	63	0.88968235	9706
NEGATIVE REGULATION OF CELL-SUBSTRATE ADHESION%GOBP%GO:0010812	0.37629125	1.2435715	50	0.8896976	3189
OXYTOCIN RECEPTOR MEDIATED SIGNALING PATHWAY%PANTHER PATHWAY%P04391	0.38583997	1.253749	47	0.8897378	6094
CONVERSION FROM APC C:CDC20 TO APC C:CDH1 IN LATE ANAPHASE%REACTOME%R-HSA-176407.3	0.43457013	1.2514912	20	0.8897446	9837
NEGATIVE REGULATION OF EPITHELIAL CELL DIFFERENTIATION%GOBP%GO:0030857	0.43059826	1.2530576	20	0.88976777	7222
POSITIVE REGULATION OF STEROID METABOLIC PROCESS%GOBP%GO:0045940	0.4327178	1.2479895	20	0.88981175	8992
NUCLEOSIDE CATABOLIC PROCESS%GOBP%GO:0009164	0.41377544	1.2437102	24	0.8898279	4806
EPIBOLY INVOLVED IN WOUND HEALING%GOBP%GO:0090505	0.42372054	1.2385336	22	0.88984495	6802
DOWNSTREAM SIGNALING OF ACTIVATED FGFR2%REACTOME%R-HSA-5654696.1	0.43899658	1.2354099	17	0.88984805	5602
MECHANOREGULATION AND PATHOLOGY OF YAP/TAZ VIA HIPPO AND NON-HIPPO MECHANISMS%WIKIPATHWAYS_20210110%WP4534%HOMO SAPIENS	0.39452028	1.2350812	31	0.88988376	5943
GLYCOGEN BREAKDOWN (GLYCOGENOLYSIS)%REACTOME DATABASE ID RELEASE 75%70221	0.4591173	1.2531998	15	0.88991123	8534
FCERI MEDIATED NF-KB ACTIVATION%REACTOME DATABASE ID RELEASE 75%2871837	0.37161964	1.2519746	75	0.8899342	8770
POSITIVE REGULATION OF PROTEASOMAL PROTEIN CATABOLIC PROCESS%GOBP%GO:1901800	0.3329565	1.1367617	89	0.8899749	7219
SEMA4D INDUCED CELL MIGRATION AND GROWTH-CONE COLLAPSE%REACTOME%R-HSA-416572.3	0.43681028	1.2441822	19	0.8899867	3322
REGULATION OF NEURON MIGRATION%GOBP%GO:2001222	0.41489	1.2524828	27	0.89000463	5844
REGULATION OF AMINO ACID TRANSPORT%GOBP%GO:0051955	0.45607463	1.2486334	15	0.89002407	2717
ELECTRON TRANSPORT CHAIN%GOBP%GO:0022900	0.3535879	1.2357036	93	0.89002526	4191
APC C-MEDIATED DEGRADATION OF CELL CYCLE PROTEINS%REACTOME DATABASE ID RELEASE 75%174143	0.33668768	1.1365176	86	0.8900564	9584
BIOCARTA_VDR_PATHWAY%MSIGDB_C2%BIOCARTA_VDR_PATHWAY	0.42777112	1.2522961	21	0.89006895	5783
REGULATION OF ACTIVATED PAK-2P34 BY PROTEASOME MEDIATED DEGRADATION%REACTOME%R-HSA-211733.1	0.34835038	1.1366231	48	0.8900892	8770
PRODUCTION OF MIRNAS INVOLVED IN GENE SILENCING BY MIRNA%GOBP%GO:0035196	0.41347387	1.2489681	29	0.89011055	7133
REGULATION OF PYRUVATE DEHYDROGENASE (PDH) COMPLEX%REACTOME DATABASE ID RELEASE 75%204174	0.4544991	1.2396356	15	0.8901527	3074
ROLE OF CALCINEURIN-DEPENDENT NFAT SIGNALING IN LYMPHOCYTES%PATHWAY INTERACTION DATABASE NCI-NATURE CURATED DATA%ROLE OF CALCINEURIN-DEPENDENT NFAT SIGNALING IN LYMPHOCYTES	0.35562706	1.1382031	50	0.8901568	6250
ATP GENERATION FROM ADP%GOBP%GO:0006757	0.40222046	1.2378033	31	0.8902329	4362
MIDBRAIN DEVELOPMENT%GOBP%GO:0030901	0.37567157	1.2484204	61	0.89024293	2385
APOPTOTIC FACTOR-MEDIATED RESPONSE%REACTOME DATABASE ID RELEASE 75%111471	0.44946212	1.2499638	17	0.8903192	3599
REGULATION OF PROTEIN DEPOLYMERIZATION%GOBP%GO:1901879	0.4078746	1.3426275	59	0.8903195	8141
CELLULAR RESPONSE TO METAL ION%GOBP%GO:0071248	0.36057407	1.2515519	113	0.8903496	2710
PLATELET ACTIVATION, SIGNALING AND AGGREGATION%REACTOME DATABASE ID RELEASE 75%76002	0.34950307	1.2490932	218	0.89036953	7087
REGULATION OF POTASSIUM ION TRANSPORT%GOBP%GO:0043266	0.3686441	1.2409424	68	0.8903821	4230
FCERI MEDIATED CA+2 MOBILIZATION%REACTOME%R-HSA-2871809.1	0.44194812	1.2480505	19	0.89041173	7481
REGULATION OF RESPONSE TO ENDOPLASMIC RETICULUM STRESS%GOBP%GO:1905897	0.3660124	1.247372	82	0.8904349	6733
ALPHA-BETA T CELL DIFFERENTIATION%GOBP%GO:0046632	0.4011424	1.1357503	17	0.8904403	6153
PID_NCADHERIN_PATHWAY%MSIGDB_C2%PID_NCADHERIN_PATHWAY	0.41386536	1.2405946	24	0.89044493	2429
CARDIAC ATRIUM MORPHOGENESIS%GOBP%GO:0003209	0.41282034	1.1367688	16	0.8904525	7365
REGULATION OF ACTIN CYTOSKELETON REORGANIZATION%GOBP%GO:2000249	0.41029066	1.240233	29	0.8905208	7338
HISTONE UBIQUITINATION%GOBP%GO:0016574	0.39754876	1.248719	36	0.8905318	2545
HETEROCHROMATIN ORGANIZATION%GOBP%GO:0070828	0.43059364	1.238568	20	0.8905618	3947
GENE SILENCING BY MIRNA%GOBP%GO:0035195	0.38201284	1.241244	46	0.89057153	7405
T CELL COSTIMULATION%GOBP%GO:0031295	0.4026037	1.2481903	30	0.8905852	4366
REGULATION OF CELLULAR RESPONSE TO DRUG%GOBP%GO:2001038	0.44813877	1.2532461	17	0.8906257	1532
NCRNA 5'-END PROCESSING%GOBP%GO:0034471	0.43529144	1.2397165	20	0.8906283	1901
CELL JUNCTION ORGANIZATION%REACTOME%R-HSA-446728.2	0.37342653	1.2537631	75	0.89064044	8092
POSITIVE REGULATION OF CATION CHANNEL ACTIVITY%GOBP%GO:2001259	0.38515988	1.2350973	44	0.8906613	4165
PYRIMIDINE NUCLEOTIDE BIOSYNTHETIC PROCESS%GOBP%GO:0006221	0.45232695	1.2359058	15	0.89069015	561
DETECTION OF CHEMICAL STIMULUS%GOBP%GO:0009593	0.35850775	1.1358073	36	0.89070934	4165
TYPE I INTERFERON INDUCTION AND SIGNALING DURING SARS-COV-2 INFECTION%WIKIPATHWAYS_20210110%WP4868%HOMO SAPIENS	0.41194087	1.252005	27	0.8907346	10153
SIGNAL TRANSDUCTION INVOLVED IN CELLULAR RESPONSE TO AMMONIUM ION%GOBP%GO:1903831	0.41800025	1.2357308	22	0.8907554	5169
PURINE NUCLEOSIDE TRIPHOSPHATE METABOLIC PROCESS%GOBP%GO:0009144	0.34559762	1.2378654	184	0.890764	6762
MESENCHYME DEVELOPMENT%GOBP%GO:0060485	0.3570495	1.2505817	136	0.8907708	8377
NEGATIVE REGULATION OF REPRODUCTIVE PROCESS%GOBP%GO:2000242	0.42514226	1.2413644	21	0.89083165	8262
ADRENALINE,NORADRENALINE INHIBITS INSULIN SECRETION%REACTOME DATABASE ID RELEASE 75%400042	0.3783682	1.1355399	27	0.89088833	9484
ID RELEASE 75%179419	0.33620647	1.1367776	72	0.8909271	8898
SPERMATID DIFFERENTIATION%GOBP%GO:0048515	0.34124443	1.1352868	60	0.89095455	5296
REGULATION OF ENDOTHELIAL CELL CHEMOTAXIS%GOBP%GO:2001026	0.4475894	1.2418389	17	0.89096117	8377
ISG15 ANTIVIRAL MECHANISM%REACTOME%R-HSA-1169408.2	0.36997885	1.2409953	70	0.8909937	3301
SINGLE FERTILIZATION%GOBP%GO:0007338	0.34911665	1.1361799	50	0.8910582	3065
SPINDLE ASSEMBLY CHECKPOINT%GOBP%GO:0071173	0.42620462	1.2474313	21	0.8910717	8983
LIMB DEVELOPMENT%GOBP%GO:0060173	0.33955425	1.1358252	67	0.89112574	6311
NUCLEOBASE-CONTAINING SMALL MOLECULE CATABOLIC PROCESS%GOBP%GO:0034656	0.3961893	1.2416323	35	0.8911589	6212
PYRUVATE METABOLISM%REACTOME DATABASE ID RELEASE 75%70268	0.40442958	1.2364714	26	0.8911673	3074
PID_RET_PATHWAY%MSIGDB_C2%PID_RET_PATHWAY	0.3646753	1.135354	30	0.8911694	4607
HEXOSE METABOLIC PROCESS%GOBP%GO:0019318	0.3658018	1.2499804	93	0.8911722	8383
ABC-FAMILY PROTEINS MEDIATED TRANSPORT%REACTOME%R-HSA-382556.4	0.36039418	1.2371143	93	0.8911807	8274
REGULATION OF NATURAL KILLER CELL MEDIATED IMMUNITY%GOBP%GO:0002715	0.4325147	1.2379508	20	0.8912004	4842
STRIATED MUSCLE CONTRACTION%GOBP%GO:0006941	0.33676937	1.1360286	69	0.8912251	8428
ATTACHMENT OF SPINDLE MICROTUBULES TO KINETOCHORE%GOBP%GO:0008608	0.3823867	1.136825	23	0.8912289	3243
NUCLEOSIDE TRIPHOSPHATE METABOLIC PROCESS%GOBP%GO:0009141	0.34741685	1.2402472	200	0.8913539	6762
RECYCLING PATHWAY OF L1%REACTOME DATABASE ID RELEASE 75%437239	0.4117277	1.2538075	26	0.8913675	1267
REGULATION OF CLATHRIN-DEPENDENT ENDOCYTOSIS%GOBP%GO:2000369	0.44814593	1.23992	15	0.89137655	7891
REGULATION OF OXIDOREDUCTASE ACTIVITY%GOBP%GO:0051341	0.3679655	1.2397426	70	0.89139426	6350
SPHINGOID METABOLIC PROCESS%GOBP%GO:0046519	0.46739277	1.3419367	21	0.8914007	5616
HALLMARK_KRAS_SIGNALING_DN%MSIGDB_C2%HALLMARK_KRAS_SIGNALING_DN	0.35375902	1.2419207	119	0.8914137	9661
HCMV INFECTION%REACTOME DATABASE ID RELEASE 75%9609646	0.3597482	1.2414222	96	0.8914196	8531
HYPOXIA RESPONSE VIA HIF ACTIVATION%PANTHER PATHWAY%P00030	0.44977134	1.2429036	15	0.891456	653
ENDOPLASMIC RETICULUM ORGANIZATION%GOBP%GO:0007029	0.37087998	1.237226	65	0.89147896	2302
MYOMETRIAL RELAXATION AND CONTRACTION PATHWAYS%WIKIPATHWAYS_20210110%WP289%HOMO SAPIENS	0.32820845	1.1358584	105	0.89148235	10576
BIOCARTA_FCER1_PATHWAY%MSIGDB_C2%BIOCARTA_FCER1_PATHWAY	0.38003406	1.132432	24	0.89150286	9509
DEOXYRIBOSE PHOSPHATE METABOLIC PROCESS%GOBP%GO:0019692	0.41922197	1.2359146	24	0.8915239	5891
POSITIVE REGULATION OF ERAD PATHWAY%GOBP%GO:1904294	0.4564612	1.2500855	15	0.8915649	6533
PHOSPHOLIPID CATABOLIC PROCESS%GOBP%GO:0009395	0.3852232	1.1368517	25	0.8916162	6378
MIRNA REGULATION OF DNA DAMAGE RESPONSE%WIKIPATHWAYS_20210110%WP1530%HOMO SAPIENS	0.34807855	1.1316109	57	0.8916267	5356
MYELOID LEUKOCYTE MIGRATION%GOBP%GO:0097529	0.3750572	1.2423769	64	0.8916278	6631
DSRNA PROCESSING%GOBP%GO:0031050	0.3942175	1.2368723	32	0.89164025	7133
ANTIGEN PROCESSING AND PRESENTATION OF PEPTIDE ANTIGEN%GOBP%GO:0048002	0.31734526	1.1317033	171	0.89170456	8541
MYD88-INDEPENDENT TOLL-LIKE RECEPTOR SIGNALING PATHWAY%GOBP%GO:0002756	0.40086764	1.2373444	30	0.8917565	6560
DEGRADATION OF AXIN%REACTOME DATABASE ID RELEASE 75%4641257	0.34818143	1.1325922	53	0.89177173	8770

VESICLE TARGETING%GOBP%GO:0006903	0.3649591	1.2365191	86	0.89178777	9586
NEURAL CREST CELL DEVELOPMENT%GOBP%GO:0014032	0.34957662	1.1343904	47	0.8917982	9324
HISTONE H4-K8 ACETYLATION%GOBP%GO:0043982	0.41039577	1.1324649	15	0.89185876	7498
REGULATION OF DEVELOPMENTAL GROWTH%GOBP%GO:0048638	0.34959328	1.2360184	148	0.8918648	5930
PID_FCER1_PATHWAY%MSIGDB_C2%PID_FCER1_PATHWAY	0.38699135	1.2424978	39	0.89186543	5574
PLATELET HOMEOSTASIS%REACTOME%R-HSA-418346.2	0.33543932	1.1321099	80	0.891935	6373
REGULATION OF BLOOD VESSEL ENDOTHELIAL CELL MIGRATION%GOBP%GO:0043535	0.3350614	1.1345878	73	0.8919431	6322
HALLMARK_COAGULATION%MSIGDB_C2%HALLMARK_COAGULATION	0.3358562	1.132221	76	0.8919521	6584
SECOND-MESSENGER-MEDIATED SIGNALING%GOBP%GO:0019932	0.3468623	1.2419871	214	0.89199436	6440
EMBRYONIC DIGIT MORPHOGENESIS%GOBP%GO:0042733	0.47066012	1.3426348	18	0.8920453	7031
AUTODEGRADATION OF CDH1 BY CDH1:APC C%REACTOME DATABASE ID RELEASE 75%174084	0.34763005	1.136867	64	0.8920563	8898
STEM CELL PROLIFERATION%GOBP%GO:0072089	0.36406812	1.1317371	36	0.892065	9751
NEGATIVE REGULATION OF ACTIN FILAMENT BUNDLE ASSEMBLY%GOBP%GO:0032232	0.3761278	1.1344396	26	0.892077	5245
APPENDAGE DEVELOPMENT%GOBP%GO:0048736	0.33955425	1.1348958	67	0.8920929	6311
GLUCOSE METABOLIC PROCESS%GOBP%GO:0006006	0.34429485	1.132628	66	0.8921235	8383
METABOLISM OF WATER-SOLUBLE VITAMINS AND COFACTORS%REACTOME%R-HSA-196849.3	0.329065	1.1313789	105	0.8921448	6657
CARBOHYDRATE BIOSYNTHETIC PROCESS%GOBP%GO:0016051	0.3642473	1.2421246	87	0.89214945	8975
PROTEIN N-LINKED GLYCOSYLATION%GOBP%GO:0006487	0.37186638	1.2501509	68	0.89215803	8585
ERYTHROCYTE HOMEOSTASIS%GOBP%GO:0034101	0.35659534	1.1347702	40	0.8921643	3153
SPINDLE ORGANIZATION%GOBP%GO:0007051	0.32269302	1.1328387	144	0.8921903	6869
POSITIVE REGULATION OF STEM CELL DIFFERENTIATION%GOBP%GO:2000738	0.4164248	1.1327258	15	0.8921933	10819
RNA POLYMERASE I PROMOTER CLEARANCE%REACTOME DATABASE ID RELEASE 75%73854	0.37430626	1.2366091	66	0.89221185	3876
REGULATION OF CATION TRANSMEMBRANE TRANSPORT%GOBP%GO:1904062	0.31557608	1.1370567	238	0.8922269	5753
REGULATION OF MITOTIC SISTER CHROMATID SEGREGATION%GOBP%GO:0033047	0.37125278	1.2429307	68	0.8922311	9847
POSITIVE REGULATION OF T CELL ACTIVATION%GOBP%GO:0050870	0.32834283	1.1346159	121	0.8923384	5015
MITOCHONDRIAL MEMBRANE ORGANIZATION%GOBP%GO:0007006	0.32651496	1.1371347	121	0.89241225	9206
NUCLEOTIDE-EXCISION REPAIR%GOBP%GO:0006289	0.327972	1.1330054	102	0.8924419	8457
CARDIAC MUSCLE TISSUE MORPHOGENESIS%GOBP%GO:0055008	0.35931796	1.1311802	36	0.8925113	7588
POSITIVE REGULATION OF PEPTIDYL-THREONINE PHOSPHORYLATION%GOBP%GO:0010800	0.37793273	1.1317468	24	0.8925158	9706
PCP CE PATHWAY%REACTOME%R-HSA-4086400.2	0.3350761	1.1368724	89	0.89254546	8997
2'-DEOXYRIBONUCLEOTIDE METABOLIC PROCESS%GOBP%GO:0009394	0.41922197	1.24254	24	0.8925721	5891
BETA-CATENIN PHOSPHORYLATION CASCADE%REACTOME DATABASE ID RELEASE 75%196299	0.40877238	1.1333082	16	0.892599	2071
PROTEIN LOCALIZATION TO VACUOLE%GOBP%GO:0072665	0.34308517	1.1373171	63	0.89259976	12450
TRAF6 MEDIATED INDUCTION OF NFKB AND MAP KINASES UPON TLR7 8 OR 9 ACTIVATION%REACTOME DATABASE ID RELEASE 75%975138	0.33208388	1.1328491	85	0.89264023	10539
WOUND HEALING, SPREADING OF CELLS%GOBP%GO:0044319	0.42372054	1.2360312	22	0.8926769	6802
REGULATION OF NEURAL PRECURSOR CELL PROLIFERATION%GOBP%GO:2000177	0.3645153	1.1331532	35	0.8928029	4879
PID_ECADHERIN_NASCENT_AJ_PATHWAY%MSIGDB_C2%PID_ECADHERIN_NASCENT_AJ_PATHWAY	0.37270325	1.1330374	27	0.89282405	6938
FATTY ACID BETA-OXIDATION%GOBP%GO:0006635	0.35163903	1.1373708	48	0.8928503	9267
COENZYME METABOLIC PROCESS%GOBP%GO:0006732	0.31521636	1.137138	250	0.8929076	4362
MET PROMOTES CELL MOTILITY%REACTOME%R-HSA-8875878.1	0.37090963	1.13177	26	0.89291704	7302
POSITIVE REGULATION OF PROTEIN LOCALIZATION TO MEMBRANE%GOBP%GO:1905477	0.32997054	1.1333475	98	0.8929238	9133
ANTIVIRAL MECHANISM BY IFN-STIMULATED GENES%REACTOME%R-HSA-1169410.4	0.33403632	1.1340237	78	0.89294153	3301
REGULATION OF CALCINEURIN-NFAT SIGNALING CASCADE%GOBP%GO:0070884	0.39170194	1.129154	23	0.8931324	3097
APOPTOSIS%REACTOME%R-HSA-109581.3	0.32189727	1.13373	163	0.89323497	9027
NLS-BEARING PROTEIN IMPORT INTO NUCLEUS%GOBP%GO:0006607	0.41141623	1.1336151	16	0.8932651	5873
RECEPTOR METABOLIC PROCESS%GOBP%GO:0043112	0.32955962	1.1289974	76	0.8933428	9345
POSITIVE REGULATION OF ION TRANSMEMBRANE TRANSPORTER ACTIVITY%GOBP%GO:0032414	0.33431318	1.1292169	73	0.89334416	4165
REGULATION OF ACTIVIN RECEPTOR SIGNALING PATHWAY%GOBP%GO:0032925	0.39316553	1.1333536	20	0.89339375	10533
RESPONSE TO AMINO ACID STARVATION%GOBP%GO:1990928	0.35330936	1.1334487	42	0.8935009	4840
REGULATION OF APC C ACTIVATORS BETWEEN G1 S AND EARLY ANAPHASE%REACTOME%R-HSA-176408.3	0.3337813	1.1307282	79	0.89353925	9584
RNA POLYMERASE II TRANSCRIPTION PRE-INITIATION AND PROMOTER OPENING%REACTOME%R-HSA-73779.2	0.41652453	1.3426805	44	0.8935525	6737
DNA INTEGRITY CHECKPOINT%GOBP%GO:0031570	0.32182628	1.1292787	131	0.89356375	6145
MESODERM DEVELOPMENT%GOBP%GO:0007498	0.35071966	1.1298306	42	0.8935703	7849
MRNA DECAY BY 3' TO 5' EXORIBONUCLEASE%REACTOME%R-HSA-429958.2	0.480845	1.342951	16	0.89358675	2943
OOCYTE DIFFERENTIATION%GOBP%GO:0009994	0.41011083	1.1337435	16	0.89368343	4396
METANEPHROS DEVELOPMENT%GOBP%GO:0001656	0.3544781	1.1294734	39	0.8937097	7728
RESPONSE TO ARSENIC-CONTAINING SUBSTANCE%GOBP%GO:0046685	0.38386184	1.1307937	20	0.89374346	6249
DIOL BIOSYNTHETIC PROCESS%GOBP%GO:0034312	0.4045772	1.1293465	17	0.8937685	3189
REGULATION OF PHOSPHOLIPASE ACTIVITY%GOBP%GO:0010517	0.34682712	1.12956	49	0.89382064	12347
CALCIUM ION IMPORT%GOBP%GO:0070509	0.3636616	1.1304104	34	0.8939092	5310
REGULATION OF PROTEIN UBIQUITINATION%GOBP%GO:0031396	0.3182815	1.1298443	167	0.8939988	9937
REGULATION OF SYNAPTIC PLASTICITY%GOBP%GO:0048167	0.32656592	1.1296071	110	0.89410514	9347
ANTIGEN PROCESSING AND PRESENTATION OF PEPTIDE ANTIGEN VIA MHC CLASS II%GOBP%GO:0002495	0.33301568	1.1300311	91	0.8941422	9094
AORTIC VALVE MORPHOGENESIS%GOBP%GO:0003180	0.44181493	1.3431334	27	0.89418036	6191
HEMOSTASIS%GOBP%GO:0007599	0.3175247	1.1299102	193	0.8942047	5743
ADORA2B MEDIATED ANTI-INFLAMMATORY CYTOKINES PRODUCTION%REACTOME%R-HSA-9660821.1	0.329185	1.1304365	91	0.8943029	7220
VACUOLAR TRANSPORT%GOBP%GO:0007034	0.324687	1.130183	136	0.8944206	10590
PROTEIN LOCALIZATION TO MITOCHONDRION%GOBP%GO:0070585	0.33786467	1.1300722	70	0.8944495	9084
PRE-NOTCH TRANSCRIPTION AND TRANSLATION%REACTOME%R-HSA-1912408.4	0.46598184	1.5342195	52	0.8946622	6040
REGULATION OF RESPONSE TO DRUG%GOBP%GO:2001023	0.40442204	1.3431429	59	0.8959118	2423
AXON EXTENSION%GOBP%GO:0048675	0.38150632	1.1282512	24	0.8961315	2926
MITOCHONDRIAL RESPIRATORY CHAIN COMPLEX I ASSEMBLY%GOBP%GO:0032981	0.39926827	1.3433487	65	0.89640325	6741
OXIDATIVE STRESS%WIKIPATHWAYS_20210110%WP408%HOMO SAPIENS	0.3881503	1.12808	20	0.89642173	12576
MULTI-ORGANISM CELLULAR PROCESS%GOBP%GO:0044764	0.5297447	1.5360205	21	0.89703065	4455
G2/M TRANSITION OF MITOTIC CELL CYCLE%GOBP%GO:0000086	0.3235182	1.1276689	132	0.89720327	7225
CELL DIFFERENTIATION IN SPINAL CORD%GOBP%GO:0021515	0.41140544	1.1277251	16	0.8974561	9791
ADHERENS JUNCTION ORGANIZATION%GOBP%GO:0034332	0.45394692	1.5141058	67	0.8974905	7220
TNF RELATED WEAK INDUCER OF APOPTOSIS (TWEAK) SIGNALING PATHWAY%WIKIPATHWAYS_20210110%WP2036%HOMO SAPIENS	0.35670108	1.1273265	33	0.89772075	11221
SIGNAL TRANSDUCTION INVOLVED IN MITOTIC G1 DNA DAMAGE CHECKPOINT%GOBP%GO:0072431	0.40771866	1.3436941	56	0.8977498	6040
NEGATIVE REGULATION OF MRNA PROCESSING%GOBP%GO:0050686	0.44961038	1.3442287	24	0.8978609	7848
POSITIVE REGULATION OF PHOSPHOLIPASE ACTIVITY%GOBP%GO:0010518	0.35275477	1.1273415	41	0.8981418	12347
TRNA SPLICING, VIA ENDONUCLEOLYTIC CLEAVAGE AND LIGATION%GOBP%GO:0006388	0.49442622	1.3433565	15	0.8981463	3959
NEUTRAL AMINO ACID TRANSPORT%GOBP%GO:0015804	0.4508864	1.3444571	26	0.89823884	7936
NICOTINAMIDE NUCLEOTIDE METABOLIC PROCESS%GOBP%GO:0046496	0.4524008	1.5433139	90	0.8982992	4362
ASSEMBLY OF THE HIV VIRION%REACTOME DATABASE ID RELEASE 75%175474	0.48693258	1.3438773	16	0.89832103	9885
REGULATION OF CHEMOTAXIS%GOBP%GO:0050920	0.31713164	1.1270511	154	0.8984438	11000
E2F TRANSCRIPTION FACTOR NETWORK%PATHWAY INTERACTION DATABASE NCI-NATURE CURATED DATA%E2F TRANSCRIPTION FACTOR NETWORK	0.4494399	1.5166047	70	0.8988744	4837
POSITIVE REGULATION OF LIPASE ACTIVITY%GOBP%GO:0060193	0.34662303	1.1267188	47	0.89894605	12347
DNA DAMAGE RESPONSE, SIGNAL TRANSDUCTION BY P53 CLASS MEDIATOR RESULTING IN CELL CYCLE ARREST%GOBP%GO:0006977	0.40628162	1.3446285	55	0.8989734	3710
NEGATIVE REGULATION OF PHOSPHATASE ACTIVITY%GOBP%GO:0010923	0.33847085	1.1268137	68	0.8990136	4252
PATHWAY%GOBP%GO:1901030	0.48741573	1.517452	32	0.89916354	4030
GOLGI ORGANIZATION%GOBP%GO:0007030	0.32513422	1.1235565	121	0.89921075	3383
PLATELET ACTIVATION%GOBP%GO:0030168	0.32982993	1.1234252	89	0.8993051	5295
RHO GTPASE CYCLE%REACTOME%R-HSA-194840.5	0.32184514	1.1236345	146	0.89935833	4957
ANTIGEN PROCESSING AND PRESENTATION%GOBP%GO:0019882	0.3188449	1.1237371	187	0.8993927	8748
SYNTHESIS OF PIPS AT THE PLASMA MEMBRANE%REACTOME%R-HSA-1660499.6	0.3464588	1.1253923	49	0.89941907	6047
HEART DEVELOPMENT%WIKIPATHWAYS_20210110%WP1591%HOMO SAPIENS	0.3647679	1.1230594	33	0.899427	9489
REGULATION OF INTERLEUKIN-2 PRODUCTION%GOBP%GO:0032663	0.43148366	1.344828	33	0.8996248	6189
NICOTINAMIDE NUCLEOTIDE BIOSYNTHETIC PROCESS%GOBP%GO:0019359	0.3426295	1.1231246	52	0.8996353	4362
GASTRIN_CCK2R_240212%PANTHER PATHWAY%P06959	0.32436576	1.125104	143	0.89970356	4523
POSITIVE REGULATION OF CELLULAR PROTEIN CATABOLIC PROCESS%GOBP%GO:1903364	0.3248705	1.1237423	122	0.8998596	7254
TNF SIGNALING%REACTOME%R-HSA-75893.5	0.35280395	1.1255109	41	0.8998867	10765
STIMULI-SENSING CHANNELS%REACTOME%R-HSA-2672351.4	0.3346068	1.1253961	79	0.8998923	5835
TOLL LIKE RECEPTOR 2 (TLR2) CASCADE%REACTOME%R-HSA-181438.1	0.3267905	1.1249341	89	0.8999742	10539
COOPERATION OF PDCL (PHLP1) AND TRIC CCT IN G-PROTEIN BETA FOLDING%REACTOME DATABASE ID RELEASE 75%6814122	0.35390264	1.1255976	39	0.8999907	9374
MRNA DECAY BY 5' TO 3' EXORIBONUCLEASE%REACTOME%R-HSA-430039.1	0.4042166	1.1228125	15	0.90000224	9567
ANTIGEN PROCESSING AND PRESENTATION OF PEPTIDE OR POLYSACCHARIDE ANTIGEN VIA MHC CLASS II%GOBP%GO:0002504	0.33301568	1.1263568	91	0.900038	9094
INFLAMMATORY RESPONSE%GOBP%GO:0006954	0.31636423	1.1251363	178	0.90004647	6810
METAPHASE PLATE CONGRESSION%GOBP%GO:0051310	0.34103832	1.124702	58	0.9000519	9477
NOTCH SIGNALING PATHWAY%GOBP%GO:0007219	0.34522304	1.1256963	56	0.90005875	7480
CELLULAR RESPONSE TO NUTRIENT%GOBP%GO:0031670	0.36277932	1.126231	32	0.9000892	564
COAGULATION%GOBP%GO:0050817	0.315667	1.12313	190	0.9000978	4862
ASSOCIATIVE LEARNING%GOBP%GO:0008306	0.40034696	1.1245781	18	0.9001527	3734
GASTRIN SIGNALING PATHWAY%WIKIPATHWAYS_20210110%WP4659%HOMO SAPIENS	0.32986286	1.1237764	94	0.9001778	11345
RECEPTOR-MEDIATED ENDOCYTOSIS%GOBP%GO:0006898	0.31924632	1.1238806	130	0.90022963	8901
INTERLEUKIN-1-MEDIATED SIGNALING PATHWAY%GOBP%GO:0070498	0.331152	1.1260942	91	0.900242	5609
EMBRYONIC CAMERA-TYPE EYE DEVELOPMENT%GOBP%GO:0031076	0.41594014	1.12573	16	0.9003877	6191
NEGATIVE REGULATION OF PROTEIN TRANSPORT%GOBP%GO:0051224	0.33216664	1.1247326	76	0.9004156	3691
ODONTOGENESIS%GOBP%GO:0042476	0.34506235	1.1224859	44	0.90046316	6752
PURINE RIBONUCLEOSIDE DIPHOSPHATE METABOLIC PROCESS%GOBP%GO:0009179	0.35820696	1.1209527	36	0.90047216	4362
DEOXYRIBONUCLEOTIDE CATABOLIC PROCESS%GOBP%GO:0009264	0.3881799	1.1206133	18	0.9004735	5891
CELL CYCLE%WIKIPATHWAYS_20210110%WP179%HOMO SAPIENS	0.33019873	1.1258199	84	0.9004756	6057
COPI-INDEPENDENT GOLGI-TO-ER RETROGRADE TRAFFIC%REACTOME DATABASE ID RELEASE 75%6811436	0.3550654	1.1225785	33	0.9005336	3142
PEPTIDYL-CYSTEINE MODIFICATION%GOBP%GO:0018198	0.35812098	1.1219126	33	0.9005602	5507
PEPTIDE CATABOLIC PROCESS%GOBP%GO:0043171	0.44639084	1.3449684	25	0.9006206	6874
SMAD2 SMAD3:SMAD4 HETEROTRIMER REGULATES TRANSCRIPTION%REACTOME DATABASE ID RELEASE 75%2173796	0.36517745	1.1238949	31	0.90065753	5648
REGULATION OF VENTRICULAR CARDIAC MUSCLE CELL ACTION POTENTIAL%GOBP%GO:0098911	0.5631536	1.5366734	15	0.9007392	2769

CELLULAR RESPONSE TO IONIZING RADIATION%GOBP%GO:0071479	0.34956267	1.1219798	46	0.900746	6927
ANAPHASE-PROMOTING COMPLEX-DEPENDENT CATABOLIC PROCESS%GOBP%GO:0031145	0.33184734	1.1209961	80	0.9007554	9584
NEGATIVE REGULATION OF CELL CYCLE PHASE TRANSITION%GOBP%GO:1901988	0.31199396	1.123974	207	0.9007947	6057
GENITALIA DEVELOPMENT%GOBP%GO:0048806	0.40991402	1.12042	15	0.900852	7031
ESTABLISHMENT OF PROTEIN LOCALIZATION TO VACUOLE%GOBP%GO:0072666	0.34758717	1.1258343	48	0.90090555	12450
BLOOD COAGULATION%GOBP%GO:0007596	0.315667	1.1212778	190	0.90090835	4862
MULTI-ORGANISM LOCALIZATION%GOBP%GO:1902579	0.33382908	1.1206222	67	0.90091646	7794
POSITIVE REGULATION OF CHROMOSOME SEPARATION%GOBP%GO:1905820	0.4064818	1.1221516	16	0.9009651	6680
VALIDATED TRANSCRIPTIONAL TARGETS OF TAP63 ISOFORMS%PATHWAY INTERACTION DATABASE NCI-NATURE CURATED DATA%VALIDATED TRANSCRIPTIONAL TARGETS OF TAP63 ISOFORMS	0.46428928	1.5397078	51	0.9009744	3536
PID_EPHA2_FWD_PATHWAY%MSIGDB_C2%PID_EPHA2_FWD_PATHWAY	0.40310648	1.122039	15	0.90097624	6134
NEGATIVE REGULATION OF PROTEIN CATABOLIC PROCESS%GOBP%GO:0042177	0.32557818	1.1210517	103	0.9010013	5005
AUTOPHAGY OF MITOCHONDRION%GOBP%GO:0000422	0.34890968	1.1150819	38	0.90103495	6527
ABC TRANSPORTER DISORDERS%REACTOME DATABASE ID RELEASE 75%5619084	0.33274633	1.1240157	71	0.9010977	8770
MITOCHONDRIAL CALCIUM ION TRANSPORT%REACTOME DATABASE ID RELEASE 75%8949215	0.37840226	1.115181	23	0.9011046	9285
EPIDERMAL CELL DIFFERENTIATION%GOBP%GO:0009913	0.32557186	1.113845	83	0.9011832	5155
DNA DAMAGE RECOGNITION IN GG-NER%REACTOME DATABASE ID RELEASE 75%5696394	0.35044524	1.1222109	38	0.9011956	5766
LYMPHOCYTE DIFFERENTIATION%GOBP%GO:0030098	0.30974305	1.0513757	89	0.9011962	6299
GENE SILENCING BY RNA%REACTOME%R-HSA-211000.2	0.3298299	1.1213177	85	0.90121895	5533
P38 MAPK SIGNALING PATHWAY%PATHWAY INTERACTION DATABASE NCI-NATURE CURATED DATA%P38 MAPK SIGNALING PATHWAY	0.34732205	1.047846	27	0.9012221	12418
RESPONSE TO GLUCOCORTICOID%GOBP%GO:0051384	0.36959517	1.120112	25	0.9012227	9383
IL1-MEDIATED SIGNALING EVENTS%PATHWAY INTERACTION DATABASE NCI-NATURE CURATED DATA%IL1-MEDIATED SIGNALING EVENTS	0.47989395	1.5154513	34	0.9012515	4048
POSITIVE REGULATION OF VIRAL TRANSCRIPTION%GOBP%GO:0050434	0.3540894	1.1187727	39	0.90125734	6975
POSITIVE REGULATION OF CD4-POSITIVE, ALPHA-BETA T CELL ACTIVATION%GOBP%GO:2000516	0.38682744	1.1206524	21	0.90126395	1524
TUMOR NECROSIS FACTOR-MEDIATED SIGNALING PATHWAY%GOBP%GO:0033209	0.32290295	1.1153553	100	0.9012819	8211
CYTOSOLIC SULFONATION OF SMALL MOLECULES%REACTOME DATABASE ID RELEASE 75%156584	0.39373454	1.1214148	18	0.9012983	4260
CARBOXYLIC ACID TRANSPORT%GOBP%GO:0046942	0.29351768	1.0464457	191	0.9013023	8680
PROTEIN LOCALIZATION TO GOLGI APPARATUS%GOBP%GO:0034067	0.37360242	1.1241866	25	0.90130633	6430
INOSITOL LIPID-MEDIATED SIGNALING%GOBP%GO:0048017	0.33295915	1.1240759	67	0.90131235	5543
DOWNSTREAM SIGNALING OF ACTIVATED FGFR1%REACTOME DATABASE ID RELEASE 75%5654687	0.3611219	1.0381742	18	0.90131825	4614
FORMATION OF INCISION COMPLEX IN GG-NER%REACTOME%R-HSA-5696395.1	0.35199952	1.1149129	42	0.90132385	6763
EMBRYONIC MORPHOGENESIS%GOBP%GO:0048598	0.29109555	1.0479085	248	0.90132993	7112
POSITIVE REGULATION OF STEM CELL PROLIFERATION%GOBP%GO:2000648	0.3495217	1.0462364	24	0.90134895	6438
REGULATION OF G1/S TRANSITION OF MITOTIC CELL CYCLE%GOBP%GO:2000045	0.32168785	1.1154413	126	0.9013679	6299
FCGR3A-MEDIATED PHAGOCYTOSIS%REACTOME%R-HSA-9664422.1	0.34043595	1.118635	50	0.90137225	6264
HOST-PATHOGEN INTERACTION OF HUMAN CORONA VIRUSES - MAPK SIGNALING%WIKIPATHWAYS_20210110%WP4877%HOMO SAPIENS	0.341688	1.0465205	32	0.9013746	10650
MALE GONAD DEVELOPMENT%GOBP%GO:0008584	0.35795978	1.1210717	35	0.9013992	8945
CELLULAR RESPONSE TO INTERLEUKIN-1%GOBP%GO:0071347	0.31755883	1.1188521	133	0.9014021	5609
GLYCOSYL COMPOUND CATABOLIC PROCESS%GOBP%GO:1901658	0.36037552	1.115218	32	0.90141386	4806
NEGATIVE REGULATION OF SECRETION BY CELL%GOBP%GO:1903531	0.3082832	1.0380418	74	0.90145826	11502
REGULATION OF BONE RESORPTION%GOBP%GO:0045124	0.38066927	1.1138873	21	0.9014655	3
CELLULAR RESPONSE TO CALCIUM ION%GOBP%GO:0071277	0.45280734	1.5144854	62	0.9014793	5759
LONG-CHAIN FATTY ACID TRANSPORT%GOBP%GO:0015909	0.33192748	1.0466768	39	0.90150344	8647
REGULATION OF INTRACELLULAR STEROID HORMONE RECEPTOR SIGNALING PATHWAY%GOBP%GO:0033143	0.31571314	1.0462878	63	0.90151364	6055
EMBRYONIC SKELETAL SYSTEM DEVELOPMENT%GOBP%GO:0048706	0.32603163	1.0513798	48	0.90153474	7143
NEGATIVE REGULATION OF PEPTIDE SECRETION%GOBP%GO:0002792	0.3264542	1.0465703	40	0.9015388	11345
MACROAUTOPHAGY%GOBP%GO:0016236	0.3153605	1.1183777	156	0.90154594	8098
CELL CYCLE G2/M PHASE TRANSITION%GOBP%GO:0044839	0.31837475	1.1201438	134	0.90157217	7225
POSITIVE REGULATION OF B CELL MEDIATED IMMUNITY%GOBP%GO:0002714	0.39469635	1.1215762	18	0.90159184	6556
HISTONE H3 DEACETYLATION%GOBP%GO:0070932	0.38324007	1.0479306	15	0.9015967	5402
RESPIRATORY ELECTRON TRANSPORT%REACTOME%R-HSA-611105.3	0.32352322	1.1190335	101	0.9015983	6736
REGULATORY CIRCUITS OF THE STAT3 SIGNALING PATHWAY%WIKIPATHWAYS_20210110%WP4538%HOMO SAPIENS	0.31498957	1.0446988	57	0.90160626	10735
REGULATION OF ENDOTHELIAL CELL MIGRATION%GOBP%GO:0010594	0.3167117	1.1195796	137	0.9016175	9790
NRF2 PATHWAY%WIKIPATHWAYS_20210110%WP2884%HOMO SAPIENS	0.30962738	1.0468432	77	0.90162	4905
RECEPTOR CATABOLIC PROCESS%GOBP%GO:0032801	0.3551916	1.0511503	24	0.9016405	9205
AURORA A SIGNALING%PATHWAY INTERACTION DATABASE NCI-NATURE CURATED DATA%AURORA A SIGNALING	0.3340042	1.0381782	30	0.9016427	7522
NEPHRON EPITHELIUM DEVELOPMENT%GOBP%GO:0072009	0.3213393	1.0497729	52	0.9016591	7094
NEGATIVE REGULATION OF FAT CELL DIFFERENTIATION%GOBP%GO:0045599	0.3538932	1.1196787	33	0.9016651	4781
PHOSPHATIDYLINOSITOL BIOSYNTHETIC PROCESS%GOBP%GO:0006661	0.3238248	1.113952	102	0.9016663	8404
GLUTATHIONE CONJUGATION%REACTOME DATABASE ID RELEASE 75%156590	0.33511657	1.0460465	32	0.9016745	5007
COLLAGEN CATABOLIC PROCESS%GOBP%GO:0030574	0.3325519	1.0388451	29	0.90169156	4002
RESPONSE TO COLD%GOBP%GO:0009409	0.3706113	1.0479991	17	0.9016992	13025
AORTA DEVELOPMENT%GOBP%GO:0035904	0.34660336	1.0459379	24	0.90171236	4346
HALLMARK_TNFA_SIGNALING_VIA_NFKB%MSIGDB_C2%HALLMARK_TNFA_SIGNALING_VIA_NFKB	0.29869813	1.0508345	151	0.90171516	5434
REGULATION OF TRANSCRIPTION BY RNA POLYMERASE III%GOBP%GO:0006359	0.38243082	1.1131805	22	0.9017173	5953
MAINTENANCE OF PROTEIN LOCATION IN CELL%GOBP%GO:0032507	0.31109196	1.0387411	56	0.9017236	6838
TISSUE HOMEOSTASIS%GOBP%GO:0001894	0.30580035	1.0510238	84	0.90174025	6910
REGULATION OF TRANSCRIPTION INITIATION FROM RNA POLYMERASE II PROMOTER%GOBP%GO:0060260	0.37172788	1.1188841	25	0.90174145	6040
G BETA:GAMMA SIGNALLING THROUGH CDC42%REACTOME DATABASE ID RELEASE 75%8964616	0.464473	1.3450804	19	0.90174925	6094
BIOCARTA_SPRY_PATHWAY%MSIGDB_C2%BIOCARTA_SPRY_PATHWAY	0.37832078	1.0458297	15	0.90175605	9509
GDNF%IOB%GDNF	0.34226713	1.0469944	26	0.9017626	4202
DNA DUPLEX UNWINDING%GOBP%GO:0032508	0.32097757	1.0514106	50	0.9017659	6153
NUCLEOTIDE-EXCISION REPAIR, DNA GAP FILLING%GOBP%GO:0006297	0.374779	1.1214188	24	0.9017669	3931
REGULATION OF VESICLE FUSION%GOBP%GO:0031338	0.39564183	1.1197617	18	0.901773	6153
REGULATION OF DEFENSE RESPONSE TO VIRUS%GOBP%GO:0050688	0.31743035	1.0503194	56	0.90179795	6496
TRANSCRIPTIONAL ACTIVITY OF SMAD2 SMAD3:SMAD4 HETEROTRIMER%REACTOME DATABASE ID RELEASE 75%2173793	0.32571158	1.0382293	43	0.90180683	10869
ANGIOTENSIN_II-STIMULATED_SIGNALING_THROUGH_G_PROTEINS_AND_BETA-ARRESTIN%PANTHER PATHWAY%P05911	0.35967627	1.1190934	36	0.90181005	7719
REGULATION OF PROTEOLYSIS INVOLVED IN CELLULAR PROTEIN CATABOLIC PROCESS%GOBP%GO:1903050	0.31347623	1.1154466	186	0.9018123	6536
ASSEMBLY OF THE PRE-REPLICATIVE COMPLEX%REACTOME DATABASE ID RELEASE 75%68867	0.3129549	1.0447377	65	0.901815	8770
DECTIN-1 MEDIATED NONCANONICAL NF-KB SIGNALING%REACTOME%R-HSA-5607761.1	0.31901425	1.049826	58	0.9018165	8770
TRNA AMINOACYLATION FOR PROTEIN TRANSLATION%GOBP%GO:0006418	0.32610446	1.0466859	43	0.9018203	8624
REGULATION OF TOLL-LIKE RECEPTOR SIGNALING PATHWAY%GOBP%GO:0034121	0.32404587	1.0502118	49	0.90182143	12849
BMP SIGNALING PATHWAY IN EYELID DEVELOPMENT%WIKIPATHWAYS_20210110%WP3927%HOMO SAPIENS	0.399082	1.1146823	17	0.9018335	10856
SULFUR AMINO ACID BIOSYNTHETIC PROCESS%GOBP%GO:0000097	0.3793822	1.0450245	15	0.9018474	6420
REGULATION OF PROTEIN LOCALIZATION TO MEMBRANE%GOBP%GO:1905475	0.31693643	1.1184123	151	0.9018681	5549
REGULATION OF HEMATOPOIETIC PROGENITOR CELL DIFFERENTIATION%GOBP%GO:1901532	0.30860564	1.0473639	81	0.90187687	9483
PID_ANGIOPOIETIN_RECEPTOR_PATHWAY%MSIGDB_C2%PID_ANGIOPOIETIN_RECEPTOR_PATHWAY	0.33580074	1.0503914	32	0.901883	4607
FGF SIGNALING PATHWAY%PATHWAY INTERACTION DATABASE NCI-NATURE CURATED DATA%FGF SIGNALING PATHWAY	0.33318993	1.0449166	42	0.90188885	4179
NEUROTRANSMITTER RECEPTOR TRANSPORT%GOBP%GO:0099637	0.36733976	1.0451112	19	0.9018985	12135
RECRUITMENT OF NUMA TO MITOTIC CENTROSOMES%REACTOME%R-HSA-380320.2	0.31170547	1.0508825	79	0.9019025	9743
SOMATIC CELL DNA RECOMBINATION%GOBP%GO:0016444	0.5366421	1.5343845	18	0.9019096	6061
BIOCARTA_FAS_PATHWAY%MSIGDB_C2%BIOCARTA_FAS_PATHWAY	0.38884467	1.1157395	21	0.90194535	5356
G2 DNA DAMAGE CHECKPOINT%GOBP%GO:0031572	0.33545092	1.0468456	30	0.9019589	8659
SIGNALING BY HIPPO%REACTOME%R-HSA-2028269.1	0.3795865	1.1139952	19	0.9019594	4245
NEGATIVE REGULATION OF LOCOMOTION%GOBP%GO:0040013	0.29154354	1.0480237	242	0.9019698	8463
REGULATION OF LEUKOCYTE MEDIATED CYTOTOXICITY%GOBP%GO:0001910	0.3389365	1.0470303	32	0.9019737	4842
POSITIVE REGULATION OF G1/S TRANSITION OF MITOTIC CELL CYCLE%GOBP%GO:1900087	0.34384295	1.0498762	26	0.9019882	7256
VITAMIN B12 METABOLISM%WIKIPATHWAYS_20210110%WP1533%HOMO SAPIENS	0.3387201	1.0500629	31	0.90200406	13130
EMBRYONIC ORGAN DEVELOPMENT%GOBP%GO:0048568	0.29625037	1.0475231	180	0.9020078	7626
POSITIVE REGULATION OF NEURON DIFFERENTIATION%GOBP%GO:0045666	0.3100145	1.1198165	211	0.902008	8439
TNFALPHA%IOB%TNFALPHA	0.29570916	1.049965	157	0.90201735	5626
IFN-GAMMA PATHWAY%PATHWAY INTERACTION DATABASE NCI-NATURE CURATED DATA%IFN-GAMMA PATHWAY	0.32492325	1.0388471	40	0.9020206	2226
CELL SURFACE INTERACTIONS AT THE VASCULAR WALL%REACTOME DATABASE ID RELEASE 75%202733	0.30824757	1.0505506	89	0.90202856	5573
PID_AURORA_B_PATHWAY%MSIGDB_C2%PID_AURORA_B_PATHWAY	0.3860925	1.1158292	20	0.9020316	5464
PID_MYC_REPRESS_PATHWAY%MSIGDB_C2%PID_MYC_REPRESS_PATHWAY	0.3169661	1.0444827	49	0.9020326	5717
NEURAL TUBE FORMATION%GOBP%GO:0001841	0.32710597	1.0389396	39	0.90203273	6318
TOLL-LIKE RECEPTOR CASCADES%REACTOME DATABASE ID RELEASE 75%168898	0.30037102	1.0504394	131	0.90205747	5640
PHAGOCYTOSIS%GOBP%GO:0006909	0.29464826	1.0382558	142	0.9020624	7411
GLOBAL GENOME NUCLEOTIDE EXCISION REPAIR (GG-NER)%REACTOME DATABASE ID RELEASE 75%5696399	0.31159896	1.0514233	83	0.90207314	6763
INSULIN IGF PATHWAY-PROTEIN KINASE B SIGNALING CASCADE%PANTHER PATHWAY%P00033	0.37826666	1.1132042	22	0.90207577	11932
STATIN PATHWAY%WIKIPATHWAYS_20210110%WP430%HOMO SAPIENS	0.3792121	1.0452541	15	0.9020847	13809
OXYGEN-DEPENDENT PROLINE HYDROXYLATION OF HYPOXIA-INDUCIBLE FACTOR ALPHA%REACTOME%R-HSA-1234176.2	0.31427342	1.0412645	64	0.90208876	8770
INTRACELLULAR TRANSPORT OF VIRUS%GOBP%GO:0075733	0.32528788	1.044757	50	0.9020946	8514
ER TO GOLGI ANTEROGRADE TRANSPORT%REACTOME DATABASE ID RELEASE 75%199977	0.30037215	1.0480793	132	0.9021079	8320
ENDOTHELIAL CELL DIFFERENTIATION%GOBP%GO:0045446	0.33281183	1.1135204	59	0.90212035	3763
NEGATIVE REGULATION OF RNA SPLICING%GOBP%GO:0033119	0.39398128	1.1191274	19	0.9021292	4952
NEGATIVE REGULATION OF NOTCH4 SIGNALING%REACTOME%R-HSA-9604323.1	0.3393723	1.11548	53	0.9021364	8913
NEGATIVE REGULATION OF CELL JUNCTION ASSEMBLY%GOBP%GO:1901889	0.3527146	1.0384251	21	0.90214133	2901
REGULATION OF MICROTUBULE-BASED MOVEMENT%GOBP%GO:0060632	0.34206718	1.0473835	26	0.90215826	6910
IMMUNOGLOBULIN MEDIATED IMMUNE RESPONSE%GOBP%GO:0016064	0.35901964	1.0451349	23	0.9021626	6379
STEROID METABOLIC PROCESS%GOBP%GO:0008202	0.32365653	1.1192259	138	0.90217084	5269
L-AMINO ACID TRANSPORT%GOBP%GO:0015807	0.35867667	1.1140534	33	0.9021709	8680
REGULATION OF LEUKOCYTE CHEMOTAXIS%GOBP%GO:0002688	0.30900523	1.0481614	84	0.90217495	11000
INTERACTOME OF POLYCOMB REPRESSIVE COMPLEX 2 (PRC2) %WIKIPATHWAYS_20210110%WP2916%HOMO SAPIENS	0.3854772	1.0544492	15	0.90217555	3418
CELLULAR RESPONSE TO GLUCOCORTICOID STIMULUS%GOBP%GO:0071385	0.40029773	1.1165532	16	0.90218306	9383
FATTY ACID OXIDATION%GOBP%GO:0019395	0.3334413	1.1132932	61	0.90218884	8900
REGULATION OF RESPONSE TO WOUNDING%GOBP%GO:1903034	0.32160872	1.1164371	113	0.90219855	2064

PROTEIN TARGETING TO VACUOLE%GOBP%GO:0006623	0.3333833	1.041524	37	0.9022034	12297
WNT SIGNALING PATHWAY (NETPATH)%WIKIPATHWAYS_20210110%WP363%HOMO SAPIENS	0.32736534	1.04541	42	0.90222126	10979
ORGANIC ACID TRANSPORT%GOBP%GO:0015849	0.2908339	1.0383075	192	0.90222204	8680
POSITIVE REGULATION OF ADAPTIVE IMMUNE RESPONSE%GOBP%GO:0002821	0.31702435	1.0417006	58	0.9022728	6556
EPITHELIAL CELL DEVELOPMENT%GOBP%GO:0002064	0.3087069	1.0505792	82	0.90227526	7094
NEURAL CREST DIFFERENTIATION%WIKIPATHWAYS_20210110%WP2064%HOMO SAPIENS	0.32473058	1.1127229	81	0.90229195	7491
ORGANELLE MEMBRANE FUSION%GOBP%GO:0090174	0.3112038	1.0470362	74	0.90230477	11256
CARDIAC MUSCLE TISSUE DEVELOPMENT%GOBP%GO:0048738	0.31062767	1.0412996	76	0.90231526	7588
FORMATION OF TUBULIN FOLDING INTERMEDIATES BY CCT TRIC%REACTOME%R-HSA-389960.2	0.3705672	1.053231	19	0.9023159	5924
INTEGRIN SIGNALLING PATHWAY%PANTHER PATHWAY%P00034	0.31579393	1.114129	165	0.9023332	5922
GLYCOPROTEIN BIOSYNTHETIC PROCESS%GOBP%GO:0009101	0.29119942	1.0489092	241	0.9023359	6418
CELL-CELL SIGNALING INVOLVED IN CARDIAC CONDUCTION%GOBP%GO:0086019	0.34942713	1.0389423	22	0.9023613	6324
NEDDYLATION%REACTOME%R-HSA-8951664.2	0.29438195	1.0539215	219	0.9023639	5498
NEGATIVE REGULATION OF CANONICAL WNT SIGNALING PATHWAY%GOBP%GO:0090090	0.29557306	1.0455655	153	0.902365	7219
PROCESSING OF CAPPED INTRON-CONTAINING PRE-MRNA%REACTOME%R-HSA-72203.3	0.29207304	1.0454632	237	0.9023747	7238
DEFECTIVE CFTR CAUSES CYSTIC FIBROSIS%REACTOME%R-HSA-5678895.3	0.31787577	1.0384545	59	0.90237665	8770
POSITIVE REGULATION OF PATHWAY-RESTRICTED SMAD PROTEIN PHOSPHORYLATION%GOBP%GO:0010862	0.35049498	1.1155301	40	0.9023891	6752
NEGATIVE REGULATION OF ANIMAL ORGAN MORPHOGENESIS%GOBP%GO:0110111	0.4006262	1.1133534	17	0.9023943	6228
REGULATION OF SYSTEMIC ARTERIAL BLOOD PRESSURE MEDIATED BY A CHEMICAL SIGNAL%GOBP%GO:0003044	0.35457635	1.0544862	25	0.9024002	10116
EPITHELIAL CELL DIFFERENTIATION INVOLVED IN KIDNEY DEVELOPMENT%GOBP%GO:0035850	0.35651854	1.0452603	23	0.9024079	6631
NEGATIVE REGULATION OF VIRAL TRANSCRIPTION%GOBP%GO:0032897	0.35653913	1.0514283	22	0.9024104	11261
LATE ENDOSOME TO VACUOLE TRANSPORT%GOBP%GO:0045324	0.3764129	1.1158433	23	0.90243334	8586
VESICLE-MEDIATED TRANSPORT BETWEEN ENDOSOMAL COMPARTMENTS%GOBP%GO:0098927	0.3638406	1.049174	23	0.90244335	13009
75%8939243	0.33089352	1.0415399	36	0.90249074	1831
EPIDERMIS DEVELOPMENT%GOBP%GO:0008544	0.30205593	1.053976	123	0.9025083	7524
REGULATION OF DNA DAMAGE RESPONSE, SIGNAL TRANSDUCTION BY P53 CLASS MEDIATOR%GOBP%GO:0043516	0.34108055	1.0413418	30	0.902509	4867
ARYL HYDROCARBON RECEPTOR PATHWAY%WIKIPATHWAYS_20210110%WP2873%HOMO SAPIENS	0.36249632	1.1141952	29	0.9025206	4701
COOPERATION OF PREFOLDIN AND TRIC CCT IN ACTIN AND TUBULIN FOLDING%REACTOME%R-HSA-389958.2	0.346049	1.0470735	26	0.90252274	5924
LYSOSOMAL TRANSPORT%GOBP%GO:0007041	0.30477363	1.048162	99	0.9025235	11894
MUSCARINIC ACETYLCHOLINE RECEPTOR 1 AND 3 SIGNALING PATHWAY%PANTHER PATHWAY%P00042	0.31772396	1.0389887	52	0.9025305	11324
SARS-COV-2 INFECTION%REACTOME DATABASE ID RELEASE 75%9694516	0.31752652	1.0540677	65	0.9025343	8585
P73 TRANSCRIPTION FACTOR NETWORK%PATHWAY INTERACTION DATABASE NCI-NATURE CURATED DATA%P73 TRANSCRIPTION FACTOR NETWORK	0.31346536	1.0541604	75	0.9025377	4153
POSITIVE REGULATION OF HISTONE METHYLATION%GOBP%GO:0031062	0.34319797	1.0532658	27	0.90255064	5917
CELLULAR RESPONSE TO GROWTH HORMONE STIMULUS%GOBP%GO:0071378	0.37500983	1.049043	15	0.90256935	6812
FGFR2 ALTERNATIVE SPLICING%REACTOME DATABASE ID RELEASE 75%6803529	0.36746556	1.1192442	26	0.902573	7470
ASSOCIATION OF TRIC CCT WITH TARGET PROTEINS DURING BIOSYNTHESIS%REACTOME DATABASE ID RELEASE 75%390471	0.32943216	1.0417054	35	0.90259373	6228
TRANSCRIPTIONAL REGULATION BY SMALL RNAS%REACTOME%R-HSA-5578749.3	0.3104063	1.0419009	63	0.90259415	5533
GAMETE GENERATION%GOBP%GO:0007276	0.30950525	1.11276	220	0.90260094	5296
PROTEIN DEACETYLATION%GOBP%GO:0006476	0.32424656	1.0542315	51	0.90263754	3962
NEGATIVE REGULATION OF PROTEIN MODIFICATION BY SMALL PROTEIN CONJUGATION OR REMOVAL%GOBP%GO:1903321	0.30810854	1.0390545	72	0.90263754	10010
NGF-STIMULATED TRANSCRIPTION%REACTOME DATABASE ID RELEASE 75%9031628	0.33185127	1.0489239	37	0.90264153	9850
REGULATION OF TRANSPOSITION%GOBP%GO:0010528	0.38581404	1.1165566	19	0.9026432	1148
G-PROTEIN ACTIVATION%REACTOME DATABASE ID RELEASE 75%202040	0.3564599	1.0514613	26	0.90264964	9314
OSTEOCLAST DIFFERENTIATION%GOBP%GO:0030316	0.39735675	1.1158978	17	0.9026567	2032
NEGATIVE REGULATION OF FGFR4 SIGNALING%REACTOME DATABASE ID RELEASE 75%5654733	0.35755986	1.0526443	22	0.902658	7222
PARASITE INFECTION%REACTOME%R-HSA-9664407.1	0.34043595	1.1161107	50	0.9026868	6264
PEPTIDE HORMONE PROCESSING%GOBP%GO:0016486	0.35803008	1.049196	23	0.9027162	10009
SULFUR COMPOUND CATABOLIC PROCESS%GOBP%GO:0044273	0.41405365	1.3463637	46	0.90272367	6312
PULMONARY VALVE MORPHOGENESIS%GOBP%GO:0003184	0.38167408	1.049289	15	0.9027272	14468
CELL-SUBSTRATE ADHERENS JUNCTION ASSEMBLY%GOBP%GO:0007045	0.5293068	1.5188043	20	0.9027297	6547
HALLMARK_PI3K_AKT_MTOR_SIGNALING%MSIGDB_C2%HALLMARK_PI3K_AKT_MTOR_SIGNALING	0.30887723	1.0428269	76	0.90273476	8620
CELL DIFFERENTIATION INVOLVED IN KIDNEY DEVELOPMENT%GOBP%GO:0061005	0.34501714	1.052427	28	0.90274	7542
CELLULAR AMINO ACID BIOSYNTHETIC PROCESS%GOBP%GO:0008652	0.31483266	1.0544906	65	0.90274566	2905
REGULATION OF MACROAUTOPHAGY%GOBP%GO:0016241	0.31546357	1.1162043	154	0.9027538	8531
ION CHANNEL TRANSPORT%REACTOME DATABASE ID RELEASE 75%983712	0.29920834	1.0481924	149	0.90276	5942
OSTEOBLAST DIFFERENTIATION%GOBP%GO:0001649	0.32105	1.0636071	72	0.9027665	5634
RESPONSE TO OXYGEN LEVELS%GOBP%GO:0070482	0.2924537	1.041756	202	0.9027687	6322
CALCIUM ION TRANSMEMBRANE TRANSPORT%GOBP%GO:0070588	0.32589942	1.1124932	91	0.9027813	3251
LATE ENDOSOME TO VACUOLE TRANSPORT VIA MULTIVESICULAR BODY SORTING PATHWAY%GOBP%GO:0032511	0.37634996	1.0528078	16	0.9027863	8586
PHOTODYNAMIC THERAPY-INDUCED AP-1 SURVIVAL SIGNALING.%WIKIPATHWAYS_20210110%WP3611%HOMO SAPIENS	0.32867473	1.0426191	37	0.90278983	10095
TRANS-SULFURATION AND ONE CARBON METABOLISM%WIKIPATHWAYS_20210110%WP2525%HOMO SAPIENS	0.3684888	1.0515182	20	0.902804	4845
ATP BIOSYNTHETIC PROCESS%GOBP%GO:0006754	0.31574196	1.0395865	57	0.9028041	7952
PHOSPHOLIPID BIOSYNTHETIC PROCESS%GOBP%GO:0008654	0.29035416	1.0390933	217	0.90284413	8404
PROGRAMMED CELL DEATH%REACTOME DATABASE ID RELEASE 75%5357801	0.31625432	1.1166213	175	0.9028533	9054
MULTICELLULAR ORGANISMAL MOVEMENT%GOBP%GO:0050879	0.36035708	1.042017	21	0.9028581	7419
UROGENITAL SYSTEM DEVELOPMENT%GOBP%GO:0001655	0.29703805	1.0419221	139	0.90285915	7728
REGULATION OF PROTEIN IMPORT%GOBP%GO:1904589	0.33405066	1.0526875	37	0.9028713	12680
NUCLEAR DIVISION%GOBP%GO:0000280	0.2929716	1.0391839	215	0.9028716	10445
MITOTIC G2-G2 M PHASES%REACTOME%R-HSA-453274.2	0.3123271	1.1123604	181	0.9028729	9513
RAS SIGNALING%WIKIPATHWAYS_20210110%WP4223%HOMO SAPIENS	0.30123964	1.053272	122	0.9028848	10579
VASCULOGENESIS%GOBP%GO:0001570	0.3436508	1.0397521	28	0.90291065	7956
DNA RECOMBINATION%GOBP%GO:0006310	0.30060694	1.0482477	145	0.90291977	12414
AXONEME ASSEMBLY%GOBP%GO:0035082	0.31786254	1.0520856	56	0.90292	5296
NEGATIVE REGULATION OF FGFR3 SIGNALING%REACTOME%R-HSA-5654732.1	0.36012906	1.0493275	20	0.9029229	8274
MEIOSIS I%GOBP%GO:0007127	0.33365265	1.1142088	62	0.9029275	12396
BIOCARTA_IGF1MTOR_PATHWAY%MSIGDB_C2%BIOCARTA_IGF1MTOR_PATHWAY	0.37479764	1.0420899	18	0.90293705	4020
MULTICELLULAR ORGANISMAL SIGNALING%GOBP%GO:0035637	0.32542926	1.1159372	106	0.90294874	7588
REGULATION OF GLUCOSE IMPORT%GOBP%GO:0046324	0.3307313	1.0398345	33	0.9029621	8214
ZINC TRANSPORTERS%REACTOME%R-HSA-435354.2	0.37749666	1.0402186	15	0.9029815	8975
GPCRS, OTHER%WIKIPATHWAYS_20210110%WP117%HOMO SAPIENS	0.33111838	1.0428532	36	0.9029868	7645
GENE SILENCING%GOBP%GO:0016458	0.3068829	1.0528463	109	0.90298784	8490
HYPOTHESIZED PATHWAYS IN PATHOGENESIS OF CARDIOVASCULAR DISEASE%WIKIPATHWAYS_20210110%WP3668%HOMO SAPIENS	0.35697868	1.0524545	23	0.90299666	2325
SENSORY PERCEPTION OF CHEMICAL STIMULUS%GOBP%GO:0007606	0.33826014	1.0533371	32	0.9030017	6078
CARDIAC MUSCLE CELL DEVELOPMENT%GOBP%GO:0055013	0.35528532	1.0483115	23	0.9030365	7365
ACTIN FILAMENT CAPPING%GOBP%GO:0051693	0.38803145	1.0634376	17	0.90303665	7948
MORPHOGENESIS OF A BRANCHING EPITHELIUM%GOBP%GO:0061138	0.3239018	1.0639341	48	0.9030394	2431
REGULATION OF ERAD PATHWAY%GOBP%GO:1904292	0.3637441	1.1127619	30	0.90305793	5492
SEPARATION OF SISTER CHROMATIDS%REACTOME DATABASE ID RELEASE 75%2467813	0.2981685	1.0519454	165	0.90305984	8983
ANTIMICROBIAL PEPTIDES%REACTOME%R-HSA-6803157.2	0.3515846	1.0544976	25	0.9030812	5899
SUMOYLATION OF CHROMATIN ORGANIZATION PROTEINS%REACTOME DATABASE ID RELEASE 75%4551638	0.32047403	1.0484928	53	0.90309024	5189
DEGRADATION OF GLI2 BY THE PROTEASOME%REACTOME DATABASE ID RELEASE 75%5610783	0.31729195	1.039599	57	0.9030958	8770
MUSCLE ORGAN DEVELOPMENT%GOBP%GO:0007517	0.29943192	1.0400803	125	0.90311885	9816
HISTONE ACETYLATION%GOBP%GO:0016573	0.32622513	1.116669	82	0.9031255	9297
NCAM1 INTERACTIONS%REACTOME%R-HSA-419037.1	0.340061	1.0636116	33	0.9031266	10879
NEURON RECOGNITION%GOBP%GO:0008038	0.33517155	1.0426213	31	0.90312684	8879
HISTONE H4 ACETYLATION%GOBP%GO:0043967	0.32382277	1.0547705	48	0.9031345	9289
REGULATION OF LONG-TERM SYNAPTIC POTENTIATION%GOBP%GO:1900271	0.34145418	1.0515255	27	0.9031386	12481
PYRIDINE NUCLEOTIDE BIOSYNTHETIC PROCESS%GOBP%GO:0019363	0.3426295	1.1065946	52	0.90314037	4362
VESICLE-MEDIATED TRANSPORT IN SYNAPSE%GOBP%GO:0099003	0.30323413	1.0398784	87	0.9031503	5759
REGULATION OF PROTEIN LOCALIZATION TO NUCLEUS%GOBP%GO:1900180	0.3282894	1.1177701	88	0.9031584	8659
CHAPERONE MEDIATED AUTOPHAGY%REACTOME DATABASE ID RELEASE 75%9613829	0.36609185	1.039974	19	0.9031682	13450
POSITIVE REGULATION OF PROTEIN CATABOLIC PROCESS%GOBP%GO:0045732	0.31615737	1.1178762	179	0.90318143	7616
NEGATIVE REGULATION OF MULTI-ORGANISM PROCESS%GOBP%GO:0043901	0.3078578	1.0636959	123	0.9031944	6622
CELLULAR RESPONSE TO LIPOPROTEIN PARTICLE STIMULUS%GOBP%GO:0071402	0.359376	1.0392876	20	0.9031953	10380
LIPID OXIDATION%GOBP%GO:0034440	0.3164228	1.0534803	64	0.9031955	8900
REGULATION OF PHOSPHATASE ACTIVITY%GOBP%GO:0010921	0.31353462	1.0639892	95	0.9032001	7459
CARGO RECOGNITION FOR CLATHRIN-MEDIATED ENDOCYTOSIS%REACTOME%R-HSA-8856825.3	0.29985577	1.0391853	95	0.90320593	10535
NEGATIVE REGULATION OF PROTEIN POLYMERIZATION%GOBP%GO:0032272	0.34035906	1.106685	48	0.9032088	6204
INTERFERON SIGNALING%REACTOME%R-HSA-913531.2	0.29683942	1.0516038	166	0.9032274	8598
BONE REMODELING%GOBP%GO:0046849	0.36465105	1.0373429	19	0.90322804	9141
PURINE RIBONUCLEOSIDE BISPHOSPHATE METABOLIC PROCESS%GOBP%GO:0034035	0.3548451	1.0421022	20	0.903232	4260
VESICLE FUSION%GOBP%GO:0006906	0.31487423	1.0428817	68	0.90323436	11141
TRANSCRIPTIONAL REGULATION BY THE AP-2 (TFAP2) FAMILY OF TRANSCRIPTION FACTORS%REACTOME%R-HSA-8864260.3	0.32920995	1.0403403	35	0.9032353	2124
REGULATION OF CELLULAR PH%GOBP%GO:0030641	0.31361428	1.0520959	70	0.90323997	8675
SARS-COV-1 INFECTION%REACTOME%R-HSA-9678108.2	0.32406372	1.0545515	48	0.9032574	6519
FIBRIN COMPLEMENT RECEPTOR 3 SIGNALING PATHWAY%WIKIPATHWAYS_20210110%WP4136%HOMO SAPIENS	0.3568419	1.0528677	23	0.90326524	5609
ANTIGEN PROCESSING AND PRESENTATION OF EXOGENOUS PEPTIDE ANTIGEN VIA MHC CLASS II%GOBP%GO:0019886	0.3274999	1.1142374	89	0.903276	9094
REGULATION OF MACROPHAGE DERIVED FOAM CELL DIFFERENTIATION%GOBP%GO:0010743	0.36018595	1.0402263	21	0.9032888	11800
POSITIVE REGULATION OF ERYTHROCYTE DIFFERENTIATION%GOBP%GO:0045648	0.35175616	1.0435362	24	0.9032904	6752
MORPHOGENESIS OF A BRANCHING STRUCTURE%GOBP%GO:0001763	0.3200485	1.0407145	53	0.90331215	7674
REGULATION OF ADAPTIVE IMMUNE RESPONSE%GOBP%GO:0002819	0.30780685	1.0429505	90	0.90331703	6556
HALLMARK_COMPLEMENT%MSIGDB_C2%HALLMARK_COMPLEMENT	0.3039781	1.0533491	111	0.9033202	4023
EARLY ENDOSOME TO LATE ENDOSOME TRANSPORT%GOBP%GO:0045022	0.3600158	1.0535458	22	0.9033271	13009
POSITIVE REGULATION OF ORGANELLE ASSEMBLY%GOBP%GO:1902117	0.31893826	1.054812	56	0.9033381	8876
REGULATION OF MICROTUBULE CYTOSKELETON%WIKIPATHWAYS_20210110%WP2038%HOMO SAPIENS	0.33647177	1.0483207	31	0.90334874	10579
SPLICEOSOMAL SNRNP ASSEMBLY%GOBP%GO:0000387	0.32524666	1.0404055	38	0.90335786	7977
POSITIVE REGULATION OF CHEMOTAXIS%GOBP%GO:0050921	0.30510733	1.0485146	94	0.9033649	10945

MEMBRANE INVAGINATION%GOBP%GO:0010324	0.32647997	1.0552028	46	0.9033691	8987
VALIDATED TARGETS OF C-MYC TRANSCRIPTIONAL REPRESSION%PATHWAY INTERACTION DATABASE NCI-NATURE CURATED DATA%VALIDATED TARGETS OF C-MYC TRANSCRIPTIONAL REPRESSION	0.31536102	1.0393361	59	0.9033715	5717
ANTIGEN PROCESSING AND PRESENTATION OF PEPTIDE ANTIGEN VIA MHC CLASS I%GOBP%GO:0002474	0.32640812	1.1120219	89	0.903401	8677
OPIOID PROOPIOMELANOCORTIN PATHWAY%PANTHER PATHWAY%P05917	0.36208826	1.1116837	30	0.9034112	10042
ESTABLISHMENT OF CHROMOSOME LOCALIZATION%GOBP%GO:0051303	0.322447	1.0642321	63	0.9034236	9477
SUMOYLATION OF SUMOYLATION PROTEINS%REACTOME DATABASE ID RELEASE 75%4085377	0.33904848	1.042144	32	0.9034249	4994
LISTERIA MONOCYTOGENES ENTRY INTO HOST CELLS%REACTOME%R-HSA-8876384.1	0.3748085	1.0637316	19	0.9034261	9175
PID_NECTIN_PATHWAY%MSIGDB_C2%PID_NECTIN_PATHWAY	0.5178738	1.5178111	22	0.90343887	1848
PI3K AKT SIGNALING IN CANCER%REACTOME%R-HSA-2219528.2	0.3061967	1.0405777	76	0.90344493	12181
RENAL SYSTEM PROCESS%GOBP%GO:0003014	0.31328934	1.0435914	70	0.9034513	3648
POSITIVE REGULATION OF DENDRITE EXTENSION%GOBP%GO:1903861	0.3728161	1.0548716	17	0.9034749	9432
KISSPEPTIN/KISSPEPTIN RECEPTOR SYSTEM IN THE OVARY%WIKIPATHWAYS_20210110%WP4871%HOMO SAPIENS	0.3420045	1.0407661	28	0.9034796	8670
REGULATION OF PHOSPHOLIPASE C ACTIVITY%GOBP%GO:1900274	0.34529632	1.0517224	34	0.9034833	8799
FORMATION OF HIV ELONGATION COMPLEX IN THE ABSENCE OF HIV TAT%REACTOME%R-HSA-167152.3	0.32539922	1.0521259	44	0.90348816	7291
SIGNALING BY KIT IN DISEASE%REACTOME DATABASE ID RELEASE 75%9669938	0.38667253	1.0609171	16	0.9034984	5320
NEURON PROJECTION EXTENSION%GOBP%GO:1990138	0.3331123	1.0641105	35	0.90350163	2926
MITOCHONDRIAL FISSION%GOBP%GO:0000266	0.47655457	1.3450935	18	0.90350455	751
REGULATION OF RESPONSE TO BIOTIC STIMULUS%GOBP%GO:0002831	0.3003286	1.037359	92	0.90350515	4545
PROTEIN LOCALIZATION TO CILIUM%GOBP%GO:0061512	0.3376124	1.0640033	36	0.9035292	8482
NEPHRIN FAMILY INTERACTIONS%REACTOME DATABASE ID RELEASE 75%373753	0.53337646	1.5373666	21	0.9035515	5243
CEREBRAL CORTEX DEVELOPMENT%GOBP%GO:0021987	0.3284083	1.0545675	46	0.90355456	4944
REGULATION OF EMBRYONIC DEVELOPMENT%GOBP%GO:0045995	0.33039355	1.1166773	81	0.90356314	6298
POSITIVE REGULATION OF TRANSLATIONAL INITIATION%GOBP%GO:0045948	0.3746695	1.1033983	23	0.90356374	3999
METABOLISM OF STEROID HORMONES%REACTOME DATABASE ID RELEASE 75%196071	0.34837642	1.0423009	26	0.9035755	2998
RNA POLYMERASE II TRANSCRIPTION TERMINATION%REACTOME DATABASE ID RELEASE 75%73856	0.32330447	1.0516053	65	0.90357894	3825
DEGRADATION OF DVL%REACTOME%R-HSA-4641258.2	0.34352097	1.1120868	55	0.90359145	9483
RNA POLYMERASE III TRANSCRIPTION INITIATION FROM TYPE 1 PROMOTER%REACTOME%R-HSA-76061.2	0.33833733	1.0429761	28	0.90359986	8398
POSITIVE REGULATION OF PROTEIN UBIQUITINATION%GOBP%GO:0031398	0.3252704	1.1066993	95	0.9036051	8074
SIGNALING BY NOTCH2%REACTOME%R-HSA-1980145.2	0.3374808	1.0436472	31	0.9036079	10885
TRANSLESION SYNTHESIS BY POLI%REACTOME%R-HSA-5656121.1	0.37543154	1.0552312	17	0.9036323	11716
COLUMNAR/CUBOIDAL EPITHELIAL CELL DEVELOPMENT%GOBP%GO:0002066	0.38085786	1.0642729	18	0.9036402	809
MBDNF AND PROBDNF REGULATION OF GABA NEUROTRANSMISSION%WIKIPATHWAYS_20210110%WP4829%HOMO SAPIENS	0.36711445	1.1117364	28	0.9036595	4944
NEGATIVE REGULATION OF MAPK CASCADE%GOBP%GO:0043409	0.30328405	1.0550197	125	0.9036602	7222
PROTEIN ALPHA-1,2-DEMANNOSYLATION%GOBP%GO:0036508	0.35830492	1.0404136	20	0.9036749	7642
TNFR2 NON-CANONICAL NF-KB PATHWAY%REACTOME DATABASE ID RELEASE 75%5668541	0.3252025	1.1045455	84	0.90368664	8770
CELLULAR MONOVALENT INORGANIC CATION HOMEOSTASIS%GOBP%GO:0030004	0.30439308	1.0371139	84	0.903724	9662
GLI3 IS PROCESSED TO GLI3R BY THE PROTEASOME%REACTOME DATABASE ID RELEASE 75%5610785	0.3135977	1.0421566	57	0.9037247	8770
HORMONE BIOSYNTHETIC PROCESS%GOBP%GO:0042446	0.33128196	1.029927	36	0.90374243	6290
TRICARBOXYLIC ACID METABOLIC PROCESS%GOBP%GO:0072350	0.33433416	1.0609514	33	0.90374464	3899
IRAK2 MEDIATED ACTIVATION OF TAK1 COMPLEX UPON TLR7 8 OR 9 STIMULATION%REACTOME%R-HSA-975163.1	0.3746631	1.0368156	15	0.9037448	13438
POSITIVE REGULATION OF STAT CASCADE%GOBP%GO:1904894	0.318728	1.059858	50	0.9037492	3051
ALPHA6BETA4INTEGRIN%NETPATH%ALPHA6BETA4INTEGRIN	0.31990334	1.0548829	55	0.9037945	11345
PRIMARY NEURAL TUBE FORMATION%GOBP%GO:0014020	0.3239846	1.0282767	37	0.90380996	6318
REGULATION OF LIPID METABOLISM BY PPARALPHA%REACTOME DATABASE ID RELEASE 75%400206	0.30514422	1.0643241	109	0.9038191	7464
LATE PHASE OF HIV LIFE CYCLE%REACTOME%R-HSA-162599.2	0.31686643	1.1035476	129	0.9038276	8531
RHO GTPASES ACTIVATE WASPS AND WAVES%REACTOME DATABASE ID RELEASE 75%5663213	0.3310822	1.0430005	34	0.9038295	7004
PID_CMYB_PATHWAY%MSIGDB_C2%PID_CMYB_PATHWAY	0.31557757	1.0436745	54	0.90385574	4770
ESTROGEN-DEPENDENT GENE EXPRESSION%REACTOME%R-HSA-9018519.1	0.30295682	1.0438652	107	0.9038822	6563
REGULATION OF NEUROTRANSMITTER LEVELS%GOBP%GO:0001505	0.3075764	1.1068403	210	0.9039168	6420
ACUTE INFLAMMATORY RESPONSE%GOBP%GO:0002526	0.3661386	1.0610079	19	0.9039183	3250
SIGNALING BY NON-RECEPTOR TYROSINE KINASES%REACTOME%R-HSA-9006927.3	0.31533155	1.0366637	48	0.9039365	13855
MODULATION BY HOST OF SYMBIONT TRANSCRIPTION%GOBP%GO:0052472	0.34644926	1.0369518	22	0.9039385	5648
REPLICATION FORK PROCESSING%GOBP%GO:0031297	0.33637953	1.063095	38	0.9039409	8860
TRANSCRIPTION ELONGATION FROM RNA POLYMERASE II PROMOTER%GOBP%GO:0006368	0.33647987	1.1174828	65	0.90394574	7669
3'-PHOSPHOADENOSINE 5'-PHOSPHOSULFATE METABOLIC PROCESS%GOBP%GO:0050427	0.3548451	1.0262114	20	0.9039458	4260
MAINTENANCE OF PROTEIN LOCATION%GOBP%GO:0045185	0.33055857	1.1166995	81	0.9039464	5972
DEVELOPMENT OF PRIMARY MALE SEXUAL CHARACTERISTICS%GOBP%GO:0046546	0.3455377	1.1045928	36	0.9039569	8945
POSITIVE REGULATION OF CARDIAC MUSCLE HYPERTROPHY%GOBP%GO:0010613	0.48315138	1.3505676	17	0.9039666	9770
CELL-CELL ADHESION VIA PLASMA-MEMBRANE ADHESION MOLECULES%GOBP%GO:0098742	0.30506417	1.0624105	135	0.9039758	2679
NEGATIVE REGULATION OF DEFENSE RESPONSE TO VIRUS%GOBP%GO:0050687	0.369445	1.0623095	19	0.9039789	6496
POSITIVE REGULATION OF POSTTRANSCRIPTIONAL GENE SILENCING%GOBP%GO:0060148	0.3471427	1.0626975	28	0.90398043	8490
EPITHELIAL TUBE FORMATION%GOBP%GO:0072175	0.33913895	1.103403	50	0.9039885	6318
STEROL TRANSPORT%GOBP%GO:0015918	0.31722623	1.0262921	43	0.90398896	8637
NEGATIVE REGULATION OF TRANSFERASE ACTIVITY%GOBP%GO:0051348	0.29243368	1.0552313	215	0.9039936	7616
INSULIN IGF PATHWAY-MITOGEN ACTIVATED PROTEIN KINASE KINASE MAP KINASE CASCADE%PANTHER PATHWAY%P00032	0.33878836	1.0437329	30	0.90399474	10294
CENTRAL NERVOUS SYSTEM NEURON DIFFERENTIATION%GOBP%GO:0021953	0.3094486	1.0430508	80	0.9040002	7025
CELLULAR RESPONSE TO VASCULAR ENDOTHELIAL GROWTH FACTOR STIMULUS%GOBP%GO:0035924	0.34665546	1.104372	37	0.90400153	7352
REGULATION OF CENTROSOME CYCLE%GOBP%GO:0046605	0.32168972	1.0606811	52	0.90401703	8711
TNF ALPHA SIGNALING PATHWAY%WIKIPATHWAYS_20210110%WP231%HOMO SAPIENS	0.306193	1.0299366	71	0.9040354	11221
C-MYC PATHWAY%PATHWAY INTERACTION DATABASE NCI-NATURE CURATED DATA%C-MYC PATHWAY	0.34513777	1.0431386	25	0.9040366	8329
CHEMOKINE-MEDIATED SIGNALING PATHWAY%GOBP%GO:0070098	0.36089295	1.1067002	30	0.9040541	5434
RESPONSE TO ENDOPLASMIC RETICULUM STRESS%GOBP%GO:0034976	0.29611123	1.0625796	229	0.9040579	5492
MATURATION OF 5.8S RRNA%GOBP%GO:0000460	0.35872152	1.1117519	33	0.90406346	5356
NUCLEOTIDE-EXCISION REPAIR, DNA DAMAGE RECOGNITION%GOBP%GO:0000715	0.34364283	1.0368204	23	0.9040636	4807
REGULATION OF MYELOID CELL DIFFERENTIATION%GOBP%GO:0045637	0.29202566	1.0263656	140	0.9040662	7447
POSITIVE REGULATION OF ESTABLISHMENT OF PROTEIN LOCALIZATION TO MITOCHONDRION%GOBP%GO:1903749	0.34154058	1.1028495	54	0.904068	9849
BETA2 ADRENERGIC RECEPTOR SIGNALING PATHWAY%PANTHER PATHWAY%P04378	0.35737532	1.0432258	22	0.9040707	6094
VIRAL MESSENGER RNA SYNTHESIS%REACTOME DATABASE ID RELEASE 75%168325	0.32971877	1.0598722	41	0.9040717	5533
TRIF(TICAM1)-MEDIATED TLR4 SIGNALING%REACTOME%R-HSA-937061.2	0.30571842	1.0610604	94	0.90408593	8274
FAS%IOB%FAS	0.32648805	1.102944	81	0.904107	5872
POSITIVE REGULATION OF MITOTIC CELL CYCLE%GOBP%GO:0045931	0.30669913	1.0643476	126	0.90411186	10555
GLYCEROPHOSPHOLIPID BIOSYNTHETIC PROCESS%GOBP%GO:0046474	0.29704127	1.0595512	198	0.9041211	8404
REGULATION OF LAMELLIPODIUM ASSEMBLY%GOBP%GO:0010591	0.35855597	1.1035836	31	0.9041243	4406
PRIMARY FOCAL SEGMENTAL GLOMERULOSCLEROSIS FSGS%WIKIPATHWAYS_20210110%WP2572%HOMO SAPIENS	0.33637565	1.10413	54	0.90412694	7644
PRESYNAPTIC PHASE OF HOMOLOGOUS DNA PAIRING AND STRAND EXCHANGE%REACTOME%R-HSA-5693616.1	0.32890546	1.0282768	37	0.9041354	12221
POSITIVE REGULATION OF MONONUCLEAR CELL MIGRATION%GOBP%GO:0071677	0.387634	1.0627477	15	0.9041565	4906
NEGATIVE REGULATION OF CELL CYCLE G2/M PHASE TRANSITION%GOBP%GO:1902750	0.3002055	1.0279717	97	0.9041821	9483
REGULATION OF RUNX3 EXPRESSION AND ACTIVITY%REACTOME%R-HSA-8941858.1	0.3209173	1.0611385	55	0.9041868	8770
MULTI-ORGANISM TRANSPORT%GOBP%GO:0044766	0.33382908	1.1167501	67	0.9042064	7794
1362300.2	0.36944032	1.0278641	16	0.9042236	3541
SIGNALING BY EGFR IN CANCER%REACTOME DATABASE ID RELEASE 75%1643713	0.345968	1.0302591	21	0.9042249	10363
EBV LMP1 SIGNALING%WIKIPATHWAYS_20210110%WP262%HOMO SAPIENS	0.39790097	1.1054839	17	0.9042285	10945
REGULATION OF NEUROTRANSMITTER SECRETION%GOBP%GO:0046928	0.33099812	1.0628175	42	0.9042584	5621
INNER MITOCHONDRIAL MEMBRANE ORGANIZATION%GOBP%GO:0007007	0.31412867	1.0272782	51	0.9042667	12034
SIGNALING IN THE KIDNEY PODOCYTE	0.44994575	1.3464179	27	0.90426874	5640
B CELL ACTIVATION%PANTHER PATHWAY%P00010	0.3439428	1.103115	51	0.904274	4681
NUCLEAR CHROMOSOME SEGREGATION%GOBP%GO:0098813	0.3132319	1.1069797	166	0.90427965	12942
TUBE FORMATION%GOBP%GO:0035148	0.3098317	1.0303352	59	0.9042826	6318
REV-MEDIATED NUCLEAR EXPORT OF HIV RNA%REACTOME%R-HSA-165054.2	0.32893005	1.0299568	32	0.9042883	4994
TRANSCRIPTION OF E2F TARGETS UNDER NEGATIVE CONTROL BY DREAM COMPLEX%REACTOME%R-HSA-1362277.2	0.36625773	1.0650845	19	0.90430623	3764
RNA POLYMERASE I PROMOTER OPENING%REACTOME DATABASE ID RELEASE 75%73728	0.35370508	1.0283179	22	0.9043089	3166
TRIGLYCERIDE HOMEOSTASIS%GOBP%GO:0070328	0.38188827	1.0624214	16	0.90431064	9399
REGULATION OF PROTEIN DEPHOSPHORYLATION%GOBP%GO:0035304	0.3098613	1.0291811	67	0.9043108	3289
NEGATIVE REGULATION OF ATP METABOLIC PROCESS%GOBP%GO:1903579	0.38219288	1.0646908	16	0.9043122	6279
RESPONSE TO NUTRIENT%GOBP%GO:0007584	0.34273395	1.109527	55	0.904318	10348
REGULATION OF NEUROLOGICAL SYSTEM PROCESS%GOBP%GO:0031644	0.3068201	1.0277389	71	0.9043198	6588
NEGATIVE REGULATION OF FATTY ACID METABOLIC PROCESS%GOBP%GO:0045922	0.3633064	1.0270686	19	0.904321	10931
EPITHELIAL TUBE MORPHOGENESIS%GOBP%GO:0060562	0.31573987	1.1068523	135	0.90432304	7674
AMINO ACID ACTIVATION%GOBP%GO:0043038	0.32021448	1.0276394	46	0.9043239	8624
REGIONALIZATION%GOBP%GO:0003002	0.29796767	1.0269693	125	0.9043262	10917
REGULATION OF IMMUNOGLOBULIN PRODUCTION%GOBP%GO:0002637	0.3348983	1.028024	28	0.90433437	8102
POSITIVE REGULATION OF DNA-TEMPLATED TRANSCRIPTION, INITIATION%GOBP%GO:2000144	0.34774515	1.0552372	28	0.9043352	6191
NEGATIVE REGULATION OF LYMPHOCYTE MEDIATED IMMUNITY%GOBP%GO:0002707	0.36525553	1.026472	17	0.90435064	4842
P53 PATHWAY%PATHWAY INTERACTION DATABASE NCI-NATURE CURATED DATA%P53 PATHWAY	0.3199193	1.059587	59	0.90435725	7080
KIDNEY DEVELOPMENT%GOBP%GO:0001822	0.29526874	1.0263753	127	0.9043606	7728
NUCLEAR ENVELOPE BREAKDOWN%REACTOME%R-HSA-2980766.2	0.31512395	1.0337979	47	0.9043649	7794
ALPHA-AMINO ACID CATABOLIC PROCESS%GOBP%GO:1901606	0.32666406	1.1045992	76	0.9043722	4168
REGULATION OF ACUTE INFLAMMATORY RESPONSE%GOBP%GO:0002673	0.32808346	1.0649657	49	0.90438193	5007
POSITIVE REGULATION OF DENDRITE DEVELOPMENT%GOBP%GO:1900006	0.3193506	1.0284908	42	0.90438896	11575
MAINTENANCE OF CELL NUMBER%GOBP%GO:0098727	0.3101204	1.0339863	63	0.90439254	5533
MICRORNA (MIRNA) BIOGENESIS%REACTOME DATABASE ID RELEASE 75%203927	0.3781222	1.1168187	24	0.9043927	5533
INTERLEUKIN-17 SIGNALING%REACTOME DATABASE ID RELEASE 75%448424	0.30774528	1.0271437	67	0.9043997	10539
SIGNALING EVENTS	0.37846622	1.1076962	23	0.90440404	4995
MRNA 3'-END PROCESSING%GOBP%GO:0031124	0.3037009	1.0338821	83	0.90440744	5209
STRESS-ACTIVATED MAPK CASCADE%GOBP%GO:0051403	0.30922875	1.055416	83	0.9044162	3310
BASE EXCISION REPAIR%REACTOME DATABASE ID RELEASE 75%73884	0.3213145	1.0652593	61	0.90441686	2059

REGULATION OF DNA-TEMPLATED TRANSCRIPTION, INITIATION%GOBP%GO:2000142	0.34380683	1.0611758	32	0.90442276	6191
PROTEIN-DNA COMPLEX SUBUNIT ORGANIZATION%GOBP%GO:0071824	0.29970258	1.0598766	176	0.9044257	9172
SKELETAL SYSTEM DEVELOPMENT%GOBP%GO:0001501	0.28776926	1.0286672	233	0.9044311	7258
TOLL LIKE RECEPTOR TLR6:TLR2 CASCADE%REACTOME%R-HSA-168188.1	0.3267905	1.1077912	89	0.9044448	10539
REGULATION OF EPITHELIAL CELL DIFFERENTIATION%GOBP%GO:0030856	0.32144892	1.103835	95	0.90444565	7222
SIGNALING BY PDGF%REACTOME%R-HSA-186797.4	0.34327796	1.107048	47	0.9044462	4607
NEGATIVE REGULATION OF CELL ACTIVATION%GOBP%GO:0050866	0.30963606	1.0651476	95	0.904448	6153
REGULATION OF CYTOPLASMIC TRANSLATION%GOBP%GO:2000765	0.3717959	1.1041557	24	0.9044589	10532
VIRAL ACUTE MYOCARDITIS%WIKIPATHWAYS_20210110%WP4298%HOMO SAPIENS	0.3417552	1.1056466	51	0.9044627	5257
ACTIVATION OF GTPASE ACTIVITY%GOBP%GO:0090630	0.32388228	1.1029642	93	0.9044674	7174
PROTEINS.%REACTOME%R-HSA-163200.1	0.29416513	1.0335844	123	0.9044674	6736
PID_FOXM1_PATHWAY%MSIGDB_C2%PID_FOXM1_PATHWAY	0.3320108	1.0292301	35	0.90447754	12786
RESPONSE TO ORGANOPHOSPHORUS%GOBP%GO:0046683	0.32633215	1.0643497	52	0.9044808	2371
NUCLEOSIDE DIPHOSPHATE METABOLIC PROCESS%GOBP%GO:0009132	0.33514467	1.1047825	53	0.9044826	7952
VITAMIN B5 (PANTOTHENATE) METABOLISM%REACTOME%R-HSA-199220.2	0.37951097	1.0273056	15	0.90449506	3883
INTERNALIZATION OF ERBB1%PATHWAY INTERACTION DATABASE NCI-NATURE CURATED DATA%INTERNALIZATION OF ERBB1	0.3463524	1.1052021	38	0.90449697	4020
GASTRULATION%GOBP%GO:0007369	0.330014	1.1091654	73	0.90449923	6910
NEGATIVE REGULATION OF EXTRINSIC APOPTOTIC SIGNALING PATHWAY IN ABSENCE OF LIGAND%GOBP%GO:2001240	0.3929833	1.1071326	18	0.90451473	5981
PROTEIN DEUBIQUITINATION%GOBP%GO:0016579	0.28331143	1.0190724	238	0.90452003	6903
REGULATION OF GTPASE ACTIVITY%GOBP%GO:0043087	0.3064443	1.1055236	233	0.9045251	8896
POSITIVE REGULATION OF TRANSCRIPTION FROM RNA POLYMERASE II PROMOTER IN RESPONSE TO STRESS%GOBP%GO:0036003	0.37415168	1.1058434	22	0.9045266	6322
TRAIL SIGNALING PATHWAY%PATHWAY INTERACTION DATABASE NCI-NATURE CURATED DATA%TRAIL SIGNALING PATHWAY	0.36963388	1.1036994	24	0.90452665	4417
PORPHYRIN-CONTAINING COMPOUND BIOSYNTHETIC PROCESS%GOBP%GO:0006779	0.36129558	1.0299824	19	0.90452725	3593
REGULATION OF NEUROTRANSMITTER TRANSPORT%GOBP%GO:0051588	0.3163543	1.0593454	68	0.9045287	5621
MUSCULOSKELETAL MOVEMENT%GOBP%GO:0050881	0.36035708	1.0283487	21	0.90453357	7419
SIGNALING BY ERBB2%REACTOME%R-HSA-1227986.7	0.31607443	1.0336591	44	0.9045355	11324
NEGATIVE REGULATION OF TUMOR NECROSIS FACTOR-MEDIATED SIGNALING PATHWAY%GOBP%GO:0010804	0.40797627	1.1035913	15	0.9045387	12553
NUCLEOTIDE-EXCISION REPAIR, DNA INCISION, 3'-TO LESION%GOBP%GO:0006295	0.37210754	1.1039203	21	0.9045414	13964
REGULATION OF RENAL SYSTEM PROCESS%GOBP%GO:0098801	0.4041891	1.1049858	15	0.90454185	6935
WNT%NETPATH%WNT	0.31264275	1.0645301	87	0.9045537	7219
WNT SIGNALING%WIKIPATHWAYS_20210110%WP428%HOMO SAPIENS	0.3111488	1.0599436	89	0.90455496	6342
POSITIVE REGULATION OF CALCIUM ION TRANSMEMBRANE TRANSPORT%GOBP%GO:1904427	0.33317742	1.0648197	39	0.90455776	9706
POSITIVE REGULATION OF PEPTIDYL-SERINE PHOSPHORYLATION%GOBP%GO:0033138	0.31505328	1.0628325	69	0.90456754	7590
MESENCHYMAL CELL DEVELOPMENT%GOBP%GO:0014031	0.3373846	1.1052921	50	0.9045707	9324
REGULATION OF MONOCYTE CHEMOTAXIS%GOBP%GO:0090025	0.3616204	1.0295881	19	0.9045724	5856
POSITIVE REGULATION OF PHOSPHOLIPASE C ACTIVITY%GOBP%GO:0010863	0.33270442	1.0265017	31	0.90457314	12347
ANTIBIOTIC CATABOLIC PROCESS%GOBP%GO:0017001	0.34310868	1.0558621	30	0.9045732	4652
REGULATION OF ERYTHROCYTE DIFFERENTIATION%GOBP%GO:0045646	0.33991078	1.0557663	33	0.9045794	6752
PID_ILK_PATHWAY%MSIGDB_C2%PID_ILK_PATHWAY	0.34344268	1.06472	31	0.9045802	5243
INTERNAL PEPTIDYL-LYSINE ACETYLATION%GOBP%GO:0018393	0.32647777	1.1072224	84	0.90458924	9297
REGULATION OF NON-CANONICAL WNT SIGNALING PATHWAY%GOBP%GO:2000050	0.38560107	1.1090345	20	0.9045972	6289
NEGATIVE REGULATION OF MUSCLE TISSUE DEVELOPMENT%GOBP%GO:1901862	0.35090804	1.0303376	22	0.90460545	2385
REGULATION OF HISTONE DEACETYLATION%GOBP%GO:0031063	0.36727893	1.0189524	16	0.9046065	12982
MAINTENANCE OF LOCATION%GOBP%GO:0051235	0.2943607	1.0294816	123	0.9046106	6838
LEISHMANIA PARASITE GROWTH AND SURVIVAL%REACTOME DATABASE ID RELEASE 75%9664433	0.31940645	1.1084963	114	0.90461695	6299
URETERIC BUD DEVELOPMENT%GOBP%GO:0001657	0.33871555	1.0552607	30	0.90461856	7728
PYRIMIDINE-CONTAINING COMPOUND BIOSYNTHETIC PROCESS%GOBP%GO:0072528	0.36797467	1.1169837	27	0.9046297	561
LYSOSOME ORGANIZATION%GOBP%GO:0007040	0.32275772	1.0560453	50	0.90463	8796
NOTCH%NETPATH%NOTCH	0.32041296	1.0603143	58	0.90463585	7098
NEGATIVE EPIGENETIC REGULATION OF RRNA EXPRESSION%REACTOME DATABASE ID RELEASE 75%5250941	0.30753934	1.0300434	65	0.90464896	6737
PEPTIDYL-LYSINE ACETYLATION%GOBP%GO:0018394	0.3113952	1.0592121	87	0.90464973	9297
CHEMOKINE RECEPTORS BIND CHEMOKINES%REACTOME DATABASE ID RELEASE 75%380108	0.36740744	1.059014	21	0.90465474	5434
ORGANELLE FUSION%GOBP%GO:0048284	0.3251934	1.1046348	92	0.9046568	11256
REGULATION OF TRANSMEMBRANE RECEPTOR PROTEIN SERINE/THREONINE KINASE SIGNALING PATHWAY%GOBP%GO:0090092	0.31373507	1.1111676	188	0.9046595	7534
CREB1 PHOSPHORYLATION THROUGH NMDA RECEPTOR-MEDIATED ACTIVATION OF RAS SIGNALING%REACTOME DATABASE ID RELEASE 75%442742	0.34320402	1.0285088	27	0.90466005	5550
PROTEIN DESTABILIZATION%GOBP%GO:0031648	0.4904961	1.5194479	31	0.90466255	4028
P53 PATHWAY%PANTHER PATHWAY%P00059	0.31633565	1.0286931	46	0.90466386	8404
PID_IGF1_PATHWAY%MSIGDB_C2%PID_IGF1_PATHWAY	0.35626638	1.0602031	22	0.90466464	4378
CELLULAR RESPONSE TO COPPER ION%GOBP%GO:0071280	0.37686235	1.0273503	15	0.9046669	2338
PEPTIDYL-LYSINE TRIMETHYLATION%GOBP%GO:0018023	0.3533592	1.0613111	25	0.9046727	4751
INTEGRATED BREAST CANCER PATHWAY%WIKIPATHWAYS_20210110%WP1984%HOMO SAPIENS	0.29723975	1.0340018	122	0.90467525	12221
POSITIVE REGULATION OF IMMUNOGLOBULIN PRODUCTION%GOBP%GO:0002639	0.36054888	1.0652908	24	0.90467614	6556
POSITIVE REGULATION OF CHOLESTEROL EFFLUX%GOBP%GO:0010875	0.37206078	1.0643965	19	0.9046792	7905
POSITIVE REGULATION OF GLUCOSE METABOLIC PROCESS%GOBP%GO:0010907	0.346233	1.0292629	22	0.9046887	11474
NEGATIVE REGULATION OF SMALL MOLECULE METABOLIC PROCESS%GOBP%GO:0062014	0.33439285	1.109546	69	0.9047018	10931
SPINAL CORD DEVELOPMENT%GOBP%GO:0021510	0.34159988	1.0611991	34	0.9047086	9791
REGULATION OF CILIUM MOVEMENT%GOBP%GO:0003352	0.39024785	1.0573018	16	0.9047111	6910
LYSOSOME VESICLE BIOGENESIS%REACTOME DATABASE ID RELEASE 75%432720	0.338492	1.0570066	35	0.90471435	4389
NAIVE CD8+ T CELLS	0.32379255	1.0304016	35	0.90471834	11091
CELLULAR RESPONSE TO TYPE I INTERFERON%GOBP%GO:0071357	0.31272462	1.0341854	50	0.9047318	6592
CRISTAE FORMATION%GOBP%GO:0042407	0.33323482	1.0342817	31	0.9047355	12034
RESPIRATORY TUBE DEVELOPMENT%GOBP%GO:0030323	0.32554263	1.0554276	47	0.904746	4082
HALLMARK_ALLOGRAFT_REJECTION%MSIGDB_C2%HALLMARK_ALLOGRAFT_REJECTION	0.31918657	1.1056865	103	0.90475035	5010
RIBOSOMAL LARGE SUBUNIT ASSEMBLY%GOBP%GO:0000027	0.3424839	1.0343704	26	0.9047544	10700
NEGATIVE REGULATION OF SIGNAL TRANSDUCTION IN ABSENCE OF LIGAND%GOBP%GO:1901099	0.3929833	1.1170671	18	0.9047592	5981
NUCLEOCYTOPLASMIC TRANSPORT%GOBP%GO:0006913	0.29682705	1.0603834	214	0.904763	5437
NEGATIVE REGULATION OF LYMPHOCYTE PROLIFERATION%GOBP%GO:0050672	0.3348959	1.0293351	30	0.9047682	5816
HATS ACETYLATE HISTONES%REACTOME DATABASE ID RELEASE 75%3214847	0.3229638	1.1050378	95	0.90477043	7922
NEPHRON TUBULE MORPHOGENESIS%GOBP%GO:0072078	0.34071743	1.0340701	28	0.90477365	7674
ESTABLISHMENT OF SPINDLE LOCALIZATION%GOBP%GO:0051293	0.31966314	1.0191863	37	0.9047778	5883
CARDIAC EPITHELIAL TO MESENCHYMAL TRANSITION%GOBP%GO:0060317	0.3650589	1.0265344	18	0.90478075	10075
LUNG DEVELOPMENT%GOBP%GO:0030324	0.34015343	1.1078156	45	0.90478945	4082
FORMATION OF THE HIV-1 EARLY ELONGATION COMPLEX%REACTOME DATABASE ID RELEASE 75%167158	0.33988595	1.0563977	33	0.90479577	6737
POSITIVE REGULATION OF ATP BIOSYNTHETIC PROCESS%GOBP%GO:2001171	0.3388661	1.0288421	25	0.90481645	2885
CDC20:PHOSPHO-APC C MEDIATED DEGRADATION OF CYCLIN A%REACTOME%R-HSA-174184.2	0.331538	1.1168306	71	0.90481776	8898
BLOOD COAGULATION%PANTHER PATHWAY%P00011	0.5194912	1.5403053	23	0.9048229	3051
POSITIVE REGULATION OF CELL CYCLE PHASE TRANSITION%GOBP%GO:1901989	0.40156853	1.3466232	75	0.90482527	7560
NEGATIVE REGULATION OF VIRAL PROCESS%GOBP%GO:0048525	0.3129216	1.05629	82	0.904828	5956
RNA POLYMERASE III TRANSCRIPTION INITIATION FROM TYPE 2 PROMOTER%REACTOME%R-HSA-76066.2	0.3607191	1.1072723	27	0.90483433	8398
BIOCARTA_G2_PATHWAY%MSIGDB_C2%BIOCARTA_G2_PATHWAY	0.3586004	1.0190725	18	0.9048354	7013
CILIUM OR FLAGELLUM-DEPENDENT CELL MOTILITY%GOBP%GO:0001539	0.36897534	1.059966	18	0.9048423	5064
MEIOTIC CELL CYCLE PROCESS%GOBP%GO:1903046	0.32256386	1.1087626	93	0.9048472	8792
DISEASES OF CARBOHYDRATE METABOLISM%REACTOME%R-HSA-5663084.3	0.5020829	1.5382447	29	0.9048494	4930
CDT1 ASSOCIATION WITH THE CDC6:ORC:ORIGIN COMPLEX%REACTOME DATABASE ID RELEASE 75%68827	0.31223467	1.0289273	57	0.9048504	8770
CADHERIN SIGNALING PATHWAY%PANTHER PATHWAY%P00012	0.31659698	1.1048025	141	0.904854	11172
MICROTUBULE ORGANIZING CENTER ORGANIZATION%GOBP%GO:0031023	0.32644525	1.1093023	91	0.9048604	5559
REGULATION OF TUMOR NECROSIS FACTOR-MEDIATED SIGNALING PATHWAY%GOBP%GO:0010803	0.3236238	1.0555898	51	0.90487117	11221
FATTY ACID TRANSPORT%GOBP%GO:0015908	0.33899873	1.1058589	47	0.9049039	8647
REGULATION OF CALCIUM ION TRANSPORT INTO CYTOSOL%GOBP%GO:0010522	0.30888858	1.0273762	60	0.90490454	10960
RESPONSE TO DECREASED OXYGEN LEVELS%GOBP%GO:0036293	0.2979173	1.0558704	190	0.9049089	6322
FAS (CD95) SIGNALING PATHWAY%PATHWAY INTERACTION DATABASE NCI-NATURE CURATED DATA%FAS (CD95) SIGNALING PATHWAY	0.3437775	1.0560666	32	0.90492225	4048
SENESCENCE-ASSOCIATED SECRETORY PHENOTYPE (SASP)%REACTOME%R-HSA-2559582.2	0.31870005	1.0590398	70	0.90492964	9837
ADIPOGENESIS%WIKIPATHWAYS_20210110%WP236%HOMO SAPIENS	0.29931226	1.026589	98	0.90493375	8102
POSITIVE REGULATION OF NEURON PROJECTION DEVELOPMENT%GOBP%GO:0010976	0.31103435	1.1091702	171	0.90493804	8439
SIGNALING BY PTK6%REACTOME%R-HSA-8848021.2	0.31533155	1.0192343	48	0.9049403	13855
REGULATION OF VIRAL-INDUCED CYTOPLASMIC PATTERN RECOGNITION RECEPTOR SIGNALING PATHWAY%GOBP%GO:0039531	0.3691485	1.0565507	19	0.9049459	4545
MITOCHONDRIAL RESPIRATORY CHAIN COMPLEX ASSEMBLY%GOBP%GO:0033108	0.29923853	1.0287026	97	0.9049527	6741
RECRUITMENT OF MITOTIC CENTROSOME PROTEINS AND COMPLEXES%REACTOME%R-HSA-380270.2	0.3140905	1.057431	80	0.9049629	9743
PROTEIN HOMOOLIGOMERIZATION%GOBP%GO:0051260	0.31299922	1.065315	103	0.9049658	5054
DNA DOUBLE STRAND BREAK RESPONSE%REACTOME%R-HSA-5693606.2	0.3216903	1.0561539	54	0.90497226	6189
NEGATIVE REGULATION OF MAP KINASE ACTIVITY%GOBP%GO:0043407	0.31113026	1.0344056	62	0.90497357	6318
POSITIVE REGULATION OF GLUCOSE IMPORT%GOBP%GO:0046326	0.34778562	1.0307202	23	0.90497464	3549
REGULATION OF MORPHOGENESIS OF AN EPITHELIUM%GOBP%GO:1905330	0.3151986	1.1060629	135	0.9049805	8689
NEGATIVE REGULATION OF TRANSLATION%GOBP%GO:0017148	0.30371365	1.0570338	109	0.90498114	7030
PARKINSONS DISEASE PATHWAY%WIKIPATHWAYS_20210110%WP2371%HOMO SAPIENS	0.33991745	1.0573248	30	0.9049941	5749
MRNA POLYADENYLATION%GOBP%GO:0006378	0.3583681	1.1073422	30	0.9049972	2859
STRESS FIBER ASSEMBLY%GOBP%GO:0043149	0.3881429	1.0613239	15	0.9049976	4069
STEROID BIOSYNTHETIC PROCESS%GOBP%GO:0006694	0.31251898	1.0554531	84	0.9050046	5171
NEPHRON DEVELOPMENT%GOBP%GO:0072006	0.3048282	1.0306112	73	0.9050069	7094
TRNA AMINOACYLATION%GOBP%GO:0043039	0.32021448	1.03041	46	0.90502125	8624
NRIF SIGNALS CELL DEATH FROM THE NUCLEUS%REACTOME%R-HSA-205043.1	0.39807495	1.1088322	16	0.9050253	4921
GLYCEROLIPID BIOSYNTHETIC PROCESS%GOBP%GO:0045017	0.2890555	1.0309052	209	0.90503067	8404
TOLL LIKE RECEPTOR TLR1:TLR2 CASCADE%REACTOME DATABASE ID RELEASE 75%168179	0.3267905	1.1085044	89	0.9050333	10539
REGULATION OF CELL MORPHOGENESIS INVOLVED IN DIFFERENTIATION%GOBP%GO:0010769	0.2970535	1.0600076	209	0.9050446	6313
TETRAPYRROLE BIOSYNTHETIC PROCESS%GOBP%GO:0033014	0.36129558	1.0192949	19	0.9050555	3593
ESTABLISHMENT OF SYNAPTIC VESICLE LOCALIZATION%GOBP%GO:0097480	0.32401943	1.0653876	66	0.9050639	2208
ANTI-INFLAMMATORY RESPONSE FAVOURING LEISHMANIA PARASITE INFECTION%REACTOME DATABASE ID RELEASE 75%9662851	0.31940645	1.1059294	114	0.905064	6299

REGULATION OF CYTOPLASMIC TRANSPORT%GOBP%GO:1903649	0.3694832	1.1111814	26	0.9050674	4209
FORMATION OF THE EARLY ELONGATION COMPLEX%REACTOME DATABASE ID RELEASE 75%113418	0.33988595	1.0586964	33	0.90507305	6737
HETEROTRIMERIC G-PROTEIN SIGNALING PATHWAY-ROD OUTER SEGMENT PHOTOTRANSDUCTION%PANTHER PATHWAY%P00028	0.35456714	1.0615062	23	0.9050867	6094
UB-SPECIFIC PROCESSING PROTEASES%REACTOME DATABASE ID RELEASE 75%5689880	0.2886237	1.0193787	172	0.9050925	8966
BRANCHING MORPHOGENESIS OF AN EPITHELIAL TUBE%GOBP%GO:0048754	0.33716816	1.107848	46	0.9051025	2431
INTEGRIN-MEDIATED CELL ADHESION%WIKIPATHWAYS_20210110%WP185%HOMO SAPIENS	0.30986577	1.0307791	68	0.9051095	5815
IL17 SIGNALING PATHWAY%WIKIPATHWAYS_20210110%WP2112%HOMO SAPIENS	0.34562913	1.0206236	23	0.90511733	5208
PYRIDINE-CONTAINING COMPOUND BIOSYNTHETIC PROCESS%GOBP%GO:0072525	0.33510077	1.1095556	55	0.9051222	5094
POSITIVE REGULATION OF CYSTEINE-TYPE ENDOPEPTIDASE ACTIVITY%GOBP%GO:2001056	0.30306542	1.0566044	119	0.90512335	6252
MULTICELLULAR ORGANISMAL REPRODUCTIVE PROCESS%GOBP%GO:0048609	0.29386038	1.0576829	250	0.9051252	9321
REGULATION OF POTASSIUM ION TRANSMEMBRANE TRANSPORT%GOBP%GO:1901379	0.4072885	1.3451239	60	0.90512675	4230
CELLULAR RESPONSE TO DECREASED OXYGEN LEVELS%GOBP%GO:0036294	0.30284494	1.0564033	134	0.9051336	7033
TRAF6-MEDIATED INDUCTION OF TAK1 COMPLEX WITHIN TLR4 COMPLEX%REACTOME DATABASE ID RELEASE 75%937072	0.3746631	1.0304724	15	0.90514576	13438
CELLULAR RESPONSE TO HEAT%GOBP%GO:0034605	0.33763763	1.0613855	37	0.9051503	6350
PROTEIN TRANSPORT WITHIN PLASMA MEMBRANE%GOBP%GO:0099632	0.36733976	1.0661638	19	0.90515834	12135
PHOSPHOLIPASES%HUMANCYC%LIPASYN-PWY	0.5040807	1.5436273	29	0.9051667	5543
COLUMNAR/CUBOIDAL EPITHELIAL CELL DIFFERENTIATION%GOBP%GO:0002065	0.3308455	1.0257562	32	0.90518147	6955
HISTAMINE H2 RECEPTOR MEDIATED SIGNALING PATHWAY%PANTHER PATHWAY%P04386	0.35737532	1.0587581	22	0.9052115	6094
ACTIVATION	0.33561414	1.0266012	30	0.9052145	5601
REGULATION OF TRANSFORMING GROWTH FACTOR BETA RECEPTOR SIGNALING PATHWAY%GOBP%GO:0017015	0.30072346	1.0361799	95	0.9052184	7534
MYD88:MAL(TIRAP) CASCADE INITIATED ON PLASMA MEMBRANE%REACTOME DATABASE ID RELEASE 75%166058	0.3267905	1.1170685	89	0.90523094	10539
METABOTROPIC GLUTAMATE RECEPTOR GROUP I PATHWAY%PANTHER PATHWAY%P00041	0.3494846	1.0309367	21	0.9052539	5700
MEIOTIC CELL CYCLE%GOBP%GO:0051321	0.3074994	1.0577426	110	0.90527487	4572
POSITIVE REGULATION OF MITOTIC SISTER CHROMATID SEGREGATION%GOBP%GO:0062033	0.3568017	1.0575387	23	0.905285	6680
AMPLIFICATION OF SIGNAL FROM THE KINETOCHORES%REACTOME DATABASE ID RELEASE 75%141424	0.3121913	1.0570474	90	0.9052909	6033
NEGATIVE REGULATION OF HYDROLASE ACTIVITY%GOBP%GO:0051346	0.28820756	1.0344075	230	0.90530103	7012
NEUROINFLAMMATORY RESPONSE%GOBP%GO:0150076	0.37715006	1.056752	17	0.90530324	1440
NEGATIVE REGULATION OF MUSCLE CELL DIFFERENTIATION%GOBP%GO:0051148	0.338319	1.0574374	35	0.9053034	5911
REGULATION OF PHOSPHATIDYLINOSITOL 3-KINASE SIGNALING%GOBP%GO:0014066	0.32311383	1.110273	90	0.9053047	9785
MYOBLAST DIFFERENTIATION%GOBP%GO:0045445	0.3831744	1.1096218	21	0.9053079	3763
CONTRACTILE ACTIN FILAMENT BUNDLE ASSEMBLY%GOBP%GO:0030038	0.3881429	1.056648	15	0.9053139	4069
O-LINKED GLYCOSYLATION OF MUCINS%REACTOME%R-HSA-913709.3	0.32467702	1.0618395	49	0.90531546	6357
CELLULAR RESPONSE TO HYPOXIA%GOBP%GO:0071456	0.30926642	1.0881811	131	0.9053205	7033
REGULATION OF SUBSTRATE ADHESION-DEPENDENT CELL SPREADING%GOBP%GO:1900024	0.32603765	1.0310148	46	0.90532076	6992
REGULATION OF MUSCLE CELL DIFFERENTIATION%GOBP%GO:0051147	0.32441047	1.1078976	92	0.9053244	5911
METABOLISM OF SPINGOLIPIDS IN ER AND GOLGI APPARATUS%WIKIPATHWAYS_20210110%WP4142%HOMO SAPIENS	0.36010224	1.0311052	18	0.905335	5616
REGULATION OF CILIUM ASSEMBLY%GOBP%GO:1902017	0.30493683	1.0193998	67	0.905337	8336
TCF DEPENDENT SIGNALING IN RESPONSE TO WNT%REACTOME%R-HSA-201681.1	0.30027956	1.0662106	181	0.90537125	6261
CELLULAR RESPONSE TO KETONE%GOBP%GO:1901655	0.3469386	1.1103604	39	0.90538585	4179
PPARA ACTIVATES GENE EXPRESSION%REACTOME DATABASE ID RELEASE 75%1989781	0.30771345	1.0617208	108	0.90539795	7464
POSITIVE REGULATION OF CYTOSOLIC CALCIUM ION CONCENTRATION%GOBP%GO:0007204	0.29205114	1.0206317	142	0.90540814	9770
ERBB2 SIGNALING PATHWAY%GOBP%GO:0038128	0.35875037	1.1073502	30	0.9054229	10165
OXIDATION BY CYTOCHROME P450%WIKIPATHWAYS_20210110%WP43%HOMO SAPIENS	0.35605615	1.065393	27	0.90542316	3948
AUTOPHAGY%REACTOME%R-HSA-9612973.2	0.29154915	1.017573	125	0.9054238	8531
NUCLEUS LOCALIZATION%GOBP%GO:0051647	0.38314083	1.0615102	15	0.90544486	9725
ION TRANSPORT BY P-TYPE ATPASES%REACTOME%R-HSA-936837.3	0.33520585	1.0882505	49	0.9054609	4139
NEGATIVE REGULATION OF T CELL PROLIFERATION%GOBP%GO:0042130	0.3491211	1.0655824	25	0.9054619	5634
MORPHOGENESIS OF EMBRYONIC EPITHELIUM%GOBP%GO:0016331	0.32777905	1.0654818	51	0.9054677	7112
PKA-MEDIATED PHOSPHORYLATION OF CREB%REACTOME%R-HSA-111931.1	0.38229546	1.1085066	19	0.9054769	2831
AXON GUIDANCE MEDIATED BY SLIT ROBO%PANTHER PATHWAY%P00008	0.3693673	1.0662798	21	0.9054837	3536
BY HDAC CLASS I	0.31431153	1.0346506	63	0.9055151	3678
BIOCARTA_AKT_PATHWAY%MSIGDB_C2%BIOCARTA_AKT_PATHWAY	0.48108158	1.3506229	16	0.9055258	4048
CENTROSOME MATURATION%REACTOME DATABASE ID RELEASE 75%380287	0.3140905	1.0577636	80	0.90556014	9743
REGULATION OF EPITHELIAL CELL APOPTOTIC PROCESS%GOBP%GO:1904035	0.312405	1.0194222	48	0.90558505	10960
LEUKOCYTE MIGRATION%GOBP%GO:0050900	0.31270513	1.110736	182	0.9055986	5609
LYTIC VACUOLE ORGANIZATION%GOBP%GO:0080171	0.32275772	1.0344142	50	0.9056091	8796
NUCLEOTIDE-EXCISION REPAIR, PREINCISION COMPLEX STABILIZATION%GOBP%GO:0006293	0.37210754	1.0886353	21	0.9056101	13964
MITOCHONDRIAL OUTER MEMBRANE PERMEABILIZATION INVOLVED IN PROGRAMMED CELL DEATH%GOBP%GO:1902686	0.39518476	1.1099886	17	0.90561265	9027
AMEBOIDAL-TYPE CELL MIGRATION%GOBP%GO:0001667	0.3008034	1.0345141	110	0.9056167	7653
COGNITION%GOBP%GO:0050890	0.3052035	1.0700865	138	0.90564317	7644
BIOCARTA_HER2_PATHWAY%MSIGDB_C2%BIOCARTA_HER2_PATHWAY	0.35525116	1.0311095	21	0.90564954	10165
INTERACTIONS	0.30471024	1.0180637	62	0.9056563	9328
POSITIVE REGULATION OF POSITIVE CHEMOTAXIS%GOBP%GO:0050927	0.3756701	1.0348028	17	0.9056639	7352
PULMONARY VALVE DEVELOPMENT%GOBP%GO:0003177	0.3608163	1.0346984	18	0.90568686	15132
REGULATION OF ADAPTIVE IMMUNE RESPONSE BASED ON SOMATIC RECOMBINATION OF IMMUNE RECEPTORS BUILT FROM IMMUNOGLOBULIN SUPERFAMILY DOMAINS%GOBP%GO:0002822	0.30382088	1.0253106	79	0.90569454	6556
REGULATION OF HIPPO SIGNALING%GOBP%GO:0035330	0.38214558	1.1096369	19	0.9057046	10494
SIGNALING BY FGFR4%REACTOME DATABASE ID RELEASE 75%5654743	0.3331801	1.0175811	30	0.90571004	13503
ACYLGLYCEROL HOMEOSTASIS%GOBP%GO:0055090	0.38188827	1.0580134	16	0.9057111	9399
POSITIVE REGULATION OF INTERLEUKIN-2 PRODUCTION%GOBP%GO:0032743	0.3760474	1.0584204	18	0.9057144	7226
CERAMIDE CATABOLIC PROCESS%GOBP%GO:0046514	0.3735346	1.0206327	15	0.90572184	6316
REGULATION OF GENE EXPRESSION, EPIGENETIC%GOBP%GO:0040029	0.28740275	1.0254953	195	0.90572494	8874
CDK-MEDIATED PHOSPHORYLATION AND REMOVAL OF CDC6%REACTOME%R-HSA-69017.2	0.3315592	1.1079118	71	0.9057268	8898
REGULATION OF MORPHOGENESIS OF A BRANCHING STRUCTURE%GOBP%GO:0060688	0.34670827	1.0198493	23	0.9057377	7094
CELLULAR RESPONSE TO HYPOXIA%REACTOME%R-HSA-1234174.2	0.33226058	1.1103846	72	0.905748	9541
PID_EPHB_FWD_PATHWAY%MSIGDB_C2%PID_EPHB_FWD_PATHWAY	0.32609186	1.0203276	34	0.9057571	11619
NIK-->NONCANONICAL NF-KB SIGNALING%REACTOME%R-HSA-5676590.1	0.32353485	1.0578078	57	0.9057576	8770
ESTABLISHMENT OR MAINTENANCE OF EPITHELIAL CELL APICAL/BASAL POLARITY%GOBP%GO:0045197	0.34490314	1.0615244	32	0.90576136	1896
G2 M TRANSITION%REACTOME DATABASE ID RELEASE 75%69275	0.31262547	1.1108103	179	0.9057627	9513
POSITIVE REGULATION OF TRANSCRIPTION INITIATION FROM RNA POLYMERASE II PROMOTER%GOBP%GO:0060261	0.3799097	1.1100564	21	0.9057696	6040
MUSCLE TISSUE MORPHOGENESIS%GOBP%GO:0060415	0.34079707	1.088278	40	0.90577734	7588
ALPHA-SYNUCLEIN SIGNALING%PATHWAY INTERACTION DATABASE NCI-NATURE CURATED DATA%ALPHA-SYNUCLEIN SIGNALING	0.33462268	1.0173666	26	0.9058021	3205
REGULATION OF ANION TRANSMEMBRANE TRANSPORT%GOBP%GO:1903959	0.3622225	1.0578923	24	0.90580285	8214
EGF RECEPTOR SIGNALING PATHWAY%PANTHER PATHWAY%P00018	0.29728526	1.0318391	121	0.90580535	7831
PID_HDAC_CLASSI_PATHWAY%MSIGDB_C2%PID_HDAC_CLASSI_PATHWAY	0.32360524	1.0655911	56	0.905807	5783
MODULATION OF TRANSCRIPTION IN OTHER ORGANISM INVOLVED IN SYMBIOTIC INTERACTION%GOBP%GO:0052312	0.34644926	1.0182102	22	0.9058073	5648
PROTEIN COMPLEX OLIGOMERIZATION%GOBP%GO:0051259	0.30299324	1.0662946	140	0.9058076	5883
RESPONSE TO INTERLEUKIN-1%GOBP%GO:0070555	0.3018427	1.0656886	148	0.905816	5609
ASPARTATE FAMILY AMINO ACID METABOLIC PROCESS%GOBP%GO:0009066	0.31619185	1.0194453	42	0.9058183	4806
REGULATION OF MRNA PROCESSING%GOBP%GO:0050684	0.2890885	1.0182995	134	0.9058242	4224
REGULATION OF CELLULAR PROTEIN CATABOLIC PROCESS%GOBP%GO:1903362	0.29918605	1.0664976	215	0.90582937	6536
HUNTINGTON DISEASE%PANTHER PATHWAY%P00029	0.31584504	1.087847	117	0.90583575	10822
LIPOPROTEIN METABOLISM%REACTOME%R-HSA-174824.4	0.31484032	1.0253594	49	0.9058519	8625
BLOOD CIRCULATION%GOBP%GO:0008015	0.29731554	1.0582746	203	0.9058771	7644
DEMETHYLATION%GOBP%GO:0070988	0.31456655	1.0244968	48	0.9058835	8790
BIOCARTA_CARM_ER_PATHWAY%MSIGDB_C2%BIOCARTA_CARM_ER_PATHWAY	0.35234666	1.0247841	22	0.90588576	1202
ANTIGEN PROCESSING-CROSS PRESENTATION%REACTOME%R-HSA-1236975.1	0.32246616	1.1019876	88	0.90588593	8770
CHROMOSOME LOCALIZATION%GOBP%GO:0050000	0.30791768	1.0311337	65	0.90589744	9477
NEPHRON TUBULE DEVELOPMENT%GOBP%GO:0072080	0.33003518	1.0206732	34	0.9058978	7674
PHOSPHOLIPASE C-ACTIVATING G PROTEIN-COUPLED RECEPTOR SIGNALING PATHWAY%GOBP%GO:0007200	0.33575657	1.0886736	48	0.9058989	6707
SWITCHING OF ORIGINS TO A POST-REPLICATIVE STATE%REACTOME DATABASE ID RELEASE 75%69052	0.31920758	1.1021942	89	0.9058993	8898
HISTONE H3 ACETYLATION%GOBP%GO:0043966	0.41990063	1.3467454	40	0.905907	4949
TGF-BETA SIGNALING PATHWAY%WIKIPATHWAYS_20210110%WP366%HOMO SAPIENS	0.29596555	1.0176178	100	0.90590906	10926
GPCRS, CLASS A RHODOPSIN-LIKE%WIKIPATHWAYS_20210110%WP455%HOMO SAPIENS	0.3121236	1.0672727	95	0.9059159	8207
SAPIENS	0.32633525	1.0889703	73	0.90591794	4065
NEGATIVE REGULATION OF BINDING%GOBP%GO:0051100	0.3105495	1.0890789	111	0.9059212	4209
HUMORAL IMMUNE RESPONSE%GOBP%GO:0006959	0.3213819	1.1104515	101	0.9059241	5991
PID_AURORA_A_PATHWAY%MSIGDB_C2%PID_AURORA_A_PATHWAY	0.3733754	1.0887645	21	0.9059286	8490
LAMELLIPODIUM ASSEMBLY%GOBP%GO:0030032	0.35846832	1.0677713	25	0.905939	6859
MAPK CASCADE%WIKIPATHWAYS_20210110%WP422%HOMO SAPIENS	0.3647106	1.1080785	29	0.90594184	8109
REGULATION OF HORMONE BIOSYNTHETIC PROCESS%GOBP%GO:0046885	0.35271522	1.0180715	19	0.90594554	13836
RESPONSE TO DSRNA%GOBP%GO:0043331	0.34743282	1.034818	22	0.9059477	3562
REGULATION OF MYOBLAST DIFFERENTIATION%GOBP%GO:0045661	0.3686712	1.1105502	27	0.905952	10532
CELL RECOGNITION%GOBP%GO:0008037	0.30983075	1.0321846	71	0.905953	10079
TOLL LIKE RECEPTOR 3 (TLR3) CASCADE%REACTOME%R-HSA-168164.3	0.31405956	1.0707067	90	0.9059539	4266
REGULATION OF STEM CELL DIFFERENTIATION%GOBP%GO:2000736	0.3181862	1.0884444	98	0.9059636	9483
POSITIVE REGULATION OF ADAPTIVE IMMUNE RESPONSE BASED ON SOMATIC RECOMBINATION OF IMMUNE RECEPTORS BUILT FROM IMMUNOGLOBULIN SUPERFAMILY DOMAINS%GOBP%GO:0002824	0.30945706	1.0198765	53	0.90596664	6556
FBXL7 DOWN-REGULATES AURKA DURING MITOTIC ENTRY AND IN EARLY MITOSIS%REACTOME%R-HSA-8854050.2	0.33941758	1.1096828	53	0.9059684	8770
POSITIVE REGULATION OF INTERLEUKIN-12 PRODUCTION%GOBP%GO:0032735	0.38290974	1.109795	21	0.90596986	1994
DETECTION OF CHEMICAL STIMULUS INVOLVED IN SENSORY PERCEPTION OF TASTE%GOBP%GO:0050912	0.36857337	1.0194912	15	0.9059844	3103
BIOCARTA_RAS_PATHWAY%MSIGDB_C2%BIOCARTA_RAS_PATHWAY	0.38515946	1.0700953	16	0.9060001	12347
REGULATION OF GLUCOKINASE BY GLUCOKINASE REGULATORY PROTEIN%REACTOME%R-HSA-170822.3	0.33414623	1.0250336	29	0.90600055	5123
BARBED-END ACTIN FILAMENT CAPPING%GOBP%GO:0051016	0.38803145	1.0875998	17	0.90600425	7948
POSITIVE REGULATION OF ION TRANSPORT%GOBP%GO:0043270	0.28759447	1.0226542	161	0.90600485	11077
MITOCHONDRIAL TRANSPORT%GOBP%GO:0006839	0.30541292	1.0876977	197	0.9060075	9206
RORA ACTIVATES GENE EXPRESSION%REACTOME DATABASE ID RELEASE 75%1368082	0.36074376	1.0319729	18	0.90601015	8128

POSITIVE REGULATION OF GENE SILENCING BY MIRNA%GOBP%GO:2000637	0.33518812	1.0246527	27	0.90601826	8490
PID_CD8_TCR_DOWNSTREAM_PATHWAY%MSIGDB_C2%PID_CD8_TCR_DOWNSTREAM_PATHWAY	0.33528417	1.0580276	33	0.90602773	11091
POSITIVE REGULATION OF ESTABLISHMENT OF PROTEIN LOCALIZATION%GOBP%GO:1904951	0.28245977	1.0195745	237	0.9060348	8992
CILIUM-DEPENDENT CELL MOTILITY%GOBP%GO:0060285	0.36897534	1.0318707	18	0.9060363	5064
IL-4 SIGNALING PATHWAY%WIKIPATHWAYS_20210110%WP395%HOMO SAPIENS	0.31440255	1.0183303	45	0.9060396	4770
NEGATIVE REGULATION OF CYTOKINE PRODUCTION%GOBP%GO:0001818	0.29249802	1.0348809	167	0.9060526	7014
SIRT1 NEGATIVELY REGULATES RRNA EXPRESSION%REACTOME DATABASE ID RELEASE 75%427359	0.35034874	1.0680475	27	0.9060597	5210
PATTERN SPECIFICATION PROCESS%GOBP%GO:0007389	0.28788766	1.0245398	192	0.9060663	10917
CHAPERONIN-MEDIATED PROTEIN FOLDING%REACTOME DATABASE ID RELEASE 75%390466	0.32159778	1.0883104	84	0.9060726	6228
CNS%WIKIPATHWAYS_20210110%WP4304%HOMO SAPIENS	0.37019712	1.089243	22	0.9060811	7094
TRANSPORT OF MATURE TRANSCRIPT TO CYTOPLASM%REACTOME%R-HSA-72202.2	0.31933707	1.0673306	79	0.90608156	5209
KERATAN SULFATE METABOLIC PROCESS%GOBP%GO:0042339	0.34859523	1.0696582	31	0.9060817	1292
REGULATION OF PRODUCTION OF MOLECULAR MEDIATOR OF IMMUNE RESPONSE%GOBP%GO:0002700	0.3036471	1.017661	73	0.9060844	6810
GLOMERULAR EPITHELIUM DEVELOPMENT%GOBP%GO:0072010	0.396481	1.0893431	15	0.90609753	6684
LPA4-MEDIATED SIGNALING EVENTS%PATHWAY INTERACTION DATABASE NCI-NATURE CURATED DATA%LPA4-MEDIATED SIGNALING EVENTS	0.39180392	1.066317	15	0.9061018	2831
TRANSPORT TO THE GOLGI AND SUBSEQUENT MODIFICATION%REACTOME%R-HSA-948021.2	0.28765923	1.0200264	162	0.90610987	6101
REGULATION OF KERATINOCYTE DIFFERENTIATION%GOBP%GO:0045616	0.3307712	1.0248086	28	0.90612227	6684
NEGATIVE REGULATION OF HORMONE SECRETION%GOBP%GO:0046888	0.3310877	1.0178325	27	0.9061253	13509
REGULATION OF ENDOTHELIAL CELL DIFFERENTIATION%GOBP%GO:0045601	0.49754518	1.507206	26	0.90612596	2901
HEXOSE CATABOLIC PROCESS%GOBP%GO:0019320	0.41888767	1.3452532	44	0.9061398	5258
REGULATION OF HISTONE METHYLATION%GOBP%GO:0031060	0.32110453	1.0177375	41	0.90613997	2517
REGULATION OF LIPID KINASE ACTIVITY%GOBP%GO:0043550	0.32450098	1.0206932	37	0.90615356	7282
POSITIVE REGULATION OF IMMUNOGLOBULIN MEDIATED IMMUNE RESPONSE%GOBP%GO:0002891	0.39469635	1.1079189	18	0.9061546	6556
RNA POLYADENYLATION%GOBP%GO:0043631	0.3583681	1.0872494	30	0.9061589	2859
REGULATION OF NEURONAL SYNAPTIC PLASTICITY%GOBP%GO:0048168	0.330799	1.0248941	30	0.90616167	7676
S1P2 PATHWAY%PATHWAY INTERACTION DATABASE NCI-NATURE CURATED DATA%S1P2 PATHWAY	0.35033575	1.0358088	23	0.90616626	11345
NUCLEAR TRANSPORT%GOBP%GO:0051169	0.2975942	1.0656964	217	0.9061679	5437
CYTOKINETIC PROCESS%GOBP%GO:0032506	0.34984502	1.0878652	33	0.9061704	3370
SEALING OF THE NUCLEAR ENVELOPE (NE) BY ESCRT-III%REACTOME%R-HSA-9668328.1	0.35717842	1.0866412	26	0.9061734	5887
HALLMARK_APICAL_JUNCTION%MSIGDB_C2%HALLMARK_APICAL_JUNCTION	0.29432738	1.0322179	135	0.90617377	7759
NEGATIVE REGULATION OF CELL-MATRIX ADHESION%GOBP%GO:0001953	0.3352584	1.031147	30	0.9061841	3189
RESPONSE TO XENOBIOTIC STIMULUS%GOBP%GO:0009410	0.31533143	1.0894263	98	0.90618426	5320
NEGATIVE REGULATION OF ERK1 AND ERK2 CASCADE%GOBP%GO:0070373	0.32349157	1.0678034	47	0.9061901	5073
SOMATIC DIVERSIFICATION OF IMMUNE RECEPTORS VIA GERMLINE RECOMBINATION WITHIN A SINGLE LOCUS%GOBP%GO:0002562	0.5366421	1.5088958	18	0.9062022	6061
POSITIVE REGULATION OF STRIATED MUSCLE CELL DIFFERENTIATION%GOBP%GO:0051155	0.34508538	1.019618	24	0.90620416	13205
AMINE CATABOLIC PROCESS%GOBP%GO:0009310	0.38041642	1.066499	17	0.9062064	9860
NEGATIVE REGULATION OF INSULIN SECRETION%GOBP%GO:0046676	0.39853784	1.0891097	15	0.90620714	11345
NEGATIVE REGULATION OF PROTEIN BINDING%GOBP%GO:0032091	0.31024992	1.0320109	73	0.90621173	5295
REGULATION OF NEUROINFLAMMATORY RESPONSE%GOBP%GO:0150077	0.35277078	1.0183735	20	0.90621233	3943
PHOSPHATIDIC ACID METABOLIC PROCESS%GOBP%GO:0046473	0.32312158	1.0349296	42	0.90621275	5174
PROTEIN KINASE B SIGNALING%GOBP%GO:0043491	0.36188424	1.0580751	22	0.906217	2885
NEGATIVE REGULATION OF DNA RECOMBINATION%GOBP%GO:0045910	0.32646284	1.0250659	32	0.906217	7857
HISTONE H4-K16 ACETYLATION%GOBP%GO:0043984	0.35500005	1.0200851	20	0.9062246	9289
NETRIN-UNC5B SIGNALING PATHWAY%WIKIPATHWAYS_20210110%WP4747%HOMO SAPIENS	0.3530992	1.102012	34	0.90622705	7759
UBIQUITIN MEDIATED DEGRADATION OF PHOSPHORYLATED CDC25A%REACTOME%R-HSA-69601.3	0.3323136	1.0868291	50	0.9062338	8770
POSITIVE REGULATION OF TORC1 SIGNALING%GOBP%GO:1904263	0.38432705	1.0697228	16	0.90623426	8759
ANTIGEN PROCESSING AND PRESENTATION OF EXOGENOUS PEPTIDE ANTIGEN VIA MHC CLASS I, TAP-DEPENDENT%GOBP%GO:0002479	0.30751127	1.02268	67	0.9062379	8677
POSITIVE REGULATION OF CALCIUM ION-DEPENDENT EXOCYTOSIS%GOBP%GO:0045956	0.3730431	1.0356896	16	0.90624595	4587
AUTODEGRADATION OF THE E3 UBIQUITIN LIGASE COP1%REACTOME DATABASE ID RELEASE 75%349425	0.32949236	1.0703217	50	0.9062488	8770
NEGATIVE REGULATION OF DEPHOSPHORYLATION%GOBP%GO:0035305	0.3140019	1.0680969	76	0.9062515	7435
REGULATION OF MICROTUBULE POLYMERIZATION OR DEPOLYMERIZATION%GOBP%GO:0031110	0.30321968	1.0198799	73	0.90627205	10936
SMAD2 3 SIGNALING	0.32404977	1.0887812	75	0.90628004	5951
AMPLIFICATION OF SIGNAL FROM UNATTACHED KINETOCHORES VIA A MAD2 INHIBITORY SIGNAL%REACTOME%R-HSA-141444.2	0.3121913	1.0683862	90	0.906281	6033
POSITIVE REGULATION OF INSULIN SECRETION%GOBP%GO:0032024	0.34158534	1.0670774	33	0.9062815	11374
DNA METHYLATION%GOBP%GO:0006306	0.3613875	1.102209	29	0.9062837	3947
MEDIATED BY VEGFR1 AND VEGFR2	0.32675812	1.0707191	63	0.90629804	4523
INTERFERON-GAMMA-MEDIATED SIGNALING PATHWAY%GOBP%GO:0060333	0.41038153	1.3508123	58	0.9063	8169
DEVELOPMENT AND HETEROGENEITY OF THE ILC FAMILY%WIKIPATHWAYS_20210110%WP3893%HOMO SAPIENS	0.37207285	1.0674713	19	0.9063149	8102
MITOTIC METAPHASE AND ANAPHASE%REACTOME%R-HSA-2555396.2	0.29650962	1.0657492	219	0.9063427	6033
REGULATION OF STEM CELL PROLIFERATION%GOBP%GO:0072091	0.341593	1.070105	35	0.90635085	6438
INTERLEUKIN-3, INTERLEUKIN-5 AND GM-CSF SIGNALING%REACTOME DATABASE ID RELEASE 75%512988	0.34351143	1.069794	33	0.90636086	4268
SENSORY PERCEPTION OF PAIN%GOBP%GO:0019233	0.3579495	1.07039	23	0.90637743	10864
ANTIGEN PROCESSING AND PRESENTATION OF EXOGENOUS ANTIGEN%GOBP%GO:0019884	0.3023331	1.0681562	160	0.9063938	11127
TRAIL%IOB%TRAIL	0.33672607	1.0868906	44	0.9064015	5356
REGULATION OF EXTRINSIC APOPTOTIC SIGNALING PATHWAY IN ABSENCE OF LIGAND%GOBP%GO:2001239	0.33944362	1.0227244	23	0.906409	6752
PROTEIN FOLDING%REACTOME%R-HSA-391251.1	0.32423273	1.0896815	90	0.9064126	6228
POSITIVE REGULATION OF INFLAMMATORY RESPONSE%GOBP%GO:0050729	0.32162187	1.0872921	78	0.9064169	4681
MAPK PATHWAY IN CONGENITAL THYROID CANCER%WIKIPATHWAYS_20210110%WP4928%HOMO SAPIENS	0.38482422	1.0709859	15	0.9064235	11091
NEGATIVE REGULATION OF CELLULAR COMPONENT MOVEMENT%GOBP%GO:0051271	0.28844854	1.0322421	233	0.906424	7616
RESPONSE TO NUTRIENT LEVELS%GOBP%GO:0031667	0.30216783	1.0707827	219	0.9064356	7178
ESTABLISHMENT OF MITOTIC SPINDLE LOCALIZATION%GOBP%GO:0040001	0.3532677	1.0673354	26	0.90644664	5097
VENTRICULAR CARDIAC MUSCLE TISSUE DEVELOPMENT%GOBP%GO:0003229	0.33015886	1.0170788	31	0.90644735	7588
PEPTIDYL-LYSINE MODIFICATION%GOBP%GO:0018205	0.2870944	1.0329127	234	0.9064605	4997
REGULATION OF TELOMERE CAPPING%GOBP%GO:1904353	0.34380075	1.0323268	25	0.9064625	12603
POSITIVE REGULATION OF MRNA CATABOLIC PROCESS%GOBP%GO:0061014	0.3146168	1.0169787	46	0.90646595	6970
DEVELOPMENTAL PROCESS INVOLVED IN REPRODUCTION%GOBP%GO:0003006	0.28790572	1.0206935	209	0.9064719	9729
NEGATIVE REGULATION OF TRANSMEMBRANE TRANSPORT%GOBP%GO:0034763	0.32449597	1.0897713	76	0.90647715	3787
CENTROSOME CYCLE%GOBP%GO:0007098	0.32085103	1.0866644	77	0.9064837	3446
RIBONUCLEOPROTEIN COMPLEX ASSEMBLY%GOBP%GO:0022618	0.29069743	1.0354234	206	0.90648514	6659
MEMBRANE DEPOLARIZATION%GOBP%GO:0051899	0.3253793	1.0324141	43	0.906491	9484
REGULATION OF PRI-MIRNA TRANSCRIPTION BY RNA POLYMERASE II%GOBP%GO:1902893	0.32830706	1.0311488	37	0.9065093	6773
MITOTIC NUCLEAR DIVISION%GOBP%GO:0140014	0.29419592	1.0326071	143	0.90651	12942
RESPONSE TO HEAT%GOBP%GO:0009408	0.31376627	1.0349393	53	0.90651685	5721
REGULATION OF TYPE I INTERFERON PRODUCTION%GOBP%GO:0032479	0.29748777	1.0312419	103	0.90652317	7658
STEM CELL DEVELOPMENT%GOBP%GO:0048864	0.33495906	1.0894421	49	0.9065384	9324
NEGATIVE REGULATION OF TORC1 SIGNALING%GOBP%GO:1904262	0.3867541	1.0675125	15	0.90654045	11724
REGULATION OF CELL ADHESION MEDIATED BY INTEGRIN%GOBP%GO:0033628	0.33947358	1.0685246	33	0.9065405	4446
POSITIVE REGULATION OF OSTEOBLAST DIFFERENTIATION%GOBP%GO:0045669	0.333269	1.0678096	40	0.9065501	10779
PROTEIN DEMANNOSYLATION%GOBP%GO:0036507	0.35830492	1.032691	20	0.90655065	7642
POSITIVE REGULATION OF OXIDOREDUCTASE ACTIVITY%GOBP%GO:0051353	0.34088287	1.0665087	35	0.90655303	10454
POSITIVE REGULATION OF ENDOTHELIAL CELL PROLIFERATION%GOBP%GO:0001938	0.31993756	1.0684172	69	0.9065632	9804
POSITIVE REGULATION OF COFACTOR METABOLIC PROCESS%GOBP%GO:0051194	0.3395311	1.0227715	22	0.9065715	2885
CALCIUM ION HOMEOSTASIS%GOBP%GO:0055074	0.30012006	1.0704341	246	0.90659654	4638
RESPONSE TO HYPOXIA%GOBP%GO:0001666	0.302222	1.0682057	187	0.90661335	6322
POSITIVE REGULATION OF PROTEIN IMPORT INTO NUCLEUS%GOBP%GO:0042307	0.35771972	1.0665925	26	0.90661776	11324
MYD88-INDEPENDENT TLR4 CASCADE%REACTOME DATABASE ID RELEASE 75%166166	0.30571842	1.0701349	94	0.90661913	8274
FOLATE METABOLISM%WIKIPATHWAYS_20210110%WP176%HOMO SAPIENS	0.31880572	1.0207404	39	0.9066324	13130
POSITIVE REGULATION OF DEFENSE RESPONSE TO VIRUS BY HOST%GOBP%GO:0002230	0.46621126	1.345463	20	0.9066536	3562
PROTEIN IMPORT%GOBP%GO:0017038	0.3119487	1.0869328	121	0.9066558	6387
ATP SYNTHESIS COUPLED ELECTRON TRANSPORT%GOBP%GO:0042773	0.39819738	1.3472164	82	0.90667117	4191
HH MUTANTS ARE DEGRADED BY ERAD%REACTOME%R-HSA-5362768.2	0.31064087	1.0228378	54	0.906672	8770
REGULATION OF VASOCONSTRICTION%GOBP%GO:0019229	0.45740953	1.346926	24	0.90668565	6031
NEGATIVE REGULATION OF LEUKOCYTE PROLIFERATION%GOBP%GO:0070664	0.34307563	1.0708152	34	0.90669435	13890
STAT CASCADE%GOBP%GO:0097696	0.34876704	1.0710104	30	0.9067203	5843
REGULATION OF INTRACELLULAR ESTROGEN RECEPTOR SIGNALING PATHWAY%GOBP%GO:0033146	0.35497475	1.0873164	30	0.9067373	11365
REGULATION OF PLASMA MEMBRANE ORGANIZATION%GOBP%GO:1903729	0.396876	1.0894978	15	0.90674466	4865
SENSORY PERCEPTION OF TASTE%GOBP%GO:0050909	0.35007	1.0223323	21	0.9067587	3688
PID_LYSOPHOSPHOLIPID_PATHWAY%MSIGDB_C2%PID_LYSOPHOSPHOLIPID_PATHWAY	0.3165819	1.0324343	44	0.906759	3663
GLUCOSE 6-PHOSPHATE METABOLIC PROCESS%GOBP%GO:0051156	0.36476204	1.022904	18	0.9067624	3328
SYNTHESIS OF SUBSTRATES IN N-GLYCAN BIOSYTHESIS%REACTOME DATABASE ID RELEASE 75%446219	0.30930778	1.0327288	59	0.90676296	7151
SUDDEN INFANT DEATH SYNDROME (SIDS) SUSCEPTIBILITY PATHWAYS%WIKIPATHWAYS_20210110%WP706%HOMO SAPIENS	0.30028188	1.0352464	112	0.9067755	10344
OTHER SEMAPHORIN INTERACTIONS%REACTOME%R-HSA-416700.1	0.36576918	1.0312617	17	0.9067856	3657
PROTEIN LOCALIZATION TO CELL PERIPHERY%GOBP%GO:1990778	0.2998577	1.0685588	176	0.90680224	6935
RESPONSE TO VIRUS%GOBP%GO:0009615	0.29061207	1.0349528	180	0.906808	6592
FACTORS AND PATHWAYS AFFECTING INSULIN-LIKE GROWTH FACTOR (IGF1)-AKT SIGNALING%WIKIPATHWAYS_20210110%WP3850%HOMO SAPIENS	0.3337058	1.0130473	26	0.90680957	12989
NEGATIVE REGULATION OF CELLULAR AMIDE METABOLIC PROCESS%GOBP%GO:0034249	0.3044098	1.0666403	133	0.90681607	7030
POSITIVE REGULATION OF CELLULAR AMIDE METABOLIC PROCESS%GOBP%GO:0034250	0.29727286	1.03504	126	0.906818	8126
REGULATION OF ANIMAL ORGAN MORPHOGENESIS%GOBP%GO:2000027	0.29331386	1.035424	158	0.9068204	8248
RESPONSE TO CHEMOKINE%GOBP%GO:1990868	0.32544124	1.0133295	36	0.9068291	10945
POSITIVE REGULATION OF PLASMA MEMBRANE BOUNDED CELL PROJECTION ASSEMBLY%GOBP%GO:0120034	0.2994604	1.0137136	81	0.90683	8228
EGFR DOWNREGULATION%REACTOME%R-HSA-182971.6	0.35598707	1.0711797	28	0.9068374	8401
BMAL1:CLOCK,NPAS2 ACTIVATES CIRCADIAN GENE EXPRESSION%REACTOME%R-HSA-1368108.4	0.3592051	1.0897837	27	0.9068468	5893
HOST-PATHOGEN INTERACTION OF HUMAN CORONA VIRUSES - INTERFERON INDUCTION%WIKIPATHWAYS_20210110%WP4880%HOMO SAPIENS	0.34589902	1.0719824	32	0.9068481	10153
TOLL LIKE RECEPTOR 4 (TLR4) CASCADE%REACTOME DATABASE ID RELEASE 75%166016	0.31399065	1.0898894	112	0.90686464	4266
KIDNEY EPITHELIUM DEVELOPMENT%GOBP%GO:0072073	0.32435372	1.0667291	58	0.9068707	7728
CELLULAR RESPONSE TO INTERLEUKIN-6%GOBP%GO:0071354	0.3585145	1.071874	26	0.906881	3503
INORGANIC ANION TRANSMEMBRANE TRANSPORT%GOBP%GO:0098661	0.31099665	1.0232382	54	0.9068835	8506

NUCLEOTIDE EXCISION REPAIR%WIKIPATHWAYS_20210110%WP4753%HOMO SAPIENS	0.32855132	1.0314198	33	0.90690625	4361
GROWTH HORMONE RECEPTOR SIGNALING%REACTOME DATABASE ID RELEASE 75%982772	0.35221326	1.0167538	20	0.90691215	12211
EAR DEVELOPMENT%GOBP%GO:0043583	0.3091452	1.0207472	57	0.9069235	7112
DNA REPLICATION%GOBP%GO:0006260	0.2865861	1.0131054	166	0.9069334	8379
ANTIGEN PROCESSING AND PRESENTATION OF EXOGENOUS PEPTIDE ANTIGEN%GOBP%GO:0002478	0.30586684	1.0712556	159	0.9069341	11127
NEGATIVE REGULATION OF CYSTEINE-TYPE ENDOPEPTIDASE ACTIVITY INVOLVED IN APOPTOTIC PROCESS%GOBP%GO:0043154	0.31018007	1.022094	56	0.90693873	4689
NEGATIVE REGULATION OF VIRAL LIFE CYCLE%GOBP%GO:1903901	0.3086022	1.0229446	68	0.9069402	4483
NUCLEAR ENVELOPE (NE) REASSEMBLY%REACTOME DATABASE ID RELEASE 75%2995410	0.305501	1.0240846	64	0.90695256	5887
PID_ARF6_PATHWAY%MSIGDB_C2%PID_ARF6_PATHWAY	0.3721136	1.1015192	24	0.9069585	4816
RESPIRATORY SYSTEM DEVELOPMENT%GOBP%GO:0060541	0.3316112	1.0922596	55	0.9069714	5993
POSITIVE REGULATION OF NF-KAPPAB TRANSCRIPTION FACTOR ACTIVITY%GOBP%GO:0051092	0.29507944	1.0208223	131	0.9070039	8670
INTERLEUKIN-2 FAMILY SIGNALING%REACTOME%R-HSA-451927.5	0.32942462	1.0133713	31	0.90701014	5320
PROTEIN LOCALIZATION TO LYSOSOME%GOBP%GO:0061462	0.3412046	1.0869483	42	0.9070112	12297
STRESS-ACTIVATED PROTEIN KINASE SIGNALING CASCADE%GOBP%GO:0031098	0.2996407	1.0211126	95	0.9070113	3310
EXECUTION PHASE OF APOPTOSIS%GOBP%GO:0097194	0.3329249	1.0924519	43	0.9070201	10500
TRIACYLGLYCEROL BIOSYNTHESIS%HUMANCYC%TRIGLSYN-PWY	0.3458368	1.0209038	24	0.9070342	3637
REGULATION OF ACTIN DYNAMICS FOR PHAGOCYTIC CUP FORMATION%REACTOME DATABASE ID RELEASE 75%2029482	0.32412782	1.0710284	52	0.9070349	6350
REGULATION OF LIPID TRANSPORT%GOBP%GO:0032368	0.32847744	1.1013974	75	0.9070394	6246
GAP-FILLING DNA REPAIR SYNTHESIS AND LIGATION IN GG-NER%REACTOME%R-HSA-5696397.1	0.35952222	1.069303	25	0.9070398	3931
CHLORIDE TRANSMEMBRANE TRANSPORT%GOBP%GO:1902476	0.32021886	1.0165262	42	0.90704644	10168
MOLECULES ASSOCIATED WITH ELASTIC FIBRES%REACTOME DATABASE ID RELEASE 75%2129379	0.43725523	1.3539971	28	0.9070543	2566
TRNA PROCESSING IN THE NUCLEUS%REACTOME DATABASE ID RELEASE 75%6784531	0.33081543	1.092341	55	0.9070558	4994
REGULATION OF MEMBRANE REPOLARIZATION%GOBP%GO:0060306	0.34059173	1.0239494	26	0.9070574	5620
TRANSCRIPTION OF THE HIV GENOME%REACTOME DATABASE ID RELEASE 75%167172	0.32939744	1.0956925	66	0.9070632	6737
FUCOSYLATION%GOBP%GO:0036065	0.36938757	1.0221479	15	0.90707624	5235
HETEROTRIMERIC G-PROTEIN SIGNALING PATHWAY-GQ ALPHA AND GO ALPHA MEDIATED PATHWAY%PANTHER PATHWAY%P00027	0.29972506	1.0350634	106	0.90707684	9374
DNA REPLICATION PRE-INITIATION%REACTOME DATABASE ID RELEASE 75%69002	0.2968855	1.0137246	81	0.9071027	8770
MET IN TYPE 1 PAPILLARY RENAL CELL CARCINOMA%WIKIPATHWAYS_20210110%WP4205%HOMO SAPIENS	0.3241011	1.0312651	45	0.9071049	7719
CHROMOSOME SEGREGATION%GOBP%GO:0007059	0.29917878	1.0713114	209	0.90710527	8418
PROTEIN K11-LINKED UBIQUITINATION%GOBP%GO:0070979	0.33364806	1.016601	27	0.90711755	9837
DEFECTIVE TPR MAY CONFER SUSCEPTIBILITY TOWARDS THYROID PAPILLARY CARCINOMA (TPC)%REACTOME%R-HSA-5619107.2	0.33414623	1.012662	29	0.9071216	5123
REGULATION OF ANGIOGENESIS%GOBP%GO:0045765	0.29823938	1.0690778	216	0.9071395	7365
C4-DICARBOXYLATE TRANSPORT%GOBP%GO:0015740	0.38584772	1.0926385	17	0.90714395	592
HALLMARK_APICAL_SURFACE%MSIGDB_C2%HALLMARK_APICAL_SURFACE	0.32570693	1.013422	33	0.9071461	5139
RNA POLYMERASE I TRANSCRIPTION INITIATION%REACTOME DATABASE ID RELEASE 75%73762	0.42374864	1.3527728	44	0.90716696	6737
TRANSCRIPTIONAL REGULATION BY E2F6%REACTOME%R-HSA-8953750.2	0.3434664	1.0685627	34	0.9071756	5189
MEIOTIC CHROMOSOME SEGREGATION%GOBP%GO:0045132	0.33436123	1.0667467	41	0.9071806	11802
PPAR ALPHA PATHWAY%WIKIPATHWAYS_20210110%WP2878%HOMO SAPIENS	0.3606699	1.0131242	16	0.9071818	12555
MITOTIC ANAPHASE%REACTOME DATABASE ID RELEASE 75%68882	0.2977351	1.0723907	218	0.9071993	6033
DISEASES OF METABOLISM%REACTOME%R-HSA-5668914.3	0.28664404	1.0232388	215	0.90720254	8647
PARKIN-UBIQUITIN PROTEASOMAL SYSTEM PATHWAY%WIKIPATHWAYS_20210110%WP2359%HOMO SAPIENS	0.33525932	1.0691593	45	0.9072072	8185
REGULATION OF CELLULAR RESPONSE TO TRANSFORMING GROWTH FACTOR BETA STIMULUS%GOBP%GO:1903844	0.29753816	1.0230515	96	0.90720916	7534
NEGATIVE REGULATION OF LEUKOCYTE DIFFERENTIATION%GOBP%GO:1902106	0.30919492	1.0122467	49	0.90721416	6752
NEGATIVE REGULATION OF DNA BINDING%GOBP%GO:0043392	0.4352653	1.3494846	32	0.907215	3838
ER QUALITY CONTROL COMPARTMENT (ERQC)%REACTOME%R-HSA-901032.5	0.37197053	1.0818603	21	0.90721714	8274
AUF1 (HNRNP D0) BINDS AND DESTABILIZES MRNA%REACTOME%R-HSA-450408.3	0.3324911	1.0823693	52	0.90722203	8770
NEGATIVE REGULATION OF BLOOD VESSEL ENDOTHELIAL CELL MIGRATION%GOBP%GO:0043537	0.34871405	1.0719866	29	0.907226	4401
B CELL MEDIATED IMMUNITY%GOBP%GO:0019724	0.36653978	1.0822651	25	0.9072315	6379
PID_CERAMIDE_PATHWAY%MSIGDB_C2%PID_CERAMIDE_PATHWAY	0.34320208	1.0724837	34	0.90723366	4417
RIBONUCLEOSIDE METABOLIC PROCESS%GOBP%GO:0009119	0.3356416	1.0824622	48	0.9072349	5423
REGULATION OF BONE REMODELING%GOBP%GO:0046850	0.3303571	1.0127178	27	0.90723926	4523
HALLMARK_UNFOLDED_PROTEIN_RESPONSE%MSIGDB_C2%HALLMARK_UNFOLDED_PROTEIN_RESPONSE	0.31550348	1.0720803	87	0.9072534	1742
SIGNAL RELEASE FROM SYNAPSE%GOBP%GO:0099643	0.3259217	1.0898985	72	0.9072536	5759
PID_IL12_STAT4_PATHWAY%MSIGDB_C2%PID_IL12_STAT4_PATHWAY	0.3962946	1.0954398	17	0.90725935	4187
HEDGEHOG SIGNALING PATHWAY%PANTHER PATHWAY%P00025	0.35343048	1.0229461	20	0.90726	2385
CELLULAR RESPONSE TO CHEMOKINE%GOBP%GO:1990869	0.32544124	1.0211303	36	0.9072659	10945
MITOCHONDRION DISASSEMBLY%GOBP%GO:0061726	0.34890968	1.095541	38	0.90728	6527
ESTABLISHMENT OF VESICLE LOCALIZATION%GOBP%GO:0051650	0.30321538	1.0958471	204	0.9072835	9131
NEGATIVE REGULATION OF LIPID STORAGE%GOBP%GO:0010888	0.39912048	1.0941676	16	0.9072841	5269
SYNTHESIS OF DNA%REACTOME DATABASE ID RELEASE 75%69239	0.3129565	1.0827588	118	0.9073065	8898
POSITIVE REGULATION OF CELL CYCLE ARREST%GOBP%GO:0071158	0.30426112	1.0209135	74	0.9073293	5039
NEGATIVE REGULATION OF CYCLIN-DEPENDENT PROTEIN SERINE/THREONINE KINASE ACTIVITY%GOBP%GO:0045736	0.3580354	1.0137504	17	0.9073339	11925
DNA GEOMETRIC CHANGE%GOBP%GO:0032392	0.3064896	1.0140313	60	0.90734386	8274
TICAM1, RIP1-MEDIATED IKK COMPLEX RECRUITMENT%REACTOME DATABASE ID RELEASE 75%168927	0.38323218	1.0924755	19	0.907351	4048
WNT%IOB%WNT	0.31594342	1.0862348	86	0.9073571	7219
ACTIVATION OF MAPKK ACTIVITY%GOBP%GO:0000186	0.3360888	1.0232843	26	0.90736634	9510
POSITIVE REGULATION OF PEPTIDYL-TYROSINE PHOSPHORYLATION%GOBP%GO:0050731	0.32124662	1.1015247	108	0.907375	11057
MESONEPHROS DEVELOPMENT%GOBP%GO:0001823	0.3384785	1.0725434	34	0.9073882	7728
MESODERMAL COMMITMENT PATHWAY%WIKIPATHWAYS_20210110%WP2857%HOMO SAPIENS	0.31040815	1.0726373	109	0.9073973	7277
EXONUCLEOLYTIC NUCLEAR-TRANSCRIBED MRNA CATABOLIC PROCESS INVOLVED IN DEADENYLATION-DEPENDENT DECAY%GOBP%GO:0043928	0.3276596	1.0122807	32	0.90740824	9274
SEGMENTATION%GOBP%GO:0035282	0.5205625	1.5456418	22	0.9074135	7112
HOMOLOGOUS DNA PAIRING AND STRAND EXCHANGE%REACTOME DATABASE ID RELEASE 75%5693579	0.33262134	1.0727329	40	0.907428	12221
DNA REPLICATION%REACTOME DATABASE ID RELEASE 75%69306	0.3155394	1.0937146	124	0.90743226	8898
RESPONSE TO TUMOR NECROSIS FACTOR%GOBP%GO:0034612	0.30851486	1.0942355	174	0.9074331	5498
SIGNALING BY LIGAND-RESPONSIVE EGFR VARIANTS IN CANCER%REACTOME%R-HSA-5637815.1	0.35432774	1.0134288	17	0.90743625	10363
DEFECTS IN VITAMIN AND COFACTOR METABOLISM%REACTOME DATABASE ID RELEASE 75%3296482	0.35390523	1.0141957	20	0.9074385	8647
FEEDING BEHAVIOR%GOBP%GO:0007631	0.36610833	1.0685904	22	0.9074444	5546
SYNDECAN INTERACTIONS%REACTOME DATABASE ID RELEASE 75%3000170	0.35302696	1.023736	19	0.9074565	5243
GPI ANCHOR BIOSYNTHETIC PROCESS%GOBP%GO:0006506	0.32732624	1.0142837	31	0.90746707	7559
POSITIVE REGULATION OF ISOTYPE SWITCHING%GOBP%GO:0045830	0.38274094	1.0713156	16	0.9074767	6556
POSITIVE REGULATION OF ENDOPEPTIDASE ACTIVITY%GOBP%GO:0010950	0.3068065	1.0821112	137	0.9074781	6298
SIGNALING EVENTS MEDIATED BY TCPTP%PATHWAY INTERACTION DATABASE NCI-NATURE CURATED DATA%SIGNALING EVENTS MEDIATED BY TCPTP	0.3464207	1.0956968	39	0.9074792	2118
CELLULAR RESPONSE TO GLUCAGON STIMULUS%GOBP%GO:0071377	0.36752686	1.0127382	15	0.9074832	2831
DOUBLE-STRAND BREAK REPAIR%GOBP%GO:0006302	0.28587076	1.0123495	169	0.9074955	8413
PATHWAYS IN CLEAR CELL RENAL CELL CARCINOMA%WIKIPATHWAYS_20210110%WP4018%HOMO SAPIENS	0.33113104	1.0926561	57	0.9074971	8404
NEGATIVE REGULATION OF ESTABLISHMENT OF PROTEIN LOCALIZATION%GOBP%GO:1904950	0.3175458	1.0729164	80	0.90749735	5395
RHOA SIGNALING PATHWAY%PATHWAY INTERACTION DATABASE NCI-NATURE CURATED DATA%RHOA SIGNALING PATHWAY	0.34014097	1.0905725	44	0.9074988	3446
DEGRADATION OF GLI1 BY THE PROTEASOME%REACTOME%R-HSA-5610780.1	0.32297897	1.0715079	57	0.90750563	8770
REGULATION OF INTERLEUKIN-12 PRODUCTION%GOBP%GO:0032655	0.3290674	1.0138838	31	0.90751046	2160
CYTOSOLIC CALCIUM ION TRANSPORT%GOBP%GO:0060401	0.31220043	1.0211524	54	0.90751594	3251
POSITIVE REGULATION OF JAK-STAT CASCADE%GOBP%GO:0046427	0.3289947	1.0828103	48	0.90751827	3770
SYNAPTIC VESICLE CYCLE%GOBP%GO:0099504	0.31239516	1.0686711	80	0.90752894	5759
RESPONSE TO CORTICOSTEROID%GOBP%GO:0031960	0.36285052	1.095892	28	0.9075496	9383
WNT SIGNALING NETWORK%PATHWAY INTERACTION DATABASE NCI-NATURE CURATED DATA%WNT SIGNALING NETWORK	0.3596245	1.0825922	25	0.9075522	2211
POSITIVE REGULATION OF CELL MORPHOGENESIS INVOLVED IN DIFFERENTIATION%GOBP%GO:0010770	0.31053245	1.0721028	97	0.90755653	8141
GROWTH HORMONE RECEPTOR SIGNALING PATHWAY%GOBP%GO:0060396	0.37500983	1.0234249	15	0.907558	6812
CILIARY BASAL BODY-PLASMA MEMBRANE DOCKING%GOBP%GO:0097711	0.29552016	1.0137731	95	0.9075642	9746
POSITIVE REGULATION OF GENE EXPRESSION, EPIGENETIC%GOBP%GO:0045815	0.31061503	1.0120395	51	0.90756816	5684
CLASS C 3 (METABOTROPIC GLUTAMATE PHEROMONE RECEPTORS)%REACTOME DATABASE ID RELEASE 75%420499	0.39061725	1.09306	16	0.9075695	618
VISUAL PHOTOTRANSDUCTION%REACTOME%R-HSA-2187338.1	0.3265882	1.0914894	67	0.90757084	4570
POSITIVE REGULATION OF LEUKOCYTE CELL-CELL ADHESION%GOBP%GO:1903039	0.3076876	1.0688617	135	0.90758	5015
MEIOTIC NUCLEAR DIVISION%GOBP%GO:0140013	0.3019648	1.021803	82	0.9075811	10438
POSITIVE REGULATION OF MITOTIC NUCLEAR DIVISION%GOBP%GO:0045840	0.30748802	1.0140548	49	0.90758353	7911
SYNAPSE ORGANIZATION%GOBP%GO:0050808	0.30662498	1.0818671	172	0.90759546	3308
NEGATIVE REGULATION OF SMOOTHENED SIGNALING PATHWAY%GOBP%GO:0045879	0.36572275	1.0951352	24	0.9075982	4911
GLYCEROPHOSPHOLIPID BIOSYNTHETIC PATHWAY%WIKIPATHWAYS_20210110%WP2533%HOMO SAPIENS	0.34759608	1.0233148	22	0.9075983	7820
INTRACELLULAR RECEPTOR SIGNALING PATHWAY%GOBP%GO:0030522	0.3155078	1.0824654	97	0.9076308	8675
PROTEIN SUMOYLATION%GOBP%GO:0016925	0.30649012	1.0124004	57	0.9076431	4997
MITOTIC SPINDLE CHECKPOINT%REACTOME%R-HSA-69618.2	0.3172137	1.0904269	107	0.90764797	6033
RELATIONSHIP BETWEEN INFLAMMATION, COX-2 AND EGFR%WIKIPATHWAYS_20210110%WP4483%HOMO SAPIENS	0.39181805	1.0899063	15	0.907649	11091
HDR THROUGH SINGLE STRAND ANNEALING (SSA)%REACTOME DATABASE ID RELEASE 75%5685938	0.3428651	1.0860578	35	0.90767	12221
CARDIAC VENTRICLE MORPHOGENESIS%GOBP%GO:0003208	0.33282652	1.0721712	46	0.90767294	7588
DNA STRAND ELONGATION INVOLVED IN DNA REPLICATION%GOBP%GO:0006271	0.46720293	1.3497107	21	0.9076812	4939
LEWIS BLOOD GROUP BIOSYNTHESIS%REACTOME DATABASE ID RELEASE 75%9037629	0.38965532	1.0932387	16	0.90769607	740
INSULIN PROCESSING%REACTOME%R-HSA-264876.2	0.3636486	1.0906304	27	0.9077015	8778
LEISHMANIA PHAGOCYTOSIS%REACTOME DATABASE ID RELEASE 75%9664417	0.34043595	1.1004914	50	0.9077019	6264
SIGNAL AMPLIFICATION%REACTOME DATABASE ID RELEASE 75%392518	0.33498764	1.0235736	29	0.9077054	9314
MHC CLASS II ANTIGEN PRESENTATION%REACTOME DATABASE ID RELEASE 75%2132295	0.32107377	1.0952154	99	0.9077063	9586
DNA METHYLATION OR DEMETHYLATION%GOBP%GO:0044728	0.33413398	1.0687231	44	0.907708	4173
LIMB MORPHOGENESIS%GOBP%GO:0035108	0.33576804	1.0937544	46	0.9077106	11245
PI3K-AKT SIGNALING PATHWAY%WIKIPATHWAYS_20210110%WP4172%HOMO SAPIENS	0.28450185	1.0211912	231	0.9077126	8404
CELLULAR BIOGENIC AMINE CATABOLIC PROCESS%GOBP%GO:0042402	0.38041642	1.0727575	17	0.9077177	9860
FOCAL ADHESION-PI3K-AKT-MTOR-SIGNALING PATHWAY%WIKIPATHWAYS_20210110%WP3932%HOMO SAPIENS	0.3037304	1.0909357	209	0.9077209	8404
RESPONSE TO ANTINEOPLASTIC AGENT%GOBP%GO:0097327	0.34911624	1.0918648	35	0.9077244	4837
REGULATION OF RELEASE OF SEQUESTERED CALCIUM ION INTO CYTOSOL%GOBP%GO:0051279	0.33524525	1.0816305	46	0.90773785	10960
MICROTUBULE BUNDLE FORMATION%GOBP%GO:0001578	0.3025944	1.0142941	79	0.90774643	8145
GENE AND PROTEIN EXPRESSION BY JAK-STAT SIGNALING AFTER INTERLEUKIN-12 STIMULATION%REACTOME DATABASE ID RELEASE 75%8950505	0.34257254	1.071548	33	0.90775865	6261

POSITIVE REGULATION OF CELL CYCLE G1/S PHASE TRANSITION%GOBP%GO:1902808	0.3515975	1.0931194	35	0.9077605	8479
CD40%IOB%CD40	0.37159875	1.0929115	22	0.90776235	11221
NEGATIVE REGULATION OF PROTEIN SERINE/THREONINE KINASE ACTIVITY%GOBP%GO:0071901	0.2941286	1.0143831	99	0.90776485	7222
POSITIVE REGULATION OF DENDRITE MORPHOGENESIS%GOBP%GO:0050775	0.37840265	1.093534	20	0.90776503	8122
CORTICAL CYTOSKELETON ORGANIZATION%GOBP%GO:0030865	0.41858244	1.3559834	43	0.90776515	11192
LDL CLEARANCE%REACTOME DATABASE ID RELEASE 75%8964038	0.3834981	1.0911313	17	0.90777236	8486
CELL COMMUNICATION INVOLVED IN CARDIAC CONDUCTION%GOBP%GO:0086065	0.33977228	1.0713406	38	0.9077744	7588
NEGATIVE REGULATION OF PROTEIN KINASE ACTIVITY%GOBP%GO:0006469	0.30690658	1.082842	172	0.9078029	7616
MYELOID CELL HOMEOSTASIS%GOBP%GO:0002262	0.32825553	1.0730448	46	0.90780324	3295
INITIATION OF TRANSCRIPTION AND TRANSLATION ELONGATION AT THE HIV-1 LTR%WIKIPATHWAYS_20210110%WP3414%HOMO SAPIENS	0.49870002	1.5061588	27	0.90780705	6669
NETRIN-MEDIATED SIGNALING EVENTS%PATHWAY INTERACTION DATABASE NCI-NATURE CURATED DATA%NETRIN-MEDIATED SIGNALING EVENTS	0.34888098	1.0917451	32	0.90781367	7719
NEGATIVE REGULATION OF MITOTIC CELL CYCLE PHASE TRANSITION%GOBP%GO:1901991	0.30382162	1.0910167	194	0.9078141	6057
CALCIUM ION REGULATED EXOCYTOSIS%GOBP%GO:0017156	0.32795036	1.089965	53	0.90781593	5087
P38 MAPK PATHWAY%PANTHER PATHWAY%P05918	0.32595763	1.0234452	37	0.9078194	3187
CLASS I PI3K SIGNALING EVENTS%PATHWAY INTERACTION DATABASE NCI-NATURE CURATED DATA%CLASS I PI3K SIGNALING EVENTS	0.32501808	1.0214496	37	0.9078294	6350
G ALPHA (Z) SIGNALLING EVENTS%REACTOME DATABASE ID RELEASE 75%418597	0.33210164	1.0729283	45	0.90784174	6440
REGULATION OF ENDOPEPTIDASE ACTIVITY%GOBP%GO:0052548	0.28391662	1.0213447	234	0.9078426	6328
CELLULAR RESPONSE TO TUMOR NECROSIS FACTOR%GOBP%GO:0071356	0.31248757	1.0942395	154	0.90784997	5498
TRNA AMINOACYLATION%REACTOME DATABASE ID RELEASE 75%379724	0.33645397	1.0819031	42	0.9078573	8624
REGULATION OF CENTROSOME DUPLICATION%GOBP%GO:0010824	0.34786758	1.0944418	39	0.90786195	8711
REGULATION OF VIRAL TRANSCRIPTION%GOBP%GO:0046782	0.30839565	1.0212361	60	0.9078725	6975
POSITIVE REGULATION OF FATTY ACID BIOSYNTHETIC PROCESS%GOBP%GO:0045723	0.40264675	1.0943292	15	0.90787864	6153
NRF2-ARE REGULATION%WIKIPATHWAYS_20210110%WP4357%HOMO SAPIENS	0.37776047	1.0938193	21	0.9078882	4523
TISSUE REGENERATION%GOBP%GO:0042246	0.3701258	1.0926629	23	0.9078971	10380
EPITHELIAL CELL MORPHOGENESIS%GOBP%GO:0003382	0.39502347	1.0959077	15	0.907923	729
RESPONSE TO METAL ION%GOBP%GO:0010038	0.3074834	1.0919162	177	0.9079396	4262
TYPE 2 PAPILLARY RENAL CELL CARCINOMA%WIKIPATHWAYS_20210110%WP4241%HOMO SAPIENS	0.36510342	1.0829078	23	0.9079486	1090
REGULATION OF LEUKOCYTE CELL-CELL ADHESION%GOBP%GO:1903037	0.2881392	1.0161543	180	0.90795046	5015
ANTIMICROBIAL HUMORAL IMMUNE RESPONSE MEDIATED BY ANTIMICROBIAL PEPTIDE%GOBP%GO:0061844	0.3591128	1.0932785	28	0.907953	5899
POSITIVE REGULATION OF CELL CYCLE PROCESS%GOBP%GO:0090068	0.29873696	1.0731065	226	0.9079594	6519
DNA-DEPENDENT DNA REPLICATION%GOBP%GO:0006261	0.31102118	1.0830113	120	0.9079753	9018
REGULATION OF CARDIAC MUSCLE CELL DIFFERENTIATION%GOBP%GO:2000725	0.39244935	1.0914918	16	0.907983	9791
ACTIVATION OF PROTEIN KINASE A ACTIVITY%GOBP%GO:0034199	0.39445996	1.091187	16	0.907988	3450
PID_ARF_3PATHWAY%MSIGDB_C2%PID_ARF_3PATHWAY	0.39624605	1.100521	15	0.907998	1935
CELLULAR RESPONSE TO CORTICOSTEROID STIMULUS%GOBP%GO:0071384	0.36604404	1.0146921	17	0.9080106	7957
CELLULAR RESPONSE TO HEAT STRESS%REACTOME%R-HSA-3371556.1	0.32076213	1.0906557	86	0.9080218	5617
FORMATION OF PRIMARY GERM LAYER%GOBP%GO:0001704	0.3308666	1.0945096	59	0.90802634	6802
SIGNAL TRANSDUCTION INVOLVED IN MITOTIC DNA INTEGRITY CHECKPOINT%GOBP%GO:1902403	0.40771866	1.3508416	56	0.9080299	6040
METABOLISM OF VITAMINS AND COFACTORS%REACTOME%R-HSA-196854.6	0.28975463	1.0145863	160	0.9080409	6480
REGULATION OF TISSUE REMODELING%GOBP%GO:0034103	0.31403115	1.0143956	41	0.908041	4523
RESPONSE TO EXTRACELLULAR STIMULUS%GOBP%GO:0009991	0.29754254	1.0734937	233	0.9080472	7178
ENDOCRINE PANCREAS DEVELOPMENT%GOBP%GO:0031018	0.3837229	1.0907519	20	0.9080503	6417
MRNA PROCESSING%WIKIPATHWAYS_20210110%WP411%HOMO SAPIENS	0.3194453	1.0912715	90	0.9080561	4115
REGULATION OF POSITIVE CHEMOTAXIS%GOBP%GO:0050926	0.35856783	1.0144836	18	0.9080745	7352
ANCHORING OF THE BASAL BODY TO THE PLASMA MEMBRANE%REACTOME DATABASE ID RELEASE 75%5620912	0.29649484	1.0215635	96	0.9080899	9746
ESC PLURIPOTENCY PATHWAYS%WIKIPATHWAYS_20210110%WP3931%HOMO SAPIENS	0.31815147	1.0900002	85	0.9081083	9118
ANTIGEN PRESENTATION: FOLDING, ASSEMBLY AND PEPTIDE LOADING OF CLASS I MHC%REACTOME DATABASE ID RELEASE 75%983170	0.36108762	1.0814358	25	0.9081093	8320
STEM CELL DIFFERENTIATION%GOBP%GO:0048863	0.32113484	1.0947918	94	0.9081141	8185
STABILIZATION OF P53%REACTOME DATABASE ID RELEASE 75%69541	0.3270736	1.0853336	55	0.90812963	8770
REGULATION OF STEROID METABOLIC PROCESS%GOBP%GO:0019218	0.30090344	1.0214542	89	0.9081313	6142
TRANSLESION SYNTHESIS BY REV1%REACTOME%R-HSA-110312.1	0.36311746	1.0088019	16	0.908146	11716
VITAMIN D RECEPTOR PATHWAY%WIKIPATHWAYS_20210110%WP2877%HOMO SAPIENS	0.2956592	1.014749	105	0.9081463	7568
INTERLEUKIN-10 SIGNALING%REACTOME%R-HSA-6783783.3	0.34808433	1.0731565	28	0.9081639	3567
REGULATION OF ENDOTHELIAL CELL APOPTOTIC PROCESS%GOBP%GO:2000351	0.3525764	1.0799733	31	0.908167	10960
REGULATION OF RELEASE OF SEQUESTERED CALCIUM ION INTO CYTOSOL BY SARCOPLASMIC RETICULUM%GOBP%GO:0010880	0.3835261	1.0938509	20	0.90817165	10012
GLUCONEOGENESIS%GOBP%GO:0006094	0.43124574	1.3556246	34	0.90818316	8383
PID_TCR_PATHWAY%MSIGDB_C2%PID_TCR_PATHWAY	0.34413162	1.0836592	39	0.9081864	5574
DIFFERENTIATION PATHWAY%WIKIPATHWAYS_20210110%WP2848%HOMO SAPIENS	0.43558085	1.3553088	33	0.90820897	3131
POSITIVE REGULATION OF LYMPHOCYTE MEDIATED IMMUNITY%GOBP%GO:0002708	0.31071702	1.014818	54	0.908225	6569
ENDOTHELIUM DEVELOPMENT%GOBP%GO:0003158	0.32634777	1.1005723	68	0.90822667	8254
LIPOPROTEIN BIOSYNTHETIC PROCESS%GOBP%GO:0042158	0.29703587	1.0088732	79	0.90822977	5699
POLO-LIKE KINASE MEDIATED EVENTS%REACTOME DATABASE ID RELEASE 75%156711	0.393294	1.0915357	16	0.9082303	10143
RAC1/PAK1/P38/MMP2 PATHWAY%WIKIPATHWAYS_20210110%WP3303%HOMO SAPIENS	0.33508694	1.1008943	50	0.90823174	11091
IMMUNOGLOBULIN PRODUCTION%GOBP%GO:0002377	0.4378421	1.3523259	30	0.90823877	8413
NOTCH SIGNALING PATHWAY%PANTHER PATHWAY%P00045	0.35688072	1.0959357	29	0.9082539	11325
EPITHELIAL TO MESENCHYMAL TRANSITION%GOBP%GO:0001837	0.33108988	1.0933089	55	0.9082561	8377
POSITIVE REGULATION OF MITOCHONDRION ORGANIZATION%GOBP%GO:0010822	0.31713897	1.0926795	105	0.90826595	9991
UNSATURATED FATTY ACID METABOLIC PROCESS%GOBP%GO:0033559	0.32385075	1.0945513	74	0.9082689	5585
HEDGEHOG%IOB%HEDGEHOG	0.43978006	1.3472575	28	0.9082789	4911
NCRNAS INVOLVED IN WNT SIGNALING IN HEPATOCELLULAR CARCINOMA%WIKIPATHWAYS_20210110%WP4336%HOMO SAPIENS	0.3280097	1.0948603	67	0.9082808	3237
G PROTEIN SIGNALING PATHWAYS%WIKIPATHWAYS_20210110%WP35%HOMO SAPIENS	0.3275587	1.0809772	61	0.9082902	6094
CELLULAR RESPONSE TO AMINO ACID STARVATION%GOBP%GO:0034198	0.33748358	1.0830343	41	0.90829194	10576
NUCLEAR RECEPTORS META-PATHWAY%WIKIPATHWAYS_20210110%WP2882%HOMO SAPIENS	0.30067214	1.0739286	196	0.9083182	5156
RIBONUCLEOPROTEIN COMPLEX SUBUNIT ORGANIZATION%GOBP%GO:0071826	0.30253544	1.0837197	219	0.9083581	6659
METENCEPHALON DEVELOPMENT%GOBP%GO:0022037	0.32370588	1.0089236	29	0.90836895	1571
BIOCARTA_MET_PATHWAY%MSIGDB_C2%BIOCARTA_MET_PATHWAY	0.34739542	1.0735054	27	0.9083894	4202
TAT-MEDIATED ELONGATION OF THE HIV-1 TRANSCRIPT%REACTOME DATABASE ID RELEASE 75%167246	0.34234458	1.1007503	42	0.90839255	7291
CERAMIDE SIGNALING PATHWAY%PATHWAY INTERACTION DATABASE NCI-NATURE CURATED DATA%CERAMIDE SIGNALING PATHWAY	0.33370528	1.0810536	45	0.908393	4417
INTEGRIN SIGNALING%REACTOME%R-HSA-354192.2	0.46253687	1.352887	20	0.90840334	713
TNFALPHA%NETPATH%TNFALPHA	0.2836391	1.0086225	197	0.90840375	5626
OSSIFICATION%GOBP%GO:0001503	0.31153142	1.0853631	120	0.908411	7433
POSITIVE REGULATION OF SPROUTING ANGIOGENESIS%GOBP%GO:1903672	0.35260886	1.0962001	33	0.9084476	7992
MITOTIC DNA INTEGRITY CHECKPOINT%GOBP%GO:0044774	0.31306413	1.0737916	96	0.9084506	5732
CD209 (DC-SIGN) SIGNALING%REACTOME DATABASE ID RELEASE 75%5621575	0.4783429	1.3540866	18	0.90845305	4366
PROTEIN%REACTOME%R-HSA-446193.1	0.29925537	1.0148414	74	0.90846246	7151
SYNAPTIC VESICLE TRANSPORT%GOBP%GO:0048489	0.32401943	1.0900162	66	0.9084692	2208
NEUROMUSCULAR JUNCTION DEVELOPMENT%GOBP%GO:0007528	0.36375389	1.0946	24	0.9084992	1059
P53-INDEPENDENT DNA DAMAGE RESPONSE%REACTOME%R-HSA-69610.4	0.3323136	1.0799899	50	0.90851444	8770
POSITIVE REGULATION OF TRANSCRIPTION BY RNA POLYMERASE I%GOBP%GO:0045943	0.37138757	1.096282	23	0.9085219	1764
ATM PATHWAY%PATHWAY INTERACTION DATABASE NCI-NATURE CURATED DATA%ATM PATHWAY	0.3458792	1.0739732	34	0.90852416	4837
MAP KINASE ACTIVATION%REACTOME%R-HSA-450294.3	0.32598895	1.0812255	62	0.9085261	10539
BETA	0.33203253	1.0092584	28	0.9085263	1593
SIGNALING BY FGFR%REACTOME%R-HSA-190236.2	0.3331455	1.1009278	68	0.9085341	7470
MELANOMA%WIKIPATHWAYS_20210110%WP4685%HOMO SAPIENS	0.33035073	1.0732594	50	0.90854365	11091
NEGATIVE REGULATION OF PROTEIN UBIQUITINATION%GOBP%GO:0031397	0.32309413	1.0731592	66	0.90854883	9937
DATABASE ID RELEASE 75%5693565	0.3285647	1.0811211	53	0.9085508	6189
G ALPHA (Q) SIGNALLING EVENTS%REACTOME%R-HSA-416476.6	0.30869862	1.0900946	161	0.90857476	7072
INTERLEUKIN RECEPTOR SHC SIGNALING%REACTOME DATABASE ID RELEASE 75%912526	0.3881748	1.0847018	18	0.90858245	4268
NEUROBLAST PROLIFERATION%GOBP%GO:0007405	0.39688474	1.0843935	16	0.9085878	8418
POSITIVE REGULATION OF MUSCLE CELL DIFFERENTIATION%GOBP%GO:0051149	0.33542272	1.0994267	48	0.9086145	6584
HALLMARK_INTERFERON_GAMMA_RESPONSE%MSIGDB_C2%HALLMARK_INTERFERON_GAMMA_RESPONSE	0.30793175	1.0735474	147	0.90861547	8598
NUCLEOSIDE BISPHOSPHATE METABOLIC PROCESS%GOBP%GO:0033865	0.29319292	1.0091338	101	0.9086309	4284
HIV TRANSCRIPTION ELONGATION%REACTOME DATABASE ID RELEASE 75%167169	0.34234458	1.1005803	42	0.9086341	7291
PATTERN RECOGNITION RECEPTOR SIGNALING PATHWAY%GOBP%GO:0002221	0.3153944	1.0806789	100	0.9086374	6996
VIF-MEDIATED DEGRADATION OF APOBEC3G%REACTOME DATABASE ID RELEASE 75%180585	0.3369547	1.0993146	51	0.90864176	8770
LAMELLIPODIUM ORGANIZATION%GOBP%GO:0097581	0.32778707	1.0094122	29	0.90864396	6859
PURINE RIBONUCLEOTIDE BIOSYNTHETIC PROCESS%GOBP%GO:0009152	0.2875538	1.0089316	136	0.90865433	6762
MODULATION BY HOST OF VIRAL TRANSCRIPTION%GOBP%GO:0043921	0.34644926	1.0090287	22	0.9086562	5648
POSITIVE REGULATION OF TRANSPORTER ACTIVITY%GOBP%GO:0032411	0.31880048	1.0959406	80	0.9086673	4165
POSITIVE REGULATION OF TELOMERASE RNA LOCALIZATION TO CAJAL BODY%GOBP%GO:1904874	0.3700357	1.0148696	15	0.90868044	5924
ERYTHROCYTE DIFFERENTIATION%GOBP%GO:0030218	0.34919572	1.09688	37	0.90868366	3153
POSITIVE REGULATION OF CALCIUM-MEDIATED SIGNALING%GOBP%GO:0050850	0.364138	1.0830381	25	0.90868634	10321
NEGATIVE REGULATION OF TRANSPOSITION%GOBP%GO:0010529	0.38581404	1.0999277	19	0.90870255	1148
ONE CARBON METABOLISM%WIKIPATHWAYS_20210110%WP241%HOMO SAPIENS	0.3876668	1.0837371	18	0.90870553	1742
PURINE RIBONUCLEOSIDE TRIPHOSPHATE BIOSYNTHETIC PROCESS%GOBP%GO:0009206	0.3031848	1.0092957	59	0.90871173	7952
HH MUTANTS ABROGATE LIGAND SECRETION%REACTOME DATABASE ID RELEASE 75%5387390	0.30430368	1.0097746	55	0.908724	8770
P53-INDEPENDENT G1 S DNA DAMAGE CHECKPOINT%REACTOME%R-HSA-69613.2	0.3323136	1.0807607	50	0.90872586	8770
TRABECULA MORPHOGENESIS%GOBP%GO:0061383	0.370153	1.0855937	21	0.9087318	8254
APPENDAGE MORPHOGENESIS%GOBP%GO:0035107	0.33576804	1.0963354	46	0.9087335	11245
NEGATIVE REGULATION OF NOTCH SIGNALING PATHWAY%GOBP%GO:0045746	0.3565744	1.0844595	28	0.9087408	9457
OPIOID PROENKEPHALIN PATHWAY%PANTHER PATHWAY%P05915	0.3558487	1.0983385	30	0.9087505	10042
CIRCULATORY SYSTEM PROCESS%GOBP%GO:0003013	0.30685443	1.0960222	208	0.908755	7644
GLYCEROPHOSPHOLIPID BIOSYNTHESIS%REACTOME DATABASE ID RELEASE 75%1483206	0.31078532	1.0853809	114	0.90875673	11335
REGULATION OF INSULIN SECRETION INVOLVED IN CELLULAR RESPONSE TO GLUCOSE STIMULUS%GOBP%GO:0061178	0.45186955	1.3516182	25	0.90875924	7178
MESONEPHRIC TUBULE MORPHOGENESIS%GOBP%GO:0072171	0.3650146	1.079714	22	0.90877575	7674
RESPONSE TO IONIZING RADIATION%GOBP%GO:0010212	0.32051393	1.0845495	88	0.90878373	8413
REGULATION OF AXON EXTENSION INVOLVED IN AXON GUIDANCE%GOBP%GO:0048841	0.3677701	1.1001242	23	0.9087876	821

PREIMPLANTATION EMBRYO%WIKIPATHWAYS_20210110%WP3527%HOMO SAPIENS	0.34046063	1.096423	40	0.90879476	11437
REGULATION OF NUCLEAR BETA CATENIN SIGNALING AND TARGET GENE TRANSCRIPTION%PATHWAY INTERACTION DATABASE NCI-NATURE CURATED DATA%REGULATION OF NUCLEAR BETA CATENIN SIGNALING AND TARGET GENE TRANSCRIPTION	0.3278838	1.0966324	71	0.9088114	3419
CANONICAL WNT SIGNALING PATHWAY%PATHWAY INTERACTION DATABASE NCI-NATURE CURATED DATA%CANONICAL WNT SIGNALING PATHWAY	0.38018715	1.0796052	19	0.90881616	10999
NEUROTRANSMITTER SECRETION%GOBP%GO:0007269	0.3259217	1.0987462	72	0.90882486	5759
CANNABINOID RECEPTOR SIGNALING%WIKIPATHWAYS_20210110%WP3869%HOMO SAPIENS	0.3723303	1.0739979	18	0.9088251	12193
REGULATION OF LEUKOCYTE MIGRATION%GOBP%GO:0002685	0.31545842	1.0986406	132	0.90882695	11269
PID_P38_MKK3_6PATHWAY%MSIGDB_C2%PID_P38_MKK3_6PATHWAY	0.37282124	1.0803177	19	0.9088483	12418
POSITIVE REGULATION OF PROTEIN AUTOPHOSPHORYLATION%GOBP%GO:0031954	0.3376285	1.0100309	23	0.90884864	12905
RIBONUCLEOSIDE BISPHOSPHATE METABOLIC PROCESS%GOBP%GO:0033875	0.29319292	1.015204	101	0.90884966	4284
REGULATION OF CHOLESTEROL METABOLIC PROCESS%GOBP%GO:0090181	0.30872774	1.0094366	51	0.90887654	6142
NEGATIVE REGULATION OF CELL MORPHOGENESIS INVOLVED IN DIFFERENTIATION%GOBP%GO:0010771	0.30364865	1.0083845	64	0.90888363	7546
NEGATIVE REGULATION OF KINASE ACTIVITY%GOBP%GO:0033673	0.30979866	1.0994669	188	0.9088853	7616
RXR AND RAR HETERODIMERIZATION WITH OTHER NUCLEAR RECEPTOR%PATHWAY INTERACTION DATABASE NCI-NATURE CURATED DATA%RXR AND RAR HETERODIMERIZATION WITH OTHER NUCLEAR RECEPTOR	0.36504838	1.0799952	23	0.90890115	6646
LOW-DENSITY LIPOPROTEIN PARTICLE CLEARANCE%GOBP%GO:0034383	0.37666875	1.0854466	21	0.90890193	8541
REGULATION OF TRANSPORTER ACTIVITY%GOBP%GO:0032409	0.281818	1.0152813	218	0.90891236	8038
PROTEIN ACETYLATION%GOBP%GO:0006473	0.31372517	1.0794859	103	0.90891534	9297
BETA3 ADRENERGIC RECEPTOR SIGNALING PATHWAY%PANTHER PATHWAY%P04379	0.37211204	1.0967093	25	0.90892094	10042
NEGATIVE REGULATION OF SECRETION%GOBP%GO:0051048	0.3192973	1.0847187	82	0.9089267	11502
REGULATION OF CALCIUM ION-DEPENDENT EXOCYTOSIS%GOBP%GO:0017158	0.32930568	1.0735668	55	0.9089371	6588
TYPE I INTERFERON SIGNALING PATHWAY%GOBP%GO:0060337	0.31272462	1.0148851	50	0.90893984	6592
REGULATION OF INTRACELLULAR PH%GOBP%GO:0051453	0.32571393	1.0964879	67	0.9089491	8331
VPR-MEDIATED NUCLEAR IMPORT OF PICS%REACTOME DATABASE ID RELEASE 75%180910	0.32603237	1.0156484	31	0.90896505	4994
POSITIVE REGULATION OF TRANSLATION%GOBP%GO:0045727	0.31829548	1.0969161	101	0.9089752	7030
CELLULAR CALCIUM ION HOMEOSTASIS%GOBP%GO:0006874	0.28186134	1.0097904	240	0.9089851	4638
THE CITRIC ACID (TCA) CYCLE AND RESPIRATORY ELECTRON TRANSPORT%REACTOME DATABASE ID RELEASE 75%1428517	0.305352	1.0830666	169	0.9089902	6736
POSITIVE REGULATION OF MUSCLE TISSUE DEVELOPMENT%GOBP%GO:1901863	0.35723093	1.0832709	30	0.9089907	5634
RESPONSE OF MTB TO PHAGOCYTOSIS%REACTOME DATABASE ID RELEASE 75%9637690	0.3426246	1.0094985	22	0.90899104	7219
TRAF6 MEDIATED IRF7 ACTIVATION%REACTOME DATABASE ID RELEASE 75%933541	0.55807	1.5094076	15	0.90899825	4417
NATURAL KILLER CELL ACTIVATION%GOBP%GO:0030101	0.3685777	1.08563	20	0.90900487	9167
RESPONSE TO INTERLEUKIN-6%GOBP%GO:0070741	0.3250493	1.0115112	29	0.9090059	3503
TRANSPORT OF VIRUS%GOBP%GO:0046794	0.33159482	1.0838667	56	0.90902126	8514
GLYCOGEN METABOLIC PROCESS%GOBP%GO:0005977	0.4482965	1.3518853	26	0.9090246	8534
AURORA B SIGNALING%PATHWAY INTERACTION DATABASE NCI-NATURE CURATED DATA%AURORA B SIGNALING	0.32033488	1.0095828	40	0.9090354	11431
NEGATIVE REGULATION OF PROTEIN LOCALIZATION TO PLASMA MEMBRANE%GOBP%GO:1903077	0.34850386	1.01572	21	0.90904516	4209
EGR2 AND SOX10-MEDIATED INITIATION OF SCHWANN CELL MYELINATION%REACTOME%R-HSA-9619665.2	0.36042646	1.0983742	26	0.9090508	11245
SIGNALING	0.35104597	1.0793538	31	0.90905327	10474
EXTRINSIC APOPTOTIC SIGNALING PATHWAY IN ABSENCE OF LIGAND%GOBP%GO:0097192	0.35459203	1.0105314	18	0.90906864	9589
SIGNALING BY EGFR%REACTOME%R-HSA-177929.2	0.3449415	1.0995299	46	0.9090699	8401
SYNTHESIS OF ACTIVE UBIQUITIN: ROLES OF E1 AND E2 ENZYMES%REACTOME DATABASE ID RELEASE 75%8866652	0.35778883	1.0999448	30	0.9090779	6258
REGULATION OF PROTEASOMAL PROTEIN CATABOLIC PROCESS%GOBP%GO:0061136	0.30720383	1.0849911	159	0.90908134	6536
DOPAMINE NEUROTRANSMITTER RELEASE CYCLE%REACTOME DATABASE ID RELEASE 75%212676	0.37608704	1.0987862	22	0.90909153	2851
NEGATIVE REGULATION OF MONONUCLEAR CELL PROLIFERATION%GOBP%GO:0032945	0.3348959	1.0153153	30	0.9091077	5816
VESICLE LOCALIZATION%GOBP%GO:0051648	0.30261883	1.0837389	207	0.9091131	9131
NGF%IOB%NGF	0.3635109	1.0149249	17	0.9091238	4202
ESTABLISHMENT OR MAINTENANCE OF CELL POLARITY%GOBP%GO:0007163	0.30902147	1.0804524	137	0.9091245	8212
HALLMARK_INTERFERON_ALPHA_RESPONSE%MSIGDB_C2%HALLMARK_INTERFERON_ALPHA_RESPONSE	0.3007174	1.0155027	77	0.90912485	8270
RESPONSE TO INTERFERON-GAMMA%GOBP%GO:0034341	0.31560168	1.0847764	103	0.9091285	11324
MAINTENANCE OF PROTEIN LOCATION IN NUCLEUS%GOBP%GO:0051457	0.3795481	1.0997208	20	0.9091334	1929
REGULATION OF RNA EXPORT FROM NUCLEUS%GOBP%GO:0046831	0.36976862	1.010034	15	0.9091457	13491
NEGATIVE REGULATION OF WNT SIGNALING PATHWAY%GOBP%GO:0030178	0.28577223	1.0106006	182	0.90914613	8211
CIRCADIAN CLOCK%REACTOME DATABASE ID RELEASE 75%400253	0.40760833	1.3560886	67	0.90915227	8423
AUTOPHAGY%GOBP%GO:0006914	0.3021337	1.0839317	190	0.9091611	8098
BIOCARTA_PYK2_PATHWAY%MSIGDB_C2%BIOCARTA_PYK2_PATHWAY	0.37130272	1.0988735	23	0.9091695	10579
ERAD PATHWAY%GOBP%GO:0036503	0.29891083	1.0150037	86	0.9091841	7642
RESPONSE TO GAMMA RADIATION%GOBP%GO:0010332	0.35402155	1.0740067	24	0.9091869	8098
SIGNALING PATHWAYS IN GLIOBLASTOMA%WIKIPATHWAYS_20210110%WP2261%HOMO SAPIENS	0.30369508	1.0153854	68	0.90918857	11997
POSITIVE REGULATION OF EPITHELIAL CELL PROLIFERATION%GOBP%GO:0050679	0.29148772	1.0106847	121	0.9091904	10363
SECONDARY PALATE DEVELOPMENT%GOBP%GO:0062009	0.39499253	1.0971786	16	0.9092145	9324
HALLMARK_UV_RESPONSE_DN%MSIGDB_C2%HALLMARK_UV_RESPONSE_DN	0.31478864	1.080123	107	0.90921843	5930
SIGNALING BY ERBB2 IN CANCER%REACTOME DATABASE ID RELEASE 75%1227990	0.37449154	1.0803274	21	0.9092226	11130
NEGATIVE REGULATION OF CELLULAR PROTEIN CATABOLIC PROCESS%GOBP%GO:1903363	0.3237839	1.0833056	71	0.9092398	5005
OLIGODENDROCYTE DIFFERENTIATION%GOBP%GO:0048709	0.32809412	1.0098069	27	0.90924156	9792
NEGATIVE REGULATION OF PURINE NUCLEOTIDE METABOLIC PROCESS%GOBP%GO:1900543	0.35722274	1.0109636	17	0.9092453	6279
OVULATION CYCLE%GOBP%GO:0042698	0.38235676	1.0800046	17	0.90926844	9141
TRNA CHARGING%HUMANCYC%TRNA-CHARGING-PWY	0.33834058	1.075108	38	0.909272	8624
STEROID CATABOLIC PROCESS%GOBP%GO:0006706	0.49114895	1.3563792	16	0.90927446	4179
REGULATION OF FZD BY UBIQUITINATION%REACTOME%R-HSA-4641263.2	0.38040906	1.0984272	19	0.90927464	5993
ARF1 PATHWAY%PATHWAY INTERACTION DATABASE NCI-NATURE CURATED DATA%ARF1 PATHWAY	0.35094926	1.0107573	19	0.9092823	4816
CELL MATURATION%GOBP%GO:0048469	0.33565053	1.0969486	54	0.90928644	4223
CELLULAR GLUCAN METABOLIC PROCESS%GOBP%GO:0006073	0.4482965	1.3473936	26	0.90930414	8534
REGULATION OF UBIQUITIN PROTEIN LIGASE ACTIVITY%GOBP%GO:1904666	0.34515303	1.011134	21	0.9093121	9773
NEGATIVE REGULATION OF PEPTIDE HORMONE SECRETION%GOBP%GO:0090278	0.38785318	1.097047	17	0.90931785	11345
REGULATION OF RESPONSE TO REACTIVE OXYGEN SPECIES%GOBP%GO:1901031	0.36908674	1.0830834	23	0.909333	2283
REGULATION OF PROTEIN KINASE B SIGNALING%GOBP%GO:0051896	0.28461826	1.0080492	161	0.90935475	8404
PROTEIN-CONTAINING COMPLEX DISASSEMBLY%GOBP%GO:0032984	0.2801765	1.0103413	220	0.9093861	6039
REGULATION OF MRNA SPLICING, VIA SPLICEOSOME%GOBP%GO:0048024	0.2934913	1.0112022	97	0.90941036	4224
CELLULAR COMPONENT MAINTENANCE%GOBP%GO:0043954	0.35037777	1.0990304	33	0.90942323	5877
PALLIUM DEVELOPMENT%GOBP%GO:0021543	0.3279983	1.0749696	57	0.90942407	4944
REPRODUCTION%REACTOME DATABASE ID RELEASE 75%1474165	0.30149612	1.0108088	76	0.9094266	5210
SPINDLE ASSEMBLY%GOBP%GO:0051225	0.3215441	1.097961	92	0.90944076	4410
ENDOSOME ORGANIZATION%GOBP%GO:0007032	0.3020057	1.010038	68	0.90944153	8796
REGULATION OF LAMELLIPODIUM ORGANIZATION%GOBP%GO:1902743	0.34424046	1.0995427	42	0.9094557	6307
POSITIVE REGULATION OF PRI-MIRNA TRANSCRIPTION BY RNA POLYMERASE II%GOBP%GO:1902895	0.3285057	1.0081103	28	0.9094631	10933
REGULATION OF NEUROTRANSMITTER RECEPTOR ACTIVITY%GOBP%GO:0099601	0.32343292	1.0740281	61	0.9094995	5740
FORMATION OF HIV-1 ELONGATION COMPLEX CONTAINING HIV-1 TAT%REACTOME%R-HSA-167200.3	0.34234458	1.0847847	42	0.90951514	7291
RESPONSE TO MONOAMINE%GOBP%GO:0071867	0.3432962	1.0109746	21	0.9095217	10576
GANGLIOSIDE METABOLIC PROCESS%GOBP%GO:0001573	0.3693229	1.0839398	22	0.9095462	2356
NEGATIVE REGULATION OF STEROID METABOLIC PROCESS%GOBP%GO:0045939	0.48340598	1.3566447	17	0.9095525	4800
REGULATION OF IMMUNE EFFECTOR PROCESS%GOBP%GO:0002697	0.29999962	1.0751338	225	0.9095679	6569
HALLMARK_GLYCOLYSIS%MSIGDB_C2%HALLMARK_GLYCOLYSIS	0.30981162	1.0971928	142	0.9095925	6065
PHOSPHATIDIC ACID BIOSYNTHETIC PROCESS%GOBP%GO:0006654	0.33549887	1.0741049	40	0.9095936	3929
RESPONSE TO LIGHT STIMULUS%GOBP%GO:0009416	0.3097144	1.0988748	190	0.90960884	4807
RENAL TUBULE MORPHOGENESIS%GOBP%GO:0061333	0.33069834	1.011226	31	0.90964025	8355
REGULATION OF VASCULATURE DEVELOPMENT%GOBP%GO:1901342	0.28227532	1.0100636	242	0.9096629	7591
REGULATION OF LYMPHOCYTE DIFFERENTIATION%GOBP%GO:0045619	0.2959691	1.0073662	88	0.90970474	6935
EXTRINSIC APOPTOTIC SIGNALING PATHWAY VIA DEATH DOMAIN RECEPTORS%GOBP%GO:0008625	0.365163	1.0977899	27	0.9097121	8580
GLYCOSYL COMPOUND METABOLIC PROCESS%GOBP%GO:1901657	0.29430583	1.0075436	92	0.9097633	2643
DNA STRAND ELONGATION%REACTOME DATABASE ID RELEASE 75%69190	0.35441467	1.097978	32	0.9098091	4939
NEGATIVE REGULATION OF NEURON APOPTOTIC PROCESS%GOBP%GO:0043524	0.40377182	1.354182	68	0.9098098	7591
INTERLEUKIN-1 FAMILY SIGNALING%REACTOME%R-HSA-446652.7	0.30908972	1.0742499	123	0.9098203	8841
REGULATION OF STEM CELL POPULATION MAINTENANCE%GOBP%GO:2000036	0.47929385	1.3508635	19	0.9098273	3300
SAPIENS	0.36634687	1.0078073	15	0.9098302	9509
PID_IFNG_PATHWAY%MSIGDB_C2%PID_IFNG_PATHWAY	0.3278429	1.0101054	28	0.90983087	2226
G BETA:GAMMA SIGNALLING THROUGH BTK%REACTOME%R-HSA-8964315.1	0.48145482	1.3479143	16	0.9098334	9314
RHO GTPASE EFFECTORS%REACTOME%R-HSA-195258.4	0.29891208	1.0751477	245	0.9099126	5900
PURINE NUCLEOSIDE DIPHOSPHATE METABOLIC PROCESS%GOBP%GO:0009135	0.34127557	1.0747364	37	0.9099223	4362
MEIOSIS%REACTOME%R-HSA-1500620.2	0.303072	1.0075818	65	0.90993655	12670
KERATAN SULFATE KERATIN METABOLISM%REACTOME%R-HSA-1638074.1	0.35350674	1.0839447	31	0.9099477	1292
HEDGEHOG 'OFF' STATE%REACTOME%R-HSA-5610787.1	0.3144125	1.0741154	95	0.90995336	8770
REGULATION OF CYSTEINE-TYPE ENDOPEPTIDASE ACTIVITY%GOBP%GO:2000116	0.28415674	1.0073833	176	0.90995705	6252
REGULATION OF BINDING%GOBP%GO:0051098	0.3047202	1.0972083	243	0.90996814	7013
TGF-BETA SIGNALING PATHWAY%PANTHER PATHWAY%P00052	0.2936468	1.0076635	86	0.90997106	10539
RAS PATHWAY%PANTHER PATHWAY%P04393	0.3223609	1.0752066	71	0.9100789	11091
POSITIVE REGULATION OF INTERLEUKIN-1 PRODUCTION%GOBP%GO:0032732	0.3471151	1.097264	34	0.9101676	5991
POSITIVE REGULATION OF COLLAGEN METABOLIC PROCESS%GOBP%GO:0010714	0.3624121	1.0071265	15	0.9101704	7542
CELL SURFACE RECEPTOR SIGNALING PATHWAY INVOLVED IN HEART DEVELOPMENT%GOBP%GO:0061311	0.37471604	1.0752828	18	0.91018534	10744
PYRIMIDINE METABOLISM%WIKIPATHWAYS_20210110%WP4022%HOMO SAPIENS	0.32355982	1.0742518	64	0.9102103	5891
BREAST CANCER PATHWAY%WIKIPATHWAYS_20210110%WP4262%HOMO SAPIENS	0.3149115	1.0974504	118	0.91027117	10363
NEGATIVE REGULATION OF INTERLEUKIN-2 PRODUCTION%GOBP%GO:0032703	0.48736075	1.3529052	16	0.91027534	6189
VENTRICULAR CARDIAC MUSCLE TISSUE MORPHOGENESIS%GOBP%GO:0055010	0.3529643	1.0973173	29	0.9103873	7588
REGULATION OF T-HELPER CELL DIFFERENTIATION%GOBP%GO:0045622	0.37710857	1.075528	19	0.91043985	1524
PID_REG_GR_PATHWAY%MSIGDB_C2%PID_REG_GR_PATHWAY	0.33302197	1.0975115	55	0.91044825	4937
AORTA MORPHOGENESIS%GOBP%GO:0035909	0.38079476	1.0789044	19	0.9104614	4346
MUSCLE CONTRACTION%REACTOME%R-HSA-397014.3	0.30435517	1.0753113	165	0.9104746	7858
IMMUNE RESPONSE-ACTIVATING CELL SURFACE RECEPTOR SIGNALING PATHWAY%GOBP%GO:0002429	0.37668896	1.3486749	238	0.9105522	7014

TBC RABGAPS%REACTOME%R-HSA-8854214.1	0.33602715	1.0787779	43	0.9105585	14502
REGULATION OF T CELL DIFFERENTIATION%GOBP%GO:0045580	0.31987035	1.0743637	76	0.9105727	4927
SULFUR AMINO ACID METABOLISM%REACTOME DATABASE ID RELEASE 75%1614635	0.35418797	1.0742534	26	0.9106006	3599
NEGATIVE REGULATION OF OXIDOREDUCTASE ACTIVITY%GOBP%GO:0051354	0.46691033	1.3510454	19	0.91060716	5317
REGULATION OF CELLULAR RESPONSE TO HEAT%GOBP%GO:1900034	0.31514585	1.0755748	79	0.9106484	4994
REGULATION OF TRANSMEMBRANE TRANSPORTER ACTIVITY%GOBP%GO:0022898	0.28324756	1.0065633	206	0.9106878	8038
SIGNAL TRANSDUCTION INVOLVED IN CELL CYCLE CHECKPOINT%GOBP%GO:0072395	0.40913978	1.3543364	72	0.9106946	5612
CROSS-PRESENTATION OF SOLUBLE EXOGENOUS ANTIGENS (ENDOSOMES)%REACTOME DATABASE ID RELEASE 75%1236978	0.31178993	1.0066543	44	0.9106995	8770
PPAR SIGNALING PATHWAY%WIKIPATHWAYS_20210110%WP3942%HOMO SAPIENS	0.33562428	1.0744032	40	0.91081893	9607
HEART MORPHOGENESIS%GOBP%GO:0003007	0.3075362	1.0753206	132	0.91084355	7728
RESPONSE TO CATECHOLAMINE%GOBP%GO:0071869	0.3432962	1.0067014	21	0.91085833	10576
PURINE NUCLEOSIDE BISPHOSPHATE METABOLIC PROCESS%GOBP%GO:0034032	0.29319292	1.0054352	101	0.9109276	4284
PID_MYC_ACTIV_PATHWAY%MSIGDB_C2%PID_MYC_ACTIV_PATHWAY	0.40795192	1.3480383	56	0.91093457	5897
MUSCLE CELL DIFFERENTIATION%GOBP%GO:0042692	0.39059654	1.3531007	113	0.9109423	8428
RENAL SYSTEM DEVELOPMENT%GOBP%GO:0072001	0.28945586	1.0067686	131	0.9109577	7728
REGULATION OF LYASE ACTIVITY%GOBP%GO:0051339	0.510813	1.5440907	27	0.91096866	4171
CALCINEURIN-REGULATED NFAT-DEPENDENT TRANSCRIPTION IN LYMPHOCYTES%PATHWAY INTERACTION DATABASE NCI-NATURE CURATED DATA%CALCINEURIN-REGULATED NFAT-DEPENDENT TRANSCRIPTION IN LYMPHOCYTES	0.44046763	1.3474255	31	0.9110139	9850
NEGATIVE REGULATION OF PEPTIDYL-THREONINE PHOSPHORYLATION%GOBP%GO:0010801	0.5514751	1.5243313	16	0.9110429	1067
PEPTIDYL-PROLINE MODIFICATION%GOBP%GO:0018208	0.34025532	1.0755763	36	0.9110441	2273
AMINO ACID IMPORT ACROSS PLASMA MEMBRANE%GOBP%GO:0089718	0.46493638	1.3483046	20	0.9110648	1776
TIE2-MEDIATED SIGNALING	0.31400236	1.0054803	42	0.9111041	7420
POSITIVE REGULATION OF WOUND HEALING%GOBP%GO:0090303	0.46898258	1.5196078	44	0.9111092	1899
CARDIAC ATRIUM DEVELOPMENT%GOBP%GO:0003230	0.34747347	1.0059432	21	0.9111412	7849
REGULATION OF PROTEIN MODIFICATION BY SMALL PROTEIN CONJUGATION OR REMOVAL%GOBP%GO:1903320	0.28329384	1.0062258	195	0.9111461	10010
SIGNAL TRANSDUCTION IN ABSENCE OF LIGAND%GOBP%GO:0038034	0.35459203	1.0061265	18	0.91115934	9589
CELLULAR RESPONSE TO CARBOHYDRATE STIMULUS%GOBP%GO:0071322	0.3226859	1.0063007	34	0.91121966	11416
SIGNAL TRANSDUCTION THROUGH IL1R%WIKIPATHWAYS_20210110%WP4496%HOMO SAPIENS	0.45360664	1.3569906	24	0.91128683	3663
POSITIVE REGULATION OF RESPONSE TO EXTERNAL STIMULUS%GOBP%GO:0032103	0.28344288	1.0059912	190	0.9112927	10822
REGULATION OF LIPASE ACTIVITY%GOBP%GO:0060191	0.30228478	1.0055095	65	0.9113145	12347
REGULATION OF PROTEIN COMPLEX DISASSEMBLY%GOBP%GO:0043244	0.40062872	1.354545	88	0.91136396	8271
LONG-TERM SYNAPTIC POTENTIATION%GOBP%GO:0060291	0.4645387	1.3566704	22	0.9114049	6482
GLUCOSAMINE-CONTAINING COMPOUND METABOLIC PROCESS%GOBP%GO:1901071	0.372534	1.0755806	20	0.911425	10227
NEGATIVE REGULATION OF PROTEIN SECRETION%GOBP%GO:0050709	0.34145045	1.075645	37	0.911555	10309
SECONDARY ALCOHOL METABOLIC PROCESS%GOBP%GO:1902652	0.29984716	1.0057131	59	0.91156477	6142
BIOCARTA_DEATH_PATHWAY%MSIGDB_C2%BIOCARTA_DEATH_PATHWAY	0.33834141	1.0055208	22	0.9115801	4417
TGF-BETA RECEPTOR SIGNALING IN EMT (EPITHELIAL TO MESENCHYMAL TRANSITION)%REACTOME%R-HSA-2173791.1	0.3638991	1.0056028	16	0.91160965	3446
PROCESS UTILIZING AUTOPHAGIC MECHANISM%GOBP%GO:0061919	0.3021337	1.0780631	190	0.9117195	8098
REGULATION OF PROTEIN LOCALIZATION TO CELL PERIPHERY%GOBP%GO:1904375	0.31139	1.075686	96	0.91179466	8401
GLOMERULUS DEVELOPMENT%GOBP%GO:0032835	0.34524223	1.078352	34	0.9118096	6684
RECEPTOR REGULATORY NETWORK	0.3031165	1.0050083	70	0.9119675	6350
PATHWAY%GOBP%GO:1900740	0.4980224	1.5039861	24	0.9120092	4030
NEGATIVE REGULATION OF G0 TO G1 TRANSITION%GOBP%GO:0070317	0.34255442	1.0781852	37	0.91206574	5189
GLUCAGON SIGNALING IN METABOLIC REGULATION%REACTOME DATABASE ID RELEASE 75%163359	0.34788492	1.0780667	31	0.9121123	6094
PROTEIN ACYLATION%GOBP%GO:0043543	0.30468324	1.0756971	141	0.91215366	9404
CLASS I PEROXISOMAL MEMBRANE PROTEIN IMPORT%REACTOME%R-HSA-9603798.2	0.34481302	1.0044621	19	0.912161	6788
PROSTAGLANDIN SYNTHESIS AND REGULATION%WIKIPATHWAYS_20210110%WP98%HOMO SAPIENS	0.34524962	1.0765123	35	0.9122151	6707
CHORDATE EMBRYONIC DEVELOPMENT%GOBP%GO:0043009	0.28206533	1.004641	162	0.9122239	8692
PRE-NOTCH EXPRESSION AND PROCESSING%REACTOME%R-HSA-1912422.1	0.46188843	1.5405198	68	0.9123264	6040
MUSCLE ORGAN MORPHOGENESIS%GOBP%GO:0048644	0.34079707	1.0767927	40	0.91234636	7588
CELLULAR RESPONSE TO MECHANICAL STIMULUS%GOBP%GO:0071260	0.31569263	1.0045023	44	0.9123481	4470
INTERLEUKIN-12 FAMILY SIGNALING%REACTOME%R-HSA-447115.5	0.3073911	1.0047998	46	0.9123484	4806
MESONEPHRIC EPITHELIUM DEVELOPMENT%GOBP%GO:0072163	0.32112896	1.004701	31	0.9123593	7728
CELL RECRUITMENT (PRO-INFLAMMATORY RESPONSE)%REACTOME DATABASE ID RELEASE 75%9664424	0.37677425	1.0757462	19	0.91237545	6246
ARYL HYDROCARBON RECEPTOR NETPATH%WIKIPATHWAYS_20210110%WP2586%HOMO SAPIENS	0.34529915	1.0772791	34	0.912425	4354
ORGANELLE FISSION%GOBP%GO:0048285	0.2984745	1.0768712	239	0.912427	8599
UBIQUITIN-DEPENDENT DEGRADATION OF CYCLIN D%REACTOME DATABASE ID RELEASE 75%75815	0.3309752	1.0766504	50	0.9124786	8770
REGULATION OF ORNITHINE DECARBOXYLASE (ODC)%REACTOME DATABASE ID RELEASE 75%350562	0.33172756	1.0773678	49	0.91249865	8770
CARGO LOADING INTO VESICLE%GOBP%GO:0035459	0.5229132	1.5072534	19	0.9125132	8126
PURINERGIC SIGNALING IN LEISHMANIASIS INFECTION%REACTOME%R-HSA-9660826.1	0.37677425	1.0765296	19	0.9125439	6246
HALLMARK_MYC_TARGETS_V1%MSIGDB_C2%HALLMARK_MYC_TARGETS_V1	0.30693355	1.0771376	142	0.91257316	8073
HAIR CYCLE PROCESS%GOBP%GO:0022405	0.3695215	1.0042294	15	0.91258794	7011
NEURODEGENERATION WITH BRAIN IRON ACCUMULATION (NBIA) SUBTYPES PATHWAY%WIKIPATHWAYS_20210110%WP4577%HOMO SAPIENS	0.34339225	1.0769278	32	0.9125987	2580
SIGNALING BY PHOSPHORYLATED JUXTAMEMBRANE, EXTRACELLULAR AND KINASE DOMAIN KIT MUTANTS%REACTOME%R-HSA-9670439.1	0.38667253	1.0770212	16	0.9126342	5320
BASE EXCISION REPAIR%WIKIPATHWAYS_20210110%WP4752%HOMO SAPIENS	0.3598671	1.0757568	27	0.91273504	1723
SYNAPTIC VESICLE LOCALIZATION%GOBP%GO:0097479	0.32186756	1.0761704	67	0.9127536	2208
DDX58 IFIH1-MEDIATED INDUCTION OF INTERFERON-ALPHA BETA%REACTOME%R-HSA-168928.7	0.44995403	1.5047461	63	0.91278243	4417
PID_CXCR3_PATHWAY%MSIGDB_C2%PID_CXCR3_PATHWAY	0.35276252	1.0762593	30	0.9128165	11091
POSITIVE REGULATION OF MUSCLE HYPERTROPHY%GOBP%GO:0014742	0.48315138	1.3570732	17	0.91285855	9770
REGULATION OF HEMATOPOIETIC STEM CELL DIFFERENTIATION%GOBP%GO:1902036	0.32182312	1.0773723	68	0.9128838	9483
NEGATIVE REGULATION OF FGFR2 SIGNALING%REACTOME%R-HSA-5654727.1	0.36720088	1.076019	22	0.91294885	8274
MYOFIBRIL ASSEMBLY%GOBP%GO:0030239	0.3426857	1.0757834	36	0.91303045	8428
NOTCH1 INTRACELLULAR DOMAIN REGULATES TRANSCRIPTION%REACTOME%R-HSA-2122947.3	0.46422917	1.509763	45	0.9130619	3016
VESICLE TRANSPORT ALONG ACTIN FILAMENT%GOBP%GO:0030050	0.3935168	1.0758588	15	0.9131422	12795
POSITIVE REGULATION OF T CELL MEDIATED IMMUNITY%GOBP%GO:0002711	0.3470679	1.0773939	31	0.91320413	4417
GENES INVOLVED IN MALE INFERTILITY%WIKIPATHWAYS_20210110%WP4673%HOMO SAPIENS	0.32070574	1.0774422	73	0.9134137	9238
DNA ALKYLATION%GOBP%GO:0006305	0.3613875	1.0774518	29	0.91378134	3947
WATER HOMEOSTASIS%GOBP%GO:0030104	0.42723775	1.3572512	44	0.9138202	3314
EFFECTS OF PIP2 HYDROLYSIS%REACTOME DATABASE ID RELEASE 75%114508	0.32818624	1.0029098	26	0.91399324	4306
INCRETIN SYNTHESIS, SECRETION, AND INACTIVATION%REACTOME DATABASE ID RELEASE 75%400508	0.54254115	1.5113672	16	0.9140758	8162
CELLULAR RESPONSE TO MONOAMINE STIMULUS%GOBP%GO:0071868	0.3432962	1.002971	21	0.91410905	10576
REGULATION OF RECEPTOR BINDING%GOBP%GO:1900120	0.36425546	1.0031838	15	0.91436875	2522
CALCIUM REGULATION IN THE CARDIAC CELL%WIKIPATHWAYS_20210110%WP536%HOMO SAPIENS	0.2921498	1.0029844	96	0.9143731	9484
POSITIVE REGULATION OF LIPID STORAGE%GOBP%GO:0010884	0.3661752	1.0035665	15	0.9144003	13538
EMBRYONIC EPITHELIAL TUBE FORMATION%GOBP%GO:0001838	0.31293026	1.0034359	45	0.9144995	6318
CASPASE CASCADE IN APOPTOSIS%PATHWAY INTERACTION DATABASE NCI-NATURE CURATED DATA%CASPASE CASCADE IN APOPTOSIS	0.31073368	1.0030257	48	0.9145494	4807
REGULATION OF REGULATED SECRETORY PATHWAY%GOBP%GO:1903305	0.2969106	1.0031929	84	0.9146468	5816
D-<I>MYO< I>-INOSITOL-5-PHOSPHATE METABOLISM%HUMANCYC%PWY-6367	0.53757584	1.5459635	19	0.9146908	5543
REGULATION OF SIGNALING BY CBL%REACTOME DATABASE ID RELEASE 75%912631	0.5470171	1.5200673	15	0.91474444	4268
PURINE NUCLEOSIDE TRIPHOSPHATE BIOSYNTHETIC PROCESS%GOBP%GO:0009145	0.3031848	1.0025692	59	0.91475254	7952
HALLMARK_UV_RESPONSE_UP%MSIGDB_C2%HALLMARK_UV_RESPONSE_UP	0.2888504	1.0032434	123	0.9147923	6927
ALZHEIMERS DISEASE%WIKIPATHWAYS_20210110%WP2059%HOMO SAPIENS	0.31195104	1.0023841	44	0.91502166	9509
PID_FAK_PATHWAY%MSIGDB_C2%PID_FAK_PATHWAY	0.31040084	1.0022299	47	0.9151913	5243
CELLULAR RESPONSE TO ALCOHOL%GOBP%GO:0097306	0.42244098	1.3573455	45	0.91530925	2831
RIBOSOMAL SMALL SUBUNIT BIOGENESIS%GOBP%GO:0042274	0.30353785	1.0020218	66	0.91553617	8277
NEGATIVE REGULATION OF EPITHELIAL CELL APOPTOTIC PROCESS%GOBP%GO:1904036	0.32996154	1.0019084	28	0.9155872	9855
GLUCAN CATABOLIC PROCESS%GOBP%GO:0009251	0.49411023	1.3575939	15	0.9156392	8534
UNTITLED%PANTHER PATHWAY%P00019	0.3004487	1.0013254	65	0.9158935	5013
NEGATIVE REGULATION OF ADAPTIVE IMMUNE RESPONSE%GOBP%GO:0002820	0.34628642	1.0013938	19	0.9159942	4996
EGFR TYROSINE KINASE INHIBITOR RESISTANCE%WIKIPATHWAYS_20210110%WP4806%HOMO SAPIENS	0.2976613	1.0014949	72	0.9159964	11091
REGULATION OF PH%GOBP%GO:0006885	0.29885325	1.0016818	73	0.916007	8675
CELLULAR RESPONSE TO CATECHOLAMINE STIMULUS%GOBP%GO:0071870	0.3432962	1.0015467	21	0.91613233	10576
POSITIVE REGULATION OF CYSTEINE-TYPE ENDOPEPTIDASE ACTIVITY INVOLVED IN APOPTOTIC PROCESS%GOBP%GO:0043280	0.2914543	1.0004214	104	0.9162931	6105
POSITIVE REGULATION OF TELOMERE MAINTENANCE VIA TELOMERASE%GOBP%GO:0032212	0.3216482	1.0010688	32	0.91638607	9510
MULTI-MULTICELLULAR ORGANISM PROCESS%GOBP%GO:0044706	0.30639172	1.0004425	55	0.91653013	4179
PROTEIN METHYLATION%REACTOME DATABASE ID RELEASE 75%8876725	0.3601673	1.0002278	17	0.9165819	588
ORC1 REMOVAL FROM CHROMATIN%REACTOME%R-HSA-68949.3	0.2986583	1.0008949	69	0.9166191	8770
CARDIOVASCULAR SYSTEM DEVELOPMENT%GOBP%GO:0072358	0.2757222	1.0004514	242	0.91680515	6859
CEREBELLUM DEVELOPMENT%GOBP%GO:0021549	0.32370588	0.9999265	29	0.9169115	1571
REGULATION OF DENDRITE EXTENSION%GOBP%GO:1903859	0.35700154	0.9987577	18	0.9169509	9432
CELLULAR RESPONSE TO OXYGEN LEVELS%GOBP%GO:0071453	0.28296202	1.0004982	145	0.91695297	7033
POSITIVE REGULATION OF GLYCOPROTEIN METABOLIC PROCESS%GOBP%GO:1903020	0.36308396	0.9980905	16	0.9169627	3550
PRE-MRNA SPLICING%REACTOME DATABASE ID RELEASE 75%72163	0.27960476	0.9981794	180	0.9169791	7153
NEUROTROPHIC FACTOR-MEDIATED TRK RECEPTOR SIGNALING%PATHWAY INTERACTION DATABASE NCI-NATURE CURATED DATA%NEUROTROPHIC FACTOR-MEDIATED TRK RECEPTOR SIGNALING	0.29995844	0.9979819	61	0.9169857	11188
RESPONSE TO GROWTH HORMONE%GOBP%GO:0060416	0.34931147	0.99882376	18	0.9170511	6812
REGULATION OF SPROUTING ANGIOGENESIS%GOBP%GO:1903670	0.3003782	0.99996877	60	0.9170877	6153
PID_RHOA_PATHWAY%MSIGDB_C2%PID_RHOA_PATHWAY	0.32188642	1.0006502	31	0.9170878	3446
MITOCHONDRIAL GENOME MAINTENANCE%GOBP%GO:0000002	0.34299833	0.99716735	20	0.91715306	6022
MOLTING CYCLE PROCESS%GOBP%GO:0022404	0.3695215	1.000531	15	0.91715413	7011
NEGATIVE REGULATION OF MICROTUBULE DEPOLYMERIZATION%GOBP%GO:0007026	0.3392593	0.997728	21	0.91720396	9475
REGULATION OF MICROTUBULE-BASED PROCESS%GOBP%GO:0032886	0.27995142	0.9985819	191	0.9172096	8880
REGULATION OF NUCLEOCYTOPLASMIC TRANSPORT%GOBP%GO:0046822	0.2945159	0.99848473	75	0.9172139	7011
PROTEIN TRANSPORT WITHIN LIPID BILAYER%GOBP%GO:0032594	0.33233234	0.99886614	24	0.9172248	12135
PEROXISOMAL LIPID METABOLISM%REACTOME%R-HSA-390918.5	0.34094337	0.9981799	20	0.91727793	6354
REGULATION OF HEART RATE%GOBP%GO:0002027	0.44893873	1.4944053	69	0.917288	5750
BIOCARTA_TFF_PATHWAY%MSIGDB_C2%BIOCARTA_TFF_PATHWAY	0.35318395	0.99779594	18	0.9172895	10165

RESPONSE TO ACID CHEMICAL%GOBP%GO:0001101	0.28340605	0.9974024	135	0.917293	6970
REGULATION OF PROTEIN BINDING%GOBP%GO:0043393	0.28311163	0.99721235	137	0.9173138	7537
NECTIN ADHESION PATHWAY%PATHWAY INTERACTION DATABASE NCI-NATURE CURATED DATA%NECTIN ADHESION PATHWAY	0.5733353	1.7321358	29	0.9173648	1848
NEGATIVE REGULATION OF LIPID BIOSYNTHETIC PROCESS%GOBP%GO:0051055	0.31092542	0.99728715	39	0.9173672	4837
CELLULAR RESPONSE TO DRUG%GOBP%GO:0035690	0.279881	0.9963045	182	0.9173825	7006
PROTEIN REFOLDING%GOBP%GO:0042026	0.35325193	0.99639684	17	0.9174166	10282
GLUTAMINE FAMILY AMINO ACID METABOLIC PROCESS%GOBP%GO:0009064	0.3097507	0.996591	39	0.91743165	4981
NEGATIVE REGULATION OF CELL MOTILITY%GOBP%GO:2000146	0.27766964	0.995791	208	0.9174343	7616
NON-RECOMBINATIONAL REPAIR%GOBP%GO:0000726	0.29735333	0.9975474	67	0.91744787	6189
NEGATIVE REGULATION OF MITOTIC NUCLEAR DIVISION%GOBP%GO:0045839	0.4315432	1.3579566	35	0.91745716	8983
CDC42 SIGNALING EVENTS%PATHWAY INTERACTION DATABASE NCI-NATURE CURATED DATA%CDC42 SIGNALING EVENTS	0.29611638	0.997444	69	0.91746855	8109
REGULATION OF CELLULAR RESPONSE TO GROWTH FACTOR STIMULUS%GOBP%GO:0090287	0.2777749	0.99567795	204	0.91748375	7329
REGULATION OF MRNA CATABOLIC PROCESS%GOBP%GO:0061013	0.2816092	0.9991639	184	0.9174985	4704
POSITIVE REGULATION OF MUSCLE ORGAN DEVELOPMENT%GOBP%GO:0048636	0.3289038	0.9982963	29	0.9175154	5634
REGULATION OF CYSTEINE-TYPE ENDOPEPTIDASE ACTIVITY INVOLVED IN APOPTOTIC PROCESS%GOBP%GO:0043281	0.28431472	0.99646276	158	0.91751945	6105
POSITIVE REGULATION OF STRIATED MUSCLE TISSUE DEVELOPMENT%GOBP%GO:0045844	0.3289038	0.9988662	29	0.9175268	5634
TNFR1-INDUCED NFKAPPAB SIGNALING PATHWAY%REACTOME%R-HSA-5357956.1	0.3315243	0.9959561	25	0.91753435	11221
FGFR3 SIGNALLING IN CHONDROCYTE PROLIFERATION AND TERMINAL DIFFERENTIATION%WIKIPATHWAYS_20210110%WP4767%HOMO SAPIENS	0.328565	0.99684954	25	0.9175488	5634
PHOSPHOLIPID METABOLISM%REACTOME%R-HSA-1483257.3	0.28273922	0.9981886	189	0.91755146	8404
CELLULAR EXTRAVASATION%GOBP%GO:0045123	0.3274131	0.996925	26	0.91759604	4862
REGULATION OF CELL CYCLE CHECKPOINT%GOBP%GO:1901976	0.32641807	0.9966101	28	0.9176677	11926
PID_NFAT_3PATHWAY%MSIGDB_C2%PID_NFAT_3PATHWAY	0.42811552	1.357605	39	0.9176749	6342
DNA-TEMPLATED TRANSCRIPTION, TERMINATION%GOBP%GO:0006353	0.29674304	0.9958067	70	0.91768473	3876
VASCULATURE DEVELOPMENT%GOBP%GO:0001944	0.2757222	0.99600357	242	0.9176873	6859
REGULATION OF ACTIN NUCLEATION%GOBP%GO:0051125	0.33973742	0.99918896	22	0.91772616	7152
DETECTION OF CHEMICAL STIMULUS INVOLVED IN SENSORY PERCEPTION%GOBP%GO:0050907	0.3685733	0.99668586	15	0.9177331	3103
REGULATION OF HEART GROWTH%GOBP%GO:0060420	0.3293383	0.9995653	28	0.91777027	9435
RESPONSE TO KETONE%GOBP%GO:1901654	0.3025294	0.9988806	59	0.91778827	6179
REGULATION OF FATTY ACID BIOSYNTHETIC PROCESS%GOBP%GO:0042304	0.31873274	0.9953858	33	0.9177899	6153
GOLGI-TO-ER RETROGRADE TRANSPORT%REACTOME%R-HSA-8856688.1	0.28731275	0.9989726	113	0.9178061	3440
REGULATION OF REGULATORY T CELL DIFFERENTIATION%GOBP%GO:0045589	0.5316333	1.5206265	19	0.9178647	1995
NEGATIVE REGULATION OF NUCLEOTIDE METABOLIC PROCESS%GOBP%GO:0045980	0.3527155	0.9960269	19	0.9179175	6279
ESTABLISHMENT OR MAINTENANCE OF BIPOLAR CELL POLARITY%GOBP%GO:0061245	0.3151087	0.9947525	36	0.91794556	11130
PROTEIN DEMETHYLATION%GOBP%GO:0006482	0.32618296	0.99543214	27	0.9179533	11808
DICARBOXYLIC ACID TRANSPORT%GOBP%GO:0006835	0.3034745	0.99465364	51	0.91795725	11287
REGULATION OF GOLGI ORGANIZATION%GOBP%GO:1903358	0.35819227	0.99920815	17	0.9179665	14606
SPHINGOLIPID PATHWAY%WIKIPATHWAYS_20210110%WP1422%HOMO SAPIENS	0.34073523	0.99482644	21	0.91801316	5616
RAC1 SIGNALING PATHWAY%PATHWAY INTERACTION DATABASE NCI-NATURE CURATED DATA%RAC1 SIGNALING PATHWAY	0.3045723	0.9945406	51	0.9180254	10725
NON-INTEGRIN MEMBRANE-ECM INTERACTIONS%REACTOME%R-HSA-3000171.2	0.31372085	0.9948984	40	0.9180927	11800
CIRCADIAN RHYTHM PATHWAY%PATHWAY INTERACTION DATABASE NCI-NATURE CURATED DATA%CIRCADIAN RHYTHM PATHWAY	0.3609044	0.99519044	16	0.918094	7169
DNA DEMETHYLATION%GOBP%GO:0080111	0.36253425	0.99921536	16	0.9182431	8790
NEUROTRANSMITTER METABOLIC PROCESS%GOBP%GO:0042133	0.3005544	0.9949301	56	0.91829765	6420
POSITIVE REGULATION OF SYNAPTIC TRANSMISSION%GOBP%GO:0050806	0.2947135	0.99929285	78	0.9183212	9345
REGULATION OF PROTEIN IMPORT INTO NUCLEUS%GOBP%GO:0042306	0.31215307	0.99501085	34	0.9183508	12680
PROTEIN LOCALIZATION TO CHROMATIN%GOBP%GO:0071168	0.34628695	0.9931856	20	0.9186039	8036
DNA MODIFICATION%GOBP%GO:0006304	0.296974	0.9935733	66	0.9186102	4807
CALMODULIN INDUCED EVENTS%REACTOME%R-HSA-111933.1	0.31452063	0.9895762	34	0.9186521	4909
PID_AR_TF_PATHWAY%MSIGDB_C2%PID_AR_TF_PATHWAY	0.31709844	0.9934563	39	0.91866446	2710
RESPONSE TO CALCIUM ION%GOBP%GO:0051592	0.3969164	1.3580849	94	0.9186699	4187
ESTABLISHMENT OR MAINTENANCE OF APICAL/BASAL CELL POLARITY%GOBP%GO:0035088	0.3151087	0.9894656	36	0.91868913	11130
LXRS REGULATE GENE EXPRESSION LINKED TO CHOLESTEROL TRANSPORT AND EFFLUX%REACTOME DATABASE ID RELEASE 75%9029569	0.31446362	0.99305457	31	0.91869897	8886
RESPONSE TO LIPOPROTEIN PARTICLE%GOBP%GO:0055094	0.34589094	0.9940942	19	0.91879517	10380
ESTABLISHMENT OF MITOCHONDRION LOCALIZATION%GOBP%GO:0051654	0.34321254	0.98962414	20	0.9188048	14557
HEDGEHOG LIGAND BIOGENESIS%REACTOME%R-HSA-5358346.1	0.29390097	0.99360144	61	0.9188222	8770
CONSTITUTIVE SIGNALING BY LIGAND-RESPONSIVE EGFR CANCER VARIANTS%REACTOME%R-HSA-1236382.1	0.35432774	0.993288	17	0.9188802	10363
POSITIVE REGULATION OF PEPTIDE HORMONE SECRETION%GOBP%GO:0090277	0.30910861	0.9931928	46	0.918881	11374
EXTRA-NUCLEAR ESTROGEN SIGNALING%REACTOME%R-HSA-9009391.3	0.29483023	0.99414825	66	0.9189191	9771
NOTCH%IOB%NOTCH	0.30096483	0.99366355	60	0.9189421	7098
ACTION POTENTIAL%GOBP%GO:0001508	0.29302046	0.98964334	72	0.9190418	9435
POSITIVE REGULATION OF OSSIFICATION%GOBP%GO:0045778	0.29936868	0.99283075	60	0.9191283	10779
PDGFR-BETA PATHWAY%WIKIPATHWAYS_20210110%WP3972%HOMO SAPIENS	0.3409459	0.99367416	22	0.9192109	4523
ACTIN FILAMENT ORGANIZATION%GOBP%GO:0007015	0.2770391	0.989649	209	0.9193189	8458
CELLULAR RESPONSE TO EXTERNAL STIMULUS%GOBP%GO:0071496	0.27629408	0.99112236	209	0.9193306	8876
SHC1 EVENTS IN ERBB2 SIGNALING%REACTOME DATABASE ID RELEASE 75%1250196	0.34586588	0.9890561	17	0.919333	13213
ACTIVATION OF ATR IN RESPONSE TO REPLICATION STRESS%REACTOME DATABASE ID RELEASE 75%176187	0.3146198	0.99266005	36	0.9193544	11716
RNA-DEPENDENT DNA BIOSYNTHETIC PROCESS%GOBP%GO:0006278	0.3463777	0.9889515	19	0.91936654	13138
NOTCH2 ACTIVATION AND TRANSMISSION OF SIGNAL TO THE NUCLEUS%REACTOME DATABASE ID RELEASE 75%2979096	0.34293976	0.99148977	22	0.91939324	8689
ESTROGEN SIGNALING PATHWAY%WIKIPATHWAYS_20210110%WP712%HOMO SAPIENS	0.5325832	1.509868	18	0.91939855	10539
NCAM SIGNALING FOR NEURITE OUT-GROWTH%REACTOME DATABASE ID RELEASE 75%375165	0.3054993	0.99370366	48	0.91941935	10879
NEGATIVE REGULATION OF ADAPTIVE IMMUNE RESPONSE BASED ON SOMATIC RECOMBINATION OF IMMUNE RECEPTORS BUILT FROM IMMUNOGLOBULIN SUPERFAMILY DOMAINS%GOBP%GO:0002823	0.35765317	0.99058795	16	0.9194388	4842
REGULATION OF ION TRANSMEMBRANE TRANSPORTER ACTIVITY%GOBP%GO:0032412	0.27714327	0.9911752	200	0.9194597	5753
NEGATIVE REGULATION OF LIPID LOCALIZATION%GOBP%GO:1905953	0.3275009	0.9925245	29	0.9194668	5549
PI3K CASCADE%REACTOME%R-HSA-109704.5	0.32641298	0.98967665	27	0.91952544	5602
SYNAPTIC VESICLE EXOCYTOSIS%GOBP%GO:0016079	0.30949572	0.99182355	39	0.91954666	5759
PROTEIN POLYMERIZATION%GOBP%GO:0051258	0.29268488	0.9890817	80	0.91955143	3370
PROTEIN PALMITOYLATION%GOBP%GO:0018345	0.3234537	0.992401	31	0.9195593	5697
ORGANIC HYDROXY COMPOUND CATABOLIC PROCESS%GOBP%GO:1901616	0.30812457	0.9904461	40	0.91957235	7273
REGULATION OF COLLAGEN BIOSYNTHETIC PROCESS%GOBP%GO:0032965	0.35457027	0.9913289	16	0.9195875	8102
PID_CD8_TCR_PATHWAY%MSIGDB_C2%PID_CD8_TCR_PATHWAY	0.32032076	0.9937326	31	0.9196195	5108
REGULATION OF CELL PROJECTION ASSEMBLY%GOBP%GO:0060491	0.27898133	0.9899345	164	0.91964006	6995
RESPONSE TO GLUCAGON%GOBP%GO:0033762	0.35163867	0.99061733	16	0.91965264	2831
POSITIVE REGULATION OF EXOCYTOSIS%GOBP%GO:0045921	0.29522744	0.9915006	61	0.9196593	12125
NEGATIVE REGULATION OF ENDOPEPTIDASE ACTIVITY%GOBP%GO:0010951	0.29009923	0.9915957	95	0.91966134	4689
COENZYME BIOSYNTHETIC PROCESS%GOBP%GO:0009108	0.2816505	0.9898266	135	0.9196627	5468
PORE COMPLEX ASSEMBLY%GOBP%GO:0046931	0.344029	0.99187636	19	0.9196761	3400
ENDOCHONDRAL OSSIFICATION%WIKIPATHWAYS_20210110%WP474%HOMO SAPIENS	0.3070414	0.9911977	44	0.91968447	12482
NEGATIVE REGULATION OF CELL ADHESION%GOBP%GO:0007162	0.27730703	0.9897203	181	0.9196899	6313
RESPONSE TO TYPE I INTERFERON%GOBP%GO:0034340	0.29650003	0.9916739	55	0.9197289	6592
OUTFLOW TRACT MORPHOGENESIS%GOBP%GO:0003151	0.3088021	0.9907842	48	0.91975343	7728
MRNA SPLICING%REACTOME DATABASE ID RELEASE 75%72172	0.27712393	0.9902835	188	0.919769	7153
RESPONSE TO INTERFERON-ALPHA%GOBP%GO:0035455	0.3532893	0.9906783	17	0.9197782	9634
PREFOLDIN MEDIATED TRANSFER OF SUBSTRATE TO CCT TRIC%REACTOME%R-HSA-389957.2	0.3342722	0.98870546	24	0.91981065	5924
POSITIVE REGULATION OF BMP SIGNALING PATHWAY%GOBP%GO:0030513	0.3370222	0.9900687	22	0.9198229	9324
NEUROTRANSMITTER TRANSPORT%GOBP%GO:0006836	0.28835598	0.99221414	115	0.91983086	4793
FEMALE GAMETE GENERATION%GOBP%GO:0007292	0.30738616	0.9899689	44	0.91983676	9238
INSULIN PATHWAY%PATHWAY INTERACTION DATABASE NCI-NATURE CURATED DATA%INSULIN PATHWAY	0.3069248	0.9919176	45	0.91984046	4465
PROTEIN TARGETING TO LYSOSOME%GOBP%GO:0006622	0.33770448	0.9908338	22	0.9199039	12297
CANONICAL AND NON-CANONICAL NOTCH SIGNALING%WIKIPATHWAYS_20210110%WP3845%HOMO SAPIENS	0.34630156	0.99012464	19	0.91995835	11325
ANGIOGENESIS%GOBP%GO:0001525	0.28023863	0.9919686	156	0.91999185	7491
MITOTIC PROMETAPHASE%REACTOME%R-HSA-68877.5	0.2782813	0.98843503	182	0.92000747	3410
RNA POLYMERASE II TRANSCRIPTION INITIATION AND PROMOTER CLEARANCE%REACTOME%R-HSA-76042.3	0.41652453	1.3581783	44	0.9202028	6737
CALCIUM ION TRANSMEMBRANE IMPORT INTO CYTOSOL%GOBP%GO:0097553	0.31220394	0.9919757	36	0.92026466	3100
NOD1 2 SIGNALING PATHWAY%REACTOME DATABASE ID RELEASE 75%168638	0.31876767	0.988442	31	0.9202811	10153
ASPARTATE FAMILY AMINO ACID BIOSYNTHETIC PROCESS%GOBP%GO:0009067	0.35275477	0.98468405	16	0.92041403	8756
UNTITLED%PANTHER PATHWAY%P05916	0.31745943	0.9845143	30	0.92062956	10042
MANNOSYLATION%GOBP%GO:0097502	0.33593464	0.98469484	23	0.9206775	9692
AMINO ACID TRANSPORT%GOBP%GO:0006865	0.29098925	0.9847745	86	0.9207328	9038
CAM PATHWAY%REACTOME%R-HSA-111997.1	0.31452063	0.9870253	34	0.9207873	4909
PROSTANOID METABOLIC PROCESS%GOBP%GO:0006692	0.43825758	1.3616275	32	0.9207928	5007
LKB1 SIGNALING EVENTS%PATHWAY INTERACTION DATABASE NCI-NATURE CURATED DATA%LKB1 SIGNALING EVENTS	0.30751187	0.9872111	42	0.9208274	9402
ARF6 TRAFFICKING EVENTS%PATHWAY INTERACTION DATABASE NCI-NATURE CURATED DATA%ARF6 TRAFFICKING EVENTS	0.45519093	1.4839126	44	0.9208694	3417
PID_WNT_NONCANONICAL_PATHWAY%MSIGDB_C2%PID_WNT_NONCANONICAL_PATHWAY	0.3310803	0.98729426	23	0.92087257	2429
LEARNING OR MEMORY%GOBP%GO:0007611	0.28618538	0.9848248	104	0.9208795	7644
PROTEIN DEALKYLATION%GOBP%GO:0008214	0.32618296	0.98757714	27	0.9208932	11808
NON-GENOMIC ACTIONS OF 1,25 DIHYDROXYVITAMIN D3%WIKIPATHWAYS_20210110%WP4341%HOMO SAPIENS	0.30233672	0.9858945	51	0.92089665	13027
REGULATION OF EXTRINSIC APOPTOTIC SIGNALING PATHWAY VIA DEATH DOMAIN RECEPTORS%GOBP%GO:1902041	0.30668232	0.9855888	40	0.9209186	5087
NEGATIVE REGULATION OF CELL MIGRATION%GOBP%GO:0030336	0.27689183	0.9862752	199	0.9209362	7616
NEUREXINS AND NEUROLIGINS%REACTOME%R-HSA-6794361.4	0.30027452	0.98676735	56	0.9209674	7681
DISEASES ASSOCIATED WITH O-GLYCOSYLATION OF PROTEINS%REACTOME DATABASE ID RELEASE 75%3906995	0.29447117	0.9868609	61	0.9209825	7593
ARF6 DOWNSTREAM PATHWAY%PATHWAY INTERACTION DATABASE NCI-NATURE CURATED DATA%ARF6 DOWNSTREAM PATHWAY	0.35594225	0.98486334	15	0.9210534	14565
NEGATIVE REGULATION OF CELL-CELL ADHESION%GOBP%GO:0022408	0.28336343	0.9854373	101	0.9210646	6298
ATP-DEPENDENT CHROMATIN REMODELING%GOBP%GO:0043044	0.2909491	0.98702615	71	0.9210777	6033
HISTAMINE H1 RECEPTOR MEDIATED SIGNALING PATHWAY%PANTHER PATHWAY%P04385	0.4284882	1.362235	42	0.9210831	6094
CILIUM ASSEMBLY%REACTOME%R-HSA-5617833.3	0.27651453	0.98632526	178	0.9210839	9080
REGULATION OF PLASMA MEMBRANE BOUNDED CELL PROJECTION ASSEMBLY%GOBP%GO:0120032	0.28069478	0.9873158	163	0.9211107	6995
POSITIVE REGULATION OF INTERFERON-GAMMA PRODUCTION%GOBP%GO:0032729	0.31719592	0.9879846	29	0.9211146	5015

SMOOTH MUSCLE CELL DIFFERENTIATION%GOBP%GO:0051145	0.35558233	0.9856214	16	0.92111766	8254
COPI-MEDIATED ANTEROGRADE TRANSPORT%REACTOME%R-HSA-6807878.1	0.2925668	0.9875866	80	0.92115456	4505
HISTONE DEMETHYLATION%GOBP%GO:0016577	0.3327703	0.9858988	23	0.9211767	11808
CANONICAL NF-KAPPAB PATHWAY%PATHWAY INTERACTION DATABASE NCI-NATURE CURATED DATA%CANONICAL NF-KAPPAB PATHWAY	0.34210834	0.98529947	20	0.92120075	4523
BLOOD VESSEL DEVELOPMENT%GOBP%GO:0001568	0.27368617	0.9848999	231	0.9212293	6137
TRANSCRIPTIONAL ACTIVATION OF MITOCHONDRIAL BIOGENESIS%REACTOME DATABASE ID RELEASE 75%2151201	0.30372003	0.9856729	52	0.92125154	6250
REGULATION OF CARDIAC MUSCLE CONTRACTION BY REGULATION OF THE RELEASE OF SEQUESTERED CALCIUM ION%GOBP%GO:0010881	0.35326052	0.9863625	16	0.9212698	14253
PROTEIN TARGETING TO MEMBRANE%GOBP%GO:0006612	0.27837917	0.9876399	155	0.9212756	5978
PID_NFKAPPAB_CANONICAL_PATHWAY%MSIGDB_C2%PID_NFKAPPAB_CANONICAL_PATHWAY	0.34995478	0.9864497	18	0.92130965	4523
REGENERATION%GOBP%GO:0031099	0.31551778	0.9849687	34	0.92131066	11814
JOUBERT SYNDROME%WIKIPATHWAYS_20210110%WP4656%HOMO SAPIENS	0.3018148	0.98409826	57	0.92131454	3561
PROTEIN LOCALIZATION TO PLASMA MEMBRANE%GOBP%GO:0072659	0.27763206	0.98772496	162	0.92132217	11928
NORC NEGATIVELY REGULATES RRNA EXPRESSION%REACTOME DATABASE ID RELEASE 75%427413	0.2943622	0.9873406	62	0.9213256	6737
NEGATIVE REGULATION OF RAS PROTEIN SIGNAL TRANSDUCTION%GOBP%GO:0046580	0.31950155	0.98594266	34	0.92133236	7222
ACTIN POLYMERIZATION OR DEPOLYMERIZATION%GOBP%GO:0008154	0.30260676	0.98505604	48	0.921335	10894
METABOLISM OF POLYAMINES%REACTOME DATABASE ID RELEASE 75%351202	0.30360168	0.98418057	55	0.9213729	9483
REGULATION OF MICROTUBULE CYTOSKELETON ORGANIZATION%GOBP%GO:0070507	0.28003746	0.9877965	162	0.9213927	8880
REGULATION OF INSULIN SECRETION%GOBP%GO:0050796	0.28760839	0.98512906	104	0.92141587	7365
HDACS DEACETYLATE HISTONES%REACTOME%R-HSA-3214815.3	0.30432796	0.98376465	47	0.92142415	5783
HOMEOSTASIS OF NUMBER OF CELLS%GOBP%GO:0048872	0.29668888	0.985987	65	0.92149484	3521
STEM CELL POPULATION MAINTENANCE%GOBP%GO:0019827	0.29580542	0.9839309	62	0.9215269	5533
PID_PS1_PATHWAY%MSIGDB_C2%PID_PS1_PATHWAY	0.46746176	1.4831005	35	0.92154545	3400
MACROMOLECULE METHYLATION%GOBP%GO:0043414	0.27477574	0.98646504	184	0.92156243	9175
PID_FANCONI_PATHWAY%MSIGDB_C2%PID_FANCONI_PATHWAY	0.31078953	0.98381245	38	0.9215706	12653
MELANOSOME TRANSPORT%GOBP%GO:0032402	0.34690705	0.9835024	18	0.9215963	2114
PDGF SIGNALING PATHWAY%PANTHER PATHWAY%P00047	0.2815445	0.9835752	128	0.9216841	11091
GLYCEROPHOSPHOLIPID CATABOLIC PROCESS%GOBP%GO:0046475	0.47680312	1.358215	19	0.92207646	6378
PID_VEGFR1_PATHWAY%MSIGDB_C2%PID_VEGFR1_PATHWAY	0.3515182	0.9832381	17	0.9220979	12255
NEGATIVE REGULATION OF NEURON DEATH%GOBP%GO:1901215	0.39986736	1.3623933	101	0.92218834	7365
NOTCH-HLH TRANSCRIPTION PATHWAY%REACTOME%R-HSA-350054.3	0.50018156	1.5019357	28	0.92220575	1054
TIGHT JUNCTION INTERACTIONS%REACTOME%R-HSA-420029.1	0.5210359	1.521044	20	0.922213	8039
NOTCH AND WNT SIGNALING	0.46326447	1.5011013	44	0.92222327	3400
PURINE NUCLEOTIDE BIOSYNTHETIC PROCESS%GOBP%GO:0006164	0.2791755	0.9828021	145	0.9222372	8304
DEUBIQUITINATION%REACTOME DATABASE ID RELEASE 75%5688426	0.27578467	0.9826979	244	0.92224723	6538
REGULATION OF ACTOMYOSIN STRUCTURE ORGANIZATION%GOBP%GO:0110020	0.40474522	1.3610798	76	0.92231715	6955
RECEPTOR-TYPE TYROSINE-PROTEIN PHOSPHATASES%REACTOME%R-HSA-388844.2	0.3433716	0.9815949	18	0.922327	12104
RESPONSE TO FLUID SHEAR STRESS%GOBP%GO:0034405	0.3507693	0.9825572	17	0.9223815	7956
METAL ION SLC TRANSPORTERS%REACTOME DATABASE ID RELEASE 75%425410	0.33077827	0.9828278	24	0.922443	5830
NOTCH SIGNALING%WIKIPATHWAYS_20210110%WP268%HOMO SAPIENS	0.476668	1.49453	32	0.92250264	1036
PRESYNAPTIC ENDOCYTOSIS%GOBP%GO:0140238	0.3064111	0.98171085	37	0.9225507	5759
PROTEIN TRANSMEMBRANE IMPORT INTO INTRACELLULAR ORGANELLE%GOBP%GO:0044743	0.30588907	0.9828852	42	0.9225641	11955
TCR SIGNALING%REACTOME%R-HSA-202403.5	0.3926312	1.361689	105	0.92256564	8770
TGF-BETA RECEPTOR SIGNALLING IN SKELETAL DYSPLASIAS%WIKIPATHWAYS_20210110%WP4816%HOMO SAPIENS	0.30151263	0.9816097	48	0.9225719	10558
SMAD PROTEIN SIGNAL TRANSDUCTION%GOBP%GO:0060395	0.3051917	0.9810228	45	0.92259085	9811
CALNEXIN CALRETICULIN CYCLE%REACTOME%R-HSA-901042.2	0.3273254	0.9823778	26	0.9226058	8274
MESONEPHRIC TUBULE DEVELOPMENT%GOBP%GO:0072164	0.32112896	0.9811011	31	0.92262256	7728
GLYCOGEN CATABOLIC PROCESS%GOBP%GO:0005980	0.49411023	1.3584546	15	0.9226617	8534
N-GLYCAN TRIMMING IN THE ER AND CALNEXIN CALRETICULIN CYCLE%REACTOME%R-HSA-532668.2	0.31752452	0.9813826	35	0.92266935	8274
ENDOCHONDRAL OSSIFICATION WITH SKELETAL DYSPLASIAS%WIKIPATHWAYS_20210110%WP4808%HOMO SAPIENS	0.3070414	0.9829099	44	0.9227691	12482
PHOSPHATIDYLINOSITOL METABOLIC PROCESS%GOBP%GO:0046488	0.27985588	0.98173285	148	0.9227742	7282
NEGATIVE REGULATION OF CYSTEINE-TYPE ENDOPEPTIDASE ACTIVITY%GOBP%GO:2000117	0.30028996	0.98111826	60	0.92285377	4689
REGULATION OF RAS BY GAPS%REACTOME DATABASE ID RELEASE 75%5658442	0.29450876	0.9811914	64	0.9229016	8770
SENSORY PERCEPTION OF BITTER TASTE%GOBP%GO:0050913	0.3669295	0.98178697	15	0.92290676	3103
SMOOTH MUSCLE CONTRACTION%GOBP%GO:0006939	0.51386803	1.5219276	24	0.92294675	7006
POSITIVE REGULATION OF PROTEIN COMPLEX DISASSEMBLY%GOBP%GO:0043243	0.4615862	1.3636287	24	0.9229565	2206
5-HYDROXYTRYPTAMINE DEGREDATION%PANTHER PATHWAY%P04372	0.34683987	0.98078597	18	0.9230192	6624
REGULATION OF PATHWAY-RESTRICTED SMAD PROTEIN PHOSPHORYLATION%GOBP%GO:0060393	0.30006203	0.98057467	53	0.92305094	4956
FEMALE PREGNANCY%GOBP%GO:0007565	0.30101463	0.98182285	46	0.9230903	4179
CELLULAR RESPONSE TO MOLECULE OF BACTERIAL ORIGIN%GOBP%GO:0071219	0.2914519	0.9804568	85	0.9231035	5722
CELL PROLIFERATION%GOBP%GO:0008283	0.2748802	0.98034394	207	0.923156	9779
NEGATIVE REGULATION OF ION TRANSPORT%GOBP%GO:0043271	0.2859921	0.9806291	88	0.9231841	6440
LIPID TRANSPORT%GOBP%GO:0006869	0.27864128	0.9820834	162	0.9232101	8750
CELLULAR RESPONSE TO HEXOSE STIMULUS%GOBP%GO:0071331	0.31761432	0.98185766	30	0.92327565	10020
SPHINGOLIPID BIOSYNTHETIC PROCESS%GOBP%GO:0030148	0.39702624	1.3625457	87	0.92338175	5803
ABC TRANSPORTERS IN LIPID HOMEOSTASIS%REACTOME DATABASE ID RELEASE 75%1369062	0.54918075	1.4912138	15	0.9234909	7443
BONE MORPHOGENESIS%GOBP%GO:0060349	0.3098254	0.9818801	34	0.92349875	6684
MITOTIC CYTOKINETIC PROCESS%GOBP%GO:1902410	0.47899273	1.367507	20	0.92350775	3302
STRESS GRANULE ASSEMBLY%GOBP%GO:0034063	0.48317873	1.3589299	18	0.92368484	5717
REGULATION OF ADENYLATE CYCLASE ACTIVITY%GOBP%GO:0045761	0.5383584	1.5460013	18	0.9243908	3176
GLUCOCORTICOID RECEPTOR PATHWAY%WIKIPATHWAYS_20210110%WP2880%HOMO SAPIENS	0.4137252	1.3585173	56	0.9244129	3974
MITOCHONDRIAL TRANSLATION%GOBP%GO:0032543	0.27969894	0.9733377	111	0.9247043	5912
GOLGI ASSOCIATED VESICLE BIOGENESIS%REACTOME%R-HSA-432722.3	0.2998756	0.9794883	54	0.9247246	6732
ENDOCARDIAL CUSHION FORMATION%GOBP%GO:0003272	0.5058126	1.3794926	15	0.9247579	6318
REGULATION OF TUBE SIZE%GOBP%GO:0035150	0.43190396	1.3798144	50	0.92488873	6031
BIOCARTA_NKCELLS_PATHWAY%MSIGDB_C2%BIOCARTA_NKCELLS_PATHWAY	0.35183147	0.9792292	16	0.92489773	11104
HALLMARK_ESTROGEN_RESPONSE_LATE%MSIGDB_C2%HALLMARK_ESTROGEN_RESPONSE_LATE	0.27776814	0.9791206	151	0.92492896	9386
TRANSCRIPTION REGULATION BY BZIP TRANSCRIPTION FACTOR%PANTHER PATHWAY%P00055	0.3043106	0.9795186	48	0.9249369	6737
DISEASES ASSOCIATED WITH GLYCOSYLATION PRECURSOR BIOSYNTHESIS%REACTOME DATABASE ID RELEASE 75%5609975	0.34612066	0.97930783	18	0.9249571	8988
FOLIC ACID METABOLIC PROCESS%GOBP%GO:0046655	0.35149488	0.9733485	17	0.9249598	5968
REGULATION OF CANONICAL WNT SIGNALING PATHWAY%GOBP%GO:0060828	0.27360648	0.979004	237	0.92498106	8799
HALLMARK_INFLAMMATORY_RESPONSE%MSIGDB_C2%HALLMARK_INFLAMMATORY_RESPONSE	0.3898839	1.3590565	124	0.9250185	6810
NEURAL PRECURSOR CELL PROLIFERATION%GOBP%GO:0061351	0.3141691	0.9737242	32	0.92501974	10704
VEGF SIGNALING PATHWAY%PANTHER PATHWAY%P00056	0.41129345	1.3636534	64	0.92502135	4523
PID_ERBB4_PATHWAY%MSIGDB_C2%PID_ERBB4_PATHWAY	0.44322023	1.3676153	32	0.9250616	8059
HEPATITIS B INFECTION%WIKIPATHWAYS_20210110%WP4666%HOMO SAPIENS	0.39216468	1.3626058	108	0.9251637	5843
BIOCARTA_ARENRF2_PATHWAY%MSIGDB_C2%BIOCARTA_ARENRF2_PATHWAY	0.3485872	0.97336483	16	0.92518973	10622
NEGATIVE REGULATION OF SIGNAL TRANSDUCTION BY P53 CLASS MEDIATOR%GOBP%GO:1901797	0.32833844	0.97952855	25	0.9251914	4867
CELLULAR RESPONSE TO ZINC ION%GOBP%GO:0071294	0.35379657	0.9788181	16	0.9252156	2525
TGF-BETA RECEPTOR SIGNALING%WIKIPATHWAYS_20210110%WP560%HOMO SAPIENS	0.30022067	0.9735438	46	0.92524594	10558
FOCAL ADHESION%WIKIPATHWAYS_20210110%WP306%HOMO SAPIENS	0.2797317	0.97374594	141	0.9252465	6309
ENDOPLASMIC RETICULUM MANNOSE TRIMMING%GOBP%GO:1904380	0.35327643	0.97305024	15	0.9252516	11516
APOPTOSIS SIGNALING PATHWAY%PANTHER PATHWAY%P00006	0.28620252	0.9757287	106	0.9253215	11324
KERATAN SULFATE BIOSYNTHESIS%REACTOME DATABASE ID RELEASE 75%2022854	0.32496554	0.97341704	26	0.9253311	1292
TYPE I COLLAGEN SYNTHESIS IN THE CONTEXT OF OSTEOGENESIS IMPERFECTA%WIKIPATHWAYS_20210110%WP4786%HOMO SAPIENS	0.32756847	0.97561455	24	0.925357	4438
SURFACTANT METABOLISM%REACTOME%R-HSA-5683826.3	0.45768818	1.3679018	23	0.9253846	4082
REGULATION OF INTERLEUKIN-8 PRODUCTION%GOBP%GO:0032677	0.2998623	0.9758851	51	0.92543745	7226
PID_S1P_S1P2_PATHWAY%MSIGDB_C2%PID_S1P_S1P2_PATHWAY	0.3428979	0.9757638	17	0.925497	11345
RNA POLYMERASE III CHAIN ELONGATION%REACTOME DATABASE ID RELEASE 75%73780	0.3412874	0.9737557	18	0.9255011	8398
REGULATION OF NUCLEOBASE-CONTAINING COMPOUND TRANSPORT%GOBP%GO:0032239	0.34667006	0.9728618	17	0.92552084	13491
NEGATIVE REGULATION OF AXON GUIDANCE%GOBP%GO:1902668	0.3339085	0.9726571	21	0.9255464	8752
POSITIVE REGULATION OF ACTIN CYTOSKELETON REORGANIZATION%GOBP%GO:2000251	0.35772306	0.97383595	15	0.9255678	8605
KERATAN SULFATE BIOSYNTHETIC PROCESS%GOBP%GO:0018146	0.32496545	0.9783807	26	0.9255943	1292
ANTIMICROBIAL HUMORAL RESPONSE%GOBP%GO:0019730	0.30062446	0.9725252	54	0.9256225	5991
STRIATED MUSCLE CONTRACTION%REACTOME%R-HSA-390522.1	0.32721353	0.9759106	24	0.9256545	7
PIGMENT GRANULE TRANSPORT%GOBP%GO:0051904	0.34690705	0.9781696	18	0.9256566	2114
APOPTOTIC NUCLEAR CHANGES%GOBP%GO:0030262	0.33170083	0.9760074	22	0.9256722	10500
REGULATION OF SYSTEMIC ARTERIAL BLOOD PRESSURE%GOBP%GO:0003073	0.3033094	0.9727099	45	0.92568517	8581
NEGATIVE REGULATION OF AXON EXTENSION INVOLVED IN AXON GUIDANCE%GOBP%GO:0048843	0.34114712	0.9773493	19	0.9257278	8752
RESPONSE TO FIBROBLAST GROWTH FACTOR%GOBP%GO:0071774	0.28202704	0.97228533	83	0.9257301	7470
LATE ENDOSOMAL MICROAUTOPHAGY%REACTOME%R-HSA-9615710.3	0.4495396	1.3807884	31	0.9257346	8531
POSITIVE REGULATION OF NEURON DEATH%GOBP%GO:1901216	0.3056863	0.9753692	40	0.92575717	3713
DEACTIVATION OF THE BETA-CATENIN TRANSACTIVATING COMPLEX%REACTOME DATABASE ID RELEASE 75%3769402	0.42630297	1.3668253	39	0.92577046	4464
RESPONSE TO MECHANICAL STIMULUS%GOBP%GO:0009612	0.28698948	0.9784175	85	0.9257801	9509
BETA-CATENIN DESTRUCTION COMPLEX DISASSEMBLY%GOBP%GO:1904886	0.46810323	1.3602465	19	0.92578137	2966
CELLULAR RESPONSE TO MONOSACCHARIDE STIMULUS%GOBP%GO:0071326	0.31761432	0.97606635	30	0.9257818	10020
GPI ANCHOR METABOLIC PROCESS%GOBP%GO:0006505	0.3115742	0.97751987	32	0.9258036	7559
NEURON CELL-CELL ADHESION%GOBP%GO:0007158	0.35506386	0.97235435	16	0.92580944	5740
INTRINSIC APOPTOTIC SIGNALING PATHWAY IN RESPONSE TO DNA DAMAGE BY P53 CLASS MEDIATOR%GOBP%GO:0042771	0.46734834	1.3628398	21	0.9258348	6040
RIBONUCLEOPROTEIN COMPLEX LOCALIZATION%GOBP%GO:0071166	0.28298846	0.9782062	119	0.9258398	5209
INACTIVATION, RECOVERY AND REGULATION OF THE PHOTOTRANSDUCTION CASCADE%REACTOME%R-HSA-2514859.2	0.47417423	1.3599209	18	0.92584175	4570
HISTONE DEACETYLATION%GOBP%GO:0016575	0.30127236	0.97383624	46	0.92585015	3962
CELLULAR RESPONSE TO NUTRIENT LEVELS%GOBP%GO:0031669	0.2745335	0.9761345	156	0.925869	8145
REGULATION OF CATECHOLAMINE SECRETION%GOBP%GO:0050433	0.31400725	0.97622246	33	0.92590356	9492
COFACTOR CATABOLIC PROCESS%GOBP%GO:0051187	0.30887067	0.9773869	39	0.9259037	6642
NEGATIVE REGULATION OF PEPTIDASE ACTIVITY%GOBP%GO:0010466	0.2861664	0.9746183	103	0.9259138	6945
NEGATIVE REGULATION OF SISTER CHROMATID SEGREGATION%GOBP%GO:0033046	0.44711226	1.3789799	28	0.9259292	8983
PROTEIN K63-LINKED DEUBIQUITINATION%GOBP%GO:0070536	0.32060605	0.97708493	30	0.92593026	10124

CARDIAC CHAMBER MORPHOGENESIS%GOBP%GO:0003206	0.2897439	0.9718984	69	0.9259831	7728
CELLULAR COMPONENT DISASSEMBLY INVOLVED IN EXECUTION PHASE OF APOPTOSIS%GOBP%GO:0006921	0.32739776	0.9703905	24	0.92598695	10500
ESTABLISHMENT OF SPINDLE ORIENTATION%GOBP%GO:0051294	0.32335636	0.9746926	26	0.92600167	9557
DNA DEALKYLATION%GOBP%GO:0035510	0.33173436	0.975183	22	0.9260128	8790
NEGATIVE REGULATION OF RHO PROTEIN SIGNAL TRANSDUCTION%GOBP%GO:0035024	0.34278855	0.9717873	18	0.9260129	5063
REGULATION OF ARP2/3 COMPLEX-MEDIATED ACTIN NUCLEATION%GOBP%GO:0034315	0.3515573	0.977535	16	0.92604357	9740
BRAIN-DERIVED NEUROTROPHIC FACTOR (BDNF) SIGNALING PATHWAY%WIKIPATHWAYS_20210110%WP2380%HOMO SAPIENS	0.281189	0.9784205	124	0.92605877	6616
POSITIVE REGULATION OF STRESS-ACTIVATED PROTEIN KINASE SIGNALING CASCADE%GOBP%GO:0070304	0.2769846	0.9719661	120	0.9260686	5415
PROTEIN K63-LINKED UBIQUITINATION%GOBP%GO:0070534	0.29955563	0.9738454	43	0.9261131	10010
REGULATION OF DOUBLE-STRAND BREAK REPAIR VIA HOMOLOGOUS RECOMBINATION%GOBP%GO:0010569	0.29787454	0.9712486	43	0.92613053	8138
INTRACELLULAR PROTEIN TRANSMEMBRANE TRANSPORT%GOBP%GO:0065002	0.29728276	0.975035	54	0.92614776	11955
DOUBLE-STRAND BREAK REPAIR VIA HOMOLOGOUS RECOMBINATION%GOBP%GO:0000724	0.28446007	0.9739336	90	0.9261478	8243
CELLULAR RESPONSE TO EXTRACELLULAR STIMULUS%GOBP%GO:0031668	0.276342	0.97622895	168	0.92617047	8145
HEPATICOBILIARY SYSTEM DEVELOPMENT%GOBP%GO:0061008	0.31531975	0.9775891	34	0.9261813	8659
POSITIVE REGULATION OF NEURAL PRECURSOR CELL PROLIFERATION%GOBP%GO:2000179	0.33369845	0.97641546	21	0.92619216	9402
NEP NS2 INTERACTS WITH THE CELLULAR EXPORT MACHINERY%REACTOME%R-HSA-168333.3	0.31571335	0.9747226	29	0.92620015	4994
REGULATION OF CYTOKINE PRODUCTION INVOLVED IN INFLAMMATORY RESPONSE%GOBP%GO:1900015	0.32049668	0.9770855	29	0.92621505	12168
PROTEIN TRANSMEMBRANE TRANSPORT%GOBP%GO:0071806	0.29484156	0.97010905	57	0.92621833	11955
NEURAL TUBE DEVELOPMENT%GOBP%GO:0021915	0.2985011	0.97040844	53	0.9262202	13223
VEGFA-VEGFR2 PATHWAY%REACTOME DATABASE ID RELEASE 75%4420097	0.28565946	0.97100836	85	0.92623127	4681
TAT-MEDIATED HIV ELONGATION ARREST AND RECOVERY%REACTOME DATABASE ID RELEASE 75%167243	0.3162346	0.9768853	30	0.9262395	7291
REGULATION OF LIPID LOCALIZATION%GOBP%GO:1905952	0.28435454	0.97629446	99	0.92625046	10145
RUNX2 REGULATES OSTEOBLAST DIFFERENTIATION%REACTOME DATABASE ID RELEASE 75%8940973	0.3362462	0.97408986	20	0.92625904	9509
EICOSANOID METABOLISM VIA CYCLO OXYGENASES (COX)%WIKIPATHWAYS_20210110%WP4719%HOMO SAPIENS	0.33198452	0.9715005	20	0.9262609	7866
NEURON MATURATION%GOBP%GO:0042551	0.4604439	1.3799728	22	0.926261	4223
REGULATION OF ISOTYPE SWITCHING%GOBP%GO:0045191	0.33772922	0.97109115	20	0.9262777	7575
NEUROPEPTIDE SIGNALING PATHWAY%GOBP%GO:0007218	0.30002433	0.9776546	48	0.9262781	6335
REGULATION OF CARBOHYDRATE CATABOLIC PROCESS%GOBP%GO:0043470	0.28688833	0.9713955	72	0.9262788	8098
SIGNALING BY FGFR2 IIIA TM%REACTOME DATABASE ID RELEASE 75%8851708	0.34527382	0.97198147	19	0.9263056	6563
GLUTAMATE SECRETION%GOBP%GO:0014047	0.3320151	0.9701696	24	0.92632556	9035
NUCLEOSIDE PHOSPHATE CATABOLIC PROCESS%GOBP%GO:1901292	0.39765245	1.3647698	90	0.92633116	5021
MITOCHONDRION TRANSPORT ALONG MICROTUBULE%GOBP%GO:0047497	0.34495524	0.97396505	17	0.9263396	14281
NEGATIVE REGULATION OF G2/M TRANSITION OF MITOTIC CELL CYCLE%GOBP%GO:0010972	0.2852292	0.96985465	85	0.92636037	9483
REGULATION OF MEIOTIC CELL CYCLE%GOBP%GO:0051445	0.51066935	1.4839476	19	0.9263909	6864
PHAGOSOME MATURATION%GOBP%GO:0090382	0.30534846	0.97125405	38	0.9263945	8362
TCR%NETPATH%TCR	0.27342787	0.974754	175	0.9264002	5202
DOUBLE-STRAND BREAK REPAIR VIA NONHOMOLOGOUS END JOINING%GOBP%GO:0006303	0.29704633	0.9777057	61	0.92641157	6189
ROOF OF MOUTH DEVELOPMENT%GOBP%GO:0060021	0.3113796	0.9767244	35	0.9264252	9716
MAMMARY GLAND DEVELOPMENT%GOBP%GO:0030879	0.3147693	0.9777912	33	0.92643744	7542
ANTERIOR/POSTERIOR AXIS SPECIFICATION%GOBP%GO:0009948	0.33688757	0.97042346	20	0.9264634	7031
NEGATIVE REGULATION OF STEROID BIOSYNTHETIC PROCESS%GOBP%GO:0010894	0.48340598	1.3591613	17	0.92646587	4800
MITOCHONDRIAL TRANSLATION INITIATION%REACTOME%R-HSA-5368286.1	0.28725362	0.9764173	88	0.92647296	6216
ENDOSOME TO LYSOSOME TRANSPORT%GOBP%GO:0008333	0.3022638	0.97411674	52	0.92647564	12135
PROTEIN FOLDING%GOBP%GO:0006457	0.27384776	0.9748201	181	0.92648864	2491
REGULATION OF MITOCHONDRIAL MEMBRANE PERMEABILITY INVOLVED IN APOPTOTIC PROCESS%GOBP%GO:1902108	0.33959195	0.9715173	19	0.92649424	2049
INTEGRIN-LINKED KINASE SIGNALING%PATHWAY INTERACTION DATABASE NCI-NATURE CURATED DATA%INTEGRIN-LINKED KINASE SIGNALING	0.29912165	0.9698972	45	0.9265256	5558
CELLULAR AMINO ACID CATABOLIC PROCESS%GOBP%GO:0009063	0.282487	0.96968853	93	0.92655	4168
NUCLEAR ENVELOPE ORGANIZATION%GOBP%GO:0006998	0.30215403	0.9695836	45	0.9265976	11648
SIGNAL TRANSDUCTION INVOLVED IN MITOTIC DNA DAMAGE CHECKPOINT%GOBP%GO:1902402	0.40771866	1.3637589	56	0.9265977	6040
POSITIVE REGULATION OF TOLL-LIKE RECEPTOR SIGNALING PATHWAY%GOBP%GO:0034123	0.34079334	0.9741692	19	0.92660785	3692
ORGANIC HYDROXY COMPOUND TRANSPORT%GOBP%GO:0015850	0.28772545	0.9704442	78	0.9266885	8688
REGULATION OF ORGANIC ACID TRANSPORT%GOBP%GO:0032890	0.31489432	0.97653717	31	0.9266906	10649
CYCLIC NUCLEOTIDE BIOSYNTHETIC PROCESS%GOBP%GO:0009190	0.5361138	1.4997647	17	0.92672086	3159
EXOCYTIC PROCESS%GOBP%GO:0140029	0.30595887	0.97641987	44	0.9267499	11596
INTEGRINS IN ANGIOGENESIS%PATHWAY INTERACTION DATABASE NCI-NATURE CURATED DATA%INTEGRINS IN ANGIOGENESIS	0.29635727	0.9741797	59	0.92686576	11800
AUTOPHAGY%WIKIPATHWAYS_20210110%WP4923%HOMO SAPIENS	0.33456886	0.97066194	21	0.9269064	2076
SYNAPSIS%GOBP%GO:0007129	0.3438104	0.97056174	18	0.92691714	1675
EPHRIN RECEPTOR SIGNALING PATHWAY%GOBP%GO:0048013	0.2840297	0.97045004	80	0.9269497	6134
G2 M CHECKPOINTS%REACTOME%R-HSA-69481.3	0.2762755	0.9693438	141	0.9269816	12765
UNFOLDED PROTEIN RESPONSE%WIKIPATHWAYS_20210110%WP4925%HOMO SAPIENS	0.55065876	1.4955871	15	0.9271587	6568
REGULATION OF CARDIOCYTE DIFFERENTIATION%GOBP%GO:1905207	0.45292926	1.3679599	24	0.92730856	9791
RETROGRADE VESICLE-MEDIATED TRANSPORT, GOLGI TO ER%GOBP%GO:0006890	0.40767992	1.3809098	82	0.92744654	1833
REGULATION OF I-KAPPAB KINASE/NF-KAPPAB SIGNALING%GOBP%GO:0043122	0.2720694	0.9684504	201	0.9275073	12327
REGULATION OF PROTEIN INSERTION INTO MITOCHONDRIAL MEMBRANE INVOLVED IN APOPTOTIC SIGNALING PATHWAY%GOBP%GO:1900739	0.4980224	1.4947014	24	0.9275202	4030
PI3K AKT SIGNALING%REACTOME%R-HSA-1257604.9	0.2707291	0.96854347	232	0.92753315	8874
ELASTIC FIBRE FORMATION%REACTOME DATABASE ID RELEASE 75%1566948	0.42947337	1.359334	39	0.9275569	2566
FANCONI ANEMIA PATHWAY%PATHWAY INTERACTION DATABASE NCI-NATURE CURATED DATA%FANCONI ANEMIA PATHWAY	0.29599077	0.96832454	47	0.92759806	12221
REGULATION OF HORMONE METABOLIC PROCESS%GOBP%GO:0032350	0.32313934	0.96860266	25	0.92765677	13836
MITOTIC PROPHASE%REACTOME%R-HSA-68875.4	0.28351614	0.96863204	93	0.927856	8074
GLUCAN METABOLIC PROCESS%GOBP%GO:0044042	0.4482965	1.3655264	26	0.9279229	8534
DNA DAMAGE RESPONSE, DETECTION OF DNA DAMAGE%GOBP%GO:0042769	0.3085359	0.9687791	36	0.9280185	4361
CELLULAR RESPONSE TO BIOTIC STIMULUS%GOBP%GO:0071216	0.2795251	0.96863985	100	0.928114	6158
OLIGODENDROCYTE DEVELOPMENT%GOBP%GO:0014003	0.33844194	0.9687975	19	0.9282513	4773
GLYCERALDEHYDE-3-PHOSPHATE METABOLIC PROCESS%GOBP%GO:0019682	0.48823094	1.3648034	16	0.928371	3548
ENDOPLASMIC RETICULUM TUBULAR NETWORK ORGANIZATION%GOBP%GO:0071786	0.5025466	1.3800232	15	0.928439	2093
BIOCARTA_KERATINOCYTE_PATHWAY%MSIGDB_C2%BIOCARTA_KERATINOCYTE_PATHWAY	0.43163216	1.365774	37	0.92849404	10153
DAG AND IP3 SIGNALING%REACTOME%R-HSA-1489509.2	0.3058865	0.9678064	40	0.9284941	4909
NEGATIVE REGULATION OF BIOMINERAL TISSUE DEVELOPMENT%GOBP%GO:0070168	0.5376055	1.4913886	16	0.92852175	2901
SIGNAL TRANSDUCTION INVOLVED IN DNA DAMAGE CHECKPOINT%GOBP%GO:0072422	0.40739235	1.3641192	69	0.92857534	5612
REGULATION OF TNFR1 SIGNALING%REACTOME%R-HSA-5357905.3	0.30982134	0.9678698	32	0.92859584	11221
CALCIUM-MEDIATED SIGNALING USING INTRACELLULAR CALCIUM SOURCE%GOBP%GO:0035584	0.5275826	1.496235	19	0.92861074	1098
VIRAL BUDDING%GOBP%GO:0046755	0.45692095	1.3637855	26	0.92863786	8531
HALLMARK_HEME_METABOLISM%MSIGDB_C2%HALLMARK_HEME_METABOLISM	0.2757128	0.9674954	128	0.9287906	9558
SULFUR AMINO ACID METABOLIC PROCESS%GOBP%GO:0000096	0.30957305	0.96734846	33	0.92893106	7906
SPINDLE LOCALIZATION%GOBP%GO:0051653	0.3055501	0.9675114	39	0.9290284	9557
INTERLEUKIN-6 FAMILY SIGNALING%REACTOME DATABASE ID RELEASE 75%6783589	0.32625863	0.9671985	22	0.9290661	1600
NEGATIVE REGULATION OF INTRACELLULAR PROTEIN TRANSPORT%GOBP%GO:0090317	0.44040805	1.3680091	29	0.92924494	2662
SYNAPTIC VESICLE ENDOCYTOSIS%GOBP%GO:0048488	0.3064111	0.9666197	37	0.92928725	5759
PYRIDINE-CONTAINING COMPOUND METABOLIC PROCESS%GOBP%GO:0072524	0.42750221	1.4843196	97	0.9294139	5174
OLFACTORY SIGNALING PATHWAY%REACTOME%R-HSA-381753.2	0.47566113	1.3693465	19	0.929445	5264
MICROTUBULE CYTOSKELETON ORGANIZATION INVOLVED IN MITOSIS%GOBP%GO:1902850	0.27958527	0.9666203	105	0.92956513	7013
NEGATIVE REGULATION OF APOPTOTIC SIGNALING PATHWAY%GOBP%GO:2001234	0.273304	0.96602046	153	0.92957294	6057
CELL JUNCTION ORGANIZATION%GOBP%GO:0034330	0.38507026	1.3689582	173	0.92958635	5922
KERATINOCYTE DIFFERENTIATION%GOBP%GO:0030216	0.4123824	1.3809514	63	0.9296869	5532
POSITIVE REGULATION OF PRODUCTION OF MOLECULAR MEDIATOR OF IMMUNE RESPONSE%GOBP%GO:0002702	0.29435587	0.9596325	56	0.9297915	6810
TP53 REGULATES TRANSCRIPTION OF CELL CYCLE GENES%REACTOME DATABASE ID RELEASE 75%6791312	0.4211206	1.365923	49	0.929813	3556
PEPTIDYL-LYSINE DIMETHYLATION%GOBP%GO:0018027	0.3376108	0.9660322	17	0.92982024	6287
GLYCOSAMINOGLYCAN CATABOLIC PROCESS%GOBP%GO:0006027	0.2972942	0.96662444	51	0.9298329	5207
PID_LIS1_PATHWAY%MSIGDB_C2%PID_LIS1_PATHWAY	0.58143616	1.7378722	21	0.92987573	2466
REGULATION OF AXON GUIDANCE%GOBP%GO:1902667	0.31246713	0.9667913	33	0.9299244	6631
CELLULAR RESPONSE TO LIPOPOLYSACCHARIDE%GOBP%GO:0071222	0.28329524	0.96619266	79	0.92992896	5722
COFACTOR BIOSYNTHETIC PROCESS%GOBP%GO:0051188	0.27344266	0.9660826	174	0.92994404	5094
NEGATIVE REGULATION OF ENDOTHELIAL CELL APOPTOTIC PROCESS%GOBP%GO:2000352	0.33583283	0.9666563	20	0.9300218	10960
GOLGI TO PLASMA MEMBRANE TRANSPORT%GOBP%GO:0006893	0.2946988	0.9596361	49	0.9300555	4453
ANTIGEN PROCESSING AND PRESENTATION OF EXOGENOUS PEPTIDE ANTIGEN VIA MHC CLASS I%GOBP%GO:0042590	0.28890634	0.9662312	72	0.9300981	8677
ECM PROTEOGLYCANS%REACTOME DATABASE ID RELEASE 75%3000178	0.29993433	0.95969117	46	0.9301713	6309
NEGATIVE REGULATION OF POTASSIUM ION TRANSMEMBRANE TRANSPORT%GOBP%GO:1901380	0.57026947	1.5465161	15	0.9302307	4230
RIBONUCLEOTIDE BIOSYNTHETIC PROCESS%GOBP%GO:0009260	0.2709604	0.9593703	143	0.9302388	6762
GLUTAMATE NEUROTRANSMITTER RELEASE CYCLE%REACTOME%R-HSA-210500.4	0.3262262	0.9592289	22	0.9303531	9035
NANOPARTICLE-MEDIATED ACTIVATION OF RECEPTOR SIGNALING%WIKIPATHWAYS_20210110%WP2643%HOMO SAPIENS	0.32039747	0.96504104	25	0.9303832	11091
S PHASE%REACTOME DATABASE ID RELEASE 75%69242	0.27313462	0.9596957	158	0.9304322	8898
DNA METHYLATION%REACTOME%R-HSA-5334118.1	0.32190037	0.95767117	23	0.9304763	3166
ACTIN-MYOSIN FILAMENT SLIDING%GOBP%GO:0033275	0.32049832	0.96450484	26	0.93049276	7804
ESTABLISHMENT OF MITOCHONDRION LOCALIZATION, MICROTUBULE-MEDIATED%GOBP%GO:0034643	0.34495524	0.9589656	17	0.9305071	14281
SIGNAL TRANSDUCTION INVOLVED IN DNA INTEGRITY CHECKPOINT%GOBP%GO:0072401	0.40739235	1.364814	69	0.9305478	5612
INFECTION WITH MYCOBACTERIUM TUBERCULOSIS%REACTOME DATABASE ID RELEASE 75%9635486	0.3262679	0.9597502	23	0.93056345	7219
GAP JUNCTION TRAFFICKING%REACTOME DATABASE ID RELEASE 75%190828	0.34432513	0.9651539	15	0.9306064	8486
REGULATION OF MAP KINASE ACTIVITY%GOBP%GO:0043405	0.26858592	0.96505713	240	0.93061084	10710
BIOCARTA_ERK_PATHWAY%MSIGDB_C2%BIOCARTA_ERK_PATHWAY	0.32301626	0.9590208	23	0.9306207	9771
PAUSING AND RECOVERY OF TAT-MEDIATED HIV ELONGATION%REACTOME DATABASE ID RELEASE 75%167238	0.3162346	0.96454567	30	0.93065107	7291
POSITIVE REGULATION OF MYELOID CELL DIFFERENTIATION%GOBP%GO:0045639	0.2922688	0.95770186	56	0.9306571	6799
PEPTIDE HORMONE METABOLISM%REACTOME DATABASE ID RELEASE 75%2980736	0.41612217	1.3818941	66	0.9306704	9013
DIGESTIVE TRACT DEVELOPMENT%GOBP%GO:0048565	0.2983529	0.9648237	48	0.93072194	9324
RESOLUTION OF D-LOOP STRUCTURES THROUGH SYNTHESIS-DEPENDENT STRAND ANNEALING (SDSA)%REACTOME%R-HSA-5693554.1	0.3197164	0.9597892	25	0.93072724	12221
CHOLESTEROL TRANSPORT%GOBP%GO:0030301	0.3039793	0.9655011	42	0.9307479	8637
POSITIVE REGULATION OF REPRODUCTIVE PROCESS%GOBP%GO:2000243	0.4765496	1.4967926	33	0.9307506	6864
DNA IR-DOUBLE STRAND BREAKS (DSBS) AND CELLULAR RESPONSE VIA ATM%WIKIPATHWAYS_20210110%WP3959%HOMO SAPIENS	0.2980282	0.95784736	42	0.93080837	5356

TELOMERE MAINTENANCE VIA TELOMERASE%GOBP%GO:0007004	0.3403641	0.9602651	18	0.93081474	13138
BIOCARTA_VIP_PATHWAY%MSIGDB_C2%BIOCARTA_VIP_PATHWAY	0.35678622	0.9645834	15	0.93082047	4937
TRNA METABOLIC PROCESS%GOBP%GO:0006399	0.27370834	0.9651783	165	0.9308235	8903
SARS CORONAVIRUS AND INNATE IMMUNITY%WIKIPATHWAYS_20210110%WP4912%HOMO SAPIENS	0.3443567	0.9646649	16	0.9308681	8054
BCR%NETPATH%BCR	0.27540055	0.96406305	116	0.93086964	4544
RAB GEFS EXCHANGE GTP FOR GDP ON RABS%REACTOME%R-HSA-8876198.3	0.2798574	0.9577192	87	0.9308823	5996
ACTIN FILAMENT BUNDLE ORGANIZATION%GOBP%GO:0061572	0.2964145	0.9642616	53	0.93088883	8605
REGULATION OF CDC42 ACTIVITY%PATHWAY INTERACTION DATABASE NCI-NATURE CURATED DATA%REGULATION OF CDC42 ACTIVITY	0.44826058	1.376464	30	0.930889	3720
RESOLUTION OF D-LOOP STRUCTURES%REACTOME%R-HSA-5693537.1	0.30848104	0.9600335	32	0.93089014	12221
METHIONINE METABOLIC PROCESS%GOBP%GO:0006555	0.34706858	0.9598114	16	0.9309386	11581
NEGATIVE REGULATION OF VIRAL GENOME REPLICATION%GOBP%GO:0045071	0.29332793	0.96413535	51	0.9309484	4483
ESTABLISHMENT OF PIGMENT GRANULE LOCALIZATION%GOBP%GO:0051905	0.32582673	0.96532696	19	0.93096447	2114
NUCLEOTIDE METABOLISM%REACTOME DATABASE ID RELEASE 75%15869	0.28487998	0.96031284	85	0.93096536	6212
STEM CELL DIVISION%GOBP%GO:0017145	0.33598164	0.95989573	18	0.9309863	7626
G ALPHA (12 13) SIGNALLING EVENTS%REACTOME%R-HSA-416482.5	0.41277787	1.382207	74	0.9309893	6134
TRNA METHYLATION%GOBP%GO:0030488	0.31605777	0.96282613	31	0.9309969	12577
FOXO-MEDIATED TRANSCRIPTION%REACTOME%R-HSA-9614085.2	0.28870744	0.96039736	59	0.93100697	9013
REGULATION OF CYTOKINE PRODUCTION INVOLVED IN IMMUNE RESPONSE%GOBP%GO:0002718	0.2970229	0.96008724	48	0.93101835	6121
REGULATION OF SIGNALING RECEPTOR ACTIVITY%GOBP%GO:0010469	0.3927495	1.3680927	115	0.93102056	6682
REGULATION OF CYTOSOLIC CALCIUM ION CONCENTRATION%GOBP%GO:0051480	0.26884446	0.95846355	167	0.9310554	10467
SIGNALING EVENTS REGULATED BY RET TYROSINE KINASE%PATHWAY INTERACTION DATABASE NCI-NATURE CURATED DATA%SIGNALING EVENTS REGULATED BY RET TYROSINE KINASE	0.30767736	0.9578574	39	0.9310568	4607
PANCREAS DEVELOPMENT%GOBP%GO:0031016	0.31186876	0.9651928	33	0.93106186	7365
PROXIMAL TUBULE TRANSPORT%WIKIPATHWAYS_20210110%WP4917%HOMO SAPIENS	0.3165987	0.9585309	28	0.9311435	10087
REGULATION OF CHOLESTEROL BIOSYNTHETIC PROCESS%GOBP%GO:0045540	0.29899007	0.95862997	41	0.9311468	5114
P75 NTR RECEPTOR-MEDIATED SIGNALLING%REACTOME%R-HSA-193704.1	0.4044286	1.369431	92	0.9311689	6299
REGULATION OF CYTOPLASMIC AND NUCLEAR SMAD2 3 SIGNALING%PATHWAY INTERACTION DATABASE NCI-NATURE CURATED DATA%REGULATION OF CYTOPLASMIC AND NUCLEAR SMAD2 3 SIGNALING	0.33034942	0.9604373	18	0.9311715	10869
BIOCARTA_INTEGRIN_PATHWAY%MSIGDB_C2%BIOCARTA_INTEGRIN_PATHWAY	0.32068658	0.96176046	26	0.93117636	7719
REGULATION OF CARDIAC MUSCLE TISSUE GROWTH%GOBP%GO:0055021	0.32463095	0.96285003	24	0.93120676	9435
CYTOPLASMIC SEQUESTERING OF PROTEIN%GOBP%GO:0051220	0.33661804	0.96294826	19	0.9312193	4801
NUCLEOTIDE BIOSYNTHETIC PROCESS%GOBP%GO:0009165	0.27029034	0.9578952	190	0.9312259	4635
SISTER CHROMATID COHESION%GOBP%GO:0007062	0.3044324	0.95819175	37	0.931234	12742
REGULATION OF SPHINGOLIPID BIOSYNTHETIC PROCESS%GOBP%GO:0090153	0.3478508	0.956172	15	0.93124425	11855
RESPONSE TO CAMP%GOBP%GO:0051591	0.31619513	0.96183014	31	0.9312547	2371
MONOVALENT INORGANIC CATION HOMEOSTASIS%GOBP%GO:0055067	0.27833882	0.9559659	98	0.9312668	10325
REGULATION OF SMOOTH MUSCLE CELL MIGRATION%GOBP%GO:0014910	0.3125822	0.95624727	32	0.93131	11369
TRANSPORT OF INORGANIC CATIONS ANIONS AND AMINO ACIDS OLIGOPEPTIDES%REACTOME DATABASE ID RELEASE 75%425393	0.28847125	0.96300244	81	0.93134797	8309
EMBRYO DEVELOPMENT ENDING IN BIRTH OR EGG HATCHING%GOBP%GO:0009792	0.27137965	0.95603466	172	0.93134826	7143
DEFENSE RESPONSE TO VIRUS%GOBP%GO:0051607	0.27868202	0.9618849	99	0.9313808	5991
INTERLEUKIN-7 SIGNALING%REACTOME%R-HSA-1266695.8	0.33378807	0.95631677	19	0.9313817	5320
AMYLOID FIBER FORMATION%REACTOME%R-HSA-977225.6	0.28529632	0.96045935	61	0.93138933	3400
POSITIVE REGULATION OF MRNA PROCESSING%GOBP%GO:0050685	0.32002234	0.9621801	26	0.9314037	12883
MACROAUTOPHAGY%REACTOME%R-HSA-1632852.8	0.27594376	0.9582288	111	0.9314047	8531
VEGFR2 MEDIATED VASCULAR PERMEABILITY%REACTOME DATABASE ID RELEASE 75%5218920	0.32165074	0.9605559	25	0.9314058	2648
REGULATION OF MACROPHAGE CHEMOTAXIS%GOBP%GO:0010758	0.33380097	0.9612647	19	0.93143445	11417
LAMININ INTERACTIONS%REACTOME DATABASE ID RELEASE 75%3000157	0.3281229	0.96146774	22	0.9314357	11800
RIBOSE PHOSPHATE BIOSYNTHETIC PROCESS%GOBP%GO:0046390	0.27205318	0.9579056	149	0.9314738	4635
ALPHA6BETA4INTEGRIN%IOB%ALPHA6BETA4INTEGRIN	0.294509	0.96224463	50	0.93150073	11345
CYCLIN A:CDK2-ASSOCIATED EVENTS AT S PHASE ENTRY%REACTOME DATABASE ID RELEASE 75%69656	0.28287712	0.9606162	84	0.9315173	9736
APOPTOTIC SIGNALING PATHWAY%GOBP%GO:0097190	0.26630348	0.9550688	211	0.9315215	8864
MULTIVESICULAR BODY ASSEMBLY%GOBP%GO:0036258	0.4381139	1.3683307	30	0.931528	7828
REGULATION OF PROTEIN TARGETING%GOBP%GO:1903533	0.28939676	0.9615252	64	0.9315462	10382
REGULATION OF CELLULAR SENESCENCE%GOBP%GO:2000772	0.3153741	0.9611206	27	0.9315487	4842
MUSCLE FILAMENT SLIDING%GOBP%GO:0030049	0.32049832	0.9607085	26	0.931552	7804
INTERACTIONS OF REV WITH HOST CELLULAR PROTEINS%REACTOME DATABASE ID RELEASE 75%177243	0.30348164	0.9623253	34	0.9315592	4994
CENTRAL NERVOUS SYSTEM NEURON DEVELOPMENT%GOBP%GO:0021954	0.3035774	0.9637232	41	0.93157107	6350
PID_WNT_SIGNALING_PATHWAY%MSIGDB_C2%PID_WNT_SIGNALING_PATHWAY	0.33020872	0.95644116	22	0.93157893	5993
POSITIVE REGULATION OF PROTEIN MODIFICATION BY SMALL PROTEIN CONJUGATION OR REMOVAL%GOBP%GO:1903322	0.27517867	0.9565364	113	0.9316071	9584
SIGNAL RELEASE%GOBP%GO:0023061	0.28155062	0.963007	106	0.93161166	6752
NEURAL RETINA DEVELOPMENT%GOBP%GO:0003407	0.30748785	0.96310204	31	0.9316234	7094
NUCLEOTIDE TRANSPORT%GOBP%GO:0006862	0.33781093	0.9571373	19	0.9316262	2515
DENDRITE MORPHOGENESIS%GOBP%GO:0048813	0.30392784	0.9549375	39	0.9316306	9141
CYCLIN E ASSOCIATED EVENTS DURING G1 S TRANSITION%REACTOME%R-HSA-69202.3	0.28230688	0.95512635	82	0.9316358	9736
ENDOSOMAL TRANSPORT%GOBP%GO:0016197	0.26689827	0.9563226	216	0.9316366	10115
LONG-CHAIN FATTY ACID BIOSYNTHETIC PROCESS%GOBP%GO:0042759	0.32654902	0.9635987	23	0.93164	5007
INHIBITION OF DNA RECOMBINATION AT TELOMERE%REACTOME%R-HSA-9670095.2	0.29916403	0.96249354	39	0.93165034	5533
CELLULAR PROTEIN COMPLEX DISASSEMBLY%GOBP%GO:0043624	0.27433172	0.95702994	131	0.93165094	5912
CALCIUM ION TRANSPORT%GOBP%GO:0006816	0.27381492	0.96198267	137	0.9316547	3251
POSITIVE REGULATION OF LIPID LOCALIZATION%GOBP%GO:1905954	0.28913078	0.96188545	59	0.9316553	11335
CELLULAR RESPONSE TO TOPOLOGICALLY INCORRECT PROTEIN%GOBP%GO:0035967	0.27307495	0.9579375	136	0.93166107	5400
REGULATION OF SEQUESTERING OF CALCIUM ION%GOBP%GO:0051282	0.2847117	0.9612714	65	0.9316938	10635
REGULATION OF POSTSYNAPSE ORGANIZATION%GOBP%GO:0099175	0.29328135	0.9623756	51	0.9316981	11421
PATHWAY%GOBP%GO:0051482	0.3435373	0.95660114	16	0.93170834	3051
REGULATION OF FIBROBLAST PROLIFERATION%GOBP%GO:0048145	0.29626432	0.9556828	44	0.9317558	10831
POSITIVE REGULATION OF MUSCLE CONTRACTION%GOBP%GO:0045933	0.6029045	1.6838232	17	0.9317569	5224
SIGNALING BY FGFR1%REACTOME DATABASE ID RELEASE 75%5654736	0.30164072	0.9555754	36	0.93176645	7222
ORGANELLE LOCALIZATION BY MEMBRANE TETHERING%GOBP%GO:0140056	0.27377015	0.96315	152	0.93176985	11660
REGULATION OF TELOMERASE RNA LOCALIZATION TO CAJAL BODY%GOBP%GO:1904872	0.3413176	0.9607112	18	0.93182296	7827
COLLAGEN DEGRADATION%REACTOME DATABASE ID RELEASE 75%1442490	0.30609483	0.9566593	33	0.9318324	11446
POSITIVE REGULATION OF ENDOCYTOSIS%GOBP%GO:0045807	0.2777853	0.95684683	98	0.9318693	7267
MICRORNAS IN CARDIOMYOCYTE HYPERTROPHY%WIKIPATHWAYS_20210110%WP1544%HOMO SAPIENS	0.41293523	1.3810023	69	0.9318952	5149
RESOLUTION OF SISTER CHROMATID COHESION%REACTOME%R-HSA-2500257.1	0.27830535	0.9551272	102	0.931906	3283
DEGRADATION OF THE EXTRACELLULAR MATRIX%REACTOME%R-HSA-1474228.4	0.28538874	0.96338856	86	0.9319421	11733
NEGATIVE REGULATION OF INTRINSIC APOPTOTIC SIGNALING PATHWAY%GOBP%GO:2001243	0.28912687	0.96317905	72	0.93197334	6322
RNA EXPORT FROM NUCLEUS%GOBP%GO:0006405	0.27216616	0.9551963	123	0.9319776	5209
REGULATION OF TRANS-SYNAPTIC SIGNALING%GOBP%GO:0099177	0.26627392	0.9566964	234	0.93200153	5740
DISORDERS OF TRANSMEMBRANE TRANSPORTERS%REACTOME DATABASE ID RELEASE 75%5619115	0.27084184	0.95440435	143	0.93200195	8770
SIGNALING EVENTS MEDIATED BY PRL%PATHWAY INTERACTION DATABASE NCI-NATURE CURATED DATA%SIGNALING EVENTS MEDIATED BY PRL	0.32654458	0.9552872	23	0.93200403	13833
SCF(SKP2)-MEDIATED DEGRADATION OF P27 P21%REACTOME DATABASE ID RELEASE 75%187577	0.28848872	0.9608355	59	0.93202245	8770
MEMBRANE DOCKING%GOBP%GO:0022406	0.26957044	0.9542811	161	0.9320582	11660
FATTY ACID CATABOLIC PROCESS%GOBP%GO:0009062	0.28351218	0.95447844	78	0.9320798	9267
MODULATION OF CHEMICAL SYNAPTIC TRANSMISSION%GOBP%GO:0050804	0.26836514	0.96071774	233	0.9320837	5740
LEARNING%GOBP%GO:0007612	0.29659578	0.96323663	51	0.932101	7537
SIGNALING EVENTS	0.31176662	0.9553385	29	0.9321371	4995
REGULATION OF PTEN STABILITY AND ACTIVITY%REACTOME%R-HSA-8948751.1	0.2840599	0.95453215	66	0.9322126	8770
REGULATION OF MEMBRANE LIPID METABOLIC PROCESS%GOBP%GO:1905038	0.3478508	0.9546176	15	0.9322569	11855
COPI-DEPENDENT GOLGI-TO-ER RETROGRADE TRAFFIC%REACTOME%R-HSA-6811434.1	0.2779347	0.95315206	80	0.9323131	6613
MRNA EXPORT FROM NUCLEUS%GOBP%GO:0006406	0.27578908	0.95298827	102	0.9324697	5209
CHOLESTEROL BIOSYNTHETIC PROCESS%GOBP%GO:0006695	0.30823714	0.95329267	30	0.93247545	3693
MITOTIC RECOMBINATION%GOBP%GO:0006312	0.3423934	0.9528621	16	0.93253386	12221
FAS SIGNALING PATHWAY%PANTHER PATHWAY%P00020	0.30725724	0.9531653	32	0.93254733	6464
TNFS BIND THEIR PHYSIOLOGICAL RECEPTORS%REACTOME%R-HSA-5669034.2	0.33675224	0.95378476	18	0.9325555	8580
PID_A6B1_A6B4_INTEGRIN_PATHWAY%MSIGDB_C2%PID_A6B1_A6B4_INTEGRIN_PATHWAY	0.3068094	0.9539876	34	0.93257344	10165
POSITIVE REGULATION OF I-KAPPAB KINASE/NF-KAPPAB SIGNALING%GOBP%GO:0043123	0.26797965	0.95388687	155	0.93257576	5813
POSITIVE REGULATION OF CALCIUM ION TRANSPORT%GOBP%GO:0051928	0.2874187	0.9535515	64	0.9326233	10960
ENDOSOMAL SORTING COMPLEX REQUIRED FOR TRANSPORT (ESCRT)%REACTOME DATABASE ID RELEASE 75%917729	0.44969285	1.3823333	31	0.9326388	8531
HALLMARK_E2F_TARGETS%MSIGDB_C2%HALLMARK_E2F_TARGETS	0.2689863	0.95331943	152	0.9326822	8735
NEGATIVE REGULATION OF ALPHA-BETA T CELL ACTIVATION%GOBP%GO:0046636	0.3201898	0.94871294	23	0.93271244	1995
NEGATIVE REGULATION OF PROTEIN LOCALIZATION TO CELL PERIPHERY%GOBP%GO:1904376	0.31801006	0.9465553	23	0.93272644	4209
NUCLEOBASE METABOLIC PROCESS%GOBP%GO:0009112	0.30887857	0.9453339	27	0.93272716	9915
NETRIN-1 SIGNALING%REACTOME%R-HSA-373752.2	0.29188344	0.94461465	47	0.9327349	6083
SIGNALING BY NOTCH1 HD DOMAIN MUTANTS IN CANCER%REACTOME DATABASE ID RELEASE 75%2691230	0.34416103	0.9455329	15	0.93274945	8274
SUMOYLATION OF TRANSCRIPTION FACTORS%REACTOME DATABASE ID RELEASE 75%3232118	0.3365529	0.94806874	18	0.93277365	10466
PROTEIN DEPHOSPHORYLATION%GOBP%GO:0006470	0.2697471	0.9456145	132	0.9327967	5981
3-PHOSPHOINOSITIDE DEGRADATION%HUMANCYC%PWY-6368	0.32894415	0.9454038	19	0.9328251	3757
EMBRYONIC ORGAN MORPHOGENESIS%GOBP%GO:0048562	0.27635354	0.9535764	119	0.93283015	7321
CONSTITUTIVE SIGNALING BY NOTCH1 HD DOMAIN MUTANTS%REACTOME%R-HSA-2691232.1	0.34416103	0.94823474	15	0.93287593	8274
PROTEIN O-LINKED GLYCOSYLATION%GOBP%GO:0006493	0.2774235	0.9456756	89	0.9328994	5659
NERVE DEVELOPMENT%GOBP%GO:0021675	0.308818	0.9465848	32	0.93291104	7365
HALLMARK_MTORC1_SIGNALING%MSIGDB_C2%HALLMARK_MTORC1_SIGNALING	0.27456373	0.95333153	138	0.93292063	5812
POSITIVE REGULATION OF MAP KINASE ACTIVITY%GOBP%GO:0043406	0.2639701	0.9401201	178	0.9329297	10710
INTERACTIONS	0.43276983	1.3765271	38	0.93295527	4796
REGULATION OF IMMUNOGLOBULIN MEDIATED IMMUNE RESPONSE%GOBP%GO:0002889	0.31811056	0.94871914	24	0.93296385	7575
AXIS SPECIFICATION%GOBP%GO:0009798	0.30152875	0.94809186	38	0.9329775	11530
ENDODERM DEVELOPMENT%GOBP%GO:0007492	0.29595783	0.94472224	42	0.9329787	6802
CELLULAR RESPONSE TO LIPID%GOBP%GO:0071396	0.2639647	0.9446202	238	0.93298674	8165

SPECIFICATION OF SYMMETRY%GOBP%GO:0009799	0.2819654	0.9487869	67	0.933057	7416
NUCLEOTIDE-EXCISION REPAIR, DNA INCISION, 5'-TO LESION%GOBP%GO:0006296	0.30149662	0.9457113	37	0.9330675	11736
PKMTS METHYLATE HISTONE LYSINES%REACTOME%R-HSA-3214841.3	0.29158148	0.9463251	44	0.933082	4751
CELL FATE DETERMINATION%GOBP%GO:0001709	0.3360076	0.9466179	17	0.9330884	8599
GLUCAGON-TYPE LIGAND RECEPTORS%REACTOME%R-HSA-420092.1	0.4547791	1.3698452	27	0.93309975	9314
REGULATION OF DNA RECOMBINATION%GOBP%GO:0000018	0.27947813	0.94075936	77	0.9331207	8138
POSITIVE REGULATION OF PROTEIN BINDING%GOBP%GO:0032092	0.28925103	0.94824344	57	0.9331244	7537
GLUCAGON-LIKE PEPTIDE-1 (GLP1) REGULATES INSULIN SECRETION%REACTOME%R-HSA-381676.3	0.30017158	0.9459975	37	0.9331265	6094
RESPONSE TO STARVATION%GOBP%GO:0042594	0.27131093	0.95252675	136	0.93313384	8876
P53-DEPENDENT G1 DNA DAMAGE RESPONSE%REACTOME DATABASE ID RELEASE 75%69563	0.2819764	0.94012576	64	0.93317604	8770
REGULATION OF MICROTUBULE DEPOLYMERIZATION%GOBP%GO:0031114	0.31428367	0.9466778	24	0.9331946	9475
PDGFR-ALPHA SIGNALING PATHWAY%PATHWAY INTERACTION DATABASE NCI-NATURE CURATED DATA%PDGFR-ALPHA SIGNALING PATHWAY	0.32389498	0.94473535	21	0.9332107	4020
ERBB2 ERBB3 SIGNALING EVENTS%PATHWAY INTERACTION DATABASE NCI-NATURE CURATED DATA%ERBB2 ERBB3 SIGNALING EVENTS	0.2969087	0.95147586	41	0.9332138	10539
POSITIVE REGULATION OF NUCLEOCYTOPLASMIC TRANSPORT%GOBP%GO:0046824	0.28855306	0.9405174	47	0.9332223	11466
RRNA METHYLATION%GOBP%GO:0031167	0.31445995	0.9461649	25	0.9332302	6730
REGULATION OF BLOOD PRESSURE%GOBP%GO:0008217	0.2781297	0.9460598	86	0.9332356	7644
ACID SECRETION%GOBP%GO:0046717	0.2959019	0.94574744	39	0.9332356	11188
RRNA MODIFICATION%GOBP%GO:0000154	0.3044877	0.9483051	37	0.9332372	6730
SMALL CELL LUNG CANCER%WIKIPATHWAYS_20210110%WP4658%HOMO SAPIENS	0.40424803	1.3775843	80	0.9332516	5922
REGULATION OF STRIATED MUSCLE CELL DIFFERENTIATION%GOBP%GO:0051153	0.28848016	0.94080275	50	0.9332701	10532
POSITIVE REGULATION OF MITOTIC CELL CYCLE PHASE TRANSITION%GOBP%GO:1901992	0.41135195	1.3694729	63	0.9332754	6680
NEGATIVE REGULATION OF CELLULAR RESPONSE TO INSULIN STIMULUS%GOBP%GO:1900077	0.31293815	0.9448068	28	0.9332963	11546
LIVER DEVELOPMENT%GOBP%GO:0001889	0.2987831	0.9423276	33	0.9333057	8659
PURINE-CONTAINING COMPOUND BIOSYNTHETIC PROCESS%GOBP%GO:0072522	0.27007964	0.9521527	158	0.93331134	5819
PHAGOSOME ACIDIFICATION%GOBP%GO:0090383	0.31509697	0.9449995	26	0.93331915	8331
PID_THROMBIN_PAR1_PATHWAY%MSIGDB_C2%PID_THROMBIN_PAR1_PATHWAY	0.3070239	0.948788	29	0.9333214	11105
MATURATION OF 5.8S RRNA FROM TRICISTRONIC RRNA TRANSCRIPT (SSU-RRNA, 5.8S RRNA, LSU-RRNA)%GOBP%GO:0000466	0.46335334	1.3768442	22	0.933328	2608
MAPK SIGNALING PATHWAY%WIKIPATHWAYS_20210110%WP382%HOMO SAPIENS	0.26597738	0.93914336	164	0.9333419	12418
MITOTIC G2/M TRANSITION CHECKPOINT%GOBP%GO:0044818	0.30618298	0.9405712	30	0.93334657	12343
NEGATIVE REGULATION OF T CELL ACTIVATION%GOBP%GO:0050868	0.28974947	0.9458041	55	0.93335223	6153
NEGATIVE REGULATION OF TOLL-LIKE RECEPTOR SIGNALING PATHWAY%GOBP%GO:0034122	0.30661154	0.94087154	26	0.9333656	12655
PROTEIN LIPIDATION%GOBP%GO:0006497	0.28088003	0.9467103	78	0.93338	5699
RESPONSE TO TEMPERATURE STIMULUS%GOBP%GO:0009266	0.28114998	0.95161486	76	0.9333873	5721
ALCOHOL CATABOLIC PROCESS%GOBP%GO:0046164	0.30889323	0.9410766	30	0.9333898	8828
TRANSMEMBRANE RECEPTOR PROTEIN SERINE/THREONINE KINASE SIGNALING PATHWAY%GOBP%GO:0007178	0.26962546	0.9398378	145	0.9333949	9811
ESR-MEDIATED SIGNALING%REACTOME%R-HSA-8939211.2	0.2699919	0.95171386	169	0.9334007	9790
MISMATCH REPAIR%GOBP%GO:0006298	0.30335537	0.9409648	32	0.9334051	8335
GLYCEROLIPIDS AND GLYCEROPHOSPHOLIPIDS%WIKIPATHWAYS_20210110%WP4722%HOMO SAPIENS	0.33325702	0.95191354	19	0.93341124	12891
APOPTOTIC DNA FRAGMENTATION%GOBP%GO:0006309	0.34394446	0.9401259	15	0.93343717	6105
PROTEIN LOCALIZATION TO CHROMOSOME%GOBP%GO:0034502	0.28779328	0.95128065	59	0.93345296	7587
IN UTERO EMBRYONIC DEVELOPMENT%GOBP%GO:0001701	0.29275298	0.9475978	49	0.9334573	707
NEGATIVE REGULATION OF TOR SIGNALING%GOBP%GO:0032007	0.2991105	0.95148295	39	0.9334636	8746
NEGATIVE REGULATION OF B CELL ACTIVATION%GOBP%GO:0050869	0.34363613	0.948322	15	0.93346417	11724
MRNA-CONTAINING RIBONUCLEOPROTEIN COMPLEX EXPORT FROM NUCLEUS%GOBP%GO:0071427	0.27578908	0.9476838	102	0.93349135	5209
REGULATION OF RHODOPSIN MEDIATED SIGNALING PATHWAY%GOBP%GO:0022400	0.34101814	0.9423518	15	0.93349904	6484
HISTONE LYSINE DEMETHYLATION%GOBP%GO:0070076	0.3182861	0.94482577	22	0.9335085	11808
PID_CDC42_REG_PATHWAY%MSIGDB_C2%PID_CDC42_REG_PATHWAY	0.5174724	1.4915528	18	0.93351305	3720
MITOCHONDRIAL TRANSLATION%REACTOME DATABASE ID RELEASE 75%5368287	0.27406815	0.9401966	94	0.93351734	6216
POSITIVE REGULATION OF PURINE NUCLEOTIDE BIOSYNTHETIC PROCESS%GOBP%GO:1900373	0.30861375	0.9521699	28	0.93353844	8383
TRANSPORT OF RIBONUCLEOPROTEINS INTO THE HOST NUCLEUS%REACTOME DATABASE ID RELEASE 75%168271	0.3110098	0.94028825	29	0.9335498	4994
CARDIAC CELL DEVELOPMENT%GOBP%GO:0055006	0.30587748	0.93511647	28	0.93355644	7365
POSITIVE REGULATION OF GLUCOSE TRANSMEMBRANE TRANSPORT%GOBP%GO:0010828	0.30574924	0.93915915	28	0.933563	8214
REGULATION OF EXOSOMAL SECRETION%GOBP%GO:1903541	0.34599498	0.9421315	17	0.933565	10507
GALANIN RECEPTOR PATHWAY%WIKIPATHWAYS_20210110%WP4970%HOMO SAPIENS	0.34457445	0.9519526	16	0.93358076	9607
FIBROBLAST GROWTH FACTOR RECEPTOR SIGNALING PATHWAY%GOBP%GO:0008543	0.28471422	0.9343631	64	0.9335842	7470
REGULATION OF LYMPHOCYTE MEDIATED IMMUNITY%GOBP%GO:0002706	0.2805358	0.9487886	75	0.933588	4842
RIBOSOME ASSEMBLY%GOBP%GO:0042255	0.27914125	0.9349921	59	0.9335974	10237
CARBOHYDRATE DERIVATIVE TRANSPORT%GOBP%GO:1901264	0.2931207	0.9517414	45	0.9335975	11179
REGULATION OF DENDRITE DEVELOPMENT%GOBP%GO:0050773	0.27646917	0.94261575	92	0.9336059	11575
POSITIVE REGULATION OF INTRINSIC APOPTOTIC SIGNALING PATHWAY%GOBP%GO:2001244	0.47144696	1.4972492	41	0.9336158	6568
REGULATION OF SYNAPTIC VESICLE TRANSPORT%GOBP%GO:1902803	0.30416197	0.93891925	29	0.93362385	4793
RETROGRADE TRANSPORT, ENDOSOME TO GOLGI%GOBP%GO:0042147	0.27437115	0.9410842	90	0.9336306	6252
NEURON CELLULAR HOMEOSTASIS%GOBP%GO:0070050	0.33170715	0.9467145	18	0.93363476	622
EXTRACELLULAR MATRIX ORGANIZATION%REACTOME%R-HSA-1474244.2	0.26352614	0.9500925	247	0.9336592	6318
METHYLATION%GOBP%GO:0032259	0.26504508	0.9413804	199	0.93366855	9175
SIGNALING BY ROBO RECEPTORS%REACTOME DATABASE ID RELEASE 75%376176	0.26315546	0.94679624	209	0.9336907	8277
RIBONUCLEOPROTEIN COMPLEX EXPORT FROM NUCLEUS%GOBP%GO:0071426	0.26939136	0.9353765	118	0.9336925	5209
REGULATION OF INTRACELLULAR PROTEIN TRANSPORT%GOBP%GO:0033157	0.26553765	0.9392136	176	0.9336942	8514
DNA-DEPENDENT DNA REPLICATION MAINTENANCE OF FIDELITY%GOBP%GO:0045005	0.28852084	0.9435185	47	0.9336984	8860
PEROXISOMAL TRANSPORT%GOBP%GO:0043574	0.2800602	0.9344146	64	0.93371886	7116
PINK1-PRKN MEDIATED MITOPHAGY%REACTOME%R-HSA-5205685.3	0.32174328	0.9395006	21	0.9337332	2992
RESPONSE TO STEROID HORMONE%GOBP%GO:0048545	0.27796257	0.9521963	88	0.9337425	8165
INSERTION OF TAIL-ANCHORED PROTEINS INTO THE ENDOPLASMIC RETICULUM MEMBRANE%REACTOME DATABASE ID RELEASE 75%9609523	0.32694748	0.93514895	19	0.9337435	3901
SYNAPTIC_VESICLE_TRAFFICKING%PANTHER PATHWAY%P05734	0.3162043	0.94235826	24	0.93374836	8454
ACTIN FILAMENT BUNDLE ASSEMBLY%GOBP%GO:0051017	0.290061	0.94185317	50	0.9337575	8605
NETWORK	0.28786227	0.94738376	54	0.9337649	10994
PROTEIN MANNOSYLATION%GOBP%GO:0035268	0.31967404	0.93481636	22	0.9337667	9692
RNA POLYMERASE III TRANSCRIPTION INITIATION%REACTOME%R-HSA-76046.2	0.30484593	0.94974464	36	0.93377364	8398
EPHB FORWARD SIGNALING%PATHWAY INTERACTION DATABASE NCI-NATURE CURATED DATA%EPHB FORWARD SIGNALING	0.29821035	0.9433807	39	0.93378305	11091
RESOLUTION OF D-LOOP STRUCTURES THROUGH HOLLIDAY JUNCTION INTERMEDIATES%REACTOME%R-HSA-5693568.1	0.30291533	0.94194704	31	0.9337874	12221
SODIUM ION TRANSMEMBRANE TRANSPORT%GOBP%GO:0035725	0.28469524	0.9438932	68	0.9337906	10146
G1 S DNA DAMAGE CHECKPOINTS%REACTOME DATABASE ID RELEASE 75%69615	0.28274775	0.9346945	66	0.9338084	8770
PROTEIN ALKYLATION%GOBP%GO:0008213	0.27494827	0.94243425	93	0.93381613	6730
SIGNALING BY FGFR3%REACTOME%R-HSA-5654741.2	0.3091161	0.9498266	29	0.9338378	7222
REGULATION OF STEROL BIOSYNTHETIC PROCESS%GOBP%GO:0106118	0.29899007	0.9499308	41	0.93383884	5114
REGULATION OF ANOIKIS%GOBP%GO:2000209	0.33505607	0.9426264	20	0.9338435	6802
FLT3 SIGNALING%REACTOME%R-HSA-9607240.5	0.30336305	0.9487934	30	0.93384534	7159
REGULATION OF B CELL MEDIATED IMMUNITY%GOBP%GO:0002712	0.31811056	0.9501206	24	0.93385524	7575
RESPONSE OF EIF2AK1 (HRI) TO HEME DEFICIENCY%REACTOME DATABASE ID RELEASE 75%9648895	0.34007642	0.9412035	15	0.9338661	7159
EYE MORPHOGENESIS%GOBP%GO:0048592	0.27850023	0.93520314	75	0.93386966	7094
BIOCARTA_EIF4_PATHWAY%MSIGDB_C2%BIOCARTA_EIF4_PATHWAY	0.3231457	0.9437645	21	0.93387115	11345
UCH PROTEINASES%REACTOME DATABASE ID RELEASE 75%5689603	0.27868107	0.9468202	88	0.93388253	8770
TOLL-LIKE RECEPTOR SIGNALING RELATED TO MYD88%WIKIPATHWAYS_20210110%WP3858%HOMO SAPIENS	0.45962393	1.3810831	25	0.93388414	5609
CHOLESTEROL METABOLIC PROCESS%GOBP%GO:0008203	0.286856	0.9392369	58	0.9338901	6142
CELL VOLUME HOMEOSTASIS%GOBP%GO:0006884	0.3221365	0.9410851	21	0.9338918	10168
NITRIC OXIDE METABOLIC PROCESS%GOBP%GO:0046209	0.34427103	0.9338252	15	0.9338925	5076
G1 S-SPECIFIC TRANSCRIPTION%REACTOME DATABASE ID RELEASE 75%69205	0.31168953	0.9435416	28	0.9339026	3930
ESTABLISHMENT OF MELANOSOME LOCALIZATION%GOBP%GO:0032401	0.32582673	0.9416936	19	0.93391514	2114
COLLAGEN METABOLIC PROCESS%GOBP%GO:0032963	0.29445708	0.94148713	41	0.9339279	6584
POSITIVE REGULATION OF HORMONE SECRETION%GOBP%GO:0046887	0.28319135	0.9413805	65	0.9339309	11558
DETERMINATION OF LEFT/RIGHT SYMMETRY%GOBP%GO:0007368	0.2860687	0.94363415	62	0.933935	7416
APOPTOSIS%WIKIPATHWAYS_20210110%WP254%HOMO SAPIENS	0.41084245	1.3700664	66	0.9339425	6568
PHOTODYNAMIC THERAPY-INDUCED HIF-1 SURVIVAL SIGNALING%WIKIPATHWAYS_20210110%WP3614%HOMO SAPIENS	0.31775883	0.9344286	23	0.9339428	6322
POSITIVE REGULATION OF MRNA METABOLIC PROCESS%GOBP%GO:1903313	0.28574154	0.950495	70	0.93394285	6970
OPIOID SIGNALLING%REACTOME%R-HSA-111885.1	0.27772573	0.9415833	87	0.9339447	6342
LIPOPROTEIN METABOLIC PROCESS%GOBP%GO:0042157	0.27187705	0.93537915	91	0.93394625	7764
RESPONSE TO ZINC ION%GOBP%GO:0010043	0.30708465	0.9395119	26	0.9339682	2525
NEGATIVE REGULATION OF FIBROBLAST PROLIFERATION%GOBP%GO:0048147	0.33357248	0.93858093	17	0.93397665	10831
VALIDATED TRANSCRIPTIONAL TARGETS OF DELTANP63 ISOFORMS%PATHWAY INTERACTION DATABASE NCI-NATURE CURATED DATA%VALIDATED TRANSCRIPTIONAL TARGETS OF DELTANP63 ISOFORMS	0.28870544	0.9345243	45	0.9339784	9661
POSITIVE REGULATION OF DENDRITIC SPINE DEVELOPMENT%GOBP%GO:0060999	0.32385728	0.9470944	23	0.93398327	11273
STRIATED MUSCLE TISSUE DEVELOPMENT%GOBP%GO:0014706	0.2740627	0.9488456	108	0.9339837	8428
RAF MAP KINASE CASCADE%REACTOME%R-HSA-5673001.10	0.26012576	0.9336828	229	0.9339924	8439
INORGANIC ION IMPORT ACROSS PLASMA MEMBRANE%GOBP%GO:0099587	0.28224385	0.9338864	63	0.93400073	10168
VIRAL GENE EXPRESSION%GOBP%GO:0019080	0.27151233	0.9501704	132	0.93400216	5120
PIGMENT GRANULE ORGANIZATION%GOBP%GO:0048753	0.3125146	0.9439085	25	0.93401587	10410
REGULATION OF ORGAN GROWTH%GOBP%GO:0046620	0.29486078	0.93398046	35	0.9340238	9435
REGULATION OF LIPID STORAGE%GOBP%GO:0010883	0.29926425	0.949036	36	0.9340278	10145
EXPORT OF VIRAL RIBONUCLEOPROTEINS FROM NUCLEUS%REACTOME DATABASE ID RELEASE 75%168274	0.30664635	0.94275916	30	0.9340326	4994
P53-DEPENDENT G1 S DNA DAMAGE CHECKPOINT%REACTOME DATABASE ID RELEASE 75%69580	0.2819764	0.93927616	64	0.93404627	8770
POSITIVE REGULATION OF INTERLEUKIN-6 PRODUCTION%GOBP%GO:0032755	0.28586918	0.9354448	47	0.934048	6298
BARD1 SIGNALING EVENTS%PATHWAY INTERACTION DATABASE NCI-NATURE CURATED DATA%BARD1 SIGNALING EVENTS	0.3098367	0.9489172	28	0.934074	8740
PID_INTEGRIN1_PATHWAY%MSIGDB_C2%PID_INTEGRIN1_PATHWAY	0.29061803	0.9355333	47	0.93408805	9272
BIOCARTA_CELLCYCLE_PATHWAY%MSIGDB_C2%BIOCARTA_CELLCYCLE_PATHWAY	0.32786593	0.9426337	19	0.9340884	10113
POSITIVE REGULATION OF NEURON APOPTOTIC PROCESS%GOBP%GO:0043525	0.3219586	0.9356162	20	0.9341226	2049
RIBONUCLEOSIDE TRIPHOSPHATE BIOSYNTHETIC PROCESS%GOBP%GO:0009201	0.28323525	0.9468244	62	0.9341359	7952
RENAL TUBULE DEVELOPMENT%GOBP%GO:0061326	0.29796797	0.93861765	36	0.9341476	8355

POSITIVE REGULATION OF CELL JUNCTION ASSEMBLY%GOBP%GO:1901890	0.30643055	0.93403625	27	0.93415153	7537
TOLL-LIKE RECEPTOR SIGNALING PATHWAY%WIKIPATHWAYS_20210110%WP75%HOMO SAPIENS	0.41127193	1.3707231	61	0.9341643	5640
ALCOHOL METABOLIC PROCESS%GOBP%GO:0006066	0.262795	0.9428089	197	0.9341706	5788
E2F MEDIATED REGULATION OF DNA REPLICATION%REACTOME DATABASE ID RELEASE 75%113510	0.32435158	0.95050615	22	0.9341816	5375
CELLULAR RESPONSE TO VIRUS%GOBP%GO:0098586	0.30515248	0.94949013	31	0.934182	3692
SUMOYLATION%REACTOME%R-HSA-2990846.4	0.2673347	0.94711655	162	0.93419766	5189
POSITIVE REGULATION OF NUCLEOTIDE BIOSYNTHETIC PROCESS%GOBP%GO:0030810	0.30861375	0.943932	28	0.9342197	8383
HEMOPOIESIS%GOBP%GO:0030097	0.26496103	0.95018786	248	0.9342245	7389
NEPHRON EPITHELIUM MORPHOGENESIS%GOBP%GO:0072088	0.30454037	0.9430947	31	0.9342459	7674
MESODERM FORMATION%GOBP%GO:0001707	0.46925735	1.3824716	23	0.9342779	5634
SECONDARY ALCOHOL BIOSYNTHETIC PROCESS%GOBP%GO:1902653	0.30823714	0.94904286	30	0.9342797	3693
THE CANONICAL RETINOID CYCLE IN RODS (TWILIGHT VISION)%REACTOME DATABASE ID RELEASE 75%2453902	0.34284282	0.949348	16	0.9342845	8805
REGULATION OF EXOCYTOSIS%GOBP%GO:0017157	0.26994383	0.94286853	137	0.934289	12252
MRNA DESTABILIZATION%GOBP%GO:0061157	0.3052123	0.9332589	28	0.9342891	6970
INTERLEUKIN-12-MEDIATED SIGNALING PATHWAY%GOBP%GO:0035722	0.29655692	0.9429509	39	0.9343329	1835
RECOMBINATIONAL REPAIR%GOBP%GO:0000725	0.279028	0.94685286	91	0.9343423	8243
CELLULAR HORMONE METABOLIC PROCESS%GOBP%GO:0034754	0.28123483	0.93562746	63	0.9343498	4928
REGULATION OF PROTEIN TARGETING TO MITOCHONDRION%GOBP%GO:1903214	0.30063975	0.950542	38	0.9343536	10382
HEME BIOSYNTHETIC PROCESS%GOBP%GO:0006783	0.34293136	0.9491125	16	0.93436664	3593
POSITIVE REGULATION OF TELOMERE MAINTENANCE VIA TELOMERE LENGTHENING%GOBP%GO:1904358	0.3054401	0.95019686	35	0.93446964	9510
REGULATION OF HORMONE SECRETION%GOBP%GO:0046883	0.26372364	0.9333869	160	0.93448925	11374
SEX DIFFERENTIATION%GOBP%GO:0007548	0.27356854	0.9332695	84	0.93452036	11443
DEVELOPMENTAL GROWTH INVOLVED IN MORPHOGENESIS%GOBP%GO:0060560	0.28633484	0.93563294	51	0.9345949	7085
POSITIVE REGULATION OF EPITHELIAL CELL DIFFERENTIATION%GOBP%GO:0030858	0.29531515	0.94912755	42	0.9345951	1740
REGULATION OF MACROPHAGE ACTIVATION%GOBP%GO:0043030	0.30912083	0.9505454	29	0.93461597	3943
BETA1 ADRENERGIC RECEPTOR SIGNALING PATHWAY%PANTHER PATHWAY%P04377	0.29931912	0.93182045	30	0.934765	10986
LONG-TERM POTENTIATION%REACTOME%R-HSA-9620244.2	0.46877575	1.3827788	21	0.93476975	5700
GENES CONTROLLING RENAL NEPHROGENESIS%WIKIPATHWAYS_20210110%WP4823%HOMO SAPIENS	0.31179562	0.95069295	29	0.93477035	8541
SPHINGOLIPID METABOLISM (GENERAL OVERVIEW)%WIKIPATHWAYS_20210110%WP4725%HOMO SAPIENS	0.32332712	0.9505827	20	0.9347786	5616
16P11.2 DISTAL DELETION SYNDROME%WIKIPATHWAYS_20210110%WP4950%HOMO SAPIENS	0.49677953	1.370968	15	0.9347858	904
NEPHRON MORPHOGENESIS%GOBP%GO:0072028	0.30454037	0.9356548	31	0.93480384	7674
CD28 CO-STIMULATION%REACTOME DATABASE ID RELEASE 75%389356	0.4919515	1.4843892	27	0.93482447	4366
TRANSLATIONAL INITIATION%GOBP%GO:0006413	0.2670942	0.9329301	123	0.9348556	6626
PROTEIN TETRAMERIZATION%GOBP%GO:0051262	0.28340915	0.93281096	61	0.9348904	4032
PYRIMIDINE NUCLEOTIDE METABOLIC PROCESS%GOBP%GO:0006220	0.3056502	0.9356946	30	0.93496907	4743
CARDIAC VENTRICLE DEVELOPMENT%GOBP%GO:0003231	0.2800337	0.9318248	65	0.9350097	7849
REGULATION OF BEHAVIOR%GOBP%GO:0050795	0.3059039	0.9357575	30	0.9350701	5957
PID_BETA_CATENIN_NUC_PATHWAY%MSIGDB_C2%PID_BETA_CATENIN_NUC_PATHWAY	0.28842184	0.93581873	49	0.9351638	11245
FORMATION OF THE BETA-CATENIN:TCF TRANSACTIVATING COMPLEX%REACTOME DATABASE ID RELEASE 75%201722	0.42545924	1.3769457	48	0.9351684	4464
REGULATION OF CELL-SUBSTRATE ADHESION%GOBP%GO:0010810	0.26876152	0.93787855	143	0.9352044	6992
NEGATIVE REGULATION OF PHOSPHOPROTEIN PHOSPHATASE ACTIVITY%GOBP%GO:0032515	0.5146665	1.4978613	20	0.93522954	3289
TRANS-GOLGI NETWORK VESICLE BUDDING%REACTOME%R-HSA-199992.3	0.27946272	0.9318311	72	0.93525034	8909
INORGANIC CATION IMPORT ACROSS PLASMA MEMBRANE%GOBP%GO:0098659	0.28224385	0.93192315	63	0.9352742	10168
NEGATIVE REGULATION OF MITOTIC SISTER CHROMATID SEGREGATION%GOBP%GO:0033048	0.45801038	1.3712361	26	0.93530786	8983
NUCLEOSIDE TRIPHOSPHATE BIOSYNTHETIC PROCESS%GOBP%GO:0009142	0.27634686	0.93585455	72	0.93534154	7952
CENTROMERE COMPLEX ASSEMBLY%GOBP%GO:0034508	0.2952439	0.93251234	37	0.93536884	8983
PDGF PATHWAY%WIKIPATHWAYS_20210110%WP2526%HOMO SAPIENS	0.3057973	0.9379104	29	0.93538606	10579
PROTEIN DEACYLATION%GOBP%GO:0035601	0.28288335	0.932393	59	0.9354026	5517
PROCESSING OF DNA DOUBLE-STRAND BREAK ENDS%REACTOME%R-HSA-5693607.1	0.276747	0.93144166	73	0.93541163	12765
B CELL RECEPTOR SIGNALING PATHWAY%WIKIPATHWAYS_20210110%WP23%HOMO SAPIENS	0.2757086	0.9313372	69	0.9354126	10960
RUNX2 REGULATES BONE DEVELOPMENT%REACTOME DATABASE ID RELEASE 75%8941326	0.31222498	0.9359309	26	0.9354176	9509
SIGNALING BY MET%REACTOME%R-HSA-6806834.2	0.27872708	0.9322796	61	0.9354351	4020
MITOCHONDRIAL GENE EXPRESSION%GOBP%GO:0140053	0.26682383	0.93206346	137	0.935457	5912
PROTEIN METHYLATION%GOBP%GO:0006479	0.27494827	0.9319362	93	0.93550116	6730
LINOLEIC ACID METABOLIC PROCESS%GOBP%GO:0043651	0.48782185	1.3701785	17	0.9355115	6972
REGULATION OF OSSIFICATION%GOBP%GO:0030278	0.26776335	0.93213403	117	0.93553936	9803
BIOCARTA_MAPK_PATHWAY%MSIGDB_C2%BIOCARTA_MAPK_PATHWAY	0.2805684	0.9376263	66	0.9355753	12418
SUMOYLATION OF DNA DAMAGE RESPONSE AND REPAIR PROTEINS%REACTOME DATABASE ID RELEASE 75%3108214	0.28009722	0.93116045	72	0.93559194	4994
PROTEIN O-LINKED MANNOSYLATION%GOBP%GO:0035269	0.33234352	0.93052185	18	0.93561345	9546
BILE ACID METABOLIC PROCESS%GOBP%GO:0008206	0.31295356	0.9359418	26	0.9356459	5259
LIPID CATABOLIC PROCESS%GOBP%GO:0016042	0.2639742	0.93748087	172	0.9356645	9431
RNA POLYMERASE I TRANSCRIPTION TERMINATION%REACTOME%R-HSA-73863.4	0.4567862	1.3725797	28	0.93568337	6737
REGULATION OF STEROID BIOSYNTHETIC PROCESS%GOBP%GO:0050810	0.28019038	0.9310108	70	0.9357062	5114
PROTEIN AUTOPHOSPHORYLATION%GOBP%GO:0046777	0.2659629	0.9361148	148	0.93571717	10650
PSEUDOURIDINE SYNTHESIS%GOBP%GO:0001522	0.33455148	0.9309031	17	0.9357173	5870
REGULATION OF PROTEIN EXPORT FROM NUCLEUS%GOBP%GO:0046825	0.3024146	0.9362007	31	0.93574876	7405
POSITIVE REGULATION OF NITRIC OXIDE BIOSYNTHETIC PROCESS%GOBP%GO:0045429	0.3283425	0.93599886	18	0.9357596	8541
REGULATION OF DEPHOSPHORYLATION%GOBP%GO:0035303	0.26617914	0.93067086	132	0.93576586	11534
NEGATIVE REGULATION OF AXON EXTENSION%GOBP%GO:0030517	0.29861727	0.9305473	29	0.93581086	5391
PTERIDINE-CONTAINING COMPOUND METABOLIC PROCESS%GOBP%GO:0042558	0.31312484	0.93072504	23	0.93589526	1784
REGULATION OF REACTIVE OXYGEN SPECIES METABOLIC PROCESS%GOBP%GO:2000377	0.2688315	0.93716806	112	0.935911	6382
NEGATIVE REGULATION OF LEUKOCYTE MEDIATED IMMUNITY%GOBP%GO:0002704	0.3210186	0.93727535	20	0.93591255	5816
G2 M DNA DAMAGE CHECKPOINT%REACTOME DATABASE ID RELEASE 75%69473	0.27360073	0.9362007	70	0.9360088	12765
REGULATION OF SYNAPTIC VESICLE EXOCYTOSIS%GOBP%GO:2000300	0.3134071	0.9363019	25	0.93602496	4793
SIGNALING IN MAST CELLS	0.4186098	1.3714483	52	0.936211	4366
REGULATION OF KIDNEY DEVELOPMENT%GOBP%GO:0090183	0.30443627	0.936926	35	0.9362618	7094
PHOTODYNAMIC THERAPY-INDUCED UNFOLDED PROTEIN RESPONSE%WIKIPATHWAYS_20210110%WP3613%HOMO SAPIENS	0.4738823	1.372843	19	0.93626374	5362
REGULATION OF MRNA STABILITY%GOBP%GO:0043488	0.26424852	0.9363075	165	0.9362674	4704
RNA METHYLATION%GOBP%GO:0001510	0.27850452	0.92981017	71	0.93634516	10173
RRNA TRANSCRIPTION%GOBP%GO:0009303	0.33680192	0.93637174	17	0.9363601	12134
LEUKOCYTE DIFFERENTIATION%GOBP%GO:0002521	0.26731643	0.9364298	140	0.93645763	6299
CELLULAR LIPID CATABOLIC PROCESS%GOBP%GO:0044242	0.26514423	0.92985684	151	0.93648	9431
WNT SIGNALING PATHWAY AND PLURIPOTENCY%WIKIPATHWAYS_20210110%WP399%HOMO SAPIENS	0.40564093	1.3829038	79	0.9365336	3237
CXCR4-MEDIATED SIGNALING EVENTS%PATHWAY INTERACTION DATABASE NCI-NATURE CURATED DATA%CXCR4-MEDIATED SIGNALING EVENTS	0.2789643	0.9365991	72	0.9365555	6927
CELLULAR RESPONSE TO STARVATION%GOBP%GO:0009267	0.26874143	0.93649274	119	0.9365559	8876
CYCLIC PURINE NUCLEOTIDE METABOLIC PROCESS%GOBP%GO:0052652	0.5361138	1.4849331	17	0.9366133	3159
MACROMOLECULE DEACYLATION%GOBP%GO:0098732	0.28288335	0.9299033	59	0.9366258	5517
LNCRNA INVOLVEMENT IN CANONICAL WNT SIGNALING AND COLORECTAL CANCER%WIKIPATHWAYS_20210110%WP4258%HOMO SAPIENS	0.2759217	0.9366478	73	0.9367011	10999
SKELETAL SYSTEM MORPHOGENESIS%GOBP%GO:0048705	0.27478558	0.9299413	81	0.9367813	6311
MAINTENANCE OF PERMEABILITY OF BLOOD-BRAIN BARRIER%GOBP%GO:0035633	0.45449615	1.3752621	28	0.9368222	5295
REGULATION OF T CELL RECEPTOR SIGNALING PATHWAY%GOBP%GO:0050856	0.47719088	1.3925565	21	0.9369986	2588
CELLULAR RESPONSE TO ALKALOID%GOBP%GO:0071312	0.50188065	1.3831302	16	0.93741214	3564
CAMERA-TYPE EYE DEVELOPMENT%GOBP%GO:0043010	0.2666171	0.9292379	119	0.9374992	9402
PRODUCTION OF MOLECULAR MEDIATOR OF IMMUNE RESPONSE%GOBP%GO:0002440	0.43795595	1.3961182	39	0.93763286	9018
REGULATION OF EXTENT OF CELL GROWTH%GOBP%GO:0061387	0.41719496	1.3932807	61	0.9376358	2093
HALLMARK_PEROXISOME%MSIGDB_C2%HALLMARK_PEROXISOME	0.27624598	0.9290788	78	0.93764156	4690
WNT/BETA-CATENIN SIGNALING PATHWAY IN LEUKEMIA%WIKIPATHWAYS_20210110%WP3658%HOMO SAPIENS	0.313406	0.9289291	21	0.9377525	7568
SUMOYLATION OF DNA REPLICATION PROTEINS%REACTOME DATABASE ID RELEASE 75%4615885	0.29055503	0.92669755	43	0.9377826	5464
REGULATION OF CELLULAR KETONE METABOLIC PROCESS%GOBP%GO:0010565	0.26460168	0.9267751	143	0.93784714	8383
BLOOD VESSEL MORPHOGENESIS%GOBP%GO:0048514	0.2594036	0.926854	206	0.93791556	6547
REGULATION OF BMP SIGNALING PATHWAY%GOBP%GO:0030510	0.27787283	0.9257019	66	0.93792033	6416
NEGATIVE REGULATION OF EXTRINSIC APOPTOTIC SIGNALING PATHWAY VIA DEATH DOMAIN RECEPTORS%GOBP%GO:1902042	0.29899156	0.9287336	29	0.93798065	4837
CARDIAC MUSCLE CELL CONTRACTION%GOBP%GO:0086003	0.44328973	1.3715183	36	0.9380931	6324
POSITIVE REGULATION OF PROTEIN TRANSPORT%GOBP%GO:0051222	0.2586694	0.9272089	200	0.9380931	8992
PKA ACTIVATION IN GLUCAGON SIGNALLING%REACTOME%R-HSA-164378.3	0.3374815	0.9268844	16	0.93809634	4721
SUMO E3 LIGASES SUMOYLATE TARGET PROTEINS%REACTOME%R-HSA-3108232.5	0.26479492	0.92571324	156	0.9381474	5189
ENDODERM FORMATION%GOBP%GO:0001706	0.2932858	0.92589664	37	0.93818235	7447
MELANOSOME LOCALIZATION%GOBP%GO:0032400	0.3165042	0.9260769	21	0.938253	6910
REGULATION OF MULTICELLULAR ORGANISM GROWTH%GOBP%GO:0040014	0.49019176	1.3729059	16	0.9382727	2885
MAINTENANCE OF CELL POLARITY%GOBP%GO:0030011	0.5110249	1.3888327	15	0.9382855	8092
GLYCEROLIPID CATABOLIC PROCESS%GOBP%GO:0046503	0.28974152	0.92690545	40	0.93830556	4075
RESPONSE TO INTERFERON-BETA%GOBP%GO:0035456	0.31995052	0.92542297	20	0.93833405	6258
RANKL%NETPATH%RANKL	0.28086013	0.92573786	60	0.9383389	14497
AMP-ACTIVATED PROTEIN KINASE (AMPK) SIGNALING%WIKIPATHWAYS_20210110%WP1403%HOMO SAPIENS	0.2856	0.9259345	48	0.93834126	4268
REGULATION OF EXTRINSIC APOPTOTIC SIGNALING PATHWAY%GOBP%GO:2001236	0.26882318	0.92720926	101	0.9383475	5981
5HT4 TYPE RECEPTOR MEDIATED SIGNALING PATHWAY%PANTHER PATHWAY%P04376	0.30488294	0.92846966	28	0.93835676	10042
NEGATIVE REGULATION OF LIPID METABOLIC PROCESS%GOBP%GO:0045833	0.27537215	0.92731434	65	0.93836546	10931
SEROTONIN NEUROTRANSMITTER RELEASE CYCLE%REACTOME%R-HSA-181429.3	0.32736012	0.928039	17	0.93837935	9035
HETEROTRIMERIC G-PROTEIN SIGNALING PATHWAY-GI ALPHA AND GS ALPHA MEDIATED PATHWAY%PANTHER PATHWAY%P00026	0.26619515	0.92783576	126	0.9383821	9374
NEGATIVE REGULATION OF INTRACELLULAR TRANSPORT%GOBP%GO:0032387	0.4347913	1.392762	44	0.93839616	2662
PID_ERBB1_INTERNALIZATION_PATHWAY%MSIGDB_C2%PID_ERBB1_INTERNALIZATION_PATHWAY	0.30150324	0.9269632	31	0.9384262	4268
DIGESTIVE SYSTEM DEVELOPMENT%GOBP%GO:0055123	0.28568476	0.9274963	50	0.93842864	9324
RESPONSE TO OXIDATIVE STRESS%GOBP%GO:0006979	0.25708687	0.9261042	233	0.9384388	11117
NEGATIVE REGULATION OF INTRACELLULAR STEROID HORMONE RECEPTOR SIGNALING PATHWAY%GOBP%GO:0033144	0.29942286	0.9279087	30	0.9384403	11365
REGULATION OF DENDRITIC SPINE MORPHOGENESIS%GOBP%GO:0061001	0.29452667	0.926313	30	0.9384445	11421
PROCESSIVE SYNTHESIS ON THE LAGGING STRAND%REACTOME%R-HSA-69183.2	0.33508632	0.927565	15	0.938513	1141
EXPORT ACROSS PLASMA MEMBRANE%GOBP%GO:0140115	0.3060621	0.9273593	29	0.93851477	5269

PID_FGF_PATHWAY%MSIGDB_C2%PID_FGF_PATHWAY	0.28929326	0.9280425	39	0.9386274	12638
CHOLESTEROL METABOLISM (INCLUDES BOTH BLOCH AND KANDUTSCH-RUSSELL PATHWAYS)%WIKIPATHWAYS_20210110%WP4718%HOMO SAPIENS	0.29244694	0.9276133	37	0.9386516	5642
LEPTIN SIGNALING PATHWAY%WIKIPATHWAYS_20210110%WP2034%HOMO SAPIENS	0.27737173	0.9261227	67	0.9386536	13549
REGULATION OF ENDOPLASMIC RETICULUM UNFOLDED PROTEIN RESPONSE%GOBP%GO:1900101	0.45612404	1.3731892	27	0.93869865	7342
NON-MOTILE CILIUM ASSEMBLY%GOBP%GO:1905515	0.3068594	0.9281115	27	0.9387117	2865
REGULATION OF AMYLOID-BETA FORMATION%GOBP%GO:1902003	0.43641055	1.373533	32	0.93878984	6101
PEPTIDYL-TYROSINE DEPHOSPHORYLATION%GOBP%GO:0035335	0.29747564	0.9251111	32	0.9388312	10994
TRANSLESION SYNTHESIS BY POLK%REACTOME%R-HSA-5655862.1	0.32761922	0.928162	17	0.9388485	11716
HEDGEHOG SIGNALING PATHWAY%WIKIPATHWAYS_20210110%WP4249%HOMO SAPIENS	0.4493935	1.3899227	31	0.938935	5995
PI METABOLISM%REACTOME%R-HSA-1483255.5	0.27478918	0.9235526	76	0.93928075	7282
PEROXISOME ORGANIZATION%GOBP%GO:0007031	0.27076417	0.92480904	76	0.9392846	7116
FATTY ACID &BETA;-OXIDATION I%HUMANCYC%FAO-PWY	0.5513622	1.5466413	16	0.93940276	8900
POSITIVE REGULATION OF PURINE NUCLEOTIDE METABOLIC PROCESS%GOBP%GO:1900544	0.28952917	0.92337507	37	0.9394706	6322
CELLULAR RESPONSE TO ACETYLCHOLINE%GOBP%GO:1905145	0.46800548	1.3836052	24	0.9394814	5169
SENSORY PERCEPTION%GOBP%GO:0007600	0.25848812	0.92355967	200	0.9395152	8570
MESENCHYME MORPHOGENESIS%GOBP%GO:0072132	0.45065796	1.3737711	31	0.93957984	6318
TCR SIGNALING IN NAIVE CD4+ T CELLS%PATHWAY INTERACTION DATABASE NCI-NATURE CURATED DATA%TCR SIGNALING IN NAIVE CD4+ T CELLS	0.4238803	1.3962562	55	0.9395851	4523
INTERLEUKIN-12 SIGNALING%REACTOME%R-HSA-9020591.1	0.29409945	0.9232181	39	0.9396212	1835
REGULATION OF TRANSLATIONAL INITIATION%GOBP%GO:0006446	0.27527544	0.92403233	69	0.939658	11429
NUCLEOSIDE PHOSPHATE BIOSYNTHETIC PROCESS%GOBP%GO:1901293	0.25786045	0.9207578	199	0.939669	6762
SPERM-EGG RECOGNITION%GOBP%GO:0035036	0.47920564	1.3831773	20	0.9396876	1829
REGULATION OF CARDIAC MUSCLE TISSUE DEVELOPMENT%GOBP%GO:0055024	0.29327995	0.92358315	35	0.9397144	9791
HALLMARK_ESTROGEN_RESPONSE_EARLY%MSIGDB_C2%HALLMARK_ESTROGEN_RESPONSE_EARLY	0.26133728	0.92093813	159	0.9397446	11117
PROTEIN-LIPID COMPLEX ASSEMBLY%GOBP%GO:0065005	0.31607875	0.9208225	20	0.9397692	15041
NEGATIVE REGULATION OF OSTEOBLAST DIFFERENTIATION%GOBP%GO:0045668	0.3031378	0.9240823	29	0.93978703	2628
POSITIVE REGULATION OF SMALL MOLECULE METABOLIC PROCESS%GOBP%GO:0062013	0.26925707	0.9238687	92	0.9397886	6322
POSITIVE REGULATION OF NUCLEOTIDE METABOLIC PROCESS%GOBP%GO:0045981	0.28952917	0.92123556	37	0.9397927	6322
REGULATION OF OSTEOBLAST DIFFERENTIATION%GOBP%GO:0045667	0.27441424	0.9211232	76	0.9398043	11042
CELLULAR RESPONSE TO GLUCOSE STIMULUS%GOBP%GO:0071333	0.30381024	0.9236422	29	0.9398278	11416
RESPONSE TO VITAMIN%GOBP%GO:0033273	0.5010083	1.3716172	15	0.9398424	11023
PID_GMCSF_PATHWAY%MSIGDB_C2%PID_GMCSF_PATHWAY	0.30990463	0.9237373	26	0.93985045	13414
REGULATION OF RUNX2 EXPRESSION AND ACTIVITY%REACTOME%R-HSA-8939902.1	0.27470353	0.92140263	68	0.9399127	8770
NAD+ BIOSYNTHETIC PATHWAYS%WIKIPATHWAYS_20210110%WP3645%HOMO SAPIENS	0.32041106	0.9203308	18	0.9399217	6869
TRAF6 MEDIATED NF-KB ACTIVATION%REACTOME DATABASE ID RELEASE 75%933542	0.47681415	1.3933547	23	0.93994004	4417
ENDOTHELINS%PATHWAY INTERACTION DATABASE NCI-NATURE CURATED DATA%ENDOTHELINS	0.27908623	0.9209541	61	0.93995565	4995
POSITIVE REGULATION OF PROTEIN KINASE B SIGNALING%GOBP%GO:0051897	0.27013674	0.92411935	120	0.9399579	8401
CELL-MATRIX ADHESION%GOBP%GO:0007160	0.41166115	1.3889743	81	0.9400126	3002
NOTCH3 ACTIVATION AND TRANSMISSION OF SIGNAL TO THE NUCLEUS%REACTOME%R-HSA-9013507.1	0.30412498	0.9212418	25	0.9400283	10885
POSITIVE REGULATION OF STRESS-ACTIVATED MAPK CASCADE%GOBP%GO:0032874	0.26704198	0.9217711	118	0.9400605	5415
RECEPTOR CLUSTERING%GOBP%GO:0043113	0.30279714	0.92154604	29	0.9400793	6576
GLUTATHIONE DERIVATIVE BIOSYNTHETIC PROCESS%GOBP%GO:1901687	0.31547186	0.92142874	19	0.94009423	5007
RHYTHMIC PROCESS%GOBP%GO:0048511	0.2680483	0.92045957	107	0.940121	8404
MONOCARBOXYLIC ACID CATABOLIC PROCESS%GOBP%GO:0072329	0.26379132	0.9201387	96	0.94014084	9373
SIMPLIFIED INTERACTION MAP BETWEEN LOXL4 AND OXIDATIVE STRESS PATHWAY%WIKIPATHWAYS_20210110%WP3670%HOMO SAPIENS	0.32853287	0.92033976	16	0.9401559	5755
CELLULAR PROTEIN-CONTAINING COMPLEX LOCALIZATION%GOBP%GO:0034629	0.331104	0.9241372	17	0.94016397	4481
AMMONIUM ION METABOLIC PROCESS%GOBP%GO:0097164	0.26123852	0.92433715	159	0.94018507	6420
TERMINATION OF O-GLYCAN BIOSYNTHESIS%REACTOME%R-HSA-977068.1	0.32458466	0.9228634	17	0.9402293	4917
TRANSCRIPTIONAL REGULATION BY RUNX1%REACTOME DATABASE ID RELEASE 75%8878171	0.2598918	0.92421705	177	0.9402316	9483
RESISTIN AS A REGULATOR OF INFLAMMATION%WIKIPATHWAYS_20210110%WP4481%HOMO SAPIENS	0.47274923	1.4044617	22	0.9402382	5059
LONG-TERM MEMORY%GOBP%GO:0007616	0.5220105	1.4867438	18	0.94025904	1634
GTP METABOLIC PROCESS%GOBP%GO:0046039	0.50936	1.4859924	19	0.9402604	3834
GONAD DEVELOPMENT%GOBP%GO:0008406	0.28146398	0.9215739	59	0.94026804	11443
16P11.2 PROXIMAL DELETION SYNDROME%WIKIPATHWAYS_20210110%WP4949%HOMO SAPIENS	0.28429547	0.92177874	51	0.9402928	9607
REGULATION OF EXTRACELLULAR MATRIX DISASSEMBLY%GOBP%GO:0010715	0.33465686	0.921868	15	0.94034016	13727
NUCLEAR EVENTS (KINASE AND TRANSCRIPTION FACTOR ACTIVATION)%REACTOME%R-HSA-198725.2	0.27601993	0.9227007	59	0.9403568	10501
NEGATIVE REGULATION OF CELL GROWTH%GOBP%GO:0030308	0.2622933	0.922588	129	0.94037396	7546
TP53 REGULATES TRANSCRIPTION OF GENES INVOLVED IN G2 CELL CYCLE ARREST%REACTOME%R-HSA-6804114.2	0.49554822	1.390101	18	0.94038785	6040
REGULATION OF CALCIUM ION IMPORT%GOBP%GO:0090279	0.3081011	0.91990864	23	0.94044507	4996
VIRAL LIFE CYCLE%GOBP%GO:0019058	0.40238255	1.4052786	133	0.9404633	7828
CELL-CELL JUNCTION ORGANIZATION%GOBP%GO:0045216	0.40370816	1.3953136	109	0.94055134	5758
POSITIVE REGULATION OF INTRACELLULAR PROTEIN TRANSPORT%GOBP%GO:0090316	0.26465642	0.92219615	122	0.94056904	10176
TISSUE MIGRATION%GOBP%GO:0090130	0.27467972	0.92186856	68	0.9405917	12734
ONE CARBON METABOLISM AND RELATED PATHWAYS%WIKIPATHWAYS_20210110%WP3940%HOMO SAPIENS	0.30181792	0.921912	29	0.94073915	12589
MELANOSOME ORGANIZATION%GOBP%GO:0032438	0.3125146	0.91945845	25	0.9407645	10410
ACYL-COA METABOLIC PROCESS%GOBP%GO:0006637	0.27170172	0.9222175	73	0.94077027	4635
ERK1 ERK2 PATHWAY%REACTOME DATABASE ID RELEASE 75%5684996	0.25587228	0.91963995	234	0.94084436	8439
REACTIVE OXYGEN SPECIES BIOSYNTHETIC PROCESS%GOBP%GO:1903409	0.313536	0.9195267	22	0.94085515	5415
INTERLEUKIN-4 AND INTERLEUKIN-13 SIGNALING%REACTOME DATABASE ID RELEASE 75%6785807	0.27282238	0.92195	85	0.94089806	12243
PID_HIF1_TFPATHWAY%MSIGDB_C2%PID_HIF1_TFPATHWAY	0.4367288	1.3880584	38	0.9409017	7033
REGULATION OF LEUKOCYTE DIFFERENTIATION%GOBP%GO:1902105	0.2631388	0.9182845	141	0.9410029	6935
GENES RELATED TO PRIMARY CILIUM DEVELOPMENT (BASED ON CRISPR)%WIKIPATHWAYS_20210110%WP4536%HOMO SAPIENS	0.27284968	0.9222175	81	0.9410237	5745
ECTODERM DIFFERENTIATION%WIKIPATHWAYS_20210110%WP2858%HOMO SAPIENS	0.26427814	0.9181135	105	0.94116306	8145
CELLULAR ALDEHYDE METABOLIC PROCESS%GOBP%GO:0006081	0.2783447	0.9183077	50	0.9412023	7965
NEGATIVE REGULATION OF AUTOPHAGY%GOBP%GO:0010507	0.41675824	1.3837445	63	0.94121045	4150
CYTOSOLIC TRANSPORT%GOBP%GO:0016482	0.26184866	0.9191629	141	0.94121534	6252
REGULATION OF TUBE DIAMETER%GOBP%GO:0035296	0.43190396	1.4056132	50	0.9413244	6031
REGULATION OF NEUROTRANSMITTER UPTAKE%GOBP%GO:0051580	0.50022024	1.3984754	16	0.94135803	2423
REGULATION OF RNA STABILITY%GOBP%GO:0043487	0.25775018	0.9183334	174	0.94138795	10310
ZYMOGEN ACTIVATION%GOBP%GO:0031638	0.44996628	1.3963977	32	0.9414748	4002
PIGMENT GRANULE LOCALIZATION%GOBP%GO:0051875	0.3165042	0.91839373	21	0.9414917	6910
MYELOID CELL DIFFERENTIATION%GOBP%GO:0030099	0.26374105	0.918478	106	0.9415376	7258
REPRODUCTIVE STRUCTURE DEVELOPMENT%GOBP%GO:0048608	0.26611605	0.91877556	109	0.9416094	8254
DEFENSE RESPONSE TO GRAM-POSITIVE BACTERIUM%GOBP%GO:0050830	0.2966237	0.9188622	31	0.94165856	6299
CYCLIN A B1 B2 ASSOCIATED EVENTS DURING G2 M TRANSITION%REACTOME%R-HSA-69273.6	0.30636975	0.9185099	25	0.9417126	11618
MITOTIC G2 DNA DAMAGE CHECKPOINT%GOBP%GO:0007095	0.31855038	0.91776973	19	0.94172233	15247
PID_TCPTP_PATHWAY%MSIGDB_C2%PID_TCPTP_PATHWAY	0.44255182	1.3939337	33	0.941772	4268
CERAMIDE BIOSYNTHETIC PROCESS%GOBP%GO:0046513	0.4267597	1.373801	49	0.94183487	1313
APOPTOSIS-RELATED NETWORK DUE TO ALTERED NOTCH3 IN OVARIAN CANCER%WIKIPATHWAYS_20210110%WP2864%HOMO SAPIENS	0.47097307	1.4850376	33	0.94184315	5464
E3 UBIQUITIN LIGASES UBIQUITINATE TARGET PROTEINS%REACTOME%R-HSA-8866654.3	0.4314172	1.3902786	50	0.94190913	3218
DEVELOPMENTAL MATURATION%GOBP%GO:0021700	0.2672328	0.91850996	96	0.94196415	10145
REGULATION OF RAC1 ACTIVITY%PATHWAY INTERACTION DATABASE NCI-NATURE CURATED DATA%REGULATION OF RAC1 ACTIVITY	0.43939868	1.3839979	37	0.9420008	10236
REGULATION OF MYELOID LEUKOCYTE DIFFERENTIATION%GOBP%GO:0002761	0.27846244	0.9174158	56	0.9420345	6799
PATHWAYS REGULATING HIPPO SIGNALING%WIKIPATHWAYS_20210110%WP4540%HOMO SAPIENS	0.27358428	0.9175181	67	0.9420574	9771
GLYCOGEN SYNTHESIS%REACTOME DATABASE ID RELEASE 75%3322077	0.53311837	1.4557153	15	0.9421458	3244
NEGATIVE REGULATION OF WOUND HEALING%GOBP%GO:0061045	0.42721713	1.3890657	45	0.94217235	2064
PID_AVB3_OPN_PATHWAY%MSIGDB_C2%PID_AVB3_OPN_PATHWAY	0.5083651	1.4798023	21	0.9422353	4020
POSITIVE REGULATION OF G PROTEIN-COUPLED RECEPTOR SIGNALING PATHWAY%GOBP%GO:0045745	0.49313742	1.4008278	18	0.9422539	7219
COMMON PATHWAYS UNDERLYING DRUG ADDICTION%WIKIPATHWAYS_20210110%WP2636%HOMO SAPIENS	0.3016554	0.91687745	27	0.9422717	7719
RESPONSE TO TOPOLOGICALLY INCORRECT PROTEIN%GOBP%GO:0035966	0.25944683	0.91717935	158	0.94231874	5400
TELENCEPHALON DEVELOPMENT%GOBP%GO:0021537	0.26701358	0.9169382	93	0.94238615	9383
BICARBONATE TRANSPORT%GOBP%GO:0015701	0.4790022	1.4617715	27	0.9424011	4370
NEGATIVE REGULATION OF MICROTUBULE POLYMERIZATION OR DEPOLYMERIZATION%GOBP%GO:0031111	0.29648444	0.9170164	32	0.9424722	10936
POSITIVE REGULATION OF VIRAL LIFE CYCLE%GOBP%GO:1903902	0.41956118	1.3933977	60	0.94255465	4455
BIOCARTA_TOLL_PATHWAY%MSIGDB_C2%BIOCARTA_TOLL_PATHWAY	0.49136	1.4045719	19	0.9425793	4048
RAS PROTEIN SIGNAL TRANSDUCTION%GOBP%GO:0007265	0.3994775	1.4012243	141	0.9426028	6927
MULTICELLULAR ORGANISMAL WATER HOMEOSTASIS%GOBP%GO:0050891	0.45686993	1.4532706	41	0.94278353	3314
POSITIVE REGULATION OF GLIOGENESIS%GOBP%GO:0014015	0.30148742	0.91631246	28	0.9428143	9199
POSITIVE REGULATION OF INTRACELLULAR TRANSPORT%GOBP%GO:0032388	0.25909424	0.9161851	168	0.94284534	8514
BUDDING AND MATURATION OF HIV VIRION%REACTOME DATABASE ID RELEASE 75%162588	0.4544425	1.3986771	28	0.9428791	8531
ESTABLISHMENT OF RNA LOCALIZATION%GOBP%GO:0051236	0.25970292	0.9160547	144	0.94289255	5209
THIOESTER METABOLIC PROCESS%GOBP%GO:0035383	0.27170172	0.9163868	73	0.94289845	4635
RETT SYNDROME CAUSING GENES%WIKIPATHWAYS_20210110%WP4312%HOMO SAPIENS	0.29040992	0.9164061	39	0.94310343	9021
PIWI-INTERACTING RNA (PIRNA) BIOGENESIS%REACTOME DATABASE ID RELEASE 75%5601884	0.47897604	1.3973875	21	0.94310945	6350
CELLULAR RESPONSE TO INTERLEUKIN-12%GOBP%GO:0071349	0.28448465	0.91562843	41	0.94314814	1835
POSITIVE REGULATION OF BLOOD COAGULATION%GOBP%GO:0030194	0.5001892	1.406232	18	0.9431532	3051
RETINA DEVELOPMENT IN CAMERA-TYPE EYE%GOBP%GO:0060041	0.2813494	0.9154861	58	0.943226	7094
CARBOXYLIC ACID CATABOLIC PROCESS%GOBP%GO:0046395	0.25585324	0.9153549	205	0.94327193	7296
DNA CONFORMATION CHANGE%GOBP%GO:0071103	0.25804874	0.91565585	180	0.9433296	8860
POSITIVE REGULATION OF DEVELOPMENTAL GROWTH%GOBP%GO:0048639	0.4141721	1.3841858	73	0.94335276	5930
BBSOME-MEDIATED CARGO-TARGETING TO CILIUM%REACTOME DATABASE ID RELEASE 75%5620922	0.31502157	0.9152135	22	0.9433681	7827
INTEGRATED CANCER PATHWAY%WIKIPATHWAYS_20210110%WP1971%HOMO SAPIENS	0.474529	1.4747475	34	0.94344634	5331
NADP METABOLIC PROCESS%GOBP%GO:0006739	0.48756072	1.4561551	23	0.94354707	3993
REPRODUCTIVE SYSTEM DEVELOPMENT%GOBP%GO:0061458	0.26611605	0.91565883	109	0.9435729	8254
PROTEIN N-LINKED GLYCOSYLATION VIA ASPARAGINE%GOBP%GO:0018279	0.45546776	1.3965039	28	0.94357336	8988
HINDBRAIN DEVELOPMENT%GOBP%GO:0030902	0.28462103	0.91465896	45	0.94362766	7479
DETERMINATION OF BILATERAL SYMMETRY%GOBP%GO:0009855	0.27371073	0.914758	66	0.94365615	7416
CELLULAR DEFENSE RESPONSE%GOBP%GO:0006968	0.3136433	0.91441005	22	0.9436999	6519

EVENTS	0.29109976	0.9148425	36	0.9437173	8355
LIPID LOCALIZATION%GOBP%GO:0010876	0.2573941	0.91429335	179	0.9437195	8750
INTRINSIC PATHWAY FOR APOPTOSIS%REACTOME%R-HSA-109606.3	0.4283549	1.3968855	50	0.94372463	4030
CLATHRIN-MEDIATED ENDOCYTOSIS%REACTOME%R-HSA-8856828.3	0.26042944	0.9145042	133	0.9437413	10535
OLIGOSACCHARIDE METABOLIC PROCESS%GOBP%GO:0009311	0.2890634	0.91491455	38	0.94379646	13231
NEGATIVE REGULATION OF ORGANELLE ASSEMBLY%GOBP%GO:1902116	0.4481813	1.406584	37	0.9438362	4923
CLASS B 2 (SECRETIN FAMILY RECEPTORS)%REACTOME DATABASE ID RELEASE 75%373080	0.40715542	1.3738786	73	0.94384784	8582
AUTONOMIC NERVOUS SYSTEM DEVELOPMENT%GOBP%GO:0048483	0.46045455	1.3852999	27	0.94384843	3710
A6B1 AND A6B4 INTEGRIN SIGNALING%PATHWAY INTERACTION DATABASE NCI-NATURE CURATED DATA%A6B1 AND A6B4 INTEGRIN SIGNALING	0.2857905	0.9140835	45	0.9439299	10165
BIOCARTA_IL1R_PATHWAY%MSIGDB_C2%BIOCARTA_IL1R_PATHWAY	0.4722722	1.4056722	23	0.94400924	3663
CELLULAR POLYSACCHARIDE CATABOLIC PROCESS%GOBP%GO:0044247	0.5030088	1.394019	16	0.9440271	8534
LYMPHOCYTE ACTIVATION INVOLVED IN IMMUNE RESPONSE%GOBP%GO:0002285	0.4580894	1.4494957	38	0.94417113	2943
UBIQUITIN PROTEASOME PATHWAY%PANTHER PATHWAY%P00060	0.43314528	1.3944156	44	0.9442005	6671
PLATELET AGGREGATION (PLUG FORMATION)%REACTOME DATABASE ID RELEASE 75%76009	0.5225425	1.5534489	24	0.94420755	1349
DIVALENT INORGANIC CATION TRANSPORT%GOBP%GO:0072511	0.25681618	0.9138386	171	0.94426036	3251
CELLULAR RESPONSE TO AMMONIUM ION%GOBP%GO:0071242	0.47684598	1.5497291	45	0.9442774	5169
AXON GUIDANCE MEDIATED BY SEMAPHORINS%PANTHER PATHWAY%P00007	0.32412314	0.91132945	17	0.9442937	2595
AMYLOID PRECURSOR PROTEIN METABOLIC PROCESS%GOBP%GO:0042982	0.48921594	1.406949	20	0.94431627	7267
REGULATION OF CELL-CELL ADHESION MEDIATED BY CADHERIN%GOBP%GO:2000047	0.32005745	0.9117521	18	0.94433063	5001
SPHINGOLIPID METABOLISM (INTEGRATED PATHWAY)%WIKIPATHWAYS_20210110%WP4726%HOMO SAPIENS	0.30917695	0.91184986	21	0.94436234	5616
HIPPO-MERLIN SIGNALING DYSREGULATION%WIKIPATHWAYS_20210110%WP4541%HOMO SAPIENS	0.26785102	0.9123783	74	0.9443927	12347
MISCELLANEOUS TRANSPORT AND BINDING EVENTS%REACTOME DATABASE ID RELEASE 75%5223345	0.30751574	0.91226804	23	0.94440013	2228
PORPHYRIN-CONTAINING COMPOUND METABOLIC PROCESS%GOBP%GO:0006778	0.30671626	0.9114994	26	0.94440633	3593
REGULATION OF T CELL MIGRATION%GOBP%GO:2000404	0.4601258	1.3844097	24	0.94445133	5015
RESPONSE TO INTERLEUKIN-12%GOBP%GO:0070671	0.28448465	0.91135865	41	0.9444818	1835
GLUTATHIONE-MEDIATED DETOXIFICATION I%HUMANCYC%PWY-4061	0.31281438	0.9115658	20	0.9444989	5007
REGULATION OF NEUTROPHIL MIGRATION%GOBP%GO:1902622	0.30151287	0.9111019	25	0.94458264	8689
SUPERPATHWAY OF CHOLESTEROL BIOSYNTHESIS%HUMANCYC%PWY66-5	0.30049908	0.90344876	25	0.94458675	5259
MORPHOGENESIS OF AN EPITHELIAL SHEET%GOBP%GO:0002011	0.44696218	1.3902993	35	0.9445886	6802
HIV ELONGATION ARREST AND RECOVERY%REACTOME DATABASE ID RELEASE 75%167287	0.29283142	0.9118531	32	0.9446044	7291
ISOPRENOID BIOSYNTHETIC PROCESS%GOBP%GO:0008299	0.32831338	0.9031048	16	0.94461125	2849
PROSTANOID BIOSYNTHETIC PROCESS%GOBP%GO:0046457	0.31999093	0.91239023	18	0.944613	4638
HALLMARK_MYOGENESIS%MSIGDB_C2%HALLMARK_MYOGENESIS	0.26226586	0.91282445	138	0.94463766	8020
RIBONUCLEOSIDE BISPHOSPHATE BIOSYNTHETIC PROCESS%GOBP%GO:0034030	0.2775363	0.9045232	50	0.94464284	8642
CRISTAE FORMATION%REACTOME%R-HSA-8949613.2	0.29042804	0.9036253	31	0.9446766	6851
REGULATION OF ENDOCYTOSIS%GOBP%GO:0030100	0.25473067	0.9029634	184	0.9446788	7267
CHONDROITIN SULFATE PROTEOGLYCAN METABOLIC PROCESS%GOBP%GO:0050654	0.28985563	0.90428084	34	0.9446811	10191
REGULATION OF TOR SIGNALING%GOBP%GO:0032006	0.26836497	0.91291344	86	0.94468427	8759
TRANSLATION OF STRUCTURAL PROTEINS%REACTOME DATABASE ID RELEASE 75%9694635	0.28504094	0.91191125	44	0.9447196	11479
FGF SIGNALING PATHWAY%PANTHER PATHWAY%P00021	0.26603594	0.91201663	110	0.9447295	5640
EBOLA VIRUS PATHWAY ON HOST%WIKIPATHWAYS_20210110%WP4217%HOMO SAPIENS	0.42217124	1.4518048	98	0.94476557	7004
DICARBOXYLIC ACID METABOLIC PROCESS%GOBP%GO:0043648	0.2720848	0.9129827	66	0.94477445	4989
PID_TOLL_ENDOGENOUS_PATHWAY%MSIGDB_C2%PID_TOLL_ENDOGENOUS_PATHWAY	0.31368116	0.9031334	19	0.9447924	6158
G0 AND EARLY G1%REACTOME DATABASE ID RELEASE 75%1538133	0.3016578	0.9036705	27	0.9448196	3764
POSITIVE REGULATION OF CYTOKINE-MEDIATED SIGNALING PATHWAY%GOBP%GO:0001961	0.2891895	0.9043299	31	0.94482577	6935
REGULATION OF VASCULAR ENDOTHELIAL CELL PROLIFERATION%GOBP%GO:1905562	0.32092515	0.9125196	17	0.94483	13230
POSITIVE REGULATION OF ATP METABOLIC PROCESS%GOBP%GO:1903580	0.29064322	0.9038933	34	0.94483	9593
PID_KIT_PATHWAY%MSIGDB_C2%PID_KIT_PATHWAY	0.28196138	0.9034493	41	0.9448301	9509
POSITIVE REGULATION OF VASCULAR SMOOTH MUSCLE CELL PROLIFERATION%GOBP%GO:1904707	0.31955215	0.9037749	19	0.9448353	12638
ATM SIGNALING PATHWAY%WIKIPATHWAYS_20210110%WP2516%HOMO SAPIENS	0.29452556	0.9032117	32	0.9448581	13962
REGULATION OF TP53 ACTIVITY THROUGH METHYLATION%REACTOME DATABASE ID RELEASE 75%6804760	0.317038	0.91239136	19	0.944859	8274
PAUSING AND RECOVERY OF HIV ELONGATION%REACTOME%R-HSA-167290.2	0.29283142	0.90408427	32	0.9448789	7291
REGULATION OF ERK1 AND ERK2 CASCADE%GOBP%GO:0070372	0.25328422	0.90452456	186	0.94488394	9779
POSITIVE REGULATION OF LIPID TRANSPORT%GOBP%GO:0032370	0.28409046	0.9039504	44	0.94494855	7905
URETERIC BUD MORPHOGENESIS%GOBP%GO:0060675	0.31918254	0.91301304	20	0.94495076	7674
ORGANIC ACID CATABOLIC PROCESS%GOBP%GO:0016054	0.25585324	0.91256434	205	0.94498384	7296
REGULATION OF COFACTOR METABOLIC PROCESS%GOBP%GO:0051193	0.26186076	0.9045732	94	0.9450225	6322
TRANSLATION OF STRUCTURAL PROTEINS%REACTOME DATABASE ID RELEASE 75%9683701	0.2960736	0.9025846	28	0.94504285	2983
GLUTATHIONE DERIVATIVE METABOLIC PROCESS%GOBP%GO:1901685	0.31547186	0.9026715	19	0.94509715	5007
IKK COMPLEX RECRUITMENT MEDIATED BY RIP1%REACTOME DATABASE ID RELEASE 75%937041	0.31425586	0.91303176	22	0.9451539	4048
DNA DOUBLE-STRAND BREAK REPAIR%REACTOME%R-HSA-5693532.3	0.25948498	0.9090873	139	0.94515437	11755
SKELETAL MUSCLE CONTRACTION%GOBP%GO:0003009	0.32689857	0.90930307	17	0.9451761	7419
IL6%NETPATH%IL6	0.27472767	0.9132191	64	0.94521534	4701
PI3 KINASE PATHWAY%PANTHER PATHWAY%P00048	0.2847331	0.9045952	41	0.9452177	4268
NUCLEOSIDE MONOPHOSPHATE BIOSYNTHETIC PROCESS%GOBP%GO:0009124	0.26499298	0.9023945	80	0.94521916	10263
CARDIAC PROGENITOR DIFFERENTIATION%WIKIPATHWAYS_20210110%WP2406%HOMO SAPIENS	0.28289428	0.90687406	41	0.9452486	11635
EPHRIN SIGNALING%REACTOME DATABASE ID RELEASE 75%3928664	0.32272503	0.91330904	18	0.94525653	11619
PID_DELTA_NP63_PATHWAY%MSIGDB_C2%PID_DELTA_NP63_PATHWAY	0.287766	0.9069707	36	0.9452755	9661
FOREBRAIN CELL MIGRATION%GOBP%GO:0021885	0.30649078	0.90970284	23	0.94530094	2097
POSITIVE REGULATION OF PROTEIN TYROSINE KINASE ACTIVITY%GOBP%GO:0061098	0.2937749	0.9130722	33	0.9453078	9489
POSITIVE REGULATION OF TELOMERE CAPPING%GOBP%GO:1904355	0.3165402	0.90934694	17	0.9453259	12603
REGULATION OF OLIGODENDROCYTE DIFFERENTIATION%GOBP%GO:0048713	0.31897098	0.90957	17	0.94533896	9248
CELL CYCLE ARREST%GOBP%GO:0007050	0.26123175	0.90703773	111	0.9453692	8329
POSITIVE REGULATION OF VIRAL GENOME REPLICATION%GOBP%GO:0045070	0.46587065	1.4620237	34	0.9453777	5766
PURINE NUCLEOSIDE BISPHOSPHATE BIOSYNTHETIC PROCESS%GOBP%GO:0034033	0.2775363	0.9090913	50	0.9453933	8642
SIGNALING BY NUCLEAR RECEPTORS%REACTOME%R-HSA-9006931.5	0.25613314	0.9106326	232	0.9453962	9790
CELL CYCLE DNA REPLICATION%GOBP%GO:0044786	0.28385767	0.9046203	42	0.9454039	11716
REGULATION OF HSF1-MEDIATED HEAT SHOCK RESPONSE%REACTOME%R-HSA-3371453.1	0.26821256	0.9074589	66	0.94540465	5617
ENDOTHELIAL CELL MIGRATION%GOBP%GO:0043542	0.27722302	0.9075638	49	0.9454155	13653
REGULATION OF SMOOTHENED SIGNALING PATHWAY%GOBP%GO:0008589	0.4248569	1.401261	53	0.94541585	2461
TCR SIGNALING IN NAIVE CD8+ T CELLS%PATHWAY INTERACTION DATABASE NCI-NATURE CURATED DATA%TCR SIGNALING IN NAIVE CD8+ T CELLS	0.43702665	1.3987396	43	0.9454347	5108
MITOCHONDRIAL TRANSMEMBRANE TRANSPORT%GOBP%GO:1990542	0.26297644	0.9073259	101	0.94544965	12034
EPO SIGNALING PATHWAY%PATHWAY INTERACTION DATABASE NCI-NATURE CURATED DATA%EPO SIGNALING PATHWAY	0.29456595	0.90974027	31	0.94547415	3310
BIOCARTA_GH_PATHWAY%MSIGDB_C2%BIOCARTA_GH_PATHWAY	0.31295988	0.9076467	20	0.9454797	12347
POSITIVE REGULATION OF DOUBLE-STRAND BREAK REPAIR VIA NONHOMOLOGOUS END JOINING%GOBP%GO:2001034	0.32394874	0.9066248	16	0.9455467	7575
GLYOXYLATE METABOLISM AND GLYCINE DEGRADATION%REACTOME%R-HSA-389661.6	0.30238277	0.90935725	26	0.94555	6149
SENESCENCE AND AUTOPHAGY IN CANCER%WIKIPATHWAYS_20210110%WP615%HOMO SAPIENS	0.26911107	0.91022027	81	0.9455912	8404
PID_SYNDECAN_1_PATHWAY%MSIGDB_C2%PID_SYNDECAN_1_PATHWAY	0.28749138	0.90704155	35	0.94560236	11154
CHROMATIN ORGANIZATION%REACTOME DATABASE ID RELEASE 75%4839726	0.2534556	0.90592784	222	0.9456303	6287
PROTEASOMAL UBIQUITIN-INDEPENDENT PROTEIN CATABOLIC PROCESS%GOBP%GO:0010499	0.31160155	0.90768796	20	0.945636	13413
ICOSANOID METABOLIC PROCESS%GOBP%GO:0006690	0.26932865	0.9046223	71	0.94564426	5007
VACUOLAR ACIDIFICATION%GOBP%GO:0007035	0.3130554	0.9057993	21	0.9456786	11205
PROLACTIN SIGNALING PATHWAY%WIKIPATHWAYS_20210110%WP2037%HOMO SAPIENS	0.27538884	0.90975	64	0.9456993	10831
INSULIN RECEPTOR SIGNALING PATHWAY%GOBP%GO:0008286	0.26811314	0.90599686	71	0.945723	8331
MUSCLE TISSUE DEVELOPMENT%GOBP%GO:0060537	0.25892428	0.90469	122	0.94573915	7750
TRANSLATION INITIATION COMPLEX FORMATION%REACTOME%R-HSA-72649.3	0.2732104	0.9020353	58	0.94575995	5978
POSITIVE REGULATION OF ERK1 AND ERK2 CASCADE%GOBP%GO:0070374	0.25891724	0.90531933	123	0.94576037	8543
SIGNALING BY TYPE 1 INSULIN-LIKE GROWTH FACTOR 1 RECEPTOR (IGF1R)%REACTOME DATABASE ID RELEASE 75%2404192	0.28845564	0.90981805	35	0.9457828	13549
DNA IR-DAMAGE AND CELLULAR RESPONSE VIA ATR%WIKIPATHWAYS_20210110%WP4016%HOMO SAPIENS	0.27186146	0.90168196	64	0.94580203	12726
RAB GERANYLGERANYLATION%REACTOME%R-HSA-8873719.2	0.27628115	0.9077218	62	0.94580555	11797
MYELOID LEUKOCYTE DIFFERENTIATION%GOBP%GO:0002573	0.2766155	0.91022664	53	0.94582725	14063
DIVALENT METAL ION TRANSPORT%GOBP%GO:0070838	0.25681618	0.90704525	171	0.9458416	3251
PROSTAGLANDIN BIOSYNTHETIC PROCESS%GOBP%GO:0001516	0.31999093	0.9053836	18	0.9458633	4638
POSITIVE REGULATION OF CANONICAL WNT SIGNALING PATHWAY%GOBP%GO:0090263	0.2598385	0.9018559	123	0.9458899	8708
PID_ERBB1_RECEPTOR_PROXIMAL_PATHWAY%MSIGDB_C2%PID_ERBB1_RECEPTOR_PROXIMAL_PATHWAY	0.2980963	0.9078991	29	0.94589293	12347
BIOLOGICAL OXIDATIONS%REACTOME%R-HSA-211859.2	0.25813746	0.9082157	144	0.945904	5673
EXTRINSIC APOPTOTIC SIGNALING PATHWAY%GOBP%GO:0097191	0.27044132	0.90601563	68	0.9459228	9589
INTRACELLULAR PH REDUCTION%GOBP%GO:0051452	0.2818131	0.9047063	41	0.9459476	8331
CELL GROWTH%GOBP%GO:0016049	0.2851794	0.9083024	45	0.94595534	2926
ENKEPHALIN RELEASE%PANTHER PATHWAY%P05913	0.29774106	0.9098321	31	0.9459999	9314
RETINOBLASTOMA GENE IN CANCER%WIKIPATHWAYS_20210110%WP2446%HOMO SAPIENS	0.27111	0.9016963	65	0.94600743	5972
REGULATION OF DENDRITIC SPINE DEVELOPMENT%GOBP%GO:0060998	0.28081244	0.9102541	45	0.94601375	11421
POSITIVE REGULATION OF EMBRYONIC DEVELOPMENT%GOBP%GO:0040019	0.43961594	1.3973967	35	0.946021	6289
POSITIVE REGULATION OF HISTONE MODIFICATION%GOBP%GO:0031058	0.26982817	0.9011192	64	0.9460224	5917
NEUROTRANSMITTER RECEPTORS AND POSTSYNAPTIC SIGNAL TRANSMISSION%REACTOME DATABASE ID RELEASE 75%112314	0.25742248	0.90498847	165	0.94602394	6719
ACTIVATION OF CYSTEINE-TYPE ENDOPEPTIDASE ACTIVITY INVOLVED IN APOPTOTIC PROCESS%GOBP%GO:0006919	0.27076498	0.90773004	73	0.94603306	5948
CALCIUM-MEDIATED SIGNALING%GOBP%GO:0019722	0.40785286	1.3845806	84	0.94603634	8401
NEGATIVE REGULATION OF FGFR1 SIGNALING%REACTOME%R-HSA-5654726.2	0.31149194	0.90793216	21	0.9460597	8274
POSITIVE REGULATION OF BLOOD CIRCULATION%GOBP%GO:1903524	0.45990896	1.4504461	33	0.94606847	7588
HOMOLOGY DIRECTED REPAIR%REACTOME%R-HSA-5693538.2	0.26285082	0.90835947	110	0.94607204	12221
POSITIVE REGULATION OF WNT SIGNALING PATHWAY%GOBP%GO:0030177	0.2583834	0.9080285	148	0.9460825	8708
ESTROGEN METABOLIC PROCESS%GOBP%GO:0008210	0.3185482	0.90990376	18	0.9460838	4928
MICROTUBULE POLYMERIZATION OR DEPOLYMERIZATION%GOBP%GO:0031109	0.27953047	0.9053946	45	0.94608635	13794
CELLULAR RESPONSE TO FIBROBLAST GROWTH FACTOR STIMULUS%GOBP%GO:0044344	0.26697543	0.90506005	81	0.94609785	7470
INORGANIC ANION TRANSPORT%GOBP%GO:0015698	0.26924822	0.90626395	83	0.94611114	8506
REGULATION OF JAK-STAT CASCADE%GOBP%GO:0046425	0.26729596	0.9060407	71	0.9461132	3153
REGULATION OF PROTEIN TARGETING TO MEMBRANE%GOBP%GO:0090313	0.29423743	0.9009601	26	0.94612676	12795

ENDOGENOUS TLR SIGNALING%PATHWAY INTERACTION DATABASE NCI-NATURE CURATED DATA%ENDOGENOUS TLR SIGNALING	0.30114302	0.904729	23	0.9461419	12415
BLOOD COAGULATION, FIBRIN CLOT FORMATION%GOBP%GO:0072378	0.51231587	1.4075917	15	0.9461558	1349
CA-DEPENDENT EVENTS%REACTOME%R-HSA-111996.1	0.28680402	0.90841824	36	0.94618714	4909
SIGNAL TRANSDUCTION INVOLVED IN MITOTIC CELL CYCLE CHECKPOINT%GOBP%GO:0072413	0.41774315	1.3853474	58	0.9462008	6040
CHROMATIN MODIFYING ENZYMES%REACTOME%R-HSA-3247509.4	0.2534556	0.9048023	222	0.94621223	6287
REGULATION OF ATP BIOSYNTHETIC PROCESS%GOBP%GO:2001169	0.26702964	0.9011202	80	0.9462636	8514
TRNA PROCESSING%GOBP%GO:0008033	0.26009208	0.9054044	107	0.9463088	7650
PURINE NUCLEOSIDE MONOPHOSPHATE BIOSYNTHETIC PROCESS%GOBP%GO:0009127	0.26515245	0.89683664	74	0.9463093	7952
TGF_BETA_RECEPTOR%NETPATH%TGF_BETA_RECEPTOR	0.2549937	0.9060479	172	0.9463418	10594
PID_ENDOTHELIN_PATHWAY%MSIGDB_C2%PID_ENDOTHELIN_PATHWAY	0.27946737	0.9011937	45	0.9463551	10474
NETWORK	0.46822286	1.4750469	37	0.9463968	2710
BIOCARTA_WNT_PATHWAY%MSIGDB_C2%BIOCARTA_WNT_PATHWAY	0.5142346	1.4867501	19	0.9464054	2966
NOREPINEPHRINE NEUROTRANSMITTER RELEASE CYCLE%REACTOME%R-HSA-181430.3	0.32987857	0.9084323	16	0.946407	9035
RNA POLYMERASE III TRANSCRIPTION INITIATION FROM TYPE 3 PROMOTER%REACTOME%R-HSA-76071.2	0.2970668	0.9012668	28	0.9464493	8398
PROTEIN K48-LINKED DEUBIQUITINATION%GOBP%GO:0071108	0.3048831	0.89684194	22	0.9465387	1890
POSITIVE REGULATION OF B CELL PROLIFERATION%GOBP%GO:0030890	0.49639753	1.4070731	19	0.9466415	8501
PID_HNF3A_PATHWAY%MSIGDB_C2%PID_HNF3A_PATHWAY	0.51026475	1.5482107	29	0.9466604	2710
NEGATIVE REGULATION OF PROTEIN COMPLEX ASSEMBLY%GOBP%GO:0031333	0.4110435	1.398987	97	0.9466805	5396
RHO GTPASES ACTIVATE PAKS%REACTOME DATABASE ID RELEASE 75%5627123	0.30969036	0.89632726	20	0.94669235	1593
NEGATIVE REGULATION OF NEURON PROJECTION DEVELOPMENT%GOBP%GO:0010977	0.26526606	0.8968751	88	0.9467112	8411
POSITIVE REGULATION OF SODIUM ION TRANSPORT%GOBP%GO:0010765	0.29031837	0.89652795	28	0.9467378	5911
SYNTHESIS OF PIPS AT THE EARLY ENDOSOME MEMBRANE%REACTOME%R-HSA-1660516.7	0.3196797	0.8969439	16	0.94680524	5287
PRE-NOTCH PROCESSING IN GOLGI%REACTOME DATABASE ID RELEASE 75%1912420	0.51590604	1.4533418	18	0.9468223	4976
POSITIVE REGULATION OF POTASSIUM ION TRANSMEMBRANE TRANSPORT%GOBP%GO:1901381	0.47716877	1.4563347	28	0.9468548	4165
ENVELOPE PROTEINS AND THEIR POTENTIAL ROLES IN EDMD PHYSIOPATHOLOGY%WIKIPATHWAYS_20210110%WP4535%HOMO SAPIENS	0.29600903	0.89633584	29	0.9469134	8299
REGULATION OF DNA REPAIR%GOBP%GO:0006282	0.25972626	0.9002558	125	0.94692856	8413
REGULATION OF KETONE BIOSYNTHETIC PROCESS%GOBP%GO:0010566	0.32866585	0.897	15	0.94693	13836
NEGATIVE REGULATION OF CYCLIN-DEPENDENT PROTEIN KINASE ACTIVITY%GOBP%GO:1904030	0.31057355	0.9004747	19	0.9469396	8404
PEPTIDYL-LYSINE METHYLATION%GOBP%GO:0018022	0.27264977	0.9003549	58	0.9469525	6287
MULTIVESICULAR BODY SORTING PATHWAY%GOBP%GO:0071985	0.4638453	1.4539158	36	0.9469579	4988
RESPONSE TO TRANSFORMING GROWTH FACTOR BETA%GOBP%GO:0071559	0.25710958	0.89708173	115	0.9469887	4704
REGULATION OF ATP METABOLIC PROCESS%GOBP%GO:1903578	0.2638114	0.90009916	102	0.9470054	8514
PTEN REGULATION%REACTOME%R-HSA-6807070.2	0.25660574	0.8960622	134	0.9470218	8874
REGULATION OF RELEASE OF CYTOCHROME C FROM MITOCHONDRIA%GOBP%GO:0090199	0.43793282	1.3903456	39	0.94710696	2885
BONE DEVELOPMENT%GOBP%GO:0060348	0.4303642	1.4569086	74	0.94712096	6311
POSITIVE REGULATION OF TRANSCRIPTION OF NOTCH RECEPTOR TARGET%GOBP%GO:0007221	0.6038774	1.674983	16	0.9471228	1090
MITOCHONDRIAL ATP SYNTHESIS COUPLED ELECTRON TRANSPORT%GOBP%GO:0042775	0.4036243	1.3867962	81	0.9471232	4191
REELIN SIGNALING PATHWAY%PATHWAY INTERACTION DATABASE NCI-NATURE CURATED DATA%REELIN SIGNALING PATHWAY	0.29058275	0.8970895	29	0.94721216	5700
SIGNALING BY FLT3 FUSION PROTEINS%REACTOME DATABASE ID RELEASE 75%9703465	0.32339057	0.8997782	17	0.9472146	8113
MRNA TRANSPORT%GOBP%GO:0051028	0.25918248	0.89324796	107	0.9472332	5209
BIOCARTA_CXCR4_PATHWAY%MSIGDB_C2%BIOCARTA_CXCR4_PATHWAY	0.32209304	0.89718276	16	0.94724035	10800
HALLMARK_PROTEIN_SECRETION%MSIGDB_C2%HALLMARK_PROTEIN_SECRETION	0.2657621	0.8998742	78	0.94724077	9586
SYNAPTIC VESICLE RECYCLING%GOBP%GO:0036465	0.2772435	0.8976225	44	0.94724125	5759
HALLMARK_EPITHELIAL_MESENCHYMAL_TRANSITION%MSIGDB_C2%HALLMARK_EPITHELIAL_MESENCHYMAL_TRANSITION	0.2560751	0.8979579	145	0.94724643	11890
CELLULAR COMPONENT ASSEMBLY INVOLVED IN MORPHOGENESIS%GOBP%GO:0010927	0.27215514	0.8929952	53	0.9472863	4028
CELLULAR RESPONSE TO RETINOIC ACID%GOBP%GO:0071300	0.28527218	0.89558935	35	0.9473034	6955
RIBOSOMAL SCANNING AND START CODON RECOGNITION%REACTOME%R-HSA-72702.3	0.27345622	0.897806	58	0.94733155	5978
NUCLEIC ACID TRANSPORT%GOBP%GO:0050657	0.25638548	0.8980165	142	0.9473627	5209
GLYCOGEN SYNTHESIS AND DEGRADATION%WIKIPATHWAYS_20210110%WP500%HOMO SAPIENS	0.29163104	0.89856803	29	0.9474026	8534
POSITIVE REGULATION OF PROTEIN SERINE/THREONINE KINASE ACTIVITY%GOBP%GO:0071902	0.24966489	0.89743227	223	0.94741	12555
IRS-RELATED EVENTS TRIGGERED BY IGF1R%REACTOME%R-HSA-2428928.1	0.28439772	0.8986772	34	0.9474133	13549
TRANSLATION INHIBITORS IN CHRONICALLY ACTIVATED PDGFRA CELLS%WIKIPATHWAYS_20210110%WP4566%HOMO SAPIENS	0.2847507	0.893047	35	0.94741464	10539
ESTROGEN-DEPENDENT NUCLEAR EVENTS DOWNSTREAM OF ESR-MEMBRANE SIGNALING%REACTOME DATABASE ID RELEASE 75%9634638	0.30969724	0.89878327	22	0.9474327	9736
REGULATION OF ENDOCRINE PROCESS%GOBP%GO:0044060	0.3007149	0.89719987	24	0.9474421	6752
AEROBIC RESPIRATION%GOBP%GO:0009060	0.2682868	0.89574826	65	0.94745284	6512
ACTIVATION OF PROTEIN KINASE ACTIVITY%GOBP%GO:0032147	0.24778649	0.8932536	223	0.947459	8109
CARGO TRAFFICKING TO THE PERICILIARY MEMBRANE%REACTOME%R-HSA-5620920.3	0.27522412	0.8976328	48	0.9474643	8908
REGULATION OF SMOOTH MUSCLE CELL DIFFERENTIATION%GOBP%GO:0051150	0.3126452	0.89562887	18	0.94746834	12638
MIRNA METABOLIC PROCESS%GOBP%GO:0010586	0.3074602	0.8994319	20	0.94747615	7405
HISTONE LYSINE METHYLATION%GOBP%GO:0034968	0.27841887	0.8988636	44	0.947492	6287
CARDIAC CHAMBER DEVELOPMENT%GOBP%GO:0003205	0.26525083	0.8981597	85	0.94754565	7849
PEPTIDYL-SERINE PHOSPHORYLATION%GOBP%GO:0018105	0.25161567	0.8980384	154	0.9475567	7013
PID_HEDGEHOG_GLI_PATHWAY%MSIGDB_C2%PID_HEDGEHOG_GLI_PATHWAY	0.284247	0.89949054	36	0.94759655	2385
INTRA-GOLGI AND RETROGRADE GOLGI-TO-ER TRAFFIC%REACTOME%R-HSA-6811442.1	0.25125772	0.8992583	179	0.9476021	9610
CILIOPATHIES%WIKIPATHWAYS_20210110%WP4803%HOMO SAPIENS	0.25897568	0.89834213	137	0.94763017	9051
UNSATURATED FATTY ACID BIOSYNTHETIC PROCESS%GOBP%GO:0006636	0.28670672	0.89328307	33	0.9476343	5007
PROTEIN INSERTION INTO MEMBRANE%GOBP%GO:0051205	0.28514934	0.89721775	35	0.9476411	13166
VIRAL GENOME REPLICATION%GOBP%GO:0019079	0.49313486	1.4508445	21	0.94766253	3670
CELL FATE SPECIFICATION%GOBP%GO:0001708	0.28825	0.89888126	36	0.9476949	9065
PHOSPHATIDYLCHOLINE ACYL-CHAIN REMODELING%GOBP%GO:0036151	0.3073838	0.89819974	22	0.9477057	14870
POSITIVE REGULATION OF JUN KINASE ACTIVITY%GOBP%GO:0043507	0.2713047	0.8950487	47	0.9477177	4638
PHOSPHORYLATION OF CD3 AND TCR ZETA CHAINS%REACTOME DATABASE ID RELEASE 75%202427	0.5046382	1.3855203	15	0.94773287	8142
NEGATIVE REGULATION OF ANTIGEN RECEPTOR-MEDIATED SIGNALING PATHWAY%GOBP%GO:0050858	0.53212374	1.4520053	15	0.9477365	6547
ENTRY INTO CELL OF OTHER ORGANISM INVOLVED IN SYMBIOTIC INTERACTION%GOBP%GO:0051806	0.4494028	1.3906573	36	0.9477796	6947
NEUROTROPHIN TRK RECEPTOR SIGNALING PATHWAY%GOBP%GO:0048011	0.54414403	1.4661605	15	0.9477924	4944
TRANSFORMING GROWTH FACTOR BETA RECEPTOR SIGNALING PATHWAY%GOBP%GO:0007179	0.2618167	0.89331746	85	0.9477988	4586
RESOLUTION OF ABASIC SITES (AP SITES)%REACTOME%R-HSA-73933.2	0.28635758	0.8951018	36	0.9478488	1723
DETECTION OF STIMULUS%GOBP%GO:0051606	0.25493452	0.8952004	131	0.94786996	13438
SELENIUM MICRONUTRIENT NETWORK%WIKIPATHWAYS_20210110%WP15%HOMO SAPIENS	0.27636367	0.8989016	52	0.947889	10796
PEPTIDE GPCRS%WIKIPATHWAYS_20210110%WP24%HOMO SAPIENS	0.4687233	1.4543945	33	0.9478947	1974
POSITIVE REGULATION OF NITRIC OXIDE METABOLIC PROCESS%GOBP%GO:1904407	0.31512982	0.89336467	19	0.94795275	9303
HS-GAG DEGRADATION%REACTOME%R-HSA-2024096.1	0.30657384	0.89469934	21	0.9479805	4930
NEGATIVE REGULATION OF POTASSIUM ION TRANSPORT%GOBP%GO:0043267	0.5266311	1.4734873	18	0.9480224	4230
ASTROCYTE DEVELOPMENT%GOBP%GO:0014002	0.3279786	0.89893544	15	0.9480586	1440
ANTIGEN RECEPTOR-MEDIATED SIGNALING PATHWAY%GOBP%GO:0050851	0.4178317	1.4623184	154	0.94810724	8250
SOMITE DEVELOPMENT%GOBP%GO:0061053	0.30692926	0.8934027	21	0.94811	7112
REGULATION OF B CELL PROLIFERATION%GOBP%GO:0030888	0.47030702	1.449562	26	0.9481627	8501
CELL DIFFERENTIATION - INDEX EXPANDED%WIKIPATHWAYS_20210110%WP2023%HOMO SAPIENS	0.3247151	0.89472395	16	0.948167	10900
CRANIAL SKELETAL SYSTEM DEVELOPMENT%GOBP%GO:1904888	0.30417904	0.89246994	23	0.9481753	6191
APOPTOTIC EXECUTION PHASE%REACTOME DATABASE ID RELEASE 75%75153	0.43504944	1.4013032	45	0.9481786	4570
MITOCHONDRIAL BIOGENESIS%REACTOME%R-HSA-1592230.2	0.26295704	0.8923414	90	0.94820297	7464
VITAMIN A AND CAROTENOID METABOLISM%WIKIPATHWAYS_20210110%WP716%HOMO SAPIENS	0.28855	0.8942556	29	0.94821805	14483
MITOCHONDRIAL TRANSLATION ELONGATION%REACTOME%R-HSA-5389840.1	0.26084182	0.8941237	88	0.9482588	6216
REGULATION OF NUCLEOTIDE BIOSYNTHETIC PROCESS%GOBP%GO:0030808	0.2579504	0.8910413	92	0.94827473	6322
POSITIVE REGULATION OF FIBROBLAST PROLIFERATION%GOBP%GO:0048146	0.29872325	0.89433295	27	0.94828606	10831
MAP3K1 ROLE IN PROMOTING AND BLOCKING GONADAL DETERMINATION%WIKIPATHWAYS_20210110%WP4872%HOMO SAPIENS	0.52291954	1.4243945	15	0.94830155	7542
'DE NOVO' PROTEIN FOLDING%GOBP%GO:0006458	0.28108153	0.8934133	35	0.9483241	11894
SKELETAL MUSCLE TISSUE DEVELOPMENT%GOBP%GO:0007519	0.28781626	0.8939697	33	0.9483469	929
POSITIVE REGULATION OF SMOOTH MUSCLE CELL PROLIFERATION%GOBP%GO:0048661	0.27605033	0.89348847	41	0.94840276	10945
REGULATION OF PURINE NUCLEOTIDE BIOSYNTHETIC PROCESS%GOBP%GO:1900371	0.2579504	0.8910911	92	0.94840884	6322
NUCLEOSIDE BISPHOSPHATE BIOSYNTHETIC PROCESS%GOBP%GO:0033866	0.2775363	0.893717	50	0.9484159	8642
PEPTIDE HORMONE SECRETION%GOBP%GO:0030072	0.28812286	0.8943829	29	0.94841605	10116
CHAPERONE-MEDIATED PROTEIN FOLDING%GOBP%GO:0061077	0.2744863	0.8916067	49	0.94853306	2491
POSITIVE REGULATION OF RESPONSE TO DNA DAMAGE STIMULUS%GOBP%GO:2001022	0.25690833	0.8937495	103	0.9485797	6301
AMINO SUGAR METABOLIC PROCESS%GOBP%GO:0006040	0.28742546	0.89112204	33	0.9485849	7706
SURFACE INTERACTIONS	0.31446493	0.8935159	19	0.94859034	1860
CIRCADIAN RHYTHM%GOBP%GO:0007623	0.2620484	0.891652	90	0.94868153	7493
NEGATIVE REGULATION OF PROTEASOMAL PROTEIN CATABOLIC PROCESS%GOBP%GO:1901799	0.27639455	0.890687	48	0.9487788	6445
POSITIVE REGULATION OF NEUROTRANSMITTER TRANSPORT%GOBP%GO:0051590	0.31367585	0.89113176	19	0.94880515	15150
POSITIVE REGULATION OF IMMUNE EFFECTOR PROCESS%GOBP%GO:0002699	0.25598922	0.8916993	117	0.94882613	6810
REGULATION OF GLUTAMATE RECEPTOR SIGNALING PATHWAY%GOBP%GO:1900449	0.27226123	0.89181525	54	0.94882905	6682
PID_FOXO_PATHWAY%MSIGDB_C2%PID_FOXO_PATHWAY	0.28177902	0.8913358	37	0.94885164	9271
IGF1R SIGNALING CASCADE%REACTOME DATABASE ID RELEASE 75%2428924	0.28439772	0.89119476	34	0.9489188	13549
REGULATION OF CARDIAC MUSCLE CONTRACTION BY CALCIUM ION SIGNALING%GOBP%GO:0010882	0.3037574	0.8839025	20	0.9489505	14253
BINDING AND UPTAKE OF LIGANDS BY SCAVENGER RECEPTORS%REACTOME DATABASE ID RELEASE 75%2173782	0.28583032	0.8918695	32	0.94895494	4400
SIGNALING BY SCF-KIT%REACTOME DATABASE ID RELEASE 75%1433557	0.27964693	0.88392687	34	0.9491373	5320
NEGATIVE REGULATION OF STRESS-ACTIVATED MAPK CASCADE%GOBP%GO:0032873	0.27769998	0.8787687	39	0.9491962	12125
PID_HDAC_CLASSIII_PATHWAY%MSIGDB_C2%PID_HDAC_CLASSIII_PATHWAY	0.4761328	1.3990532	21	0.9492103	2049
ACTIVATED NOTCH1 TRANSMITS SIGNAL TO THE NUCLEUS%REACTOME%R-HSA-2122948.3	0.28368738	0.8865524	31	0.9492203	8274
PHOSPHOINOSITIDES METABOLISM%WIKIPATHWAYS_20210110%WP4971%HOMO SAPIENS	0.4653744	1.4871639	40	0.9492638	6047
RESPONSE TO ACETYLCHOLINE%GOBP%GO:1905144	0.46800548	1.4076084	24	0.9492699	5169
INTRACELLULAR LIPID TRANSPORT%GOBP%GO:0032365	0.28255406	0.8866379	33	0.9492807	8128
BY HDAC CLASS II	0.2836102	0.87966245	33	0.94928217	9614
210744.2	0.67115146	1.8332077	15	0.9492867	1090
PID_VEGFR1_2_PATHWAY%MSIGDB_C2%PID_VEGFR1_2_PATHWAY	0.27052683	0.883959	49	0.9493029	4523
ANATOMICAL STRUCTURE HOMEOSTASIS%GOBP%GO:0060249	0.24879707	0.8903066	198	0.94930357	8335
BETA-CATENIN-TCF COMPLEX ASSEMBLY%GOBP%GO:1904837	0.51048654	1.5467119	28	0.9493055	4464

NEGATIVE REGULATION OF MAPK PATHWAY%REACTOME DATABASE ID RELEASE 75%5675221	0.27604875	0.878787	41	0.9493948	4896
O-LINKED GLYCOSYLATION%REACTOME%R-HSA-5173105.4	0.2592421	0.88355446	97	0.94942087	7593
MRNA CIS SPLICING, VIA SPLICEOSOME%GOBP%GO:0045292	0.29363266	0.8901355	28	0.949423	3825
MAP3K8 (TPL2)-DEPENDENT MAPK1 3 ACTIVATION%REACTOME%R-HSA-5684264.2	0.605819	1.6710134	16	0.9494328	4048
REGULATION OF OSTEOCLAST DIFFERENTIATION%GOBP%GO:0045670	0.29158202	0.88493615	26	0.9494503	11888
DOPAMINERGIC NEUROGENESIS%WIKIPATHWAYS_20210110%WP2855%HOMO SAPIENS	0.49616387	1.4572253	24	0.94946635	2385
75%8936459	0.27130356	0.87898135	52	0.94947356	5509
ALPHA-AMINO ACID BIOSYNTHETIC PROCESS%GOBP%GO:1901607	0.27267793	0.88665795	52	0.9494788	6420
REGULATION OF CYCLIN-DEPENDENT PROTEIN KINASE ACTIVITY%GOBP%GO:1904029	0.2607344	0.88409394	83	0.94950306	9937
PEPTIDYL-GLUTAMIC ACID MODIFICATION%GOBP%GO:0018200	0.29123935	0.8796633	25	0.9495146	8813
RNA POLYMERASE III TRANSCRIPTION%REACTOME DATABASE ID RELEASE 75%74158	0.27560863	0.8839609	41	0.94953597	8398
NECROTIC CELL DEATH%GOBP%GO:0070265	0.29579094	0.86989003	21	0.9495433	926
EPH-EPHRIN SIGNALING%REACTOME DATABASE ID RELEASE 75%2682334	0.25881922	0.88244057	88	0.9495531	6134
PID_INSULIN_GLUCOSE_PATHWAY%MSIGDB_C2%PID_INSULIN_GLUCOSE_PATHWAY	0.31934866	0.886271	17	0.9495668	8913
POSITIVE REGULATION OF DNA REPAIR%GOBP%GO:0045739	0.2638297	0.88605136	73	0.9495715	6301
INTERFERON ALPHA BETA SIGNALING%REACTOME DATABASE ID RELEASE 75%909733	0.26692736	0.878815	53	0.9495761	10565
SODIUM ION TRANSPORT%GOBP%GO:0006814	0.26031065	0.88498396	81	0.94959056	10146
SIGNAL TRANSDUCTION IN RESPONSE TO DNA DAMAGE%GOBP%GO:0042770	0.41115156	1.3994123	95	0.9495932	6189
HISTONE EXCHANGE%GOBP%GO:0043486	0.27603668	0.882181	40	0.9496003	8364
NUCLEOTIDE-EXCISION REPAIR, DNA INCISION%GOBP%GO:0033683	0.27860808	0.8790284	39	0.9496103	11736
POSITIVE REGULATION OF MITOCHONDRIAL MEMBRANE PERMEABILITY INVOLVED IN APOPTOTIC PROCESS%GOBP%GO:1902110	0.32325348	0.88671213	15	0.9496105	9027
RNA POLYMERASE III TRANSCRIPTION TERMINATION%REACTOME%R-HSA-73980.3	0.29802582	0.882047	23	0.94961864	9135
LEUKOCYTE HOMEOSTASIS%GOBP%GO:0001776	0.31044802	0.88252366	20	0.94961905	9855
SIGNALING BY VEGF%REACTOME%R-HSA-194138.2	0.2574	0.8833337	95	0.9496487	6350
REGULATION OF EPIDERMAL CELL DIFFERENTIATION%GOBP%GO:0045604	0.28088143	0.87970066	37	0.9496727	6684
CHLORIDE TRANSPORT%GOBP%GO:0006821	0.2636873	0.88226384	62	0.94967383	5821
HISTONE METHYLATION%GOBP%GO:0016571	0.2637691	0.8861116	62	0.9496791	7080
MUCOPOLYSACCHARIDE METABOLIC PROCESS%GOBP%GO:1903510	0.26372945	0.8897887	79	0.9496813	7296
AMYLOID-BETA CLEARANCE%GOBP%GO:0097242	0.30941743	0.88272774	19	0.9496835	10145
METABOLISM OF STEROIDS%REACTOME%R-HSA-8957322.2	0.25269702	0.86945117	125	0.94969696	5156
DEVELOPMENTAL GROWTH%GOBP%GO:0048589	0.24994116	0.86919844	114	0.94971186	7085
RNA TRANSPORT%GOBP%GO:0050658	0.25638548	0.88410556	142	0.9497176	5209
REGULATION OF EARLY ENDOSOME TO LATE ENDOSOME TRANSPORT%GOBP%GO:2000641	0.30182028	0.86954725	19	0.94973344	4209
POSITIVE REGULATION OF JNK CASCADE%GOBP%GO:0046330	0.25013024	0.8699075	98	0.94973725	5208
DEVELOPMENT OF PRIMARY SEXUAL CHARACTERISTICS%GOBP%GO:0045137	0.2643834	0.8781356	61	0.9497705	11443
CELLULAR RESPONSE TO PEPTIDE HORMONE STIMULUS%GOBP%GO:0071375	0.24856679	0.885009	171	0.9497764	5356
POST-GOLGI VESICLE-MEDIATED TRANSPORT%GOBP%GO:0006892	0.25632936	0.8790526	95	0.9497926	5948
SIGNALING BY TGF-BETA RECEPTOR COMPLEX%REACTOME DATABASE ID RELEASE 75%170834	0.26051286	0.88418055	71	0.9498089	5995
POSITIVE REGULATION OF EPITHELIAL TO MESENCHYMAL TRANSITION%GOBP%GO:0010718	0.2771161	0.88289636	42	0.94981354	9704
PROTEIN-PROTEIN INTERACTIONS AT SYNAPSES%REACTOME DATABASE ID RELEASE 75%6794362	0.25789946	0.8742668	85	0.94983244	11575
NEGATIVE REGULATION OF PROTEIN TYROSINE KINASE ACTIVITY%GOBP%GO:0061099	0.47820053	1.4014986	21	0.94983536	4988
GLYCOSAMINOGLYCAN METABOLISM%REACTOME DATABASE ID RELEASE 75%1630316	0.25344265	0.88253176	116	0.94983876	5207
AMINO ACID TRANSMEMBRANE TRANSPORT%GOBP%GO:0003333	0.27591154	0.8867181	44	0.94983923	6444
BEHAVIOR%GOBP%GO:0007610	0.23956311	0.85013175	184	0.9498475	7276
PROTEIN EXPORT FROM NUCLEUS%GOBP%GO:0006611	0.25407654	0.8898223	140	0.9498524	5437
SIGNALING BY NODAL%REACTOME DATABASE ID RELEASE 75%1181150	0.30808768	0.8829913	18	0.9498607	7849
REGULATION OF PURINE NUCLEOTIDE METABOLIC PROCESS%GOBP%GO:1900542	0.25307938	0.87914	115	0.94986266	6512
COBALAMIN (CBL, VITAMIN B12) TRANSPORT AND METABOLISM%REACTOME%R-HSA-196741.2	0.3123878	0.869235	16	0.94986516	125
AMINOGLYCAN CATABOLIC PROCESS%GOBP%GO:0006026	0.26628035	0.8700788	55	0.9498711	5207
MATURATION OF LSU-RRNA%GOBP%GO:0000470	0.29568887	0.8868116	27	0.9498868	8718
KIDNEY MORPHOGENESIS%GOBP%GO:0060993	0.27776912	0.8798281	36	0.9498869	7674
POSITIVE REGULATION OF RECEPTOR-MEDIATED ENDOCYTOSIS%GOBP%GO:0048260	0.28033122	0.8870437	41	0.9498878	7174
NUCLEAR DNA REPLICATION%GOBP%GO:0033260	0.27663484	0.8827414	41	0.94989115	11716
BMP SIGNALING PATHWAY%GOBP%GO:0030509	0.26712802	0.8888868	66	0.94989204	6318
VACUOLE ORGANIZATION%GOBP%GO:0007033	0.25492892	0.86994624	104	0.9498943	8796
BIOMINERAL TISSUE DEVELOPMENT%GOBP%GO:0031214	0.29447109	0.88424855	25	0.9498993	5793
REGULATION OF PLASMA LIPOPROTEIN PARTICLE LEVELS%GOBP%GO:0097006	0.2681316	0.8797016	55	0.9499065	8637
SIGNALLING TO ERKS%REACTOME DATABASE ID RELEASE 75%187687	0.27723268	0.8509856	31	0.9499069	13275
NEGATIVE REGULATION OF LYMPHOCYTE ACTIVATION%GOBP%GO:0051250	0.25395423	0.8507335	70	0.94990695	7575
NEGATIVE REGULATION OF STRIATED MUSCLE TISSUE DEVELOPMENT%GOBP%GO:0045843	0.30078426	0.87829703	21	0.94991183	2293
POSITIVE REGULATION OF CHROMOSOME ORGANIZATION%GOBP%GO:2001252	0.24127501	0.85035104	152	0.94991404	8968
NEURAL TUBE CLOSURE%GOBP%GO:0001843	0.2732563	0.8502211	34	0.94991565	13223
ATM SIGNALING NETWORK IN DEVELOPMENT AND DISEASE %WIKIPATHWAYS_20210110%WP3878%HOMO SAPIENS	0.26789907	0.8508539	37	0.94991606	14116
BASIGIN INTERACTIONS%REACTOME%R-HSA-210991.1	0.5054477	1.457751	20	0.9499197	1860
JAK-STAT CASCADE%GOBP%GO:0007259	0.2825675	0.86955965	29	0.94994	6812
RNA INTERFERENCE%GOBP%GO:0016246	0.31822753	0.87432534	16	0.9499471	8137
RESPONSE TO STEROL%GOBP%GO:0036314	0.29317546	0.8514544	20	0.9499516	881
PID_SYNDECAN_2_PATHWAY%MSIGDB_C2%PID_SYNDECAN_2_PATHWAY	0.304232	0.88897336	21	0.9499521	2049
TRANSCRIPTIONAL REGULATION BY RUNX2%REACTOME DATABASE ID RELEASE 75%8878166	0.2583048	0.8868929	111	0.9499533	9509
NUCLEAR EXPORT%GOBP%GO:0051168	0.25093907	0.8815252	148	0.94996524	5437
MONOVALENT INORGANIC CATION TRANSPORT%GOBP%GO:0015672	0.24543326	0.878152	212	0.94996864	7432
MTOR SIGNALING PATHWAY%PATHWAY INTERACTION DATABASE NCI-NATURE CURATED DATA%MTOR SIGNALING PATHWAY	0.25512013	0.8515736	66	0.9499695	2347
NEGATIVE REGULATION OF GROWTH%GOBP%GO:0045926	0.25138274	0.8850259	161	0.9499749	7546
CELLULAR RESPONSE TO UNFOLDED PROTEIN%GOBP%GO:0034620	0.24992219	0.8701428	117	0.9499785	5400
POSITIVE REGULATION OF CELL MIGRATION INVOLVED IN SPROUTING ANGIOGENESIS%GOBP%GO:0090050	0.3070246	0.85106826	16	0.9499806	7992
NEGATIVE REGULATION OF ENDOTHELIAL CELL MIGRATION%GOBP%GO:0010596	0.2774718	0.8895346	43	0.9499846	11891
SENSORY PERCEPTION OF MECHANICAL STIMULUS%GOBP%GO:0050954	0.26048273	0.8799961	78	0.9500123	8381
PROTEIN HOMOTETRAMERIZATION%GOBP%GO:0051289	0.27376974	0.8845415	44	0.95002043	4032
NEGATIVE REGULATION OF TYPE I INTERFERON PRODUCTION%GOBP%GO:0032480	0.27619016	0.8738006	38	0.95002794	9855
PHOTODYNAMIC THERAPY-INDUCED NF-KB SURVIVAL SIGNALING%WIKIPATHWAYS_20210110%WP3617%HOMO SAPIENS	0.2870705	0.85052365	24	0.9500535	11221
DEFENSE RESPONSE TO OTHER ORGANISM%GOBP%GO:0098542	0.2467559	0.8798602	179	0.95005536	6382
POSITIVE REGULATION OF CILIUM ASSEMBLY%GOBP%GO:0045724	0.30098385	0.85039294	18	0.9500601	8853
CAMKK2 PATHWAY%WIKIPATHWAYS_20210110%WP4874%HOMO SAPIENS	0.29057097	0.88135564	25	0.95007765	10110
AXON GUIDANCE%GOBP%GO:0007411	0.24733159	0.8791495	193	0.9500786	10433
POSITIVE REGULATION OF LEUKOCYTE MIGRATION%GOBP%GO:0002687	0.2596668	0.8830007	89	0.9500788	11417
NUCLEOBASE CATABOLISM%REACTOME DATABASE ID RELEASE 75%8956319	0.5147369	1.5503395	26	0.95008314	6212
NEGATIVE REGULATION OF G PROTEIN-COUPLED RECEPTOR SIGNALING PATHWAY%GOBP%GO:0045744	0.47889397	1.4633514	27	0.9500852	5565
POSITIVE REGULATION OF CHOLESTEROL TRANSPORT%GOBP%GO:0032376	0.30359775	0.8842665	24	0.9500982	11335
POSITIVE REGULATION OF STEROL TRANSPORT%GOBP%GO:0032373	0.30359775	0.88705736	24	0.95009935	11335
MICROTUBULE ANCHORING%GOBP%GO:0034453	0.3087466	0.8855447	20	0.9501125	9743
NEGATIVE REGULATION OF EPITHELIAL CELL MIGRATION%GOBP%GO:0010633	0.26653197	0.88156414	58	0.9501254	11891
CELLULAR RESPONSE TO STEROID HORMONE STIMULUS%GOBP%GO:0071383	0.26377502	0.87435156	65	0.9501262	8165
RELEASE 75%72662	0.26473096	0.8768711	59	0.95013714	5978
PID_SHP2_PATHWAY%MSIGDB_C2%PID_SHP2_PATHWAY	0.27557418	0.8889959	42	0.95014197	13414
REGULATION OF MRNA STABILITY BY PROTEINS THAT BIND AU-RICH ELEMENTS%REACTOME DATABASE ID RELEASE 75%450531	0.2609817	0.88517696	84	0.9501424	8913
NEURON PROJECTION GUIDANCE%GOBP%GO:0097485	0.24733159	0.87674063	193	0.9501474	10433
EMBRYONIC HEART TUBE DEVELOPMENT%GOBP%GO:0035050	0.2706013	0.8701728	43	0.95015377	8065
NEGATIVE REGULATION OF SODIUM ION TRANSPORT%GOBP%GO:0010766	0.32463777	0.873857	15	0.95016044	6080
ADENYLATE CYCLASE-INHIBITING G PROTEIN-COUPLED RECEPTOR SIGNALING PATHWAY%GOBP%GO:0007193	0.2616759	0.85108525	45	0.9501669	8186
NEGATIVE REGULATION OF PROTEIN LOCALIZATION TO NUCLEUS%GOBP%GO:1900181	0.30285475	0.85171384	17	0.9501711	13669
ESTABLISHMENT OF PROTEIN LOCALIZATION TO PEROXISOME%GOBP%GO:0072663	0.25405	0.8518392	63	0.95017207	7116
REGULATION OF CD4-POSITIVE, ALPHA-BETA T CELL DIFFERENTIATION%GOBP%GO:0043370	0.28976858	0.88504094	28	0.9501853	1524
INTERLEUKIN-37 SIGNALING%REACTOME DATABASE ID RELEASE 75%9008059	0.29416174	0.8515752	19	0.95018923	8841
FOLIC ACID-CONTAINING COMPOUND METABOLIC PROCESS%GOBP%GO:0006760	0.3015764	0.8739647	20	0.95019144	5968
PHENOL-CONTAINING COMPOUND BIOSYNTHETIC PROCESS%GOBP%GO:0046189	0.31814912	0.8892001	16	0.9501926	14868
ANION TRANSMEMBRANE TRANSPORT%GOBP%GO:0098656	0.25361785	0.8890805	143	0.95020187	10244
PID_AMB2_NEUTROPHILS_PATHWAY%MSIGDB_C2%PID_AMB2_NEUTROPHILS_PATHWAY	0.3032925	0.8800087	18	0.95022273	3503
NEGATIVE REGULATION OF STRESS-ACTIVATED PROTEIN KINASE SIGNALING CASCADE%GOBP%GO:0070303	0.27769998	0.88431835	39	0.9502286	12125
RNA POLYMERASE II TRANSCRIBES SNRNA GENES%REACTOME%R-HSA-6807505.2	0.25681996	0.8746642	74	0.9502395	5629
GROWTH%GOBP%GO:0040007	0.2523547	0.8774069	115	0.9502404	7831
POLY(A)+ MRNA EXPORT FROM NUCLEUS%GOBP%GO:0016973	0.32256263	0.88710755	15	0.9502407	2719
RESPONSE TO UNFOLDED PROTEIN%GOBP%GO:0006986	0.24322511	0.8521778	137	0.95024484	5617
OXIDATIVE STRESS INDUCED SENESCENCE%REACTOME%R-HSA-2559580.4	0.26017076	0.87776	80	0.9502615	9509
REACTIVE NITROGEN SPECIES METABOLIC PROCESS%GOBP%GO:2001057	0.31834477	0.88858527	16	0.9502708	5076
DNA REPLICATION%PANTHER PATHWAY%P00017	0.29608926	0.8511486	19	0.95027274	11637
REGULATION OF CYCLIN-DEPENDENT PROTEIN SERINE/THREONINE KINASE ACTIVITY%GOBP%GO:0000079	0.2592794	0.87691873	79	0.9502803	9937
CHEMICAL SYNAPTIC TRANSMISSION, POSTSYNAPTIC%GOBP%GO:0099565	0.2961469	0.8772708	23	0.9502822	9397
GAP JUNCTION TRAFFICKING AND REGULATION%REACTOME DATABASE ID RELEASE 75%157858	0.31497338	0.8747468	16	0.9503012	13178
REGULATION OF LIPOPOLYSACCHARIDE-MEDIATED SIGNALING PATHWAY%GOBP%GO:0031664	0.32386005	0.88159156	15	0.95030206	9855
REGULATION OF COAGULATION%GOBP%GO:0050818	0.42113343	1.3855515	52	0.95031065	3153
MAINTENANCE OF PROTEIN LOCALIZATION IN ORGANELLE%GOBP%GO:0072595	0.27954632	0.87449706	36	0.95031774	1929
POSITIVE REGULATION OF EPIDERMAL CELL DIFFERENTIATION%GOBP%GO:0045606	0.30983874	0.88555634	17	0.95032895	6684
RESPONSE TO ESTROGEN%GOBP%GO:0043627	0.27634287	0.8518666	31	0.95033836	10989
REGULATION OF NUCLEOTIDE METABOLIC PROCESS%GOBP%GO:0006140	0.24921988	0.8743611	119	0.9503409	6512
RNA POLYMERASE III ABORTIVE AND RETRACTIVE INITIATION%REACTOME%R-HSA-749476.2	0.27560863	0.88519394	41	0.9503443	8398
NEGATIVE REGULATION OF INSULIN RECEPTOR SIGNALING PATHWAY%GOBP%GO:0046627	0.29656112	0.88843393	27	0.9503452	11546
FORMATION OF THE TERNARY COMPLEX, AND SUBSEQUENTLY, THE 43S COMPLEX%REACTOME%R-HSA-72695.2	0.26703444	0.87485254	51	0.9503455	5978

POSITIVE REGULATION OF LEUKOCYTE CHEMOTAXIS%GOBP%GO:0002690	0.2673368	0.8800644	63	0.9503458	10945
NUCLEOBASE-CONTAINING COMPOUND TRANSPORT%GOBP%GO:0015931	0.25057605	0.8892372	169	0.9503591	3349
IRS-MEDIATED SIGNALLING%REACTOME%R-HSA-112399.3	0.28566056	0.88530207	30	0.9503625	5602
REGULATION OF GENE SILENCING%GOBP%GO:0060968	0.24861105	0.8702983	117	0.95037	8763
SIGNALING BY RETINOIC ACID%REACTOME%R-HSA-5362517.2	0.2762232	0.8701738	39	0.9503814	8599
NEGATIVE CHEMOTAXIS%GOBP%GO:0050919	0.28703463	0.8871512	31	0.95039254	15428
SPROUTING ANGIOGENESIS%GOBP%GO:0002040	0.26781505	0.87744063	50	0.9504053	11896
POSITIVE REGULATION OF MACROAUTOPHAGY%GOBP%GO:0016239	0.2640388	0.8718151	51	0.95041084	8876
MONOVALENT INORGANIC ANION HOMEOSTASIS%GOBP%GO:0055083	0.3178263	0.8764808	15	0.9504218	12268
LYMPHOCYTE CHEMOTAXIS%GOBP%GO:0048247	0.30543151	0.8809522	21	0.9504242	13528
PI3K-AKT-MTOR SIGNALING PATHWAY AND THERAPEUTIC OPPORTUNITIES%WIKIPATHWAYS_20210110%WP3844%HOMO SAPIENS	0.30139205	0.8763465	23	0.95045245	14021
GLYCOSAMINOGLYCAN METABOLIC PROCESS%GOBP%GO:0030203	0.24492137	0.8521803	120	0.950463	10247
NEGATIVE REGULATION OF LEUKOCYTE ACTIVATION%GOBP%GO:0002695	0.2589532	0.8770478	82	0.95048743	7591
SYNAPTIC ADHESION-LIKE MOLECULES%REACTOME DATABASE ID RELEASE 75%8849932	0.3016767	0.8703621	20	0.950491	11575
CELLULAR PIGMENTATION%GOBP%GO:0033059	0.2733905	0.88011014	44	0.95049137	10410
CELLULAR RESPONSE TO ESTRADIOL STIMULUS%GOBP%GO:0071392	0.31056297	0.8769258	17	0.9505	10989
TRANSPORT	0.2969638	0.87585133	25	0.9505016	541
MODULATION BY HOST OF VIRAL GENOME REPLICATION%GOBP%GO:0044827	0.54093266	1.4766841	15	0.95050544	4524
REGULATION OF MESONEPHROS DEVELOPMENT%GOBP%GO:0061217	0.31257218	0.8724829	17	0.95053005	7094
EPH-EPHRIN MEDIATED REPULSION OF CELLS%REACTOME DATABASE ID RELEASE 75%3928665	0.27149376	0.8760651	46	0.9505442	10885
HORMONE SECRETION%GOBP%GO:0046879	0.28288874	0.8748635	34	0.9505588	10116
CELL-EXTRACELLULAR MATRIX INTERACTIONS%REACTOME%R-HSA-446353.1	0.3011271	0.8518667	16	0.9505603	5877
NUCLEAR IMPORT OF REV PROTEIN%REACTOME DATABASE ID RELEASE 75%180746	0.28570026	0.8756898	31	0.95056134	4994
REGULATION OF INSULIN-LIKE GROWTH FACTOR RECEPTOR SIGNALING PATHWAY%GOBP%GO:0043567	0.31064042	0.8719768	17	0.95056236	10531
COBALAMIN METABOLIC PROCESS%GOBP%GO:0009235	0.31238776	0.8774771	16	0.9505709	125
NEGATIVE REGULATION OF CHROMOSOME ORGANIZATION%GOBP%GO:2001251	0.2551452	0.8718441	106	0.9505874	11921
REGULATION OF RESPONSE TO OXIDATIVE STRESS%GOBP%GO:1902882	0.25526983	0.849572	62	0.9505954	2283
LIPID STORAGE%GOBP%GO:0019915	0.29616764	0.8684865	21	0.95059615	13538
DAP12 SIGNALING%REACTOME DATABASE ID RELEASE 75%2424491	0.51471305	1.4595084	17	0.9506038	4268
ACTIVATION OF NMDA RECEPTORS AND POSTSYNAPTIC EVENTS%REACTOME DATABASE ID RELEASE 75%442755	0.26511762	0.8871572	68	0.95061964	3763
POSITIVE REGULATION OF CHROMATIN ORGANIZATION%GOBP%GO:1905269	0.26122233	0.88038546	78	0.9506235	8737
CELLULAR RESPONSE TO OXIDATIVE STRESS%GOBP%GO:0034599	0.2453256	0.8705273	165	0.9506291	12582
GLIAL CELL ACTIVATION%GOBP%GO:0061900	0.31495276	0.88015795	15	0.95063263	13909
DNA PACKAGING%GOBP%GO:0006323	0.25130466	0.8733701	117	0.9506333	9172
REGULATION OF VASCULAR SMOOTH MUSCLE CELL PROLIFERATION%GOBP%GO:1904705	0.28586137	0.8809647	31	0.9506353	12638
SUMOYLATION OF RNA BINDING PROTEINS%REACTOME DATABASE ID RELEASE 75%4570464	0.2745253	0.8804966	43	0.95063955	4994
REGULATION OF CELL MIGRATION INVOLVED IN SPROUTING ANGIOGENESIS%GOBP%GO:0090049	0.2838717	0.87253946	30	0.9506484	11891
STEROL HOMEOSTASIS%GOBP%GO:0055092	0.26210603	0.8494055	54	0.9506547	8196
PLACENTA DEVELOPMENT%GOBP%GO:0001890	0.27995554	0.87588745	36	0.9506622	5017
POSITIVE REGULATION OF COENZYME METABOLIC PROCESS%GOBP%GO:0051197	0.30067992	0.8521875	18	0.95067036	8098
REGULATION OF PROTEIN ACTIVATION CASCADE%GOBP%GO:2000257	0.47562835	1.4759531	30	0.95068073	3829
MONOAMINE TRANSPORT%WIKIPATHWAYS_20210110%WP727%HOMO SAPIENS	0.2891569	0.85255545	23	0.95068085	13130
TRANSMISSION ACROSS CHEMICAL SYNAPSES%REACTOME%R-HSA-112315.6	0.24417198	0.88024235	224	0.95068717	6324
SULFUR COMPOUND BIOSYNTHETIC PROCESS%GOBP%GO:0044272	0.25011662	0.87491643	148	0.9506873	5288
REGULATION OF ANION TRANSPORT%GOBP%GO:0044070	0.26277038	0.87502885	59	0.95070046	10649
PURINE RIBONUCLEOSIDE MONOPHOSPHATE BIOSYNTHETIC PROCESS%GOBP%GO:0009168	0.26515245	0.8806972	74	0.950702	7952
PROTEIN LOCALIZATION%REACTOME%R-HSA-9609507.2	0.24863426	0.87274337	153	0.95070726	11573
ENDOPLASMIC RETICULUM UNFOLDED PROTEIN RESPONSE%GOBP%GO:0030968	0.258685	0.8872226	94	0.95071214	5400
PACKAGING OF TELOMERE ENDS%REACTOME DATABASE ID RELEASE 75%171306	0.29106236	0.87036526	24	0.9507131	5210
RRNA METABOLIC PROCESS%GOBP%GO:0016072	0.2448292	0.87200904	211	0.9507298	8718
NEGATIVE REGULATION OF DNA-BINDING TRANSCRIPTION FACTOR ACTIVITY%GOBP%GO:0043433	0.25151283	0.87608504	123	0.95073736	11245
POTASSIUM ION TRANSMEMBRANE TRANSPORT%GOBP%GO:0071805	0.24818863	0.85225964	107	0.95076036	7418
IONOTROPIC GLUTAMATE RECEPTOR PATHWAY%PANTHER PATHWAY%P00037	0.27861294	0.8728275	40	0.95077944	10042
HALLMARK_CHOLESTEROL_HOMEOSTASIS%MSIGDB_C2%HALLMARK_CHOLESTEROL_HOMEOSTASIS	0.26410872	0.86850643	56	0.95078874	5591
NICOTINE ACTIVITY ON DOPAMINERGIC NEURONS%WIKIPATHWAYS_20210110%WP1602%HOMO SAPIENS	0.32243404	0.8875239	16	0.95080197	5169
SIGNALING BY NTRK1 (TRKA)%REACTOME%R-HSA-187037.2	0.2565036	0.8874078	110	0.9508049	10501
UPTAKE AND ACTIONS OF BACTERIAL TOXINS%REACTOME DATABASE ID RELEASE 75%5339562	0.30147347	0.88728917	23	0.95080835	8454
VEGFR1 SPECIFIC SIGNALS%PATHWAY INTERACTION DATABASE NCI-NATURE CURATED DATA%VEGFR1 SPECIFIC SIGNALS	0.28699088	0.87508875	26	0.95081747	7004
RAS PROCESSING%REACTOME%R-HSA-9648002.2	0.30242327	0.8754394	19	0.9508262	12905
REGULATION OF TRANSCRIPTION BY RNA POLYMERASE I%GOBP%GO:0006356	0.2807002	0.88052255	31	0.9508276	1764
PLASMA LIPOPROTEIN PARTICLE ASSEMBLY%GOBP%GO:0034377	0.30459818	0.870542	18	0.95083207	15041
REGULATION OF GLYCOLYTIC PROCESS%GOBP%GO:0006110	0.26082116	0.87255937	67	0.95084226	8098
MITOTIC SPINDLE ORGANIZATION%GOBP%GO:0007052	0.2576373	0.8720702	82	0.9508456	6869
REGULATION OF GLUCOSE METABOLIC PROCESS%GOBP%GO:0010906	0.2602826	0.86823374	68	0.95085764	11546
TRYPTOPHAN METABOLISM%WIKIPATHWAYS_20210110%WP465%HOMO SAPIENS	0.28686315	0.8721833	26	0.9508609	8900
HUMAN COMPLEMENT SYSTEM%WIKIPATHWAYS_20210110%WP2806%HOMO SAPIENS	0.43964058	1.421187	47	0.9508629	6471
NEGATIVE REGULATION OF AMYLOID PRECURSOR PROTEIN CATABOLIC PROCESS%GOBP%GO:1902992	0.5401305	1.5514913	19	0.9508759	5841
CELLULAR RESPONSE TO PEPTIDE%GOBP%GO:1901653	0.23927265	0.85256255	211	0.9508875	7921
RNA DESTABILIZATION%GOBP%GO:0050779	0.27752852	0.85230994	30	0.95088977	6970
BIOCARTA_ATM_PATHWAY%MSIGDB_C2%BIOCARTA_ATM_PATHWAY	0.3072107	0.8676134	17	0.95089203	12221
SENSORY PERCEPTION OF SOUND%GOBP%GO:0007605	0.25984696	0.8677324	72	0.9508963	8381
REGULATION OF ACTIN FILAMENT-BASED MOVEMENT%GOBP%GO:1903115	0.52040994	1.6245376	33	0.95092934	5665
MITOCHONDRIAL TRANSLATIONAL ELONGATION%GOBP%GO:0070125	0.2552889	0.8680789	89	0.9509328	6216
EMBRYONIC PLACENTA DEVELOPMENT%GOBP%GO:0001892	0.30905637	0.8751459	19	0.9509407	707
HEDGEHOG SIGNALING EVENTS MEDIATED BY GLI PROTEINS%PATHWAY INTERACTION DATABASE NCI-NATURE CURATED DATA%HEDGEHOG SIGNALING EVENTS MEDIATED BY GLI PROTEINS	0.2687155	0.87297446	48	0.9509549	2385
NEGATIVE REGULATION OF CYTOKINE-MEDIATED SIGNALING PATHWAY%GOBP%GO:0001960	0.26999113	0.87083614	41	0.95095956	13762
REGULATION OF PEPTIDE HORMONE SECRETION%GOBP%GO:0090276	0.25480512	0.88755727	123	0.95097864	8214
HORMONE METABOLIC PROCESS%GOBP%GO:0042445	0.24881285	0.8528802	111	0.9509789	10137
NEGATIVE REGULATION OF NF-KAPPAB TRANSCRIPTION FACTOR ACTIVITY%GOBP%GO:0032088	0.26271015	0.872841	63	0.95098734	7202
ACTIVATION OF THE PRE-REPLICATIVE COMPLEX%REACTOME DATABASE ID RELEASE 75%68962	0.27986705	0.86792034	32	0.9509973	5812
RESPONSE TO PEPTIDE HORMONE%GOBP%GO:0043434	0.24398345	0.87523115	202	0.9509989	6255
ERROR-PRONE TRANSLESION SYNTHESIS%GOBP%GO:0042276	0.29274786	0.85274965	20	0.951006	14199
CELLULAR SENESCENCE%REACTOME%R-HSA-2559583.4	0.24260703	0.8526106	147	0.95102483	9837
CEREBELLAR CORTEX MORPHOGENESIS%GOBP%GO:0021696	0.30972776	0.8706782	16	0.95103794	162
AMINOGLYCAN METABOLIC PROCESS%GOBP%GO:0006022	0.24925555	0.8705457	131	0.9510538	10480
POSITIVE REGULATION OF REACTIVE OXYGEN SPECIES BIOSYNTHETIC PROCESS%GOBP%GO:1903428	0.2910861	0.87089866	25	0.95107126	8541
NCRNA TRANSCRIPTION%GOBP%GO:0098781	0.25434718	0.8677472	87	0.9510978	5872
SYMPATHETIC NERVOUS SYSTEM DEVELOPMENT%GOBP%GO:0048485	0.5398554	1.4751754	16	0.9511004	5930
NEUROTRANSMITTER RELEASE CYCLE%REACTOME%R-HSA-112310.5	0.26828218	0.87301123	46	0.95111537	9035
RIPK1-MEDIATED REGULATED NECROSIS%REACTOME DATABASE ID RELEASE 75%5213460	0.27910525	0.85291266	27	0.95114774	9054
COPPER HOMEOSTASIS%WIKIPATHWAYS_20210110%WP3286%HOMO SAPIENS	0.28147188	0.88792634	40	0.95115685	12842
REGULATION OF STAT CASCADE%GOBP%GO:1904892	0.25868395	0.86722636	74	0.951172	3153
HALLMARK_ADIPOGENESIS%MSIGDB_C2%HALLMARK_ADIPOGENESIS	0.2530013	0.8875671	148	0.9511978	6209
INTEGRIN CELL SURFACE INTERACTIONS%REACTOME%R-HSA-216083.3	0.267656	0.87094235	53	0.95121455	11800
PRADER-WILLI AND ANGELMAN SYNDROME%WIKIPATHWAYS_20210110%WP3998%HOMO SAPIENS	0.2613085	0.8535135	51	0.9512209	6179
METALLOPROTEASE DUBS%REACTOME DATABASE ID RELEASE 75%5689901	0.48164567	1.4244831	24	0.95122457	6210
BILE ACID BIOSYNTHETIC PROCESS%GOBP%GO:0006699	0.29333845	0.856755	22	0.9512562	12613
SUBSTANTIA NIGRA DEVELOPMENT%GOBP%GO:0021762	0.2710737	0.86250156	41	0.9513055	1275
REGULATION OF PTEN GENE TRANSCRIPTION%REACTOME%R-HSA-8943724.1	0.2590366	0.85294807	58	0.95130795	7813
PID_EPO_PATHWAY%MSIGDB_C2%PID_EPO_PATHWAY	0.28586817	0.86238766	26	0.9513087	3310
BIOCARTA_NUCLEARRS_PATHWAY%MSIGDB_C2%BIOCARTA_NUCLEARRS_PATHWAY	0.31160524	0.8565898	15	0.95132387	14483
NEGATIVE REGULATION OF TRANSLATIONAL INITIATION%GOBP%GO:0045947	0.30205193	0.85682553	18	0.95135397	10576
RRNA MODIFICATION IN THE NUCLEUS AND CYTOSOL%REACTOME%R-HSA-6790901.4	0.26147497	0.8672429	59	0.9513654	1764
DUAL INCISION IN GG-NER%REACTOME%R-HSA-5696400.1	0.2683664	0.8538077	40	0.95136595	14199
NEGATIVE REGULATION OF DOUBLE-STRAND BREAK REPAIR VIA HOMOLOGOUS RECOMBINATION%GOBP%GO:2000042	0.3129997	0.8712106	17	0.9513681	7254
POSITIVE REGULATION OF LEUKOCYTE MEDIATED IMMUNITY%GOBP%GO:0002705	0.26264307	0.8710806	66	0.9513881	6569
POLYOL BIOSYNTHETIC PROCESS%GOBP%GO:0046173	0.26775697	0.8535436	36	0.9513926	5788
REGULATION OF LEUKOCYTE DEGRANULATION%GOBP%GO:0043300	0.2838131	0.8616175	26	0.95140606	5816
RELEASE OF SEQUESTERED CALCIUM ION INTO CYTOSOL%GOBP%GO:0051209	0.29533744	0.8569193	20	0.9514122	3051
DAP12 INTERACTIONS%REACTOME%R-HSA-2172127.1	0.5122876	1.4710655	21	0.951418	5573
SARS-COV INFECTIONS%REACTOME DATABASE ID RELEASE 75%9679506	0.24803369	0.8625547	137	0.95142204	8585
ALZHEIMER DISEASE-AMYLOID SECRETASE PATHWAY%PANTHER PATHWAY%P00003	0.26400238	0.87095267	63	0.951423	10539
PLASMA LIPOPROTEIN PARTICLE REMODELING%GOBP%GO:0034369	0.29901853	0.8641535	19	0.95142674	8196
METABOLISM OF FAT-SOLUBLE VITAMINS%REACTOME%R-HSA-6806667.6	0.27080423	0.8538931	38	0.9514351	6121
POSITIVE REGULATION OF PHOSPHATIDYLINOSITOL 3-KINASE ACTIVITY%GOBP%GO:0043552	0.3145461	0.8875678	18	0.9514356	104
REGULATION OF TELOMERASE ACTIVITY%GOBP%GO:0051972	0.26153985	0.852987	48	0.9514667	12868
THIOESTER BIOSYNTHETIC PROCESS%GOBP%GO:0035384	0.27306953	0.86218566	36	0.9514679	8269
NUCLEAR SIGNALING BY ERBB4%REACTOME DATABASE ID RELEASE 75%1251985	0.4618915	1.4230331	28	0.95147413	8059
MONOCARBOXYLIC ACID BIOSYNTHETIC PROCESS%GOBP%GO:0072330	0.24261957	0.8536244	150	0.9514767	5288
REGULATED NECROSIS%REACTOME%R-HSA-5218859.3	0.27910525	0.8486597	27	0.95149153	9054
POSITIVE REGULATION OF NUCLEAR-TRANSCRIBED MRNA CATABOLIC PROCESS, DEADENYLATION-DEPENDENT DECAY%GOBP%GO:1900153	0.30079645	0.8614535	18	0.95149356	4704
GLUTAMATE METABOLIC PROCESS%GOBP%GO:0006536	0.29118454	0.8620538	21	0.9514965	4354
TRANSPORT OF THE SLBP INDEPENDENT MATURE MRNA%REACTOME%R-HSA-159227.2	0.2715817	0.84879357	32	0.95149815	4994
POSITIVE REGULATION OF HEMOPOIESIS%GOBP%GO:1903708	0.24637404	0.8561235	117	0.9515037	6935
PID_IL12_2PATHWAY%MSIGDB_C2%PID_IL12_2PATHWAY	0.27525914	0.8626155	35	0.95153576	9096
REGULATION OF HUMORAL IMMUNE RESPONSE%GOBP%GO:0002920	0.4458487	1.4077476	34	0.9515369	3829

NUCLEAR ENVELOPE REASSEMBLY%GOBP%GO:0031468	0.31296876	0.8876207	18	0.9515566	11648
HALLMARK_APOPTOSIS%MSIGDB_C2%HALLMARK_APOPTOSIS	0.24839094	0.85695547	111	0.9515689	6568
NEGATIVE REGULATION OF ENDOCYTOSIS%GOBP%GO:0045806	0.25750932	0.8484781	50	0.9515783	11179
PROTEIN LOCALIZATION TO PEROXISOME%GOBP%GO:0072662	0.25405	0.86418796	63	0.9515923	7116
POSITIVE REGULATION OF GLYCOLYTIC PROCESS%GOBP%GO:0045821	0.30067992	0.8611533	18	0.95159626	8098
MITOCHONDRION LOCALIZATION%GOBP%GO:0051646	0.27008763	0.85543424	36	0.9516031	6322
SEMAPHORIN-PLEXIN SIGNALING PATHWAY%GOBP%GO:0071526	0.27208236	0.854174	36	0.951608	7434
I-KAPPAB KINASE/NF-KAPPAB SIGNALING%GOBP%GO:0007249	0.25651392	0.853029	56	0.9516154	13587
PROTEIN-CONTAINING COMPLEX LOCALIZATION%GOBP%GO:0031503	0.23835614	0.85618234	217	0.9516204	5707
REGULATION OF POSTTRANSCRIPTIONAL GENE SILENCING%GOBP%GO:0060147	0.25377446	0.8616208	87	0.95162624	8514
ALCOHOL BIOSYNTHETIC PROCESS%GOBP%GO:0046165	0.2552712	0.8618724	73	0.9516281	6121
MITOCHONDRIAL TRANSLATIONAL TERMINATION%GOBP%GO:0070126	0.24882573	0.8555397	89	0.95163876	6216
MITOCHONDRIAL TRANSLATION TERMINATION%REACTOME%R-HSA-5419276.1	0.25132298	0.8539007	88	0.95164216	6216
WATER-SOLUBLE VITAMIN METABOLIC PROCESS%GOBP%GO:0006767	0.25651127	0.85703385	69	0.95164603	10327
LISSENCEPHALY GENE (LIS1) IN NEURONAL MIGRATION AND DEVELOPMENT%PATHWAY INTERACTION DATABASE NCI-NATURE CURATED DATA%LISSENCEPHALY GENE (LIS1) IN NEURONAL MIGRATION AND DEVELOPMENT	0.515778	1.6113853	29	0.9516474	2466
SIGNALING EVENTS MEDIATED BY STEM CELL FACTOR RECEPTOR (C-KIT)%PATHWAY INTERACTION DATABASE NCI-NATURE CURATED DATA%SIGNALING EVENTS MEDIATED BY STEM CELL FACTOR RECEPTOR (C-KIT)	0.2606986	0.8550346	48	0.9516521	9509
POSITIVE REGULATION OF FILOPODIUM ASSEMBLY%GOBP%GO:0051491	0.28166226	0.85477287	24	0.9516565	5930
CELLULAR RESPONSE TO TRANSFORMING GROWTH FACTOR BETA STIMULUS%GOBP%GO:0071560	0.24624625	0.8542722	109	0.9516565	4704
INTRA-GOLGI VESICLE-MEDIATED TRANSPORT%GOBP%GO:0006891	0.2714238	0.8540096	31	0.9516672	4505
BIOCARTA_EGF_PATHWAY%MSIGDB_C2%BIOCARTA_EGF_PATHWAY	0.29593718	0.85850984	22	0.95166904	9509
REGULATION OF NEURON PROJECTION ARBORIZATION%GOBP%GO:0150011	0.5238884	1.4581349	15	0.9516712	1332
PID_BARD1_PATHWAY%MSIGDB_C2%PID_BARD1_PATHWAY	0.27980593	0.85589904	25	0.95168483	8740
REGULATION OF NUCLEOTIDE CATABOLIC PROCESS%GOBP%GO:0030811	0.26082116	0.86265266	67	0.9516872	8098
REGULATION OF INTRINSIC APOPTOTIC SIGNALING PATHWAY IN RESPONSE TO DNA DAMAGE%GOBP%GO:1902229	0.28136668	0.85562634	30	0.9517082	3656
REGULATION OF EPIDERMIS DEVELOPMENT%GOBP%GO:0045682	0.26526198	0.8631475	46	0.95170933	6684
TRK RECEPTOR SIGNALING MEDIATED BY THE MAPK PATHWAY%PATHWAY INTERACTION DATABASE NCI-NATURE CURATED DATA%TRK RECEPTOR SIGNALING MEDIATED BY THE MAPK PATHWAY	0.26906022	0.85487056	34	0.9517138	12437
TP53 REGULATES TRANSCRIPTION OF GENES INVOLVED IN CYTOCHROME C RELEASE%REACTOME%R-HSA-6803204.1	0.5146816	1.4676578	19	0.9517207	4030
POTASSIUM ION TRANSPORT%GOBP%GO:0006813	0.24276403	0.8482642	116	0.9517276	7418
SERINE FAMILY AMINO ACID METABOLIC PROCESS%GOBP%GO:0009069	0.27529263	0.8666858	36	0.95172966	4354
SUMOYLATION OF UBIQUITINYLATION PROTEINS%REACTOME%R-HSA-3232142.3	0.27631697	0.8652103	35	0.95173514	4996
NEGATIVE REGULATION OF TRANSMEMBRANE RECEPTOR PROTEIN SERINE/THREONINE KINASE SIGNALING PATHWAY%GOBP%GO:0090101	0.24771997	0.8543534	96	0.9517385	7534
REGULATION OF EPITHELIAL CELL PROLIFERATION%GOBP%GO:0050678	0.23899081	0.8562396	212	0.9517435	7352
REGULATION OF GLUCOSE TRANSMEMBRANE TRANSPORT%GOBP%GO:0010827	0.26699936	0.86337227	44	0.95174617	8214
RELEASE 75%5625886	0.2895316	0.8642274	24	0.9517501	3656
REGULATION OF MONONUCLEAR CELL MIGRATION%GOBP%GO:0071675	0.27276936	0.8616752	34	0.9517502	11417
PROTEIN-LIPID COMPLEX REMODELING%GOBP%GO:0034368	0.29901853	0.8583339	19	0.95175505	8196
POSITIVE REGULATION OF PHOSPHATIDYLINOSITOL 3-KINASE SIGNALING%GOBP%GO:0014068	0.256062	0.8573332	63	0.9517634	9785
TUBE CLOSURE%GOBP%GO:0060606	0.2732563	0.8611814	34	0.95177466	13223
BIOCARTA_GSK3_PATHWAY%MSIGDB_C2%BIOCARTA_GSK3_PATHWAY	0.53557605	1.4696457	16	0.9517965	4020
IMPORT ACROSS PLASMA MEMBRANE%GOBP%GO:0098739	0.25346452	0.85856664	96	0.9518014	10244
RETINOID METABOLISM AND TRANSPORT%REACTOME%R-HSA-975634.2	0.27913573	0.86676955	35	0.95180184	4090
PHOSPHOLIPID DEPHOSPHORYLATION%GOBP%GO:0046839	0.27360466	0.86054474	37	0.951805	6928
SIGNALING BY NTRKS%REACTOME%R-HSA-166520.4	0.24657299	0.8570701	129	0.9518059	10501
NEGATIVE REGULATION OF AXONOGENESIS%GOBP%GO:0050771	0.2650425	0.8544357	45	0.9518082	8752
THERMOGENESIS%WIKIPATHWAYS_20210110%WP4321%HOMO SAPIENS	0.25132424	0.8530427	80	0.95181197	11091
REGULATION OF INTERLEUKIN-6 PRODUCTION%GOBP%GO:0032675	0.25492746	0.85506064	69	0.9518284	8102
PID_TRAIL_PATHWAY%MSIGDB_C2%PID_TRAIL_PATHWAY	0.46830893	1.4025028	24	0.95184714	4417
REGULATION OF RESPONSE TO DNA DAMAGE STIMULUS%GOBP%GO:2001020	0.2401382	0.85567224	202	0.9518526	8413
CHONDROITIN SULFATE METABOLIC PROCESS%GOBP%GO:0030204	0.27785727	0.84182435	29	0.95185286	10191
RRNA PROCESSING%GOBP%GO:0006364	0.24327497	0.8631897	187	0.9518583	8718
FOXO-MEDIATED TRANSCRIPTION OF OXIDATIVE STRESS, METABOLIC AND NEURONAL GENES%REACTOME DATABASE ID RELEASE 75%9615017	0.28614086	0.862676	25	0.9518681	12223
CELL REDOX HOMEOSTASIS%GOBP%GO:0045454	0.27378708	0.841683	31	0.95187604	5778
ACIDIC AMINO ACID TRANSPORT%GOBP%GO:0015800	0.2743012	0.8545224	34	0.9518827	9035
LXR-MEDIATED SIGNALING%REACTOME DATABASE ID RELEASE 75%9024446	0.27581364	0.86426824	38	0.9519047	9188
POSTMITOTIC NUCLEAR PORE COMPLEX (NPC) REFORMATION%REACTOME DATABASE ID RELEASE 75%9615933	0.28354466	0.8406172	24	0.95190793	4994
RNA CATABOLIC PROCESS%GOBP%GO:0006401	0.2362575	0.8551454	231	0.95191044	3651
SKIN EPIDERMIS DEVELOPMENT%GOBP%GO:0098773	0.30673602	0.8573586	16	0.9519405	2757
HALLMARK_TGF_BETA_SIGNALING%MSIGDB_C2%HALLMARK_TGF_BETA_SIGNALING	0.27054182	0.862882	43	0.95194393	7491
ORGANIC ACID BIOSYNTHETIC PROCESS%GOBP%GO:0016053	0.24097332	0.86522126	240	0.9519459	5288
MUSCARINIC ACETYLCHOLINE RECEPTOR 2 AND 4 SIGNALING PATHWAY%PANTHER PATHWAY%P00043	0.26170304	0.84800565	51	0.9519499	11141
ANTIBACTERIAL HUMORAL RESPONSE%GOBP%GO:0019731	0.29703736	0.8571098	19	0.95195466	7071
REGULATION OF CHROMATIN SILENCING%GOBP%GO:0031935	0.27905902	0.86362684	29	0.9519552	8763
MODIFIED AMINO ACID TRANSPORT%GOBP%GO:0072337	0.2943421	0.86057836	22	0.9519662	486
NUCLEAR RECEPTORS IN LIPID METABOLISM AND TOXICITY%WIKIPATHWAYS_20210110%WP299%HOMO SAPIENS	0.31160524	0.863375	15	0.95196766	14483
FLT3 SIGNALING IN DISEASE%REACTOME%R-HSA-9682385.1	0.28998628	0.8660731	26	0.9519679	8274
TRNA 5'-END PROCESSING%GOBP%GO:0099116	0.31753922	0.866432	15	0.95198536	1901
EPITHELIAL CELL MIGRATION%GOBP%GO:0010631	0.2564699	0.8590848	62	0.952	13653
NEGATIVE REGULATION OF HEMOPOIESIS%GOBP%GO:1903707	0.25348982	0.84186846	77	0.9520025	7447
NEGATIVE REGULATION OF NIK/NF-KAPPAB SIGNALING%GOBP%GO:1901223	0.3055669	0.85857856	17	0.95200366	6538
POSITIVE REGULATION OF RECEPTOR INTERNALIZATION%GOBP%GO:0002092	0.29358095	0.84068304	20	0.9520078	7174
SNRNA TRANSCRIPTION%GOBP%GO:0009301	0.25707254	0.8661743	70	0.9520093	5629
PEPTIDYL-ASPARAGINE MODIFICATION%GOBP%GO:0018196	0.4560035	1.4016237	29	0.9520129	9088
NEGATIVE REGULATION OF INTERLEUKIN-6 PRODUCTION%GOBP%GO:0032715	0.29327315	0.8401533	18	0.9520135	5213
REGULATION OF PROTEIN SECRETION%GOBP%GO:0050708	0.24253625	0.86371386	176	0.95202225	8214
DEPHOSPHORYLATION%GOBP%GO:0016311	0.24268398	0.86530215	185	0.9520322	6165
POSITIVE REGULATION OF AUTOPHAGY%GOBP%GO:0010508	0.24813429	0.8574213	106	0.9520417	8876
CARBOXYLIC ACID BIOSYNTHETIC PROCESS%GOBP%GO:0046394	0.24097332	0.86627245	240	0.9520457	5288
REGULATION OF GENE SILENCING BY MIRNA%GOBP%GO:0060964	0.24851823	0.84250075	86	0.9520462	8514
HETEROPHILIC CELL-CELL ADHESION VIA PLASMA MEMBRANE CELL ADHESION MOLECULES%GOBP%GO:0007157	0.27738446	0.85804343	31	0.9520493	7759
LIPID HOMEOSTASIS%GOBP%GO:0055088	0.24384849	0.8403941	98	0.9520608	8214
PROTEIN LOCALIZATION TO ENDOPLASMIC RETICULUM%GOBP%GO:0070972	0.24729846	0.8626896	133	0.9520679	8277
SENSORY ORGAN MORPHOGENESIS%GOBP%GO:0090596	0.24482195	0.84196657	106	0.9520689	7112
EMBRYONIC EYE MORPHOGENESIS%GOBP%GO:0048048	0.29342106	0.8586653	21	0.95207876	6191
REGULATION OF CELL-MATRIX ADHESION%GOBP%GO:0001952	0.25258362	0.86429316	88	0.95208496	3949
BIOCARTA_IL2RB_PATHWAY%MSIGDB_C2%BIOCARTA_IL2RB_PATHWAY	0.28908268	0.86075526	24	0.9520926	13503
HEME METABOLIC PROCESS%GOBP%GO:0042168	0.2863262	0.858779	23	0.9520972	3593
POSITIVE REGULATION OF CELLULAR CARBOHYDRATE METABOLIC PROCESS%GOBP%GO:0010676	0.27086544	0.84075016	30	0.95210594	12660
NEGATIVE REGULATION OF CELL DEVELOPMENT%GOBP%GO:0010721	0.2441941	0.86062175	165	0.95210755	9395
TRNA PROCESSING%REACTOME%R-HSA-72306.6	0.25020626	0.86342293	104	0.95211107	7835
EPHRIN B REVERSE SIGNALING%PATHWAY INTERACTION DATABASE NCI-NATURE CURATED DATA%EPHRIN B REVERSE SIGNALING	0.2826199	0.83902705	22	0.9521231	6134
PID_ERBB1_DOWNSTREAM_PATHWAY%MSIGDB_C2%PID_ERBB1_DOWNSTREAM_PATHWAY	0.24873522	0.842051	83	0.9521534	11091
LAGGING STRAND SYNTHESIS%REACTOME DATABASE ID RELEASE 75%69186	0.2915346	0.8413755	20	0.9521578	3931
CARDIAC SEPTUM DEVELOPMENT%GOBP%GO:0003279	0.2541966	0.8391383	57	0.9521627	14496
HDR THROUGH HOMOLOGOUS RECOMBINATION (HRR) OR SINGLE STRAND ANNEALING (SSA)%REACTOME%R-HSA-5693567.2	0.24901414	0.8575976	104	0.95217824	12765
INTERLEUKIN SIGNALING PATHWAY%PANTHER PATHWAY%P00036	0.25141665	0.842543	68	0.9521947	11345
EYE DEVELOPMENT%GOBP%GO:0001654	0.23339836	0.8219635	165	0.9521967	8958
ERBB SIGNALING PATHWAY%GOBP%GO:0038127	0.25386572	0.8401752	58	0.95219743	9727
CELLULAR POTASSIUM ION TRANSPORT%GOBP%GO:0071804	0.24818863	0.8591021	107	0.9521977	7418
POSITIVE REGULATION OF TUMOR NECROSIS FACTOR SUPERFAMILY CYTOKINE PRODUCTION%GOBP%GO:1903557	0.26123396	0.84082526	48	0.9522002	6569
TERMINATION OF RNA POLYMERASE II TRANSCRIPTION%GOBP%GO:0006369	0.27558857	0.86545146	37	0.9522046	3487
'DE NOVO' POSTTRANSLATIONAL PROTEIN FOLDING%GOBP%GO:0051084	0.27203932	0.8473447	31	0.9522171	11894
PID_INSULIN_PATHWAY%MSIGDB_C2%PID_INSULIN_PATHWAY	0.27699107	0.86532414	36	0.9522189	4465
HALLMARK_MYC_TARGETS_V2%MSIGDB_C2%HALLMARK_MYC_TARGETS_V2	0.26010385	0.84368473	45	0.95223594	12761
REGULATION OF SODIUM ION TRANSPORT%GOBP%GO:0002028	0.25930464	0.8643268	70	0.9522547	6246
EPO%IOB%EPO	0.26324022	0.83881444	40	0.95226115	7719
SLC-MEDIATED TRANSMEMBRANE TRANSPORT%REACTOME DATABASE ID RELEASE 75%425407	0.23969267	0.8574231	189	0.95226336	6668
MITOCHONDRIAL PROTEIN IMPORT%REACTOME DATABASE ID RELEASE 75%1268020	0.25527418	0.8576681	64	0.95227134	11455
REGULATION OF MEGAKARYOCYTE DIFFERENTIATION%GOBP%GO:0045652	0.2604365	0.84223443	48	0.95228237	6689
REGULATION OF RIBOSOME BIOGENESIS%GOBP%GO:0090069	0.29605362	0.8220445	15	0.9522843	1764
NEGATIVE REGULATION OF CALCIUM ION TRANSMEMBRANE TRANSPORT%GOBP%GO:1903170	0.27950183	0.84327155	26	0.9522867	3787
GENERAL TRANSCRIPTION REGULATION%PANTHER PATHWAY%P00023	0.27783862	0.8587965	32	0.9522895	8326
PROTEIN MONOUBIQUITINATION%GOBP%GO:0006513	0.2520674	0.8268738	56	0.9523076	2545
REGULATION OF TOLL-LIKE RECEPTOR 4 SIGNALING PATHWAY%GOBP%GO:0034143	0.3002457	0.8391801	15	0.95231014	12849
CYTOCHROME P450 - ARRANGED BY SUBSTRATE TYPE%REACTOME%R-HSA-211897.2	0.27206957	0.8577672	37	0.95231414	10371
SIGNALING BY RAS MUTANTS%REACTOME%R-HSA-6802949.2	0.26108727	0.82714	38	0.9523297	4909
BIOCARTA_HDAC_PATHWAY%MSIGDB_C2%BIOCARTA_HDAC_PATHWAY	0.29845732	0.84207284	17	0.95233095	11273
POLYSACCHARIDE CATABOLIC PROCESS%GOBP%GO:0000272	0.5030088	1.3856562	16	0.95233524	8534
PID_CXCR4_PATHWAY%MSIGDB_C2%PID_CXCR4_PATHWAY	0.26070297	0.8656203	66	0.9523525	4268
METALLO-SULFUR CLUSTER ASSEMBLY%GOBP%GO:0031163	0.2881868	0.8473902	22	0.95235926	5242
LIPID MODIFICATION%GOBP%GO:0030258	0.24290915	0.85913575	165	0.95236045	7116
RESPONSE TO PH%GOBP%GO:0009268	0.2955697	0.840858	18	0.95236206	13585
ACYL-COA BIOSYNTHETIC PROCESS%GOBP%GO:0071616	0.27306953	0.8654759	36	0.95238906	8269
PID_PRL_SIGNALING_EVENTS_PATHWAY%MSIGDB_C2%PID_PRL_SIGNALING_EVENTS_PATHWAY	0.2931875	0.842555	19	0.9523938	13833
PH REDUCTION%GOBP%GO:0045851	0.26847425	0.8643737	42	0.95240515	8331

SNRNA METABOLIC PROCESS%GOBP%GO:0016073	0.24649015	0.8437088	93	0.9524196	5870
GLUTATHIONE METABOLIC PROCESS%GOBP%GO:0006749	0.27324203	0.860078	37	0.9524205	5007
POSITIVE REGULATION OF CHROMOSOME SEGREGATION%GOBP%GO:0051984	0.26399973	0.83924454	33	0.95242053	6680
POST NMDA RECEPTOR ACTIVATION EVENTS%REACTOME DATABASE ID RELEASE 75%438064	0.25031716	0.82693124	55	0.9524278	14567
TRNA MODIFICATION%GOBP%GO:0006400	0.247128	0.84094983	73	0.95242804	9322
VENTRICULAR SEPTUM DEVELOPMENT%GOBP%GO:0003281	0.26843047	0.84747434	36	0.95243144	13568
FERROPTOSIS%WIKIPATHWAYS_20210110%WP4313%HOMO SAPIENS	0.27238294	0.8220825	25	0.9524383	4354
INSULIN RECEPTOR SIGNALLING CASCADE%REACTOME DATABASE ID RELEASE 75%74751	0.26121712	0.82733685	36	0.9524514	13549
CELLULAR RESPONSE TO CADMIUM ION%GOBP%GO:0071276	0.28303495	0.83032244	23	0.9524583	12728
GLYCOSAMINOGLYCAN BIOSYNTHETIC PROCESS%GOBP%GO:0006024	0.24042803	0.8222046	94	0.9524628	10247
RNA LOCALIZATION%GOBP%GO:0006403	0.23828363	0.84329444	164	0.95246875	4994
INHIBITION OF VOLTAGE GATED CA2+ CHANNELS VIA GBETA GAMMA SUBUNITS%REACTOME DATABASE ID RELEASE 75%997272	0.46828273	1.4214313	28	0.95249844	6719
NEPHROTIC SYNDROME%WIKIPATHWAYS_20210110%WP4758%HOMO SAPIENS	0.27862754	0.86566085	30	0.9525033	14145
DENDRITIC SPINE MORPHOGENESIS%GOBP%GO:0060997	0.29543635	0.8271562	17	0.952519	8879
REGULATION OF GLIOGENESIS%GOBP%GO:0014013	0.2614358	0.8597685	46	0.95253706	9716
G1 S TRANSITION%REACTOME DATABASE ID RELEASE 75%69206	0.24490677	0.8591623	129	0.9525385	13833
REGULATION OF LEUKOCYTE MEDIATED IMMUNITY%GOBP%GO:0002703	0.23896231	0.8305391	101	0.9525499	6569
POSITIVE REGULATION OF LYMPHOCYTE DIFFERENTIATION%GOBP%GO:0045621	0.25483328	0.8395547	55	0.95256275	8693
POSITIVE REGULATION OF T CELL CYTOKINE PRODUCTION%GOBP%GO:0002726	0.3111894	0.8644086	15	0.95256317	4417
POSITIVE REGULATION OF INTERLEUKIN-1 BETA PRODUCTION%GOBP%GO:0032731	0.27342144	0.8475137	31	0.9525795	9399
VITAMIN METABOLIC PROCESS%GOBP%GO:0006766	0.24597114	0.8409803	88	0.95259374	10327
MEIOTIC RECOMBINATION%REACTOME%R-HSA-912446.2	0.26381546	0.84373707	41	0.95259565	12495
PDGFR-BETA SIGNALING PATHWAY%PATHWAY INTERACTION DATABASE NCI-NATURE CURATED DATA%PDGFR-BETA SIGNALING PATHWAY	0.24270993	0.8425629	117	0.9525998	5884
IRON-SULFUR CLUSTER ASSEMBLY%GOBP%GO:0016226	0.2881868	0.86463004	22	0.9526021	5242
NEGATIVE REGULATION OF PROTEIN KINASE B SIGNALING%GOBP%GO:0051898	0.2755575	0.8645125	36	0.95260394	4842
TRANSCYTOSIS%GOBP%GO:0045056	0.30045408	0.8303645	15	0.9526055	14170
EMBRYONIC HEART TUBE MORPHOGENESIS%GOBP%GO:0003143	0.25918764	0.82084155	39	0.9526152	7588
POSITIVE REGULATION OF SMOOTH MUSCLE CELL MIGRATION%GOBP%GO:0014911	0.30045614	0.84280944	17	0.9526165	12638
REGULATION OF SMOOTH MUSCLE CELL PROLIFERATION%GOBP%GO:0048660	0.24647968	0.8273638	69	0.9526241	10558
CGMP EFFECTS%REACTOME%R-HSA-418457.1	0.30729607	0.83964485	15	0.9526263	3649
CALCIUM ION TRANSPORT INTO CYTOSOL%GOBP%GO:0060402	0.2625899	0.8368927	42	0.95262694	3100
AMINOGLYCAN BIOSYNTHETIC PROCESS%GOBP%GO:0006023	0.2457576	0.83924675	100	0.9526341	10480
POSITIVE REGULATION OF NUCLEOTIDE CATABOLIC PROCESS%GOBP%GO:0030813	0.30067992	0.8469749	18	0.9526365	8098
BRANCHED-CHAIN AMINO ACID CATABOLISM%REACTOME%R-HSA-70895.4	0.29820588	0.8598382	21	0.9526461	9777
FAT CELL DIFFERENTIATION%GOBP%GO:0045444	0.25872815	0.8429187	52	0.9526493	9607
EPITHELIUM MIGRATION%GOBP%GO:0090132	0.255869	0.8426513	64	0.952664	12734
PIGMENTATION%GOBP%GO:0043473	0.24712765	0.82842773	63	0.9526641	10410
CENTROSOME LOCALIZATION%GOBP%GO:0051642	0.2845217	0.822209	20	0.9526688	7557
POSITIVE REGULATION OF SECRETION BY CELL%GOBP%GO:1903532	0.23059551	0.823581	163	0.95266896	11558
VIRAL ENTRY INTO HOST CELL%GOBP%GO:0046718	0.47887912	1.470202	28	0.9526708	6935
REGULATION OF SYNAPSE STRUCTURE OR ACTIVITY%GOBP%GO:0050803	0.24457957	0.84329915	122	0.95268136	11575
POSITIVE REGULATION OF SIGNAL TRANSDUCTION BY P53 CLASS MEDIATOR%GOBP%GO:1901798	0.2848823	0.82093203	20	0.95268214	11724
NEURAL NUCLEUS DEVELOPMENT%GOBP%GO:0048857	0.2595176	0.8446165	46	0.9526851	6298
FACTORS INVOLVED IN MEGAKARYOCYTE DEVELOPMENT AND PLATELET PRODUCTION%REACTOME%R-HSA-983231.2	0.23780683	0.82148397	112	0.9526859	4127
REGULATION OF GENE SILENCING BY RNA%GOBP%GO:0060966	0.25377446	0.85920244	87	0.9526886	8514
REGULATION OF TELOMERE MAINTENANCE VIA TELOMERE LENGTHENING%GOBP%GO:1904356	0.25005612	0.8305817	58	0.95269495	9660
VITAMIN D-SENSITIVE CALCIUM SIGNALING IN DEPRESSION%WIKIPATHWAYS_20210110%WP4698%HOMO SAPIENS	0.2756344	0.8206567	25	0.9527092	4566
REGULATION OF COMPLEMENT ACTIVATION%GOBP%GO:0030449	0.47562835	1.4661998	30	0.95271343	3829
REGULATION OF SODIUM ION TRANSMEMBRANE TRANSPORTER ACTIVITY%GOBP%GO:2000649	0.2585196	0.8393365	46	0.95271605	6080
METABOLIC DISORDERS OF BIOLOGICAL OXIDATION ENZYMES%REACTOME%R-HSA-5579029.2	0.28099206	0.8369644	24	0.95272267	14367
INITIATION OF NUCLEAR ENVELOPE (NE) REFORMATION%REACTOME%R-HSA-2995383.5	0.29431593	0.8277062	16	0.9527256	2914
REGULATION OF RNA SPLICING%GOBP%GO:0043484	0.2372961	0.8308203	139	0.95274955	11537
REGULATION OF CAMP-MEDIATED SIGNALING%GOBP%GO:0043949	0.2770596	0.8264567	27	0.9527518	7645
SERINE FAMILY AMINO ACID BIOSYNTHETIC PROCESS%GOBP%GO:0009070	0.29511043	0.82297826	16	0.9527532	5997
PARADOXICAL ACTIVATION OF RAF SIGNALING BY KINASE INACTIVE BRAF%REACTOME%R-HSA-6802955.2	0.26108727	0.8299938	38	0.9527556	4909
DISEASES OF GLYCOSYLATION%REACTOME%R-HSA-3781865.1	0.23504004	0.82323396	135	0.95276976	8607
TELOMERE MAINTENANCE VIA TELOMERE LENGTHENING%GOBP%GO:0010833	0.2703631	0.8254488	25	0.9527726	13165
STEROL BIOSYNTHETIC PROCESS%GOBP%GO:0016126	0.27406463	0.8437628	33	0.9527766	3693
SMOOTH MUSCLE CONTRACTION%REACTOME DATABASE ID RELEASE 75%445355	0.2592777	0.8226855	37	0.9527785	13514
ASTROCYTE DIFFERENTIATION%GOBP%GO:0048708	0.29549873	0.8298472	17	0.9527802	1440
NEGATIVE REGULATION OF SEQUESTERING OF CALCIUM ION%GOBP%GO:0051283	0.27976596	0.82930565	21	0.95278233	3051
HDR THROUGH HOMOLOGOUS RECOMBINATION (HRR)%REACTOME%R-HSA-5685942.1	0.2562897	0.84468997	64	0.95278305	12221
CELLULAR GLUCOSE HOMEOSTASIS%GOBP%GO:0001678	0.2549498	0.8294372	45	0.952789	11932
SUMOYLATION OF TRANSCRIPTION COFACTORS%REACTOME DATABASE ID RELEASE 75%3899300	0.2581567	0.8248794	42	0.9527917	1831
REGULATION OF TRANSCRIPTION ELONGATION FROM RNA POLYMERASE II PROMOTER%GOBP%GO:0034243	0.2807559	0.8454569	27	0.95280004	3447
N-GLYCAN ANTENNAE ELONGATION%REACTOME DATABASE ID RELEASE 75%975577	0.3093466	0.84480613	15	0.9528036	4976
RIBONUCLEOSIDE MONOPHOSPHATE BIOSYNTHETIC PROCESS%GOBP%GO:0009156	0.25099924	0.84675205	77	0.95280546	10263
IL-5 SIGNALING PATHWAY%WIKIPATHWAYS_20210110%WP127%HOMO SAPIENS	0.2679301	0.8273802	30	0.9528089	11091
HSP90 CHAPERONE CYCLE FOR SHRS%REACTOME%R-HSA-3371497.4	0.26598278	0.8309148	36	0.9528164	10397
HALLMARK_FATTY_ACID_METABOLISM%MSIGDB_C2%HALLMARK_FATTY_ACID_METABOLISM	0.24170665	0.8284625	101	0.95282346	12644
POTASSIUM ION HOMEOSTASIS%GOBP%GO:0055075	0.28388733	0.8251368	20	0.9528296	12268
RNA 3'-END PROCESSING%GOBP%GO:0031123	0.23633657	0.8236112	122	0.952836	5641
DIGESTIVE SYSTEM PROCESS%GOBP%GO:0022600	0.2681168	0.82791877	27	0.95283645	9383
SIGNALING BY MODERATE KINASE ACTIVITY BRAF MUTANTS%REACTOME DATABASE ID RELEASE 75%6802946	0.26108727	0.8209655	38	0.9528386	4909
REGULATION OF BIOMINERAL TISSUE DEVELOPMENT%GOBP%GO:0070167	0.24952094	0.8230521	60	0.95284295	9803
REGULATION OF EPITHELIAL TO MESENCHYMAL TRANSITION%GOBP%GO:0010717	0.24541545	0.8274869	80	0.95285326	9065
PID_REELIN_PATHWAY%MSIGDB_C2%PID_REELIN_PATHWAY	0.27959466	0.8249659	22	0.95286465	4378
RHO GTPASES ACTIVATE ROCKS%REACTOME DATABASE ID RELEASE 75%5627117	0.29605573	0.84658587	18	0.95286584	3322
REGULATION OF HISTONE MODIFICATION%GOBP%GO:0031056	0.2385076	0.81827873	101	0.9528707	6277
OUTFLOW TRACT SEPTUM MORPHOGENESIS%GOBP%GO:0003148	0.2766726	0.8222174	24	0.95287085	7728
PHOSPHATIDYLINOSITOL DEPHOSPHORYLATION%GOBP%GO:0046856	0.27708846	0.8242688	23	0.95288336	10243
ALPHA 6 BETA 4 SIGNALING PATHWAY%WIKIPATHWAYS_20210110%WP244%HOMO SAPIENS	0.2729077	0.8306009	26	0.95288646	10110
BIOCARTA_IGF1_PATHWAY%MSIGDB_C2%BIOCARTA_IGF1_PATHWAY	0.2879353	0.8210649	17	0.9528961	4020
MAMMALIAN DISORDER OF SEXUAL DEVELOPMENT%WIKIPATHWAYS_20210110%WP4842%HOMO SAPIENS	0.29824898	0.85921127	18	0.9528967	7542
TRANSLATIONAL TERMINATION%GOBP%GO:0006415	0.2398497	0.8237075	96	0.952898	6216
REGULATION OF DOUBLE-STRAND BREAK REPAIR%GOBP%GO:2000779	0.24630667	0.8375169	81	0.95289886	11802
PID_TRKR_PATHWAY%MSIGDB_C2%PID_TRKR_PATHWAY	0.263206	0.8443559	47	0.9529001	11091
CELLULAR RESPONSE TO BMP STIMULUS%GOBP%GO:0071773	0.24656756	0.8280063	73	0.95290154	6318
NEGATIVE REGULATION OF VASCULATURE DEVELOPMENT%GOBP%GO:1901343	0.24850093	0.84421426	94	0.95290285	13549
75%168643	0.25380623	0.82398	47	0.95290416	10344
PRION DISEASE PATHWAY%WIKIPATHWAYS_20210110%WP3995%HOMO SAPIENS	0.27839	0.8252259	24	0.952905	9509
ENDOCHONDRAL BONE MORPHOGENESIS%GOBP%GO:0060350	0.29056627	0.8277274	18	0.95290506	6684
VESICLE DOCKING%GOBP%GO:0048278	0.25295275	0.8198149	48	0.9529052	14606
SENSORY SYSTEM DEVELOPMENT%GOBP%GO:0048880	0.230822	0.82328	172	0.9529061	8958
REGULATION OF ALCOHOL BIOSYNTHETIC PROCESS%GOBP%GO:1902930	0.24942303	0.82620543	58	0.952907	5114
NEGATIVE REGULATION OF BLOOD VESSEL MORPHOGENESIS%GOBP%GO:2000181	0.24356417	0.82411104	85	0.9529073	8463
STEROID HORMONE MEDIATED SIGNALING PATHWAY%GOBP%GO:0043401	0.25707147	0.82273483	42	0.9529081	8165
PURINE NUCLEOBASE METABOLIC PROCESS%GOBP%GO:0006144	0.3033756	0.828543	15	0.95291287	11012
NEGATIVE REGULATION OF CHROMATIN ORGANIZATION%GOBP%GO:1905268	0.26098916	0.8372418	43	0.95291734	13016
REGULATION OF NECROTIC CELL DEATH%GOBP%GO:0010939	0.2997289	0.836976	17	0.9529212	4145
POSITIVE REGULATION OF COLD-INDUCED THERMOGENESIS%GOBP%GO:0120162	0.24440233	0.8281272	82	0.95292276	8830
TRANSFERRIN ENDOCYTOSIS AND RECYCLING%REACTOME DATABASE ID RELEASE 75%917977	0.2656667	0.82548827	30	0.95292443	6556
POSITIVE REGULATION OF CD4-POSITIVE, ALPHA-BETA T CELL DIFFERENTIATION%GOBP%GO:0043372	0.29793954	0.83124465	17	0.95293313	3567
POSITIVE REGULATION OF CALCIUM ION TRANSPORT INTO CYTOSOL%GOBP%GO:0010524	0.27252677	0.84549516	32	0.95295024	10960
POSITIVE REGULATION OF TRANSCRIPTION REGULATORY REGION DNA BINDING%GOBP%GO:2000679	0.5047734	1.4636445	21	0.95295125	6684
PID_AVB3_INTEGRIN_PATHWAY%MSIGDB_C2%PID_AVB3_INTEGRIN_PATHWAY	0.25468796	0.8376136	52	0.9529526	11800
MITOTIC G1 PHASE AND G1 S TRANSITION%REACTOME%R-HSA-453279.4	0.24159007	0.84378785	145	0.9529534	13833
NEGATIVE REGULATION OF CELL PROJECTION ORGANIZATION%GOBP%GO:0031345	0.24292792	0.8373543	114	0.95295423	8411
REGULATION OF COENZYME METABOLIC PROCESS%GOBP%GO:0051196	0.24453348	0.8286428	79	0.9529577	6322
HALLMARK_P53_PATHWAY%MSIGDB_C2%HALLMARK_P53_PATHWAY	0.23512414	0.83095634	164	0.95296204	6397
GLUCONEOGENESIS%REACTOME DATABASE ID RELEASE 75%70263	0.4811855	1.4623483	27	0.95296276	7952
OVERVIEW OF LEUKOCYTE-INTRINSIC HIPPO PATHWAY FUNCTIONS%WIKIPATHWAYS_20210110%WP4542%HOMO SAPIENS	0.28830278	0.83812696	20	0.95296884	11997
KIT RECEPTOR SIGNALING PATHWAY%WIKIPATHWAYS_20210110%WP304%HOMO SAPIENS	0.2553566	0.82959825	51	0.95297134	10095
NEGATIVE REGULATION OF JNK CASCADE%GOBP%GO:0046329	0.2812682	0.8594221	30	0.9529766	12125
INTRAFLAGELLAR TRANSPORT%REACTOME DATABASE ID RELEASE 75%5620924	0.25951755	0.82945085	36	0.9529815	8559
HISTONE H2A UBIQUITINATION%GOBP%GO:0033522	0.2853043	0.818344	21	0.95298165	8413
POSITIVE REGULATION OF CELL DIVISION%GOBP%GO:0051781	0.26540157	0.83134496	33	0.9529887	3358
HIPPO-YAP SIGNALING PATHWAY%WIKIPATHWAYS_20210110%WP4537%HOMO SAPIENS	0.30817646	0.8448193	15	0.9530018	11076
POSITIVE REGULATION OF CYCLIN-DEPENDENT PROTEIN KINASE ACTIVITY%GOBP%GO:1904031	0.2956893	0.84520805	20	0.9530042	6614
CXCR4%IOB%CXCR4	0.25130576	0.83704424	71	0.9530184	4268
TRANSPORT OF THE SLBP DEPENDANT MATURE MRNA%REACTOME DATABASE ID RELEASE 75%159230	0.25911248	0.8211177	33	0.95302725	4994
RAF ACTIVATION%REACTOME DATABASE ID RELEASE 75%5673000	0.26328957	0.81987774	33	0.9530277	4909
LONG-CHAIN FATTY ACID METABOLIC PROCESS%GOBP%GO:0001676	0.25619677	0.8592623	79	0.9530356	13582
MOLTING CYCLE%GOBP%GO:0042303	0.2910257	0.82374287	18	0.953053	7011
NUCLEOSOME ASSEMBLY%REACTOME%R-HSA-774815.1	0.25551888	0.8245686	46	0.9530572	6033
RETROGRADE TRANSPORT AT THE TRANS-GOLGI-NETWORK%REACTOME%R-HSA-6811440.1	0.2534901	0.82429034	49	0.9530601	12072
POSITIVE REGULATION OF SUBSTRATE ADHESION-DEPENDENT CELL SPREADING%GOBP%GO:1900026	0.26568696	0.8222233	31	0.95307165	6992

FOREBRAIN DEVELOPMENT%GOBP%GO:0030900	0.23806494	0.838184	138	0.9530864	9386
REGULATION OF SYNAPSE ORGANIZATION%GOBP%GO:0050807	0.23793313	0.81770736	113	0.9530874	12095
REGULATION OF JUN KINASE ACTIVITY%GOBP%GO:0043506	0.25286946	0.84397393	56	0.95308894	5270
CAMERA-TYPE EYE MORPHOGENESIS%GOBP%GO:0048593	0.256442	0.8463163	56	0.9531032	9716
REGULATION OF SODIUM ION TRANSMEMBRANE TRANSPORT%GOBP%GO:1902305	0.25156802	0.82550913	54	0.9531044	6080
B CELL DIFFERENTIATION%GOBP%GO:0030183	0.2616952	0.84383017	43	0.95310646	4466
ZINC ION TRANSMEMBRANE TRANSPORT%GOBP%GO:0071577	0.30290997	0.8379123	16	0.95310646	8975
REGULATION OF NECROPTOTIC CELL DEATH%REACTOME%R-HSA-5675482.4	0.27910525	0.8455279	27	0.9531169	9054
TGF_BETA_RECEPTOR%IOB%TGF_BETA_RECEPTOR	0.23823684	0.8376444	143	0.953118	10507
RNA MODIFICATION%GOBP%GO:0009451	0.24138094	0.8450164	140	0.9531196	9322
EXCITATORY POSTSYNAPTIC POTENTIAL%GOBP%GO:0060079	0.30058825	0.8456534	17	0.95312816	9137
CELLULAR RESPONSE TO ACID CHEMICAL%GOBP%GO:0071229	0.23925978	0.8259211	101	0.9531292	6970
GMCSF-MEDIATED SIGNALING EVENTS%PATHWAY INTERACTION DATABASE NCI-NATURE CURATED DATA%GMCSF-MEDIATED SIGNALING EVENTS	0.2704802	0.83098274	31	0.9531377	13502
TRNA EXPORT FROM NUCLEUS%GOBP%GO:0006409	0.25915045	0.81837547	34	0.953139	4994
CHROMATIN ASSEMBLY%GOBP%GO:0031497	0.24582694	0.83593506	90	0.9531435	8824
SYNAPTIC TRANSMISSION, CHOLINERGIC%GOBP%GO:0007271	0.28758234	0.8202216	17	0.95314384	5169
VESICLE-MEDIATED TRANSPORT TO THE PLASMA MEMBRANE%GOBP%GO:0098876	0.2492095	0.83645236	73	0.9531593	11660
DETERMINATION OF HEART LEFT/RIGHT ASYMMETRY%GOBP%GO:0061371	0.26451343	0.8286433	38	0.95317113	7849
HAIR CYCLE%GOBP%GO:0042633	0.2910257	0.825601	18	0.9531787	7011
SYNTHESIS OF PC%REACTOME DATABASE ID RELEASE 75%1483191	0.27502328	0.83604074	28	0.9531852	10931
REGULATION OF INTERLEUKIN-1 BETA PRODUCTION%GOBP%GO:0032651	0.25486436	0.8313534	45	0.95319283	6121
POSITIVE REGULATION OF DNA METABOLIC PROCESS%GOBP%GO:0051054	0.22988653	0.8222827	201	0.9531941	8968
ACETYLCHOLINE NEUROTRANSMITTER RELEASE CYCLE%REACTOME DATABASE ID RELEASE 75%264642	0.30422997	0.84482515	16	0.9532097	11141
SYNAPTIC VESICLE CYTOSKELETAL TRANSPORT%GOBP%GO:0099514	0.29372555	0.8198849	15	0.95322746	11957
ADAPTIVE IMMUNE RESPONSE%GOBP%GO:0002250	0.42360765	1.4079522	67	0.9532314	6528
REGULATION OF FATTY ACID METABOLIC PROCESS%GOBP%GO:0019217	0.24467137	0.8177422	65	0.95324636	10931
PID_HDAC_CLASSII_PATHWAY%MSIGDB_C2%PID_HDAC_CLASSII_PATHWAY	0.28480104	0.84584355	24	0.9532494	10466
REGULATION OF CELLULAR EXTRAVASATION%GOBP%GO:0002691	0.28884986	0.8457085	20	0.95325375	11269
TSH%NETPATH%TSH	0.24735719	0.82429844	63	0.9532589	10378
MTORC1-MEDIATED SIGNALLING%REACTOME%R-HSA-166208.3	0.28053054	0.8361216	24	0.9532668	2347
SAPIENS	0.30216917	0.8199792	15	0.95329887	4780
PID_NOTCH_PATHWAY%MSIGDB_C2%PID_NOTCH_PATHWAY	0.25322622	0.81840575	42	0.953306	6496
PARKINSON DISEASE%PANTHER PATHWAY%P00049	0.24726318	0.83766145	86	0.9533089	9093
CEREBELLAR CORTEX DEVELOPMENT%GOBP%GO:0021695	0.2810737	0.81911814	19	0.9533166	162
VITAMIN TRANSPORT%GOBP%GO:0051180	0.2759877	0.83622104	26	0.9533214	13087
NEGATIVE REGULATION OF DOUBLE-STRAND BREAK REPAIR%GOBP%GO:2000780	0.26643434	0.82563186	31	0.9533467	9018
REGULATION OF ALPHA-BETA T CELL DIFFERENTIATION%GOBP%GO:0046637	0.26846322	0.8459079	34	0.953356	3567
CELLULAR AMINO ACID METABOLIC PROCESS%GOBP%GO:0006520	0.2359458	0.8460332	236	0.9533608	4806
DNA REPLICATION%WIKIPATHWAYS_20210110%WP466%HOMO SAPIENS	0.27114323	0.8177956	28	0.9533749	11716
REGULATION OF PROTEIN POLYUBIQUITINATION%GOBP%GO:1902914	0.29511005	0.8286448	17	0.95338386	6480
NEGATIVE REGULATION OF RESPONSE TO DRUG%GOBP%GO:2001024	0.29279578	0.83150566	18	0.95338523	1532
REGULATION OF EXTRACELLULAR MATRIX ORGANIZATION%GOBP%GO:1903053	0.2645668	0.8313654	37	0.95338804	8580
REGULATION OF OXIDATIVE STRESS-INDUCED NEURON DEATH%GOBP%GO:1903203	0.29597685	0.8186314	16	0.95338935	6322
PID_PDGFRB_PATHWAY%MSIGDB_C2%PID_PDGFRB_PATHWAY	0.2415744	0.8287761	89	0.9533953	5884
IL4-MEDIATED SIGNALING EVENTS%PATHWAY INTERACTION DATABASE NCI-NATURE CURATED DATA%IL4-MEDIATED SIGNALING EVENTS	0.25980392	0.83563423	45	0.95340836	8782
HALLMARK_SPERMATOGENESIS%MSIGDB_C2%HALLMARK_SPERMATOGENESIS	0.24287878	0.8191963	70	0.9534179	9573
AGING%GOBP%GO:0007568	0.24539363	0.81845576	65	0.95343643	12347
GLUTAMINE FAMILY AMINO ACID CATABOLIC PROCESS%GOBP%GO:0009065	0.2928576	0.8168695	15	0.95348984	4132
ENERGY METABOLISM%WIKIPATHWAYS_20210110%WP1541%HOMO SAPIENS	0.2549321	0.8192724	41	0.9535196	8383
POSITIVE REGULATION OF TUMOR NECROSIS FACTOR PRODUCTION%GOBP%GO:0032760	0.2599443	0.831818	45	0.953525	6569
DE NOVO PURINE BIOSYNTHESIS%PANTHER PATHWAY%P02738	0.263855	0.8187954	29	0.9535544	6472
LONG-CHAIN FATTY-ACYL-COA METABOLIC PROCESS%GOBP%GO:0035336	0.27417797	0.81711453	21	0.9535553	8103
POST-TRANSLATIONAL PROTEIN PHOSPHORYLATION%REACTOME DATABASE ID RELEASE 75%8957275	0.24471599	0.83151835	87	0.95357907	11925
ALPHA-AMINO ACID METABOLIC PROCESS%GOBP%GO:1901605	0.23128861	0.8186396	164	0.95358604	4981
VISUAL SYSTEM DEVELOPMENT%GOBP%GO:0150063	0.23134223	0.81721216	166	0.9536188	10969
CHROMATIN REMODELING%GOBP%GO:0006338	0.23511627	0.81617326	145	0.9536807	5762
HISTONE H2A ACETYLATION%GOBP%GO:0043968	0.28958893	0.8168804	17	0.953684	8935
REGULATION OF CELLULAR RESPIRATION%GOBP%GO:0043457	0.2848426	0.8163006	19	0.95369923	11618
RESPONSE TO MOLECULE OF BACTERIAL ORIGIN%GOBP%GO:0002237	0.23784226	0.83157796	132	0.9537045	8196
SIGNALING DOWNSTREAM OF RAS MUTANTS%REACTOME DATABASE ID RELEASE 75%9649948	0.26108727	0.8318352	38	0.9537115	4909
NEGATIVE REGULATION OF NMDA RECEPTOR-MEDIATED NEURONAL TRANSMISSION%REACTOME%R-HSA-9617324.1	0.49013603	1.4251618	20	0.95371354	6250
REGULATION OF GRANULOCYTE CHEMOTAXIS%GOBP%GO:0071622	0.26614532	0.81642467	33	0.95372844	11417
DEPOSITION OF NEW CENPA-CONTAINING NUCLEOSOMES AT THE CENTROMERE%REACTOME DATABASE ID RELEASE 75%606279	0.25551888	0.81598943	46	0.95373803	6033
REGULATION OF CYTOKINE-MEDIATED SIGNALING PATHWAY%GOBP%GO:0001959	0.23418447	0.81650066	121	0.9538278	12211
VXPX CARGO-TARGETING TO CILIUM%REACTOME%R-HSA-5620916.2	0.28807506	0.83188593	19	0.95384985	12947
REGULATION OF ALTERNATIVE MRNA SPLICING, VIA SPLICEOSOME%GOBP%GO:0000381	0.2433988	0.81568277	68	0.9539664	4224
PID_RETINOIC_ACID_PATHWAY%MSIGDB_C2%PID_RETINOIC_ACID_PATHWAY	0.48555943	1.4217243	21	0.9540107	7004
REGULATION OF ENDOTHELIAL CELL PROLIFERATION%GOBP%GO:0001936	0.24249864	0.8319001	108	0.9540418	9804
NEGATIVE REGULATION OF CHROMOSOME SEGREGATION%GOBP%GO:0051985	0.4626493	1.4017742	29	0.9540635	8983
POSITIVE REGULATION OF CYTOKINE PRODUCTION INVOLVED IN IMMUNE RESPONSE%GOBP%GO:0002720	0.2635151	0.8151495	34	0.95413065	4842
POSITIVE REGULATION OF RELEASE OF SEQUESTERED CALCIUM ION INTO CYTOSOL%GOBP%GO:0051281	0.28851414	0.8350546	22	0.9541572	16496
PID_TAP63_PATHWAY%MSIGDB_C2%PID_TAP63_PATHWAY	0.4915888	1.5536175	39	0.9541619	3242
G13 SIGNALING PATHWAY%WIKIPATHWAYS_20210110%WP524%HOMO SAPIENS	0.4783409	1.4584554	27	0.95416534	5640
RHO PROTEIN SIGNAL TRANSDUCTION%GOBP%GO:0007266	0.43478432	1.424591	52	0.9541706	7265
D-<I>MYO< I>-INOSITOL (1,4,5)-TRISPHOSPHATE BIOSYNTHESIS%HUMANCYC%PWY-6351	0.48164356	1.4641228	25	0.95417064	6089
RESPONSE TO ISCHEMIA%GOBP%GO:0002931	0.49694115	1.4666646	21	0.95417684	3300
PHOSPHATIDYLCHOLINE METABOLIC PROCESS%GOBP%GO:0046470	0.24861452	0.8319278	63	0.95421284	12587
RESPONSE TO CADMIUM ION%GOBP%GO:0046686	0.27248996	0.8152356	24	0.95421463	2710
POSITIVE REGULATION OF ALPHA-BETA T CELL DIFFERENTIATION%GOBP%GO:0046638	0.27721664	0.8320237	25	0.95427185	3567
POSITIVE REGULATION OF CARBOHYDRATE METABOLIC PROCESS%GOBP%GO:0045913	0.2569773	0.83255255	48	0.9542766	11474
GANGLIOSIDE BIOSYNTHETIC PROCESS%GOBP%GO:0001574	0.3067848	0.8324209	15	0.9542888	1313
MELATONIN METABOLISM AND EFFECTS%WIKIPATHWAYS_20210110%WP3298%HOMO SAPIENS	0.48632005	1.4596591	26	0.95438147	5632
CENTRIOLE ASSEMBLY%GOBP%GO:0098534	0.26593277	0.8146779	27	0.9543919	5411
CXCR3-MEDIATED SIGNALING EVENTS%PATHWAY INTERACTION DATABASE NCI-NATURE CURATED DATA%CXCR3-MEDIATED SIGNALING EVENTS	0.27046415	0.83476144	32	0.95441353	15839
NONALCOHOLIC FATTY LIVER DISEASE%WIKIPATHWAYS_20210110%WP4396%HOMO SAPIENS	0.40946582	1.42217	121	0.95441556	4417
PLASMA LIPOPROTEIN REMODELING%REACTOME DATABASE ID RELEASE 75%8963899	0.290272	0.8152363	17	0.9544243	9399
MICROTUBULE ORGANIZING CENTER LOCALIZATION%GOBP%GO:0061842	0.2845217	0.8147725	20	0.9544673	7557
REGULATION OF TELOMERE MAINTENANCE VIA TELOMERASE%GOBP%GO:0032210	0.25271404	0.8325622	51	0.95447624	9660
BIOCARTA_NGF_PATHWAY%MSIGDB_C2%BIOCARTA_NGF_PATHWAY	0.29989716	0.8320298	16	0.95447886	4064
NEGATIVE REGULATION OF CHROMOSOME SEPARATION%GOBP%GO:1905819	0.48549914	1.4255534	24	0.9544816	2966
PEPTIDYL-SERINE MODIFICATION%GOBP%GO:0018209	0.23511131	0.8321544	170	0.95449704	7013
POSITIVE REGULATION OF VASCULAR ENDOTHELIAL GROWTH FACTOR PRODUCTION%GOBP%GO:0010575	0.2724174	0.8094383	21	0.95449936	10576
NEGATIVE REGULATION OF GENE EXPRESSION, EPIGENETIC%GOBP%GO:0045814	0.24294782	0.80955684	66	0.954539	9436
PROTEIN TARGETING TO ER%GOBP%GO:0045047	0.23587275	0.8144346	106	0.95454127	8277
NUCLEOTIDE-SUGAR BIOSYNTHETIC PROCESS%GOBP%GO:0009226	0.48227555	1.4086682	21	0.95455235	5673
RECEPTOR LOCALIZATION TO SYNAPSE%GOBP%GO:0097120	0.26695538	0.80896235	27	0.9545617	13235
SCAVENGING BY CLASS A RECEPTORS%REACTOME DATABASE ID RELEASE 75%3000480	0.29558715	0.81427777	15	0.95456946	8637
SYNAPTIC VESICLE TRANSPORT ALONG MICROTUBULE%GOBP%GO:0099517	0.29372555	0.80274415	15	0.95457464	11957
IL12-MEDIATED SIGNALING EVENTS%PATHWAY INTERACTION DATABASE NCI-NATURE CURATED DATA%IL12-MEDIATED SIGNALING EVENTS	0.25483015	0.80965006	39	0.95461595	9096
LYSOSOME LOCALIZATION%GOBP%GO:0032418	0.2551987	0.7998691	34	0.95461667	5925
NEGATIVE REGULATION OF ANGIOGENESIS%GOBP%GO:0016525	0.24156834	0.8137914	83	0.9546639	13549
PID_LKB1_PATHWAY%MSIGDB_C2%PID_LKB1_PATHWAY	0.26842892	0.83257437	33	0.95467323	9402
REGULATION OF DENDRITE MORPHOGENESIS%GOBP%GO:0048814	0.24534826	0.8091632	59	0.9546912	9742
REGULATION OF CHROMATIN ORGANIZATION%GOBP%GO:1902275	0.23129204	0.8108718	140	0.9547041	11353
DEVELOPMENTAL CELL GROWTH%GOBP%GO:0048588	0.25862524	0.83430517	44	0.9547153	2926
D-<I>MYO< I>-INOSITOL (1,3,4)-TRISPHOSPHATE BIOSYNTHESIS%HUMANCYC%PWY-6364	0.29543796	0.8138886	15	0.9547274	11114
POSITIVE REGULATION OF SECRETION%GOBP%GO:0051047	0.22789784	0.8086908	177	0.9547296	11558
EPHB-MEDIATED FORWARD SIGNALING%REACTOME DATABASE ID RELEASE 75%3928662	0.26048315	0.813299	33	0.9547332	6134
NEGATIVE REGULATION OF COLD-INDUCED THERMOGENESIS%GOBP%GO:0120163	0.24859676	0.8027775	43	0.95473355	15798
NEGATIVE REGULATION OF NEURON DIFFERENTIATION%GOBP%GO:0045665	0.23723747	0.8140193	112	0.95474553	9395
MAPK TARGETS NUCLEAR EVENTS MEDIATED BY MAP KINASES%REACTOME DATABASE ID RELEASE 75%450282	0.26620898	0.83265907	31	0.95475346	10539
IL2%NETPATH%IL2	0.2457986	0.80897015	60	0.95475984	4268
CIRCADIAN REGULATION OF GENE EXPRESSION%GOBP%GO:0032922	0.24520288	0.8096873	61	0.95476395	7491
PROTEIN-CONTAINING COMPLEX REMODELING%GOBP%GO:0034367	0.27709755	0.80981046	21	0.95478976	2531
N-GLYCAN PROCESSING%GOBP%GO:0006491	0.2807358	0.8109494	19	0.9547937	5966
POSITIVE REGULATION OF EPIDERMIS DEVELOPMENT%GOBP%GO:0045684	0.27283978	0.79987687	23	0.95481175	6684
GASTRIC CANCER NETWORK 1%WIKIPATHWAYS_20210110%WP2361%HOMO SAPIENS	0.27631292	0.81121385	21	0.9548137	10555
REGULATION OF COLD-INDUCED THERMOGENESIS%GOBP%GO:0120161	0.23244229	0.8018172	126	0.9548351	8541
RESPONSE TO BMP%GOBP%GO:0071772	0.24656756	0.8343647	73	0.9548352	6318
REGULATION OF FATTY ACID OXIDATION%GOBP%GO:0046320	0.27435723	0.81336695	24	0.9548441	11345
INSULIN RECEPTOR RECYCLING%REACTOME%R-HSA-77387.3	0.26497415	0.8099109	25	0.9548503	8331
NIK/NF-KAPPAB SIGNALING%GOBP%GO:0038061	0.24288721	0.81277066	69	0.9548532	9017
MEMBRANE ASSEMBLY%GOBP%GO:0071709	0.26290792	0.8084496	30	0.95486724	5740
REGULATION OF PROTEIN LOCALIZATION TO CELL SURFACE%GOBP%GO:2000008	0.4800764	1.4768616	29	0.95488703	6320
PEPTIDE SECRETION%GOBP%GO:0002790	0.24220479	0.81288445	72	0.9548878	11458
NEGATIVE REGULATION OF MUSCLE ORGAN DEVELOPMENT%GOBP%GO:0048635	0.286499	0.8327016	22	0.9548982	2293
GLUCOSE HOMEOSTASIS%GOBP%GO:0042593	0.23311357	0.8005663	93	0.95490235	12261

ANION HOMEOSTASIS%GOBP%GO:0055081	0.25263128	0.80070204	37	0.9549136	2682
DENDRITE DEVELOPMENT%GOBP%GO:0016358	0.2439771	0.81058043	63	0.9549156	9141
KETONE BIOSYNTHETIC PROCESS%GOBP%GO:0042181	0.27472153	0.80278677	20	0.954928	10268
SENSORY ORGAN DEVELOPMENT%GOBP%GO:0007423	0.22697824	0.81170833	216	0.95495033	9716
OXIDATIVE STRESS RESPONSE%PANTHER PATHWAY%P00046	0.25391215	0.81126446	47	0.95495045	12211
NEGATIVE REGULATION OF I-KAPPAB KINASE/NF-KAPPAB SIGNALING%GOBP%GO:0043124	0.26046723	0.8102792	34	0.9549511	14598
NEGATIVE REGULATION OF HISTONE MODIFICATION%GOBP%GO:0031057	0.25978953	0.7999103	32	0.9549689	12957
INSULIN SIGNALING%WIKIPATHWAYS_20210110%WP481%HOMO SAPIENS	0.23943457	0.8340016	124	0.95498085	15254
ESTABLISHMENT OF PROTEIN LOCALIZATION TO ENDOPLASMIC RETICULUM%GOBP%GO:0072599	0.23151392	0.8013975	110	0.9549846	8277
TETRAPYRROLE METABOLIC PROCESS%GOBP%GO:0033013	0.2503996	0.81010544	42	0.95498866	3593
PID_CDC42_PATHWAY%MSIGDB_C2%PID_CDC42_PATHWAY	0.24933442	0.810958	52	0.9549914	10725
POSITIVE REGULATION OF DNA REPLICATION%GOBP%GO:0045740	0.26710185	0.81038344	26	0.95500284	9444
ANTEROGRADE SYNAPTIC VESICLE TRANSPORT%GOBP%GO:0048490	0.29372555	0.8099438	15	0.95501554	11957
PID_ATF2_PATHWAY%MSIGDB_C2%PID_ATF2_PATHWAY	0.25809205	0.83289486	47	0.9550219	11874
HEAD AND NECK SQUAMOUS CELL CARCINOMA%WIKIPATHWAYS_20210110%WP4674%HOMO SAPIENS	0.24243951	0.8018278	60	0.95502615	11091
PEROXISOMAL PROTEIN IMPORT%REACTOME%R-HSA-9033241.3	0.24643187	0.8015192	56	0.9550287	6488
REGULATION OF AMPA RECEPTOR ACTIVITY%GOBP%GO:2000311	0.27230048	0.8003141	24	0.9550364	12135
BRANCHING INVOLVED IN URETERIC BUD MORPHOGENESIS%GOBP%GO:0001658	0.2862397	0.8129282	18	0.95504177	5634
POSITIVE REGULATION OF INTERFERON-BETA PRODUCTION%GOBP%GO:0032728	0.26728734	0.80000496	26	0.95504385	4889
FAT-SOLUBLE VITAMIN METABOLIC PROCESS%GOBP%GO:0006775	0.2848436	0.8133719	20	0.95504767	14483
MEMBRANE BIOGENESIS%GOBP%GO:0044091	0.26742825	0.833015	33	0.9550535	5740
CELLULAR MODIFIED AMINO ACID CATABOLIC PROCESS%GOBP%GO:0042219	0.2753618	0.8001409	19	0.9550614	4827
CARBOHYDRATE HOMEOSTASIS%GOBP%GO:0033500	0.23188487	0.8011928	95	0.9550664	12261
FACTOR NETWORK	0.4266597	1.4230514	59	0.9550735	7601
ESTABLISHMENT OF PROTEIN LOCALIZATION TO MITOCHONDRIAL MEMBRANE%GOBP%GO:0090151	0.2943144	0.8007154	15	0.9551029	12767
PROTEIN TARGETING TO PEROXISOME%GOBP%GO:0006625	0.25405	0.8327061	63	0.95510674	7116
REGULATION OF MUSCLE CELL APOPTOTIC PROCESS%GOBP%GO:0010660	0.26537883	0.8081339	26	0.9551129	9786
ACTIVATION OF MAPK ACTIVITY%GOBP%GO:0000187	0.23237018	0.80280405	113	0.955118	10710
SIGNALING BY BRAF AND RAF FUSIONS%REACTOME DATABASE ID RELEASE 75%6802952	0.24663138	0.8117264	57	0.95513433	4202
SRP-DEPENDENT COTRANSLATIONAL PROTEIN TARGETING TO MEMBRANE%REACTOME DATABASE ID RELEASE 75%1799339	0.23590304	0.8112733	109	0.9551474	8277
ATR SIGNALING PATHWAY%PATHWAY INTERACTION DATABASE NCI-NATURE CURATED DATA%ATR SIGNALING PATHWAY	0.25352386	0.81141186	39	0.9551682	11716
POSITIVE REGULATION OF PROTEIN TARGETING TO MITOCHONDRION%GOBP%GO:1903955	0.26940408	0.83375794	30	0.95516974	9849
NEGATIVE REGULATION OF JAK-STAT CASCADE%GOBP%GO:0046426	0.2824637	0.80290896	17	0.9551762	3153
PEPTIDYL-TYROSINE MODIFICATION%GOBP%GO:0018212	0.24656048	0.81182927	58	0.95519596	10403
RESPONSE TO PROGESTERONE%GOBP%GO:0032570	0.29015768	0.8031937	16	0.95519984	10957
REGULATION OF CHOLESTEROL BIOSYNTHESIS BY SREBP (SREBF)%REACTOME%R-HSA-1655829.2	0.24407579	0.8019953	52	0.9552173	5114
ANDROGEN RECEPTOR SIGNALING PATHWAY%WIKIPATHWAYS_20210110%WP138%HOMO SAPIENS	0.24244346	0.80184084	64	0.9552184	10483
KSRP (KHSRP) BINDS AND DESTABILIZES MRNA%REACTOME%R-HSA-450604.2	0.2871981	0.80076796	17	0.9552394	16930
CRMPS IN SEMA3A SIGNALING%REACTOME%R-HSA-399956.1	0.29965776	0.8330344	16	0.9552406	1871
PRESYNAPSE ORGANIZATION%GOBP%GO:0099172	0.2801588	0.80745226	19	0.9552471	15104
POSITIVE REGULATION OF KIDNEY DEVELOPMENT%GOBP%GO:0090184	0.27066928	0.81231636	27	0.9553042	7094
CASPASE ACTIVATION VIA EXTRINSIC APOPTOTIC SIGNALLING PATHWAY%REACTOME DATABASE ID RELEASE 75%5357769	0.26823783	0.8029497	24	0.95533323	10616
BLADDER CANCER%WIKIPATHWAYS_20210110%WP2828%HOMO SAPIENS	0.2629074	0.8120076	32	0.9553436	11091
TRNA-CONTAINING RIBONUCLEOPROTEIN COMPLEX EXPORT FROM NUCLEUS%GOBP%GO:0071431	0.25915045	0.81186396	34	0.95534736	4994
RECOGNITION OF DNA DAMAGE BY PCNA-CONTAINING REPLICATION COMPLEX%REACTOME DATABASE ID RELEASE 75%110314	0.26135546	0.8121394	30	0.95535797	4361
AUTOPHAGOSOME ASSEMBLY%GOBP%GO:0000045	0.24591579	0.8032144	51	0.9553775	6814
REGULATION OF GLIAL CELL DIFFERENTIATION%GOBP%GO:0045685	0.2628424	0.80200815	31	0.9554074	4466
LIPID PHOSPHORYLATION%GOBP%GO:0046834	0.24709532	0.80347735	47	0.9554215	11642
JNK (C-JUN KINASES) PHOSPHORYLATION AND ACTIVATION MEDIATED BY ACTIVATED HUMAN TAK1%REACTOME%R-HSA-450321.2	0.28920197	0.8074752	17	0.95542157	7967
DISEASES ASSOCIATED WITH GLYCOSAMINOGLYCAN METABOLISM%REACTOME DATABASE ID RELEASE 75%3560782	0.24690399	0.8007685	40	0.9554451	5207
JNK CASCADE%GOBP%GO:0007254	0.25037503	0.83304036	63	0.95545024	2983
PID_PDGFRA_PATHWAY%MSIGDB_C2%PID_PDGFRA_PATHWAY	0.29943004	0.8334111	17	0.9555127	4020
MRNA CATABOLIC PROCESS%GOBP%GO:0006402	0.22572859	0.8075358	205	0.95553637	5978
POSITIVE REGULATION OF DNA BIOSYNTHETIC PROCESS%GOBP%GO:2000573	0.24276955	0.8032405	62	0.9555518	12868
SIGNALING BY INSULIN RECEPTOR%REACTOME DATABASE ID RELEASE 75%74752	0.23879308	0.80396485	59	0.95557064	8331
POSITIVE REGULATION OF INTERLEUKIN-8 PRODUCTION%GOBP%GO:0032757	0.25219834	0.80364764	39	0.9556057	7226
CELL CYCLE G1/S PHASE TRANSITION%GOBP%GO:0044843	0.23238026	0.8034925	110	0.9556065	14204
BIOCARTA_NTHI_PATHWAY%MSIGDB_C2%BIOCARTA_NTHI_PATHWAY	0.52413297	1.4712114	18	0.9556306	3663
REGULATION OF CELL JUNCTION ASSEMBLY%GOBP%GO:1901888	0.24554886	0.83306116	79	0.9556333	3536
ORGANIC HYDROXY COMPOUND BIOSYNTHETIC PROCESS%GOBP%GO:1901617	0.23392516	0.80376875	115	0.955635	6438
TRANSPORT OF VITAMINS, NUCLEOSIDES, AND RELATED MOLECULES%REACTOME%R-HSA-425397.3	0.25668448	0.8075954	35	0.95566297	12915
REGULATION OF INTRINSIC APOPTOTIC SIGNALING PATHWAY BY P53 CLASS MEDIATOR%GOBP%GO:1902253	0.27164757	0.7989466	21	0.9556862	4867
REGULATION OF BROWN FAT CELL DIFFERENTIATION%GOBP%GO:0090335	0.29008228	0.806683	17	0.9557096	7911
REGULATION OF NEUTROPHIL CHEMOTAXIS%GOBP%GO:0090022	0.28192347	0.803997	20	0.95572996	10373
CHROMATIN SILENCING%GOBP%GO:0006342	0.24676596	0.8063695	48	0.9557325	9436
SIGNALLING TO RAS%REACTOME%R-HSA-167044.4	0.2874321	0.8051669	17	0.955756	12972
ENDOCRINE SYSTEM DEVELOPMENT%GOBP%GO:0035270	0.2609255	0.83309644	46	0.95579845	14204
STRIATED MUSCLE CONTRACTION PATHWAY%WIKIPATHWAYS_20210110%WP383%HOMO SAPIENS	0.29051042	0.8052648	16	0.95582545	14149
VESICLE DOCKING INVOLVED IN EXOCYTOSIS%GOBP%GO:0006904	0.2607359	0.80452317	30	0.9558297	11596
RAB REGULATION OF TRAFFICKING%REACTOME DATABASE ID RELEASE 75%9007101	0.23281777	0.8067281	118	0.9558533	6995
RETINA MORPHOGENESIS IN CAMERA-TYPE EYE%GOBP%GO:0060042	0.2681343	0.80685145	28	0.9558877	7094
SOMATIC STEM CELL POPULATION MAINTENANCE%GOBP%GO:0035019	0.2578914	0.80402607	34	0.95589113	5533
G1/S TRANSITION OF MITOTIC CELL CYCLE%GOBP%GO:0000082	0.23528175	0.8063857	108	0.9559189	14204
RESPONSE TO NICOTINE%GOBP%GO:0035094	0.29205337	0.80533236	15	0.95593494	7004
REGULATION OF MAST CELL ACTIVATION INVOLVED IN IMMUNE RESPONSE%GOBP%GO:0033006	0.28118464	0.798593	18	0.9559479	14775
PID_TXA2PATHWAY%MSIGDB_C2%PID_TXA2PATHWAY	0.25877658	0.8045741	33	0.955964	10110
REGULATION OF BONE MINERALIZATION%GOBP%GO:0030500	0.24907371	0.80469507	50	0.9559976	11852
PID_CASPASE_PATHWAY%MSIGDB_C2%PID_CASPASE_PATHWAY	0.2574359	0.80422246	35	0.9560345	4807
TARGET OF RAPAMYCIN (TOR) SIGNALING%WIKIPATHWAYS_20210110%WP1471%HOMO SAPIENS	0.25699857	0.79479593	29	0.95604634	14990
REGULATION OF PEPTIDE SECRETION%GOBP%GO:0002791	0.22603476	0.804055	194	0.9560553	8214
REGULATION OF VASCULAR PERMEABILITY%GOBP%GO:0043114	0.2668724	0.8048027	25	0.9560573	7537
PROTEIN LOCALIZATION TO CHROMOSOME, CENTROMERIC REGION%GOBP%GO:0071459	0.28144982	0.79724	18	0.95607233	7587
ACTIVATION OF MATRIX METALLOPROTEINASES%REACTOME DATABASE ID RELEASE 75%1592389	0.27478865	0.7958297	20	0.9560857	3686
RESPONSE TO COPPER ION%GOBP%GO:0046688	0.27105924	0.79535675	21	0.9560994	2338
TRANSLOCATION OF SLC2A4 (GLUT4) TO THE PLASMA MEMBRANE%REACTOME DATABASE ID RELEASE 75%1445148	0.24710177	0.8053357	50	0.95613676	14477
CIRCADIAN RHYTHM RELATED GENES%WIKIPATHWAYS_20210110%WP3594%HOMO SAPIENS	0.2295785	0.80547863	145	0.95614034	8479
CHAPERONE COFACTOR-DEPENDENT PROTEIN REFOLDING%GOBP%GO:0051085	0.26326248	0.79592615	26	0.956162	11894
INTRACELLULAR STEROID HORMONE RECEPTOR SIGNALING PATHWAY%GOBP%GO:0030518	0.2531264	0.79484034	38	0.9561953	8165
5HT1 TYPE RECEPTOR MEDIATED SIGNALING PATHWAY%PANTHER PATHWAY%P04373	0.25459522	0.8055689	36	0.9562217	9826
REGULATION OF INTERFERON-BETA PRODUCTION%GOBP%GO:0032648	0.25155336	0.7955589	37	0.95623064	5208
MONONUCLEAR CELL PROLIFERATION%GOBP%GO:0032943	0.4944912	1.4125311	18	0.9562339	6569
SNRNA TRANSCRIPTION BY RNA POLYMERASE II%GOBP%GO:0042795	0.23778027	0.79648364	69	0.9562418	5629
NEGATIVE REGULATION OF DEVELOPMENTAL GROWTH%GOBP%GO:0048640	0.2442313	0.7969418	53	0.9562494	9065
POSITIVE REGULATION OF CHEMOKINE PRODUCTION%GOBP%GO:0032722	0.2598458	0.7953978	32	0.9562499	8102
PID_ALK1_PATHWAY%MSIGDB_C2%PID_ALK1_PATHWAY	0.2762587	0.7972568	20	0.956256	9704
POSITIVE REGULATION OF GRANULOCYTE CHEMOTAXIS%GOBP%GO:0071624	0.28008002	0.79663295	17	0.9562566	10373
NEGATIVE REGULATION OF EMBRYONIC DEVELOPMENT%GOBP%GO:0045992	0.29612452	0.8056784	15	0.95627135	5456
LIPOPOLYSACCHARIDE-MEDIATED SIGNALING PATHWAY%GOBP%GO:0031663	0.29145688	0.7941384	15	0.9562833	1958
SECRETORY GRANULE ORGANIZATION%GOBP%GO:0033363	0.2754623	0.79505295	19	0.95631886	6594
IRON UPTAKE AND TRANSPORT%REACTOME DATABASE ID RELEASE 75%917937	0.24385354	0.79394644	53	0.95634496	3933
NEGATIVE REGULATION OF PLASMA MEMBRANE BOUNDED CELL PROJECTION ASSEMBLY%GOBP%GO:0120033	0.26679808	0.7942365	24	0.9563592	3059
ADULT BEHAVIOR%GOBP%GO:0030534	0.25513574	0.79592717	37	0.95636636	6482
MEMBRANE DEPOLARIZATION DURING CARDIAC MUSCLE CELL ACTION POTENTIAL%GOBP%GO:0086012	0.2800223	0.7948661	17	0.9563681	7006
SPINAL CORD INJURY%WIKIPATHWAYS_20210110%WP2431%HOMO SAPIENS	0.23616143	0.79607457	78	0.9563768	10205
ENTRY INTO HOST%GOBP%GO:0044409	0.4494028	1.4079717	36	0.9563919	6947
FATTY ACID DERIVATIVE METABOLIC PROCESS%GOBP%GO:1901568	0.2298964	0.8057346	116	0.9564016	5288
BIOCARTA_PDGF_PATHWAY%MSIGDB_C2%BIOCARTA_PDGF_PATHWAY	0.27149636	0.79729086	22	0.9564225	4523
PID_MAPK_TRK_PATHWAY%MSIGDB_C2%PID_MAPK_TRK_PATHWAY	0.26422855	0.7961984	28	0.9564235	11306
MYOTUBE DIFFERENTIATION%GOBP%GO:0014902	0.48444784	1.4200039	23	0.9564364	2885
NEGATIVE REGULATION OF REACTIVE OXYGEN SPECIES BIOSYNTHETIC PROCESS%GOBP%GO:1903427	0.27187288	0.7966497	21	0.9564413	4317
POSITIVE REGULATION OF UBIQUITIN-PROTEIN TRANSFERASE ACTIVITY%GOBP%GO:0051443	0.46913064	1.4444658	29	0.9564468	2122
UBIQUINONE BIOSYNTHETIC PROCESS%GOBP%GO:0006744	0.28265807	0.7937003	16	0.9564679	1231
REGULATION OF EXPRESSION OF SLITS AND ROBOS%REACTOME DATABASE ID RELEASE 75%9010553	0.22415738	0.7935425	165	0.9564823	8277
MICROVILLUS ORGANIZATION%GOBP%GO:0032528	0.2906924	0.797386	15	0.95650095	3604
PROTEIN-LIPID COMPLEX SUBUNIT ORGANIZATION%GOBP%GO:0071825	0.25588608	0.7933751	34	0.956504	15041
LEPTIN%IOB%LEPTIN	0.25215915	0.7942763	39	0.956511	14850
NEGATIVE REGULATION OF RESPONSE TO CYTOKINE STIMULUS%GOBP%GO:0060761	0.24696642	0.79798704	45	0.95654494	13762
INSULIN SECRETION%GOBP%GO:0030073	0.28022635	0.7931791	18	0.95655406	14067
REGULATION OF TRANSCRIPTION REGULATORY REGION DNA BINDING%GOBP%GO:2000677	0.44089627	1.4088347	36	0.9565895	5005
POSITIVE REGULATION OF T CELL DIFFERENTIATION%GOBP%GO:0045582	0.24433282	0.79741365	48	0.95666885	4927
ENDODERMAL CELL DIFFERENTIATION%GOBP%GO:0035987	0.2559387	0.7976628	31	0.95676595	12343
ANTIBIOTIC METABOLIC PROCESS%GOBP%GO:0016999	0.23247676	0.7974289	87	0.9568555	1400
NEGATIVE REGULATION OF PEPTIDYL-SERINE PHOSPHORYLATION%GOBP%GO:0033137	0.50973475	1.4676684	20	0.9570188	5004
PID_P53_DOWNSTREAM_PATHWAY%MSIGDB_C2%PID_P53_DOWNSTREAM_PATHWAY	0.40883592	1.4092242	109	0.9570501	5039
GLYCOLYSIS I%HUMANCYC%GLYCOLYSIS	0.4981986	1.411953	18	0.95719486	4362
NEGATIVE REGULATION OF CELL CYCLE ARREST%GOBP%GO:0071157	0.5129269	1.4643652	17	0.95732594	4489
STING PATHWAY IN KAWASAKI-LIKE DISEASE AND COVID-19%WIKIPATHWAYS_20210110%WP4961%HOMO SAPIENS	0.5261216	1.4256505	15	0.95749956	5202

MICROGLIA PATHOGEN PHAGOCYTOSIS PATHWAY%WIKIPATHWAYS_20210110%WP3937%HOMO SAPIENS	0.48212424	1.411471	20	0.95752096	5884
ENDOTHELIAL CELL DEVELOPMENT%GOBP%GO:0001885	0.44563395	1.4193832	37	0.9577332	1510
MICROTUBULE POLYMERIZATION%GOBP%GO:0046785	0.25467607	0.7907052	30	0.95773846	16058
SKELETAL MUSCLE ORGAN DEVELOPMENT%GOBP%GO:0060538	0.24760202	0.79049075	40	0.9578129	10230
REGULATION OF CARBOHYDRATE METABOLIC PROCESS%GOBP%GO:0006109	0.22559266	0.7907157	141	0.9579273	8514
EVENTS	0.25044283	0.7902474	32	0.9579315	2049
PROTEIN DEGLYCOSYLATION%GOBP%GO:0006517	0.26520833	0.79076636	28	0.9580599	6622
FERRIC IRON TRANSPORT%GOBP%GO:0015682	0.25017133	0.79090744	35	0.9580905	8331
DOWNREGULATION OF TGF-BETA RECEPTOR SIGNALING%REACTOME DATABASE ID RELEASE 75%2173788	0.26475015	0.7911374	25	0.95820916	5995
INFLAMMATORY RESPONSE PATHWAY%WIKIPATHWAYS_20210110%WP453%HOMO SAPIENS	0.5123326	1.4402506	17	0.95823014	5691
RESPONSE TO BACTERIUM%GOBP%GO:0009617	0.22224994	0.7912694	211	0.95823467	12495
MTOR SIGNALLING%REACTOME%R-HSA-165159.6	0.24741605	0.7909211	40	0.9582789	2750
ARACHIDONIC ACID METABOLISM%REACTOME%R-HSA-2142753.5	0.24437305	0.7840688	40	0.9583209	4142
GLIOGENESIS%GOBP%GO:0042063	0.23062608	0.7845323	91	0.95833147	6246
NEGATIVE REGULATION OF DNA METABOLIC PROCESS%GOBP%GO:0051053	0.22862747	0.79163826	115	0.9583549	12382
MEMBRANE REPOLARIZATION%GOBP%GO:0086009	0.5229042	1.4715496	19	0.95838356	4165
IL3%NETPATH%IL3	0.23874985	0.791292	63	0.95841104	4523
FATTY ACID BIOSYNTHETIC PROCESS%GOBP%GO:0006633	0.23062703	0.78971756	79	0.95841473	5288
TRIVALENT INORGANIC CATION TRANSPORT%GOBP%GO:0072512	0.25017133	0.7895397	35	0.9584396	8331
FOXO FAMILY SIGNALING%PATHWAY INTERACTION DATABASE NCI-NATURE CURATED DATA%FOXO FAMILY SIGNALING	0.24162583	0.7845987	47	0.95845705	7013
MODULATION BY VIRUS OF HOST MORPHOLOGY OR PHYSIOLOGY%GOBP%GO:0019048	0.256661	0.78635836	28	0.95847493	5367
NUCLEAR-TRANSCRIBED MRNA CATABOLIC PROCESS%GOBP%GO:0000956	0.22166257	0.7840801	189	0.95850784	4078
POSITIVE REGULATION OF REACTIVE OXYGEN SPECIES METABOLIC PROCESS%GOBP%GO:2000379	0.24017668	0.78501797	54	0.9585283	6382
POSITIVE REGULATION OF LIPID KINASE ACTIVITY%GOBP%GO:0090218	0.2679639	0.7861479	21	0.95853055	104
TSLP%NETPATH%TSLP	0.22717844	0.7864717	119	0.9585352	11601
ARTERY MORPHOGENESIS%GOBP%GO:0048844	0.24775276	0.7846775	36	0.9585562	5930
REGULATION OF MYELINATION%GOBP%GO:0031641	0.26371065	0.78595436	24	0.95855635	2741
INTERLEUKIN-11 SIGNALING PATHWAY%WIKIPATHWAYS_20210110%WP2332%HOMO SAPIENS	0.24725036	0.78881913	38	0.95856774	12732
CHROMOSOME CONDENSATION%GOBP%GO:0030261	0.26107612	0.7832189	25	0.9585797	3034
FACE DEVELOPMENT%GOBP%GO:0060324	0.27358776	0.7889505	18	0.9585925	10245
NS1 MEDIATED EFFECTS ON HOST PATHWAYS%REACTOME DATABASE ID RELEASE 75%168276	0.25035015	0.78909713	37	0.9586022	5367
REGULATION OF MAST CELL DEGRANULATION%GOBP%GO:0043304	0.28118464	0.79129654	18	0.95860904	14775
DIACYLGLYCEROL METABOLIC PROCESS%GOBP%GO:0046339	0.26782653	0.78414655	21	0.95862263	16066
SIGNALING BY TGFB FAMILY MEMBERS%REACTOME%R-HSA-9006936.5	0.23086107	0.7892335	96	0.9586257	5995
AUTOPHAGOSOME ORGANIZATION%GOBP%GO:1905037	0.24179608	0.78474283	52	0.9586722	6814
MICROVILLUS ASSEMBLY%GOBP%GO:0030033	0.2906924	0.7865062	15	0.9586957	3604
C21-STEROID HORMONE METABOLIC PROCESS%GOBP%GO:0008207	0.27474025	0.7885644	18	0.95869833	5805
FATTY ACID METABOLIC PROCESS%GOBP%GO:0006631	0.21902516	0.7850347	220	0.9587067	9399
TRNA MODIFICATION IN THE NUCLEUS AND CYTOSOL%REACTOME DATABASE ID RELEASE 75%6782315	0.24169461	0.7866288	42	0.95873886	12701
MYELINATION%GOBP%GO:0042552	0.23723587	0.78322136	54	0.9587802	10239
QUINONE BIOSYNTHETIC PROCESS%GOBP%GO:1901663	0.28265807	0.78529304	16	0.9587842	1231
OUTER DYNEIN ARM ASSEMBLY%GOBP%GO:0036158	0.27834255	0.7854632	16	0.9587864	7437
DOWNREGULATION OF SMAD2 3:SMAD4 TRANSCRIPTIONAL ACTIVITY%REACTOME%R-HSA-2173795.1	0.2670569	0.7833551	23	0.958812	9716
AUTOPHAGOSOME MATURATION%GOBP%GO:0097352	0.25328672	0.78671795	31	0.9588361	7602
FATTY-ACYL-COA METABOLIC PROCESS%GOBP%GO:0035337	0.24880567	0.7850428	37	0.9588967	6972
METABOTROPIC GLUTAMATE RECEPTOR GROUP II PATHWAY%PANTHER PATHWAY%P00040	0.24746147	0.78553003	39	0.95890474	11141
REGULATION OF INTERLEUKIN-17 PRODUCTION%GOBP%GO:0032660	0.28866032	0.7882432	15	0.95890844	1995
TUMOR SUPPRESSOR ACTIVITY OF SMARCB1%WIKIPATHWAYS_20210110%WP4204%HOMO SAPIENS	0.25851163	0.7834114	26	0.9589461	5578
TRANSLESION SYNTHESIS BY POLH%REACTOME DATABASE ID RELEASE 75%110320	0.27032268	0.786724	19	0.9590321	11716
PHOSPHATIDYLETHANOLAMINE METABOLIC PROCESS%GOBP%GO:0046337	0.25633165	0.7826959	27	0.9590423	11295
REGULATION OF ACTIN FILAMENT DEPOLYMERIZATION%GOBP%GO:0030834	0.4605339	1.4470011	33	0.95904684	8493
NEGATIVE REGULATION OF AMYLOID-BETA FORMATION%GOBP%GO:1902430	0.5560317	1.5543816	17	0.9591048	5841
PROTEIN LOCALIZATION TO SYNAPSE%GOBP%GO:0035418	0.25791064	0.7867978	29	0.95914763	13235
CATECHOLAMINE METABOLIC PROCESS%GOBP%GO:0006584	0.26391715	0.78693694	23	0.95916826	6194
THE ROLE OF NEF IN HIV-1 REPLICATION AND DISEASE PATHOGENESIS%REACTOME DATABASE ID RELEASE 75%164952	0.48427474	1.4680566	26	0.9592129	4674
CELL AGING%GOBP%GO:0007569	0.24243632	0.78705156	43	0.95921636	8329
DOMAINS%GOBP%GO:0002460	0.43669286	1.4424036	54	0.95922333	6528
L1CAM INTERACTIONS%REACTOME%R-HSA-373760.2	0.22742671	0.78238666	92	0.9592333	4223
FATTY ACID METABOLISM%REACTOME%R-HSA-8978868.5	0.2254327	0.7871985	144	0.95923376	8291
REGULATION OF IGF ACTIVITY BY IGFBP%REACTOME%R-HSA-381426.2	0.22972628	0.78781235	96	0.959271	11925
RMTS METHYLATE HISTONE ARGININES%REACTOME DATABASE ID RELEASE 75%3214858	0.24561988	0.7876111	41	0.9593205	5762
REGULATION OF BRANCHING INVOLVED IN URETERIC BUD MORPHOGENESIS%GOBP%GO:0090189	0.29180428	0.78726983	15	0.95935	7094
TRANSLATIONAL ELONGATION%GOBP%GO:0006414	0.22812682	0.7874062	110	0.9593812	6575
TLR4 SIGNALING AND TOLERANCE%WIKIPATHWAYS_20210110%WP3851%HOMO SAPIENS	0.48829472	1.4418178	23	0.95945776	5609
REGULATION OF BICELLULAR TIGHT JUNCTION ASSEMBLY%GOBP%GO:2000810	0.51663435	1.4446269	18	0.9595845	3419
REGULATION OF BLOOD VESSEL SIZE%GOBP%GO:0050880	0.43190396	1.4186492	50	0.9596183	6031
G PROTEIN GATED POTASSIUM CHANNELS%REACTOME%R-HSA-1296059.2	0.46828273	1.4125338	28	0.9596607	6719
NAD METABOLIC PROCESS%GOBP%GO:0019674	0.42823443	1.4093163	63	0.9596633	4362
REGULATION OF RECEPTOR RECYCLING%GOBP%GO:0001919	0.5113589	1.4258595	16	0.95967114	1124
COMPLEMENT CASCADE%REACTOME%R-HSA-166658.4	0.49103138	1.4769449	25	0.9600525	3934
ESTABLISHMENT OF ENDOTHELIAL BARRIER%GOBP%GO:0061028	0.4767392	1.4411256	29	0.9603306	3604
REGULATION OF MUSCLE ORGAN DEVELOPMENT%GOBP%GO:0048634	0.23376663	0.7805456	63	0.9603324	5634
NEGATIVE REGULATION OF LEUKOCYTE CELL-CELL ADHESION%GOBP%GO:1903038	0.2379892	0.78057986	64	0.96049094	6153
SIGNALING EVENTS MEDIATED BY HEPATOCYTE GROWTH FACTOR RECEPTOR (C-MET)%PATHWAY INTERACTION DATABASE NCI-NATURE CURATED DATA%SIGNALING EVENTS MEDIATED BY HEPATOCYTE GROWTH FACTOR RECEPTOR (C-MET)	0.4244339	1.4388733	80	0.9605354	4378
POSITIVE REGULATION OF LIPID BIOSYNTHETIC PROCESS%GOBP%GO:0046889	0.2369099	0.7806851	57	0.96056175	14697
REGULATION OF AXON EXTENSION%GOBP%GO:0030516	0.43647075	1.4428048	57	0.96056676	5395
NATURAL KILLER CELL MEDIATED IMMUNITY%GOBP%GO:0002228	0.28349912	0.7783373	15	0.9606167	3465
RESPONSE TO INSULIN%GOBP%GO:0032868	0.21955839	0.7781539	136	0.96063566	11997
DETOXIFICATION%GOBP%GO:0098754	0.25145733	0.77961195	35	0.9606766	13788
TRANSPORT OF BILE SALTS AND ORGANIC ACIDS, METAL IONS AND AMINE COMPOUNDS%REACTOME%R-HSA-425366.3	0.23366497	0.77876824	62	0.9606877	3196
LEPTIN%NETPATH%LEPTIN	0.22951108	0.7789056	81	0.9607187	10541
CATECHOL-CONTAINING COMPOUND METABOLIC PROCESS%GOBP%GO:0009712	0.26391715	0.7807128	23	0.96073306	6194
SYNTHESIS OF VERY LONG-CHAIN FATTY ACYL-COAS%REACTOME%R-HSA-75876.5	0.26846328	0.7796915	21	0.96078163	11036
HEPARAN SULFATE PROTEOGLYCAN BIOSYNTHETIC PROCESS%GOBP%GO:0015012	0.27044827	0.778354	19	0.96079654	9999
DEFENSE RESPONSE TO BACTERIUM%GOBP%GO:0042742	0.22877492	0.7808078	82	0.96081305	12182
GLIAL CELL DEVELOPMENT%GOBP%GO:0021782	0.23738018	0.77782655	49	0.960837	5395
IL-2 SIGNALING PATHWAY%WIKIPATHWAYS_20210110%WP49%HOMO SAPIENS	0.24765253	0.77980405	33	0.9608493	13503
REGULATION OF CELL DIVISION%GOBP%GO:0051302	0.22748671	0.77893347	98	0.9608867	11660
CARBOHYDRATE TRANSPORT%GOBP%GO:0008643	0.25988957	0.77991074	27	0.960927	12555
SUPERPATHWAY OF D-<I>MYO< I>-INOSITOL (1,4,5)-TRISPHOSPHATE METABOLISM%HUMANCYC%PWY-6358	0.26848438	0.77841496	20	0.9609273	8404
T CELL RECEPTOR SIGNALING PATHWAY%GOBP%GO:0050852	0.41098827	1.4433297	136	0.96093684	8250
REGULATION OF INSULIN SECRETION%REACTOME%R-HSA-422356.4	0.23718365	0.7792116	70	0.9609537	9484
REGULATION OF PHOSPHOLIPID METABOLIC PROCESS%GOBP%GO:1903725	0.23993996	0.78081876	53	0.96100235	6137
POSITIVE REGULATION OF MORPHOGENESIS OF AN EPITHELIUM%GOBP%GO:1905332	0.25863627	0.77900535	27	0.9610024	7094
HYALURONAN METABOLISM%REACTOME%R-HSA-2142845.1	0.51151323	1.4100616	15	0.9610068	543
REGULATION OF EPIDERMAL GROWTH FACTOR-ACTIVATED RECEPTOR ACTIVITY%GOBP%GO:0007176	0.473232	1.4104813	23	0.9611713	7831
REGULATION OF TELOMERE MAINTENANCE%GOBP%GO:0032204	0.23083512	0.7767432	76	0.9613356	9660
PEPTIDYL-TYROSINE AUTOPHOSPHORYLATION%GOBP%GO:0038083	0.26407632	0.7768965	24	0.9613442	9771
RESPONSE TO AMMONIUM ION%GOBP%GO:0060359	0.44213912	1.4404062	50	0.9613853	5169
REGULATION OF CELLULAR CARBOHYDRATE METABOLIC PROCESS%GOBP%GO:0010675	0.22869462	0.77698433	84	0.961445	11546
COLLAGEN BIOSYNTHESIS AND MODIFYING ENZYMES%REACTOME DATABASE ID RELEASE 75%1650814	0.2313418	0.77713776	65	0.96145934	4534
SKIN DEVELOPMENT%GOBP%GO:0043588	0.4063973	1.4095123	105	0.9614642	5155
CD40 CD40L SIGNALING%PATHWAY INTERACTION DATABASE NCI-NATURE CURATED DATA%CD40 CD40L SIGNALING	0.46148267	1.4127467	29	0.96154076	5640
CELL DEATH IN RESPONSE TO OXIDATIVE STRESS%GOBP%GO:0036473	0.27694702	0.7763777	18	0.9615818	12456
NEGATIVE REGULATION OF NUCLEAR DIVISION%GOBP%GO:0051784	0.45058224	1.4260577	38	0.9620212	8983
ACTIVATION OF RRNA EXPRESSION BY ERCC6 (CSB) AND EHMT2 (G9A)%REACTOME%R-HSA-427389.2	0.24434075	0.7748219	35	0.9620599	5210
REGULATION OF CELLULAR RESPONSE TO INSULIN STIMULUS%GOBP%GO:1900076	0.23717222	0.7758029	47	0.96207774	11546
L13A-MEDIATED TRANSLATIONAL SILENCING OF CERULOPLASMIN EXPRESSION%REACTOME%R-HSA-156827.3	0.22434013	0.7752348	108	0.96215683	6211
MICROTUBULE NUCLEATION%GOBP%GO:0007020	0.2613397	0.7753939	23	0.9621654	16058
REGULATION OF INTERFERON-GAMMA PRODUCTION%GOBP%GO:0032649	0.23884793	0.77488285	49	0.96218866	7365
PROSTACYCLIN SIGNALLING THROUGH PROSTACYCLIN RECEPTOR%REACTOME DATABASE ID RELEASE 75%392851	0.5094114	1.4375428	18	0.9621954	6094
INTRINSIC APOPTOTIC SIGNALING PATHWAY IN RESPONSE TO DNA DAMAGE%GOBP%GO:0008630	0.4330552	1.4130976	49	0.96219814	6040
ATP SYNTHESIS COUPLED PROTON TRANSPORT%GOBP%GO:0015986	0.26275265	0.7735196	22	0.96220976	12988
MITOCHONDRIAL ELECTRON TRANSPORT, NADH TO UBIQUINONE%GOBP%GO:0006120	0.4798166	1.5552937	46	0.9622345	4191
TRANSFERRIN TRANSPORT%GOBP%GO:0033572	0.25017133	0.7728302	35	0.9622396	8331
NEGATIVE REGULATION OF CALCIUM ION TRANSMEMBRANE TRANSPORTER ACTIVITY%GOBP%GO:1901020	0.26180795	0.7754933	23	0.96224606	3787
REGULATION OF DOUBLE-STRAND BREAK REPAIR VIA NONHOMOLOGOUS END JOINING%GOBP%GO:2001032	0.25502703	0.7733032	29	0.96226025	8134
VALIDATED NUCLEAR ESTROGEN RECEPTOR ALPHA NETWORK%PATHWAY INTERACTION DATABASE NCI-NATURE CURATED DATA%VALIDATED NUCLEAR ESTROGEN RECEPTOR ALPHA NETWORK	0.23289487	0.7680244	61	0.9622619	15357
UBIQUINONE METABOLIC PROCESS%GOBP%GO:0006743	0.2755918	0.7735965	17	0.96232444	14854
MATRIX METALLOPROTEINASES%WIKIPATHWAYS_20210110%WP129%HOMO SAPIENS	0.26944044	0.7742708	18	0.9623331	3686
RESPONSE TO EPIDERMAL GROWTH FACTOR%GOBP%GO:0070849	0.25378165	0.7730766	29	0.9623407	13450
NEGATIVE REGULATION OF BMP SIGNALING PATHWAY%GOBP%GO:0030514	0.24493413	0.7725461	37	0.96235645	7849
PHASE 0 - RAPID DEPOLARISATION%REACTOME%R-HSA-5576892.2	0.25182757	0.7749076	31	0.9623619	7858
EVENTS	0.25032732	0.7740651	30	0.96236515	13502
PHENOL-CONTAINING COMPOUND METABOLIC PROCESS%GOBP%GO:0018958	0.24304381	0.77371687	39	0.9623702	14962

REGULATION OF SMAD PROTEIN SIGNAL TRANSDUCTION%GOBP%GO:0060390	0.26350603	0.7728808	21	0.9623774	14496
REGULATION OF ESTABLISHMENT OF CELL POLARITY%GOBP%GO:2000114	0.600895	1.6773188	17	0.9624142	3063
ROS AND RNS PRODUCTION IN PHAGOCYTES%REACTOME%R-HSA-1222556.9	0.24832454	0.76804197	29	0.9624427	5942
REGULATION OF BLOOD COAGULATION%GOBP%GO:0030193	0.43581215	1.4140007	50	0.96244407	3153
POSITIVE REGULATION OF INTERLEUKIN-10 PRODUCTION%GOBP%GO:0032733	0.2822404	0.77380574	15	0.96247417	5415
NEGATIVE REGULATION OF NEUROGENESIS%GOBP%GO:0050768	0.21992826	0.7743066	141	0.9624939	9395
CHAPERONE-MEDIATED PROTEIN COMPLEX ASSEMBLY%GOBP%GO:0051131	0.26664495	0.76765805	19	0.96250546	12297
POSITIVE REGULATION OF TELOMERE MAINTENANCE%GOBP%GO:0032206	0.24060181	0.7720124	48	0.9626032	12868
VISUAL PERCEPTION%GOBP%GO:0007601	0.23089032	0.7680661	71	0.96261847	7696
NEGATIVE REGULATION OF EXTRINSIC APOPTOTIC SIGNALING PATHWAY%GOBP%GO:2001237	0.22816862	0.76874	69	0.9626235	5981
DIGESTIVE TRACT MORPHOGENESIS%GOBP%GO:0048546	0.27302402	0.7721369	18	0.9626535	9324
ENERGY COUPLED PROTON TRANSPORT, DOWN ELECTROCHEMICAL GRADIENT%GOBP%GO:0015985	0.26275265	0.7707294	22	0.962686	12988
CENTROSOME DUPLICATION%GOBP%GO:0051298	0.24566099	0.76882833	33	0.9627232	3302
POSITIVE REGULATION OF CARTILAGE DEVELOPMENT%GOBP%GO:0061036	0.26899475	0.76830316	18	0.9627392	15602
REGULATION OF TP53 ACTIVITY THROUGH ACETYLATION%REACTOME%R-HSA-6804758.3	0.2527583	0.769312	30	0.96274483	7282
POSITIVE REGULATION OF SODIUM ION TRANSMEMBRANE TRANSPORT%GOBP%GO:1902307	0.26962456	0.76675117	18	0.96274674	3648
SIGNALING BY RAF1 MUTANTS%REACTOME%R-HSA-9656223.1	0.24874769	0.7708382	33	0.9627674	4909
REGULATION OF LEUKOCYTE ADHESION TO VASCULAR ENDOTHELIAL CELL%GOBP%GO:1904994	0.2720561	0.7691221	17	0.9627681	11244
PID_HES_HEY_PATHWAY%MSIGDB_C2%PID_HES_HEY_PATHWAY	0.45724666	1.4459313	38	0.96276814	6322
ARACHIDONIC ACID METABOLIC PROCESS%GOBP%GO:0019369	0.24077974	0.77166986	36	0.96280533	4142
THYMIC STROMAL LYMPHOPOIETIN (TSLP) SIGNALING PATHWAY%WIKIPATHWAYS_20210110%WP2203%HOMO SAPIENS	0.2455493	0.76807874	36	0.96280646	10939
PLASMA LIPOPROTEIN PARTICLE ORGANIZATION%GOBP%GO:0071827	0.244322	0.7659863	32	0.96281886	15041
CELL COMMUNICATION BY ELECTRICAL COUPLING%GOBP%GO:0010644	0.2800908	0.7704409	15	0.9628241	14253
NEGATIVE REGULATION OF CALCIUM ION TRANSPORT%GOBP%GO:0051926	0.2454474	0.76684815	35	0.96284443	6440
REGULATION OF HETEROTYPIC CELL-CELL ADHESION%GOBP%GO:0034114	0.27020818	0.7709376	18	0.9628548	6547
PID_INTEGRIN_A4B1_PATHWAY%MSIGDB_C2%PID_INTEGRIN_A4B1_PATHWAY	0.26091453	0.77022064	23	0.9628772	12420
NEGATIVE REGULATION OF STAT CASCADE%GOBP%GO:1904893	0.27110845	0.7683507	18	0.9628826	3153
POSITIVE REGULATION OF CELL CYCLE G2/M PHASE TRANSITION%GOBP%GO:1902751	0.48395014	1.4379915	23	0.96289015	5071
CHOLESTEROL HOMEOSTASIS%GOBP%GO:0042632	0.23382396	0.76696825	53	0.9629019	8196
NUCLEOTIDE SALVAGE%REACTOME DATABASE ID RELEASE 75%8956321	0.2602538	0.7664317	21	0.96290237	5423
IL5%NETPATH%IL5	0.23902747	0.7693446	44	0.9629062	10176
ACYL CHAIN REMODELLING OF PC%REACTOME DATABASE ID RELEASE 75%1482788	0.2638475	0.7688391	19	0.9629136	12587
CELLULAR RESPONSE TO INSULIN STIMULUS%GOBP%GO:0032869	0.22211818	0.76945984	124	0.9629689	11997
REGULATION OF MUSCLE TISSUE DEVELOPMENT%GOBP%GO:1901861	0.23150177	0.77134866	61	0.96297824	9791
PEPTIDYL-THREONINE DEPHOSPHORYLATION%GOBP%GO:0035970	0.26037186	0.7709954	22	0.96299124	5904
GTP HYDROLYSIS AND JOINING OF THE 60S RIBOSOMAL SUBUNIT%REACTOME%R-HSA-72706.2	0.2214615	0.7659926	109	0.9630124	6211
NEGATIVE REGULATION OF CELL AGING%GOBP%GO:0090344	0.2773674	0.7697627	16	0.96301395	199
PLATELET CALCIUM HOMEOSTASIS%REACTOME DATABASE ID RELEASE 75%418360	0.47088164	1.4453285	26	0.9630308	4139
POSITIVE REGULATION OF GLIAL CELL DIFFERENTIATION%GOBP%GO:0045687	0.27061662	0.77113384	18	0.9630316	4466
P38 MAPK SIGNALING PATHWAY%WIKIPATHWAYS_20210110%WP400%HOMO SAPIENS	0.251595	0.7656007	28	0.9630631	1281
NEGATIVE REGULATION OF SMALL GTPASE MEDIATED SIGNAL TRANSDUCTION%GOBP%GO:0051058	0.239873	0.7670001	41	0.96306366	7222
UNFOLDED PROTEIN RESPONSE (UPR)%REACTOME%R-HSA-381119.2	0.22243932	0.7660803	88	0.9631122	5400
NEGATIVE REGULATION OF STRIATED MUSCLE CELL DIFFERENTIATION%GOBP%GO:0051154	0.2611027	0.7694895	21	0.9631324	7219
CARBOXYLIC ACID TRANSMEMBRANE TRANSPORT%GOBP%GO:1905039	0.22701119	0.7698003	80	0.96316695	10244
GO ID NOT FOUND IN OBO FILE ONTOLOGY DEFINITIONS%GOBP%GO:0150104	0.22610655	0.76389056	72	0.96321493	3349
DEFENSE RESPONSE TO GRAM-NEGATIVE BACTERIUM%GOBP%GO:0050829	0.2483473	0.7652068	29	0.96330243	6382
SEMA3A PAK DEPENDENT AXON REPULSION%REACTOME%R-HSA-399954.2	0.2789643	0.7638925	16	0.9634125	6350
EAR MORPHOGENESIS%GOBP%GO:0042471	0.24406943	0.7647549	33	0.9634151	7112
POSITIVE REGULATION OF RESPONSE TO CYTOKINE STIMULUS%GOBP%GO:0060760	0.2427665	0.7641664	37	0.96350116	6935
FOLLICLE STIMULATING HORMONE (FSH) SIGNALING PATHWAY%WIKIPATHWAYS_20210110%WP2035%HOMO SAPIENS	0.259727	0.76398176	21	0.963507	14021
POSITIVE REGULATION OF DNA-TEMPLATED TRANSCRIPTION, ELONGATION%GOBP%GO:0032786	0.25429583	0.764481	25	0.9635275	2158
BIOCARTA_P53HYPOXIA_PATHWAY%MSIGDB_C2%BIOCARTA_P53HYPOXIA_PATHWAY	0.51231545	1.4152604	16	0.9635459	2049
APOPTOSIS MODULATION AND SIGNALING%WIKIPATHWAYS_20210110%WP1772%HOMO SAPIENS	0.43569848	1.4446905	70	0.96356887	3663
REGULATION OF CARTILAGE DEVELOPMENT%GOBP%GO:0061035	0.24417336	0.76479095	35	0.9635735	15998
REGULATION OF NUCLEAR-TRANSCRIBED MRNA CATABOLIC PROCESS, DEADENYLATION-DEPENDENT DECAY%GOBP%GO:1900151	0.26483443	0.7642252	20	0.9636308	4704
ASPARTATE FAMILY AMINO ACID CATABOLIC PROCESS%GOBP%GO:0009068	0.4919808	1.4263505	20	0.963653	4168
CAP-DEPENDENT TRANSLATION INITIATION%REACTOME DATABASE ID RELEASE 75%72737	0.21993226	0.763256	116	0.96373653	6211
ENERGY DEPENDENT REGULATION OF MTOR BY LKB1-AMPK%REACTOME%R-HSA-380972.1	0.2519312	0.76240486	28	0.96391946	2750
GDNF/RET SIGNALLING AXIS%WIKIPATHWAYS_20210110%WP4830%HOMO SAPIENS	0.2662519	0.76244843	19	0.9640723	10136
NOTCH3 INTRACELLULAR DOMAIN REGULATES TRANSCRIPTION%REACTOME%R-HSA-9013508.1	0.5551536	1.6144315	21	0.9640975	1090
ORGAN OR TISSUE SPECIFIC IMMUNE RESPONSE%GOBP%GO:0002251	0.27452767	0.76246387	15	0.96425676	13967
RESPONSE TO LIPOPOLYSACCHARIDE%GOBP%GO:0032496	0.21767424	0.7626312	122	0.96426123	8196
REACTIVE OXYGEN SPECIES METABOLIC PROCESS%GOBP%GO:0072593	0.22833437	0.7610356	66	0.96466285	5415
GPVI-MEDIATED ACTIVATION CASCADE%REACTOME DATABASE ID RELEASE 75%114604	0.47724727	1.4388849	24	0.9647218	5665
REGULATION OF ACTION POTENTIAL%GOBP%GO:0098900	0.43898854	1.4141495	46	0.96479213	5257
ORGANELLE TRANSPORT ALONG MICROTUBULE%GOBP%GO:0072384	0.23259266	0.76104546	61	0.964853	12380
POSITIVE REGULATION OF FATTY ACID METABOLIC PROCESS%GOBP%GO:0045923	0.25285164	0.76122123	25	0.96486145	6191
IRE1ALPHA ACTIVATES CHAPERONES%REACTOME%R-HSA-381070.1	0.23314711	0.76126873	49	0.96500677	5400
AMINE METABOLIC PROCESS%GOBP%GO:0009308	0.23543091	0.7600136	51	0.96519727	10610
NEGATIVE REGULATION OF TRANSFORMING GROWTH FACTOR BETA RECEPTOR SIGNALING PATHWAY%GOBP%GO:0030512	0.23082209	0.760107	62	0.9652927	8274
NEGATIVE REGULATION OF MACROAUTOPHAGY%GOBP%GO:0016242	0.46966156	1.4173896	26	0.9653067	7087
NEGATIVE REGULATION OF NERVOUS SYSTEM DEVELOPMENT%GOBP%GO:0051961	0.21623503	0.75968426	154	0.96537393	9395
POSITIVE REGULATION OF REGULATED SECRETORY PATHWAY%GOBP%GO:1903307	0.2417245	0.760196	34	0.96538883	5087
ENTRY INTO OTHER ORGANISM INVOLVED IN SYMBIOTIC INTERACTION%GOBP%GO:0051828	0.4494028	1.4131273	36	0.96548736	6947
REGULATION OF T CELL MEDIATED CYTOTOXICITY%GOBP%GO:0001914	0.5102573	1.4302319	18	0.9655245	4842
1D-<I>MYO< I>-INOSITOL HEXAKISPHOSPHATE BIOSYNTHESIS II (MAMMALIAN)%HUMANCYC%PWY-6362	0.2794504	0.75911915	15	0.9655898	7110
PID_NEPHRIN_NEPH1_PATHWAY%MSIGDB_C2%PID_NEPHRIN_NEPH1_PATHWAY	0.52891785	1.6120871	26	0.9655971	5640
UBIQUITIN-DEPENDENT PROTEIN CATABOLIC PROCESS VIA THE MULTIVESICULAR BODY SORTING PATHWAY%GOBP%GO:0043162	0.49166653	1.4360039	22	0.9655982	7660
CARDIAC SEPTUM MORPHOGENESIS%GOBP%GO:0060411	0.23628403	0.75889647	43	0.9656426	14496
ONCOGENIC MAPK SIGNALING%REACTOME DATABASE ID RELEASE 75%6802957	0.22237076	0.7592292	75	0.9656707	4202
POSITIVE REGULATION OF G2/M TRANSITION OF MITOTIC CELL CYCLE%GOBP%GO:0010971	0.5041142	1.4349061	20	0.9657158	5071
RETROGRADE TRANSPORT, ENDOSOME TO PLASMA MEMBRANE%GOBP%GO:1990126	0.25122437	0.7586015	24	0.9657505	13585
AMYOTROPHIC LATERAL SCLEROSIS (ALS)%WIKIPATHWAYS_20210110%WP2447%HOMO SAPIENS	0.46953312	1.4154063	24	0.966028	4202
NEURON PROJECTION ORGANIZATION%GOBP%GO:0106027	0.23881522	0.7580475	37	0.9661629	10885
B-WICH COMPLEX POSITIVELY REGULATES RRNA EXPRESSION%REACTOME%R-HSA-5250924.2	0.4395834	1.4342966	46	0.9664861	5533
REGULATION OF POSTSYNAPTIC MEMBRANE POTENTIAL%GOBP%GO:0060078	0.24128027	0.7573004	31	0.96659344	5700
REGULATION OF HEMOSTASIS%GOBP%GO:1900046	0.43581215	1.4295872	50	0.9665951	3153
TELOMERE MAINTENANCE%GOBP%GO:0000723	0.22301964	0.75743794	85	0.966635	12221
REGULATION OF LIPID CATABOLIC PROCESS%GOBP%GO:0050994	0.24654816	0.75546783	33	0.9668106	11374
EGF/EGFR SIGNALING PATHWAY%WIKIPATHWAYS_20210110%WP437%HOMO SAPIENS	0.21987297	0.7558136	116	0.96683604	11091
NUCLEOTIDE-BINDING OLIGOMERIZATION DOMAIN (NOD) PATHWAY%WIKIPATHWAYS_20210110%WP1433%HOMO SAPIENS	0.24925058	0.75687784	25	0.9668497	13130
EUKARYOTIC TRANSLATION INITIATION%REACTOME%R-HSA-72613.3	0.21993226	0.755575	116	0.9668909	6211
MONOCYTE CHEMOTAXIS%GOBP%GO:0002548	0.25525302	0.7566516	21	0.9669085	15443
ERROR-FREE TRANSLESION SYNTHESIS%GOBP%GO:0070987	0.2568085	0.7558603	21	0.966989	11789
NOTCH-MEDIATED HES HEY NETWORK%PATHWAY INTERACTION DATABASE NCI-NATURE CURATED DATA%NOTCH-MEDIATED HES HEY NETWORK	0.4451261	1.4143285	43	0.96702045	6322
REGULATION OF TP53 ACTIVITY THROUGH PHOSPHORYLATION%REACTOME DATABASE ID RELEASE 75%6804756	0.22240704	0.755998	87	0.96703744	12708
HEART LOOPING%GOBP%GO:0001947	0.23987508	0.7549979	35	0.96711886	7321
AXONEMAL DYNEIN COMPLEX ASSEMBLY%GOBP%GO:0070286	0.24579565	0.754809	28	0.9671239	7437
MATURATION OF SPIKE PROTEIN%REACTOME%R-HSA-9694548.1	0.2459033	0.7560821	29	0.96713823	11479
SPLICEOSOMAL COMPLEX ASSEMBLY%GOBP%GO:0000245	0.23153663	0.75625443	52	0.9671461	13999
ACTIVATION OF G PROTEIN GATED POTASSIUM CHANNELS%REACTOME%R-HSA-1296041.2	0.46828273	1.4269226	28	0.96718276	6719
POSITIVE REGULATION OF COAGULATION%GOBP%GO:0050820	0.5001892	1.4263847	18	0.9672655	3051
INFLUENZA VIRAL RNA TRANSCRIPTION AND REPLICATION%REACTOME DATABASE ID RELEASE 75%168273	0.21347997	0.7523118	130	0.9674377	8277
NEGATIVE REGULATION OF INTRINSIC APOPTOTIC SIGNALING PATHWAY BY P53 CLASS MEDIATOR%GOBP%GO:1902254	0.26642743	0.7521307	17	0.9674456	4867
CELLULAR RESPONSE TO FATTY ACID%GOBP%GO:0071398	0.26190868	0.7542938	20	0.9674754	11345
RHO GTPASES ACTIVATE NADPH OXIDASES%REACTOME%R-HSA-5668599.6	0.26118773	0.7518628	19	0.9675369	11324
NEGATIVE REGULATION OF FOCAL ADHESION ASSEMBLY%GOBP%GO:0051895	0.2732109	0.7523355	16	0.96760994	311
REGULATION OF PRODUCTION OF MIRNAS INVOLVED IN GENE SILENCING BY MIRNA%GOBP%GO:1903798	0.25998464	0.7437076	19	0.96761364	8401
CDP-DIACYLGLYCEROL BIOSYNTHESIS I%HUMANCYC%PWY-5667	0.26469243	0.7539826	18	0.96761376	2790
TELOMERE ORGANIZATION%GOBP%GO:0032200	0.22167206	0.7535648	87	0.96765304	12221
REGULATION OF PRODUCTION OF SMALL RNA INVOLVED IN GENE SILENCING BY RNA%GOBP%GO:0070920	0.25998464	0.74346995	19	0.96765536	8401
INNER EAR DEVELOPMENT%GOBP%GO:0048839	0.2300678	0.7537226	48	0.96768016	5812
KITRECEPTOR%IOB%KITRECEPTOR	0.22532782	0.7515265	70	0.9677011	9509
TRANSITION METAL ION TRANSPORT%GOBP%GO:0000041	0.22010106	0.7437733	82	0.9677438	9442
AXON ENSHEATHMENT%GOBP%GO:0008366	0.2243183	0.7431629	56	0.96777284	10239
DENDRITIC SPINE DEVELOPMENT%GOBP%GO:0060996	0.25725228	0.7523523	22	0.967793	10365
REGULATION OF VASCULAR ENDOTHELIAL GROWTH FACTOR PRODUCTION%GOBP%GO:0010574	0.25340828	0.753242	22	0.96780056	10576
REGULATION OF MYELOID LEUKOCYTE MEDIATED IMMUNITY%GOBP%GO:0002886	0.24996382	0.7495386	26	0.9678416	14775
KITRECEPTOR%NETPATH%KITRECEPTOR	0.22058329	0.7438386	77	0.9678783	9509
REGULATION OF JNK CASCADE%GOBP%GO:0046328	0.21366014	0.75244015	133	0.96789634	7312
REGULATION OF REPRODUCTIVE PROCESS%GOBP%GO:2000241	0.43470722	1.4351697	62	0.967916	6864
REGULATION OF FILOPODIUM ASSEMBLY%GOBP%GO:0051489	0.23589034	0.7490651	38	0.9679435	6834
POSITIVE REGULATION OF AUTOPHAGY OF MITOCHONDRION%GOBP%GO:1903599	0.27518284	0.7529411	15	0.96794534	2992
MEMBRANE DEPOLARIZATION DURING ACTION POTENTIAL%GOBP%GO:0086010	0.24431685	0.7498105	29	0.9679567	7858
CHROMATIN REMODELING AT CENTROMERE%GOBP%GO:0031055	0.24282153	0.75274116	32	0.967962	8699
GLYCOSYLATION AND RELATED CONGENITAL DEFECTS%WIKIPATHWAYS_20210110%WP4521%HOMO SAPIENS	0.24980992	0.7525257	22	0.9680003	8988
ACTIN NUCLEATION%GOBP%GO:0045010	0.25301903	0.7499452	23	0.9680117	14074

PRESYNAPSE ASSEMBLY%GOBP%GO:0099054	0.26383185	0.74917537	18	0.96801627	15104
REGULATION OF LIPID BIOSYNTHETIC PROCESS%GOBP%GO:0046890	0.21322244	0.7495495	142	0.9680282	14697
REGULATION OF GENERATION OF PRECURSOR METABOLITES AND ENERGY%GOBP%GO:0043467	0.21477827	0.7506606	127	0.96803176	8214
CELLULAR MODIFIED AMINO ACID BIOSYNTHETIC PROCESS%GOBP%GO:0042398	0.23897617	0.7438657	32	0.968044	4827
PID_TELOMERASE_PATHWAY%MSIGDB_C2%PID_TELOMERASE_PATHWAY	0.22789389	0.7483625	53	0.9680864	12221
ENSHEATHMENT OF NEURONS%GOBP%GO:0007272	0.2243183	0.7426623	56	0.968093	10239
POSITIVE REGULATION OF MRNA SPLICING, VIA SPLICEOSOME%GOBP%GO:0048026	0.26234445	0.7424512	17	0.96810836	230
REGULATION OF CIRCADIAN RHYTHM%GOBP%GO:0042752	0.21672109	0.7500354	96	0.9681132	9071
PLASMA MEMBRANE ORGANIZATION%GOBP%GO:0007009	0.22033295	0.74397504	72	0.96812916	3221
NEGATIVE REGULATION OF DNA REPAIR%GOBP%GO:0045738	0.2436436	0.7481115	33	0.9681427	8134
PLC BETA MEDIATED EVENTS%REACTOME DATABASE ID RELEASE 75%112043	0.22838224	0.7414412	51	0.96814424	4995
EPITHELIAL CELL PROLIFERATION%GOBP%GO:0050673	0.23313814	0.74218476	39	0.96817505	8141
MITOCHONDRIAL RNA PROCESSING%GOBP%GO:0000963	0.26866922	0.75088376	16	0.96819514	4795
SHP2 SIGNALING%PATHWAY INTERACTION DATABASE NCI-NATURE CURATED DATA%SHP2 SIGNALING	0.2298049	0.75068855	50	0.9681978	13414
REGULATION OF MEMBRANE DEPOLARIZATION%GOBP%GO:0003254	0.5146527	1.5559816	27	0.9682026	2892
SYNAPTIC TRANSMISSION, GLUTAMATERGIC%GOBP%GO:0035249	0.2641498	0.7501198	18	0.96822137	4936
SYNAPTIC SIGNALING PATHWAYS ASSOCIATED WITH AUTISM SPECTRUM DISORDER%WIKIPATHWAYS_20210110%WP4539%HOMO SAPIENS	0.23268937	0.74096215	39	0.9682323	14990
NEGATIVE REGULATION OF CELLULAR RESPONSE TO TRANSFORMING GROWTH FACTOR BETA STIMULUS%GOBP%GO:1903845	0.22571991	0.75029844	63	0.9682336	8274
CHROMATIN ASSEMBLY OR DISASSEMBLY%GOBP%GO:0006333	0.21660523	0.7484008	107	0.9682445	9436
DNA CATABOLIC PROCESS, ENDONUCLEOLYTIC%GOBP%GO:0000737	0.25453332	0.74034905	22	0.9682517	6105
REGULATION OF COMPLEMENT CASCADE%REACTOME DATABASE ID RELEASE 75%977606	0.4899018	1.4335358	23	0.96825725	3829
HORMONE-MEDIATED SIGNALING PATHWAY%GOBP%GO:0009755	0.22127052	0.7455431	75	0.9682602	8830
ORGANIC ACID TRANSMEMBRANE TRANSPORT%GOBP%GO:1903825	0.21987776	0.74112135	81	0.96826726	10244
HEPARAN SULFATE PROTEOGLYCAN METABOLIC PROCESS%GOBP%GO:0030201	0.24989231	0.7457193	25	0.96827716	8804
PHOTORECEPTOR CELL MAINTENANCE%GOBP%GO:0045494	0.24713466	0.7485483	26	0.9682948	6910
PHOSPHATIDYLCHOLINE BIOSYNTHETIC PROCESS%GOBP%GO:0006656	0.23883717	0.74145997	33	0.96832234	12555
CELLULAR ZINC ION HOMEOSTASIS%GOBP%GO:0006882	0.24498807	0.7439767	26	0.9683256	2338
REGULATION OF SMOOTH MUSCLE CONTRACTION%GOBP%GO:0006940	0.25523856	0.7458035	21	0.9683846	11369
POSITIVE REGULATION OF TOR SIGNALING%GOBP%GO:0032008	0.23501328	0.7440982	37	0.96839434	8759
TRNA WOBBLE BASE MODIFICATION%GOBP%GO:0002097	0.2630836	0.7403725	17	0.9684263	9183
REGULATION OF TUMOR NECROSIS FACTOR PRODUCTION%GOBP%GO:0032680	0.21873772	0.7442475	74	0.9684371	6935
REGULATION OF CYTOKINESIS%GOBP%GO:0032465	0.22104962	0.744595	73	0.96845526	11660
NICOTINIC ACETYLCHOLINE RECEPTOR SIGNALING PATHWAY%PANTHER PATHWAY%P00044	0.22059053	0.74171036	72	0.96846116	13358
HDMS DEMETHYLATE HISTONES%REACTOME%R-HSA-3214842.3	0.25236076	0.7415145	24	0.9684657	11808
PID_IL2_1PATHWAY%MSIGDB_C2%PID_IL2_1PATHWAY	0.22579925	0.7229349	41	0.9684941	14655
RRNA PROCESSING IN THE NUCLEUS AND CYTOSOL%REACTOME DATABASE ID RELEASE 75%8868773	0.20847662	0.74049276	188	0.9685048	7111
RETINA LAYER FORMATION%GOBP%GO:0010842	0.2599286	0.7235333	16	0.968509	4021
MAJOR PATHWAY OF RRNA PROCESSING IN THE NUCLEOLUS AND CYTOSOL%REACTOME%R-HSA-6791226.3	0.21179518	0.7443639	178	0.9685158	8105
NUCLEIC ACID PHOSPHODIESTER BOND HYDROLYSIS%GOBP%GO:0090305	0.21197881	0.745853	143	0.96853006	11736
ENTRY INTO HOST CELL%GOBP%GO:0030260	0.4494028	1.436181	36	0.96853554	6947
REGULATION OF CHEMOKINE PRODUCTION%GOBP%GO:0032642	0.23248732	0.7446136	44	0.96863604	6810
PEPTIDYL-TYROSINE PHOSPHORYLATION%GOBP%GO:0018108	0.21774673	0.7219428	55	0.9686421	10403
ID%NETPATH%ID	0.23614302	0.7200116	30	0.968665	14046
IL9%NETPATH%IL9	0.25822043	0.72316027	16	0.96867186	13296
NEGATIVE REGULATION OF SYNAPTIC TRANSMISSION%GOBP%GO:0050805	0.24084839	0.72294503	26	0.968678	3281
SELENOAMINO ACID METABOLISM%REACTOME DATABASE ID RELEASE 75%2408522	0.20775688	0.7225183	112	0.96867824	3703
INTERFERON-GAMMA SIGNALING PATHWAY%PANTHER PATHWAY%P00035	0.23767231	0.7235533	28	0.9686854	13156
RRNA CATABOLIC PROCESS%GOBP%GO:0016075	0.26360917	0.7220936	17	0.968686	2943
AXONAL TRANSPORT%GOBP%GO:0098930	0.22352609	0.72228366	48	0.9686997	14570
HALLMARK_KRAS_SIGNALING_UP%MSIGDB_C2%HALLMARK_KRAS_SIGNALING_UP	0.21325119	0.7458704	132	0.96871185	13152
DNA DAMAGE BYPASS%REACTOME DATABASE ID RELEASE 75%73893	0.23006575	0.7447097	48	0.9687265	4545
PHOSPHATIDYLSERINE METABOLIC PROCESS%GOBP%GO:0006658	0.251365	0.7448648	23	0.96876794	13303
CYTOPLASMIC MICROTUBULE ORGANIZATION%GOBP%GO:0031122	0.223723	0.7384553	54	0.9687842	16058
ENDODERM DIFFERENTIATION%WIKIPATHWAYS_20210110%WP2853%HOMO SAPIENS	0.20769845	0.7200638	107	0.9688117	7117
PERK REGULATES GENE EXPRESSION%REACTOME DATABASE ID RELEASE 75%381042	0.2410777	0.73858047	31	0.96884793	16930
ESTABLISHMENT OF CELL POLARITY%GOBP%GO:0030010	0.21867147	0.73895943	75	0.9688549	9725
IL23-MEDIATED SIGNALING EVENTS%PATHWAY INTERACTION DATABASE NCI-NATURE CURATED DATA%IL23-MEDIATED SIGNALING EVENTS	0.2433225	0.723561	24	0.9688718	5320
PHOSPHATIDYLGLYCEROL BIOSYNTHESIS II (NON-PLASTIDIC)%HUMANCYC%PWY4FS-8	0.24660765	0.7182533	19	0.9688831	2790
REGULATION OF TUMOR NECROSIS FACTOR SUPERFAMILY CYTOKINE PRODUCTION%GOBP%GO:1903555	0.21276887	0.7208409	79	0.9688846	6935
CELL-CELL JUNCTION ORGANIZATION%REACTOME%R-HSA-421270.5	0.43056282	1.4155071	50	0.9688905	7574
CCR1%IOB%CCR1	0.25965884	0.7458805	21	0.96889776	4619
PID_AR_PATHWAY%MSIGDB_C2%PID_AR_PATHWAY	0.22581162	0.7188961	40	0.96890044	10416
CILIUM MOVEMENT%GOBP%GO:0003341	0.22628513	0.73814136	47	0.96890324	7416
ARTERY DEVELOPMENT%GOBP%GO:0060840	0.22409502	0.72016716	42	0.9689124	5930
DRUG TRANSPORT%GOBP%GO:0015893	0.20870508	0.71843207	102	0.96892065	13177
RNA PHOSPHODIESTER BOND HYDROLYSIS%GOBP%GO:0090501	0.22317125	0.74699384	65	0.96892333	4104
TRANSCRIPTION BY RNA POLYMERASE III%GOBP%GO:0006383	0.23559493	0.72371167	29	0.9689336	8398
DEGRADATION OF BETA CATENIN%PATHWAY INTERACTION DATABASE NCI-NATURE CURATED DATA%DEGRADATION OF BETA CATENIN	0.50059354	1.4272169	17	0.96893895	8192
PHASE I - FUNCTIONALIZATION OF COMPOUNDS%REACTOME%R-HSA-211945.5	0.21445772	0.7214068	69	0.96894264	10371
LUNG FIBROSIS%WIKIPATHWAYS_20210110%WP3624%HOMO SAPIENS	0.22639753	0.7190463	38	0.96895355	14537
POTENTIAL THERAPEUTICS FOR SARS%REACTOME%R-HSA-9679191.2	0.21639244	0.7205431	73	0.96896106	3851
NUCLEOSOME ASSEMBLY%GOBP%GO:0006334	0.22000054	0.73769146	70	0.9689678	9436
RAF-INDEPENDENT MAPK1 3 ACTIVATION%REACTOME%R-HSA-112409.2	0.25131187	0.73922545	22	0.968968	11618
ESTROGEN RECEPTOR SIGNALING	0.2253605	0.7185825	37	0.9689766	11105
TOR SIGNALING%GOBP%GO:0031929	0.24213006	0.72094107	26	0.9689818	6847
REGULATION OF STRESS-ACTIVATED PROTEIN KINASE SIGNALING CASCADE%GOBP%GO:0070302	0.20902345	0.73862505	173	0.9689993	7312
PID_MTOR_4PATHWAY%MSIGDB_C2%PID_MTOR_4PATHWAY	0.2223874	0.7390061	51	0.9690058	14132
SIALIC ACID METABOLISM%REACTOME%R-HSA-4085001.2	0.24137886	0.74593437	30	0.9690433	7050
REGULATION OF PROTEIN AUTOPHOSPHORYLATION%GOBP%GO:0031952	0.23114349	0.73721546	38	0.96904767	15604
FLK2 FLT3%IOB%FLK2 FLT3	0.25918984	0.7279193	17	0.9690514	3187
XBP1(S) ACTIVATES CHAPERONE GENES%REACTOME DATABASE ID RELEASE 75%381038	0.22056453	0.72021985	47	0.96905863	5400
NEGATIVE REGULATION OF RESPONSE TO DNA DAMAGE STIMULUS%GOBP%GO:2001021	0.21853718	0.727701	72	0.969067	11016
NEGATIVE REGULATION OF EPITHELIAL TO MESENCHYMAL TRANSITION%GOBP%GO:0010719	0.23951802	0.73775595	30	0.9691054	9065
TRNA TRANSPORT%GOBP%GO:0051031	0.23624156	0.7466326	36	0.9691075	4994
KINESINS%REACTOME%R-HSA-983189.3	0.22661036	0.7237259	41	0.9691149	9477
CENTRAL NERVOUS SYSTEM NEURON AXONOGENESIS%GOBP%GO:0021955	0.2528856	0.74699557	22	0.9691204	10394
EPITHELIAL CILIUM MOVEMENT%GOBP%GO:0003351	0.25913322	0.7241494	16	0.96912056	4026
COLLAGEN FORMATION%REACTOME%R-HSA-1474290.3	0.20878394	0.72099626	85	0.96912724	4848
NEGATIVE REGULATION OF CELLULAR RESPONSE TO GROWTH FACTOR STIMULUS%GOBP%GO:0090288	0.20722361	0.7190653	119	0.9691276	7979
BRANCHED-CHAIN AMINO ACID CATABOLIC PROCESS%GOBP%GO:0009083	0.24379568	0.72390807	22	0.9691485	9777
METABOTROPIC GLUTAMATE RECEPTOR GROUP III PATHWAY%PANTHER PATHWAY%P00039	0.22325392	0.73923457	60	0.9691578	10042
ER-NUCLEUS SIGNALING PATHWAY%GOBP%GO:0006984	0.23336983	0.72798043	32	0.96918887	6040
METABOLIC REPROGRAMMING IN COLON CANCER%WIKIPATHWAYS_20210110%WP4290%HOMO SAPIENS	0.25270185	0.7459795	20	0.9691974	154
REGULATION OF TELOMERASE%PATHWAY INTERACTION DATABASE NCI-NATURE CURATED DATA%REGULATION OF TELOMERASE	0.22256182	0.74617505	64	0.9691991	12221
PROTEIN K48-LINKED UBIQUITINATION%GOBP%GO:0070936	0.22018644	0.72817785	52	0.96920073	9855
AXO-DENDRITIC TRANSPORT%GOBP%GO:0008088	0.22704463	0.7372177	53	0.9692416	14570
NEGATIVE REGULATION OF SMOOTH MUSCLE CELL PROLIFERATION%GOBP%GO:0048662	0.24727863	0.74631727	26	0.96924275	12223
REGULATION OF DNA-TEMPLATED TRANSCRIPTION, ELONGATION%GOBP%GO:0032784	0.22788855	0.73387265	46	0.969245	12958
TYROBP CAUSAL NETWORK%WIKIPATHWAYS_20210110%WP3945%HOMO SAPIENS	0.23167408	0.71913785	36	0.96925294	15106
REGULATION OF CELL-SUBSTRATE JUNCTION ASSEMBLY%GOBP%GO:0090109	0.21887277	0.7241517	52	0.9693125	12086
POSITIVE REGULATION OF DNA RECOMBINATION%GOBP%GO:0045911	0.24145342	0.7367307	32	0.96933085	7747
ONCOSTATIN M SIGNALING PATHWAY%WIKIPATHWAYS_20210110%WP2374%HOMO SAPIENS	0.22357711	0.7339574	54	0.9693638	14655
REGULATION OF BLOOD VESSEL DIAMETER%GOBP%GO:0097746	0.43190396	1.4158971	50	0.9693699	6031
REGULATION OF SIGNAL TRANSDUCTION BY P53 CLASS MEDIATOR%GOBP%GO:1901796	0.20570037	0.72818696	161	0.969388	4867
OXIDOREDUCTION COENZYME METABOLIC PROCESS%GOBP%GO:0006733	0.41239744	1.4302565	107	0.96941334	4362
HALLMARK_ANGIOGENESIS%MSIGDB_C2%HALLMARK_ANGIOGENESIS	0.24127972	0.73445493	27	0.96946687	1899
STEROL REGULATORY ELEMENT-BINDING PROTEINS (SREBP) SIGNALLING%WIKIPATHWAYS_20210110%WP1982%HOMO SAPIENS	0.2211703	0.7165346	49	0.96947736	11398
BODY MORPHOGENESIS%GOBP%GO:0010171	0.25199652	0.7282727	20	0.969501	10245
REGULATION OF FOCAL ADHESION ASSEMBLY%GOBP%GO:0051893	0.21887277	0.7241553	52	0.96950245	12086
M-CSF%IOB%M-CSF	0.23004758	0.7363483	39	0.96951747	2924
MITOCHONDRIAL TRNA AMINOACYLATION%REACTOME DATABASE ID RELEASE 75%379726	0.2510692	0.73397595	21	0.9695402	6751
SIGNALING	0.23054567	0.73453504	45	0.96958095	11660
MONONUCLEAR CELL MIGRATION%GOBP%GO:0071674	0.24279281	0.71660393	24	0.9696092	15443
REGULATION OF POLYSACCHARIDE METABOLIC PROCESS%GOBP%GO:0032881	0.23702906	0.7356624	32	0.9696384	11546
THE INFLUENCE OF LAMINOPATHIES ON WNT SIGNALING%WIKIPATHWAYS_20210110%WP4844%HOMO SAPIENS	0.24056543	0.72420853	27	0.9696463	14204
BLOOD VESSEL ENDOTHELIAL CELL MIGRATION%GOBP%GO:0043534	0.25166246	0.73526067	21	0.9696538	17819
REGULATION OF STRIATED MUSCLE TISSUE DEVELOPMENT%GOBP%GO:0016202	0.2192105	0.7340547	60	0.9696579	10532
DENDRITIC SPINE ORGANIZATION%GOBP%GO:0097061	0.24162242	0.7348476	27	0.96967304	10365
IL-6 SIGNALING PATHWAY%WIKIPATHWAYS_20210110%WP364%HOMO SAPIENS	0.22748926	0.726844	35	0.96968704	10314
PID_ERA_GENOMIC_PATHWAY%MSIGDB_C2%PID_ERA_GENOMIC_PATHWAY	0.2269834	0.7282757	47	0.96969384	15272
RETINA HOMEOSTASIS%GOBP%GO:0001895	0.22367407	0.71670455	48	0.96970624	9167
G-PROTEIN MEDIATED EVENTS%REACTOME%R-HSA-112040.1	0.22244057	0.726608	52	0.9697165	4995
HS-GAG BIOSYNTHESIS%REACTOME%R-HSA-2022928.1	0.22813633	0.715173	31	0.96972007	4090
REGULATION OF RESPONSE TO CYTOKINE STIMULUS%GOBP%GO:0060759	0.2115742	0.7350016	131	0.9697246	12211
CELLULAR ROLES OF ANTHRAX TOXIN%PATHWAY INTERACTION DATABASE NCI-NATURE CURATED DATA%CELLULAR ROLES OF ANTHRAX TOXIN	0.25428152	0.71165484	16	0.9697334	9706
SIGNALING PATHWAY	0.2306455	0.71535903	32	0.9697427	12347
NEOVASCULARISATION PROCESSES%WIKIPATHWAYS_20210110%WP4331%HOMO SAPIENS	0.24355696	0.7357244	28	0.96976507	7352

NUCLEAR RECEPTOR TRANSCRIPTION PATHWAY%REACTOME DATABASE ID RELEASE 75%383280	0.2355327	0.7345453	34	0.96976936	7033
ABORTIVE ELONGATION OF HIV-1 TRANSCRIPT IN THE ABSENCE OF TAT%REACTOME DATABASE ID RELEASE 75%167242	0.24337313	0.7148858	23	0.9697709	6669
PID_BMP_PATHWAY%MSIGDB_C2%PID_BMP_PATHWAY	0.23717995	0.7358959	31	0.9697778	15132
MITOCHONDRIAL GENE EXPRESSION%WIKIPATHWAYS_20210110%WP391%HOMO SAPIENS	0.2555965	0.7157544	19	0.96978194	10018
REGULATION OF CARDIAC MUSCLE CELL PROLIFERATION%GOBP%GO:0060043	0.25569555	0.73531175	20	0.9697935	9065
BIOCARTA_EICOSANOID_PATHWAY%MSIGDB_C2%BIOCARTA_EICOSANOID_PATHWAY	0.26232842	0.72836506	17	0.9697986	4220
EXERCISE-INDUCED CIRCADIAN REGULATION%WIKIPATHWAYS_20210110%WP410%HOMO SAPIENS	0.24322459	0.72547364	27	0.96981514	8113
MAMMARY GLAND DEVELOPMENT PATHWAY - PREGNANCY AND LACTATION (STAGE 3 OF 4)%WIKIPATHWAYS_20210110%WP2817%HOMO SAPIENS	0.24458586	0.7242379	22	0.96981585	10831
SIGNALING EVENTS MEDIATED BY PTP1B%PATHWAY INTERACTION DATABASE NCI-NATURE CURATED DATA%SIGNALING EVENTS MEDIATED BY PTP1B	0.22171728	0.7145913	44	0.9698389	5320
PID_IL4_2PATHWAY%MSIGDB_C2%PID_IL4_2PATHWAY	0.23060346	0.73038626	38	0.9698406	15268
NUCLEOSOME ORGANIZATION%GOBP%GO:0034728	0.21127972	0.73015976	106	0.9698531	9436
PID_RAC1_PATHWAY%MSIGDB_C2%PID_RAC1_PATHWAY	0.23006271	0.716727	38	0.96987885	10725
REGULATION OF PEPTIDYL-LYSINE ACETYLATION%GOBP%GO:2000756	0.21899208	0.71541893	48	0.9698818	4902
MRNA CLEAVAGE%GOBP%GO:0006379	0.24693693	0.73116904	22	0.9698845	9331
REGULATION OF DNA BIOSYNTHETIC PROCESS%GOBP%GO:2000278	0.21130045	0.73155236	96	0.9699077	12868
REGULATION OF ACTIVATED T CELL PROLIFERATION%GOBP%GO:0046006	0.24324971	0.7169015	24	0.96990776	5724
POSITIVE REGULATION OF FOCAL ADHESION ASSEMBLY%GOBP%GO:0051894	0.2575611	0.73216087	17	0.96991616	7352
REGULATION OF MESENCHYMAL CELL PROLIFERATION%GOBP%GO:0010464	0.26744038	0.7245422	15	0.96991825	2385
RESPONSE TO ANTIBIOTIC%GOBP%GO:0046677	0.20745657	0.7142846	98	0.96992064	9562
RESPONSE TO ETHANOL%GOBP%GO:0045471	0.25720096	0.71580666	16	0.9699232	15452
NEGATIVE REGULATION OF GENE SILENCING%GOBP%GO:0060969	0.24186738	0.7255682	25	0.9699237	722
PROTEIN SECRETION%GOBP%GO:0009306	0.21505503	0.7116552	62	0.9699246	11458
VESICLE CYTOSKELETAL TRAFFICKING%GOBP%GO:0099518	0.22090115	0.7304901	56	0.9699412	12202
REGULATION OF PROTEIN ACETYLATION%GOBP%GO:1901983	0.22113638	0.7257285	56	0.96995544	4902
PID_RB_1PATHWAY%MSIGDB_C2%PID_RB_1PATHWAY	0.43487108	1.4307035	49	0.96996063	4567
SNRNA 3'-END PROCESSING%GOBP%GO:0034472	0.25074828	0.7259139	20	0.969976	15068
CELLULAR KETONE METABOLIC PROCESS%GOBP%GO:0042180	0.21984787	0.7111402	45	0.96998245	1231
IL-7%NETPATH%IL-7	0.25035813	0.7242623	21	0.96998376	13296
REGULATION OF GLYCOPROTEIN METABOLIC PROCESS%GOBP%GO:1903018	0.23579434	0.7318835	33	0.9699839	15100
SPLICING FACTOR NOVA REGULATED SYNAPTIC PROTEINS%WIKIPATHWAYS_20210110%WP4148%HOMO SAPIENS	0.2327418	0.72837055	34	0.9699877	14567
REGULATION OF FAT CELL DIFFERENTIATION%GOBP%GO:0045598	0.21110956	0.71178484	87	0.97000116	8107
RESPONSE TO AMINO ACID%GOBP%GO:0043200	0.22454412	0.7108528	39	0.9700398	8449
ERBB1 DOWNSTREAM SIGNALING%PATHWAY INTERACTION DATABASE NCI-NATURE CURATED DATA%ERBB1 DOWNSTREAM SIGNALING	0.21392478	0.7316088	103	0.970049	11091
ANGIOPOIETIN LIKE PROTEIN 8 REGULATORY PATHWAY%WIKIPATHWAYS_20210110%WP3915%HOMO SAPIENS	0.21221724	0.73058677	100	0.9700496	12418
SENSORY PERCEPTION OF LIGHT STIMULUS%GOBP%GO:0050953	0.21250257	0.71305275	75	0.9700536	7696
CELLULAR METABOLIC COMPOUND SALVAGE%GOBP%GO:0043094	0.23444714	0.7326204	32	0.9700538	14414
MITOCHONDRIAL RESPIRATORY CHAIN COMPLEX IV ASSEMBLY%GOBP%GO:0033617	0.24363396	0.71282595	20	0.97005385	9623
IRON ION TRANSPORT%GOBP%GO:0006826	0.22263798	0.72459984	49	0.97005725	9228
SIGNALING BY HIGH-KINASE ACTIVITY BRAF MUTANTS%REACTOME%R-HSA-6802948.1	0.23944433	0.7311877	28	0.9700608	4202
ACTIVATED TAK1 MEDIATES P38 MAPK ACTIVATION%REACTOME DATABASE ID RELEASE 75%450302	0.25832453	0.7322077	18	0.97006935	10095
NEGATIVE REGULATION OF CALCIUM-MEDIATED SIGNALING%GOBP%GO:0050849	0.2486355	0.7259966	21	0.9700921	3097
ANDROGEN RECEPTOR ACTIVITY	0.22247699	0.7307431	56	0.97009224	10416
IL2-MEDIATED SIGNALING EVENTS%PATHWAY INTERACTION DATABASE NCI-NATURE CURATED DATA%IL2-MEDIATED SIGNALING EVENTS	0.22199935	0.71213394	44	0.97009385	14655
CRANIAL NERVE DEVELOPMENT%GOBP%GO:0021545	0.25494978	0.72475404	17	0.9701012	9489
HISTONE PHOSPHORYLATION%GOBP%GO:0016572	0.24970771	0.7123171	18	0.97012854	6722
RRNA PROCESSING%REACTOME DATABASE ID RELEASE 75%72312	0.20398277	0.7323296	198	0.9701412	7111
POSITIVE REGULATION OF LIPID METABOLIC PROCESS%GOBP%GO:0045834	0.20573695	0.71184206	96	0.97014695	6191
POSITIVE REGULATION OF CYTOKINESIS%GOBP%GO:0032467	0.24036007	0.72490364	27	0.97014904	11648
PHOSPHODIESTERASES IN NEURONAL FUNCTION%WIKIPATHWAYS_20210110%WP4222%HOMO SAPIENS	0.23523642	0.7136031	32	0.9701538	3649
NEGATIVE REGULATION OF AMINE TRANSPORT%GOBP%GO:0051953	0.2666204	0.7284009	15	0.97015494	10649
COLLAGEN CHAIN TRIMERIZATION%REACTOME DATABASE ID RELEASE 75%8948216	0.22713111	0.73271096	43	0.9701651	11154
RHO GTPASES ACTIVATE PKNS%REACTOME%R-HSA-5625740.1	0.2244509	0.72960466	50	0.97017574	3656
NEGATIVE REGULATION OF TUMOR NECROSIS FACTOR PRODUCTION%GOBP%GO:0032720	0.23706968	0.7130612	28	0.9702391	6935
CELLULAR RESPONSE TO EPIDERMAL GROWTH FACTOR STIMULUS%GOBP%GO:0071364	0.24220389	0.7284943	26	0.970268	6843
DETECTION OF STIMULUS INVOLVED IN SENSORY PERCEPTION%GOBP%GO:0050906	0.22441444	0.71237606	40	0.9702692	5898
REGULATION OF STRESS-ACTIVATED MAPK CASCADE%GOBP%GO:0032872	0.20228377	0.7136673	171	0.97028154	7312
GOLGI TO ENDOSOME TRANSPORT%GOBP%GO:0006895	0.2600424	0.72886276	16	0.97029895	4684
PRC2 METHYLATES HISTONES AND DNA%REACTOME DATABASE ID RELEASE 75%212300	0.23411088	0.7286586	32	0.9703124	3166
UROKINASE-TYPE PLASMINOGEN ACTIVATOR (UPA) AND UPAR-MEDIATED SIGNALING%PATHWAY INTERACTION DATABASE NCI-NATURE CURATED DATA%UROKINASE-TYPE PLASMINOGEN ACTIVATOR (UPA) AND UPAR-MEDIATED SIGNALING	0.53837997	1.6160265	27	0.970388	4127
SECONDARY METABOLIC PROCESS%GOBP%GO:0019748	0.23679368	0.7130955	27	0.9703977	8093
FORMATION OF ATP BY CHEMIOSMOTIC COUPLING%REACTOME%R-HSA-163210.2	0.25550866	0.7291245	18	0.97043085	12988
PEPTIDYL-ARGININE MODIFICATION%GOBP%GO:0018195	0.25945958	0.72892797	16	0.97043467	12356
GLIAL CELL DIFFERENTIATION%GOBP%GO:0010001	0.21070744	0.70944273	74	0.9704846	5395
NUCLEOTIDE CATABOLIC PROCESS%GOBP%GO:0009166	0.4201153	1.4275101	82	0.9705448	5021
REGULATION OF OSTEOBLAST PROLIFERATION%GOBP%GO:0033688	0.2615842	0.70998436	15	0.9706009	14913
SYNTHESIS OF PIPS AT THE GOLGI MEMBRANE%REACTOME%R-HSA-1660514.2	0.25717613	0.7095108	15	0.9706203	14749
MACROPHAGE ACTIVATION%GOBP%GO:0042116	0.23870172	0.7083977	23	0.9706206	14063
AROMATIC AMINO ACID FAMILY CATABOLIC PROCESS%GOBP%GO:0009074	0.25086114	0.70810527	19	0.9706812	3817
REGULATION OF HEART RATE BY CARDIAC CONDUCTION%GOBP%GO:0086091	0.45237884	1.4316632	36	0.970708	6324
HEPARAN SULFATE HEPARIN (HS-GAG) METABOLISM%REACTOME%R-HSA-1638091.1	0.21688434	0.7096271	54	0.9707101	5746
PIGMENT BIOSYNTHETIC PROCESS%GOBP%GO:0046148	0.22698595	0.70873296	34	0.97072136	4107
HISTONE H2A MONOUBIQUITINATION%GOBP%GO:0035518	0.25136572	0.7084612	17	0.9707596	1713
STABILIZATION AND EXPANSION OF THE E-CADHERIN ADHERENS JUNCTION%PATHWAY INTERACTION DATABASE NCI-NATURE CURATED DATA%STABILIZATION AND EXPANSION OF THE E-CADHERIN ADHERENS JUNCTION	0.48840463	1.5785773	41	0.9707952	7649
ATF4 ACTIVATES GENES IN RESPONSE TO ENDOPLASMIC RETICULUM STRESS%REACTOME DATABASE ID RELEASE 75%380994	0.23747407	0.7088443	26	0.9708212	16930
RESPIRATORY CHAIN COMPLEX IV ASSEMBLY%GOBP%GO:0008535	0.23987378	0.7076944	24	0.9708397	9623
LONG-CHAIN FATTY-ACYL-COA BIOSYNTHETIC PROCESS%GOBP%GO:0035338	0.2543069	0.7059057	15	0.97120064	8103
REGULATION OF GLYCOGEN METABOLIC PROCESS%GOBP%GO:0070873	0.23440698	0.70604193	26	0.97127604	11546
TRANSCRIPTION INITIATION FROM RNA POLYMERASE I PROMOTER%GOBP%GO:0006361	0.4662513	1.428473	30	0.9713978	6737
SLC TRANSPORTER DISORDERS%REACTOME%R-HSA-5619102.6	0.2084448	0.706109	72	0.9714124	8514
OVARIAN TUMOR DOMAIN PROTEASES%REACTOME DATABASE ID RELEASE 75%5689896	0.22676107	0.70667666	35	0.9715122	13796
POSITIVE REGULATION OF TELOMERASE ACTIVITY%GOBP%GO:0051973	0.22275522	0.7064358	35	0.9715245	12868
DEVELOPMENT OF PRIMARY FEMALE SEXUAL CHARACTERISTICS%GOBP%GO:0046545	0.23249516	0.7061682	26	0.9715574	15108
ORGANOPHOSPHATE ESTER TRANSPORT%GOBP%GO:0015748	0.20992036	0.70501846	72	0.9717539	6919
POSITIVE REGULATION OF HEMOSTASIS%GOBP%GO:1900048	0.5001892	1.4160476	18	0.9718042	3051
REGULATION OF GLUCONEOGENESIS%GOBP%GO:0006111	0.22907463	0.7044281	26	0.9718615	1794
PID_ATR_PATHWAY%MSIGDB_C2%PID_ATR_PATHWAY	0.22484255	0.7027461	30	0.97189975	11716
POSITIVE REGULATION OF PEPTIDE SECRETION%GOBP%GO:0002793	0.2077113	0.7044412	87	0.9720434	11374
DISEASES OF PROGRAMMED CELL DEATH%REACTOME%R-HSA-9645723.3	0.23158048	0.7032663	26	0.9720453	9167
REGULATION OF OXIDATIVE PHOSPHORYLATION%GOBP%GO:0002082	0.2337715	0.703464	27	0.9720653	2478
REGULATION OF INTERLEUKIN-10 PRODUCTION%GOBP%GO:0032653	0.23581333	0.70274997	24	0.97208804	13296
ACTIVATION OF GENE EXPRESSION BY SREBF (SREBP)%REACTOME DATABASE ID RELEASE 75%2426168	0.22194433	0.70364153	39	0.9721079	5114
ADHERENS JUNCTION ASSEMBLY%GOBP%GO:0034333	0.46943158	1.4325278	29	0.9721802	7220
REGULATION OF VIRAL ENTRY INTO HOST CELL%GOBP%GO:0046596	0.22857505	0.7037644	30	0.9722055	16577
MICROTUBULE-BASED MOVEMENT%GOBP%GO:0007018	0.19617976	0.70276093	212	0.97227013	9557
REGULATION OF PLATELET ACTIVATION%GOBP%GO:0010543	0.5533919	1.6248194	23	0.97274923	1408
NEGATIVE REGULATION OF UBIQUITIN-DEPENDENT PROTEIN CATABOLIC PROCESS%GOBP%GO:2000059	0.44604483	1.430866	39	0.9728765	3064
POSITIVE REGULATION OF AMYLOID-BETA FORMATION%GOBP%GO:1902004	0.25346577	0.6996022	16	0.97333354	4446
ZINC ION TRANSPORT%GOBP%GO:0006829	0.24192725	0.69981176	19	0.97335875	8975
CELLULAR RESPONSE TO ANTIBIOTIC%GOBP%GO:0071236	0.21380936	0.70048076	63	0.97337776	3536
CELL MIGRATION INVOLVED IN SPROUTING ANGIOGENESIS%GOBP%GO:0002042	0.2463629	0.7000056	17	0.9733915	17819
TRANSCRIPTION BY RNA POLYMERASE I%GOBP%GO:0006360	0.46509117	1.4276587	32	0.97347707	6737
IL-7 SIGNALING PATHWAY%WIKIPATHWAYS_20210110%WP205%HOMO SAPIENS	0.2393891	0.70009136	20	0.97351396	13296
RESPONSE TO AXON INJURY%GOBP%GO:0048678	0.2551005	0.70053774	15	0.9735219	486
EPO RECEPTOR SIGNALING%WIKIPATHWAYS_20210110%WP581%HOMO SAPIENS	0.24598321	0.6986634	18	0.9735277	12211
ZINC ION HOMEOSTASIS%GOBP%GO:0055069	0.23446834	0.6991229	27	0.97353697	2338
PROTEIN LOCALIZATION TO CELL SURFACE%GOBP%GO:0034394	0.23450224	0.6988815	25	0.9735435	14601
CD28 DEPENDENT PI3K AKT SIGNALING%REACTOME DATABASE ID RELEASE 75%389357	0.4887671	1.4317588	20	0.97409165	4268
REGULATION OF BETA-CELL DEVELOPMENT%REACTOME%R-HSA-186712.2	0.5875715	1.8109003	31	0.9743534	1571
NEGATIVE REGULATION OF INTRINSIC APOPTOTIC SIGNALING PATHWAY IN RESPONSE TO DNA DAMAGE%GOBP%GO:1902230	0.2310215	0.69692755	26	0.9743631	3656
INTRA-GOLGI TRAFFIC%REACTOME%R-HSA-6811438.1	0.21582721	0.6953642	41	0.9744337	4816
POSITIVE REGULATION OF RNA SPLICING%GOBP%GO:0033120	0.22707795	0.6960834	30	0.97444546	928
UBIQUITIN-DEPENDENT ERAD PATHWAY%GOBP%GO:0030433	0.20670919	0.69577795	70	0.97449076	7642
CEREBRAL CORTEX CELL MIGRATION%GOBP%GO:0021795	0.2514866	0.69697034	16	0.97452056	2097
PHOSPHATIDYLINOSITOL PHOSPHORYLATION%GOBP%GO:0046854	0.21925437	0.69543904	37	0.9745701	11621
STEROL METABOLIC PROCESS%GOBP%GO:0016125	0.2059416	0.6971437	70	0.974571	6142
DARPP-32 EVENTS%REACTOME%R-HSA-180024.3	0.23663896	0.69613665	23	0.9745964	14253
GABA SYNTHESIS, RELEASE, REUPTAKE AND DEGRADATION%REACTOME DATABASE ID RELEASE 75%888590	0.24946454	0.69628435	16	0.9746673	15210
GENOTOXICITY PATHWAY%WIKIPATHWAYS_20210110%WP4286%HOMO SAPIENS	0.21582194	0.6947821	49	0.9747	6598
ASSEMBLY AND CELL SURFACE PRESENTATION OF NMDA RECEPTORS%REACTOME%R-HSA-9609736.3	0.5345113	1.6177725	24	0.97530997	5700
REGULATION OF INSULIN RECEPTOR SIGNALING PATHWAY%GOBP%GO:0046626	0.21558338	0.6933842	43	0.97540236	11546
RESPONSE TO REACTIVE OXYGEN SPECIES%GOBP%GO:0000302	0.20178226	0.69359285	103	0.9754326	10153
REGULATION OF RECEPTOR-MEDIATED ENDOCYTOSIS%GOBP%GO:0048259	0.20707344	0.6930707	76	0.97544926	10336
G-CSF%IOB%G-CSF	0.2214157	0.69233984	33	0.9756491	2118
REGULATION OF RHOA ACTIVITY%PATHWAY INTERACTION DATABASE NCI-NATURE CURATED DATA%REGULATION OF RHOA ACTIVITY	0.5041392	1.6199133	45	0.9756923	2916
OVARIAN INFERTILITY GENES%WIKIPATHWAYS_20210110%WP34%HOMO SAPIENS	0.24261563	0.69124967	18	0.975714	8057

POSITIVE REGULATION OF PROTEIN SECRETION%GOBP%GO:0050714	0.20559305	0.69149333	75	0.97571653	11374
REGULATION OF NATURAL KILLER CELL ACTIVATION%GOBP%GO:0032814	0.2574609	0.69099927	15	0.9757202	3983
HEMATOPOIETIC PROGENITOR CELL DIFFERENTIATION%GOBP%GO:0002244	0.2250062	0.6923955	28	0.97580236	12265
GM-CSF%IOB%GM-CSF	0.21175595	0.69183683	55	0.9758361	10539
MODIFICATION BY SYMBIONT OF HOST MORPHOLOGY OR PHYSIOLOGY%GOBP%GO:0044003	0.22548912	0.69155324	30	0.97586083	5367
PROTEIN QUALITY CONTROL FOR MISFOLDED OR INCOMPLETELY SYNTHESIZED PROTEINS%GOBP%GO:0006515	0.5159703	1.5564636	26	0.9761555	1890
REGULATION OF NITRIC OXIDE BIOSYNTHETIC PROCESS%GOBP%GO:0045428	0.22284332	0.68964833	30	0.9765623	11179
O-GLYCAN PROCESSING%GOBP%GO:0016266	0.2100797	0.6811801	47	0.9768459	6357
PID_IL3_PATHWAY%MSIGDB_C2%PID_IL3_PATHWAY	0.2406287	0.6804646	17	0.9769679	10764
POSITIVE REGULATION OF PHOSPHOLIPID METABOLIC PROCESS%GOBP%GO:1903727	0.22614326	0.68068427	27	0.9770047	104
SIGNALING BY NTRK2 (TRKB)%REACTOME DATABASE ID RELEASE 75%9006115	0.23600805	0.68855506	21	0.97700775	12347
RIBOSOMAL SMALL SUBUNIT ASSEMBLY%GOBP%GO:0000028	0.23327702	0.6811846	19	0.9770327	12977
PID_IL8_CXCR2_PATHWAY%MSIGDB_C2%PID_IL8_CXCR2_PATHWAY	0.2330206	0.68471795	21	0.9770597	4995
REGULATION OF PRESYNAPSE ASSEMBLY%GOBP%GO:1905606	0.2487705	0.68494	16	0.9770851	318
AMINO ACIDS REGULATE MTORC1%REACTOME DATABASE ID RELEASE 75%9639288	0.2111634	0.6887028	54	0.9770921	8746
O-GLYCOSYLATION OF TSR DOMAIN-CONTAINING PROTEINS%REACTOME DATABASE ID RELEASE 75%5173214	0.21587622	0.6813232	38	0.97711533	13281
FRAGILE X SYNDROME %WIKIPATHWAYS_20210110%WP4549%HOMO SAPIENS	0.20160574	0.6861295	93	0.9771431	8486
PROTEIN HYDROXYLATION%GOBP%GO:0018126	0.23193893	0.68151414	24	0.9771661	9558
NUCLEOSIDE BIOSYNTHETIC PROCESS%GOBP%GO:0009163	0.22763844	0.68175864	25	0.97717816	4355
POSITIVE REGULATION OF AMYLOID PRECURSOR PROTEIN CATABOLIC PROCESS%GOBP%GO:1902993	0.23489118	0.6799329	20	0.97717816	9790
CELLULAR RESPONSE TO PH%GOBP%GO:0071467	0.25006694	0.68582666	15	0.9771782	13585
CENP-A CONTAINING CHROMATIN ORGANIZATION%GOBP%GO:0061641	0.2193974	0.6820009	30	0.9771857	8364
HUMAN THYROID STIMULATING HORMONE (TSH) SIGNALING PATHWAY%WIKIPATHWAYS_20210110%WP2032%HOMO SAPIENS	0.20878607	0.6852913	56	0.97720337	14021
NEGATIVE REGULATION OF ADHERENS JUNCTION ORGANIZATION%GOBP%GO:1903392	0.2395939	0.68502533	19	0.97720987	311
REGULATION OF SINGLE STRANDED VIRAL RNA REPLICATION VIA DOUBLE STRANDED DNA INTERMEDIATE%GOBP%GO:0045091	0.24351811	0.6795922	15	0.977222	8140
BIOCARTA_MTOR_PATHWAY%MSIGDB_C2%BIOCARTA_MTOR_PATHWAY	0.23841746	0.6842294	18	0.97722715	10960
BMP RECEPTOR SIGNALING%PATHWAY INTERACTION DATABASE NCI-NATURE CURATED DATA%BMP RECEPTOR SIGNALING	0.21632232	0.6855085	40	0.9772349	15132
HORMONE TRANSPORT%GOBP%GO:0009914	0.21410298	0.68214256	42	0.9772721	14403
INTRACELLULAR ESTROGEN RECEPTOR SIGNALING PATHWAY%GOBP%GO:0030520	0.25403738	0.6861926	15	0.9772857	8165
FORMATION OF A POOL OF FREE 40S SUBUNITS%REACTOME%R-HSA-72689.2	0.1981998	0.6863387	98	0.97736484	6506
POSITIVE REGULATION OF NEUROLOGICAL SYSTEM PROCESS%GOBP%GO:0031646	0.21922398	0.6870486	33	0.97739017	6588
NEGATIVE REGULATION OF TUMOR NECROSIS FACTOR SUPERFAMILY CYTOKINE PRODUCTION%GOBP%GO:1903556	0.2249705	0.6865151	29	0.97741795	6935
POSITIVE REGULATION OF PHOSPHATASE ACTIVITY%GOBP%GO:0010922	0.23495568	0.68218213	19	0.977434	14021
MEMBRANE RAFT ORGANIZATION%GOBP%GO:0031579	0.58729285	1.6839331	19	0.9774447	3372
HISTONE MODIFICATIONS%WIKIPATHWAYS_20210110%WP2369%HOMO SAPIENS	0.22001174	0.687178	33	0.97748035	7394
TGF-BETA RECEPTOR SIGNALING ACTIVATES SMADS%REACTOME DATABASE ID RELEASE 75%2173789	0.22123443	0.68364924	31	0.97748446	5995
REGULATION OF PRESYNAPSE ORGANIZATION%GOBP%GO:0099174	0.2487705	0.68668103	16	0.9774847	318
NEGATIVE REGULATION OF T CELL DIFFERENTIATION%GOBP%GO:0045581	0.22372153	0.6789474	24	0.9775017	6153
T CELL MEDIATED IMMUNITY%GOBP%GO:0002456	0.5748922	1.5748055	16	0.9775107	3643
GASTRIC CANCER NETWORK 2%WIKIPATHWAYS_20210110%WP2363%HOMO SAPIENS	0.22730151	0.68229765	26	0.9775362	16400
VENTRICULAR SEPTUM MORPHOGENESIS%GOBP%GO:0060412	0.22958228	0.68733805	26	0.97755903	13568
NCRNA EXPORT FROM NUCLEUS%GOBP%GO:0097064	0.21661438	0.68272924	38	0.9776016	4994
PHOSPHOLIPID TRANSLOCATION%GOBP%GO:0045332	0.2316243	0.6824116	20	0.9776418	15790
CHONDROITIN SULFATE DERMATAN SULFATE METABOLISM%REACTOME DATABASE ID RELEASE 75%1793185	0.20843811	0.67844296	48	0.97766733	10480
COLLAGEN FIBRIL ORGANIZATION%GOBP%GO:0030199	0.21928059	0.6828674	33	0.9776917	4400
DNA REPLICATION-INDEPENDENT NUCLEOSOME ASSEMBLY%GOBP%GO:0006336	0.2181633	0.6830985	39	0.97770697	9172
POSITIVE REGULATION OF PROTEIN TARGETING TO MEMBRANE%GOBP%GO:0090314	0.23507477	0.68734473	22	0.9777444	13753
CELLULAR DETOXIFICATION%GOBP%GO:1990748	0.22245727	0.66603154	25	0.97786176	13788
RETROGRADE PROTEIN TRANSPORT, ER TO CYTOSOL%GOBP%GO:0030970	0.23949635	0.6655243	17	0.9780078	4545
CELLULAR RESPONSE TO AMINO ACID STIMULUS%GOBP%GO:0071230	0.21127638	0.66604364	34	0.9780427	8449
POSITIVE REGULATION OF NEUTROPHIL CHEMOTAXIS%GOBP%GO:0090023	0.24331078	0.6661108	15	0.9781881	17879
MRNA SPLICE SITE SELECTION%GOBP%GO:0006376	0.21754779	0.6649131	30	0.97822195	13999
PID_EPHRINB_REV_PATHWAY%MSIGDB_C2%PID_EPHRINB_REV_PATHWAY	0.23188642	0.6772979	20	0.97830105	6134
REGULATION OF TRANSFORMING GROWTH FACTOR BETA PRODUCTION%GOBP%GO:0071634	0.2185216	0.6664999	26	0.97830373	10558
CELLULAR AMINE METABOLIC PROCESS%GOBP%GO:0044106	0.20432387	0.6644328	45	0.97835565	10610
REGULATION OF HISTONE ACETYLATION%GOBP%GO:0035065	0.21218349	0.6764211	42	0.978369	4837
OVARIAN FOLLICLE DEVELOPMENT%GOBP%GO:0001541	0.24341178	0.6661169	15	0.9783733	14917
ENDOPLASMIC RETICULUM TO CYTOSOL TRANSPORT%GOBP%GO:1903513	0.23949635	0.6666297	17	0.97840744	4545
PHOTOTRANSDUCTION%GOBP%GO:0007602	0.24284542	0.6765483	16	0.9784681	13337
HALLMARK_ANDROGEN_RESPONSE%MSIGDB_C2%HALLMARK_ANDROGEN_RESPONSE	0.19886644	0.66395664	63	0.9784905	11800
ACTIVATION OF PROTEIN KINASE B ACTIVITY%GOBP%GO:0032148	0.21349221	0.6627775	29	0.9785279	3763
RIBONUCLEOSIDE CATABOLIC PROCESS%GOBP%GO:0042454	0.24456573	0.66331446	15	0.97854596	4355
TRANSPORT ALONG MICROTUBULE%GOBP%GO:0010970	0.19133939	0.6635842	125	0.97855085	3266
PYRIMIDINE NUCLEOSIDE BIOSYNTHETIC PROCESS%GOBP%GO:0046134	0.23335762	0.66246265	18	0.97855747	4355
CARTILAGE DEVELOPMENT%GOBP%GO:0051216	0.20220514	0.675646	60	0.9785675	13836
IRE1-MEDIATED UNFOLDED PROTEIN RESPONSE%GOBP%GO:0036498	0.202229	0.66665256	54	0.9785826	5400
CENP-A CONTAINING NUCLEOSOME ASSEMBLY%GOBP%GO:0034080	0.2193974	0.67665344	30	0.978587	8364
NEGATIVE REGULATION OF LIPID TRANSPORT%GOBP%GO:0032369	0.23974283	0.6674551	16	0.97858953	5549
ARP2/3 COMPLEX-MEDIATED ACTIN NUCLEATION%GOBP%GO:0034314	0.23025681	0.66770744	19	0.97861373	14074
RESPONSE TO TOXIC SUBSTANCE%GOBP%GO:0009636	0.19370377	0.67499983	142	0.9786422	7468
BODY FLUID SECRETION%GOBP%GO:0007589	0.22594477	0.66708714	21	0.9786552	14593
CONNECTIVE TISSUE DEVELOPMENT%GOBP%GO:0061448	0.1968711	0.67522925	81	0.97867256	9248
POST-TRANSLATIONAL MODIFICATION: SYNTHESIS OF GPI-ANCHORED PROTEINS%REACTOME DATABASE ID RELEASE 75%163125	0.1992172	0.6667847	69	0.9786789	6547
SYNAPSE ASSEMBLY%GOBP%GO:0007416	0.20011568	0.674661	75	0.97868526	11679
LAMINOPATHIES%WIKIPATHWAYS_20210110%WP4879%HOMO SAPIENS	0.2056464	0.6628056	39	0.97869927	5331
FEMALE GONAD DEVELOPMENT%GOBP%GO:0008585	0.22731923	0.67571884	25	0.97870445	15108
EXTRACELLULAR MATRIX DISASSEMBLY%GOBP%GO:0022617	0.20679231	0.667748	47	0.9787742	4496
ANTEROGRADE AXONAL TRANSPORT%GOBP%GO:0008089	0.21409743	0.67292863	37	0.9787919	14570
DNA REPLICATION-INDEPENDENT NUCLEOSOME ORGANIZATION%GOBP%GO:0034724	0.20995978	0.66825056	40	0.9788191	9172
TRISTETRAPROLIN (TTP, ZFP36) BINDS AND DESTABILIZES MRNA%REACTOME%R-HSA-450513.2	0.23750967	0.6679496	17	0.97883165	15466
COFACTOR TRANSPORT%GOBP%GO:0051181	0.21750635	0.6724692	34	0.97892153	14196
DNA DOUBLE-STRAND BREAK PROCESSING%GOBP%GO:0000729	0.2352067	0.6721956	19	0.97892714	15464
REGULATION OF REACTIVE OXYGEN SPECIES BIOSYNTHETIC PROCESS%GOBP%GO:1903426	0.20324254	0.6729917	54	0.97893435	6382
ACTIN FILAMENT POLYMERIZATION%GOBP%GO:0030041	0.21864131	0.66833293	28	0.97895014	6164
FEMALE SEX DIFFERENTIATION%GOBP%GO:0046660	0.21663705	0.66858894	31	0.9789554	15108
N-GLYCAN ANTENNAE ELONGATION IN THE MEDIAL TRANS-GOLGI%REACTOME%R-HSA-975576.1	0.22368973	0.6710698	25	0.9789661	11540
CELLULAR BIOGENIC AMINE METABOLIC PROCESS%GOBP%GO:0006576	0.20432387	0.66153264	45	0.9789736	10610
MICROTUBULE-BASED TRANSPORT%GOBP%GO:0099111	0.19133939	0.66910225	125	0.9789984	3266
DETECTION OF EXTERNAL STIMULUS%GOBP%GO:0009581	0.20539267	0.669334	50	0.979022	13337
TRANSPORT OF MATURE MRNA DERIVED FROM AN INTRONLESS TRANSCRIPT%REACTOME%R-HSA-159231.2	0.21063063	0.6701415	39	0.9790258	4994
PHOSPHATIDYLETHANOLAMINE ACYL-CHAIN REMODELING%GOBP%GO:0036152	0.2378769	0.6687028	17	0.97906417	17303
NEGATIVE REGULATION OF PROTEIN ACETYLATION%GOBP%GO:1901984	0.24653505	0.6717401	15	0.9790689	11246
CYTOSKELETON-DEPENDENT INTRACELLULAR TRANSPORT%GOBP%GO:0030705	0.1907422	0.67300975	159	0.97911024	11972
PIGMENT METABOLIC PROCESS%GOBP%GO:0042440	0.21172759	0.6734975	41	0.9791244	4107
RESPONSE TO EXOGENOUS DSRNA%GOBP%GO:0043330	0.24056493	0.67109823	18	0.9791338	3562
IL-3 SIGNALING PATHWAY%WIKIPATHWAYS_20210110%WP286%HOMO SAPIENS	0.21626836	0.6702535	33	0.979135	4268
VISUAL BEHAVIOR%GOBP%GO:0007632	0.24697311	0.6736962	15	0.9791696	5841
POSITIVE REGULATION OF CYCLIN-DEPENDENT PROTEIN SERINE/THREONINE KINASE ACTIVITY%GOBP%GO:0045737	0.23472777	0.66009635	17	0.9791751	12236
REGULATION OF SYNAPSE ASSEMBLY%GOBP%GO:0051963	0.20779924	0.6731709	49	0.97918546	12295
DETECTION OF ABIOTIC STIMULUS%GOBP%GO:0009582	0.20298208	0.66936463	54	0.979193	13337
REGULATION OF RIG-I SIGNALING PATHWAY%GOBP%GO:0039535	0.23692977	0.6602535	16	0.97925407	4545
POSITIVE REGULATION OF CELL-SUBSTRATE ADHESION%GOBP%GO:0010811	0.19432934	0.6591069	71	0.9792691	8928
POSITIVE REGULATION OF NIK/NF-KAPPAB SIGNALING%GOBP%GO:1901224	0.20089114	0.65967417	51	0.9792705	4417
GLAND DEVELOPMENT%GOBP%GO:0048732	0.19523309	0.6703107	112	0.97928977	9100
TRP CHANNELS%REACTOME DATABASE ID RELEASE 75%3295583	0.23892145	0.6711391	16	0.97929806	2319
MITOCHONDRIAL RNA METABOLIC PROCESS%GOBP%GO:0000959	0.21039434	0.660458	37	0.9793051	4795
GOLGI TO PLASMA MEMBRANE PROTEIN TRANSPORT%GOBP%GO:0043001	0.21650895	0.66068363	30	0.9793536	10958
DETOXIFICATION OF REACTIVE OXYGEN SPECIES%REACTOME%R-HSA-3299685.5	0.21911806	0.66936755	28	0.97938156	5007
POSITIVE REGULATION OF SYNAPTIC TRANSMISSION, GLUTAMATERGIC%GOBP%GO:0051968	0.229527	0.6586073	19	0.9794122	13027
REGULATION OF ATPASE ACTIVITY%GOBP%GO:0043462	0.20148954	0.65830725	53	0.9794194	8878
NUCLEOBASE-CONTAINING SMALL MOLECULE INTERCONVERSION%GOBP%GO:0015949	0.21679927	0.6591436	27	0.9794321	8749
REGULATION OF CELLULAR RESPONSE TO VASCULAR ENDOTHELIAL GROWTH FACTOR STIMULUS%GOBP%GO:1902547	0.22898734	0.65798736	19	0.97944677	14138
REGULATION OF NIK/NF-KAPPAB SIGNALING%GOBP%GO:1901222	0.19552654	0.657663	71	0.979466	12415
ORGAN GROWTH%GOBP%GO:0035265	0.21674575	0.6572118	25	0.97956836	3855
ACYLGLYCEROL METABOLIC PROCESS%GOBP%GO:0006639	0.19576395	0.6558564	65	0.980039	18513
ACYLGLYCEROL BIOSYNTHETIC PROCESS%GOBP%GO:0046463	0.22710843	0.6561466	20	0.98005396	17051
REGULATION OF CARDIAC MUSCLE CELL ACTION POTENTIAL%GOBP%GO:0098901	0.5081677	1.5759823	31	0.9800843	2769
POSTREPLICATION REPAIR%GOBP%GO:0006301	0.19995274	0.65362346	50	0.98049504	4837
NEUROMUSCULAR PROCESS%GOBP%GO:0050905	0.21653444	0.6540051	29	0.98062927	11097
DEFECTIVE B3GALTL CAUSES PETERS-PLUS SYNDROME (PPS)%REACTOME%R-HSA-5083635.1	0.20959811	0.6545626	37	0.9806508	13281
NUCLEOBASE BIOSYNTHETIC PROCESS%GOBP%GO:0046112	0.2380633	0.6536759	15	0.98065096	11012
NEUTRAL LIPID METABOLIC PROCESS%GOBP%GO:0006638	0.19576395	0.65308356	65	0.9806556	18513
INFLUENZA INFECTION%REACTOME DATABASE ID RELEASE 75%168255	0.18596976	0.65425664	149	0.9806583	8514
CELLULAR CARBOHYDRATE CATABOLIC PROCESS%GOBP%GO:0044275	0.5096157	1.5613261	27	0.98071146	8534
POSITIVE REGULATION OF HEART CONTRACTION%GOBP%GO:0045823	0.517713	1.5586122	26	0.98110354	7588
MORPHOGENESIS OF A POLARIZED EPITHELIUM%GOBP%GO:0001738	0.21781537	0.6519505	26	0.9811562	5922
NEUTRAL LIPID BIOSYNTHETIC PROCESS%GOBP%GO:0046460	0.22710843	0.65073586	20	0.98136467	17051

PYRIDINE NUCLEOTIDE METABOLIC PROCESS%GOBP%GO:0019362	0.4524008	1.5625383	90	0.9813695	4362
FOREBRAIN GENERATION OF NEURONS%GOBP%GO:0021872	0.21128303	0.65002304	28	0.9814004	3770
ESTABLISHMENT OF PROTEIN LOCALIZATION TO PLASMA MEMBRANE%GOBP%GO:0061951	0.20366861	0.651122	44	0.9814978	14336
MOTOR NEURON AXON GUIDANCE%GOBP%GO:0008045	0.23603237	0.6508201	15	0.9814983	10110
DEVELOPMENTAL PIGMENTATION%GOBP%GO:0048066	0.21296328	0.65010047	26	0.98153967	10124
REGULATION OF PHAGOCYTOSIS%GOBP%GO:0050764	0.1978251	0.6486623	52	0.9816585	11179
S1P1 PATHWAY%PATHWAY INTERACTION DATABASE NCI-NATURE CURATED DATA%S1P1 PATHWAY	0.22654144	0.6492819	19	0.98166096	10940
INFLAMMASOMES%REACTOME%R-HSA-622312.1	0.23554495	0.6488451	17	0.98173213	5101
ENDOCYTIC RECYCLING%GOBP%GO:0032456	0.20287548	0.6476933	39	0.9820609	14747
MITOTIC SPINDLE ASSEMBLY%GOBP%GO:0090307	0.20083661	0.6473695	43	0.98206526	15422
THE HUMAN IMMUNE RESPONSE TO TUBERCULOSIS%WIKIPATHWAYS_20210110%WP4197%HOMO SAPIENS	0.22315057	0.6458765	20	0.98221654	271
NITRIC OXIDE STIMULATES GUANYLATE CYCLASE%REACTOME DATABASE ID RELEASE 75%392154	0.22294134	0.6466762	21	0.9822978	12436
PID_ER_NONGENOMIC_PATHWAY%MSIGDB_C2%PID_ER_NONGENOMIC_PATHWAY	0.21112154	0.6462429	30	0.9823743	12446
REGULATION OF CELLULAR RESPONSE TO OXIDATIVE STRESS%GOBP%GO:1900407	0.19568865	0.6459227	55	0.98237664	2283
INTEGRATION OF ENERGY METABOLISM%REACTOME DATABASE ID RELEASE 75%163685	0.18670698	0.6443811	97	0.9827355	9484
INTRACELLULAR TRAFFICKING PROTEINS INVOLVED IN CMT NEUROPATHY%WIKIPATHWAYS_20210110%WP4856%HOMO SAPIENS	0.21547845	0.64466	22	0.98275775	13585
POSITIVE REGULATION OF PROTEIN EXPORT FROM NUCLEUS%GOBP%GO:0046827	0.23151192	0.6398333	16	0.9832074	14931
FATTY ACYL-COA BIOSYNTHESIS%REACTOME%R-HSA-75105.6	0.2073295	0.6399339	34	0.9833338	11336
DNA CATABOLIC PROCESS%GOBP%GO:0006308	0.21120541	0.6400551	28	0.98345125	18155
T CELL ACTIVATION%PANTHER PATHWAY%P00053	0.489281	1.6059912	54	0.9835032	4378
75%8862803	0.22110337	0.6415276	19	0.9835243	9167
TRANSPORT OF MATURE MRNAS DERIVED FROM INTRONLESS TRANSCRIPTS%REACTOME DATABASE ID RELEASE 75%159234	0.20112622	0.6411719	40	0.98354447	4994
RESPONSE TO FATTY ACID%GOBP%GO:0070542	0.21102922	0.64080894	28	0.98357725	11932
FSH%NETPATH%FSH	0.21066755	0.6417134	29	0.98359483	14958
LOCOMOTORY BEHAVIOR%GOBP%GO:0007626	0.20500994	0.6401137	35	0.983606	5783
POSITIVE REGULATION OF DOUBLE-STRAND BREAK REPAIR%GOBP%GO:2000781	0.20241916	0.6419278	40	0.9836528	7575
BIOCARTA_INSULIN_PATHWAY%MSIGDB_C2%BIOCARTA_INSULIN_PATHWAY	0.23022719	0.64218277	17	0.9836849	12347
BIOCARTA_IGF1R_PATHWAY%MSIGDB_C2%BIOCARTA_IGF1R_PATHWAY	0.22995144	0.6403022	17	0.98368883	12347
CELLULAR MODIFIED AMINO ACID METABOLIC PROCESS%GOBP%GO:0006575	0.18316299	0.64246917	134	0.9837012	14712
TYPE II INTERFERON SIGNALING (IFNG)%WIKIPATHWAYS_20210110%WP619%HOMO SAPIENS	0.21741532	0.6383551	21	0.9838942	2588
VEGFR2 MEDIATED CELL PROLIFERATION%REACTOME%R-HSA-5218921.3	0.2353118	0.6373637	16	0.9841045	12905
CELLULAR SENESCENCE%GOBP%GO:0090398	0.20906137	0.6376308	29	0.98413247	3307
ALLOGRAFT REJECTION%WIKIPATHWAYS_20210110%WP2328%HOMO SAPIENS	0.48715138	1.5788015	45	0.98421293	4189
REGULATION OF RECEPTOR INTERNALIZATION%GOBP%GO:0002090	0.20328918	0.63610494	38	0.9844348	9455
NEURODEGENERATIVE DISEASES%REACTOME DATABASE ID RELEASE 75%8863678	0.22110337	0.6357307	19	0.9844709	9167
REGULATION OF AUTOPHAGY OF MITOCHONDRION%GOBP%GO:1903146	0.21186265	0.63625276	27	0.98453724	7219
NEGATIVE REGULATION OF SIGNALING RECEPTOR ACTIVITY%GOBP%GO:2000272	0.541161	1.5636604	20	0.9846986	4988
DIGESTION%GOBP%GO:0007586	0.20019037	0.63480663	38	0.98481727	10349
BRANCHED-CHAIN AMINO ACID METABOLIC PROCESS%GOBP%GO:0009081	0.2105696	0.6343881	24	0.9848786	9777
BIOCARTA_NFKB_PATHWAY%MSIGDB_C2%BIOCARTA_NFKB_PATHWAY	0.58967215	1.6875254	18	0.9854334	4048
INHIBITION OF EXOSOME BIOGENESIS AND SECRETION BY MANUMYCIN A IN CRPC CELLS%WIKIPATHWAYS_20210110%WP4301%HOMO SAPIENS	0.22960407	0.63119775	16	0.9857356	15680
SUBSTRATE-DEPENDENT CELL MIGRATION%GOBP%GO:0006929	0.22606848	0.6323706	16	0.98582155	15004
NEGATIVE REGULATION OF LEUKOCYTE APOPTOTIC PROCESS%GOBP%GO:2000107	0.22091632	0.6312335	19	0.98590404	10945
BONE MINERALIZATION%GOBP%GO:0030282	0.2240793	0.63173884	17	0.9859979	5131
SMOOTHENED SIGNALING PATHWAY%GOBP%GO:0007224	0.20270777	0.6313918	33	0.9860025	11474
OSTEOPONTIN-MEDIATED EVENTS%PATHWAY INTERACTION DATABASE NCI-NATURE CURATED DATA%OSTEOPONTIN-MEDIATED EVENTS	0.5371482	1.6259669	26	0.98656297	4179
SUPEROXIDE METABOLIC PROCESS%GOBP%GO:0006801	0.21692719	0.6292226	20	0.9866447	10652
REGULATION OF HOMOTYPIC CELL-CELL ADHESION%GOBP%GO:0034110	0.55152935	1.5817746	19	0.9867146	3153
ASSEMBLY OF COLLAGEN FIBRILS AND OTHER MULTIMERIC STRUCTURES%REACTOME DATABASE ID RELEASE 75%2022090	0.19126007	0.6283023	52	0.9867777	15121
FOCAL ADHESION ASSEMBLY%GOBP%GO:0048041	0.5293068	1.5566506	20	0.9867945	6547
CELLULAR RESPONSE TO AMYLOID-BETA%GOBP%GO:1904646	0.20627713	0.62792194	30	0.9868064	8541
POSITIVE REGULATION OF PROTEIN DEPHOSPHORYLATION%GOBP%GO:0035307	0.20040199	0.6284636	34	0.98687464	11534
PID_P38_ALPHA_BETA_DOWNSTREAM_PATHWAY%MSIGDB_C2%PID_P38_ALPHA_BETA_DOWNSTREAM_PATHWAY	0.20645845	0.6258412	27	0.98772275	3187
GLYCOPROTEIN CATABOLIC PROCESS%GOBP%GO:0006516	0.2175682	0.62530905	20	0.98781544	7005
MAP2K AND MAPK ACTIVATION%REACTOME DATABASE ID RELEASE 75%5674135	0.20211498	0.621567	32	0.98828954	4202
ADRENALINE AND NORADRENALINE BIOSYNTHESIS%PANTHER PATHWAY%P00001	0.20456323	0.61637974	24	0.98835707	15210
NICOTINE PHARMACODYNAMICS PATHWAY%PANTHER PATHWAY%P06587	0.20119545	0.6217848	31	0.9883626	5169
SYNTHESIS OF BILE ACIDS AND BILE SALTS VIA 7ALPHA-HYDROXYCHOLESTEROL%REACTOME DATABASE ID RELEASE 75%193368	0.2184226	0.61673605	18	0.98836374	5156
DOPAMINE RECEPTOR SIGNALING PATHWAY%GOBP%GO:0007212	0.22034009	0.6227517	18	0.98842007	9405
MONOAMINE GPCRS%WIKIPATHWAYS_20210110%WP58%HOMO SAPIENS	0.21972899	0.6229672	18	0.98849165	8114
DISEASES ASSOCIATED WITH N-GLYCOSYLATION OF PROTEINS%REACTOME DATABASE ID RELEASE 75%3781860	0.22009593	0.6204679	17	0.98850745	518
FOREBRAIN NEURON DIFFERENTIATION%GOBP%GO:0021879	0.20864941	0.62184924	21	0.988519	3770
REGULATION OF TP53 ACTIVITY%REACTOME%R-HSA-5633007.3	0.17466657	0.61675125	154	0.98854345	15588
REGULATION OF TP53 EXPRESSION AND DEGRADATION%REACTOME%R-HSA-6806003.2	0.19810204	0.62075216	37	0.98855406	14021
DETECTION OF MECHANICAL STIMULUS%GOBP%GO:0050982	0.21382634	0.62317115	23	0.9885715	12565
SNRNA PROCESSING%GOBP%GO:0016180	0.20914888	0.6233287	25	0.9886781	17581
SNORNA METABOLIC PROCESS%GOBP%GO:0016074	0.22037028	0.62190235	17	0.98867863	8438
REGULATION OF LEUKOCYTE APOPTOTIC PROCESS%GOBP%GO:2000106	0.19316626	0.61684537	39	0.98868215	16548
BIOCARTA_IL6_PATHWAY%MSIGDB_C2%BIOCARTA_IL6_PATHWAY	0.21891958	0.6108515	18	0.9887243	13890
ERK MAPK TARGETS%REACTOME%R-HSA-198753.1	0.21271272	0.61692816	22	0.9888273	10440
BUTYRATE RESPONSE FACTOR 1 (BRF1) BINDS AND DESTABILIZES MRNA%REACTOME%R-HSA-450385.2	0.21887596	0.61766946	17	0.9888324	16930
REGULATION OF ADHERENS JUNCTION ORGANIZATION%GOBP%GO:1903391	0.18582018	0.614739	60	0.9888348	12086
BIOCARTA_RAC1_PATHWAY%MSIGDB_C2%BIOCARTA_RAC1_PATHWAY	0.22300857	0.6113745	16	0.9888495	10579
SYNTHESIS OF BILE ACIDS AND BILE SALTS%REACTOME DATABASE ID RELEASE 75%192105	0.20337538	0.61505234	28	0.98885816	5156
DNA DAMAGE TELOMERE STRESS INDUCED SENESCENCE%REACTOME DATABASE ID RELEASE 75%2559586	0.18708152	0.61096674	49	0.98886126	12708
POSITIVE REGULATION OF NEUTROPHIL MIGRATION%GOBP%GO:1902624	0.21923424	0.61904	18	0.9888813	17879
PROTEOGLYCAN METABOLIC PROCESS%GOBP%GO:0006029	0.18247135	0.617074	69	0.98894584	12209
ENDOGENOUS STEROLS%REACTOME%R-HSA-211976.4	0.20904048	0.6178127	20	0.9889466	15391
SULFUR COMPOUND TRANSPORT%GOBP%GO:0072348	0.19650127	0.61139673	33	0.98902416	4390
ACTIVATION OF BAD AND TRANSLOCATION TO MITOCHONDRIA%REACTOME%R-HSA-111447.2	0.22805019	0.6190719	15	0.9890522	6342
REGULATION OF TRIGLYCERIDE METABOLIC PROCESS%GOBP%GO:0090207	0.21415533	0.6183062	22	0.98907155	14656
BIOCARTA_PTDINS_PATHWAY%MSIGDB_C2%BIOCARTA_PTDINS_PATHWAY	0.21464512	0.61383176	19	0.98911184	3509
POTASSIUM ION IMPORT ACROSS PLASMA MEMBRANE%GOBP%GO:1990573	0.19781807	0.61781937	35	0.9891308	10168
TELOMERE C-STRAND (LAGGING STRAND) SYNTHESIS%REACTOME DATABASE ID RELEASE 75%174417	0.19717081	0.6133114	34	0.9891896	11716
POTASSIUM ION IMPORT%GOBP%GO:0010107	0.19781807	0.61140925	35	0.9892047	10168
PROTON TRANSMEMBRANE TRANSPORT%GOBP%GO:1902600	0.19061589	0.61269087	49	0.9893011	5819
GLOBO SPHINGOLIPID METABOLISM%WIKIPATHWAYS_20210110%WP1424%HOMO SAPIENS	0.2229548	0.61151403	15	0.98933434	4449
METAPATHWAY BIOTRANSFORMATION PHASE I AND II%WIKIPATHWAYS_20210110%WP702%HOMO SAPIENS	0.18072791	0.6115561	89	0.9894991	4847
ENDOPLASMIC RETICULUM CALCIUM ION HOMEOSTASIS%GOBP%GO:0032469	0.55165577	1.5831265	20	0.98951375	4182
JAK STAT SIGNALING PATHWAY%PANTHER PATHWAY%P00038	0.22327025	0.6079202	15	0.9895918	13296
PROCESSIVE SYNTHESIS ON THE C-STRAND OF THE TELOMERE%REACTOME DATABASE ID RELEASE 75%174414	0.21314746	0.60864985	19	0.9896145	17393
BIOCARTA_BIOPEPTIDES_PATHWAY%MSIGDB_C2%BIOCARTA_BIOPEPTIDES_PATHWAY	0.20919004	0.6070411	20	0.98964864	12347
CHROMOSOME MAINTENANCE%REACTOME DATABASE ID RELEASE 75%73886	0.17623678	0.6115849	110	0.98967123	6033
SRP-DEPENDENT COTRANSLATIONAL PROTEIN TARGETING TO MEMBRANE%GOBP%GO:0006614	0.17720059	0.6080519	89	0.9897177	8277
REGULATION OF TYPE I INTERFERON-MEDIATED SIGNALING PATHWAY%GOBP%GO:0060338	0.20228206	0.60725456	25	0.9897279	1297
REGULATION OF DNA-DEPENDENT DNA REPLICATION%GOBP%GO:0090329	0.18887722	0.604685	42	0.9900305	13064
TRIGLYCERIDE METABOLIC PROCESS%GOBP%GO:0006641	0.19251816	0.6050203	40	0.9900611	18946
POSITIVE REGULATION OF EXTRACELLULAR MATRIX ORGANIZATION%GOBP%GO:1903055	0.21018606	0.6056932	20	0.9901111	15386
BIOCARTA_EDG1_PATHWAY%MSIGDB_C2%BIOCARTA_EDG1_PATHWAY	0.20938028	0.605297	17	0.9901182	10110
NONSENSE MEDIATED DECAY (NMD) INDEPENDENT OF THE EXON JUNCTION COMPLEX (EJC)%REACTOME%R-HSA-975956.1	0.17278557	0.59177405	92	0.9906904	8277
REGULATION OF MAST CELL ACTIVATION%GOBP%GO:0033003	0.19841765	0.5920058	23	0.99076843	14775
ACYL CHAIN REMODELLING OF PE%REACTOME DATABASE ID RELEASE 75%1482839	0.2058682	0.6022495	20	0.99076897	11742
INTERCONVERSION OF NUCLEOTIDE DI- AND TRIPHOSPHATES%REACTOME DATABASE ID RELEASE 75%499943	0.1996965	0.60182667	26	0.990789	8749
REGULATION OF ALPHA-BETA T CELL PROLIFERATION%GOBP%GO:0046640	0.562445	1.5719962	17	0.9908255	2681
TRIGLYCERIDE BIOSYNTHETIC PROCESS%GOBP%GO:0019432	0.21760929	0.59006596	15	0.9908964	18513
TELOMERE MAINTENANCE VIA SEMI-CONSERVATIVE REPLICATION%GOBP%GO:0032201	0.19716278	0.59212554	25	0.9909014	11716
MAPKAP KINASES	0.20553172	0.60230523	21	0.9909279	6462
AROMATIC AMINO ACID FAMILY METABOLIC PROCESS%GOBP%GO:0009072	0.19948436	0.5908231	24	0.9909288	6438
CARDIAC HYPERTROPHIC RESPONSE%WIKIPATHWAYS_20210110%WP2795%HOMO SAPIENS	0.4905395	1.5704867	45	0.9909718	4523
REGULATION OF PROTON TRANSPORT%GOBP%GO:0010155	0.21513075	0.5901925	15	0.9910237	926
RESPONSE OF EIF2AK4 (GCN2) TO AMINO ACID DEFICIENCY%REACTOME DATABASE ID RELEASE 75%9633012	0.17188726	0.59219164	98	0.9910529	8277
HISTONE MRNA METABOLIC PROCESS%GOBP%GO:0008334	0.19415344	0.5890356	28	0.99115217	11934
FATTY-ACYL-COA BIOSYNTHETIC PROCESS%GOBP%GO:0046949	0.19160853	0.5868406	27	0.9911684	8103
POSITIVE REGULATION OF LEUKOCYTE DIFFERENTIATION%GOBP%GO:1902107	0.17417952	0.5877	81	0.99116945	8693
POSITIVE REGULATION OF CELL-MATRIX ADHESION%GOBP%GO:0001954	0.18698505	0.587216	36	0.9911887	8541
VESICLE TRANSPORT ALONG MICROTUBULE%GOBP%GO:0047496	0.18774113	0.58808535	37	0.9911932	11957
NEGATIVE REGULATION OF SPROUTING ANGIOGENESIS%GOBP%GO:1903671	0.19868393	0.59224784	24	0.99121517	5225
COTRANSLATIONAL PROTEIN TARGETING TO MEMBRANE%GOBP%GO:0006613	0.17017251	0.5882954	96	0.9912879	9500
TRANSLESION SYNTHESIS%GOBP%GO:0019985	0.18469961	0.5933397	41	0.9912952	17672
REGULATION OF NUCLEAR-TRANSCRIBED MRNA POLY(A) TAIL SHORTENING%GOBP%GO:0060211	0.21732435	0.5937187	15	0.99130815	4424
DOLICHOL-LINKED OLIGOSACCHARIDE BIOSYNTHETIC PROCESS%GOBP%GO:0006488	0.21154092	0.5924229	17	0.9913248	6418
VIRAL TRANSCRIPTION%GOBP%GO:0019083	0.1721696	0.5947209	114	0.99143195	8514
NEGATIVE REGULATION OF INTERFERON-GAMMA PRODUCTION%GOBP%GO:0032689	0.20538364	0.59425	19	0.99145454	16131
DNA BIOSYNTHETIC PROCESS%GOBP%GO:0071897	0.18129653	0.59793586	67	0.9914563	14269
REGULATION OF TP53 DEGRADATION%REACTOME DATABASE ID RELEASE 75%6804757	0.19140269	0.59544706	36	0.99147755	14021
HIPPOCAMPUS DEVELOPMENT%GOBP%GO:0021766	0.20684645	0.5924925	19	0.9914799	10931
TRANSCRIPTIONAL REGULATION OF PLURIPOTENT STEM CELLS%REACTOME%R-HSA-452723.2	0.21523987	0.59374774	16	0.9914809	7568

PID_IL2_STAT5_PATHWAY%MSIGDB_C2%PID_IL2_STAT5_PATHWAY	0.21024294	0.59495246	18	0.99151766	4020
SELENOCYSTEINE SYNTHESIS%REACTOME DATABASE ID RELEASE 75%2408557	0.17420943	0.5969758	90	0.9915226	14816
ISOPRENOID METABOLIC PROCESS%GOBP%GO:0006720	0.17625684	0.5973637	84	0.9915402	14907
STAT5	0.20508543	0.5850329	20	0.9915427	14204
IFN-GAMMA%IOB%IFN-GAMMA	0.19435276	0.5980317	32	0.9915967	2710
INTRACILIARY TRANSPORT%GOBP%GO:0042073	0.18468678	0.5983932	47	0.9916144	5707
SIGNALING BY WNT IN CANCER%REACTOME DATABASE ID RELEASE 75%4791275	0.19291438	0.5955028	31	0.99163765	6869
POSITIVE REGULATION OF PHAGOCYTOSIS%GOBP%GO:0050766	0.18892871	0.5991213	37	0.99165183	11179
DORSAL/VENTRAL PATTERN FORMATION%GOBP%GO:0009953	0.19283357	0.5986935	31	0.9916646	13507
REGULATION OF GLYCOPROTEIN BIOSYNTHETIC PROCESS%GOBP%GO:0010559	0.19709553	0.5994957	29	0.9916711	15100
NEGATIVE REGULATION OF ALPHA-BETA T CELL DIFFERENTIATION%GOBP%GO:0046639	0.20892864	0.58373314	16	0.99171126	888
POSITIVE REGULATION OF ATPASE ACTIVITY%GOBP%GO:0032781	0.18645445	0.58506525	37	0.99171644	4139
POSTSYNAPSE ASSEMBLY%GOBP%GO:0099068	0.206961	0.58409905	16	0.9917448	6482
AMINO ACID METABOLISM%WIKIPATHWAYS_20210110%WP3925%HOMO SAPIENS	0.1821363	0.59558266	51	0.99178725	12644
SIGNALING BY PDGFR IN DISEASE%REACTOME DATABASE ID RELEASE 75%9671555	0.20483837	0.5830779	17	0.991789	12347
EMBRYONIC DIGESTIVE TRACT DEVELOPMENT%GOBP%GO:0048566	0.20727465	0.5957616	19	0.9918918	9324
SUMOYLATION OF INTRACELLULAR RECEPTORS%REACTOME%R-HSA-4090294.3	0.19338702	0.5691519	22	0.99192506	2077
POSITIVE REGULATION OF DEPHOSPHORYLATION%GOBP%GO:0035306	0.17844167	0.5659584	40	0.9920419	6576
PROTEIN HETEROOLIGOMERIZATION%GOBP%GO:0051291	0.20319045	0.5702844	16	0.992048	12883
TRANSCRIPTIONAL REGULATION BY MECP2%REACTOME%R-HSA-8986944.2	0.1725768	0.56916696	56	0.99210376	15490
NEGATIVE REGULATION OF ANION TRANSPORT%GOBP%GO:1903792	0.19889763	0.56812453	19	0.9921392	15150
REGULATION OF SYNAPTIC TRANSMISSION, GLUTAMATERGIC%GOBP%GO:0051966	0.17583227	0.5660764	43	0.9921758	12135
DNA SYNTHESIS INVOLVED IN DNA REPAIR%GOBP%GO:0000731	0.1744333	0.56746286	48	0.9922077	17750
REGULATION OF B CELL RECEPTOR SIGNALING PATHWAY%GOBP%GO:0050855	0.20729895	0.5703024	15	0.99222636	10380
PID_S1P_S1P1_PATHWAY%MSIGDB_C2%PID_S1P_S1P1_PATHWAY	0.2086537	0.57068896	15	0.9922633	15654
IL3-MEDIATED SIGNALING EVENTS%PATHWAY INTERACTION DATABASE NCI-NATURE CURATED DATA%IL3-MEDIATED SIGNALING EVENTS	0.19271518	0.5691865	22	0.99227905	14253
REGULATION OF GLYCOGEN BIOSYNTHETIC PROCESS%GOBP%GO:0005979	0.19439851	0.566157	23	0.99232763	17029
QUINONE METABOLIC PROCESS%GOBP%GO:1901661	0.18618177	0.566596	27	0.99235016	16622
FILOPODIUM ASSEMBLY%GOBP%GO:0046847	0.20628713	0.5709016	15	0.99236345	17040
NEGATIVE REGULATION OF TELOMERE MAINTENANCE%GOBP%GO:0032205	0.18100686	0.56261	34	0.9925241	12221
ALK1 SIGNALING EVENTS%PATHWAY INTERACTION DATABASE NCI-NATURE CURATED DATA%ALK1 SIGNALING EVENTS	0.19406815	0.5709534	25	0.99252516	7491
INTRASPECIES INTERACTION BETWEEN ORGANISMS%GOBP%GO:0051703	0.18837158	0.56351596	24	0.9925588	15452
RA BIOSYNTHESIS PATHWAY%REACTOME%R-HSA-5365859.2	0.20089616	0.57279223	19	0.9925643	14483
GLYCOSYL COMPOUND BIOSYNTHETIC PROCESS%GOBP%GO:1901659	0.19051468	0.5712266	28	0.992608	4355
BILE ACID AND BILE SALT METABOLISM%REACTOME%R-HSA-194068.1	0.1836376	0.57313174	32	0.99261916	10904
CELLULAR RESPONSE TO NERVE GROWTH FACTOR STIMULUS%GOBP%GO:1990090	0.18890022	0.5628216	22	0.9926302	7992
REGULATION OF HEART MORPHOGENESIS%GOBP%GO:2000826	0.18347998	0.5637995	27	0.9926475	16936
POSITIVE REGULATION OF LEUKOCYTE APOPTOTIC PROCESS%GOBP%GO:2000108	0.20478494	0.57341987	17	0.9926958	16548
REGULATION OF DNA REPLICATION%GOBP%GO:0006275	0.16996199	0.57429075	83	0.9927264	15247
ALPHA AND P38-BETA	0.18124887	0.5718881	35	0.9927279	15828
REGULATION OF VASCULAR ENDOTHELIAL GROWTH FACTOR RECEPTOR SIGNALING PATHWAY%GOBP%GO:0030947	0.18760799	0.56140566	22	0.99277097	13565
OLIGOSACCHARIDE-LIPID INTERMEDIATE BIOSYNTHETIC PROCESS%GOBP%GO:0006490	0.1974157	0.57128215	18	0.99277186	6418
BDNF%IOB%BDNF	0.17966929	0.5735753	42	0.99282056	10689
P53 PATHWAY FEEDBACK LOOPS 2%PANTHER PATHWAY%P04398	0.17938186	0.5745197	41	0.99282384	6040
TELOMERE MAINTENANCE%REACTOME DATABASE ID RELEASE 75%157579	0.16718657	0.57485247	83	0.9928804	13165
NEGATIVE REGULATION OF LYMPHOCYTE DIFFERENTIATION%GOBP%GO:0045620	0.1917539	0.5776505	27	0.992904	6752
REGULATION OF GLUCAN BIOSYNTHETIC PROCESS%GOBP%GO:0010962	0.19439851	0.5756068	23	0.9929674	17029
NICOTINATE METABOLISM%REACTOME%R-HSA-196807.3	0.51509684	1.558727	28	0.9929723	2361
LIPID TRANSLOCATION%GOBP%GO:0034204	0.19529904	0.57784855	22	0.9930156	15790
MAMMARY GLAND EPITHELIUM DEVELOPMENT%GOBP%GO:0061180	0.20523556	0.57497364	17	0.9930169	7542
PID_PTP1B_PATHWAY%MSIGDB_C2%PID_PTP1B_PATHWAY	0.18302876	0.55857444	31	0.99303174	11679
NEGATIVE REGULATION OF EPITHELIAL CELL PROLIFERATION%GOBP%GO:0050680	0.17125133	0.576858	78	0.99303806	6688
REGULATION OF TRANSCRIPTION INVOLVED IN G1/S TRANSITION OF MITOTIC CELL CYCLE%GOBP%GO:0000083	0.1850538	0.5780979	30	0.9930944	4548
DISRUPTION OF POSTSYNAPTIC SIGNALLING BY CNV%WIKIPATHWAYS_20210110%WP4875%HOMO SAPIENS	0.19248663	0.57577205	24	0.99309546	7588
POSITIVE REGULATION OF BONE MINERALIZATION%GOBP%GO:0030501	0.18684559	0.5784043	33	0.9931531	9803
RESPONSE TO NERVE GROWTH FACTOR%GOBP%GO:1990089	0.18890022	0.55859387	22	0.99320817	7992
TERMINATION OF TRANSLESION DNA SYNTHESIS%REACTOME DATABASE ID RELEASE 75%5656169	0.18410434	0.5759718	32	0.99320847	17805
NEGATIVE REGULATION OF RESPONSE TO OXIDATIVE STRESS%GOBP%GO:1902883	0.17586742	0.55889535	36	0.9932851	6947
PEPTIDE CHAIN ELONGATION%REACTOME%R-HSA-156902.2	0.16921635	0.5784999	86	0.99330133	14816
MODULATION BY VIRUS OF HOST PROCESS%GOBP%GO:0019054	0.1940379	0.5572656	20	0.99330664	5367
AMINE LIGAND-BINDING RECEPTORS%REACTOME DATABASE ID RELEASE 75%375280	0.20111531	0.5787572	21	0.9933843	15377
SOCIAL BEHAVIOR%GOBP%GO:0035176	0.18837158	0.55909795	24	0.9933968	15452
CELLULAR RESPONSE TO REACTIVE OXYGEN SPECIES%GOBP%GO:0034614	0.16353668	0.55551475	79	0.9935406	13925
CELLULAR IRON ION HOMEOSTASIS%GOBP%GO:0006879	0.17059164	0.5556608	50	0.9936752	3587
AXON GUIDANCE MEDIATED BY NETRIN%PANTHER PATHWAY%P00009	0.57582337	1.7404208	29	0.99377227	5844
PCNA-DEPENDENT LONG PATCH BASE EXCISION REPAIR%REACTOME DATABASE ID RELEASE 75%5651801	0.19079678	0.55338794	21	0.9940746	18146
PID_IL6_7_PATHWAY%MSIGDB_C2%PID_IL6_7_PATHWAY	0.17610376	0.55153984	34	0.99431306	13890
DETECTION OF LIGHT STIMULUS%GOBP%GO:0009583	0.1867031	0.55203503	22	0.99433637	14907
EUKARYOTIC TRANSLATION ELONGATION%REACTOME%R-HSA-156842.2	0.16078033	0.5488585	91	0.9944855	14816
CORONARY VASCULATURE DEVELOPMENT%GOBP%GO:0060976	0.1998706	0.55048424	16	0.99448884	5930
POSITIVE REGULATION OF PROTEIN ACETYLATION%GOBP%GO:1901985	0.17459516	0.54989415	37	0.9945112	18025
REGULATION OF MECP2 EXPRESSION AND ACTIVITY%REACTOME%R-HSA-9022692.1	0.17866421	0.54895693	29	0.99463844	15490
GLUTAMATE BINDING, ACTIVATION OF AMPA RECEPTORS AND SYNAPTIC PLASTICITY%REACTOME DATABASE ID RELEASE 75%399721	0.17256778	0.51845866	26	0.99471456	17342
ETHANOL EFFECTS ON HISTONE MODIFICATIONS%WIKIPATHWAYS_20210110%WP3996%HOMO SAPIENS	0.18328448	0.5474524	23	0.99475485	5591
TRK RECEPTOR SIGNALING MEDIATED BY PI3K AND PLC-GAMMA%PATHWAY INTERACTION DATABASE NCI-NATURE CURATED DATA%TRK RECEPTOR SIGNALING MEDIATED BY PI3K AND PLC-GAMMA	0.16284242	0.5185762	36	0.99486846	14132
VIRAL MRNA TRANSLATION%REACTOME%R-HSA-192823.4	0.1617748	0.5464615	86	0.9948986	14816
HYDROGEN PEROXIDE METABOLIC PROCESS%GOBP%GO:0042743	0.17929746	0.51885235	20	0.99497366	14683
PID_PI3K_PLC_TRK_PATHWAY%MSIGDB_C2%PID_PI3K_PLC_TRK_PATHWAY	0.17885141	0.54547626	27	0.9950418	14132
TRAFFICKING OF AMPA RECEPTORS%REACTOME%R-HSA-399719.1	0.17256778	0.51985985	26	0.9950747	17342
POSITIVE REGULATION OF PEPTIDYL-LYSINE ACETYLATION%GOBP%GO:2000758	0.16931297	0.5190755	28	0.99509597	8383
POSITIVE REGULATION OF ADHERENS JUNCTION ORGANIZATION%GOBP%GO:1903393	0.18058847	0.5435119	24	0.9951222	10440
HETEROTYPIC CELL-CELL ADHESION%GOBP%GO:0034113	0.56326485	1.656654	21	0.9951396	5257
METABOLISM OF COFACTORS%REACTOME DATABASE ID RELEASE 75%8978934	0.18865839	0.5393043	18	0.99516463	13497
REGULATION OF CHONDROCYTE DIFFERENTIATION%GOBP%GO:0032330	0.17043336	0.5201822	27	0.9951689	15998
POSITIVE CHEMOTAXIS%GOBP%GO:0050918	0.1976823	0.5439138	16	0.9951768	13855
SCHWANN CELL DIFFERENTIATION%GOBP%GO:0014037	0.19088575	0.52076894	15	0.9951893	1067
RESOLUTION OF AP SITES VIA THE MULTIPLE-NUCLEOTIDE PATCH REPLACEMENT PATHWAY%REACTOME%R-HSA-110373.2	0.17967246	0.54418015	25	0.9952701	18146
BIOCARTA_G1_PATHWAY%MSIGDB_C2%BIOCARTA_G1_PATHWAY	0.18439725	0.52116346	19	0.99527025	4489
POLYMERASE SWITCHING ON THE C-STRAND OF THE TELOMERE%REACTOME DATABASE ID RELEASE 75%174411	0.1776318	0.5395629	26	0.9952713	4257
REGULATION OF MITOCHONDRIAL TRANSLATION%GOBP%GO:0070129	0.17890704	0.5214054	22	0.995394	12254
RETINOID METABOLIC PROCESS%GOBP%GO:0001523	0.16123033	0.5397195	64	0.9954119	14907
REGULATION OF VASCULAR ENDOTHELIAL GROWTH FACTOR SIGNALING PATHWAY%GOBP%GO:1900746	0.19161317	0.53770167	17	0.99545866	14138
TRANSLESION SYNTHESIS BY Y FAMILY DNA POLYMERASES BYPASSES LESIONS ON DNA TEMPLATE%REACTOME%R-HSA-110313.1	0.16041733	0.5098477	39	0.99546695	17805
EPIDERMAL GROWTH FACTOR RECEPTOR SIGNALING PATHWAY%GOBP%GO:0007173	0.16008642	0.5053663	39	0.9954684	9727
ALTERNATIVE MRNA SPLICING, VIA SPLICEOSOME%GOBP%GO:0000380	0.18715984	0.51421326	15	0.9954686	10350
PHOSPHOLIPID TRANSPORT%GOBP%GO:0015914	0.15735222	0.5068201	48	0.9954793	10931
PURINERGIC SIGNALING%WIKIPATHWAYS_20210110%WP4900%HOMO SAPIENS	0.1801977	0.51341623	19	0.99548316	564
MODULATION OF EXCITATORY POSTSYNAPTIC POTENTIAL%GOBP%GO:0098815	0.17568707	0.5418037	27	0.9954848	6588
LYMPHATIC ENDOTHELIUM	0.16757908	0.5074721	25	0.9955015	10095
SIGNAL TRANSDUCTION OF S1P RECEPTOR%WIKIPATHWAYS_20210110%WP26%HOMO SAPIENS	0.18067838	0.5043407	18	0.9955227	15654
REGULATION OF ESTABLISHMENT OR MAINTENANCE OF CELL POLARITY%GOBP%GO:0032878	0.55165535	1.5639678	19	0.9955402	3063
REGULATION OF CARBOHYDRATE BIOSYNTHETIC PROCESS%GOBP%GO:0043255	0.16260679	0.53682244	57	0.9955461	13390
PATHWAY	0.181081	0.5214624	19	0.9955609	15654
DRUG TRANSMEMBRANE TRANSPORT%GOBP%GO:0006855	0.16521712	0.5398322	56	0.9955629	14196
REGULATION OF POLYSACCHARIDE BIOSYNTHETIC PROCESS%GOBP%GO:0032885	0.16792977	0.5056942	29	0.99557227	11546
CELLULAR TRANSITION METAL ION HOMEOSTASIS%GOBP%GO:0046916	0.14908902	0.50791013	85	0.99557626	3587
NEGATIVE REGULATION OF DNA REPLICATION%GOBP%GO:0008156	0.17755623	0.5409488	28	0.9955842	10866
GASTRIN-CREB SIGNALLING PATHWAY VIA PKC AND MAPK%REACTOME DATABASE ID RELEASE 75%881907	0.18518233	0.5100485	16	0.99559647	14484
RESPONSE TO AMYLOID-BETA%GOBP%GO:1904645	0.16241539	0.5143313	36	0.9956194	8687
RESPONSE TO CHOLESTEROL%GOBP%GO:0070723	0.18944769	0.52186316	16	0.99564296	881
BIOCARTA_P38MAPK_PATHWAY%MSIGDB_C2%BIOCARTA_P38MAPK_PATHWAY	0.16914906	0.5082409	28	0.9956758	1237
TELENCEPHALON CELL MIGRATION%GOBP%GO:0022029	0.17112131	0.49566394	22	0.9957176	2097
REGULATION OF OXIDATIVE STRESS-INDUCED CELL DEATH%GOBP%GO:1903201	0.16645862	0.53991836	46	0.9957204	1532
PNAT%PANTHER PATHWAY%P05912	0.15032084	0.48972723	51	0.99572223	15654
PID_LYMPH_ANGIOGENESIS_PATHWAY%MSIGDB_C2%PID_LYMPH_ANGIOGENESIS_PATHWAY	0.17498343	0.51011807	22	0.9957613	10095
ODONTOGENESIS OF DENTIN-CONTAINING TOOTH%GOBP%GO:0042475	0.16955033	0.5018379	22	0.9957667	6684
REGULATION OF MEMBRANE LIPID DISTRIBUTION%GOBP%GO:0097035	0.16030997	0.51068234	41	0.9958054	16087
REGULATION OF MYOTUBE DIFFERENTIATION%GOBP%GO:0010830	0.1723039	0.52189595	28	0.9958146	14766
ALPHA ADRENERGIC RECEPTOR SIGNALING PATHWAY%PANTHER PATHWAY%P00002	0.16700825	0.49677414	22	0.99583364	15529
POSITIVE REGULATION OF MYELOID LEUKOCYTE DIFFERENTIATION%GOBP%GO:0002763	0.16416554	0.49584827	25	0.9958591	4523
PID_IL23_PATHWAY%MSIGDB_C2%PID_IL23_PATHWAY	0.16955388	0.4998338	21	0.99586457	13130
NEURONAL ACTION POTENTIAL%GOBP%GO:0019228	0.1610816	0.465176	20	0.99586487	3847
TRANSMISSION OF NERVE IMPULSE%GOBP%GO:0019226	0.16021077	0.5021557	33	0.9958688	14382
POSITIVE REGULATION OF MITOCHONDRIAL TRANSLATION%GOBP%GO:0070131	0.17851636	0.48986176	15	0.9958755	15425
NEGATIVE REGULATION OF GTPASE ACTIVITY%GOBP%GO:0034260	0.16497369	0.48366764	20	0.99590176	5279
POSITIVE REGULATION OF ANIMAL ORGAN MORPHOGENESIS%GOBP%GO:0110110	0.16285779	0.5229055	44	0.995909	14766

NEUTRAL LIPID CATABOLIC PROCESS%GOBP%GO:0046461	0.17596738	0.52673846	24	0.99592304	19358
RESPONSE TO ESTRADIOL%GOBP%GO:0032355	0.1603072	0.4979441	32	0.9959341	10989
LIMBIC SYSTEM DEVELOPMENT%GOBP%GO:0021761	0.17557895	0.5331189	27	0.9959353	10931
IRON ION HOMEOSTASIS%GOBP%GO:0055072	0.15166566	0.49867055	54	0.9959505	3587
TP53 REGULATES METABOLIC GENES%REACTOME DATABASE ID RELEASE 75%5628897	0.15494657	0.5220575	82	0.9959528	9623
SYNAPTIC VESICLE PATHWAY%WIKIPATHWAYS_20210110%WP2267%HOMO SAPIENS	0.1604465	0.51081395	40	0.99595803	16686
NEGATIVE REGULATION OF OXIDATIVE STRESS-INDUCED CELL DEATH%GOBP%GO:1903202	0.15772137	0.49700907	34	0.9959622	6947
POSITIVE REGULATION OF FAT CELL DIFFERENTIATION%GOBP%GO:0045600	0.1486191	0.4840441	47	0.9960073	17054
CHOLESTEROL BIOSYNTHESIS%REACTOME DATABASE ID RELEASE 75%191273	0.16405538	0.48572114	22	0.996024	15391
NEGATIVE REGULATION OF CELLULAR RESPONSE TO OXIDATIVE STRESS%GOBP%GO:1900408	0.15772137	0.4900026	34	0.99602705	6947
ACYLGLYCEROL CATABOLIC PROCESS%GOBP%GO:0046464	0.17596738	0.5270147	24	0.9960289	19358
DITERPENOID METABOLIC PROCESS%GOBP%GO:0016101	0.14702179	0.49086055	66	0.99603075	14907
DIENCEPHALON DEVELOPMENT%GOBP%GO:0021536	0.16048567	0.46097916	20	0.9960319	17102
MODULATION BY SYMBIONT OF HOST CELLULAR PROCESS%GOBP%GO:0044068	0.15638076	0.46526247	22	0.99603194	18529
PIGMENT CELL DIFFERENTIATION%GOBP%GO:0050931	0.17507593	0.49986756	20	0.99603784	10124
RHO GTPASES ACTIVATE CIT%REACTOME%R-HSA-5625900.2	0.16950983	0.47043064	17	0.99603856	16291
POSITIVE REGULATION OF HISTONE ACETYLATION%GOBP%GO:0035066	0.15640093	0.45981282	23	0.99604017	5917
CELLULAR RESPONSE TO TOXIC SUBSTANCE%GOBP%GO:0097237	0.13826431	0.4724839	81	0.99604285	10600
DOPAMINE METABOLIC PROCESS%GOBP%GO:0042417	0.18856373	0.5339059	17	0.9960618	14962
EXTENSION OF TELOMERES%REACTOME DATABASE ID RELEASE 75%180786	0.14384982	0.46929514	50	0.9960645	17393
ESTABLISHMENT OR MAINTENANCE OF MONOPOLAR CELL POLARITY%GOBP%GO:0061339	0.16350697	0.45744544	17	0.9960673	13454
GLUTAMATE RECEPTOR SIGNALING PATHWAY%GOBP%GO:0007215	0.15644348	0.48715013	35	0.9960795	9854
PERIPHERAL NERVOUS SYSTEM DEVELOPMENT%GOBP%GO:0007422	0.16919905	0.5332362	39	0.99608266	1609
RESPONSE TO HYDROGEN PEROXIDE%GOBP%GO:0042542	0.14868487	0.48456037	49	0.9960839	9855
PID_TGFBR_PATHWAY%MSIGDB_C2%PID_TGFBR_PATHWAY	0.16782829	0.522908	34	0.99609095	7435
ZINC HOMEOSTASIS%WIKIPATHWAYS_20210110%WP3529%HOMO SAPIENS	0.15388274	0.4616975	27	0.996091	13097
INACTIVATION OF MAPK ACTIVITY%GOBP%GO:0000188	0.16732514	0.493113	22	0.9960927	10994
POSITIVE REGULATION OF RESPONSE TO BIOTIC STIMULUS%GOBP%GO:0002833	0.173439	0.52416426	25	0.99609846	12125
REGULATION OF PROTEIN SUMOYLATION%GOBP%GO:0033233	0.17485422	0.49135476	17	0.996109	4975
MITOCHONDRIAL CIV ASSEMBLY%WIKIPATHWAYS_20210110%WP4922%HOMO SAPIENS	0.15165815	0.45829907	26	0.9961106	9623
BRAIN MORPHOGENESIS%GOBP%GO:0048854	0.18391348	0.5234661	18	0.99611866	8404
REGULATION OF RUFFLE ASSEMBLY%GOBP%GO:1900027	0.1840693	0.5273338	20	0.9961222	6710
CELLULAR RESPONSE TO DSRNA%GOBP%GO:0071359	0.1780899	0.49214372	16	0.99612373	7560
PROCESSING OF INTRONLESS PRE-MRNAS%REACTOME DATABASE ID RELEASE 75%77595	0.18153238	0.5246744	19	0.9961322	2690
PID_ATM_PATHWAY%MSIGDB_C2%PID_ATM_PATHWAY	0.15807025	0.46561146	21	0.9961567	16656
PROCESSING OF CAPPED INTRONLESS PRE-MRNA%REACTOME%R-HSA-75067.2	0.17858917	0.5341648	28	0.99616194	11934
NEGATIVE REGULATION OF GLIOGENESIS%GOBP%GO:0014014	0.17237304	0.47276843	16	0.99616927	10351
EUKARYOTIC TRANSLATION TERMINATION%REACTOME%R-HSA-72764.4	0.15571967	0.53097093	90	0.99618405	8277
CYTOPLASMIC RIBOSOMAL PROTEINS%WIKIPATHWAYS_20210110%WP477%HOMO SAPIENS	0.14394099	0.48577845	81	0.99619275	14816
CARBOXYTERMINAL POST-TRANSLATIONAL MODIFICATIONS OF TUBULIN%REACTOME%R-HSA-8955332.1	0.15065947	0.47048706	35	0.9962112	16225
INTRACILIARY TRANSPORT INVOLVED IN CILIUM ASSEMBLY%GOBP%GO:0035735	0.16235554	0.52500546	38	0.9962231	16464
PROTEOGLYCAN BIOSYNTHETIC PROCESS%GOBP%GO:0030166	0.14671667	0.48115987	53	0.9962251	12209
TRANSITION METAL ION HOMEOSTASIS%GOBP%GO:0055076	0.1356135	0.46184406	93	0.9962473	3841
NCRNA 3'-END PROCESSING%GOBP%GO:0043628	0.16772427	0.5273866	37	0.9962896	16930
GLYPICAN 1 NETWORK%PATHWAY INTERACTION DATABASE NCI-NATURE CURATED DATA%GLYPICAN 1 NETWORK	0.15314683	0.44889554	21	0.99630344	15332
CARBOHYDRATE TRANSMEMBRANE TRANSPORT%GOBP%GO:0034219	0.15579891	0.45239657	21	0.9963136	12555
INNER EAR MORPHOGENESIS%GOBP%GO:0042472	0.14890443	0.4500514	28	0.9963151	7112
CLATHRIN-DEPENDENT ENDOCYTOSIS%GOBP%GO:0072583	0.15668638	0.46572953	24	0.9963185	8875
SODIUM ION HOMEOSTASIS%GOBP%GO:0055078	0.1666325	0.47281095	17	0.99634266	9949
A TETRASACCHARIDE LINKER SEQUENCE IS REQUIRED FOR GAG SYNTHESIS%REACTOME DATABASE ID RELEASE 75%1971475	0.17627674	0.53105396	26	0.9963437	11991
TERPENOID METABOLIC PROCESS%GOBP%GO:0006721	0.14207675	0.47942352	70	0.99637794	14907
PROGRAMMED NECROTIC CELL DEATH%GOBP%GO:0097300	0.15418097	0.4456979	19	0.9964032	926
ESTABLISHMENT OF APICAL/BASAL CELL POLARITY%GOBP%GO:0035089	0.17443004	0.47335207	15	0.996421	13454
ACTIVATION OF JUN KINASE ACTIVITY%GOBP%GO:0007257	0.16935334	0.52887446	31	0.99642706	1218
SUMOYLATION OF DNA METHYLATION PROTEINS%REACTOME%R-HSA-4655427.3	0.19179624	0.5274805	15	0.9964493	15992
HISTONE MRNA CATABOLIC PROCESS%GOBP%GO:0071044	0.16532889	0.45015326	15	0.9964809	7144
POSITIVE REGULATION OF BIOMINERAL TISSUE DEVELOPMENT%GOBP%GO:0070169	0.14530006	0.45245004	37	0.9964848	9803
NEGATIVE REGULATION OF DNA-DEPENDENT DNA REPLICATION%GOBP%GO:2000104	0.16590284	0.46576276	17	0.99649346	10866
NONSENSE-MEDIATED DECAY (NMD)%REACTOME%R-HSA-927802.2	0.12561886	0.4312074	112	0.99650115	13820
HEPATITIS C AND HEPATOCELLULAR CARCINOMA%WIKIPATHWAYS_20210110%WP3646%HOMO SAPIENS	0.13652965	0.4250786	34	0.9965164	11221
PID_RAS_PATHWAY%MSIGDB_C2%PID_RAS_PATHWAY	0.15346201	0.44610897	21	0.99652326	5279
IL6-MEDIATED SIGNALING EVENTS%PATHWAY INTERACTION DATABASE NCI-NATURE CURATED DATA%IL6-MEDIATED SIGNALING EVENTS	0.14886338	0.4736932	42	0.9965348	15407
NEURON APOPTOTIC PROCESS%GOBP%GO:0051402	0.16503079	0.47659522	20	0.99655515	19221
MONOSACCHARIDE TRANSMEMBRANE TRANSPORT%GOBP%GO:0015749	0.15579891	0.45312896	21	0.9965612	12555
REGULATION OF LYMPHOCYTE APOPTOTIC PROCESS%GOBP%GO:0070228	0.1428773	0.4290336	24	0.99658066	16548
ICOSANOID BIOSYNTHETIC PROCESS%GOBP%GO:0046456	0.15154882	0.47443244	33	0.9965834	3186
CHONDROCYTE DIFFERENTIATION%GOBP%GO:0002062	0.16907583	0.5289596	34	0.9965852	14256
PID_PI3KCI_AKT_PATHWAY%MSIGDB_C2%PID_PI3KCI_AKT_PATHWAY	0.17315263	0.5275488	27	0.9966133	12418
INTERLEUKIN-20 FAMILY SIGNALING%REACTOME%R-HSA-8854691.6	0.15248819	0.43150637	18	0.99664587	13967
TGF-BETA RECEPTOR SIGNALING%PATHWAY INTERACTION DATABASE NCI-NATURE CURATED DATA%TGF-BETA RECEPTOR SIGNALING	0.13100936	0.42690966	52	0.9966576	6496
BDNF-TRKB SIGNALING%WIKIPATHWAYS_20210110%WP3676%HOMO SAPIENS	0.1562119	0.4769957	29	0.9966579	14021
CELLULAR RESPONSE TO HYDROGEN PEROXIDE%GOBP%GO:0070301	0.15028583	0.47490135	36	0.99667704	1054
NEGATIVE REGULATION OF CARTILAGE DEVELOPMENT%GOBP%GO:0061037	0.15575264	0.42517626	15	0.9966857	6955
NEGATIVE REGULATION OF DNA BIOSYNTHETIC PROCESS%GOBP%GO:2000279	0.13449267	0.41834587	31	0.99670327	11925
NUCLEAR-TRANSCRIBED MRNA CATABOLIC PROCESS, NONSENSE-MEDIATED DECAY%GOBP%GO:0000184	0.12544556	0.43230996	112	0.9967311	6506
PID_P38_ALPHA_BETA_PATHWAY%MSIGDB_C2%PID_P38_ALPHA_BETA_PATHWAY	0.14682017	0.41941947	20	0.9967673	10994
NEGATIVE REGULATION OF CARBOHYDRATE METABOLIC PROCESS%GOBP%GO:0045912	0.1389921	0.43274593	33	0.9968551	9189
NEGATIVE REGULATION OF CELLULAR CARBOHYDRATE METABOLIC PROCESS%GOBP%GO:0010677	0.14226931	0.44073525	30	0.9969049	14347
SIGNALING BY BMP%REACTOME DATABASE ID RELEASE 75%201451	0.13921101	0.4194334	25	0.9969454	15132
PID_IL2_PI3K_PATHWAY%MSIGDB_C2%PID_IL2_PI3K_PATHWAY	0.14381069	0.4328844	25	0.9970162	5010
GLYCOSIDE METABOLIC PROCESS%GOBP%GO:0016137	0.15636879	0.43423358	16	0.99702007	1181
ESTABLISHMENT OF MONOPOLAR CELL POLARITY%GOBP%GO:0061162	0.15544215	0.43502605	16	0.99709773	1234
NONSENSE MEDIATED DECAY (NMD) ENHANCED BY THE EXON JUNCTION COMPLEX (EJC)%REACTOME%R-HSA-975957.1	0.12561886	0.43560073	112	0.99720377	13820
NEURON DEATH%GOBP%GO:0070997	0.14557661	0.4365786	23	0.9972594	19221
PID_IL1_PATHWAY%MSIGDB_C2%PID_IL1_PATHWAY	0.5161882	1.5790842	27	0.99752873	5609
CENTRAL NERVOUS SYSTEM PROJECTION NEURON AXONOGENESIS%GOBP%GO:0021952	0.14890398	0.40495667	15	0.99781936	6155
PKC-GAMMA CALCIUM SIGNALING PATHWAY IN ATAXIA%WIKIPATHWAYS_20210110%WP4760%HOMO SAPIENS	0.13322319	0.37725246	16	0.9983101	10289
REGULATION OF P38MAPK CASCADE%GOBP%GO:1900744	0.122678146	0.3791925	32	0.99836093	14402
EVENTS MEDIATED BY AKT	0.119503856	0.38123742	35	0.99840915	14253
AEROBIC ELECTRON TRANSPORT CHAIN%GOBP%GO:0019646	0.13106252	0.36523166	18	0.99845845	19902
FATTY ACID DERIVATIVE BIOSYNTHETIC PROCESS%GOBP%GO:1901570	0.11595786	0.3927116	69	0.99849147	5288
PID_ALPHA_SYNUCLEIN_PATHWAY%MSIGDB_C2%PID_ALPHA_SYNUCLEIN_PATHWAY	0.12597577	0.3675963	20	0.9985142	10454
BIOCARTA_CTCF_PATHWAY%MSIGDB_C2%BIOCARTA_CTCF_PATHWAY	0.1275096	0.3595148	18	0.998549	4020
NUCLEOBASE BIOSYNTHESIS%REACTOME%R-HSA-8956320.1	0.14473408	0.39400488	15	0.9985647	17154
REGULATION OF NUCLEASE ACTIVITY%GOBP%GO:0032069	0.130706	0.38144913	20	0.9985763	1113
NEGATIVE REGULATION OF ENDOTHELIAL CELL PROLIFERATION%GOBP%GO:0001937	0.12515414	0.3891714	35	0.9985773	14571
MITOCHONDRIAL ELECTRON TRANSPORT, CYTOCHROME C TO OXYGEN%GOBP%GO:0006123	0.13106252	0.3692815	18	0.99860334	19902
NEGATIVE REGULATION OF UBIQUITIN-PROTEIN TRANSFERASE ACTIVITY%GOBP%GO:0051444	0.13858694	0.38327786	15	0.99864244	3536
CENTRIOLE REPLICATION%GOBP%GO:0007099	0.1216526	0.35188156	21	0.9986848	18319
POSITIVE REGULATION OF P38MAPK CASCADE%GOBP%GO:1900745	0.12933955	0.3837471	21	0.9987907	14402
IFN-ALPHA%IOB%IFN-ALPHA	0.10748388	0.33280414	30	0.9991627	14021
VIRAL TRANSLATION%GOBP%GO:0019081	0.58154374	1.5838279	15	0.9993708	6626
INTERFERON TYPE I SIGNALING PATHWAYS%WIKIPATHWAYS_20210110%WP585%HOMO SAPIENS	0.0964496	0.30748925	46	0.999542	13503
OXIDATIVE DAMAGE%WIKIPATHWAYS_20210110%WP3941%HOMO SAPIENS	0.5959099	1.8347147	30	1	5640
ANTIGEN ACTIVATES B CELL RECEPTOR (BCR) LEADING TO GENERATION OF SECOND MESSENGERS%REACTOME DATABASE ID RELEASE 75%983695	0.60160613	1.7857169	22	1	3095
POSITIVE REGULATION OF NOTCH SIGNALING PATHWAY%GOBP%GO:0045747	0.5564413	1.7786394	40	1	1976
MIRNA REGULATION OF P53 PATHWAY IN PROSTATE CANCER%WIKIPATHWAYS_20210110%WP3982%HOMO SAPIENS	0.61174893	1.7546278	19	1	3242
HALLMARK_WNT_BETA_CATENIN_SIGNALING%MSIGDB_C2%HALLMARK_WNT_BETA_CATENIN_SIGNALING	0.575594	1.7541193	30	1	1907
FOXO-MEDIATED TRANSCRIPTION OF CELL DEATH GENES%REACTOME%R-HSA-9614657.1	0.6392342	1.7454071	15	1	1102
GENERATION OF SECOND MESSENGER MOLECULES%REACTOME DATABASE ID RELEASE 75%202433	0.55959153	1.7095668	25	1	6952
HALLMARK_PANCREAS_BETA_CELLS%MSIGDB_C2%HALLMARK_PANCREAS_BETA_CELLS	0.55703664	1.7045689	31	1	2708
1Q21.1 COPY NUMBER VARIATION SYNDROME%WIKIPATHWAYS_20210110%WP4905%HOMO SAPIENS	0.5936075	1.6975405	20	1	1510
REGULATION OF CARDIAC MUSCLE CELL CONTRACTION%GOBP%GO:0086004	0.5520348	1.692333	31	1	5034
VIRION ASSEMBLY%GOBP%GO:0019068	0.5256333	1.6879941	40	1	2983
NEGATIVE REGULATION OF BLOOD COAGULATION%GOBP%GO:0030195	0.54961926	1.6598033	27	1	3153
B CELL RECEPTOR SIGNALING PATHWAY%GOBP%GO:0050853	0.54801995	1.6583943	25	1	6379
REGULATION OF VOLTAGE-GATED CALCIUM CHANNEL ACTIVITY%GOBP%GO:1901385	0.5470349	1.6470923	25	1	5106
NEGATIVE REGULATION OF HEMOSTASIS%GOBP%GO:1900047	0.54961926	1.6451582	27	1	3153
TRANSCRIPTION PREINITIATION COMPLEX ASSEMBLY%GOBP%GO:0070897	0.60016096	1.64387	15	1	5173
P75NTR SIGNALS VIA NF-KB%REACTOME DATABASE ID RELEASE 75%193639	0.5899707	1.6372426	16	1	4905
MEMBRANE REPOLARIZATION DURING ACTION POTENTIAL%GOBP%GO:0086011	0.5824317	1.6356848	16	1	4165
NEGATIVE REGULATION OF RELEASE OF CYTOCHROME C FROM MITOCHONDRIA%GOBP%GO:0090201	0.58499587	1.6339521	16	1	2885
APOPTOTIC CLEAVAGE OF CELLULAR PROTEINS%REACTOME%R-HSA-111465.3	0.52322435	1.6278474	34	1	3356
IMMUNOREGULATORY INTERACTIONS BETWEEN A LYMPHOID AND A NON-LYMPHOID CELL%REACTOME%R-HSA-198933.6	0.4888515	1.6273456	51	1	6549
NEGATIVE REGULATION OF COAGULATION%GOBP%GO:0050819	0.52278864	1.6272141	29	1	2064
RELAXATION OF MUSCLE%GOBP%GO:0090075	0.5973013	1.6261554	15	1	7006
NEGATIVE REGULATION OF LEUKOCYTE MIGRATION%GOBP%GO:0002686	0.5156373	1.6001692	31	1	6104

COMPLEMENT AND COAGULATION CASCADES%WIKIPATHWAYS_20210110%WP558%HOMO SAPIENS	0.51096547	1.5988224	32	1	3934
PID_UPA_UPAR_PATHWAY%MSIGDB_C2%PID_UPA_UPAR_PATHWAY	0.5644022	1.5974187	19	1	2466
HOMOTYPIC CELL-CELL ADHESION%GOBP%GO:0034109	0.4910166	1.5916495	39	1	5295
HALLMARK_NOTCH_SIGNALING%MSIGDB_C2%HALLMARK_NOTCH_SIGNALING	0.5442772	1.5904169	21	1	357
HIPPO SIGNALING%GOBP%GO:0035329	0.5224672	1.5894673	26	1	4685
ENDOTHELIN PATHWAYS%WIKIPATHWAYS_20210110%WP2197%HOMO SAPIENS	0.52423495	1.5892973	25	1	5524
CELL JUNCTION MAINTENANCE%GOBP%GO:0034331	0.570698	1.5847397	15	1	5877
REGULATION OF MITOCHONDRIAL OUTER MEMBRANE PERMEABILIZATION INVOLVED IN APOPTOTIC SIGNALING PATHWAY%GOBP%GO:1901028	0.50062716	1.5840766	41	1	4317
T CELL ACTIVATION INVOLVED IN IMMUNE RESPONSE%GOBP%GO:0002286	0.54420745	1.5840503	20	1	1876
PYRIMIDINE-CONTAINING COMPOUND CATABOLIC PROCESS%GOBP%GO:0072529	0.5064638	1.5678614	31	1	4743
NEGATIVE REGULATION OF MITOCHONDRION ORGANIZATION%GOBP%GO:0010823	0.49555945	1.5655676	39	1	2885
PID_RHOA_REG_PATHWAY%MSIGDB_C2%PID_RHOA_REG_PATHWAY	0.49877688	1.5646584	36	1	4426
